# Genome-Wide Approach to Identify Quantitative Trait Loci for Drought Tolerance in Tetraploid Potato (*Solanum tuberosum* L.)

**DOI:** 10.3390/ijms22116123

**Published:** 2021-06-07

**Authors:** Christina Schumacher, Susanne Thümecke, Florian Schilling, Karin Köhl, Joachim Kopka, Heike Sprenger, Dirk Karl Hincha, Dirk Walther, Sylvia Seddig, Rolf Peters, Ellen Zuther, Manuela Haas, Renate Horn

**Affiliations:** 1 Department of Plant Genetics, Institute of Biological Sciences, University of Rostock, Albert-Einstein-Str. 3, 18059 Rostock, Germany; christina.schumacher2017@outlook.com (C.S.); susanne.thuemecke@uni-rostock.de (S.T.); florian.schilling@uni-rostock.de (F.S.); 2 Max Planck Institute of Molecular Plant Physiology, Am Mühlenberg 1, 14476 Potsdam, Germany; koehl@mpimp-golm.mpg.de (K.K.); kopka@mpimp-golm.mpg.de (J.K.); Heike.Sprenger@bfr.bund.de (H.S.); Hincha@mpimp-golm.mpg.de (D.K.H.); Walther@mpimp-golm.mpg.de (D.W.); Zuther@mpimp-golm.mpg.de (E.Z.); manuela.haas@mluk.brandenburg.de (M.H.); 3 Institute for Resistance Research and Stress Tolerance, Julius-Kühn Institut, Federal Research Centre for Cultivated Plants, Rudolf-Schick-Platz 3, 18190 Sanitz, Germany; sylvia.seddig@julius-kuehn.de; 4 Landwirtschaftskammer Niedersachsen, Dethlingen 14, 29633 Munster, Germany; peters@docpotato.de

**Keywords:** drought tolerance, ethylene, brassinosteroids, cell wall, tuber starch content, tuber starch yield, metabolites, transcript markers, potato, *Solanum tuberosum*

## Abstract

Drought represents a major abiotic stress factor negatively affecting growth, yield and tuber quality of potatoes. Quantitative trait locus (QTL) analyses were performed in cultivated potatoes for drought tolerance index DRYM (deviation of relative starch yield from the experimental median), tuber starch content, tuber starch yield, tuber fresh weight, selected transcripts and metabolites under control and drought stress conditions. Eight genomic regions of major interest for drought tolerance were identified, three representing standalone DRYM QTL. Candidate genes, e.g., from signaling pathways for ethylene, abscisic acid and brassinosteroids, and genes encoding cell wall remodeling enzymes were identified within DRYM QTL. Co-localizations of DRYM QTL and QTL for tuber starch content, tuber starch yield and tuber fresh weight with underlying genes of the carbohydrate metabolism were observed. Overlaps of DRYM QTL with metabolite QTL for ribitol or galactinol may indicate trade-offs between starch and compatible solute biosynthesis. Expression QTL confirmed the drought stress relevance of selected transcripts by overlaps with DRYM QTL. Bulked segregant analyses combined with next-generation sequencing (BSAseq) were used to identify mutations in genes under the DRYM QTL on linkage group 3. Future analyses of identified genes for drought tolerance will give a better insight into drought tolerance in potatoes.

## 1. Introduction

Facing global climate change and increasing demands for food production, the improvement of crop yields under limited water supply conditions and other abiotic stresses such as heat or salinity is vital for mankind [1,2,3,4]. Water deficit represents one of the major limiting factors in crop production. As increased irrigation is not only expensive but also ecologically detrimental, improving the plant’s own mechanisms to cope with abiotic and biotic stresses is a favorable approach [5]. Identification of molecular factors underlying stress-response mechanisms represents a prerequisite for effective breeding of tolerant plants [6].

The access to a fully sequenced potato genome [7] and the rapidly developing “omics” technologies make it possible to screen for known and to identify novel stress-related factors in tetraploid potato cultivars [8,9]. Recent “omics” approaches to study drought stress response in potatoes have been performed on transcript and metabolite levels [10,11,12,13,14], transcription factors [15] and heat shock proteins [16]. Mapping of quantitative trait loci (QTL) to identify genomic regions associated with drought [17,18], foliar symptoms caused by potato virus Y [19], cold sweetening [20], tuber starch content and tuber starch yield [21], and resistance to pathogens [22] has been successfully applied in potatoes to locate candidate genes. Commonly known candidates associated with abiotic stress response regulation in plants are phytohormones such as abscisic acid (ABA), ethylene and jasmonic acid (JA)—recently accompanied by gibberellic acid (GA), but also reactive oxygen species (ROS), transcription factors (e.g., ethylene response factors, dehydration responsive element binding (DREB) proteins, zinc finger proteins (ZNF, WRKY, MYB)) and regulatory protein kinases such as mitogen-activated protein kinases (MAPKs) and receptor protein kinases [6,23].

Drought stress induces a broad range of molecular response mechanisms with potentially both positive and negative effects on plant yield [5,24,25]. As a result, the improvement of stress tolerance in crops bears the risk of yield penalties [26].

Various indices to evaluate drought stress tolerance have been developed [27], including the stress susceptibility index (SSI) [28], geometric mean productivity (GMP), stress tolerance index (STI) [29], tolerance index (TOL), harmonic mean (HARM) [30], yield stability index (YSI) [31] and deviation of relative starch yield (DRYM) [32]. Comparing the power of these indices to distinguish genotypes of contrasting yield stability independent of the yield potential, DRYM outperformed SSI, STI and GMP [32]. While most indices are mainly based on yield losses under drought conditions, the DRYM index allows separation of drought tolerance from yield potential. Nevertheless, a significant, negative correlation between drought stress tolerance and starch yield under none-stressed conditions was found, indicating a yield penalty for drought-tolerant potato cultivars [32]. To elucidate the mechanism responsible for this observed correlation, a separation of drought tolerance and yield parameters will be essential.

In this study, we analyzed drought tolerance (DRYM index), yield (tuber starch content, tuber starch yield, tuber fresh weight), transcript and metabolite data by mapping QTL in cultivated tetraploid potatoes in order to identify trait-relevant genomic regions. QTL analyses allowed the allocation of potentially relevant genomic loci for drought tolerance, but, in a number of cases, it also revealed significant overlaps with QTL for tuber starch content, tuber starch yield and tuber fresh weight. The combined use of QTL mapping and the potato reference genome [7] provided the possibility to identify candidate genes underlying QTL loci, given that adjacent microsatellite markers can be used to determine the exact QTL position in the genome. In addition, detailed genomic analyses were performed for the DRYM QTL on linkage group (LG) 3 by combining bulked segregant analyses with next-generation sequencing (BSAseq). Data from drought-sensitive and drought-tolerant bulks, as well as from the parental varieties, Albatros and Ramses, were evaluated for mutations relevant for drought tolerance. By focusing on genomic regions relevant for drought stress, we identified a number of potential candidate genes for drought tolerance within these regions that can now be further analyzed to eventually help to discriminate between drought-tolerant and -sensitive cultivars in potato breeding programs.

## 2. Results

### 2.1. Informative Markers for Defining Linkage Groups in Starch Potato Cultivars

**Figure 1:**
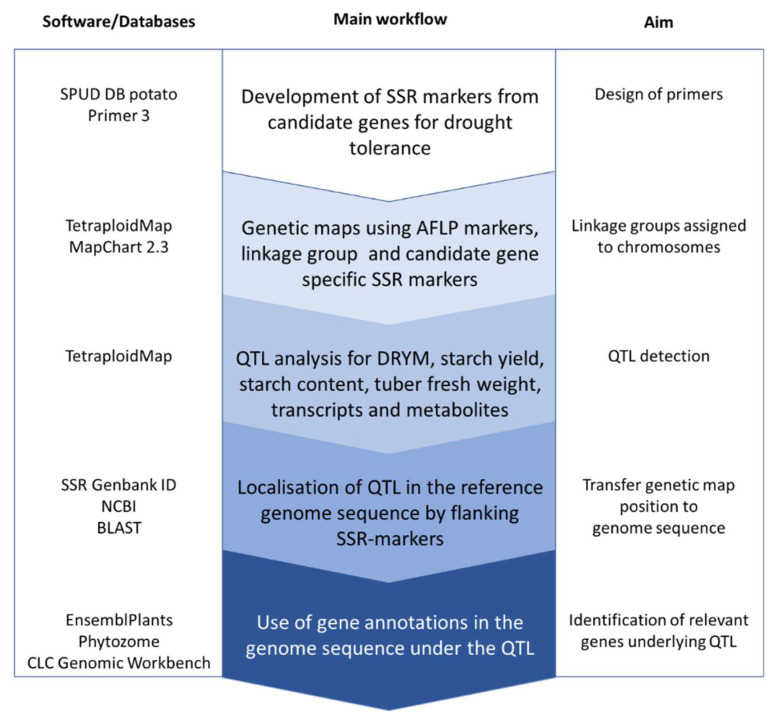
Flow scheme to identify candidate genes underlying QTL using the potato reference genome sequence.

In order to identify informative markers for genetic mapping, amplified fragment length polymorphic (AFLP) marker and simple sequence repeat (SSR) marker analyses were performed in the F_1_ population representing a cross of the drought-tolerant cultivar Albatros (A) and the drought-sensitive cultivar Ramses (R). Linkage group specific SSR-markers [33] and SSR-markers derived from candidate genes for drought tolerance with known locations in the potato reference genome [34] were used to assign the genetic maps to the potato chromosomes. In total, 73 SSR-markers and additional 505 AFLP-markers with informative segregation ratios could be mapped in the F_1_ A × R, resulting in separate genetic maps for both parents covering 1061.9 cM (Albatros) and 1024.4 cM (Ramses), respectively (overall linkage groups for each cultivar are given in Figure S1, distribution of markers on the homoeologous chromosomes in Figures S2–S5). On average, each overall linkage group carried four to five SSR-markers, varying between two and eight SSR-markers per linkage group. Mapping of SSR-markers allowed the assignment of our linkage groups to the potato reference genetic map [33]. Based on the genetic maps for both parents, QTL analyses were performed to identify genomic regions and underlying candidate genes relevant for drought tolerance (Figure 1).

### 2.2. QTL Mapping Links Drought Tolerance Index DRYM to Starch Yield Parameters

**Figure 2:**
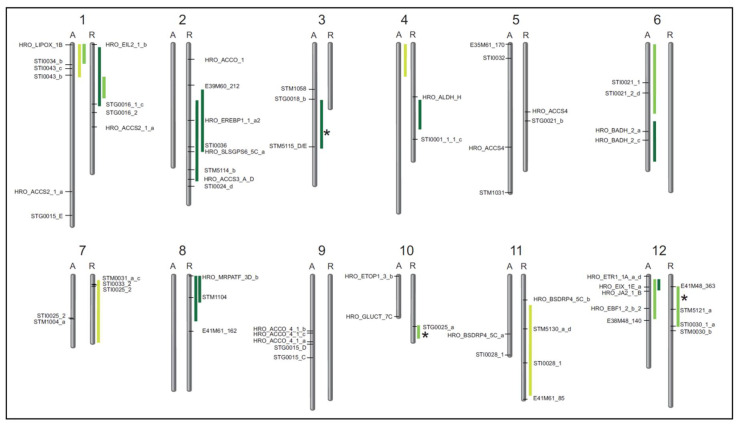
Schematic overview of DRYM QTL mapped in cultivated potato. QTL for the DRYM index (95% confidence interval) mark the genomic regions associated with drought tolerance on the genetic maps of each parental chromosome (1–12) in the drought-tolerant parent Albatros (A, **left**) and in the drought-sensitive parent Ramses (R, **right**). Most DRYM QTL (2014, light green; 2015, green; 2016, dark green) co-localized with tuber starch yield QTL (Table 1), and only few DRYM QTL (asterisks) did not. Microsatellite markers (descriptions in Tables S1 and S2) and AFLP markers within or flanking QTL regions are shown.

For the QTL analyses, data for drought tolerance (DRYM index) and yield data (tuber starch yield, tuber starch content, tuber fresh weight) collected from two locations with five different drought test systems over three years (2014–2016), and with a sixth drought stress scenario at a third location over two years, under control (Co) and drought stress (Ds) conditions were used. QTL analyses were performed separately for treatments, locations and years. QTL mapping resulted in 19 DRYM QTL, with eight located on chromosomes of the drought-tolerant parent Albatros (A) and 11 on chromosomes of the drought-sensitive parent Ramses (R) (Figure 2). In total, DRYM QTL were detected on 10 of the 12 chromosomes in the A × R population, either on one parental linkage group or on both. Only on chromosome 5 and chromosome 9 were no DRYM QTL mapped on either parental linkage group.

Agreements between DRYM QTL for two different years could be found on LG1 (A, 2014, 2015), LG1 (R, 2015 and 2016) and on LG12 (A, 2015, 2016). Moreover, QTL on LG2 (R) and LG8 (R) both overlapped for two locations in the same year (2016). However, about half of the DRYM QTL mapped on individual linkage groups and resulted from data of a single year each, with no corresponding QTL in the other two investigated years (Figure 2), reflecting potential interactions between drought tolerance and other environmental parameters (e.g., temperature) in different years.

Most QTL for tuber starch content, tuber starch yield and tuber fresh weight co-localized with DRYM QTL (Table 1). The DRYM QTL (green) mapping on the respective parental linkage groups under control (Co) and drought stress (Ds) conditions are listed together with QTL for tuber starch content (starch_g_per_kg), tuber starch yield (starch_yield_g_per_plant) and tuber fresh weight (tuber_FW_kg_per_plant). Co-localizing QTL are highlighted in green, and non-overlapping QTL neighboring DRYM QTL are shown in grey.

The population displayed 12 tuber starch QTL (including starch content and starch yield) under drought stress and 17 under control conditions overlapping with 11 DRYM QTL (Table 1).

In addition, four mean QTL (MW) for starch content (one for both drought and control conditions, on LG7 (R) and LG11 (R), respectively) co-localized with DRYM QTL. After normalization of tuber starch yield to account for spatial effects, two tuber starch yield QTL under drought stress (LG2 (R) and LG8 (R)) were also overlapping with DRYM QTL (Table 1). In addition, QTL for tuber fresh weight and tuber starch parameters usually overlapped. Hence, 14 QTL for tuber fresh weight (6 QTL under drought, and 8 QTL under control conditions) also co-localized with 12 DRYM QTL. Two additional mean QTL (MW) for tuber fresh weight were observed on LG2 (R) under DRYM QTL. Only three DRYM QTL on the linkage groups LG3 (A), LG10 (R) and LG12 (R) did not directly overlap with any tuber starch content, tuber starch yield or tuber fresh weight QTL (Figure 2, marked by asterisks; Table 1).

### 2.3. Carbohydrate Metabolism Candidate Genes in QTL Regions

Focusing on five major overlapping regions of DRYM QTL and QTL for yield parameters (tuber starch content, tuber starch yield and tuber fresh weight), we were interested in genes annotated in the potato reference genome underlying these yield-related QTL. SSR-markers flanking the QTL were used to localize the relevant areas in the potato genome sequence. The genomic regions covered approximately 87 Mb, with 10.9 Mb on LG2 (R), 36.76 Mb on LG7 (R), 1.3 Mb on LG8 (R), 36.3 Mb on LG11 (R) and 1.74 Mb on LG12 (A) (Figure S6, Table 2). The presence of genes involved in carbohydrate metabolism [35,36,37] within these genomic regions could be a first indication for a linkage between starch and sucrose metabolism and drought tolerance.

Genomic regions of DRYM QTL overlapping with tuber starch QTL (starch content, starch yield) revealed the presence of genes encoding enzymes of starch metabolism such as soluble starch synthase 3 (*SS3*, PGSC0003DMG400016481), starch synthase V (*SS5*, PGSC0003DMG400030619), protein targeting to starch (*PTST*, PGSC0003DMG400030609) and adjoining starch synthase IV (*SS4*, PGSC0003DMG400008322) on LG2 (R); a de-branching enzyme (*DBE*) and a SEX4-like phosphatase (PGSC0003DMG400027327) on LG11 (R); as well as glucose-1-phosphate adenylyltransferase (*AGP*, PGSC0003DMG400046891) on LG12 (A) (Table 2). In addition, the SSR-marker STM1104 for granule-bound starch synthesis (*WAXY*, PGSC0003DMG400012111) was detected within a DRYM QTL on LG8 (R) (Figure 2).

Furthermore, candidate genes involved in sucrose metabolism were present (Table 2). Pyrophosphate-fructose-6-phosphate 1-phosphotransferase subunit beta (*PFP-BETA*, PGSC0003DMG400016726) is localized on LG2 (R) under a combined DRYM/tuber starch QTL. On LG7 (R), underneath the wide-spanning DRYM QTL, sucrose-phosphate-synthase (*SPS*, PGSC0003DMG400027936) and sucrose synthase 2 (*SUS II*, PGSC0003DMG400013546) were present. Moreover, LG11 (R) revealed a co-localization of a DRYM QTL with a trans-aldolase (*TAL1*, PGSC0003DMG402028027) and two membrane-associated transporters, namely adenylate transporter (*ANT*, PGSC0003DMG400013596) and sucrose transporter 1 (*SUT1*, PGSC0003DMG400009213).

We also observed yield-related QTL, which did not overlap with DRYM QTL. The most prominent QTL region on LG5 displayed five QTL (2015 and 2016) for tuber starch content (three under drought stress and two for control conditions) and two for tuber starch yield (one for drought stress and control conditions, respectively), and a mean QTL for tuber starch content (one under control and drought stress conditions, respectively) (Figure S6). In this proximal region of LG 5 (A), genes for alpha-1,4 glucan phosphorylase L-2 isozyme (PGSC0003DMG400028382) and for fructose-biphosphate aldolase (PGSC0003DMG400030565) are annotated in the potato reference genome [7]. In the distal area of LG 5 (R), another group of QTL for tuber starch content (two under drought stress in 2015, one in 2016 and one under control also in 2015) and one for tuber starch yield were detectable. In addition, two mean QTL for tuber starch content, one under drought and one under control condition, were located in the area.

### 2.4. Ethylene Synthesis and Other Stress-Related Factors Co-Localize with DRYM QTL

Genetic mapping displayed co-localization of candidate gene derived SSR-markers for ethylene biosynthesis and ethylene signaling with DRYM QTL. On LG1 (R), the DRYM QTL of 2016 is flanked by the marker HRO_EIL2_1 derived from ethylene insensitive-like 2 (*EIL2*, PGSC0003DMG400008712). On LG2 (R), a DRYM QTL was found within the same genomic region as a marker for 1-aminocyclopropane-1-carboxylate synthase (ACS), an essential enzyme of the ethylene biosynthesis (HRO_ACCS3, PGSC0003DMG400021426, Figure 2, Table 3). In addition, a marker for ethylene responsive element binding protein 1 (HRO_EREBP1, PGSC0003DMG400029713) was present in the same region.

On LG12 (A), the two DRYM QTL (2015, 2016) co-localized with a marker (HRO_EIX_1E) for ethylene-inducing xylanase (*EIX*, PGSC0003DMG400007876), while only the DRYM QTL for 2015 additionally overlaps with markers for EIN3 (ethylene-insensitive3)-binding F-box protein 1 (HRO_EBF1_2, PGSC0003DMG400002914) and jasmonic acid 2 (HRO_JA2, PGSC0003DMG400015342) (Figure 2, Table 3). Both DRYM QTL are flanked on the proximal side by the marker HRO_ETR1_1A_a_d representing the ethylene receptor 1 (*ETR1*, PGSC0003DMG400007843). The SSR-marker STM5121 within the stand-alone DRYM QTL on LG12 (R) is assigned to a gene of unknown function (PGSC0003DMG400000292). Furthermore, a DRYM QTL co-localizing with a QTL for tuber fresh weight on LG1 (A) displayed co-localization with a JA biosynthesis associated lipoxygenase (HRO_LIPOX_1B, PGSC0003DMG400032207) and markers for a zinc finger protein (STI0043, PGSC0003DMG400016379) and a fasciclin-like arabinogalactan protein (STI0034, PGSC0003DMG400021372) (Figure 2, Table 3).

Interestingly, the SSR-marker STG0016_1_c corresponding to the gene for a chromo domain carrying protein, LIKE HETEROCHROMATIN PROTEIN 1 (*LHP1*, PGSC0003DMG400031112) mapped under the DRYM QTL on LG1 of the other parent (R) in the adjacent region. The SSR-marker derived from an aldehyde dehydrogenase (HRO_ALDH_H, PGSC0003 DMG400034597) mapped in the flank of the DRYM QTL on LG4 (R).

In addition, other candidate gene derived SSR-markers associated with stress signaling are physically located within DRYM QTL regions (Figure 2, Table 3), e.g., on LG2 (R), a marker for a disease resistance response protein (STM5114, PGSC0003DMG403001521). Both LG6 (A) and LG7 (R) displayed markers for heat stress transcription factors (STI0021, PGSC0003DMG400016270 and STI0033, PGSC0003DMG400032793) within genomic locations of DRYM QTL. Moreover, a marker for bacterial spot disease resistance protein 4 (HRO_BSDRP4, PGSC0003DMG400002427), identified as one of 20 transcript markers for drought tolerance [14], can be detected adjacent to a DRYM QTL area on LG11 (R).

### 2.5. Standalone QTL on LG3 Includes Cytochrome P450, Cell Wall Remodeling Genes and Phytohormone Signaling Factors

As proof of concept that our approach from QTL analyses towards genome analyses (Figure 1) works for the identification of genes relevant for drought tolerance, the DRYM QTL on LG3 was selected for further analyses. This DRYM QTL represents one of the three standalone DRYM QTL, which are the most interesting ones for drought tolerance because the drought response is not linked to tuber starch content or tuber starch yield QTL. In addition, the standalone QTL on LG3 (A) is directly flanked by two SSR-markers STG0018_b (glutamine-rich protein, PGSC0003DMG400026490) and STM5115_D/E (glycerol kinase, PGSC0003DMG400014144), which is ideal for further analyses on the genomic level, as the genome positions of the SSR-markers are known (Table S1 [33]).

The DRYM QTL on LG3 spans approximately 10 Mb (47,424,062–57,312,556 bp) in the reference potato genome assembly SolTub 4.03 (Figure 2, Table S3). In total, 916 genes are annotated in this region including genes coding for several cytochrome P450, kinases (receptor-like kinases, MAPKs), transcription factors (AP2, BHLH, C2H2L, ERF, MYB, NAC, WRKY, Zinc finger), aquaporins and DEHYDRATION-INDUCED 19 homolog 6. There are also genes for biosynthesis of carbohydrates (raffinose synthase 2, sucrose synthase), flavonoids (chalcone reductase, cinnamoyl CoA reductase, flavonol synthase/flavanone 3-hydroxylase) and proteins (elongation factor TuA, chloroplastic, eukaryotic translation initiation factor 5A-5, prolyl-tRNA synthetase, ribosomal proteins, signal recognition particle 54 kDa protein 1) present in this region, but also genes encoding for DNA repair enzymes (DNA-3-methyladenine glycosylase, excision repair cross-complementing 1 ercc1, protein kinase atmrk1). In addition, a number of genes encoding proteins involved in cell wall remodeling such as expansin, fasciclin-like arabinogalactan protein 9, pectinesterase, pectinesterase inhibitor, pectate lyase and xyloglucan endotransglucosylase/hydrolase protein 9 are annotated in this region. Genes involved in phytohormone signaling (BRASSINOSTEROID INSENSITIVE 1-associated receptor kinase 1, DELLA protein RGL1, gibberellin receptor GID1, PP2C) also reside within this standalone DRYM QTL on LG3. In addition, 141 conserved genes of unknown function are underneath the QTL. Interestingly, despite the fact that no QTL for tuber starch content or tuber starch yield were mapped in our biparental population in this region, two genes important for starch biosynthesis were annotated in the region defined by this DRYM QTL: sucrose synthase (PGSC0003DMG400031046) and sucrose transporter (PGSC0003DMG400024489).

In order to further reduce the number of potential candidate genes in this area, next-generation sequencing (NGS) data of a drought-tolerant and a drought-sensitive bulk of A × R were compared with each other. As the bulks consisted of the most tolerant and the most sensitive F_1_ individuals of the cross A × R, detected differences between them are likely to be related to drought tolerance. The comparison revealed 25,205 SNPs differentiating the two bulks under this DRYM QTL (Table S4). An overview of the different types of mutations detected in this area of LG3 distinguishing the bulks is given in Figure S7. Only 45 genes of the 916 genes showed no differences (including sucrose transporter, PGSC0003DMG400024489), and 11 genes carried only synonymous mutations between the drought-tolerant and -sensitive bulk. Sucrose synthase (PGSC0003DMG400031046) showed one missense mutation and 9 SNPs in the 3′UTR region in comparison to the drought-sensitive bulk. Missense mutations between the bulks were also found in both genes encoding raffinose synthase 2 (PGSC0003DMG400037864, PGSC0003DMG400018109) located under the DRYM QTL. In addition, raffinose synthase 2 (PGSC0003DMG400018109) showed SNPs in the 3′UTR and the upstream region. The presence of O-methyltransferase (PGSC0003DMG400012024), which shows one missense mutation and five additional SNPs in the 3′UTR, was supported by three eQTL for O-methyltransferase (both control and drought conditions in 2014) overlapping with the DRYM QTL.

**Figure 3:**
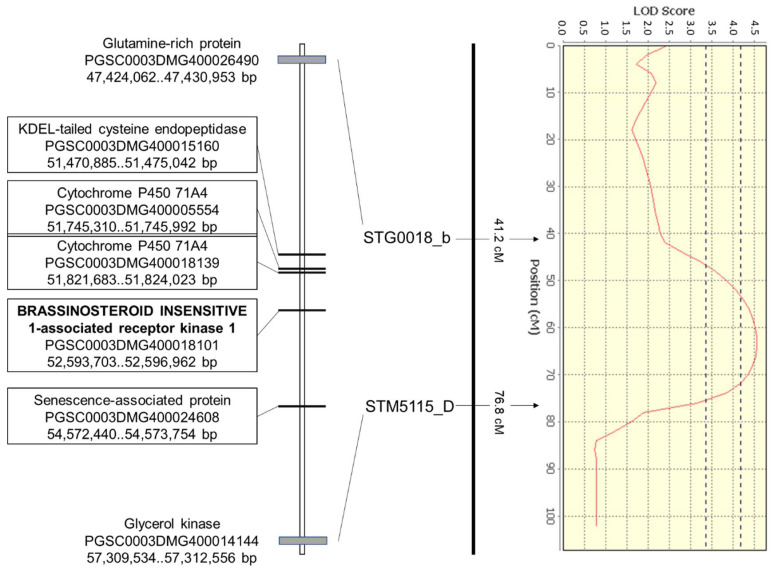
Region under the standalone DRYM QTL on LG3 (A) flanked by the SSR-markers STG0018_b and STM5115_D. Apart from positions of the flanking genes, the locations of five genes in the central region of the DRYM QTL carrying nonsense mutations are shown as annotated in SolTub v4.03.

Interestingly, 63 genes showed SNPs resulting in stop codons (Table S4). These nonsense mutations were observed, for example, in genes encoding BRASSINOSTEROID INSENSITIVE 1-associated receptor kinase 1 (*BAK1*, PGSC0003DMG400018101), cytochrome P450 71A4 (PGSC0003DMG400018139, PGSC0003DMG400005554), senescence associated protein (PGSC0003DMG400024608) and KDEL-tailed cysteine endopeptidase (PGSC0003DMG400015160) (Figure 3). *BAK1* (PGSC0003DMG400018101), which is centrally located under the DRYM QTL (ST4.03ch03 52,593,703..52,596,962), shows a premature stop codon in the drought-sensitive cultivar. This SNP, leading to a loss-of-function mutation, is otherwise only present in the drought-sensitive bulk, but not in the drought-tolerant bulk. A second paralog of *BAK1* (PGSC0003DMG400025330, ST4.03ch03 53,228,956..53,233,993), which is also present in this QTL region on LG3, shows one splice mutation, two missense mutations and a number of mutations in the upstream area as well as 5′- and 3′-UTR regions. Both Cytochrome P450 71A4 genes (PGSC0003DMG400018139 and PGSC0003DMG400005554) located adjacent to *BAK1* (ST4.03ch03 51,821,683..51,824,023 and ST4.03ch03 51,745,310..51,745,992) also contain SNP-derived premature stop codons.

The fourth gene in the central area of the DRYM QTL showing a nonsense mutation is KDEL-tailed cysteine endopeptidase (PGSC0003DMG400015160) at ST4.03ch03 51,470,885..51,475,042. The senescence associated protein (PGSC0003DMG400024608) carrying a stop codon is located at the end of the DRYM QTL.

Apart from the two *BAK1* genes, seven other genes involved in phytohormone signaling are present in the standalone DRYM QTL on LG3 (Table 4). With the exception of *ERF* (PGSC003DMG400014196, no mutation), all genes showed at least one missense mutation (Table S4).

Fourteen genes involved in cell wall stability and flexibility are located under the DRYM QTL on LG3 (Table 5). These genes encode fasciclin-like arabinogalactan protein 9, pectinesterase, pectinesterase inhibitors, pectate lyases, protein COBRA, COBRA3, xyloglucan endotransglucosylase/hydrolase protein 9 and expansins. Seven of the genes are located in the central area of the standalone DRYM QTL on LG3, but only five of them carry missense mutations. These mutations are present in genes encoding three pectinesterase inhibitors (PGSC0003DMG400040957, PGSC0003DMG400034620, PGSC0003DMG400018189), fasciclin-like arabinogalactan protein 9 (PGSC0003DMG400018093) and pectate lyase (PGSC0003DMG400018142) (Table S4).

### 2.6. Expression QTL for Drought Transcript Markers Overlap with DRYM QTL

Expression QTL (eQTL) mapping of 43 selected transcripts (Table S5), previously identified as marker candidates in a model predicting drought tolerance [14] and used in marker-assisted selection [38], displayed various co-localizations with DRYM QTL (Figure S6, Table S6). Most frequent overlaps included ethylene-inducing xylanase (*EIX*, PGSC0003DMT400020366) with a total of 10 eQTL on LG3 (A), LG4 (R) and LG12 (A and R) and bacterial spot disease resistance protein 4 (*BSDRP4*, PGSC0003DMT400080813) with a total of six eQTL distributed on LG7 (R), LG11 (R) and LG12 (A). Additional frequent overlaps with DRYM QTL included eQTL (five each) for acidic class II 1,3-beta-glucanase (PGSC0003DMT400027201), cytochrome P450 (PGSC0003DMT400008547), desacetoxyvindoline 4-hydrolase (PGSC0003DMT400041989), o-methyltransferase (PGSC0003DMT400031370), reticuline oxidase (PGSC0003DMT400046308) and UDP-glucose:glucosyltransferase (PGSC0003DMT400049125) (Figure S6, Table S6).

Overlaps of four eQTL with DRYM QTL were observed for nine transcripts, e.g., beta-D-glucan exohydrolase (PGSC0003DMT400015224), cc-nbs-lrr resistance protein (PGSC0003DMT400049097), lipoxygenase (*LOX*, PGSC0003DMT400082023), poly (ADP-ribose) glycohydrolase (PGSC0003DMT400075512) and (S)-norcoclaurine synthase (PGSC0003DMT400008278).

On LG3 (A), the standalone DRYM QTL fell together with three eQTL for o-methyltransferase (PGSC0003DMT400031370, two under control and one under drought stress conditions), two eQTL each for reticuline oxidase and ethylene-inducing xylanase (one under control and one under drought conditions, respectively) and one eQTL for poly (ADP-ribose) glycohydrolase (PGSC0003DMT400075512, under control conditions). Interestingly, only a single eQTL was mapped on LG10 (R) and co-localized with the second standalone DRYM QTL. This eQTL was for fatty acid desaturase (PGSC0003DMT400083859), which also showed an overlap with another DRYM QTL on LG11 (R). The third standalone DRYM QTL on LG12 (R) mapped together with 14 eQTL, including, e.g., ethylene-inducing xylanase (PGSC0003DMT400020366), glycosyltransferase (PGSC0003DMT400021019), two nbs-lrr resistance proteins (PGSC0003DMT400035714, PGSC0003DMT400049097), TMV (tobacco mosaic virus) resistance protein N (PGSC0003DMT400046899) and NADPH-dependent codeinone reductase (PGSC0003DMT400037483). Most eQTL spanned larger genomic regions (Figure S6). Consequently, 40 of the 43 transcripts revealed at least one eQTL overlapping with DRYM QTL.

### 2.7. QTL of Drought-Responsive Metabolites Overlap with DRYM QTL

The analysis of metabolite QTL (mQTL) was performed for 15 metabolites (chlorogenic acid (i.e., quinic acid, 3-caffeoyl-, trans-), fumaric acid, galactaric acid, galactinol, malic acid, raffinose, ribitol, salicylic acid-glucopyranoside, threonic acid and six unknown metabolites) out of 36 that had previously been identified as drought responsive (*p* < 0.01) in at least one out of four European reference cultivars under field conditions (Ref. [13], Table S7). These 15 metabolites resulted from filtering with the requirement of at least 90% of data points to be present in both locations and treatments. Altogether, 47 mQTL for Albatros and 50 for Ramses were identified with most of them located on LG1 (8) and LG12 (9) for Albatros and on LG1 (9) and LG9 (8) for Ramses (Figure S6). Thirty-one mQTL overlapped with DRYM QTL, on average two mQTL with one DRYM QTL (Figure S6, Table S8). For ribitol, a maximum of six mQTL co-localized with DRYM QTL on LG1 (A) (two under drought and two under control conditions) and on LG1 (R) (one for drought and one for control). For the unidentified metabolite A27004-101, four overlaps of mQTL with DRYM QTL could be discovered, which were located on LG1 (R) and LG8 (R), each with one mQTL under drought stress and LG7 (R), with one mQTL for drought and control conditions. Four mQTL overlapping with DRYM QTL were mapped for fumaric acid, one each under control conditions on LG1 (A), LG2 (R) and LG8 (R), and one under drought stress on LG11 (R). For salicylic acid-glucopyranoside, one mQTL each under drought conditions was found together with DRYM QTL on LG2 (R), LG3 (A) and LG12 (A). The fourth mQTL for salicylic acid-glucopyranoside also mapped with a DRYM QTL on LG2 (R), but under control conditions. Both ribitol and fumaric acid belong to a group of 24 metabolites with high predictive values for identifying drought-tolerant varieties from a panel of 31 potato cultivars [14].

Additional DRYM QTL overlaps with three mQTL were identified for malic acid and the unidentified metabolites A197007-101; overlaps with two mQTL for galactinol, A237001-101 and chlorogenic acid (Table S8). Ultimately, 11 of the 15 selected candidate metabolites (exceptions are the three unidentified metabolites A148006-101, A174001-101 and A250002-101 as well as raffinose) displayed at least one mQTL overlapping with DRYM QTL. Thus, previous approaches to predict potato drought tolerance with a group of metabolites have been validated by discovering overlaps of DRYM QTL with mQTL of previously reported predictive metabolites [14].

## 3. Discussion

### 3.1. Abiotic vs. Biotic Stress Response under Drought

Our QTL analyses confirmed the association of common abiotic stress responses with drought tolerance in potatoes. Markers flanking or overlapping DRYM QTL were derived, e.g., from heat stress transcription factors (STI0021, PGSC0003DMG400016270; STI0033, PGSC0003DMG400032793) and enzymes of ethylene biosynthesis such as 1-aminocyclopropane 1-carboxylate synthase 3 (*ACS3*, PGSC0003DMG400021426). ACS facilitates conversion of S-adenosyl methionine (S-AdoMet) to ethylene via 1-aminocyclopropane-1-carboxylic acid (ACC) as an intermediate, which is oxidized by ACC oxidase (ACO) [39]. Microsatellite analyses in a potato association panel detected allelic differences for ACS3 associated with drought sensitivity [34]. Biosynthesis of ethylene has been frequently cited to play a major role in stress response regulation in plants [23,26,40]. Furthermore, markers underlying DRYM QTL in our study belonged to the ethylene signaling pathway such as, e.g., EIN3-binding F-box protein 1 (*EBF1*, PGSC0003DMG400002914, LG12) and ethylene responsive element binding protein 1 (*EREBP1*, PGSC0003DMG400029713, LG2). In the absence of ethylene, EIN3 is negatively regulated by EIN3-binding F-box protein 1 (EBF1), which helps binding of EIN3 to the SCF complex for ubiquitination and thereby targets EIN3 for degradation via 26S-proteasome [41]. EIN3 and EIL1 act together downstream of EIN2 as part of the ethylene signaling pathway [41] and represent major transcription factors for the ethylene-dependent gene expression [42]. In the presence of ethylene, EIN2 is involved in the proteolysis of EBF1 and EBF2, thereby allowing the accumulation of EIN3 [43]. Ethylene responsive element binding protein 1 (*EREBP1*, PGSC0003DMG400029713) shows the highest coverage (82%) with ethylene-responsive transcription factor 9 (ERF9) in *A. thaliana* with 69.4% similarity (SPUD DB). Two additional ERF genes (PGSC0003DMG400024606, PGSC0003DMG400014196) and Ethylene-responsive element-binding family protein (PGSC0003DMG400010135) underlie the standalone DRYM QTL on LG3 (A). ERFs represent plant-specific transcription factors involved in the ethylene-controlled gene expression [44,45].

Taken together, ethylene biosynthesis and signaling seem to play a major role in drought sensitivity in our population A × R. Among its diverse roles from germination to growth, ethylene is also known to be involved in senescence of plant organs and the abscission of leaves [46,47,48,49], which negatively influences photosynthesis rates. Apart from high photosynthesis rates, total tuber yield is also dependent on the ability of the potato plants to form a completely closed canopy covering the ground throughout the growing season [50]. Leaf wilting or early leaf fall due to drought would lead to yield losses not only due to reduced light absorption and less photosynthesis, but also because a reduced canopy closure negatively affects tuber growth.

Ethylene biosynthesis is also part of an antagonistic crosstalk regulation of ethylene and nitric oxide (NO) induced abscisic acid (ABA) signaling that plays a major role in stomatal closure, counteracting water loss under drought [51].

Another player in drought tolerance seems to be jasmonic acid. The SSR-marker derived from jasmonic acid 2 (HRO_JA2_1_B, PGSC0003DMG400015342) as well as SSR-marker and eQTL for lipoxygenase (HRO_LIPOX_1B, PGSC0003DMT400082023) overlapped with DRYM QTL. Lipoxygenases catalyze the oxygenation of fatty acids to oxylipins such as jasmonic acid [52]. Similar to ethylene, jasmonic acid is known to be involved in plant seed germination, growth, senescence, stomatal aperture and stress response [48,53,54]. In addition, dehydrin genes are induced by signaling molecules such as ABA, Me-JA and salicylic acid (SA) as reaction to abiotic stress [55]. Interestingly, a highly upregulated gene under drought stress conditions in potatoes, *TAS14* (PGSC0003DMG400003530), coding for a dehydrin [56], is located under the DRYM QTL on LG2 (R) at 40.1 Mb. It is also interesting that the SSR-marker HRO_ALDH_H derived from the gene encoding an aldehyde dehydrogenase (PGSC0003 DMG400034597) mapped in the flanking region of the DRYM QTL on LG4 (R). The SSR-marker derived from the aldehyde dehydrogenase gene (PGSC0003DMG400034597) had shown allelic variation in a potato panel significantly associated with drought sensitivity [34]. Our results indicate and further substantiate also a relation of drought and biotic stress-related factors, e.g., by several overlaps of DRYM QTL with eQTL for resistance proteins such as bacterial spot disease resistance protein 4 (BSDRP4, PGSC0003DMT400080813), nbs-lrr resistance proteins (PGSC0003DMT400035714, PGSC0003DMT400049097) and TMV (tobacco mosaic virus) resistance protein N (PGSC0003DMT400046899). Pathogen resistance inducers have been described to be involved in abiotic stress-induced responses and vice versa [57,58]. The elicited signaling pathways may represent global defense mechanisms shared in abiotic and biotic stress responses that regulate plant metabolism or protect plant organs. Understanding the multifaceted crosstalk between abiotic and biotic stress signaling will be vital to understand stress tolerance/defense [59].

### 3.2. Cell Wall Remodeling Genes under DRYM QTL

Co-localizations of DRYM QTL with eQTL for poly(ADP-ribose) glycohydrolase (*PARG*) were found on four linkage groups (LG1 (A), LG3 (A), LG4 (A) and LG8 (R)). The transcripts of this gene (PGSC0003DMG40029361) had high weight in the Random Forest model for drought tolerance prediction [14]. PARG enzymes increase cellular ADP-ribose by hydrolysis of the respective polymers synthesized by their enzymatic counterpart poly(ADP-ribose) polymerase (PARP) [60]. While PARGs are known to be involved in animal embryonic development, cell death and DNA repair [61,62], less is known about their role in plants [63]. In *Arabidopsis*, silencing PARP increases abiotic stress tolerance, while *parg1-3* mutants show reduced drought tolerance [63,64,65]. More recently, a transcriptomic approach in *Arabidopsis* revealed a link of poly(ADP-ribosyl)ation with cell-wall associated pectin esterases. Here, *parg1* knockout led to downregulation of a pectin methylesterase inhibitor gene [66]. Pectin methylesterases enable alterations in cell wall properties (stiffening or loosening) as the primary cell wall is composed of cellulose, hemicellulose and pectin [67]. Interestingly, the standalone DRYM locus on LG3 (A) comprises genes encoding pectin esterase (PGSC0003DMG400018146), pectin esterase inhibitors (PGSC0003DMG400018189, PGSC0003DMG400034620, PGSC0003DMG400040957) and pectate lyases (PGSC0003DMG400015230 and PGSC0003DMG400018142). The DRYM QTL on LG3 also includes a gene coding for xyloglucan endotransglucosylase/hydrolase protein 9 (PGSC0003DMG402010181), two genes for COBRA proteins (PGSC0003DMG400024530, PGSC0003DMG400024628) and four genes encoding expansins (PGSC0003DMG400024646, PGSC0003DMG400024647, PGSC0003DMG400024648 and PGSC0003DMG400019507). Expansins play a major role in cell wall extension [68]. In combination with xyloglucan endotransglucosylase/hydrolase, expansins loosen the cell wall structure and allow cell growth driven by turgor pressure [69]. COBRA proteins belong to a multigene family also involved in cell expansions and biosynthesis of cell wall components [70]. From the genes involved in cell wall remodeling, pectate lyase (PGSC0003DMG400018142), a pectin modifying enzyme [69], centrally located under the DRYM QTL on LG3 (A), may be one of the most promising candidate genes for drought tolerance in potatoes. With 65 mutations (including one splice and five missense mutations), this gene revealed the highest number of mutations between the bulks, even though most of the other genes involved in cell wall remodeling also showed at least one missense mutation apart from other potentially relevant mutations in non-coding regions. Pectate lyase activity is essential for normal cell growth as well as for the induction of leaf senescence [71].

In addition, the SSR-marker STI0034 for fasciclin-like arabinogalactan protein (*FLA*, PGSC0003DMG400021372) co-localized with two overlapping DRYM QTL on LG1 (A) supporting the relevance of cell wall signal perception and remodeling under drought stress. Arabinogalactan proteins (AGPs) and the FLA subgroup are cell wall associated glycoproteins that are assumed to play a role in cell wall integrity sensing and stress-related cell wall remodeling [67]. The cell wall poses the plant’s very first barrier to sense and protect against environmental impacts [69]. A change in its properties via cell wall remodeling and/or stabilization represents an important component of the plant response to abiotic stress [72]. Under drought stress, an increase in cell membrane stability may also permit higher yield in drought-tolerant potato cultivars [73].

### 3.3. Nonsense Mutations in Genes under DRYM QTL on LG3

Mutations leading to premature stop codons severely affect gene properties and might be relevant for drought tolerance. In total, 63 genes carrying nonsense mutations are located in the genomic region of the DRYM QTL on LG3 (A), about one-third of them representing genes of unknown function. The four most interesting candidate genes that may be responsible for the DRYM QTL are *BAK1* (PGSC0003DMG400018101), flanked by two cytochrome P450 71A4 genes (PGSC0003DMG400018139, PGSC0003DMG400005554), and KDEL-tailed cysteine endopeptidase (PGSC0003DMG400015160). All four genes are located in the central region of the DRYM QTL. Another gene of interest encoding a senescence-associated protein (PGSC0003DMG400024608) resides in the distal flank of the QTL. BAK1 is part of the brassinosteroid (BR) signaling pathway and also plays an essential role in the ABA signaling pathway involved in stomatal closure [74]. ABA supports the complex formation between BAK1 and SnRK2.6 (also known as OST1), which is required for stomata closure. OST1 autophosphorylates and is transphosphorylated by BAK1, whereas PP2C (also known as ABI1) dephosphorylates both OST1 and BAK1. Knock-out of BAK1 by a premature stop codon in the first exon, as detected in our study in the drought-sensitive cultivar and the drought-sensitive bulk, may result in impairment or failure of proper stomatal closure. The second gene copy of BAK1 (PGSC0003DMG400025330) located in the same genomic region may also not be functional due to a detected splice mutation.

Cytochrome P450 is encoded by a very large gene family involved in secondary metabolism, phytohormone biosynthesis, antioxidative substances and detoxification [75]. Given their numerous functions, possible roles in drought tolerance are conceivable, but would require further studies. The other two genes with stop codons in the central region of the DRYM QTL on LG3, namely KDEL-tailed cysteine endopeptidase (KDEL-CysEPs) and senescence-associated protein, are known to play a role in programmed cell death (PCD). KDEL-CysEPs appears to be important for remodeling of meristematic tissues (elongation of cell walls and separation of cells) necessary for the formation of young roots [76], which may be relevant for drought tolerance. Likely, a premature stop codon in the KDEL-CYsEp (PGSC0003DMG400015160) coding sequence may have an impact on root formation. Drought stress is tailgated by accelerated leaf senescence and leaf abscission, resulting in reduced photosynthetic activity and decreased yield [77]. In contrast, a delay in leaf senescence can lead to extreme drought tolerance as could be demonstrated by transgenic tobacco plants expressing an isopentenyltransferase gene under the control of the promoter of a senescence-associated receptor protein kinase [77]. A number of senescence-associated genes (sags) involved in different processes of senescence have been identified in the last years [78,79]. Mutations in senescence-associated genes can improve plant performance under control and under drought conditions [80]. Hence, a nonsense mutation in a gene coding for a senescence-associated protein may be relevant for drought tolerance.

Furthermore, other possible candidate genes with nonsense or missense mutations, but also ones with mutations in the 5′ and 3′UTR affecting gene expression, may be responsible for the DRYM QTL on LG3 (A). The NGS data of the whole genome sequences of Albatros, Ramses and the two bulks represent short paired-end reads. However, assemblies of polyploid genomes using only short reads do not allow capturing of haplotype variation and thereby only represent a single consensus sequence alignment to the chromosome scaffolds [81]. Therefore, it is not possible to assign SNPs to individual alleles representing the genetic constitution of the genes. To obtain this information, additional Sanger sequencing of long-range PCR fragments or long-read sequencing offered by PacBio or Oxford Nanopore will be required [81]. Furthermore, expression analyses and functional studies will be necessary to understand the respective role of the identified candidate genes regarding drought tolerance in potatoes.

### 3.4. Co-Localization of Drought Tolerance and Candidate Genes for Starch Metabolism

For the assessment of drought tolerance, we applied the DRYM index (the deviation of relative starch yield from the experimental median) that can separate drought tolerance from yield potential [32]. Nonetheless, most DRYM QTL displayed strong physical association with QTL for tuber starch yield-related parameters. Only three linkage groups LG3 (A), LG10 (R) and LG12 (R) showed standalone DRYM QTL with no co-localizing tuber starch QTL (Figure S6).

Analyses of the genomic regions underlying DRYM QTL overlapping with QTL for tuber starch content and tuber starch yield indicated a possible linkage between drought tolerance and carbohydrate metabolism. Key enzymes of carbohydrate metabolism and transport are well known [35,36,37]. In our study, genes encoding prominent enzymes involved in synthesis and degradation of starch, such as soluble starch synthase III (*SS3*, PGSC0003DMG400016481), a debranching enzyme (*DBE*) and glucose-1-phosphate adenylyltransferase (*AGP*, PGSC0003DMG400046891), were found under DRYM QTL overlapping with QTL for tuber starch content and tuber starch yield. Moreover, genes encoding a sucrose metabolism enzyme (*PFP-BETA*, PGSC0003DMG400016726) as well as sucrose transporter (*SUT1*, PGSC0003DMG400009213) and adenine nucleotide translocator (*ANT*, PGSC0003DMG400013596) revealed physical co-localizations with combined DRYM and tuber starch QTL. In addition, DRYM QTL on LG2 included starch synthase V (*SS5*, PGSC0003DMG400030619) and a protein targeting to starch (*PTST*, PGSC0003DMG400030609). The latter is involved in starch granule initiation in *Arabidopsis* [82]. The co-localization of DRYM QTL with tuber starch QTL could have two reasons: (1) the DRYM calculation is based on the starch yield, which could lead to an identification of genomic regions involved in starch metabolism, potentially through differential regulation of starch-related genes in different genotypes, or (2) genes for drought tolerance are located in close vicinity to genes of carbohydrate metabolism. A genetic linkage could lead to yield penalties if genes positively affecting yield are closely linked to genes negatively affecting drought tolerance or vice versa. Such a trade-off has been shown in rice for the green revolution gene semi dwarf1 (*sd1*) for reduced plant height that is closely linked to a yield-related gene negatively affecting drought tolerance [83]. In potatoes, drought tolerant cultivars showed significantly lower tuber starch yields compared to drought sensitive cultivars described as yield penalty [32,73]. Survival strategies to cope with water deficit primarily involve minimization of water-loss, for example via stomatal closure to reduce transpiration [3], protection of cellular structures through accumulation of compatible solutes [84] and/or changes in the permeability of cell membranes and the plasticity and/or thickness of the cell wall [71]. All these mechanisms require metabolic energy not available for starch synthesis. In addition, decreased stomatal conductance leads to a significant reduction in photosynthetic carbon fixation [11].

In general, our QTL analyses regarding tuber starch content and tuber starch yield confirmed about two-thirds of the 46 genomic regions identified in the QUEST population and the PIN184 population, especially the strong QTL region for tuber starch content and tuber starch yield in the upper part of LG5 [37]. Both traits, higher starch content and higher starch yield of the tubers, are positively correlated with late maturity [21]. The candidate gene for maturity control *StCDF1* is located under a QTL for plant maturity on LG5, in the same region as QTL for tuber starch content and tuber starch yield [37]. *StCDF1* (PGSC0003DMG400018408, ST4.03ch05: 4,539,029..4,541,329), which encodes for a cycling DOF (DNA binding with One Finger) transcription factor, controls tuberization in dependence of the day length [85]. A SNP-marker derived from *StCDF1* (PGSC0003DMG400018408) was successfully developed [21]. However, in our biparental population A × R both parents belong to the medium early maturity class, so QTL for tuber starch content and tuber starch yield on LG5 should not be due to differences in plant maturity. Another SNP-marker for fructose-biphosphate aldolase (PGSC0003DMG400030565, ST4.03ch05: 3,707,428..3,710,390), which correlates also with tuber starch content and tuber starch yield [37], is present in the same area on LG5 and might explain the major QTL. In contrast to the QUEST population, our study did not detect any QTL for tuber starch content and tuber starch yield on LG3 and LG10. Our results also showed a distribution of QTL for tuber starch content and yield all over LG9. Furthermore, strong QTL were detected on the lower part of LG8 and especially on the upper part of LG11, where highly differential SNPs were only found in the QUEST population with the SolCap SNP array, but not in the PIN184 population [37]. For several candidate genes underlying QTL for tuber starch content and tuber starch yield, SNP-markers were successfully developed and validated [21,37,85]. Interestingly, one of the candidate genes, starch synthase IV, is located in the same region as the DRYM QTL on LG2 in our population, which in addition overlaps with tuber starch yield QTL.

### 3.5. Improvement of Drought Tolerance Requires Identification of Stress-Related Mechanisms that Do Not Affect Yield-Relevant Metabolism

We performed quantitative analyses of drought tolerance based on DRYM index calculations that aimed at a yield-independent characterization of drought tolerance. Different drought response mechanisms within the same population were observed over the investigated three years, when comparing the distribution of DRYM QTL over the genome in three consecutive years. Such differences in drought tolerance within cultivars have also been seen in other studies [86]. Plant responses to drought also depend on stress severity and exposure time [1,32,72]. Few QTL analyses for drought tolerance have been performed in potatoes. Studies in diploid potato populations have identified drought-related regions for physiological and morphological traits with varying results on most [18] to almost all chromosomes, except for 11 and 12 [17]. Interestingly, chromosome 5 was identified as an important drought-specific region [18,55]. Although we have also identified DRYM QTL on most chromosomes, none were mapped on LG5 in our A × R population. However, differences in QTL for drought stress detected in different biparental populations are easy to explain as only differences between the parents can be mapped in the corresponding biparental population, and drought stress response represents a very complex trait involving very intricated networks of gene expression [26]. Our results demonstrate that the response to drought is indeed a multifaceted process involving a number of different metabolic and signaling pathways. Our QTL analyses revealed frequent co-localization of DRYM QTL with mQTL for ribitol, fumaric acid, galactinol and salicylic acid glucopyranoside. Ribitol represents an osmoprotectant under abiotic stress [87], and galactinol acts as donor of activated galactose for the synthesis of raffinose family oligosaccharides [88]. In *Arabidopsis*, overexpression of galactinol synthase (*AtGolS2*) results in drought tolerance accompanied by increased levels of galactinol and raffinose [89,90]. These two sugars have also been implicated in ABA-mediated stress response [91]. Ribitol and galactinol were both consistently increased in potato leaves under drought stress [13]. In wheat, ribitol is also significantly increased under drought, while fumaric acid is only increased in leaves but decreased in roots [92]. Ribitol and fumaric acid were also used in a Random Forest model for the prediction of drought tolerance based on field training data comprising 24 metabolites [14]. The synthesis of compatible solutes uses energy and resources making them unavailable for starch biosynthesis. Synthesis of compatible solutes such as ribitol could be a possible explanation for the yield penalty observed in drought-tolerant potato cultivars [32]. In addition, we found multiple overlaps between DRYM QTL and QTL for tuber starch content, tuber starch yield and tuber fresh weight. Well-known genes of the carbohydrate metabolism were localized under DRYM QTL indicating a linkage between drought tolerance and tuber starch content as well as tuber starch yield. Using SSR-markers derived from candidate genes for drought tolerance [34] and the information of the annotated potato genome sequence, we identified candidate genes for drought tolerance underlying DRYM QTL that are interesting for future studies of drought response mechanisms in potatoes and that partly overlap with previously identified transcript candidate genes for drought tolerance prediction [14]. Combined use of SNP-based markers for the identified candidate genes for drought tolerance and for carbohydrate metabolism will be necessary in breeding for drought tolerance in potatoes in order to avoid reductions in tuber starch yield.

## 4. Materials and Methods

### 4.1. Plant Material and Experimental Design

A cross between two tetraploid potato cultivars (2n = 4x = 48), drought-tolerant Albatros and drought-sensitive Ramses [34], was used for the QTL studies. Albatros and Ramses represent potato varieties with medium early maturity (120–140 days). Both potato varieties are cultivated for industrial use, mainly in the starch industry. Albatros has a starch content of about 22%, Ramses of about 21%. Seeds of the F_1_ population Albatros x Ramses (A × R) were provided by German potato breeding companies (Böhm-Nordkartoffel-Agrarproduktion GmbH & Co OHG, Strehlow; Norika Nordring-Kartoffelzucht- u. Vermehrungs-GmbH, Groß-Lüsewitz; SaKa Pflanzenzucht GmbH & Co. KG, Hamburg, Germany). In 2013, leaf material for marker analyses was collected from 265 individual F_1_ potato plants grown in the polytunnel at the MPI-MP, Golm, Germany. DNA was extracted as previously described by Doyle and Doyle [93]. For the following drought stress experiments, F1 plants needed to be propagated to have enough tubers for the field trials and sufficient F1 plantlets by in vitro propagation for the other experiments. However, not all F1 plants stayed healthy and produced enough tubers and F1 plantlets by in vitro propagation. This reduced the population to 88 F1 plants in 2014. The experiment IDs are the primary identifiers of the experiments used in the MPI-MP plant experiment database [94], which are consecutively issued to all experiments done by the MPI-MP or performed in cooperation with other institutes. The locations for the experiments under control (Co) and stress (Ds) conditions were MPI-MP Golm polytunnel (67199, 72247, 76240), MPI-MP Golm field (72275, 76219), JKI Groß Lüsewitz field (67518, 72396, 76529), JKI Groß Lüsewitz shelter (68015, 72292, 76354) in 2014, 2015 and 2016, respectively. In 2015 and 2016 Dethlingen field (72482, 76528) was added as a third location. In 2014, two replicates of each F1 plant for control and drought stress were cultivated in a randomized split-block design at the JKI Groß Lüsewitz (67518, 68015). At the MPI-MP Golm, three replicates of one plant per clone were grown in the polytunnel (67199) in a randomized split-block design. In 2015 and 2016, only a subpopulation of F_1_ progenies representing low and high DRYM values were analyzed by QTL mapping, but with higher numbers of replications for better statistical resolution. At the JKI, two replicates for each treatment (control and drought) with three repetitions per clone were analyzed in the shelter (72292, 76354) and, in addition, two replicates with five repetitions per clone in the field trials (72396, 76529). At the MPI-MP Golm in 2015, two replicates of three plants per clone were grown under control and drought conditions in the polytunnel (72247) and three replications of five plants per clone under control and drought conditions in the field (72275). In 2016, five replicates of one plant per clone were cultivated in the polytunnel (76240) and three replications of eight plants per clone in the field (76219). All trials in 2016 had a randomized split-block design investigated under control and drought conditions. The field trials in Dethlingen (72482, 76528) in 2015 and 2016 were performed in two replicates with 10 plants per replicate.

### 4.2. Control and Drought Stress Treatment

All drought stress treatments, with exemption of Dethlingen, represented early stress scenarios starting before flowering, but experiments were performed in each location according to the facilities available and the long-term experience with drought experiments. Experiments in Dethlingen corresponded to late stress after flowering. Six different drought stress treatments were executed, three consistent over a time of 3 years (one in the MPI-MP Golm polytunnel and two at the JKI). Another drought stress scenario was applied at Dethlingen for two consecutive years on the field. For the field trials at the MPI-MP Golm, drought stress treatments differed slightly in 2015 and 2016 (see below). F_1_ progenies were grown either in big-bags, in pots or in the field with different drought stress treatments [38].

For the big-bag trials at the MPI Golm polytunnel (67199, 72247, 76240), the drought stress was started at the five-leaf stage and continued until maturity of the plants (>BBCH 90). All potato plants were drip-irrigated, but the drought-stressed plants received only 50% of the water obtained by the plants grown under optimal conditions. The volume reduction was achieved by increasing the time interval between irrigations. In the MPI Golm field trials (72275, 76219) plants in the control and drought stress block were drip-irrigated with a volume of 10 L/m^2^ when turgor loss was observed at noon (control) or at 7:00 a.m. (stress treatment). Stress-treated plants were grown under a rain-out shelter while control plants were grown in the open field. The total amount of water received by the stressed plant amounted to 35% and 32% of the volume received by the control plants (irrigation plus rain) in the two experiments in 2015 and 2016, respectively (more details see in Haas et al. [38]).

In the pot trials at JKI Groß-Lüsewitz under a rain-out shelter (68015, 72292, 76354) drought stress was initiated at the three-leaf stage. Plants in the drought stress block went through a continuous alternation between no irrigation and watering until harvest. Plants in the drought stress block were irrigated (each time about 10 days after beginning of the stress phase) with an amount of water corresponding to three times the daily evapotranspiration when 50% of the plants were beginning to show turgor loss. For the control block, the potato plants were weighed daily to estimate the loss of water, and the evaporated water was replaced so that the water capacity could be kept at 50%. For the field trials in Groß-Lüsewitz (67518, 72396, 76529), potatoes were watered once at the beginning to allow emergence of the plants. The drought-stressed plants located under a shelter did not obtain any further water until the end of the experiment, whereas the control plants received artificial irrigation in addition to the regular rain fall to maintain optimal water conditions. Experiments in Groß-Lüsewitz for pot trials (rain-out shelter) and field were terminated when 50% of the plants reached complete senescence (BBCH 97). Potato tubers were harvested two weeks later.

In the field trials in Dethlingen (72482, 76528) the potato plants were irrigated to maintain 50% field capacity until buds of first inflorescence extended to 5 mm (BBCH 55) in 2015 (72482), then irrigation was stopped for two weeks for the drought stress block, while irrigation continued for the control. In 2016, the potato plants were irrigated to maintain 50% field capacity until 30% of berries in the first fructification had reached full size (BBCH 73), and then irrigation was stopped for one week for the drought stress block, while the control plants received irrigation as before (76528). In Dethlingen, potato plants were harvested in both years at BBCH 93. For further details on climate conditions and irrigation volumes see Haas et al. [38].

### 4.3. SSR and AFLP Analyses

In total, 59 linkage group specific SSR-primer combinations [33,95,96,97,98] (Table S1) as well as 34 candidate gene specific SSR-primers (Table S2) were used to amplify genomic DNA. The development of SSR-primer combinations from the candidate genes specific for drought tolerance has been previously published [34]. GenBank accession numbers to retrieve the sequences surrounding the linkage group specific SSRs were obtained from the supplementary file of Ghislain et al. [33] and used for BLAST against *Solanum tuberosum* in EnsemblPlants to obtain the location in the potato reference genome assembly SolTub_3.0. In case of missing GenBank accession numbers, primer sequences of SSRs were directly used for the BLAST. Forward primers were synthesized with an additional M13-tail. A M13 primer carrying a fluorescent dye (IRD700 or IRD800) for infrared detection using a LI-COR 4300 DNA analysis system was added to the PCR for labelling. PCR reactions were performed with minor changes as described by Sajer et al. [99].

AFLP analyses were performed [100] using genomic DNA digested with *Eco*RI and *Mse*I. Fragments were ligated to E and M adapters. Preamplification was performed with E01 and M02 as primers. In total, 47 primer pairs were used for selective amplifications (Table S9). All AFLP primer sequences and numbers were used according to Keygene, N.V., Wageningen, NL (http://wheat.pw.usda.gov/ggpages/keygeneAFLPs.html, accessed on 3 June 2021).

### 4.4. Linkage Mapping Using Tetraploid Map

Of all scored markers, only those with informative segregation ratios (simplex [A000 × 0000], 1:1; duplex [AA00 × 0000], 5:1; or double-simplex [A000 × A000], 3:1; Ratio_Sig > 0.1 for AFLP-markers, Ratio_Sig > 0.01 for SSR-markers) were selected. Markers segregating 11:1 (simplex x duplex [A000 × AA00]) and 35:1 (duplex x duplex [AA00 × AA00]) were omitted from the linkage analysis as they are not useful for the estimation of recombination rates. Markers were mapped to the corresponding parental linkage group using a modified version of the TetraploidMap software for linkage and QTL mapping in autotetraploid species [101,102], which was adapted to handle larger numbers of markers (up to 800 markers) than the original version. TetraploidMap cluster analyses were separately performed for each parent, allowing the grouping of the markers into 12 groups (overall linkage groups). The markers of the groups were then phased using the group code provided by the program so that maps for all four homoeologous chromosomes could be created.

### 4.5. QTL Analysis of Drought Tolerance and Yield-Associated Traits

To determine drought tolerance-relevant QTL based on yield (comprising tuber starch yield, tuber starch content, tuber weight) and drought tolerance index (DRYM), data from all locations and years under control and drought conditions were used. Tubers had been harvested about 100 days after the start of the experiments. Raw data are available at Edal [83]. Tuber weight was measured around four weeks after harvesting of the potatoes. Tuber starch content in g per kg was estimated using a starch balance (Type E6100, MEKU). Tuber starch yield represents the product of tuber weight and tuber starch content for each replicate. DRYM was used as drought tolerance index [32]. DRYM was calculated on the basis of the relative tuber starch content (RelSy_GxEi_) of a genotype, which represents the quotient of the tuber starch yield of a genotype in an experiment under drought stress and the tuber starch yield of a genotype in an experiment under control conditions. DRYM values were obtained by subtracting the median of the relative tuber starch content of the whole experiment (median (RelSYE_i_) from the relative tuber starch yield of the genotype (relSyG_xi_E_i_) according to the equation:

$$DRYM_{G_{x},E_{i}}=RelSY_{G_{x},E_{i}}-median\left(RelSY_{E_{i}}\right)$$

The metabolome and transcript data were based on two trials: MPI Golm polytunnel (61711) and JKI shelter (68015) in 2014 measuring 95 A × R F_1_ clones, as described in Sprenger et al. [13,14]. Leaf material for metabolome and transcriptome analyses was sampled at the stage of full flowering (BBCH 60–65) from control (Co) and drought stress plants (Ds) (Tables S5 and S7). Leaf samples were taken about 65 days after planting of the tubers. Two primary leaflets were harvested from the first fully developed leaf and instantly frozen in liquid nitrogen. Samples were stored at −80 °C until analysis. Metabolome analyses by gas chromatography–mass spectrometry and transcript analyses by qRT-PCR are described in detail in Sprenger et al. [14].

QTL analyses were performed separately for each location, each treatment and each year and with normalized combined data over all three years. Data for starch yield were normalized with regard to the factors block (B), row (R) and ridge (D). Starch yield values were modeled as a result of a linear-effects model associated with the variables B (if two or more blocks were set up in the experiment), R and D by applying the “lm” function for coding a linear model and by treating the variables B, R and D as categorical factors (ANOVA, “avo” function in R). The obtained model (M) was used to compute normalized starch yield values (SY_norm_) using the “predict” function, where SYM (B, R, D) are the regressed values of SY based on the obtained linear model, M, and adding the median of the raw values to preserve the absolute magnitude of values before and after normalization. The statistical evaluation was performed using R (3.2.3, RStudio v. 1.0.143, RStudio Inc., Boston, MA, USA) and SAS v. 9.4 (SAS Institute Inc., Cary, NC, USA).

On the basis of the linkage maps and the trait datasets for DRYM, tuber starch content, tuber starch yield, tuber fresh weight, transcripts and metabolites, QTL analyses were performed for each linkage group separately using the default parameters in TetraploidMap for Windows [91]. Interval mapping displayed the LOD score profile charts for each trait, including the LOD score statistics and the percentage of explained variance. To test statistical significance of each QTL position, a permutation test (*n* = 500) was performed using TetraploidMap. Only QTL with a LOD > 3 were considered. QTL were mapped showing the threshold of 90% and 95%. QTL analyses were done separately for both parents of the F_1_ population A × R. A graphical visualization of the linkage maps and QTL was performed using MapChart 2.30 [103].

### 4.6. Search for Candidate Genes in Databases

Search for candidate genes for *Solanum tuberosum* using gene IDs was performed in the database of EnsemblPlants (http://plants.ensembl.org/index.html) SolTub v3.0 and in Phytozome v12.1 for *Solanum tuberosum* genome assembly SolTub v4.03 (https://phytozome.jgi.doe.gov/pz/portal.html). Additional information was retrieved from SPUD DB (http://solanaceae.plantbiology.msu.edu/) for genome assembly SolTub v6.1 and NCBI (https://www.ncbi.nlm.nih.gov/). All web sites in this paragraph were accessed on 3 June 2021.

### 4.7. Whole-Genome Sequencing in Tetraploid Potato

Next-generation sequencing was performed for two cultivars, Albatros and Ramses, which represent the parents of the cross, and bulks of drought-tolerant (20 plants with the highest DRYM values) and drought-sensitive (20 plants with the lowest DRYM values) F1 plants of the cross A × R based on the experimental trials of 2014. Ranking was performed in SAS version 9.4 (SAS Institute, Inc., Cary, NC, USA). Whole-genome sequencing on an Illumina HiSeq platform by GENEWIZ was accomplished using a sequencing configuration of 2 × 150 bp paired-end reads within the Illumina TrueSeq Paired-End Sequencing workflow. A genome coverage of 120x was targeted. Alignment of the reads and variant calling was performed by GENEWIZ using the Dynamic Read Analysis for GENomics (DRAGEN) platform in combination with GATK (Genome Analysis Toolkit) [104,105]. The diploid potato genome assembly DM v4.03 derived from the doubled monoploid potato (*S. tuberosum* Group Phureja) clone DM1-3 516 R44 was used as the reference genome [7]. The annotated VCF files were provided by GENEWIZ and contained all information about single-nucleotide variants (SNVs) and Insertion/Deletions (INDELs) as well as the effects of the variants on the genes for each of the four samples. The detected number of SNPs was then reduced from approximately 20 million SNPs per sample to about 6 million SNPs per sample by applying the following filter settings: GQ score of 99 or higher, a minimum allele coverage ≥15 reads, total coverage between 80 to 360 reads and an allele frequency of at least 10%. The number of SNPs was further reduced by considering only those SNPs of interest, which were present in only one bulk, but absent (0%) in the other, either the drought-tolerant or the drought-sensitive bulk. SNP analyses were restricted to SNPs targeting genes covered by the DRYM QTL on LG3 (ST4.03ch3: 47,424,062 bp–57,312,556 bp). Using Perl scripts, these parameters were applied using the individual SNP calling files (VCF format) of Albatros, Ramses and the drought-tolerant and -sensitive bulks in comparison to the GFF3 files from the potato DM v4.03 genome annotation. Results from the remaining 25,273 SNPs located in the region of the standalone DRYM QTL on LG3 are reported in this study. 

## 5. Conclusions

This study represents the first report of using the newly derived SSR-primer combinations from candidate genes for drought tolerance [34] in constructing genetic maps in tetraploid potatoes. Interestingly, in some cases these SSR-markers coincide with DRYM QTL in the F_1_ population A × R and might represent allelic variations that result in the DRYM QTL. In addition, a comprehensive analysis of drought stress experiments including DRYM, tuber starch content, tuber starch yield, tuber fresh weight, transcriptome and metabolome data is presented, confirming also some of the published QTL. New is the combination of whole-genome sequences of the two corresponding parental varieties, Albatros and Ramses, with the application of BSAseq. Applying BSAseq gave an insight into mutations within candidate genes under the DRYM QTL on LG3 that might be relevant for drought tolerance. The whole-genome sequences of the parental varieties allow the assignment of the origin of the mutation to one of the parents. So far, only whole-genome sequences of six polyploid landraces (*Solanum* spp.) have been published [106], but no tetraploid potato genomes of cultivated varieties. Mutations in the potential candidate genes for drought tolerance that encode BAK1, ethylene signaling pathway components, ERFs and enzymes associated with cell wall remodeling such as pectate lyase, pectin esterases, pectin esterase inhibitors and expansins may provide interesting starting points to develop diagnostic SNP-based markers and to breed for drought tolerance in potato by marker-assisted selection.

## Figures and Tables

**Table 1:** Overlap of DRYM QTL (green) with tuber starch content, tuber starch yield and tuber fresh weight QTL. QTL underlying DRYM QTL are shown in white, and flanking QTL are in grey.

LG	QTL	LOD Threshold (95%)	Max LOD Score	Position (cM)	1 LOD Region (cM)	2 LOD Region (cM)	Explained Variance (%)
1A	72292_2015_Ds_drym	3.86	6.22	8	0–12	0–14	63.9
1A	68015_2014_Ds_drym	3.65	3.97	8	0–14	0–24	19.1
1A	76354_2016_Ds_tuber_FW_kg_per_plant	4.45	4.74	46	28–62	6–64	59.7
1A	68015_2014_Co_starch_g_per_kg	3.37	4.13	64	62–66	62–68	20.1
1A	67518_2014_Co_starch_g_per_kg	3.37	3.76	128	122–132	74–132	17.2
1A	76354_2016_Ds_starch_g_per_kg	4.49	5.48	94	86–132	84–132	79.4
1A	67199_2014_Ds_tuber_FW_kg_per_plant	3.54	5.69	124	118–132	114–132	25.8
1R	76219_2016_Ds_drym	3.84	4.29	84	4–40	2–46	43.0
1R	67518_2014_Co_starch_yield_g_per_plant	3.33	3.78	84	4–16	2–22	14.1
1R	72292_2015_Ds_drym	3.88	4.60	66	26–38	24–40	53.5
1R	67518_2014_Co_tuber_FW_kg_per_plant	3.27	3.42	84	2–16	2–30	12.4
2R	76240_2016_Ds_drym	3.87	4.01	56	42–74	34–80	52.6
2R	76528_2016_Ds_starch_yield_g_per_plant	4.06	4.36	88	48–96	28–100	50.3
2R	MW_Ds_tuber_FW_kg_per_plant	4.34	4.17	48	36–100	32–106	58.9
2R	norm_Ds_starch_yield_g_per_plant	4.99	4.75	58	40–74	36–82	58.5
2R	67518_2014_Co_starch_yield_g_per_plant	3.54	4.43	74	46–80	38–90	20.8
2R	67518_2014_Co_tuber_FW_kg_per_plant	3.31	4.23	58	46–82	38–100	20.4
2R	76528_2016_Ds_drym	7.27	8.01	88	52–94	42–102	74.4
2R	76528_2016_Co_starch_yield_g_per_plant	4.35	6.54	92	86–96	84–100	69.3
2R	72275_2015_Co_tuber_FW_kg_per_plant	4.76	4.77	88	84–94	84–100	52.7
2R	72482_2015_Co_tuber_FW_kg_per_plant	4.11	4.92	88	86–94	84–104	53.7
2R	76528_2016_Co_tuber_FW_kg_per_plant	4.55	6.08	92	86–96	86–102	66.2
2R	MW_Co_tuber_FW_kg_per_plant	4.39	4.38	88	84–98	84–100	48.6
3A	76354_2016_Ds_drym	4.41	4.56	64	48–74	42–78	53.0
4A	68015_2014_Ds_drym	3.47	4.63	0	0–10	0–24	18.0
4A	67199_2014_Ds_starch_g_per_kg	3.50	3.83	8	0–12	0–28	13.8
4A	67199_2014_Co_starch_g_per_kg	3.26	5.71	8	0–20	0–32	23.9
4A	68015_2014_Co_starch_g_per_kg	3.42	3.97	0	0–18	0–40	15.2
4A	68015_2014_Co_starch_yield_g_per_plant	3.37	5.09	8	0–24	0–36	19.6
4A	MW_Co_starch_g_per_kg	5.26	5.29	88	64–94	58–96	60.1
4A	72482_2015_Co_starch_g_per_kg	4.03	4.77	88	70–122	60–122	53.4
4R	76529_2016_Ds_drym	4.52	5.25	54	44–62	42–64	72.7
4R	68015_2014_Co_starch_g_per_kg	3.29	4.71	32	28–56	22–66	17.9
4R	68015_2014_Co_tuber_FW_kg_per_plant	3.47	5.35	28	20–52	16–58	22.3
4R	67199_2014_Ds_tuber_FW_kg_per_plant	3.57	3.94	0	0–2	0–4	14.0
4R	76528_2016_Ds_tuber_FW_kg_per_plant	4.23	5.59	4	0–6	0–8	54.5
6A	72292_2015_Ds_drym	3.99	4.07	36	20–44	0–52	40.0
6A	68015_2014_Co_starch_g_per_kg	3.29	3.47	14	0–22	0–22	14.8
6A	76354_2016_Ds_drym	4.23	5.26	66	60–80	58–88	56.8
6A	76240_2016_Ds_starch_yield_g_per_plant	3.86	4.22	92	70–92	64–92	41.6
6A	76354_2016_Ds_tuber_FW_kg_per_plant	3.90	4.66	46	46–86	40–92	51.9
6A	76240_2016_Ds_tuber_FW_kg_per_plant	3.86	3.90	90	68–92	62–92	39.0
7R	67518_2014_Ds_drym	3.32	3.62	48	3–49	3–51	14.0
7R	67518_2014_Ds_starch_g_per_kg	3.51	3.94	46	3–17	3–31	14.2
7R	72247_2015_Ds_starch_g_per_kg	4.07	5.33	48	3–31	3–39	49.1
7R	72275_2015_Ds_starch_g_per_kg	4.26	7.11	48	3–7	3–41	61.9
7R	MW_Ds_starch_g_per_kg	4.45	4.55	30	17–37	3–45	45.2
7R	72275_2015_Co_starch_g_per_kg	4.41	6.72	48	3–19	3–33	59.6
7R	76219_2016_Co_starch_g_per_kg	4.05	4.22	42	3–35	3–45	42.0
7R	72482_2015_Co_starch_g_per_kg	4.08	4.27	48	3–43	3–47	40.8
7R	68015_2014_Co_starch_g_per_kg	3.44	4.28	8	25–51	3–51	16.4
7R	76528_2016_Co_starch_g_per_kg	3.75	3.90	24	15–47	3–51	42.5
7R	MW_Co_starch_g_per_kg	5.16	4.60	48	3–37	3–49	42.8
7R	72292_2015_Co_starch_yield_g_per_plant	3.82	4.21	30	15–45	9–51	47.0
7R	72482_2015_Ds_starch_yield_g_per_plant	4.12	4.35	0	47–51	43–51	46.9
7R	72482_2015_Ds_tuber_FW_kg_per_plant	4.03	4.48	0	47–51	43–51	47.3
8R	76528_2016_Ds_drym	7.04	7.14	0	0–20	0–20	72.0
8R	76219_2016_Ds_drym	3.86	3.98	14	0–28	0–34	53.5
8R	norm_Ds_starch_yield_g_per_plant	5.03	5.69	0	0–16	0–16	62.8
10R	72247_2015_Ds_drym	4.10	4.13	44	40–48	38–48	46.9
10R	76219_2016_Ds_tuber_FW_kg_per_plant	3.61	4.22	4	2–10	2–12	39.3
10R	76240_2016_Ds_tuber_FW_kg_per_plant	3.70	4.52	4	2–8	0–10	42.1
11R	67518_2014_Ds_drym	3.42	4.86	40	26–60	22–90	23.2
11R	72292_2015_Ds_starch_g_per_kg	4.22	5.60	90	82–90	82–90	57.5
11R	76219_2016_Ds_starch_g_per_kg	3.76	3.85	86	82–90	82–90	41.0
11R	76240_2016_Ds_starch_g_per_kg	4.08	5.53	84	82–90	82–90	55.6
11R	MW_Ds_starch_g_per_kg	4.61	6.42	86	82–90	82–90	61.1
11R	MW_Co_starch_g_per_kg	4.85	4.62	86	82–90	82–90	46.5
11R	72247_2015_Co_tuber_FW_kg_per_plant	3.85	4.08	90	86–90	84–90	42.2
11R	72292_2015_Ds_tuber_FW_kg_per_plant	4.00	4.57	90	84–90	82–90	49.2
11R	76219_2016_Ds_tuber_FW_kg_per_plant	4.03	4.83	86	82–90	82–90	50.8
12A	76354_2016_Ds_drym	4.41	5.46	66	3–9	3–11	58.3
12A	72275_2015_Ds_drym	3.73	3.95	64	3–23	3–33	39.9
12A	76219_2016_Co_starch_g_per_kg	4.31	4.36	64	3–9	3–17	50.4
12A	72396_2015_Co_starch_g_per_kg	3.62	4.37	64	3–11	3–19	47.4
12A	76528_2016_Co_starch_g_per_kg	3.84	4.18	64	3–11	3–23	43.8
12A	76528_2016_Ds_starch_g_per_kg	3.94	4.04	64	3–23	3–25	42.0
12A	76219_2016_Ds_starch_g_per_kg	3.51	3.98	66	3–23	3–25	42.7
12A	68015_2014_Co_tuber_FW_kg_per_plant	3.22	4.03	64	3–11	3–49	15.9
12R	72292_2015_Ds_drym	3.92	4.51	20	12–24	8–38	71.1
12R	76219_2016_Ds_starch_g_per_kg	3.84	4.41	94	72–94	68–94	56.8

**Table 2:** Genomic regions of DRYM QTL and candidate genes for carbohydrate metabolism located in these regions. The candidate genes encoding enzymes involved in starch metabolism and underlying DRYM QTL (green) are shown in blue, flanking markers are in grey, and markers underlying DRYM QTL are in white. Genomic positions of the SSR-markers are given in Table 3.

LG	QTL/ Marker Correlation	Position (cM)	Starch Candidate Gene	Location Phytozome Mb	Phytozome ID/ GenBank PGSC0003	Annotation
**2R**	**76240_2016_Ds_drym**	**34–80**				
	**76528_2016_Ds_drym**	**42–102**				
	E39M60_212	30.2	*SS4*	30.14	DMG400008322	Starch synthase IV
	HRO_EREBP1_1_a2	56.7	*PFP-BETA*	36.84	DMG400016726	Pyrophosphate-fructose 6-phosphate 1-phosphotransferase subunit beta
	STI0036	76.6	*SS3*	36.38	DMG400016481	Soluble starch synthase III; chloroplastic/amyloplastic
	STM5114y_b	93.6	*PTST1*	41.93	DMG400030609	Protein targeting to starch
	HRO_ACCS3_A_D	100.6	*SS5*	42.10	DMG400030619	Starch synthase V
	STI0024_d	105.7				
**7R**	**67518_2014_Ds_drym**	**3–51**				
	STM0031_a_c	6.3	*SPS*	3.89	DMG400027936	Sucrose-phosphate-synthase
	STI0033_2	6.5	*SUS II*	40.64	DMG400013546	Sucrose synthase 2
	STI0025_2	7.9				
**8R**	**76528_2016_Ds_drym**	**0–20**				
	**76219_2016_Ds_drym**	**0–34**				
	HRO_MRP_ATF_3D_b	0				
	STM1104	16.3	*WAXY*	56.8	DMG400012111	Granule-bound starch synthase
	E41M61_162	41.6				
**11R**	**67518_2014_Ds_drym**	**22–90**	*DBE*	3.95	A52190.1	De-branching enzyme
	HRO_BSDRP4_5C_b	18.0	*SEX4-like*	4.3	DMG400027327	Protein tyrosine phosphatase
	STM5130_a_d	39.6	*SUT1*	9.05	DMG400009213	Sucrose transport protein
	STI0028_1	65.2	*TAL1*	19.47	DMG402028027	Transaldolase
	E41M61_85	92.5	*ANT*	34.62	DMG400013596	ADP; ATP carrier protein
**12A**	**76354_2016_Ds_drym**	**3–11**				
	**72275_2015_Ds_drym**	**3–33**				
	HRO_ETR1_1A_a_d	0.0	*AGP*	1.22	DMG400046891	Glucose-1-phosphate adenylyltransferase
	HRO_EIX_1E_a	8.4				
	HRO_JA2_1_B	11.6				
	HRO_EBF1_2_b_2	24.7				
	E38M48_140	33.7				

**Table 3:** Genomic regions of DRYM QTL (green) and candidate genes for drought tolerance (light green) derived from SSR markers mapping in these genomic regions. Flanking markers are shown in grey, and underlying markers are in white.

LG	QTL/Marker Correlation	Position (cM)	Explained Variance in %	Drought Candidate Gene	Annotation	Phytozome PGSC0003	Location Phytozome (Mb)
**1A**	**72292_2015_Ds_drym**	**0–14**	63.9				
	**68015_2014_Ds_drym**	**0–24**	19.1				
	HRO_LIPOX_1B	0		*LOX*	Lipoxygenase	DMG400032207	2.15
	STI0034_b	15.2		*FLA14*	Fasciclin-like arabinogalactan protein 14	DMG400021372	2.66
	STI0043_c	18.2			Zinc finger protein	DMG400016379	3.5
	STI0043_b	23			Zinc finger protein	DMG400016379	3.5
**1R**	**76219_2016_Ds_drym**	**2–46**	43.0				
	**72292_2015_Ds_drym**	**24–40**	53.5				
	HRO_EIL2_1_b	0		*EIL2*	Ethylene insensitive 3-like2	DMG400008712	6.14
	STG0016_1_c	44.6		*LHP1*	Chromo domain protein LHP1	DMG400031112	67.23
	STG0016_2	51.1		*LHP1*	Chromo domain protein LHP1	DMG400031112	67.23
**2R**	**76240_2016_Ds_drym**	**34–80**	52.6				
	**76528_2016_Ds_drym**	**42–102**	74.4				
	E39M60_212	30.2			-		
	HRO_EREBP1_1_a2	56.7		*EREBP1*	putative ethylene responsive element binding protein 1	DMG400029713	33.63
	STI0036	76.6			Transcriptional regulator family protein	DMG400028477	31.85
	STM5114y_b	93.6			Disease resistance response protein	DMG403001521	38.55
	HRO_ACCS3_A_D	100.6		*ACS3*	1-aminocyclopropane-1-carboxylate synthase 3	DMG400021426	42.42
	STI0024_d	105.7		*HRGP*	Hydroxyproline-rich glycoprotein family protein	DMG400010074	44.53
**3A**	**76354_2016_Ds_drym**	**42–78**	53				
	**STG0018_b**	**41.2**			Glutamine-rich protein	DMG400026490	47.43
	**STM5115_D**	**76.8**		*GK*	Glycerol kinase	DMG400014144	57.31
**4R**	**76529_2016_Ds_drym**	**42–64**	72.7				
	**HRO_ALDH_H**	39.6		*ALDH*	Aldehyde dehydrogenase family 7 member	DMG400034597	22.59
	**STI0001_1_c**	73		*TSSR*	Tuber-specific and sucrose- responsive element binding protein	DMG400007994	68.72
**6A**	**72292_2015_Ds_drym**	**0–52**	40.0				
	HRO_LEA_1_A_2	0		*LEA*	Late embryogenesis abundant protein 5	DMG400017936	0.46
	STI0021_2_c	21.4		*HSFA6b*	Heat stress transcription factor A-6b	DMG400016270	40.22
	STI0021_1	28.7		*HSFA6b*	Heat stress transcription factor A-6b	DMG400016270	40.22
	STI0021_2_d	36.4		*HSFA6b*	Heat stress transcription factor A-6b	DMG400016270	40.22
	STM5126_1	58.1			Conserved gene of unknown function	DMG400004051	50.92
**6A**	**76354_2016_Ds_drym**	**58–88**	56.8				
	STM5126_1	58.1			Conserved gene of unknown function	DMG400004051	50.92
	STM5126_3	64.2			Conserved gene of unknown function	DMG400004051	50.92
	HRO_BADH_2_c	65.6		*BADH*	Betaine aldehyde dehydrogenase	DMG400033028	52.13
	HRO_BADH_2_a	72.1		*BADH*	Betaine aldehyde dehydrogenase	DMG400033028	52.13
	STI004_2_a	82.0			Nucleic acid binding protein	DMG400003372	55.86
	STI004_1	94.2			Nucleic acid binding protein	DMG400003372	55.86
**7R**	**67518_2014_Ds_drym**	**3–51**	14.4				
	STM0031_a_c	6.3			-		
	STI0033_2	6.5		*HSFA9*	Heat stress transcription factor HSFA9	DMG400032793	36.27
	STI0025_2	7.9			-		
**8R**	**76528_2016_Ds_drym**	**0–20**	72.0				
	**76219_2016_Ds_drym**	**0–34**	53.5				
	HRO_MRP_ATF_3D_b	0		*MRP*	Multidrug resistance protein ABC transporter	DMG400012167	55.53
	STM1104	16.3			-		
	E41M61_162	41.6			-		
**10R**	**72247_2015_Ds_drym**	**38–48**	46.9				
	STG0025	38.6			Oxidoreductase/transition metal ion binding protein	DMG400028767	33.54
**11R**	**67518_2014_Ds_drym**	**22–90**	23.2				
	HRO_BSDRP4_5C_b	18.0		*Bs4*	Bacterial spot disease resistance protein 4	DMG400033334	37.64
	STM5130_a_d	39.6		*SNRNP*	U11/U12 small nuclear ribonucleoprotein	DMG400031069	3.78
	STI0028_1	65.2			Conserved gene unknown function	DMG400007365	37.97
	E41M61_85	92.5			-		
**12A**	**76354_2016_Ds_drym**	**3–11**	58.3				
	**72275_2015_Ds_drym**	**3–33**	39.9				
	HRO_ETR1_1A_a_d	0.0		*ETR1*	Ethylene receptor 1	DMG400007843	1.11
	HRO_EIX_1E_a	8.4		*EIX*	Ethylene-inducing xylanase	DMG400007876	1.81
	HRO_JA2_1_B	11.6		*JA2*	Jasmonic acid 2	DMG400015342	0.82
	HRO_EBF1_2_b_2	24.7		*EBF1*	EIN3-binding F-box protein 1	DMG400002914	2.85
	E38M48_140	33.7			-		
**12R**	**72292_2015_Ds_drym**	**8–38**	71.1				
	HRO_EBF1_2_a	1.4		*EBF1*	EIN3-binding F-box protein 1	DMG400002914	2.85
	STM5121_a	25			Conserved gene unknown function	DMG400000292	4.0
	STI0030_1_a	37.9			Conserved gene unknown function	DMG400014472	49.06

**Table 4:** Genes encoding enzymes involved in phytohormone metabolism underlying the DRYM QTL on LG3.

Chromosome	Region	ID	Name
ST4.03ch03	49,754,402..49,754,857	PGSC0003DMG400010135	Ethylene-responsive element-binding family protein
ST4.03ch03	50,652,743..50,654,344	PGSC0003DMG400015255	DELLA protein RGL1
ST4.03ch03	50,902,946..50,905,075	PGSC0003DMG400015188	Auxin-independent growth promoter
ST4.03ch03	51,989,958..51,991,673	PGSC0003DMG400018128	Protein phosphatase 2C
ST4.03ch03	52,593,703..52,596,962	PGSC0003DMG400018101	BAK1
ST4.03ch03	52,736,187..52,740,429	PGSC0003DMG400018153	Gibberellin receptor GID1
ST4.03ch03	53,228,956..53,233,993	PGSC0003DMG400025330	BAK1
ST4.03ch03	54,533,650..54,534,364	PGSC0003DMG400024606	ERF transcription factor
ST4.03ch03	57,039,987..57,041,187	PGSC0003DMG400014196	Ethylene response factor

**Table 5:** Genes involved in cell wall stability and flexibility.

Chromosome	Region	ID	Name
ST4.03ch03	49,222,889..49,226,202	PGSC0003DMG402010181	Xyloglucan endotransglucosylase/hydrolase protein 9
ST4.03ch03	51,432,584..51,434,561	PGSC0003DMG400015230	Pectate lyase
ST4.03ch03	52,023,650..52,024,174	PGSC0003DMG400040957	Pectinesterase inhibitor
ST4.03ch03	52,075,082..52,075,546	PGSC0003DMG400034620	Pectinesterase inhibitor
ST4.03ch03	52,079,987..52,080,508	PGSC0003DMG400018189	Pectinesterase inhibitor
ST4.03ch03	52,778,937..52,780,494	PGSC0003DMG400018093	Fasciclin-like arabinogalactan protein 9
ST4.03ch03	52,906,784..52,909,093	PGSC0003DMG400018146	Pectinesterase
ST4.03ch03	53,068,010..53,070,815	PGSC0003DMG400018142	Pectate lyase
ST4.03ch03	54,961,544..54,963,584	PGSC0003DMG400024530	Protein COBRA
ST4.03ch03	54,963,884..54,966,403	PGSC0003DMG400024628	COBRA 3
ST4.03ch03	55,316,389..55,316,718	PGSC0003DMG400024646	Expansin
ST4.03ch03	55,320,358..55,324,943	PGSC0003DMG400024647	Expansin
ST4.03ch03	55,333,818..55,338,034	PGSC0003DMG400024648	Expansin
ST4.03ch03	55,854,740..55,856,726	PGSC0003DMG400019507	Expansin

## Data Availability

Field data were deposited at e!DAL—PGP Repository at the IPK, Gatersleben, Germany. Original data are available at https://doi.ipk-gatersleben.de/DOI/afa7cb69-224a-4f3b-b153-a013faff51c2/a0cd444d-0616-4ac3-91f4-e4ba987f5752/2/1847940088 (accessed on 3 June 2021). Whole-genome sequencing data for Albatros, Ramses and the two bulks are available in the NCBI Sequence Read Archive (https://www.ncbi.nlm.nih.gov/sra/, accessible upon publication date of this study) under the accession numbers SRR14400529 (Albatros), SRR14400528 (Ramses), SRR14400527 (drought-tolerant bulk) and SRR14400526 (drought-sensitive bulk).

## Supplementary Materials

The following are available online at https://www.mdpi.com/article/10.3390/ijms22116123/s1, Figure S1: Overall genetic maps for Albatros (A drought tolerant) and Ramses (R drought sensitive). Informative linkage group specific SSR-markers (STM, STI, STG, reviewed in Ghislain et al. [33]). Drought tolerance candidate gene specific SSR-markers (HRO_ [34] and AFLP-markers (E) were mapped in the F_1_ A × R. Parent Albatros (A) left, parent Ramses (R) right for all linkage groups LG1-12. Figure S2: Distribution of SSR- and AFLP-markers on the homoeologous chromosomes of Albatros (A × R, LG 1-6). Figure S3: Distribution of SSR- and AFLP-markers on the homoeologous chromosomes of Albatros (A × R, LG 7-12). Figure S4: Distribution of SSR- and AFLP-markers on the homoeologous chromosomes of Ramses (A × R, LG 1-6). Figure S5: Distribution of SSR- and AFLP-markers on the homoeologous chromosomes of Ramses (A × R, LG 7-12). Figure S6: DRYM, tuber starch content, tuber starch yield, metabolite and transcript QTL mapped on the 12 linkage groups in Albatros x Ramses. QTL mapping co-localization of drought tolerance associated DRYM index (95% confidence interval. blue), tuber starch parameters and tuber fresh weight (95% confidence interval green; red for normalized and mean QTL), transcripts (eQTL black) and metabolites (mQTL burgundy) are displayed on the 12 parental chromosomes (drought tolerant parent Albatros A at the top, drought sensitive parent Ramses R below; Co under control conditions, Ds under drought stress). Figure S7: Overview over the different types of mutations occurring between the drought tolerant and drought sensitive bulk at the DRYM QTL on LG3. Table S1: Linkage group specific SSR-markers (reviewed in Ghislain et al. [33]). Table S2: SSR-markers derived from candidate genes for drought tolerance [34] mapped in A × R. Table S3: Genes located under the standalone DRYM QTL on LG3 flanked by the SSR-markers STG0018_b (glutamine-rich protein, PGSC0003DMG400026490) and STM5115_D/E (glycerol kinase, PGSC0003DMG400014144). Table S4: Overview about the different mutation types and locations present in the genes under the DRYM QTL on LG3 (A). Table S5: Transcript data used for eQTL analysis in the F1 population A × R. Normalized transcript data (2−ΔCt values) for 43 marker genes were used from two trials, MPI Golm polytunnel (61711) and JKI shelter (68015), in the year 2014 measuring 95 A × R lines. For further details of the experiments see Haas et al. [38] and for details on the measurements see Sprenger et al. [14]. Missing data are presented by -99. Table S6: Transcripts derived from a set of marker candidates for drought tolerance [14] and respective eQTL overlapping with DRYM QTL. Table S7: Metabolite data used for mQTL analysis in the F_1_ population A × R. Normalized metabolite data for 15 metabolites were used from two trials (61711 (MPI Golm polytunnel) and 68015 (JKI shelter) in the year 2014 measuring 95 A × R clones. For further details of the drought experiments see Haas et al. [38]. Original intensities of each metabolite were normalized to the average original intensity (response) of all annotated metabolites in a sample and log_10_-transformed. Transformed data were corrected for analytical batch and sequence effects according to the procedure described in the methods section (missing data are given as -99 for TetraploidMap). Table S8: Selected metabolites previously identified as drought-responsive [13] and respective mQTL overlapping with DRYM QTL. Table S9: AFLP primer pairs used for the development of the genetic maps.
