# Supplementary material for: Genome-Wide Approach to Identify Quantitative Trait Loci for Drought Tolerance in Tetraploid Potato (Solanum tuberosum L.)
Source: Int J Mol Sci. 2021 Jun 7;22(11):6123. doi: 10.3390/ijms22116123 (PMC8201130; doi:10.3390/ijms22116123)
Supplement: Supplementary file 1 [file ijms-22-06123-s001.zip › ijms-1231666-supplementary.pdf]

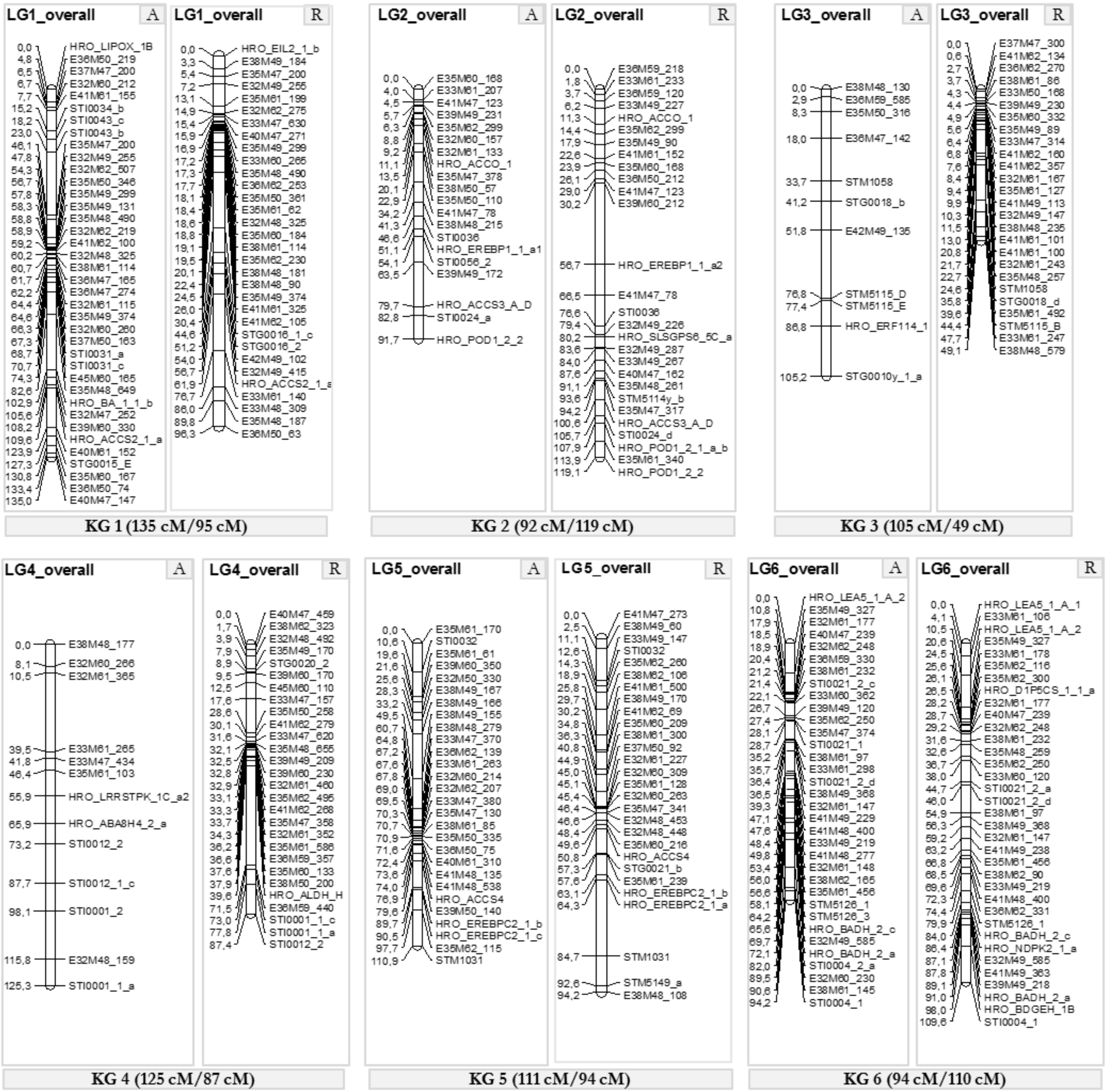

Figure S1: Overall genetic maps for Albatros (A drought tolerant) and Ramses (R drought sensitive). Informative linkage group specific SSR-markers (STM, STI, STG, reviewed in Ghislain *et al*. [33]). Drought tolerance candidate gene specific SSR-markers (HRO_, [34] and AFLP-markers (E) were mapped in the F_1 A×R. Parent Albatros (A) left, parent Ramses (R) right for all linkage groups LG1-12.

Figure S1 continued

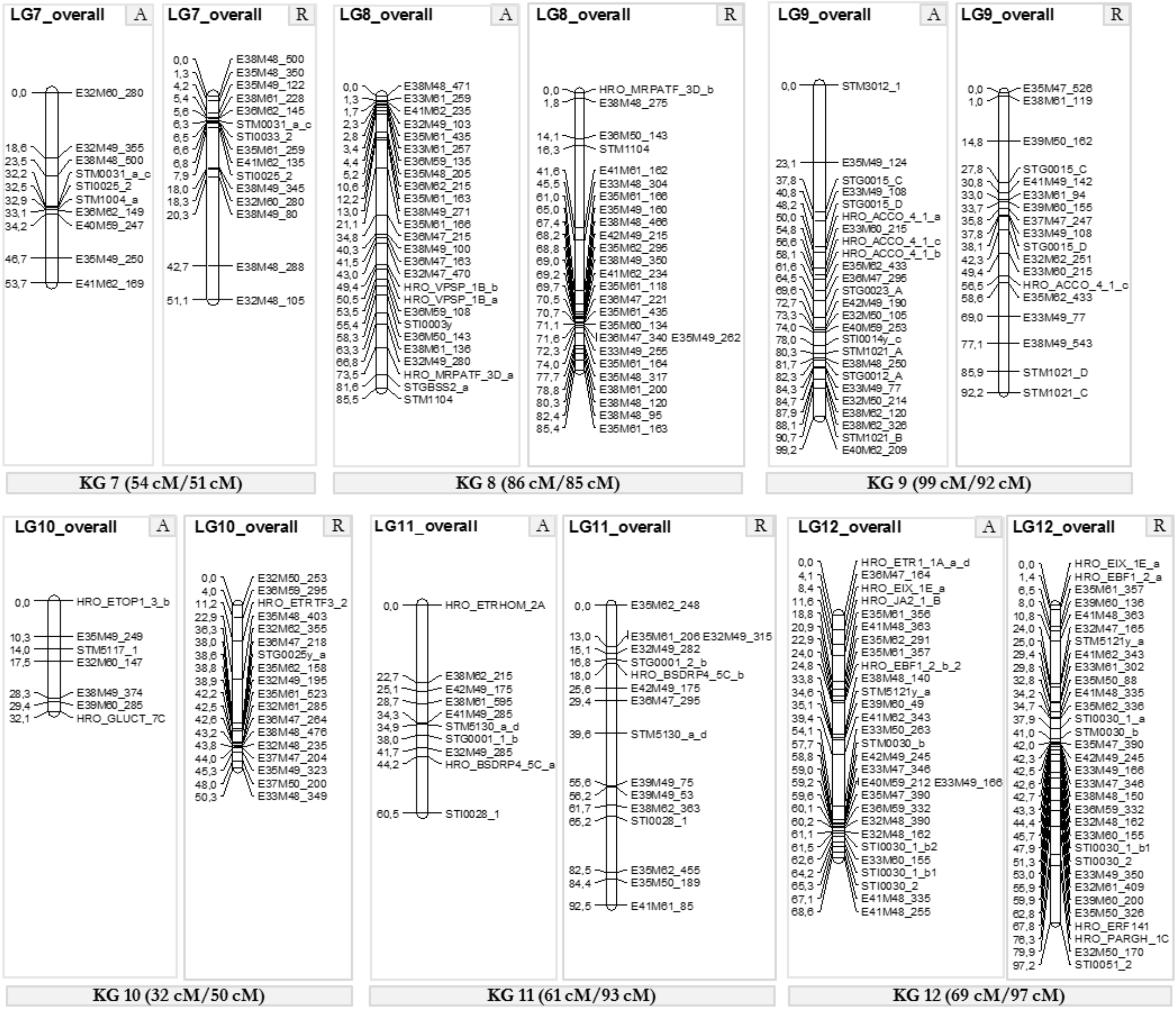

Distribution of SSR and AFLP markers on the homoeologous chromosomes in the F1-population A×R

A×R – Albatros:

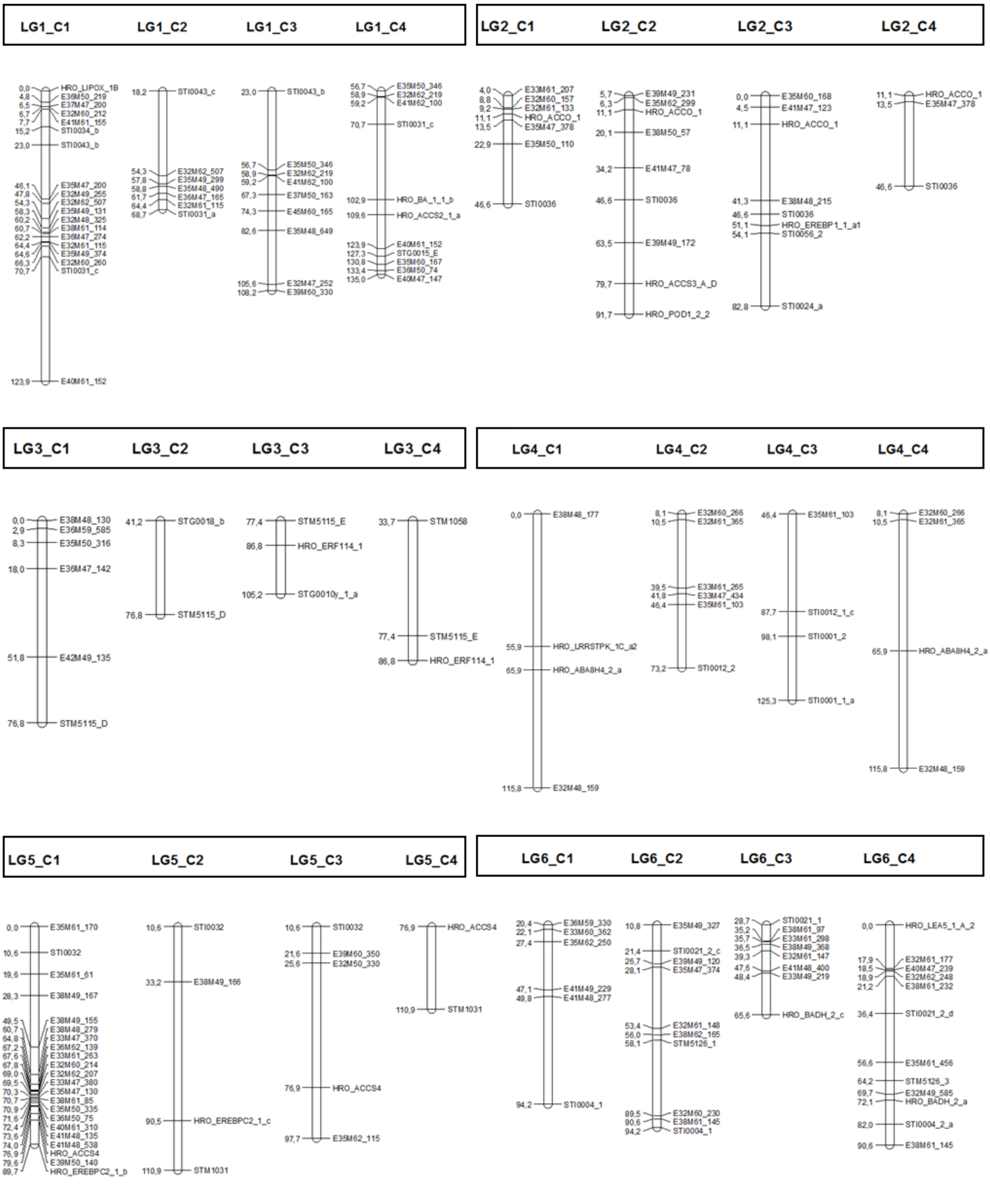

Figure S2: Distribution of SSR an AFLP markers on the homoeologous chromosomes of Albatros (A×R, LG 1-6)

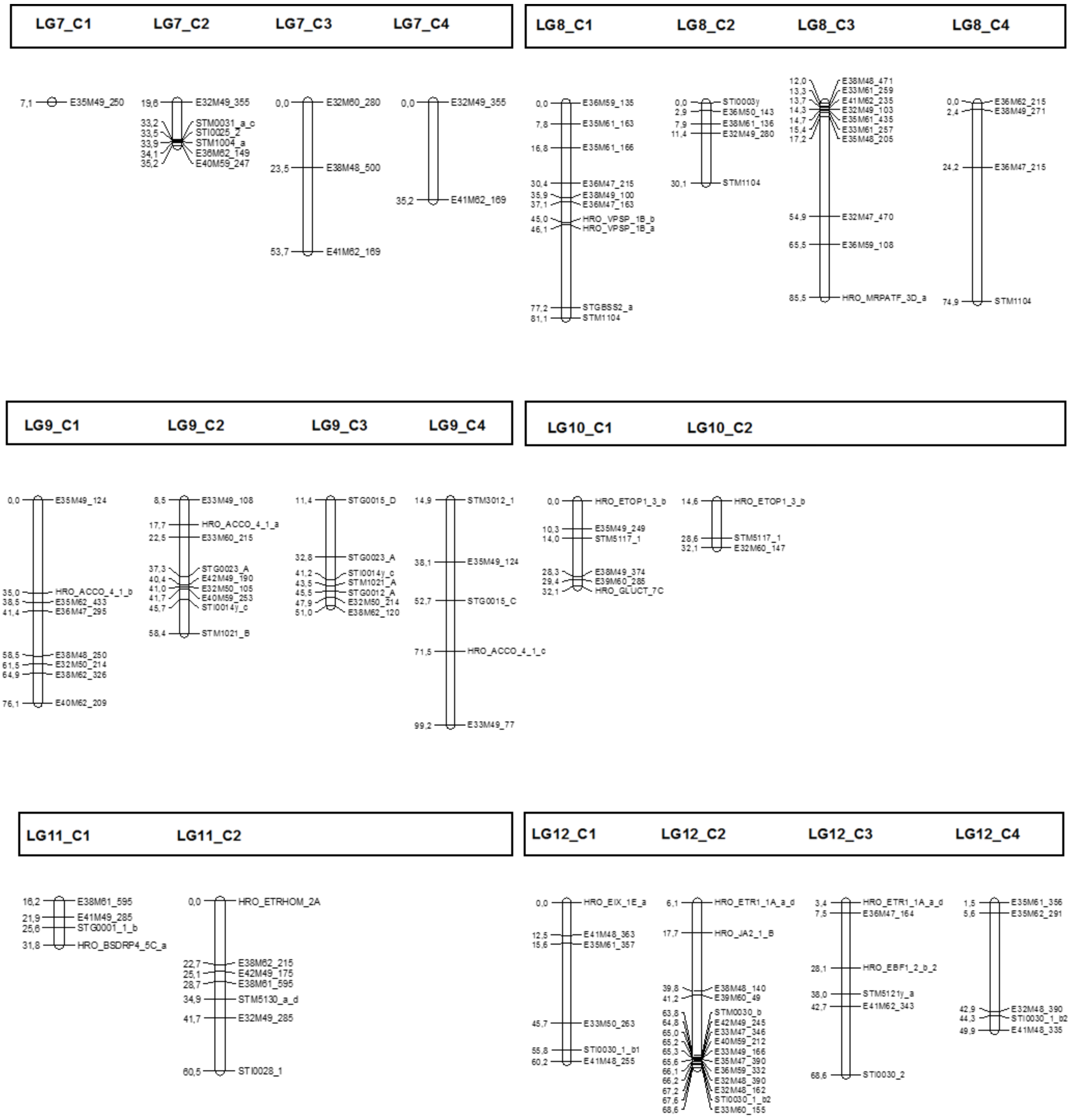

Figure S3: Distribution of SSR and AFLP markers on the homoeologous chromosomes of Albatros (A×R, LG 7-12)

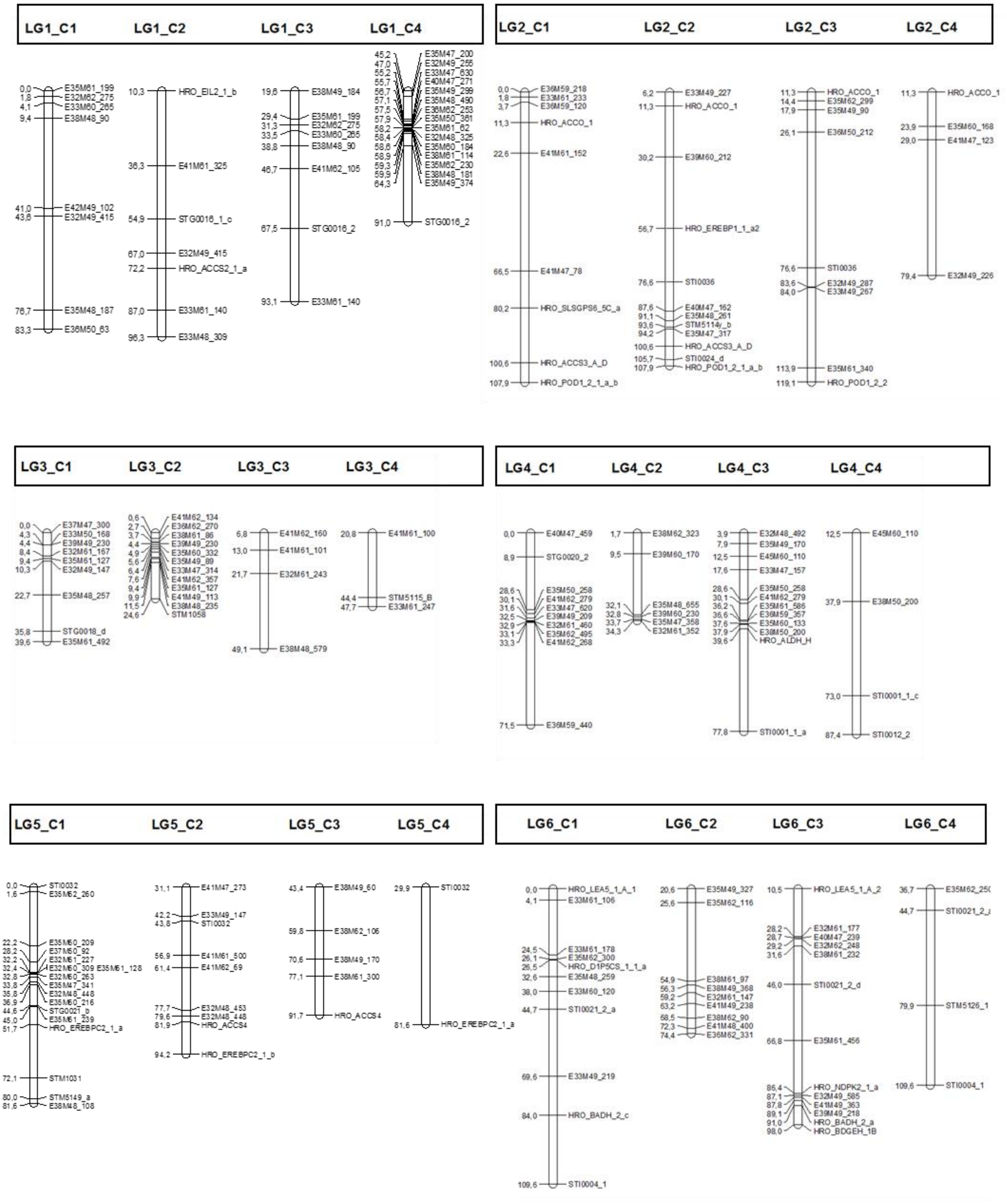

Figure S4: Distribution of SSR and AFLP markers on the homoeologous chromosomes of Ramses (A×R, LG 1-6)

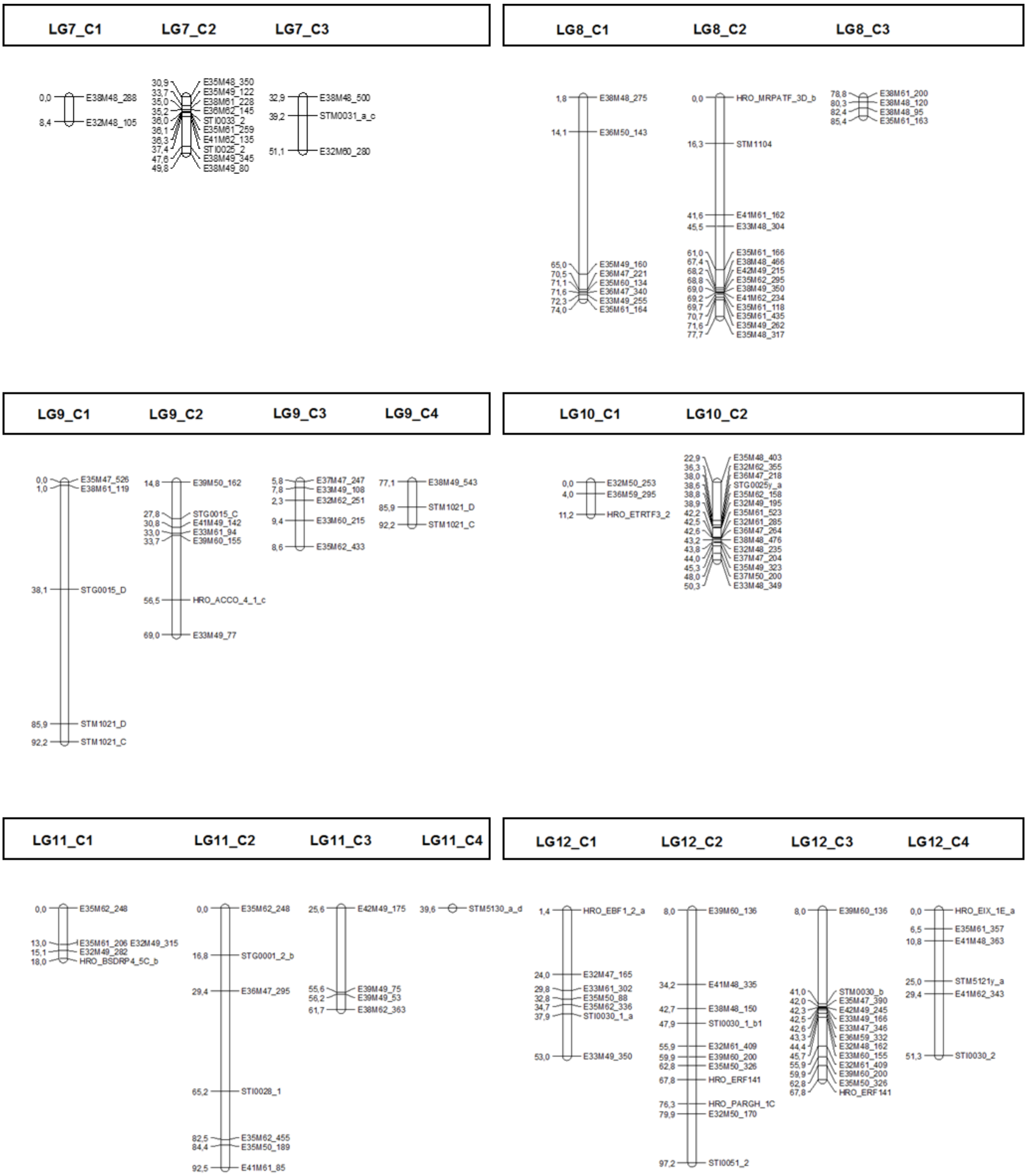

Figure S5: Distribution of SSR and AFLP markers on the homoeologous chromosomes of Ramses (A×R, LG 7-12)

Figure S6: DRYM, yield, metabolite and transcript QTL mapped on the 12 linkage groups in Albatros x Ramses. QTL mapping co-localisation of drought tolerance associated DRYM index (95 % confidence interval. blue), tuber starch parameters and tuber fresh weight (95 % confidence interval green; red for normalised and mean QTL), transcripts (eQTL black), and metabolites (mQTL dark red) on the 12 parental chromosomes (drought tolerant parent Albatros A at the top, drought sensitive parent Ramses R below). Metabolites (burgundy) and transcripts (black) are shown on the respective linkage groups LG1-LG12 (Co under control conditions, Ds under drought stress).

LG1 A

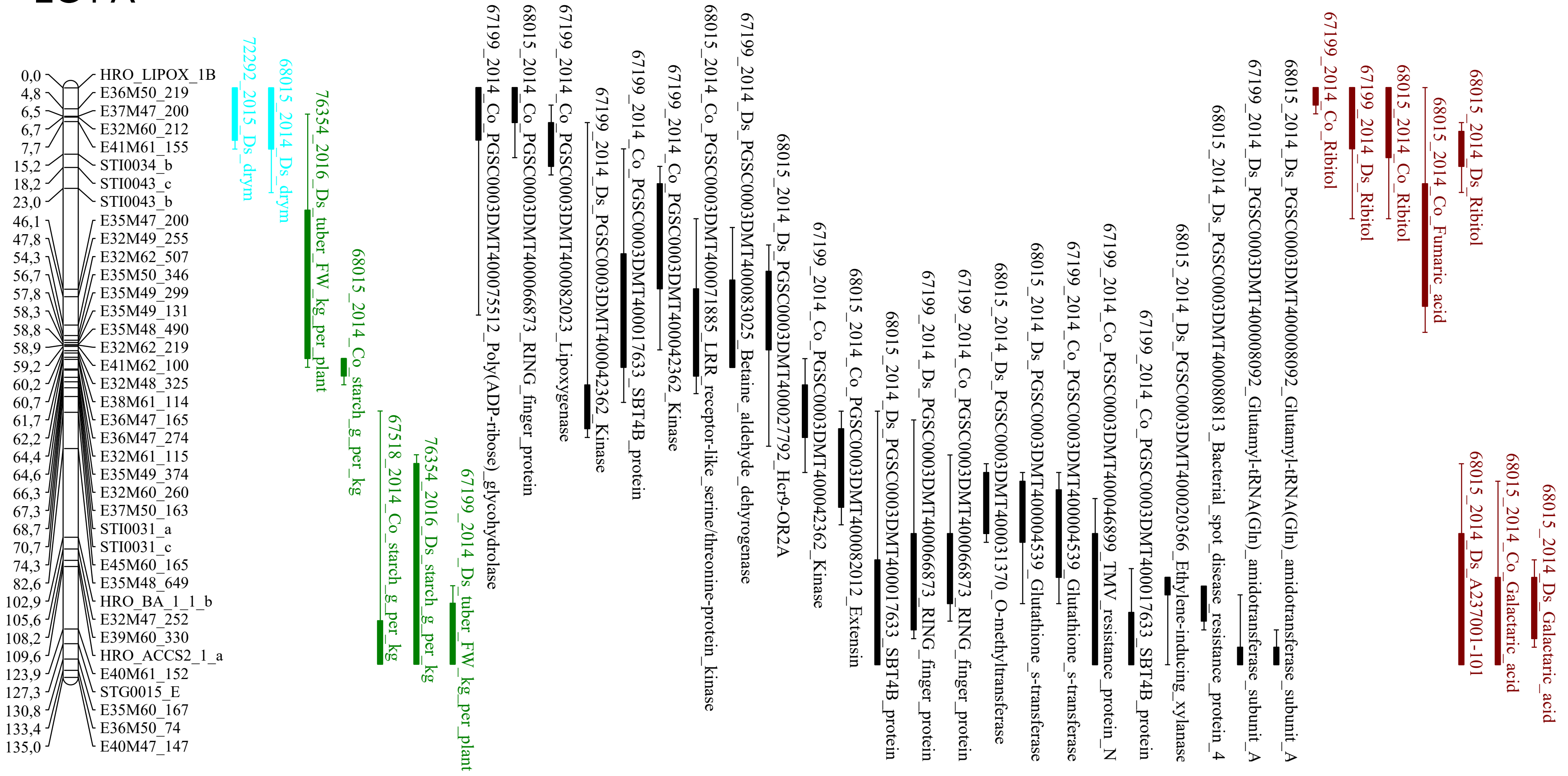

LG1 R

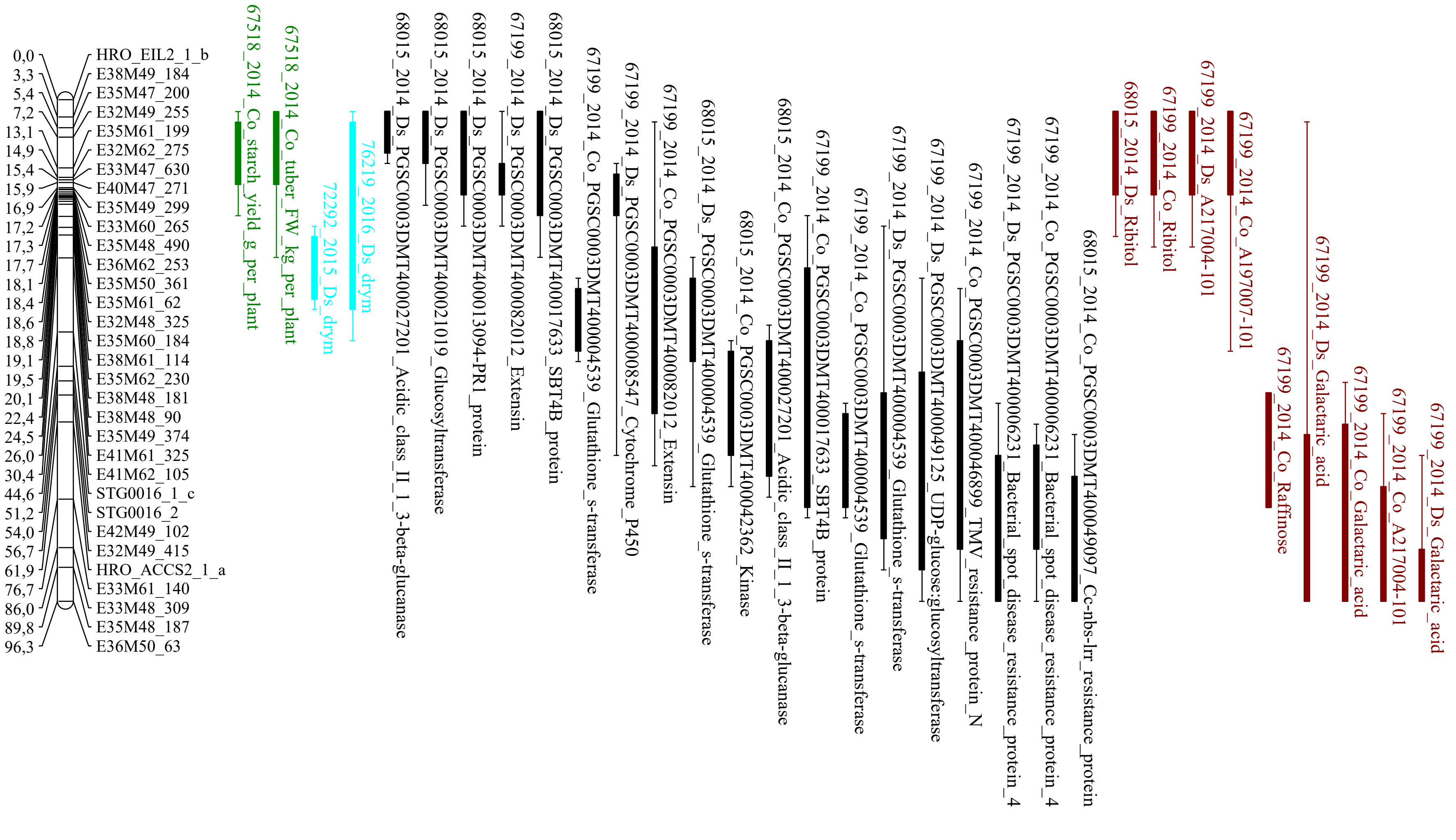

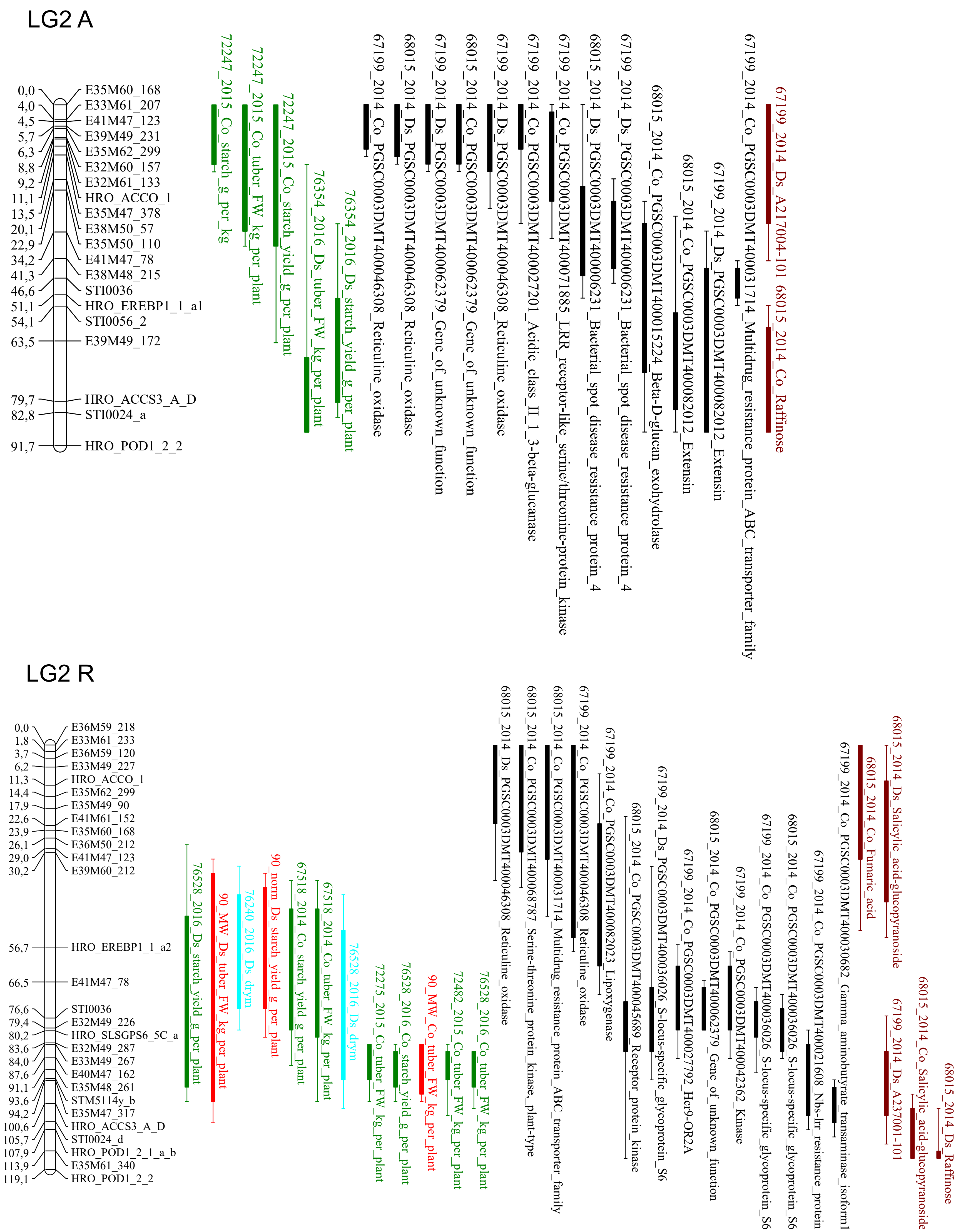
LG2 A
0,0 E35M60_168
4,0 E33M61_207
4,5 E41M47_123
5,7 E39M49_231
6,3 E35M62_299
8,8 E32M60_157
9,2 E32M61_133
11,1 HRO_ACCO_1
13,5 E35M47_378
20,1 E38M50_57
22,9 E35M50_110
34,2 E41M47_78
41,3 E38M48_215
46,6 STI0036
51,1 HRO_EREBP1_1_a1
54,1 STI0056_2
63,5 E39M49_172
79,7 HRO_ACCS3_A_D
82,8 STI0024_a
91,7 HRO_POD1_2_2
72247_2015_Co_starch_g_per_kg
72247_2015_Co_tuber_FW_kg_per_plant
72247_2015_Co_starch_yield_g_per_plant
76354_2016_Ds_tuber_FW_kg_per_plant
76354_2016_Ds_starch_yield_g_per_plant
67199_2014_Co_PGSC0003DMT400046308_Reticuline_oxidase
68015_2014_Ds_PGSC0003DMT400046308_Reticuline_oxidase
67199_2014_Ds_PGSC0003DMT400062379_Gene_of_unknown_function
68015_2014_Co_PGSC0003DMT400062379_Gene_of_unknown_function
67199_2014_Ds_PGSC0003DMT400046308_Reticuline_oxidase
67199_2014_Co_PGSC0003DMT400027201_Acidic_class_II_1_3-beta-glucanase
67199_2014_Co_PGSC0003DMT400071885_LRR_receptor-like_serine/threonine-protein_kinase
68015_2014_Ds_PGSC0003DMT400006231_Bacterial_spot_disease_resistance_protein_4
67199_2014_Ds_PGSC0003DMT400006231_Bacterial_spot_disease_resistance_protein_4
68015_2014_Co_PGSC0003DMT400015224_Beta-D-glucan_exohydrolase
68015_2014_Co_PGSC0003DMT400082012_Extensin
67199_2014_Ds_PGSC0003DMT400082012_Extensin
67199_2014_Co_PGSC0003DMT400031714_Multidrug_resistance_protein_ABC_transporter_family
67199_2014_Ds_A217004-101 68015_2014_Co_Raffinose
LG2 R
0,0 E36M59_218
1,8 E33M61_233
3,7 E36M59_120
6,2 E33M49_227
11,3 HRO_ACCO_1
14,4 E35M62_299
17,9 E35M49_90
22,6 E41M61_152
23,9 E35M60_168
26,1 E36M50_212
29,0 E41M47_123
30,2 E39M60_212
56,7 HRO_EREBP1_1_a2
66,5 E41M47_78
76,6 STI0036
79,4 E32M49_226
80,2 HRO_SLSGPS6_5C_a
83,6 E32M49_287
84,0 E33M49_267
87,6 E40M47_162
91,1 E35M48_261
93,6 STM5114y_b
94,2 E35M47_317
100,6 HRO_ACCS3_A_D
105,7 STI0024_d
107,9 HRO_POD1_2_1_a_b
113,9 E35M61_340
119,1 HRO_POD1_2_2
76528_2016_Ds_starch_yield_g_per_plant
90_MW_Ds_tuber_FW_kg_per_plant
76240_2016_Ds_drym
90_norm_Ds_starch_yield_g_per_plant
67518_2014_Co_starch_yield_g_per_plant
67518_2014_Co_tuber_FW_kg_per_plant
76528_2016_Ds_drym
72275_2015_Co_tuber_FW_kg_per_plant
76528_2016_Co_starch_yield_g_per_plant
90_MW_Co_tuber_FW_kg_per_plant
72482_2015_Co_tuber_FW_kg_per_plant
76528_2016_Co_tuber_FW_kg_per_plant
68015_2014_Ds_PGSC0003DMT400046308_Reticuline_oxidase
68015_2014_Co_PGSC0003DMT400068787_Serine-threonine_protein_kinase_plant-type
68015_2014_Co_PGSC0003DMT400031714_Multidrug_resistance_protein_ABC_transporter_family
67199_2014_Co_PGSC0003DMT400046308_Reticuline_oxidase
67199_2014_Co_PGSC0003DMT400082023_Lipoxygenase
68015_2014_Co_PGSC0003DMT400045689_Receptor_protein_kinase
67199_2014_Ds_PGSC0003DMT400036026_S-locus-specific_glycoprotein_S6
67199_2014_Co_PGSC0003DMT400027792_Hcr9-OR2A
68015_2014_Co_PGSC0003DMT400062379_Gene_of_unknown_function
67199_2014_Co_PGSC0003DMT400042362_Kinase
67199_2014_Co_PGSC0003DMT400036026_S-locus-specific_glycoprotein_S6
68015_2014_Co_PGSC0003DMT400036026_S-locus-specific_glycoprotein_S6
67199_2014_Co_PGSC0003DMT400021608_Nbs-lrr_resistance_protein
67199_2014_Co_PGSC0003DMT400030682_Gamma_aminobutyrate_transaminase_isoform1
68015_2014_Co_Fumaric_acid
68015_2014_Ds_Salicylic_acid-glucopyranoside
67199_2014_Ds_A237001-101
68015_2014_Co_Salicylic_acid-glucopyranoside
68015_2014_Ds_Raffinose

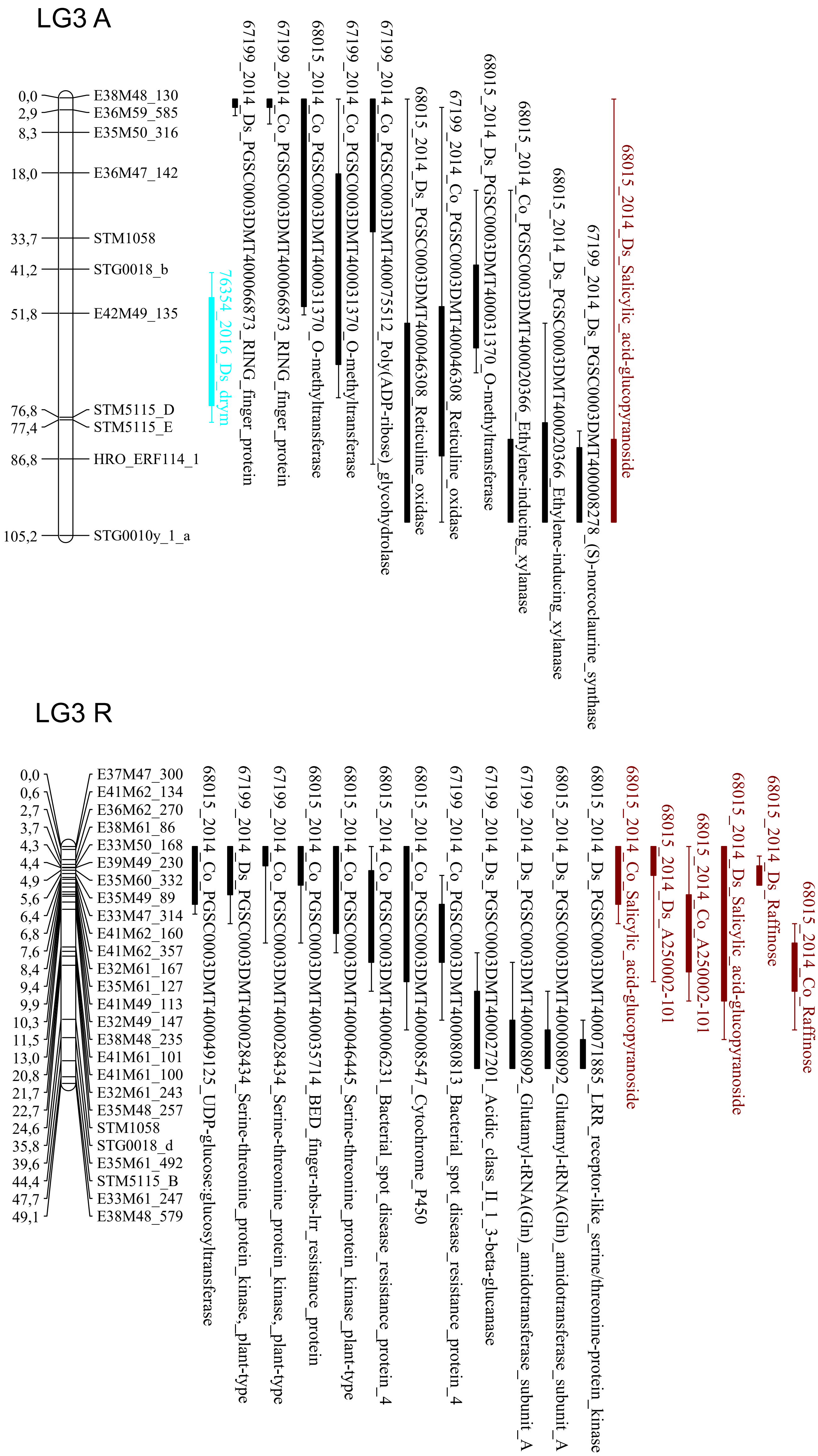

LG3 A
0,0 E38M48_130
2,9 E36M59_585
8,3 E35M50_316
18,0 E36M47_142
33,7 STM1058
41,2 STG0018_b
51,8 E42M49_135
76,8 STM5115_D
77,4 STM5115_E
86,8 HRO_ERF114_1
105,2 STG0010y_1_a
76354_2016_Ds_drym
67199_2014_Ds_PGSC0003DMT400066873_RING_finger_protein
67199_2014_Co_PGSC0003DMT400066873_RING_finger_protein
68015_2014_Co_PGSC0003DMT400031370_O-methyltransferase
67199_2014_Co_PGSC0003DMT400031370_O-methyltransferase
67199_2014_Co_PGSC0003DMT400075512_Poly(ADP-ribose)_glycohydrolase
68015_2014_Ds_PGSC0003DMT400046308_Reticuline_oxidase
67199_2014_Co_PGSC0003DMT400046308_Reticuline_oxidase
68015_2014_Ds_PGSC0003DMT400031370_O-methyltransferase
68015_2014_Co_PGSC0003DMT400020366_Ethylene-inducing_xylanase
68015_2014_Ds_PGSC0003DMT400020366_Ethylene-inducing_xylanase
67199_2014_Ds_PGSC0003DMT400008278_(S)-norcoclaurine_synthase
68015_2014_Ds_Salicylic_acid-glucopyranoside
LG3 R
0,0 E37M47_300
0,6 E41M62_134
2,7 E36M62_270
3,7 E38M61_86
4,3 E33M50_168
4,4 E39M49_230
4,9 E35M60_332
5,6 E35M49_89
6,4 E33M47_314
6,8 E41M62_160
7,6 E41M62_357
8,4 E32M61_167
9,4 E35M61_127
9,9 E41M49_113
10,3 E32M49_147
11,5 E38M48_235
13,0 E41M61_101
20,8 E41M61_100
21,7 E32M61_243
22,7 E35M48_257
24,6 STM1058
35,8 STG0018_d
39,6 E35M61_492
44,4 STM5115_B
47,7 E33M61_247
49,1 E38M48_579
68015_2014_Co_PGSC0003DMT400049125_UDP-glucose:glucosyltransferase
67199_2014_Ds_PGSC0003DMT400028434_Serine-threonine_protein_kinase,_plant-type
67199_2014_Co_PGSC0003DMT400028434_Serine-threonine_protein_kinase,_plant-type
68015_2014_Co_PGSC0003DMT400035714_BED_finger-nbs-lrr_resistance_protein
68015_2014_Co_PGSC0003DMT400046445_Serine-threonine_protein_kinase_plant-type
68015_2014_Co_PGSC0003DMT400006231_Bacterial_spot_disease_resistance_protein_4
68015_2014_Co_PGSC0003DMT400008547_Cytochrome_P450
67199_2014_Co_PGSC0003DMT400080813_Bacterial_spot_disease_resistance_protein_4
67199_2014_Ds_PGSC0003DMT400027201_Acidic_class_II_1_3-beta-glucanase
67199_2014_Ds_PGSC0003DMT400008092_Glutamyl-tRNA(Gln)_amidotransferase_subunit_A
68015_2014_Ds_PGSC0003DMT400008092_Glutamyl-tRNA(Gln)_amidotransferase_subunit_A
68015_2014_Ds_PGSC0003DMT400071885_LRR_receptor-like_serine/threonine-protein_kinase
68015_2014_Co_Salicylic_acid-glucopyranoside
68015_2014_Ds_A250002-101
68015_2014_Co_A250002-101
68015_2014_Ds_Salicylic_acid-glucopyranoside
68015_2014_Ds_Raffinose
68015_2014_Co_Raffinose

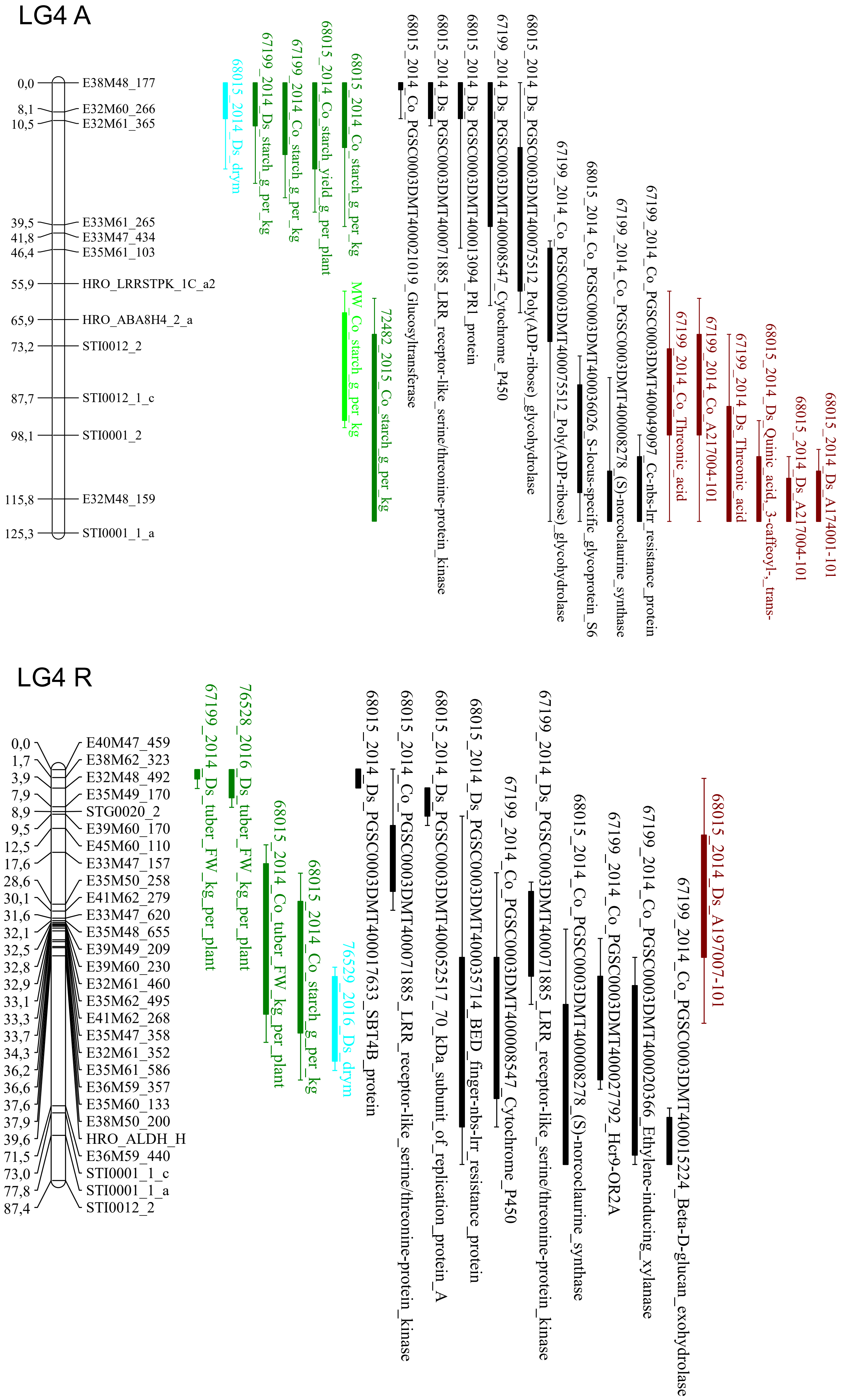

LG4 A
0,0 E38M48_177
8,1 E32M60_266
10,5 E32M61_365
39,5 E33M61_265
41,8 E33M47_434
46,4 E35M61_103
55,9 HRO_LRRSTPK_1C_a2
65,9 HRO_ABA8H4_2_a
73,2 STI0012_2
87,7 STI0012_1_c
98,1 STI0001_2
115,8 E32M48_159
125,3 STI0001_1_a
68015_2014_Ds_drym
67199_2014_Ds_starch_g_per_kg
67199_2014_Co_starch_g_per_kg
68015_2014_Co_starch_yield_g_per_plant
68015_2014_Co_starch_g_per_kg
MW_Co_starch_g_per_kg
72482_2015_Co_starch_g_per_kg
68015_2014_Co_PGSC0003DMT400021019_Glucosyltransferase
68015_2014_Ds_PGSC0003DMT400071885_LRR_receptor-like_serine/threonine-protein_kinase
68015_2014_Ds_PGSC0003DMT400013094_PR1_protein
67199_2014_Ds_PGSC0003DMT400008547_Cytochrome_P450
68015_2014_Ds_PGSC0003DMT400075512_Poly(ADP-ribose)_glycohydrolase
67199_2014_Co_PGSC0003DMT400075512_Poly(ADP-ribose)_glycohydrolase
68015_2014_Co_PGSC0003DMT400036026_S-locus-specific_glycoprotein_S6
67199_2014_Co_PGSC0003DMT400008278_(S)-norcoclaurine_synthase
67199_2014_Co_PGSC0003DMT400049097_Cc-nbs-lrr_resistance_protein
67199_2014_Co_Threonic_acid
67199_2014_Co_A217004-101
67199_2014_Ds_Threonic_acid
68015_2014_Ds_Quinic_acid,_3-caffeoyl-,_trans-
68015_2014_Ds_A217004-101
68015_2014_Ds_A174001-101
LG4 R
0,0 E40M47_459
1,7 E38M62_323
3,9 E32M48_492
7,9 E35M49_170
8,9 STG0020_2
9,5 E39M60_170
12,5 E45M60_110
17,6 E33M47_157
28,6 E35M50_258
30,1 E41M62_279
31,6 E33M47_620
32,1 E35M48_655
32,5 E39M49_209
32,8 E39M60_230
32,9 E32M61_460
33,1 E35M62_495
33,3 E41M62_268
33,7 E35M47_358
34,3 E32M61_352
36,2 E35M61_586
36,6 E36M59_357
37,6 E35M60_133
37,9 E38M50_200
39,6 HRO_ALDH_H
71,5 E36M59_440
73,0 STI0001_1_c
77,8 STI0001_1_a
87,4 STI0012_2
67199_2014_Ds_tuber_FW_kg_per_plant
76528_2016_Ds_tuber_FW_kg_per_plant
68015_2014_Co_tuber_FW_kg_per_plant
68015_2014_Co_starch_g_per_kg
76529_2016_Ds_drym
68015_2014_Ds_PGSC0003DMT400017633_SBT4B_protein
68015_2014_Co_PGSC0003DMT400071885_LRR_receptor-like_serine/threonine-protein_kinase
68015_2014_Ds_PGSC0003DMT400052517_70_kDa_subunit_of_replication_protein_A
68015_2014_Ds_PGSC0003DMT400035714_BED_finger-nbs-lrr_resistance_protein
67199_2014_Co_PGSC0003DMT400008547_Cytochrome_P450
67199_2014_Ds_PGSC0003DMT400071885_LRR_receptor-like_serine/threonine-protein_kinase
68015_2014_Co_PGSC0003DMT400008278_(S)-norcoclaurine_synthase
67199_2014_Co_PGSC0003DMT400027792_Hcr9-OR2A
67199_2014_Co_PGSC0003DMT400020366_Ethylene-inducing_xylanase
67199_2014_Co_PGSC0003DMT400015224_Beta-D-glucan_exohydrolase
68015_2014_Ds_A197007-101

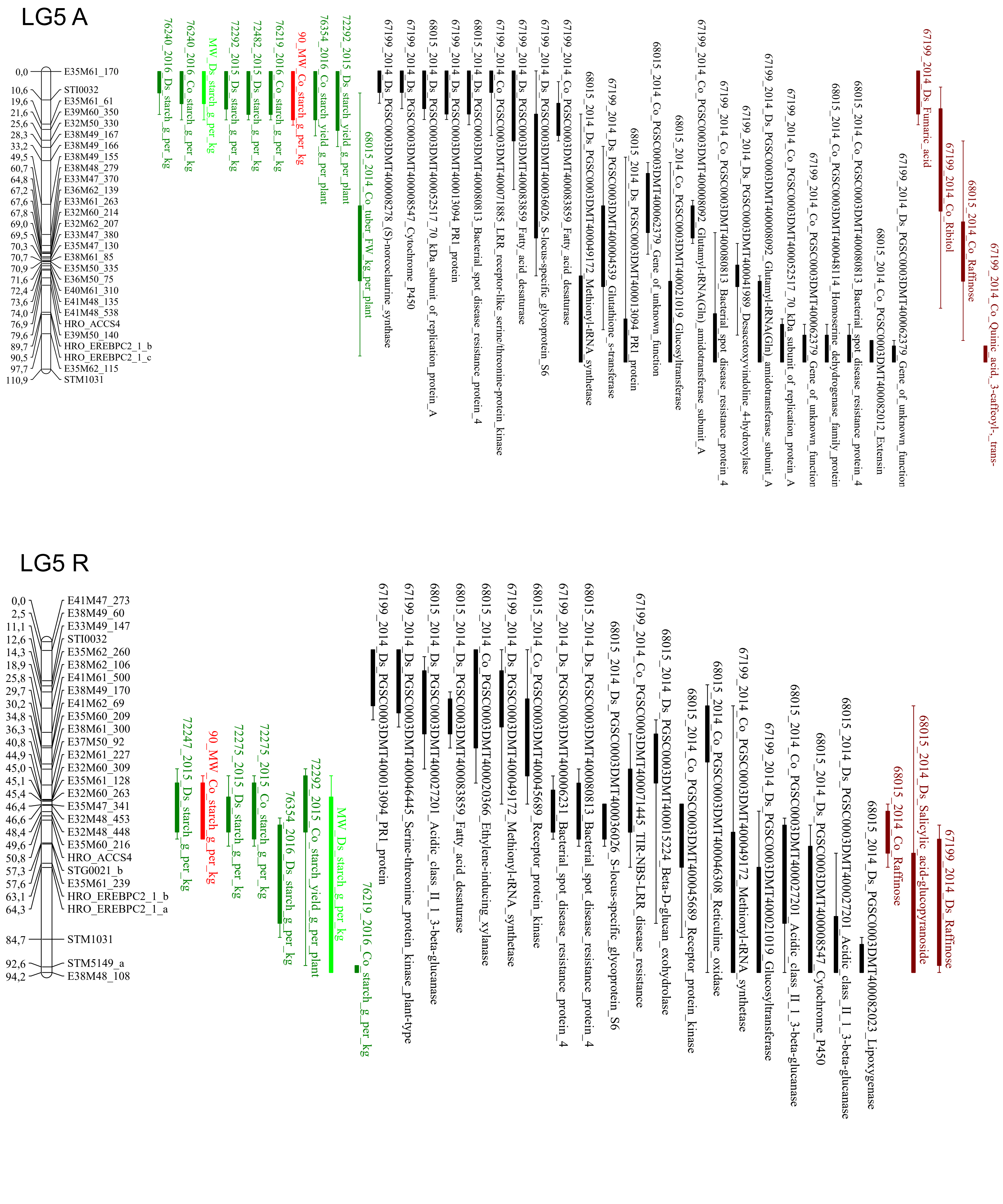
LG5 A
0,0 E35M61_170
10,6 STI0032
19,6 E35M61_61
21,6 E39M60_350
25,6 E32M50_330
28,3 E38M49_167
33,2 E38M49_166
49,5 E38M49_155
60,7 E38M48_279
64,8 E33M47_370
67,2 E36M62_139
67,6 E33M61_263
67,8 E32M60_214
69,0 E32M62_207
69,5 E33M47_380
70,3 E35M47_130
70,7 E38M61_85
70,9 E35M50_335
71,6 E36M50_75
72,4 E40M61_310
73,6 E41M48_135
74,0 E41M48_538
76,9 HRO_ACCS4
79,6 E39M50_140
89,7 HRO_EREBPC2_1_b
90,5 HRO_EREBPC2_1_c
97,7 E35M62_115
110,9 STM1031
76240_2016_Ds_starch_g_per_kg
76240_2016_Co_starch_g_per_kg
MW_Ds_starch_g_per_kg
72292_2015_Ds_starch_g_per_kg
72482_2015_Ds_starch_g_per_kg
76219_2016_Co_starch_g_per_kg
90_MW_Co_starch_g_per_kg
76354_2016_Co_starch_yield_g_per_plant
72292_2015_Ds_starch_yield_g_per_plant
68015_2014_Co_tuber_FW_kg_per_plant
67199_2014_Ds_PGSC0003DMT400008278_(S)-norcoclaurine_synthase
67199_2014_Ds_PGSC0003DMT400008547_Cytochrome_P450
68015_2014_Ds_PGSC0003DMT400052517_70_kDa_subunit_of_replication_protein_A
67199_2014_Ds_PGSC0003DMT400013094_PR1_protein
68015_2014_Ds_PGSC0003DMT400080813_Bacterial_spot_disease_resistance_protein_4
67199_2014_Co_PGSC0003DMT400071885_LRR_receptor-like_serine/threonine-protein_kinase
67199_2014_Ds_PGSC0003DMT400083859_Fatty_acid_desaturase
67199_2014_Ds_PGSC0003DMT400036026_S-locus-specific_glycoprotein_S6
67199_2014_Co_PGSC0003DMT400083859_Fatty_acid_desaturase
68015_2014_Ds_PGSC0003DMT400049172_Methionyl-tRNA_synthetase
67199_2014_Ds_PGSC0003DMT400004539_Glutathione_s-transferase
68015_2014_Ds_PGSC0003DMT400013094_PR1_protein
68015_2014_Co_PGSC0003DMT400062379_Gene_of_unknown_function
68015_2014_Co_PGSC0003DMT400021019_Glucosyltransferase
67199_2014_Co_PGSC0003DMT400008092_Glutamyl-tRNA(Gln)_amidotransferase_subunit_A
67199_2014_Co_PGSC0003DMT400080813_Bacterial_spot_disease_resistance_protein_4
67199_2014_Ds_PGSC0003DMT400041989_Desacetoxyvindoline_4-hydroxylase
67199_2014_Ds_PGSC0003DMT400008092_Glutamyl-tRNA(Gln)_amidotransferase_subunit_A
67199_2014_Co_PGSC0003DMT400052517_70_kDa_subunit_of_replication_protein_A
67199_2014_Co_PGSC0003DMT400062379_Gene_of_unknown_function
68015_2014_Co_PGSC0003DMT400048114_Homoserine_dehydrogenase_family_protein
68015_2014_Co_PGSC0003DMT400080813_Bacterial_spot_disease_resistance_protein_4
68015_2014_Co_PGSC0003DMT400082012_Extensin
67199_2014_Ds_PGSC0003DMT400062379_Gene_of_unknown_function
67199_2014_Ds_Fumaric acid
67199_2014_Co_Ribitol
68015_2014_Co_Raffinose
67199_2014_Co_Quinic acid,_3-caffeoyl-,_trans-
LG5 R
0,0 E41M47_273
2,5 E38M49_60
11,1 E33M49_147
12,6 STI0032
14,3 E35M62_260
18,9 E38M62_106
25,8 E41M61_500
29,7 E38M49_170
30,2 E41M62_69
34,8 E35M60_209
36,3 E38M61_300
40,8 E37M50_92
44,9 E32M61_227
45,0 E32M60_309
45,1 E35M61_128
45,4 E32M60_263
46,4 E35M47_341
46,6 E32M48_453
48,4 E32M48_448
49,6 E35M60_216
50,8 HRO_ACCS4
57,3 STG0021_b
57,6 E35M61_239
63,1 HRO_EREBPC2_1_b
64,3 HRO_EREBPC2_1_a
84,7 STM1031
92,6 STM5149_a
94,2 E38M48_108
72247_2015_Ds_starch_g_per_kg
90_MW_Co_starch_g_per_kg
72275_2015_Ds_starch_g_per_kg
72275_2015_Co_starch_g_per_kg
76354_2016_Ds_starch_g_per_kg
72292_2015_Co_starch_yield_g_per_plant
MW_Ds_starch_g_per_kg
76219_2016_Co_starch_g_per_kg
67199_2014_Ds_PGSC0003DMT400013094_PR1_protein
67199_2014_Ds_PGSC0003DMT400004645_Serine-threonine_protein_kinase_plant-type
68015_2014_Ds_PGSC0003DMT400027201_Acidic_class_II_1_3-beta-glucanase
68015_2014_Ds_PGSC0003DMT400083859_Fatty_acid_desaturase
68015_2014_Co_PGSC0003DMT400020366_Ethylene-inducing_xylanase
67199_2014_Ds_PGSC0003DMT400049172_Methionyl-tRNA_synthetase
68015_2014_Co_PGSC0003DMT400045689_Receptor_protein_kinase
67199_2014_Ds_PGSC0003DMT400006231_Bacterial_spot_disease_resistance_protein_4
68015_2014_Ds_PGSC0003DMT400080813_Bacterial_spot_disease_resistance_protein_4
68015_2014_Ds_PGSC0003DMT400036026_S-locus-specific_glycoprotein_S6
67199_2014_Co_PGSC0003DMT400071445_TIR-NBS-LRR_disease_resistance
68015_2014_Ds_PGSC0003DMT400015224_Beta-D-glucan_exohydrolase
68015_2014_Co_PGSC0003DMT400045689_Receptor_protein_kinase
68015_2014_Co_PGSC0003DMT400046308_Reticuline_oxidase
67199_2014_Co_PGSC0003DMT400049172_Methionyl-tRNA_synthetase
67199_2014_Ds_PGSC0003DMT400021019_Glucosyltransferase
68015_2014_Co_PGSC0003DMT400027201_Acidic_class_II_1_3-beta-glucanase
68015_2014_Ds_PGSC0003DMT400008547_Cytochrome_P450
68015_2014_Ds_PGSC0003DMT400027201_Acidic_class_II_1_3-beta-glucanase
68015_2014_Ds_PGSC0003DMT400082023_Lipoxygenase
68015_2014_Co_Raffinose
68015_2014_Ds_Salicylic_acid-glucopyranoside
67199_2014_Ds_Raffinose

LG6 A

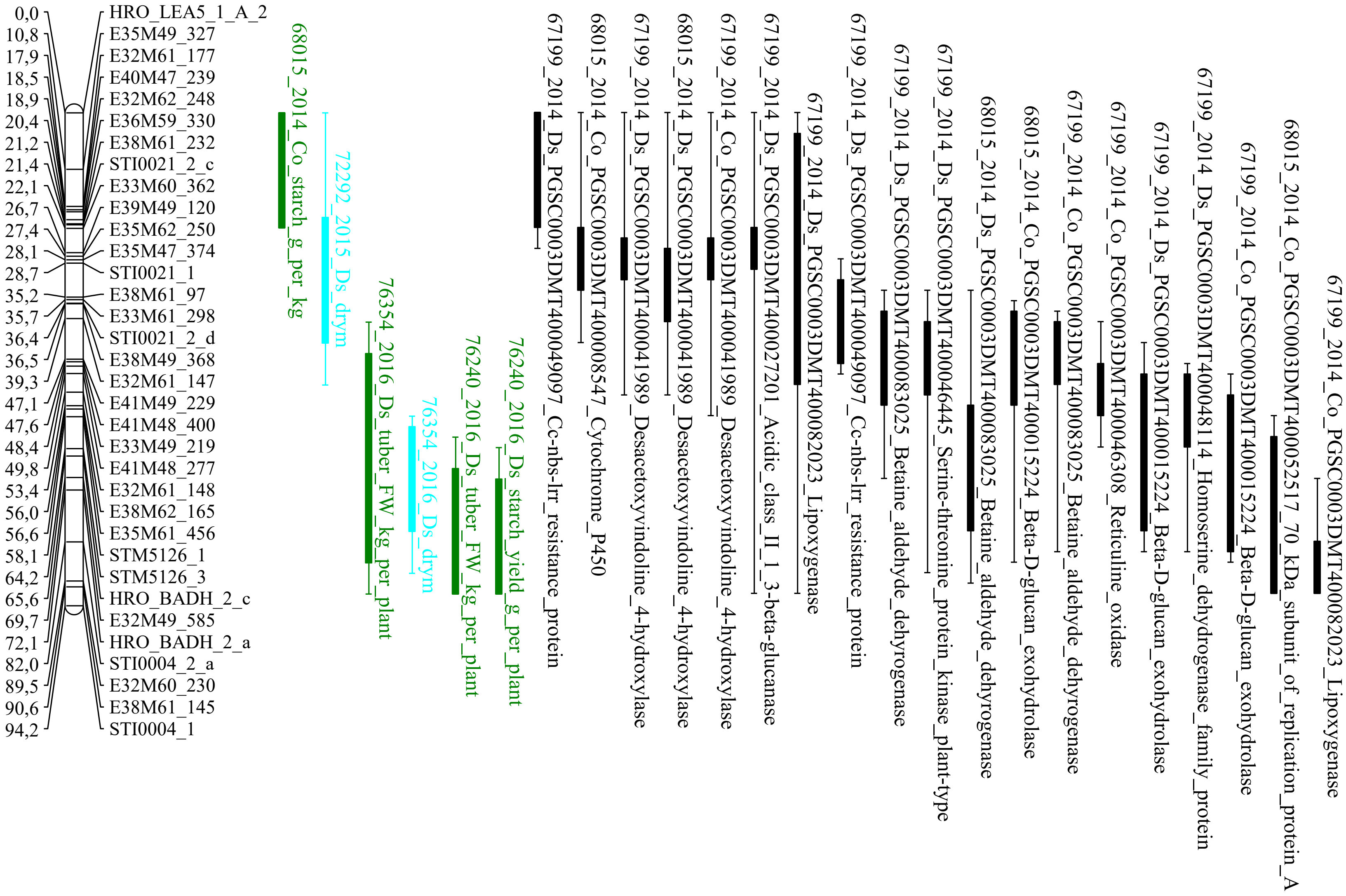

0,0 HRO_LEA5_1_A_2
10,8 E35M49_327
17,9 E32M61_177
18,5 E40M47_239
18,9 E32M62_248
20,4 E36M59_330
21,2 E38M61_232
21,4 STI0021_2_c
22,1 E33M60_362
26,7 E39M49_120
27,4 E35M62_250
28,1 E35M47_374
28,7 STI0021_1
35,2 E38M61_97
35,7 E33M61_298
36,4 STI0021_2_d
36,5 E38M49_368
39,3 E32M61_147
47,1 E41M49_229
47,6 E41M48_400
48,4 E33M49_219
49,8 E41M48_277
53,4 E32M61_148
56,0 E38M62_165
56,6 E35M61_456
58,1 STM5126_1
64,2 STM5126_3
65,6 HRO_BADH_2_c
69,7 E32M49_585
72,1 HRO_BADH_2_a
82,0 STI0004_2_a
89,5 E32M60_230
90,6 E38M61_145
94,2 STI0004_1
68015_2014_Co_starch_g_per_kg
72292_2015_Ds_drym
76354_2016_Ds_tuber_FW_kg_per_plant
76354_2016_Ds_drym
76240_2016_Ds_tuber_FW_kg_per_plant
76240_2016_Ds_starch_yield_g_per_plant
67199_2014_Ds_PGSC0003DMT400049097_Cc-nbs-lrr_resistance_protein
68015_2014_Co_PGSC0003DMT400008547_Cytochrome_P450
67199_2014_Ds_PGSC0003DMT400041989_Desacetoxyvindoline_4-hydroxylase
68015_2014_Ds_PGSC0003DMT400041989_Desacetoxyvindoline_4-hydroxylase
67199_2014_Co_PGSC0003DMT400041989_Desacetoxyvindoline_4-hydroxylase
67199_2014_Ds_PGSC0003DMT400027201_Acidic_class_II_1_3-beta-glucanase
67199_2014_Ds_PGSC0003DMT400082023_Lipoxygenase
67199_2014_Ds_PGSC0003DMT400049097_Cc-nbs-lrr_resistance_protein
67199_2014_Ds_PGSC0003DMT400083025_Betaine_aldehyde_dehyrogenase
67199_2014_Ds_PGSC0003DMT400046445_Serine-threonine_protein_kinase_plant-type
68015_2014_Ds_PGSC0003DMT400083025_Betaine_aldehyde_dehyrogenase
68015_2014_Co_PGSC0003DMT400015224_Beta-D-glucan_exohydrolase
67199_2014_Co_PGSC0003DMT400083025_Betaine_aldehyde_dehyrogenase
67199_2014_Co_PGSC0003DMT400046308_Reticuline_oxidase
67199_2014_Ds_PGSC0003DMT400015224_Beta-D-glucan_exohydrolase
67199_2014_Ds_PGSC0003DMT400048114_Homoserine_dehydrogenase_family_protein
67199_2014_Co_PGSC0003DMT400015224_Beta-D-glucan_exohydrolase
68015_2014_Co_PGSC0003DMT400052517_70_kDa_subunit_of_replication_protein_A
67199_2014_Co_PGSC0003DMT400082023_Lipoxygenase

LG6 R

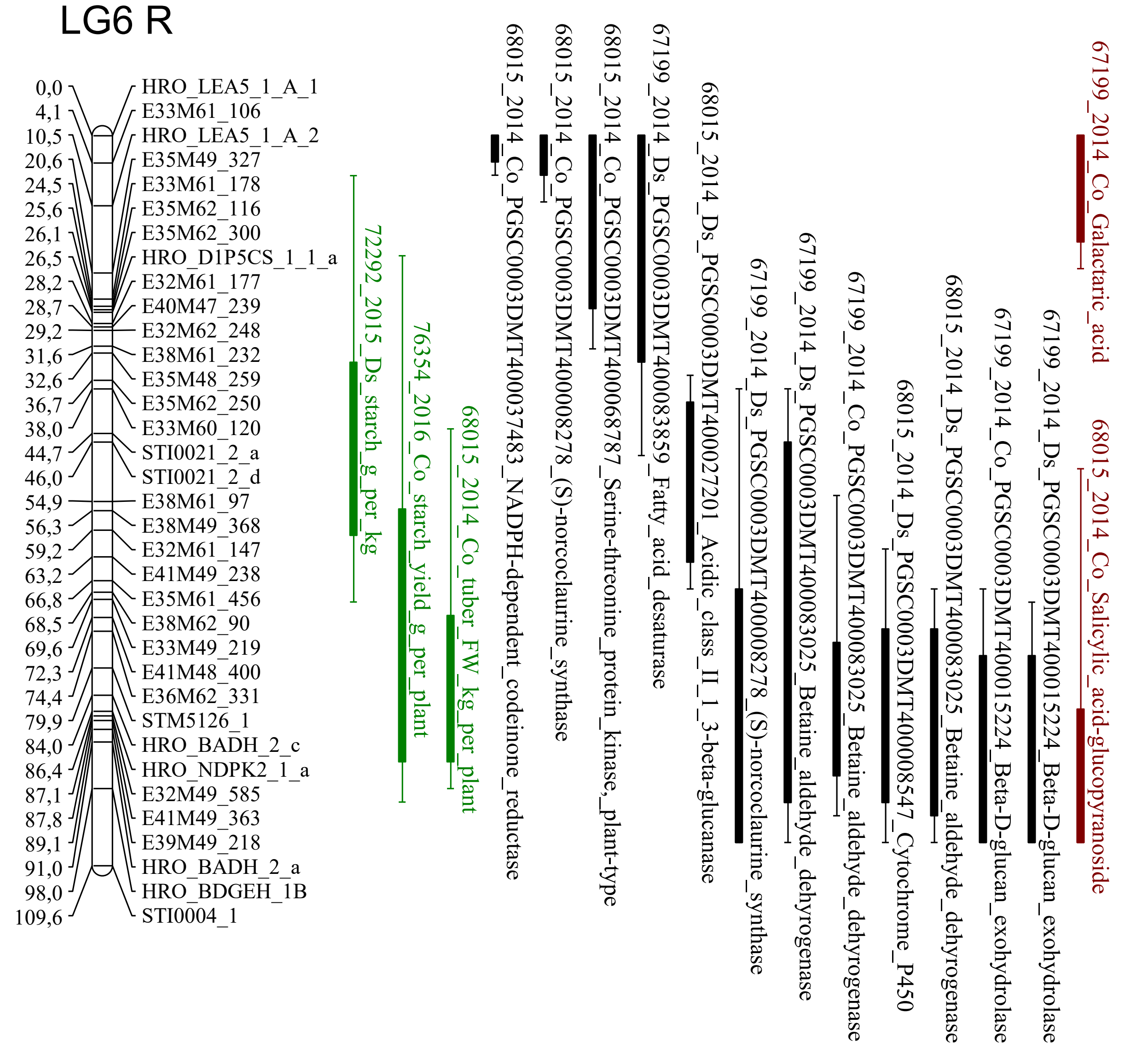

0,0 HRO_LEA5_1_A_1
4,1 E33M61_106
10,5 HRO_LEA5_1_A_2
20,6 E35M49_327
24,5 E33M61_178
25,6 E35M62_116
26,1 E35M62_300
26,5 HRO_D1P5CS_1_1_a
28,2 E32M61_177
28,7 E40M47_239
29,2 E32M62_248
31,6 E38M61_232
32,6 E35M48_259
36,7 E35M62_250
38,0 E33M60_120
44,7 STI0021_2_a
46,0 STI0021_2_d
54,9 E38M61_97
56,3 E38M49_368
59,2 E32M61_147
63,2 E41M49_238
66,8 E35M61_456
68,5 E38M62_90
69,6 E33M49_219
72,3 E41M48_400
74,4 E36M62_331
79,9 STM5126_1
84,0 HRO_BADH_2_c
86,4 HRO_NDPK2_1_a
87,1 E32M49_585
87,8 E41M49_363
89,1 E39M49_218
91,0 HRO_BADH_2_a
98,0 HRO_BDGEH_1B
109,6 STI0004_1
72292_2015_Ds_starch_g_per_kg
76354_2016_Co_starch_yield_g_per_plant
68015_2014_Co_tuber_FW_kg_per_plant
68015_2014_Co_PGSC0003DMT400037483_NADPH-dependent_codeinone_reductase
68015_2014_Co_PGSC0003DMT400008278_(S)-norcoclaurine_synthase
68015_2014_Co_PGSC0003DMT400046787_Serine-threonine_protein_kinase_plant-type
67199_2014_Ds_PGSC0003DMT400083859_Fatty_acid_desaturase
68015_2014_Ds_PGSC0003DMT400027201_Acidic_class_II_1_3-beta-glucanase
67199_2014_Ds_PGSC0003DMT400008278_(S)-norcoclaurine_synthase
67199_2014_Ds_PGSC0003DMT400083025_Betaine_aldehyde_dehyrogenase
67199_2014_Co_PGSC0003DMT400083025_Betaine_aldehyde_dehyrogenase
68015_2014_Ds_PGSC0003DMT400008547_Cytochrome_P450
68015_2014_Ds_PGSC0003DMT400083025_Betaine_aldehyde_dehyrogenase
67199_2014_Co_PGSC0003DMT400015224_Beta-D-glucan_exohydrolase
67199_2014_Ds_PGSC0003DMT400015224_Beta-D-glucan_exohydrolase
67199_2014_Co_Galactaric_acid
68015_2014_Co_Salicylic_acid-glucopyranoside

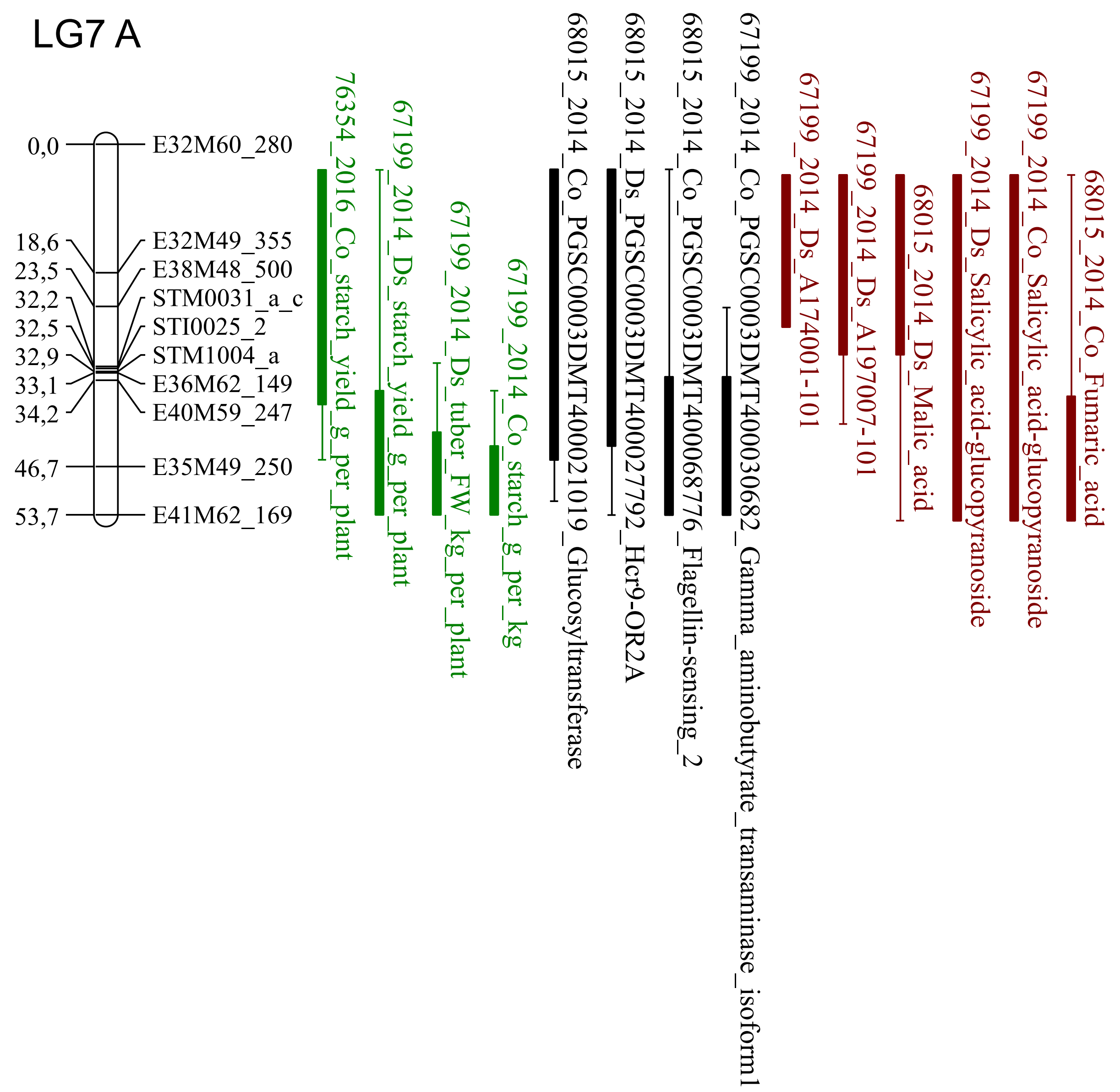
LG7 A
0,0 E32M60_280
18,6 E32M49_355
23,5 E38M48_500
32,2 STM0031_a_c
32,5 STI0025_2
32,9 STM1004_a
33,1 E36M62_149
34,2 E40M59_247
46,7 E35M49_250
53,7 E41M62_169
76354_2016_Co_starch_yield_g_per_plant
67199_2014_Ds_starch_yield_g_per_plant
67199_2014_Ds_tuber_FW_kg_per_plant
67199_2014_Co_starch_g_per_kg
68015_2014_Co_PGSC0003DMT400021019_Glucosyltransferase
68015_2014_Ds_PGSC0003DMT400027792_Hcr9-OR2A
68015_2014_Co_PGSC0003DMT400068776_Flagellin-sensing_2
67199_2014_Co_PGSC0003DMT400030682_Gamma_aminobutyrate_transaminase_isoform1
67199_2014_Ds_A174001-101
67199_2014_Ds_A197007-101
68015_2014_Ds_Malic_acid
67199_2014_Ds_Salicylic_acid-glucopyranoside
67199_2014_Co_Salicylic_acid-glucopyranoside
68015_2014_Co_Fumaric_acid

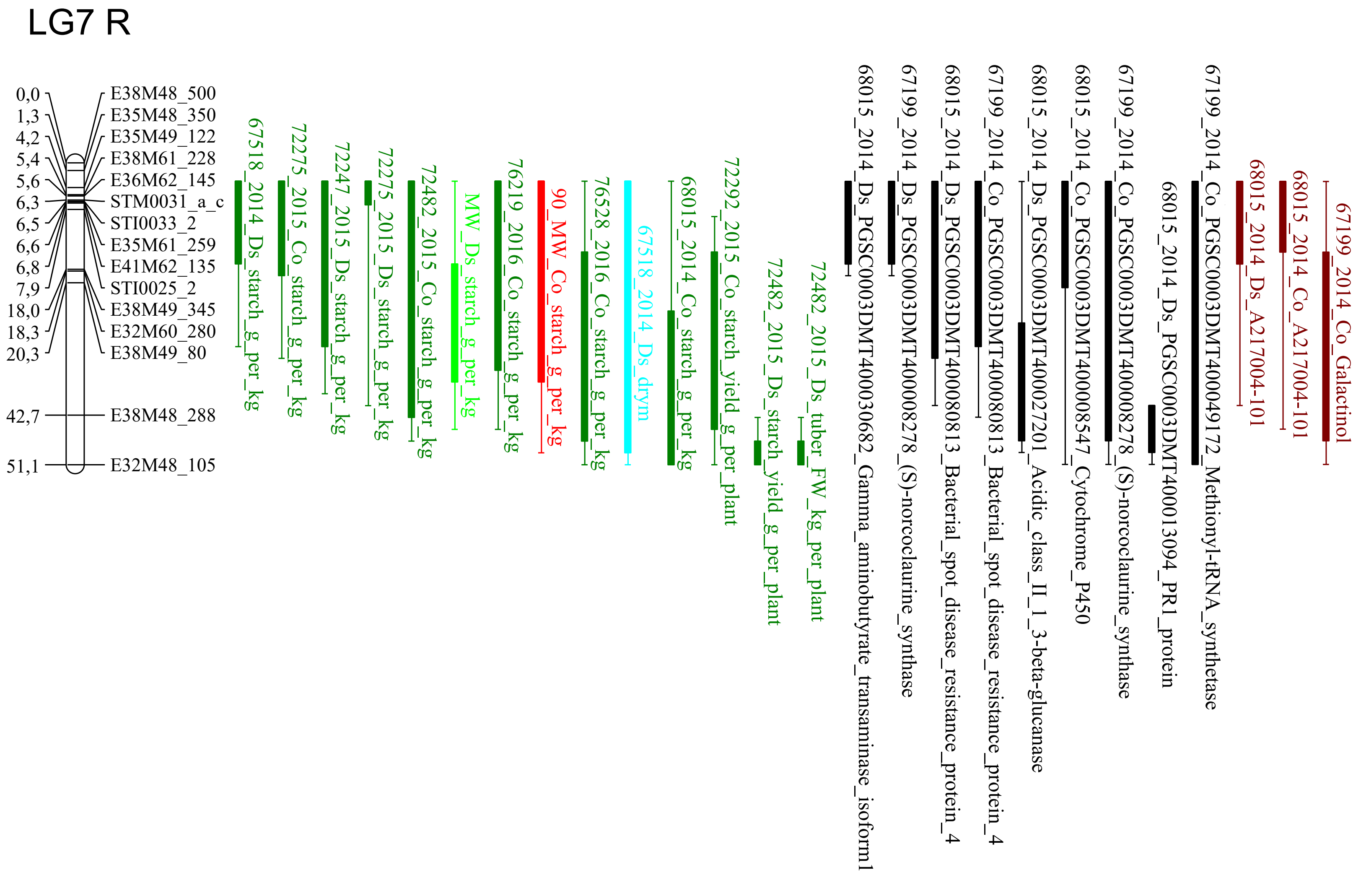
LG7 R
0,0 E38M48_500
1,3 E35M48_350
4,2 E35M49_122
5,4 E38M61_228
5,6 E36M62_145
6,3 STM0031_a_c
6,5 STI0033_2
6,6 E35M61_259
6,8 E41M62_135
7,9 STI0025_2
18,0 E38M49_345
18,3 E32M60_280
20,3 E38M49_80
42,7 E38M48_288
51,1 E32M48_105
67518_2014_Ds_starch_g_per_kg
72275_2015_Co_starch_g_per_kg
72247_2015_Ds_starch_g_per_kg
72275_2015_Ds_starch_g_per_kg
72482_2015_Co_starch_g_per_kg
MW_Ds_starch_g_per_kg
76219_2016_Co_starch_g_per_kg
90_MW_Co_starch_g_per_kg
76528_2016_Co_starch_g_per_kg
67518_2014_Ds_drym
68015_2014_Co_starch_g_per_kg
72292_2015_Co_starch_yield_g_per_plant
72482_2015_Ds_starch_yield_g_per_plant
72482_2015_Ds_tuber_FW_kg_per_plant
68015_2014_Ds_PGSC0003DMT400030682_Gamma_aminobutyrate_transaminase_isoform1
67199_2014_Ds_PGSC0003DMT400008278_(S)-norcoclaurine_synthase
68015_2014_Ds_PGSC0003DMT400080813_Bacterial_spot_disease_resistance_protein_4
67199_2014_Co_PGSC0003DMT400080813_Bacterial_spot_disease_resistance_protein_4
68015_2014_Ds_PGSC0003DMT400027201_Acidic_class_II_1_3-beta-glucanase
68015_2014_Co_PGSC0003DMT400008547_Cytochrome_P450
67199_2014_Co_PGSC0003DMT400008278_(S)-norcoclaurine_synthase
68015_2014_Ds_PGSC0003DMT400013094_PR1_protein
67199_2014_Co_PGSC0003DMT400049172_Methionyl-tRNA_synthetase
68015_2014_Ds_A217004-101
68015_2014_Co_A217004-101
67199_2014_Co_Galactinol

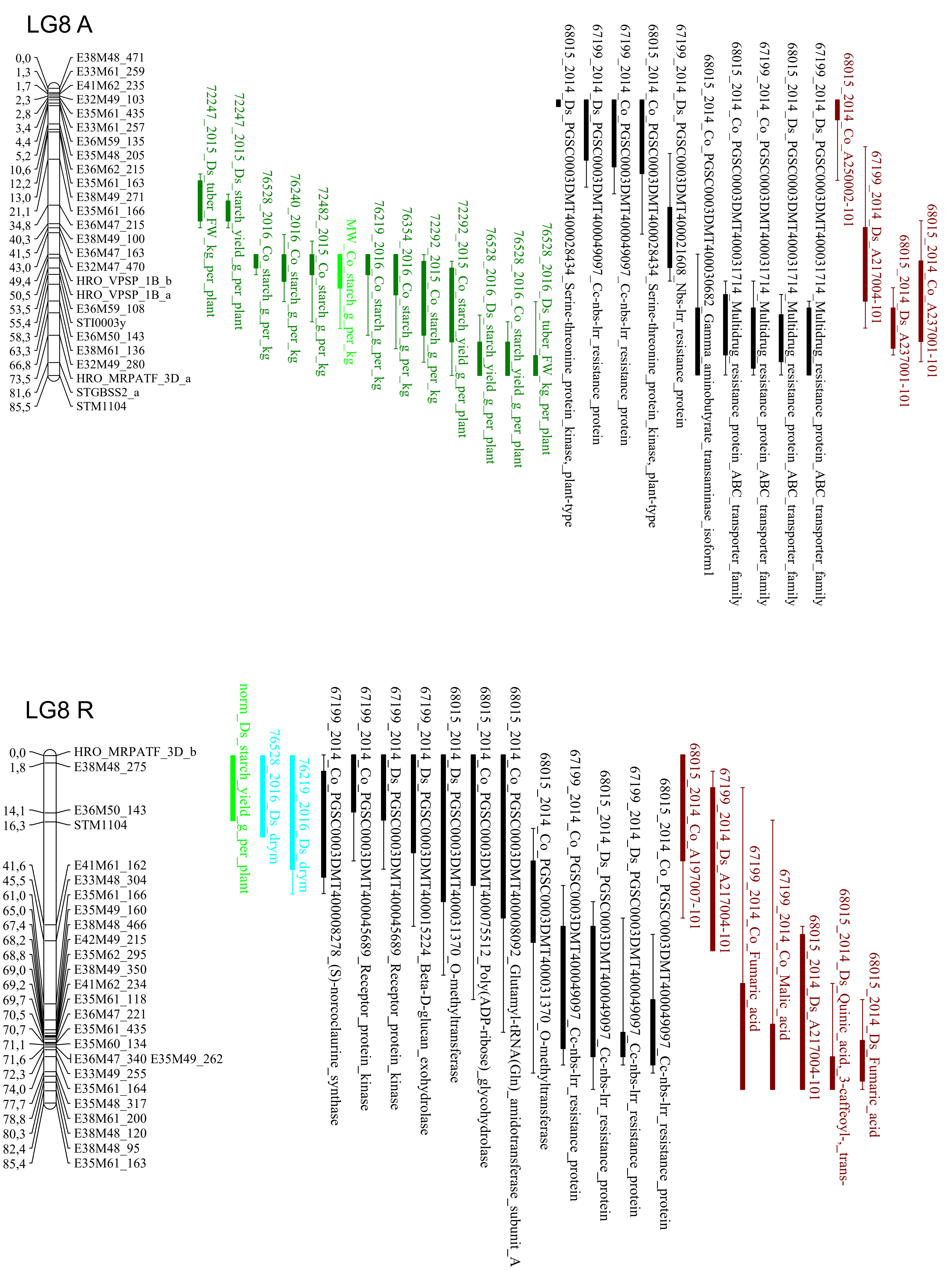

LG8 A
0,0 E38M48_471
1,3 E33M61_259
1,7 E41M62_235
2,3 E32M49_103
2,8 E35M61_435
3,4 E33M61_257
4,4 E36M59_135
5,2 E35M48_205
10,6 E36M62_215
12,2 E35M61_163
13,0 E38M49_271
21,1 E35M61_166
34,8 E36M47_215
40,3 E38M49_100
41,5 E36M47_163
43,0 E32M47_470
49,4 HRO_VPSP_1B_b
50,5 HRO_VPSP_1B_a
53,5 E36M59_108
55,4 STI0003y
58,3 E36M50_143
63,3 E38M61_136
66,8 E32M49_280
73,5 HRO_MRPATF_3D_a
81,6 STGBSS2_a
85,5 STM1104
72247_2015_Ds_tuber_FW_kg_per_plant
72247_2015_Ds_starch_yield_g_per_plant
76528_2016_Co_starch_g_per_kg
76240_2016_Co_starch_g_per_kg
72482_2015_Co_starch_g_per_kg
MW_Co_starch_g_per_kg
76219_2016_Co_starch_g_per_kg
76354_2016_Co_starch_g_per_kg
72292_2015_Co_starch_g_per_kg
72292_2015_Co_starch_yield_g_per_plant
76528_2016_Ds_starch_yield_g_per_plant
76528_2016_Co_starch_yield_g_per_plant
76528_2016_Ds_tuber_FW_kg_per_plant
68015_2014_Ds_PGSC0003DMT400028434_Serine-threonine_protein_kinase,_plant-type
67199_2014_Ds_PGSC0003DMT400049097_Cc-nbs-lrr_resistance_protein
67199_2014_Co_PGSC0003DMT400049097_Cc-nbs-lrr_resistance_protein
68015_2014_Co_PGSC0003DMT400028434_Serine-threonine_protein_kinase,_plant-type
67199_2014_Ds_PGSC0003DMT400021608_Nbs-lrr_resistance_protein
68015_2014_Co_PGSC0003DMT400030682_Gamma_aminobutyrate_transaminase_isoform1
68015_2014_Co_PGSC0003DMT400031714_Multidrug_resistance_protein_ABC_transporter_family
67199_2014_Co_PGSC0003DMT400031714_Multidrug_resistance_protein_ABC_transporter_family
68015_2014_Ds_PGSC0003DMT400031714_Multidrug_resistance_protein_ABC_transporter_family
67199_2014_Ds_PGSC0003DMT400031714_Multidrug_resistance_protein_ABC_transporter_family
68015_2014_Co_A250002-101
67199_2014_Ds_A217004-101
68015_2014_Ds_A237001-101
68015_2014_Co_A237001-101
LG8 R
0,0 HRO_MRPATF_3D_b
1,8 E38M48_275
14,1 E36M50_143
16,3 STM1104
41,6 E41M61_162
45,5 E33M48_304
61,0 E35M61_166
65,0 E35M49_160
67,4 E38M48_466
68,2 E42M49_215
68,8 E35M62_295
69,0 E38M49_350
69,2 E41M62_234
69,7 E35M61_118
70,5 E36M47_221
70,7 E35M61_435
71,1 E35M60_134
71,6 E36M47_340 E35M49_262
72,3 E33M49_255
74,0 E35M61_164
77,7 E35M48_317
78,8 E38M61_200
80,3 E38M48_120
82,4 E38M48_95
85,4 E35M61_163
norm_Ds_starch_yield_g_per_plant
76528_2016_Ds_drym
76219_2016_Ds_drym
67199_2014_Co_PGSC0003DMT400008278_(S)-norcoclaurine_synthase
67199_2014_Co_PGSC0003DMT400045689_Receptor_protein_kinase
67199_2014_Ds_PGSC0003DMT400045689_Receptor_protein_kinase
67199_2014_Ds_PGSC0003DMT400015224_Beta-D-glucan_exohydrolase
68015_2014_Ds_PGSC0003DMT400031370_O-methyltransferase
68015_2014_Co_PGSC0003DMT400075512_Poly(ADP-ribose)_glycohydrolase
68015_2014_Co_PGSC0003DMT400008092_Glutamyl-tRNA(Gln)_amidotransferase_subunit_A
68015_2014_Co_PGSC0003DMT400031370_O-methyltransferase
67199_2014_Co_PGSC0003DMT400049097_Cc-nbs-lrr_resistance_protein
68015_2014_Ds_PGSC0003DMT400049097_Cc-nbs-lrr_resistance_protein
67199_2014_Ds_PGSC0003DMT400049097_Cc-nbs-lrr_resistance_protein
68015_2014_Co_PGSC0003DMT400049097_Cc-nbs-lrr_resistance_protein
68015_2014_Co_A197007-101
67199_2014_Ds_A217004-101
67199_2014_Co_Fumaric_acid
67199_2014_Co_Malic_acid
68015_2014_Ds_A217004-101
68015_2014_Ds_Quinic_acid,_3-caffeoyl-,_trans-
68015_2014_Ds_Fumaric_acid

LG9 A

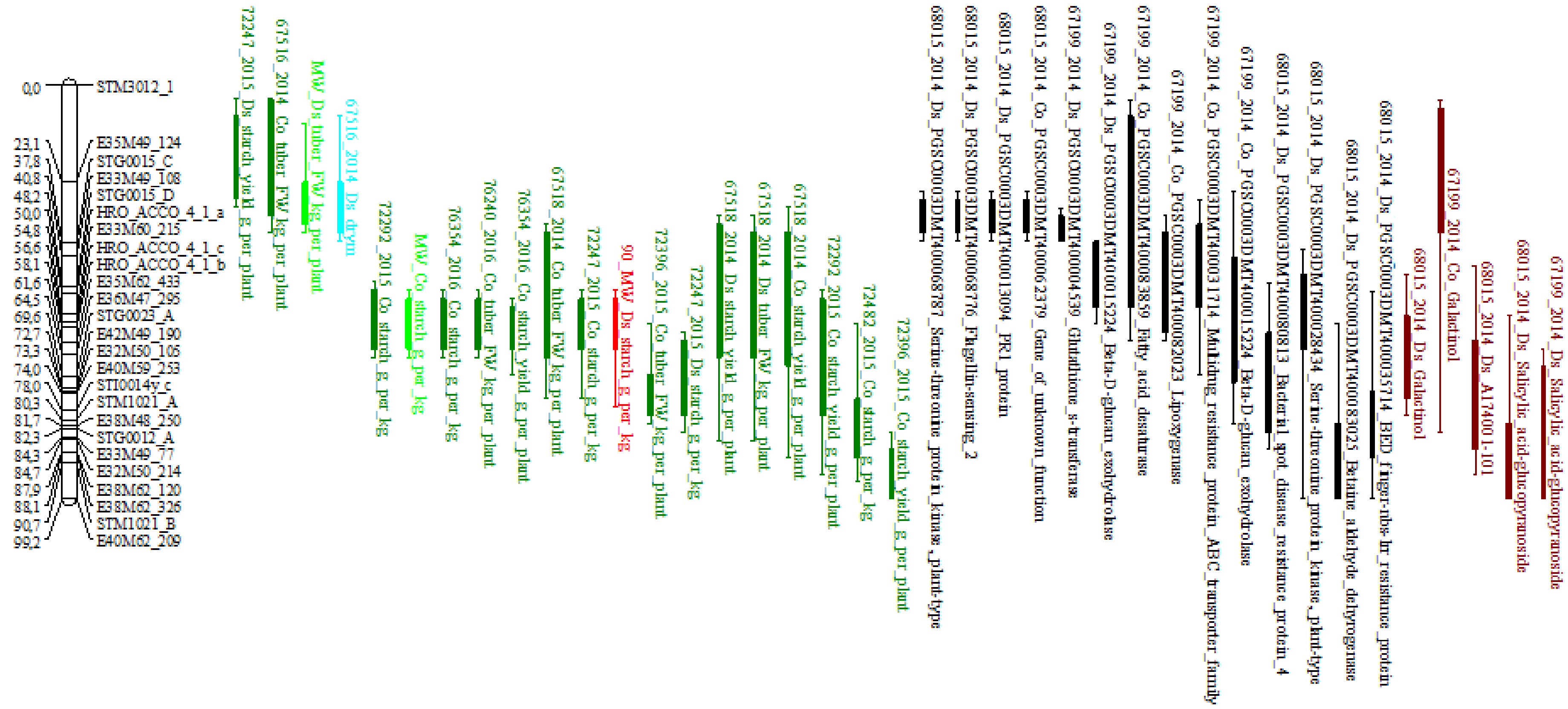

0,0 STM3012_1
23,1 E35M49_124
37,8 STG0015_C
40,8 E33M49_108
48,2 STG0015_D
50,0 HRO_ACCO_4_1_a
54,8 E33M60_215
56,6 HRO_ACCO_4_1_c
58,1 HRO_ACCO_4_1_b
61,6 E35M62_433
64,5 E36M47_295
69,6 STG0025_A
72,7 E42M49_190
73,3 E32M50_105
74,0 E40M59_253
78,0 STI0014y_c
80,3 STM1021_A
81,7 E38M48_250
82,3 STG0012_A
84,3 E33M49_77
84,7 E32M50_214
87,9 E38M62_120
88,1 E38M62_326
90,7 STM1021_B
99,2 E40M62_209
72247_2015_Ds_starch_yield_g_per_plant
67516_2014_Co_tuber_FW_kg_per_plant
MW_Ds_tuber_FW_kg_per_plant
67516_2014_Ds_drym
72292_2015_Co_starch_g_per_kg
MW_Co_starch_g_per_kg
76354_2016_Co_starch_g_per_kg
76240_2016_Co_tuber_FW_kg_per_plant
76354_2016_Co_starch_yield_g_per_plant
67518_2014_Co_tuber_FW_kg_per_plant
72247_2015_Co_starch_g_per_kg
90_MW_Ds_starch_g_per_kg
72396_2015_Co_tuber_FW_kg_per_plant
72247_2015_Ds_starch_g_per_kg
67518_2014_Ds_starch_yield_g_per_plant
67518_2014_Ds_tuber_FW_kg_per_plant
67518_2014_Co_starch_yield_g_per_plant
72292_2015_Co_starch_yield_g_per_plant
72482_2015_Co_starch_g_per_kg
72396_2015_Co_starch_yield_g_per_plant
68015_2014_Ds_PGSC0003DMT400068787_Serine-threonine_protein_kinase_plant-type
68015_2014_Ds_PGSC0003DMT400068776_Flagellin-sensing_2
68015_2014_Ds_PGSC0003DMT400013094_PR1_protein
68015_2014_Co_PGSC0003DMT400062379_Gene_of_unknown_function
67199_2014_Ds_PGSC0003DMT400004539_Glutathione_s-transferase
67199_2014_Ds_PGSC0003DMT400015224_Beta-D-glucan_exohydrolase
67199_2014_Co_PGSC0003DMT400083859_Fatty_acid_desaturase
67199_2014_Co_PGSC0003DMT400082023_Lipoxygenase
67199_2014_Co_PGSC0003DMT400031714_Multidrug_resistance_protein_ABC_transporter_family
67199_2014_Co_PGSC0003DMT400015224_Beta-D-glucan_exohydrolase
68015_2014_Ds_PGSC0003DMT400080813_Bacterial_spot_disease_resistance_protein_4
68015_2014_Ds_PGSC0003DMT400028434_Serine-threonine_protein_kinase_plant-type
68015_2014_Ds_PGSC0003DMT400083025_Betaine_aldehyde_dehydrogenase
68015_2014_Ds_PGSC0003DMT400035714_BED_finger-nbs-lrr_resistance_protein
67199_2014_Co_Galactinol
68015_2014_Ds_Galactinol
68015_2014_Ds_A174001-101
68015_2014_Ds_Salicylic_acid-glucopyranoside
67199_2014_Ds_Salicylic_acid-glucopyranoside

LG9 R

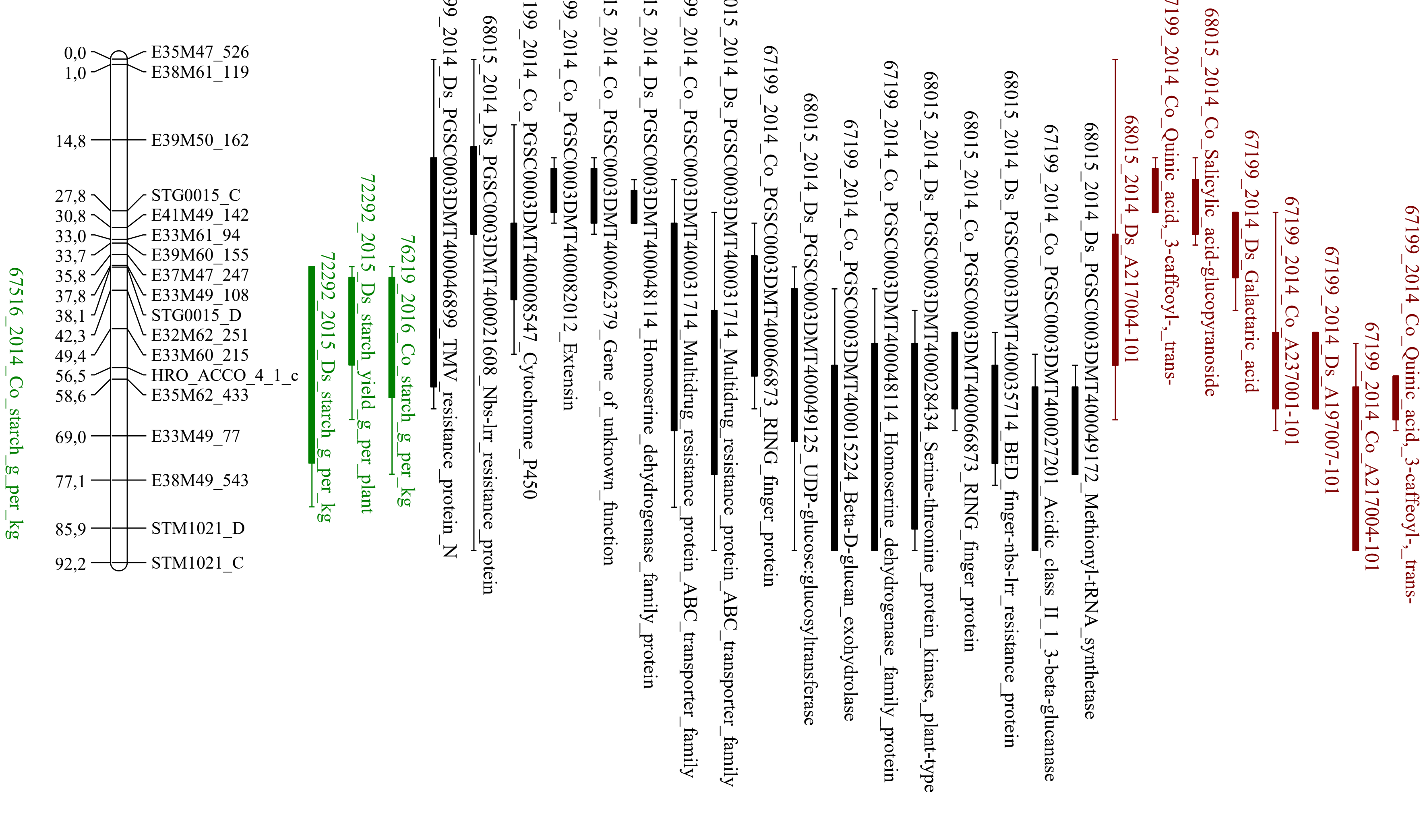

0,0 E35M47_526
1,0 E38M61_119
14,8 E39M50_162
27,8 STG0015_C
30,8 E41M49_142
33,0 E33M61_94
33,7 E39M60_155
35,8 E37M47_247
37,8 E33M49_108
38,1 STG0015_D
42,3 E32M62_251
49,4 E33M60_215
56,5 HRO_ACCO_4_1_c
58,6 E35M62_433
69,0 E33M49_77
77,1 E38M49_543
85,9 STM1021_D
92,2 STM1021_C
67516_2014_Co_starch_g_per_kg
72292_2015_Ds_starch_g_per_kg
72292_2015_Ds_starch_yield_g_per_plant
76219_2016_Co_starch_g_per_kg
67199_2014_Ds_PGSC0003DMT400046899_TMV_resistance_protein_N
68015_2014_Ds_PGSC0003DMT400021608_Nbs-lrr_resistance_protein
67199_2014_Co_PGSC0003DMT400008547_Cytochrome_P450
67199_2014_Co_PGSC0003DMT400082012_Extensin
68015_2014_Co_PGSC0003DMT400062379_Gene_of_unknown_function
68015_2014_Ds_PGSC0003DMT400048114_Homoserine_dehydrogenase_family_protein
67199_2014_Co_PGSC0003DMT400031714_Multidrug_resistance_protein_ABC_transporter_family
68015_2014_Ds_PGSC0003DMT400031714_Multidrug_resistance_protein_ABC_transporter_family
67199_2014_Co_PGSC0003DMT400066873_RING_finger_protein
68015_2014_Ds_PGSC0003DMT400049125_UDP-glucose:glucosyltransferase
67199_2014_Co_PGSC0003DMT400015224_Beta-D-glucan_exohydrolase
67199_2014_Co_PGSC0003DMT400048114_Homoserine_dehydrogenase_family_protein
68015_2014_Ds_PGSC0003DMT400028434_Serine-threonine_protein_kinase_plant-type
68015_2014_Co_PGSC0003DMT400066873_RING_finger_protein
68015_2014_Ds_PGSC0003DMT400035714_BED_finger-nbs-lrr_resistance_protein
67199_2014_Co_PGSC0003DMT400027201_Acidic_class_II_1_3-beta-glucanase
68015_2014_Ds_PGSC0003DMT400049172_Methionyl-tRNA_synthetase
68015_2014_Ds_A217004-101
67199_2014_Co_Quinic_acid,_3-caffeoyl-,_trans-
68015_2014_Co_Salicylic_acid-glucopyranoside
67199_2014_Ds_Galactaric_acid
67199_2014_Co_A237001-101
67199_2014_Ds_A197007-101
67199_2014_Co_A217004-101
67199_2014_Co_Quinic_acid,_3-caffeoyl-,_trans-

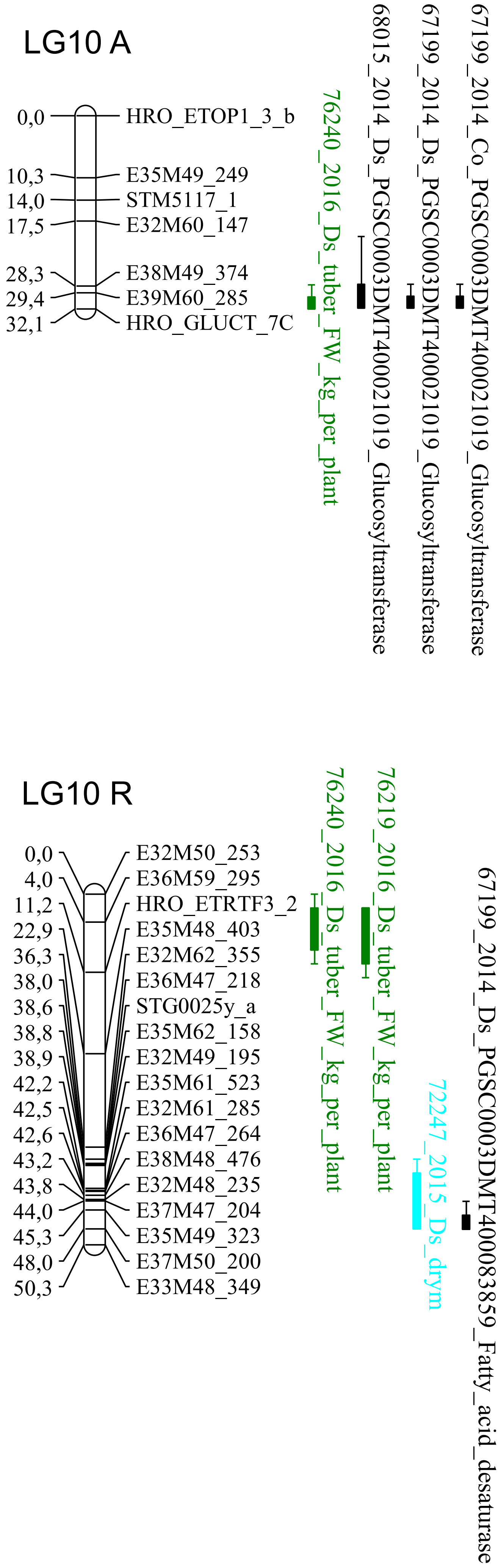
LG10 A
0,0 HRO_ETOP1_3_b
10,3 E35M49_249
14,0 STM5117_1
17,5 E32M60_147
28,3 E38M49_374
29,4 E39M60_285
32,1 HRO_GLUCT_7C
76240_2016_Ds_tuber_FW_kg_per_plant
68015_2014_Ds_PGSC0003DMT400021019_Glucosyltransferase
67199_2014_Ds_PGSC0003DMT400021019_Glucosyltransferase
67199_2014_Co_PGSC0003DMT400021019_Glucosyltransferase
LG10 R
0,0 E32M50_253
4,0 E36M59_295
11,2 HRO_ETRTF3_2
22,9 E35M48_403
36,3 E32M62_355
38,0 E36M47_218
38,6 STG0025y_a
38,8 E35M62_158
38,9 E32M49_195
42,2 E35M61_523
42,5 E32M61_285
42,6 E36M47_264
43,2 E38M48_476
43,8 E32M48_235
44,0 E37M47_204
45,3 E35M49_323
48,0 E37M50_200
50,3 E33M48_349
76240_2016_Ds_tuber_FW_kg_per_plant
76219_2016_Ds_tuber_FW_kg_per_plant
72247_2015_Ds_drym
67199_2014_Ds_PGSC0003DMT400083859_Fatty_acid_desaturase

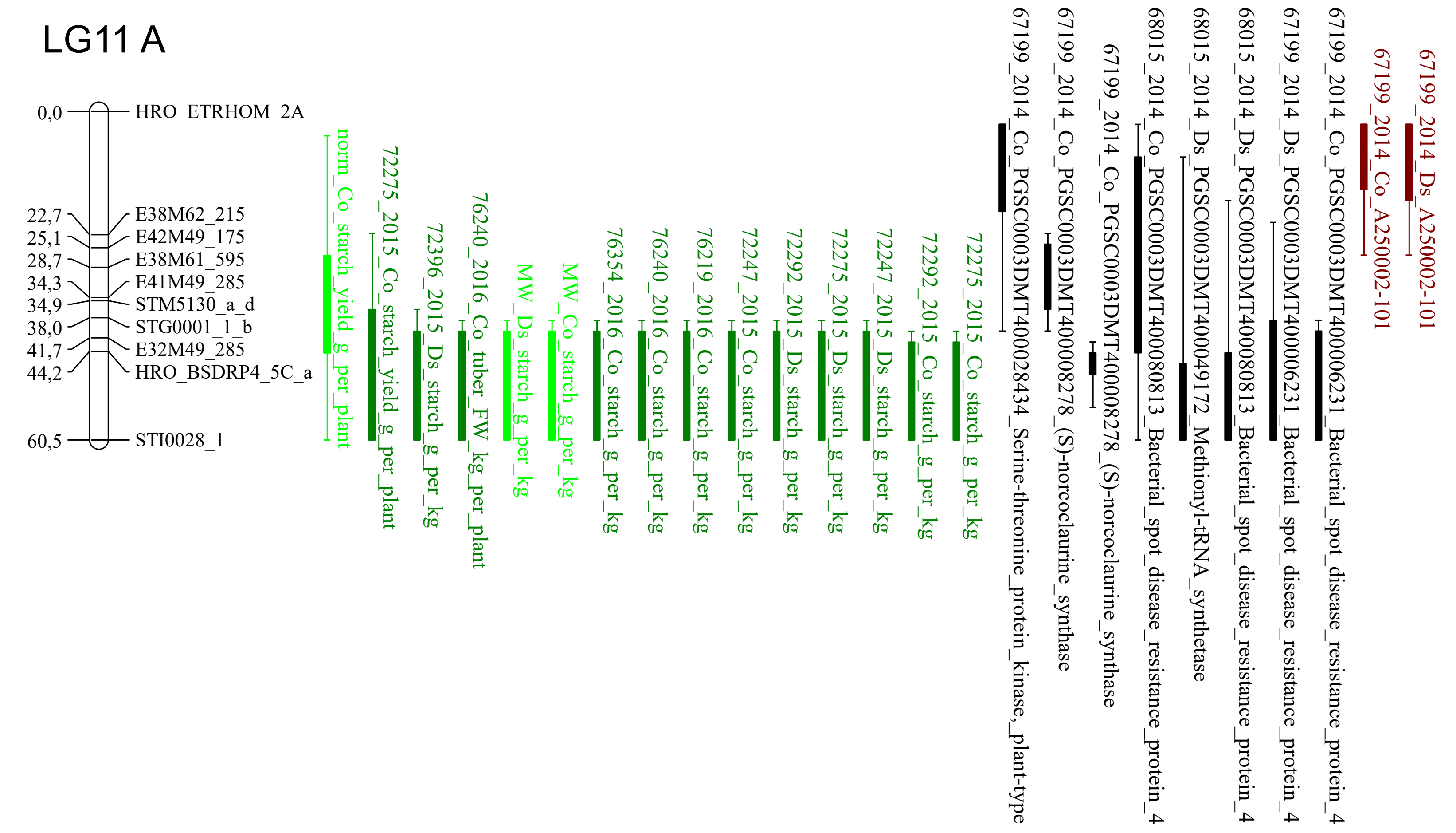
LG11 A
0,0 HRO_ETRHOM_2A
22,7 E38M62_215
25,1 E42M49_175
28,7 E38M61_595
34,3 E41M49_285
34,9 STM5130_a_d
38,0 STG0001_1_b
41,7 E32M49_285
44,2 HRO_BSDRP4_5C_a
60,5 STI0028_1
norm_Co_starch_yield_g_per_plant
72275_2015_Co_starch_yield_g_per_plant
72396_2015_Ds_starch_g_per_kg
76240_2016_Co_tuber_FW_kg_per_plant
MW_Ds_starch_g_per_kg
MW_Co_starch_g_per_kg
76354_2016_Co_starch_g_per_kg
76240_2016_Co_starch_g_per_kg
76219_2016_Co_starch_g_per_kg
72247_2015_Co_starch_g_per_kg
72292_2015_Ds_starch_g_per_kg
72275_2015_Ds_starch_g_per_kg
72247_2015_Ds_starch_g_per_kg
72292_2015_Co_starch_g_per_kg
72275_2015_Co_starch_g_per_kg
67199_2014_Co_PGSC0003DMT400028434_Serine-threonine_protein_kinase,_plant-type
67199_2014_Co_PGSC0003DMT400008278_(S)-norcoclaurine_synthase
67199_2014_Co_PGSC0003DMT400008278_(S)-norcoclaurine_synthase
68015_2014_Co_PGSC0003DMT400080813_Bacterial_spot_disease_resistance_protein_4
68015_2014_Ds_PGSC0003DMT400049172_Methionyl-tRNA_synthetase
68015_2014_Ds_PGSC0003DMT400080813_Bacterial_spot_disease_resistance_protein_4
67199_2014_Ds_PGSC0003DMT400006231_Bacterial_spot_disease_resistance_protein_4
67199_2014_Co_PGSC0003DMT400006231_Bacterial_spot_disease_resistance_protein_4
67199_2014_Co_A250002-101
67199_2014_Ds_A250002-101

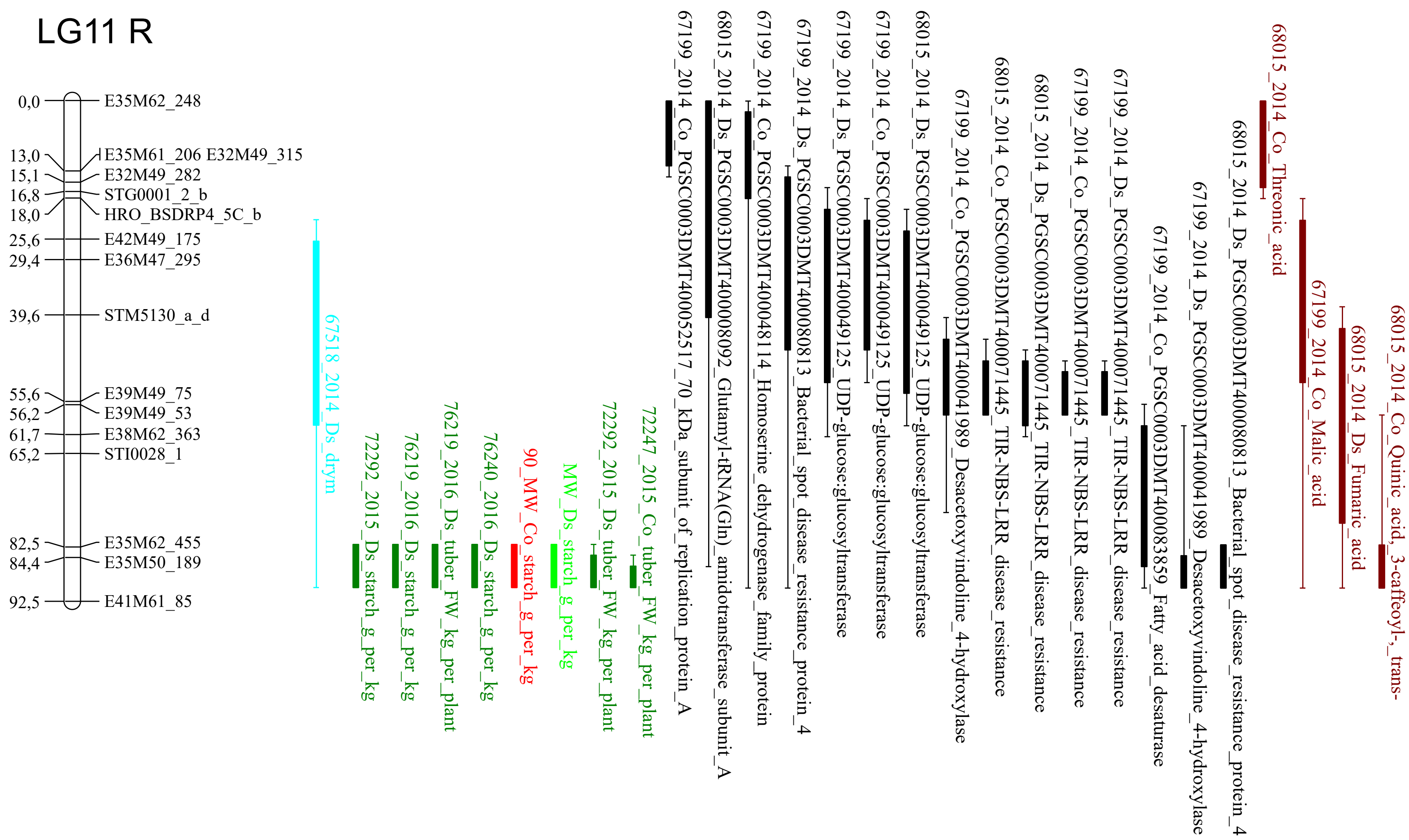
LG11 R
0,0 E35M62_248
13,0 E35M61_206 E32M49_315
15,1 E32M49_282
16,8 STG0001_2_b
18,0 HRO_BSDRP4_5C_b
25,6 E42M49_175
29,4 E36M47_295
39,6 STM5130_a_d
55,6 E39M49_75
56,2 E39M49_53
61,7 E38M62_363
65,2 STI0028_1
82,5 E35M62_455
84,4 E35M50_189
92,5 E41M61_85
67518_2014_Ds_drym
72292_2015_Ds_starch_g_per_kg
76219_2016_Ds_starch_g_per_kg
76219_2016_Ds_tuber_FW_kg_per_plant
76240_2016_Ds_starch_g_per_kg
90_MW_Co_starch_g_per_kg
MW_Ds_starch_g_per_kg
72292_2015_Ds_tuber_FW_kg_per_plant
72247_2015_Co_tuber_FW_kg_per_plant
67199_2014_Co_PGSC0003DMT400052517_70_kDa_subunit_of_replication_protein_A
68015_2014_Ds_PGSC0003DMT400008092_Glutamyl-tRNA(Gln)_amidotransferase_subunit_A
67199_2014_Co_PGSC0003DMT400048114_Homoserine_dehydrogenase_family_protein
67199_2014_Ds_PGSC0003DMT400080813_Bacterial_spot_disease_resistance_protein_4
67199_2014_Ds_PGSC0003DMT400049125_UDP-glucose:glucosyltransferase
67199_2014_Co_PGSC0003DMT400049125_UDP-glucose:glucosyltransferase
68015_2014_Ds_PGSC0003DMT400049125_UDP-glucose:glucosyltransferase
67199_2014_Co_PGSC0003DMT400041989_Desacetoxyvindoline_4-hydroxylase
68015_2014_Co_PGSC0003DMT400071445_TIR-NBS-LRR_disease_resistance
68015_2014_Ds_PGSC0003DMT400071445_TIR-NBS-LRR_disease_resistance
67199_2014_Co_PGSC0003DMT400071445_TIR-NBS-LRR_disease_resistance
67199_2014_Ds_PGSC0003DMT400071445_TIR-NBS-LRR_disease_resistance
67199_2014_Co_PGSC0003DMT400083859_Fatty_acid_desaturase
67199_2014_Ds_PGSC0003DMT400041989_Desacetoxyvindoline_4-hydroxylase
68015_2014_Ds_PGSC0003DMT400080813_Bacterial_spot_disease_resistance_protein_4
68015_2014_Co_Threonic_acid
67199_2014_Co_Malic_acid
68015_2014_Ds_Fumaric_acid
68015_2014_Co_Quinic_acid,_3-caffeoyl-,_trans-

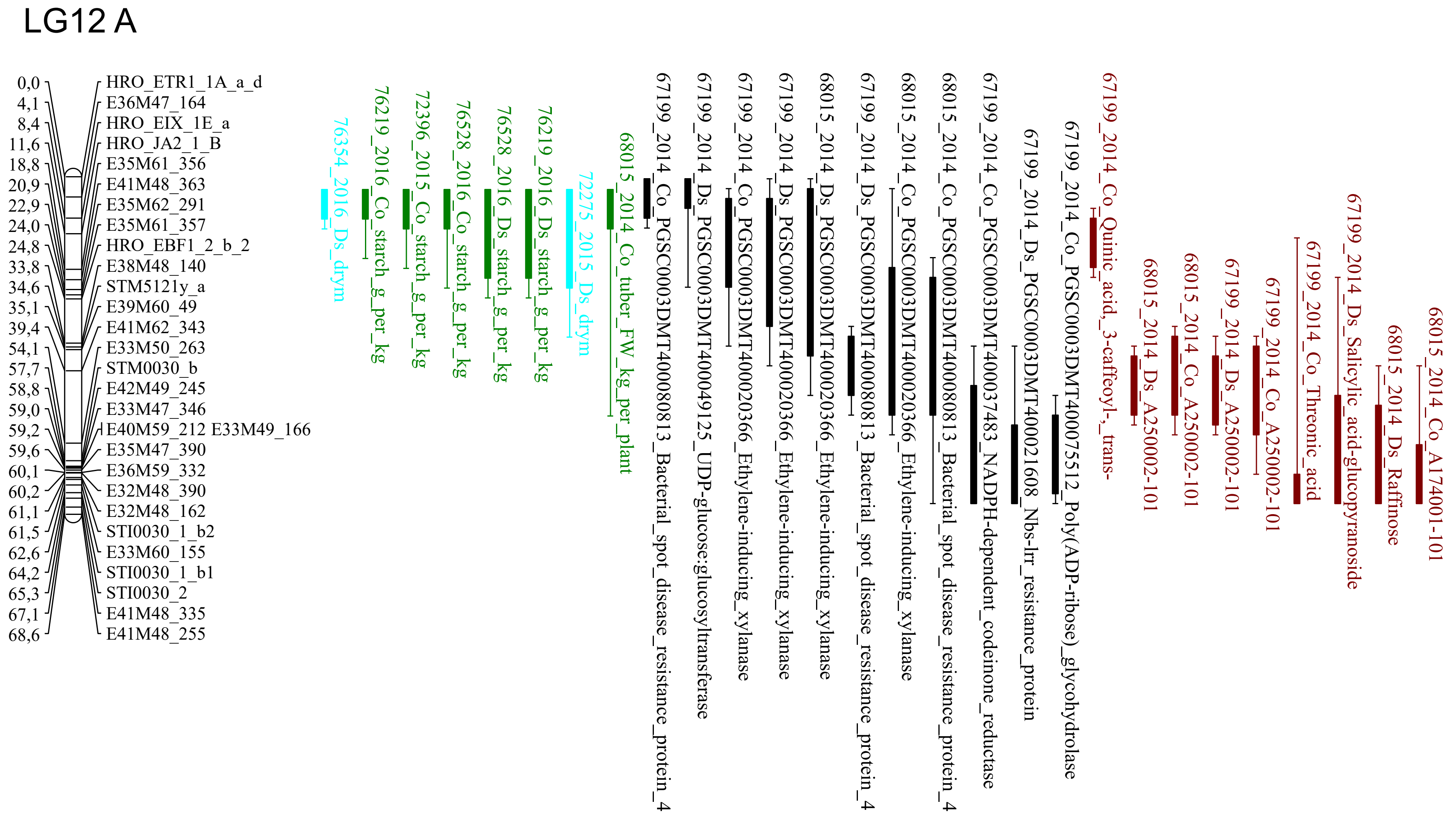
LG12 A
0,0 HRO_ETR1_1A_a_d
4,1 E36M47_164
8,4 HRO_EIX_1E_a
11,6 HRO_JA2_1_B
18,8 E35M61_356
20,9 E41M48_363
22,9 E35M62_291
24,0 E35M61_357
24,8 HRO_EBF1_2_b_2
33,8 E38M48_140
34,6 STM5121y_a
35,1 E39M60_49
39,4 E41M62_343
54,1 E33M50_263
57,7 STM0030_b
58,8 E42M49_245
59,0 E33M47_346
59,2 E40M59_212 E33M49_166
59,6 E35M47_390
60,1 E36M59_332
60,2 E32M48_390
61,1 E32M48_162
61,5 STI0030_1_b2
62,6 E33M60_155
64,2 STI0030_1_b1
65,3 STI0030_2
67,1 E41M48_335
68,6 E41M48_255
76354_2016_Ds_drym
76219_2016_Co_starch_g_per_kg
72396_2015_Co_starch_g_per_kg
76528_2016_Co_starch_g_per_kg
76528_2016_Ds_starch_g_per_kg
76219_2016_Ds_starch_g_per_kg
72275_2015_Ds_drym
68015_2014_Co_tuber_FW_kg_per_plant
67199_2014_Co_PGSC0003DMT400080813_Bacterial_spot_disease_resistance_protein_4
67199_2014_Ds_PGSC0003DMT400049125_UDP-glucose:glucosyltransferase
67199_2014_Co_PGSC0003DMT400020366_Ethylene-inducing_xylanase
67199_2014_Ds_PGSC0003DMT400020366_Ethylene-inducing_xylanase
68015_2014_Ds_PGSC0003DMT400020366_Ethylene-inducing_xylanase
67199_2014_Ds_PGSC0003DMT400080813_Bacterial_spot_disease_resistance_protein_4
68015_2014_Co_PGSC0003DMT400020366_Ethylene-inducing_xylanase
68015_2014_Co_PGSC0003DMT400080813_Bacterial_spot_disease_resistance_protein_4
67199_2014_Co_PGSC0003DMT400037483_NADPH-dependent_codeinone_reductase
67199_2014_Ds_PGSC0003DMT400021608_Nbs-lrr_resistance_protein
67199_2014_Co_PGSC0003DMT400075512_Poly(ADP-ribose)_glycohydrolase
67199_2014_Co_Quinic_acid,_3-caffeoyl-,_trans-
68015_2014_Ds_A250002-101
68015_2014_Co_A250002-101
67199_2014_Ds_A250002-101
67199_2014_Co_A250002-101
67199_2014_Co_Threonic_acid
67199_2014_Ds_Salicylic_acid-glucopyranoside
68015_2014_Ds_Raffinose
68015_2014_Co_A174001-101

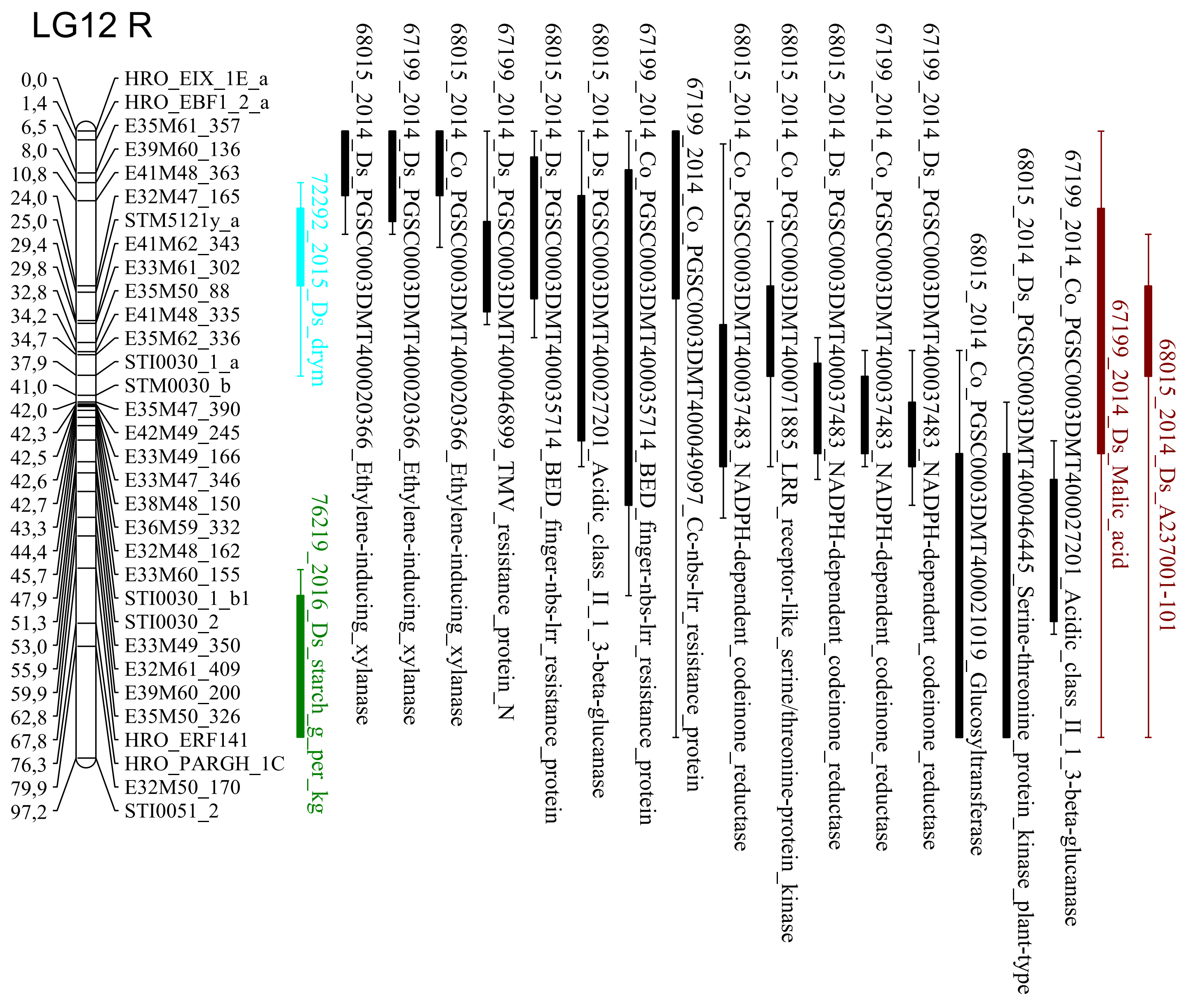
LG12 R
0,0 HRO_EIX_1E_a
1,4 HRO_EBF1_2_a
6,5 E35M61_357
8,0 E39M60_136
10,8 E41M48_363
24,0 E32M47_165
25,0 STM5121y_a
29,4 E41M62_343
29,8 E33M61_302
32,8 E35M50_88
34,2 E41M48_335
34,7 E35M62_336
37,9 STI0030_1_a
41,0 STM0030_b
42,0 E35M47_390
42,3 E42M49_245
42,5 E33M49_166
42,6 E33M47_346
42,7 E38M48_150
43,3 E36M59_332
44,4 E32M48_162
45,7 E33M60_155
47,9 STI0030_1_b1
51,3 STI0030_2
53,0 E33M49_350
55,9 E32M61_409
59,9 E39M60_200
62,8 E35M50_326
67,8 HRO_ERF141
76,3 HRO_PARGH_1C
79,9 E32M50_170
97,2 STI0051_2
72292_2015_Ds_drym
76219_2016_Ds_starch_g_per_kg
68015_2014_Ds_PGSC0003DMT400020366_Ethylene-inducing_xylanase
67199_2014_Ds_PGSC0003DMT400020366_Ethylene-inducing_xylanase
68015_2014_Co_PGSC0003DMT400020366_Ethylene-inducing_xylanase
67199_2014_Ds_PGSC0003DMT400046899_TMV_resistance_protein_N
68015_2014_Ds_PGSC0003DMT400035714_BED_finger-nbs-lrr_resistance_protein
68015_2014_Ds_PGSC0003DMT400027201_Acidic_class_II_1,3-beta-glucanase
67199_2014_Co_PGSC0003DMT400035714_BED_finger-nbs-lrr_resistance_protein
67199_2014_Co_PGSC0003DMT400049097_Cc-nbs-lrr_resistance_protein
68015_2014_Co_PGSC0003DMT400037483_NADPH-dependent_codeinone_reductase
68015_2014_Co_PGSC0003DMT400071885_LRR_receptor-like_serine/threonine-protein_kinase
68015_2014_Ds_PGSC0003DMT400037483_NADPH-dependent_codeinone_reductase
67199_2014_Co_PGSC0003DMT400037483_NADPH-dependent_codeinone_reductase
67199_2014_Ds_PGSC0003DMT400037483_NADPH-dependent_codeinone_reductase
68015_2014_Co_PGSC0003DMT400021019_Glucosyltransferase
68015_2014_Ds_PGSC0003DMT400046645_Serine-threonine_protein_kinase_plant-type
67199_2014_Co_PGSC0003DMT400027201_Acidic_class_II_1,3-beta-glucanase
67199_2014_Ds_Malic_acid
68015_2014_Ds_A237001-101

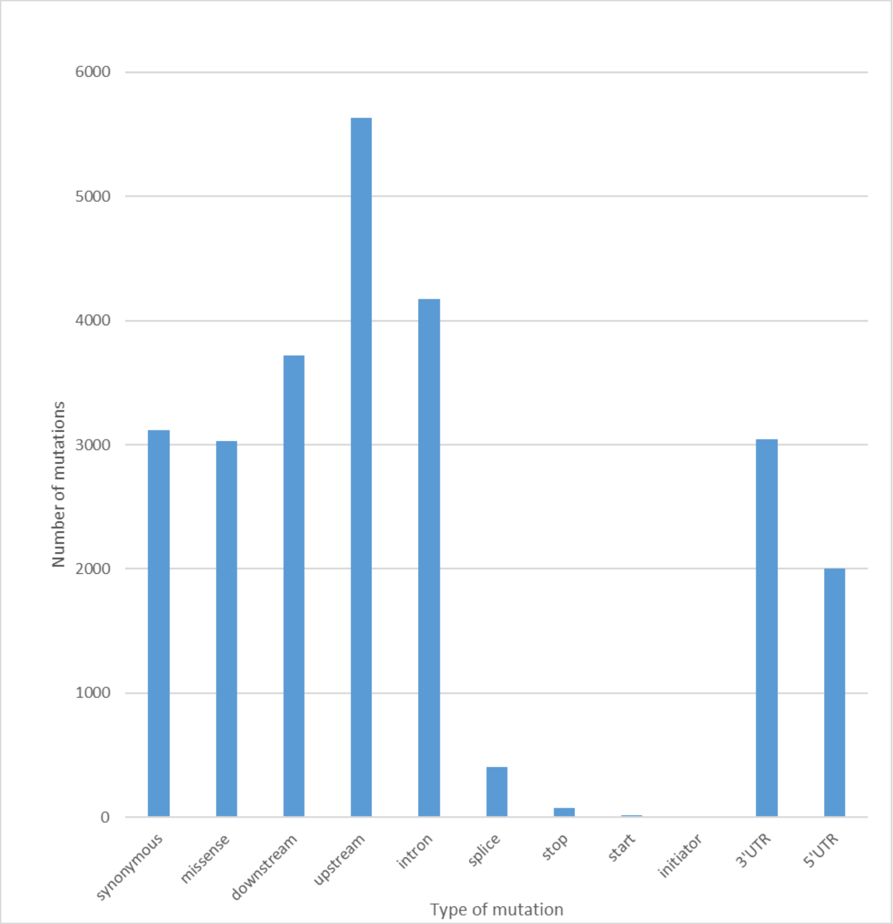

Figure S7: Overview over the different types of mutations occurring between the drought tolerant and drought sensitive bulk at the DRYM QTL on LG3.

Table S1: Linkage group specific SSR markers (reviewed in Ghislain *et al.* [33]).

SSR Primer	Candidate gene Ensemble Plants	Alternative name	LG	EnsemblPlants	Location (EnsemblPlants)	Primary transcript	Primer forward	Primer reverse
STG0001	NtRab11D	Ras-related protein Rab11D-like	**11**	PGSC0003DMG400016224	11: 2,745,709-2,748,048	PGSC0003DMT400041813	CAgCCAACATTTgTACCCCT	ACCCCCACTTgCCATATTTT
STG0010	Conserved gene of unknown function		**3**	PGSC0003DMG400022897	3: 61,403,444-61,405,401	PGSC0003DMT400058939	CgATCTCTgCTTTgCAggTA	gTTCATCACTACCgCCgACT
STG0012	MAR-binding protein	probable nucleolar protein 5-2	**9**	PGSC0003DMG400006104	9: 55,116,198-55,120,131	PGSC0003DMT400015638	ACACgCTgATgTTgAggATg	CTTAAAAACACgCCACCCAT
STG0015	RNA binding protein	putative G3BP-like protein	**9**	PGSC0003DMG400027944	9: 31,218,417-31,224,429	PGSC0003DMT400071822	TgTAgCTgAAATCCgTggTg	ggCgAggAAACTACAATggA
STG0016	Chromo domain protein LHP1	Chromo domain protein LHP1	**1**	PGSC0003DMG400031112	1: 67,227,146-67,233,452	PGSC0003DMT400079885	AgCTgCTCAgCATCAAgAgA	ACCACCTCAggCACTTCATC
STG0018	Glutamine-rich protein	bromodomain-containing protein DDB_G0280777	**3**	PGSC0003DMG400026490	3: 46,403,236-46,410,127	PGSC0003DMT400068108	ACCCgAATCCAAACCCTAAC	AACCCgTgTCAACTTCTgCT
STG0020	Sodium/potassium/calcium exchanger	glutamic acid-rich protein-like	**4**	PGSC0003DMG400006339	4: 7,257,001-7,261,389	PGSC0003DMT400016219	gTCATCCgAAgATggAggAA	CTggACTgTCTTCACCACCA
STG0021	Cornichon family protein	protein cornichon homolog 4-like	**5**	PGSC0003DMG400007015	5: 45,581,656-45,587,896	PGSC0003DMT400018058	TgCCTACTgCCCAAAACATT	ACTggCTgggAAgCATACAC
STG0023	Binding protein	uncharacterised	**9**	PGSC0003DMG400031841	9: 53,064,362-53,065,293	PGSC0003DMT400081442	CTgCTAAAATTggACCCgAg	gCAAgATTCgCCATCATTTT
STG0025	Oxidoreductase/ transition metal ion binding protein		**10**	PGSC0003DMG400028767	10: 33,537,359-33,540,698	PGSC0003DMT400074007	TggAATCCgAATTACgCTCT	AggTTTTACCACTCgggCTT
STGBSS2	Granule-bound starch synthase 1 (GBSS)	WAXY (SSG1_SOLTU)	**8**	Q00775	8: 56,782,186-56,785,790		AATCggTgATAAATgTgAATgC	ATgCTTgCCATgTgATgTgT
STI0001	Tuber-specific and sucrose-responsive element binding factor	transcription factor MYB44-like	**4**	PGSC0003DMG400007994	4: 68,721,298-68,722,349	PGSC0003DMT400020635	CAgCAAAATCAgAACCCgAT	ggATCATCAAATTCACCgCT
STI0003	Methionine rich arabinogalactan	arabinogalactan protein 9	**8**	PGSC0003DMG400026224	8: 51,167,796-51,168,630	PGSC0003DMT400067461	ACCATCCACCATgTCAATgC	CTCATggATggTgTCATTgg
STI0004	Nucleic acid binding protein	protein indeterminate-domain 9-like	**6**	PGSC0003DMG400003372	6: 55,860,945-55,863,589	PGSC0003DMT400008694	GCTgCTAAACACTCAAgCAgAA	CAACTACAAgATTCCATCCACAg
STI0012	cold-stress inducible protein (C17)		**4**	U69633.1			gAAgCgACTTCCAAAATCAgA	AAAgggAggAATAgAAACCAAAA
STI0014	Auxin responsive protein	auxin-responsive protein IAA14-like	**9**	PGSC0003DMG400006093	9: 55,351,420-55,354,606	PGSC0003DMT400015610	AgAAACTgAgTTgTgTTTgggA	TCAACAgTCTCAgAAAACCCTCT
STI0021	Heat stress transcription factor A-6b	heat stress transcription factor A-6b-like	**6**	PGSC0003DMG400016270	6: 40,218,132-40,220,250	PGSC0003DMT400041944	TCATCAAGTCGTCGTCATCAA	TAGAATGATCCAAAGCTTCC
STI0024	Hydroxyproline-rich glycoprotein family protein	nuclear pore complex protein NUP58	**2**	PGSC0003DMG400010074	2: 44,529,204-44,537,572	PGSC0003DMT400026136	CgCCATTCTCTCAgATCACTC	gCTgCAgCAgTTgTTgTTgT
STI0025	afadin- and alpha-actinin-binding protein-like		**7**	XM_006361516.2			CTgCCgCAAAAAgTgAAAAC	TgAATgTAggCCAAATTTTgAA
STI0028	Conserved gene of unknown function		**11**	PGSC0003DMG400007365	11: 37,968,820-37,970,009	PGSC0003DMT400019013	ATACCCTCCAATgggTCCTT	CTTggAgATttgCAAgAAgAA
STI0030	Conserved gene of unknown function	probable beta-1,4-xylosyltransferase IRX10L	**12**	PGSC0003DMG400014472	12: 49,058,583-49,067,549	PGSC0003DMT400037513	TTgACCCTCCAACTATAgATTCTTC	TgACAACTTTAAAgCATATgTCAgC
STI0032	Membrane associated ring finger 1,8	uncharacterized protein LOC102582422	**5**	PGSC0003DMG400018414	5: 4,786,918-4,792,370	PGSC0003DMT400047386	TgggAAgAATCCTgAAATgg	TgCTCTACCAATTAACggCA
STI0033	Heat stress transcription factor HSFA9	heat shock factor protein HSF30-like	**7**	PGSC0003DMG400032793	7: 36,269,190-36,271,391	PGSC0003DMT400082752	TgAgggTTTTCAgAAAgggA	CATCCTTgCAACAACCTCCT
STI0034	Fasciclin-like arabinogalactan protein 14	fasciclin-like arabinogalactan protein 17	**1**	PGSC0003DMG400021372		PGSC0003DMT400055078	CAAgAAACCAAgAgCAAATTTCA	TggCgAATgTgAgAAACAAA
STI0036	Transcriptional regulator family protein	uncharacterised protein	**2**	PGSC0003DMG400028477	2: 31,847,395-31,855,085		ggACTggCTgACCATgAACT	TTACAggAAATgCAAACTTCg
STI0043	Zinc finger protein	protein indeterminate-domain 5, chloroplastic-like	**1**	PGSC0003DMG400016379	1: 3,502,584-3,504,030	PGSC0003DMT400042209	CAATgCgAATgTTgCTACTggT	ATCCACCAAgACCTCCAgAA
STI0051	Dead box ATP-dependent RNA helicase		**12**	PGSC0003DMG400004585	12: 59,520,252-59,526,095	PGSC0003DMT400011665	ggTCTCCATTAgCCCTCTgAg	ACATAAATggATCACACA
STI0056	Acyl-CoA thioesterase	1,4-dihydroxy-2-naphthoyl-CoA thioesterase 1	**2**	PGSC0003DMG400022385	2: 34,101,866-34,103,039	PGSC0003DMT400057647	gACAgAgAATATgggACCACCA	gCAgCACCTTAAATggCTgAC
STM0030	not annotated		**12**	-	12: 22,813,884-22,813,902		AgAgATCgATgTAAAACACgT	gTggCATTTTgATggATT
STM0031	not annotated		**7**	-	7: 17,864,488-17,864,661		CATACgCACgCACgTACAC	TTCAACCTATCATTTTgTgAgTCg
STM1004	1-Aminocyclopropane-1-carboxylate synthase (ACS2)	1-aminocyclopropane-1-carboxylate synthase 4-like	**7**	PGSC0003DMG400017039	7: 25,011,892-25,013,488	PGSC0003DMT400043903	ATATgAAATTCTCTCgATgTTTCg	TCAgCCCATAAAXCTTTAgTTACCT
STM1021	proteinase inhibitor 1-like (PPI3), serine protease inhibitor (PIN)		**9**	XM_006367721.2			ggAgTCAAAgTTTgCTCACATC	CACCCTCAACCCCCATATC
STM1031	Actin-58		**5**	PGSC0003DMG400023429	5: 50,245,290-50,248,122	PGSC0003DMT400060225	TgTgTTTgTTTTTCTgTAT	AATTCTATCCTCATCTCTA
STM1058	not annotated		**3**	-	3: 49,869,169-49,869,195		ACAATTTAATTCAAgAAgCTAgg	CCAAATTTgTATACTTCAATATgA
STM1104	granule-bound starch synthase (GBSS) (WAXY)		**8**	PGSC0003DMG400012111	8: 56,782,186-56,785,790		TgATTCTCTTgCCTACTgTAATCg	CAAAgTggTgTgAAgCTgTgA
STM3012	not annotated		**9**	-	9: 3,964,718-3,964,881		CAACTCAAACCAgAAggCAAA	gAgAAATgggCACAAAAAACA
STM5114	Disease resistance response protein		**2**	PGSC0003DMG403001521	2: 38,554,309-38,555,180	PGSC0003DMT400003860	AATggCTCTCTCTgTATgCT	gCTgTCCCAACTATCTTTgA
STM5115	Glycerol kinase		**3**	PGSC0003DMG400014144	3: 57,209,534-57,212,556		TCTCCCCCTTCTATCCTCTT	TCAACCTggTgAgATCCAACA
STM5117	U2 snrnp auxiliary factor, small subunit	splicing factor U2af small subunit B-like	**10**	PGSC0003DMG400028265	10: 55,512,395-55,515,132		TCCACATTAgAAAACCCCTC	AggACgCTggTACATgTTAgA
STM5121	Conserved gene of unknown function		**12**	PGSC0003DMG400000292	12: 4,002,106-4,002,649		CACCggAATAAgCggATCT	TCTTCCCTTCCATTTgTCA
STM5126	uncharacterised		**6**	XP_006354728.1			gCAACAgCgCATCAACAAA	TCCAAATCCATCCCATTgAg
STM5130	U11/U12 small nuclear ribonucleoprotein		**11**	PGSC0003DMG400031069	11: 3,780,507-3,783,394		AAAgTACAgCgAAgATgACgAC	TTACCTTTgCAACCTTgCC
STM5149	not annotated		**5**	-	5: 51,904,687-51,904,711		CTCCCAAACTAACCCTAATTAC	gCgACTCTATTAATCACTTgAC

Table S2: SSR markers derived from candidate genes for drought tolerance by Schumacher *et al.* [34] and mapped in A×R.

Candidate gene	Abbr.	SSR primer	LG	Gene ID EnsemblPlants	Location (EnsemblPlants)	Primary transcript
ABA8'-hydroxylase CYP707A2	ABA8H	HRO_ABA8H4_2	4	PGSC0003DMG400007972	4: 68,244,330-68,248,224	PGSC0003 DMT400020589
1-aminocyclopropane 1-carboxylate oxidase	ACO	HRO_ACCO_1	2	PGSC0003DMG400021476	2: 17,740,757-17,743,016	PGSC0003 DMT400055332
1-aminocyclopropane 1-carboxylate oxidase	ACO	HRO_ACCO_4	9	PGSC0003DMG400009719	9: 49,787,843-49,791,601	PGSC0003 DMT400025148
1-aminocyclopropane-1-carboxylate synthase 2	ACS2	HRO_ACCS2_1	1	PGSC0003DMG400000193	1: 72,662,888-72,665,831	PGSC0003 DMT400000553
1-aminocyclopropane 1-carboxylate synthase 3	ACS3	HRO_ACCS3_1	2	PGSC0003DMG400021426	2: 42,416,137-42,418,380	PGSC0003 DMT400055203
1-aminocyclopropane 1-carboxylate synthase 4	ACS4	HRO_ACCS4_1	5	PGSC0003DMG400021651	5: 44,070,623-44,073,744	PGSC0003 DMT400055753
Aldehyde dehydrogenase family 7 member	ALDH	HRO_ALDH_H	4	PGSC0003DMG400034597	9: 22,591,541-22,597,210	PGSC0003 DMT400085026
Beta-amylase	BA	HRO_BA_1	1	PGSC0003DMG400000169	1: 72,104,919-72,114,159	PGSC0003 DMT400000485
Betaine aldehyde dehydrogenase	BADH	HRO_BADH_2	6	PGSC0003DMG400033028	6: 52,130,166-52,135,413	PGSC0003 DMT400083025
Beta-D-glucan exohydrolase	BDGEH	HRO_BDGEH_1B	6	PGSC0003DMG402005942	6: 54,345,177-54,347,242	PGSC0003 DMT400015224
Bacterial spot disease resistance protein 4 (BS4)	BS4	HRO_BSDRP4_5C	11	PGSC0003DMG400033334	11: 37,644,263-37,650,835	PGSC0003 DMT400006231
Delta 1-pyrroline-5-carboxylate synthetase	P5CS	HRO_D1P5CS_1	6	PGSC0003DMG402026767	6: 15,776,837-15,786,259	PGSC0003 DMT400083471
EIN3-binding F-box protein 1	EBF1	HRO_EBF1_2	12	PGSC0003DMG400002914	12: 2,844,416-2,848,113	PGSC0003 DMT400007545
Ethylene insensitive-like 2	EIL2	HRO_EIL2_1	1	PGSC0003DMG400008712	1: 6,142,379-6,146,461	PGSC0003 DMT400022477
Ethylene-inducing xylananse	EIX	HRO_EIX_1E	12	PGSC0003DMG400007876	12: 1,808,690-1,811,450	PGSC0003 DMT400020367
Ethylene responsive element binding protein 1	EREBP1	HRO_EREBP1_1	2	PGSC0003DMG400029713	2: 33,632,854-33,633,640	PGSC0003 DMT400076408
Ethylene responsive element binding protein C2	EREBPC2	HRO_EREBPC2_1	5	PGSC0003DMG400016003	5: 47,308,711-47,310,174	PGSC0003 DMT400041327
Ethylene response factor 114	ERF114/ERF80	HRO_ERF114_1	3	PGSC0003DMG400014240	3: 57,768,986-57,771,196	PGSC0003 DMT400036934
Ethylene response factor 141	ERF141	HRO_ERF141	12	PGSC0003DMG400028853	12: 53,036,109-53,038,939	PGSC0003 DMT400074248
Ethylene overproduction protein 1	ETO1	HRO_ETOP1_3	10	PGSC0003DMG400015898	10: 49,687,923-49,692,818	PGSC0003 DMT400041081
Ethylene receptor 1	ETR1	HRO_ETR1_1A	12	PGSC0003DMG400007843	12: 1,111,718-1,121,848	PGSC0003 DMT400020285
Ethylene receptor homolog	ETR homolog	HRO_ETRHOM_2A	11	PGSC0003DMG400028694	11: 6,479,282-6,486,765	PGSC0003 DMT400073847
Ethylene responsive transcription factor 3	ETRTF3	HRO_ETRTF3_2	10	PGSC0003DMG400014417	10: 1,121,218-1,122,800	PGSC0003 DMT400037359
Glucosyltransferase	GT	HRO_GLUCT_7C	10	PGSC0003DMG400008138	10: 58,444,348-58,445,769	PGSC0003 DMT400021019
Jasmonic acid 2	JA2	HRO_JA2_1	12	PGSC0003DMG400015342	12: 822,922-825,609	PGSC0003 DMT400039670
Late embryogenesis abundant protein 5	LEA5	HRO_LEA5_1	6	PGSC0003DMG400017936	6: 461,908-463,086	PGSC0003 DMT400046207
Lipoxygenase	LOX	HRO_LIPOX_1B	1	PGSC0003DMG400032207	1: 2,151,329-2,152,141	PGSC0003 DMT400082023
LRR receptor-like serine/threonine-protein kinase	LRR-STPK	HRO_LRRSTPK_1C	4	PGSC0003DMG400027968	4: 63,318,594-63,322,913	PGSC0003 DMT400071885
Multidrug resistance protein ABC transporter family	MRP	HRO_MRPATF_3D	8	PGSC0003DMG400012167	8: 55,531,912-55,539,184	PGSC0003 DMT400031714
Nucleoside diphosphate kinase 2	NDPK	HRO_NDPK2_1	6	PGSC0003DMG400026976	6: 52,807,449-52,811,263	PGSC0003 DMT400069352
Poly (ADP-ribose) glycohydrolase	PARG	HRO_PARGH_1C	12	PGSC0003DMG400029361	12: 58,212,212-58,218,855	PGSC0003 DMT400075512
Proline oxidase/ dehydrogenase 1	PRODH/PDH	HRO_POD1_2	2	PGSC0003DMG400010050	2: 45,033,737-45,039,948	PGSC0003 DMT400026049
S-locus-specific glycoprotein S6	SLSG6	HRO_SLSGPS6_5C	2	PGSC0003DMG401013876	2: 35,398,866-35,403,067	PGSC0003 DMT400036023
Vacuolar protein sorting protein	VPS	HRO_VPSP_1B	8	PGSC0003DMG400003501	00: 36,520,614-36,528,235	PGSC0003 DMT400008980

Table S3: Genes located under the standalone DRYM QTL on LG3 flanked by the SSR markers STG0018_b (glutamine-rich protein, PGSC0003DMG400026490) and STM5115_D/E (glycerol kinase, PGSC0003DMG400014144)

Chromosome	Region	ID	name
ST4.03ch03	47424062..47430953	PGSC0003DMG400026490	Glutamine-rich protein
ST4.03ch03	47437840..47440384	PGSC0003DMG400026476	Transporter
ST4.03ch03	47449204..47458885	PGSC0003DMG400026491	Sigma factor
ST4.03ch03	47460195..47461543	PGSC0003DMG400026477	Transparent testa glabra 1
ST4.03ch03	47463695..47467667	PGSC0003DMG400026492	Protein kinase family protein
ST4.03ch03	47467748..47468285	PGSC0003DMG400026498	Gene of unknown function
ST4.03ch03	47487516..47493633	PGSC0003DMG400026478	Transcription regulator
ST4.03ch03	47494514..47503012	PGSC0003DMG400026493	DNA-binding protein smubp-2
ST4.03ch03	47519505..47521516	PGSC0003DMG400026479	Metal ion binding protein
ST4.03ch03	47531214..47533611	PGSC0003DMG400026494	Palmitoyl-acyl carrier protein thioesterase
ST4.03ch03	47547518..47548053	PGSC0003DMG400026499	Gene of unknown function
ST4.03ch03	47553314..47554113	PGSC0003DMG400026480	Mitotic-spindle organizing protein 1B
ST4.03ch03	47563557..47564906	PGSC0003DMG400026481	Inositol polyphosphate kinase
ST4.03ch03	47578499..47579122	PGSC0003DMG400026482	Protein HVA22
ST4.03ch03	47585757..47589106	PGSC0003DMG400026483	HVA22 e
ST4.03ch03	47594326..47595170	PGSC0003DMG400026484	Gene of unknown function
ST4.03ch03	47596864..47599863	PGSC0003DMG400026485	Steroleosin
ST4.03ch03	47600579..47602276	PGSC0003DMG400042259	Steroleosin
ST4.03ch03	47604195..47610674	PGSC0003DMG400026495	Gene of unknown function
ST4.03ch03	47632362..47632668	PGSC0003DMG400031743	Conserved gene of unknown function
ST4.03ch03	47650991..47659469	PGSC0003DMG400031735	Conserved gene of unknown function
ST4.03ch03	47695147..47700963	PGSC0003DMG400031730	Protein DEK
ST4.03ch03	47704728..47706692	PGSC0003DMG400031731	Feruloyl transferase
ST4.03ch03	47711499..47712968	PGSC0003DMG400031744	SAUR family protein
ST4.03ch03	47744480..47747638	PGSC0003DMG400031732	ATP binding
ST4.03ch03	47749812..47751781	PGSC0003DMG400031737	Conserved gene of unknown function
ST4.03ch03	47761057..47771749	PGSC0003DMG400031733	Ribosomal pseudouridine synthase
ST4.03ch03	47781309..47783293	PGSC0003DMG400031738	UPA16
ST4.03ch03	47825166..47826980	PGSC0003DMG401031741	UPA16
ST4.03ch03	47838609..47840449	PGSC0003DMG402031741	UPA16
ST4.03ch03	47860239..47862426	PGSC0003DMG400031742	UPA16
ST4.03ch03	47867400..47868097	PGSC0003DMG400031746	Gene of unknown function
ST4.03ch03	47958591..47959970	PGSC0003DMG400004335	MTN3 homolog
ST4.03ch03	47987093..47988146	PGSC0003DMG400004345	Gene of unknown function
ST4.03ch03	47989956..47990672	PGSC0003DMG400004330	Ulp1 protease family, C-terminal catalytic domain containing protein
ST4.03ch03	47994923..47996770	PGSC0003DMG400004337	MTN3
ST4.03ch03	48011707..48012164	PGSC0003DMG402004331	Conserved gene of unknown function
ST4.03ch03	48011922..48016603	PGSC0003DMG401004331	Zinc finger protein
ST4.03ch03	48017044..48019156	PGSC0003DMG400004338	Mitochondrial import inner membrane translocase subunit Tim8/small zinc finger
ST4.03ch03	48030127..48031759	PGSC0003DMG400004332	NAC domain protein
ST4.03ch03	48035601..48037948	PGSC0003DMG400004333	Conserved gene of unknown function

ST4.03ch03	48081373..48088382	PGSC0003DMG401004334	Pyruvate dehydrogenase
ST4.03ch03	48084220..48087524	PGSC0003DMG402004334	Pyruvate dehydrogenase E1 component subunit beta
ST4.03ch03	48087606..48088378	PGSC0003DMG403004334	Gene of unknown function
ST4.03ch03	48090273..48096387	PGSC0003DMG400004340	DNA binding protein
ST4.03ch03	48125374..48128012	PGSC0003DMG400004342	O-methyltransferase
ST4.03ch03	48129666..48130040	PGSC0003DMG400004343	Flavonoid 8-O-methyltransferase
ST4.03ch03	48231939..48233260	PGSC0003DMG400012024	O-methyltransferase
ST4.03ch03	48240576..48242568	PGSC0003DMG400045265	Gene of unknown function
ST4.03ch03	48248422..48249570	PGSC0003DMG400012023	O-methyltransferase
ST4.03ch03	48273251..48280627	PGSC0003DMG400012026	Conserved gene of unknown function
ST4.03ch03	48288363..48291063	PGSC0003DMG400012025	UPF0497 membrane protein 8
ST4.03ch03	48293775..48294625	PGSC0003DMG400012022	MdSFBB3-alpha protein
ST4.03ch03	48308180..48310280	PGSC0003DMG400012021	Armadillo/beta-catenin repeat family protein
ST4.03ch03	48325359..48325795	PGSC0003DMG400019863	RNase H family protein
ST4.03ch03	48335188..48338656	PGSC0003DMG400019852	Translocon-associated protein, beta subunit
ST4.03ch03	48339685..48342920	PGSC0003DMG400019853	Sterile alpha motif domain-containing protein
ST4.03ch03	48343038..48343841	PGSC0003DMG401019860	Alpha-2, 8-sialyltransferase 8b
ST4.03ch03	48343038..48349287	PGSC0003DMG402019860	Glycosyl transferase family 29 protein
ST4.03ch03	48382584..48385594	PGSC0003DMG400019861	BHLH transcription factor Upa20
ST4.03ch03	48396332..48396781	PGSC0003DMG400019855	Uncharacterized mitochondrial protein
ST4.03ch03	48398752..48407280	PGSC0003DMG400019856	Poly(A) polymerase
ST4.03ch03	48443252..48446715	PGSC0003DMG400019858	Pentatricopeptide repeat-containing protein
ST4.03ch03	48449743..48452807	PGSC0003DMG400019859	Nuclear transcription factor, X-box binding
ST4.03ch03	48458585..48461113	PGSC0003DMG400033693	UPA16
ST4.03ch03	48462312..48464791	PGSC0003DMG400033684	Cyc07
ST4.03ch03	48467210..48471492	PGSC0003DMG400033683	Dehydrogenase
ST4.03ch03	48482204..48483578	PGSC0003DMG400033696	MAPKK
ST4.03ch03	48492783..48495099	PGSC0003DMG400033682	Potassium channel SKOR
ST4.03ch03	48525070..48525564	PGSC0003DMG400033692	Stelar K+ outward rectifying channel
ST4.03ch03	48526013..48530094	PGSC0003DMG400033691	50S ribosomal protein L29, chloroplastic
ST4.03ch03	48530148..48532446	PGSC0003DMG400033695	DC1 domain containing protein
ST4.03ch03	48533468..48536913	PGSC0003DMG400033690	Conserved gene of unknown function
ST4.03ch03	48537414..48543917	PGSC0003DMG400033681	GTP-binding protein alpha subunit, gna
ST4.03ch03	48544949..48550522	PGSC0003DMG400033680	Protein SSU72
ST4.03ch03	48554650..48558518	PGSC0003DMG400033689	Conserved gene of unknown function
ST4.03ch03	48558976..48561647	PGSC0003DMG400033688	Tartrate-resistant acid phosphatase type 5
ST4.03ch03	48565204..48572401	PGSC0003DMG400033687	Hydrolase, alpha/beta fold family protein
ST4.03ch03	48598932..48602581	PGSC0003DMG400033685	Calmodulin
ST4.03ch03	48607958..48608787	PGSC0003DMG400033694	Zinc finger homeodomain protein SZF-HD1
ST4.03ch03	48688066..48691049	PGSC0003DMG400031049	Nucleic acid binding protein
ST4.03ch03	48718437..48719844	PGSC0003DMG400037321	UPF0497 membrane protein
ST4.03ch03	48724509..48725606	PGSC0003DMG400031035	Conserved gene of unknown function
ST4.03ch03	48726874..48730768	PGSC0003DMG401031036	Chalcone reductase
ST4.03ch03	48728065..48729471	PGSC0003DMG402031036	Aldo-keto reductase

ST4.03ch03	48729228..48737666	PGSC0003DMG400031050	Conserved gene of unknown function
ST4.03ch03	48733753..48734143	PGSC0003DMG400031060	Gene of unknown function
ST4.03ch03	48738340..48742254	PGSC0003DMG400031051	Conserved gene of unknown function
ST4.03ch03	48745281..48746801	PGSC0003DMG401031052	Conserved gene of unknown function
ST4.03ch03	48747013..48751559	PGSC0003DMG402031052	Polysialic acid capsule expression protein kpsF
ST4.03ch03	48761799..48765775	PGSC0003DMG401031037	ATP binding protein
ST4.03ch03	48767478..48771567	PGSC0003DMG402031037	Kinase family protein
ST4.03ch03	48785885..48793264	PGSC0003DMG400031053	Tubulin-specific chaperone B
ST4.03ch03	48796402..48804322	PGSC0003DMG400031039	Conserved gene of unknown function
ST4.03ch03	48805756..48810618	PGSC0003DMG400031054	Transcription factor
ST4.03ch03	48812918..48813880	PGSC0003DMG400031040	Glycosyl hydrolases family 17 protein
ST4.03ch03	48851581..48856943	PGSC0003DMG400031057	Serine palmitoyltransferase
ST4.03ch03	48894786..48895620	PGSC0003DMG400037684	Double-stranded RNA binding protein
ST4.03ch03	48900767..48907402	PGSC0003DMG400031041	D-cysteine desulfhydrase
ST4.03ch03	48908751..48912422	PGSC0003DMG400031042	Glutamate decarboxylase isoform1
ST4.03ch03	48913347..48918844	PGSC0003DMG400031043	DNA polymerase epsilon subunit B
ST4.03ch03	48925944..48929097	PGSC0003DMG400031044	MYB transcription factor
ST4.03ch03	48938395..48945842	PGSC0003DMG401031045	AGO1-1
ST4.03ch03	48940900..48941627	PGSC0003DMG402031045	Argonaute 1
ST4.03ch03	48948994..48950010	PGSC0003DMG400031046	Sucrose synthase
ST4.03ch03	48969450..48973461	PGSC0003DMG400031047	Ornithine decarboxylase
ST4.03ch03	48982747..48987087	PGSC0003DMG400031059	Conserved gene of unknown function
ST4.03ch03	49030586..49031219	PGSC0003DMG400028082	Gene of unknown function
ST4.03ch03	49068083..49068790	PGSC0003DMG400024452	Pyridoxal-dependent decarboxylase, C-terminal sheet domain containing protein
ST4.03ch03	49135756..49136999	PGSC0003DMG400025628	Pyridoxal-dependent decarboxylase, C-terminal sheet domain containing protein
ST4.03ch03	49139317..49143992	PGSC0003DMG400025632	Myb-like DNA-binding protein
ST4.03ch03	49152065..49158071	PGSC0003DMG400025629	Conserved gene of unknown function
ST4.03ch03	49159146..49163130	PGSC0003DMG400025630	Conserved gene of unknown function
ST4.03ch03	49164765..49165850	PGSC0003DMG400025633	F-box protein interaction domain containing protein
ST4.03ch03	49166847..49168407	PGSC0003DMG400025634	F-box protein interaction domain containing protein
ST4.03ch03	49169514..49169897	PGSC0003DMG400025636	Receptor kinase
ST4.03ch03	49185119..49186624	PGSC0003DMG400025631	F-box protein interaction domain containing protein
ST4.03ch03	49188663..49191683	PGSC0003DMG400025635	Leucine rich repeat containing protein
ST4.03ch03	49213733..49215517	PGSC0003DMG400010183	Receptor kinase
ST4.03ch03	49217068..49217644	PGSC0003DMG401010182	Receptor kinase
ST4.03ch03	49217733..49218479	PGSC0003DMG400010159	Conserved gene of unknown function
ST4.03ch03	49219417..49222365	PGSC0003DMG401010181	Pentatricopeptide repeat domain containing protein
ST4.03ch03	49222889..49226202	PGSC0003DMG402010181	Xyloglucan endotransglucosylase/hydrolase protein 9
ST4.03ch03	49226872..49231147	PGSC0003DMG400010180	TPR domain containing protein
ST4.03ch03	49230741..49235734	PGSC0003DMG400010158	Kinase interacting protein
ST4.03ch03	49236384..49246086	PGSC0003DMG400010157	Forever young oxidoreductase
ST4.03ch03	49247565..49251425	PGSC0003DMG400010156	Small nuclear ribonucleoprotein polypeptide
ST4.03ch03	49252642..49254408	PGSC0003DMG400010155	Gene of unknown function
ST4.03ch03	49268925..49270398	PGSC0003DMG400010154	Conserved gene of unknown function

ST4.03ch03	49280471..49283620	PGSC0003DMG400010153	ARF-GAP domain 15
ST4.03ch03	49297425..49302439	PGSC0003DMG400010179	Gene of unknown function
ST4.03ch03	49303047..49304192	PGSC0003DMG400010178	F-box family protein
ST4.03ch03	49304609..49306399	PGSC0003DMG400010177	Pentatricopeptide
ST4.03ch03	49307894..49311517	PGSC0003DMG400010176	Zinc finger protein
ST4.03ch03	49312334..49318212	PGSC0003DMG400010152	Integral membrane protein
ST4.03ch03	49320205..49329385	PGSC0003DMG400010151	ARC6H
ST4.03ch03	49339491..49346448	PGSC0003DMG400010175	DNA-binding protein RHL1
ST4.03ch03	49346924..49353854	PGSC0003DMG400010150	Conserved gene of unknown function
ST4.03ch03	49358488..49364040	PGSC0003DMG400010174	Peroxisomal membrane 22 kDa family protein
ST4.03ch03	49371716..49373868	PGSC0003DMG400010173	Avr9/Cf-9 rapidly elicited protein 140
ST4.03ch03	49375470..49376370	PGSC0003DMG400010149	Conserved gene of unknown function
ST4.03ch03	49377506..49378824	PGSC0003DMG400010148	Miraculin
ST4.03ch03	49385273..49385821	PGSC0003DMG400045366	Conserved gene of unknown function
ST4.03ch03	49392670..49393484	PGSC0003DMG400010147	Kunitz-type enzyme inhibitor S9C11
ST4.03ch03	49448372..49449153	PGSC0003DMG400010146	Kunitz-type tuber invertase inhibitor
ST4.03ch03	49463988..49464383	PGSC0003DMG400045144	Gene of unknown function
ST4.03ch03	49498051..49498873	PGSC0003DMG400010145	Cysteine protease inhibitor 9
ST4.03ch03	49514289..49514969	PGSC0003DMG400010144	Stigma expressed protein
ST4.03ch03	49548299..49549151	PGSC0003DMG400010143	Cysteine protease inhibitor 1
ST4.03ch03	49567115..49567661	PGSC0003DMG400010189	Gene of unknown function
ST4.03ch03	49611604..49612275	PGSC0003DMG400010141	Stigma expressed protein
ST4.03ch03	49629957..49632989	PGSC0003DMG400010140	Succinic semialdehyde reductase isofom2
ST4.03ch03	49644579..49645247	PGSC0003DMG400010139	Cysteine protease inhibitor 1
ST4.03ch03	49665048..49665619	PGSC0003DMG400010188	Gene of unknown function
ST4.03ch03	49685391..49686264	PGSC0003DMG400010137	Cysteine protease inhibitor 1
ST4.03ch03	49713158..49714012	PGSC0003DMG400010136	Stigma expressed protein
ST4.03ch03	49754402..49754857	PGSC0003DMG400010135	Ethylene-responsive element-binding family protein
ST4.03ch03	49755550..49756903	PGSC0003DMG400010172	Gene of unknown function
ST4.03ch03	49773998..49774469	PGSC0003DMG400010186	Kunitz-type enzyme inhibitor S9C11
ST4.03ch03	49784928..49785518	PGSC0003DMG400010134	Cysteine protease inhibitor 1
ST4.03ch03	49838683..49840071	PGSC0003DMG400010170	Miraculin
ST4.03ch03	49840939..49843914	PGSC0003DMG400010133	Sexual organ expressed protein
ST4.03ch03	49859174..49860099	PGSC0003DMG400010132	Conserved gene of unknown function
ST4.03ch03	49862777..49863532	PGSC0003DMG400010131	Nb cell deth marker
ST4.03ch03	49870078..49871332	PGSC0003DMG400010130	Conserved gene of unknown function
ST4.03ch03	49926018..49926578	PGSC0003DMG400009512	Kunitz-type proteinase inhibitor
ST4.03ch03	49930058..49930845	PGSC0003DMG400009513	Aspartic protease inhibitor 5
ST4.03ch03	49933648..49935405	PGSC0003DMG400036195	Gene of unknown function
ST4.03ch03	49941306..49942079	PGSC0003DMG400009514	Kunitz-type protease inhibitor
ST4.03ch03	49987262..49993251	PGSC0003DMG400039813	Gene of unknown function
ST4.03ch03	50030746..50031538	PGSC0003DMG400004548	Proteinase inhibitor type-2 CEVI57
ST4.03ch03	50033588..50034132	PGSC0003DMG400004549	Proteinase inhibitor type-2 CEVI57
ST4.03ch03	50056094..50056945	PGSC0003DMG400004547	Proteinase inhibitor type-2 P303.51

ST4.03ch03	50110387..50111368	PGSC0003DMG400015290	Proteinase inhibitor type-2 CEVI57
ST4.03ch03	50127073..50129569	PGSC0003DMG400015307	Gene of unknown function
ST4.03ch03	50143509..50144247	PGSC0003DMG400015289	Proteinase inhibitor type-2 CEVI57
ST4.03ch03	50172620..50181230	PGSC0003DMG400035007	Conserved gene of unknown function
ST4.03ch03	50185156..50186034	PGSC0003DMG400015287	Proteinase inhibitor type-2 CEVI57
ST4.03ch03	50189248..50189808	PGSC0003DMG400039762	Proteinase inhibitor type-2 CEVI57
ST4.03ch03	50197884..50198633	PGSC0003DMG400015286	Miraculin
ST4.03ch03	50225873..50226839	PGSC0003DMG400015219	Miraculin
ST4.03ch03	50228194..50231917	PGSC0003DMG400015218	F-box protein interaction domain containing protein
ST4.03ch03	50237284..50239059	PGSC0003DMG400015284	EMB2016
ST4.03ch03	50242011..50244591	PGSC0003DMG400037483	Gene of unknown function
ST4.03ch03	50249138..50250573	PGSC0003DMG400015217	F-box protein interaction domain containing protein
ST4.03ch03	50253994..50257278	PGSC0003DMG400015283	Receptor kinase
ST4.03ch03	50257731..50259270	PGSC0003DMG400015282	F-box protein interaction domain containing protein
ST4.03ch03	50259543..50266313	PGSC0003DMG400015216	CDT1a protein
ST4.03ch03	50277358..50284514	PGSC0003DMG400015281	Conserved gene of unknown function
ST4.03ch03	50285444..50293060	PGSC0003DMG400015215	Asparaginyl-tRNA synthetase, chloroplastic/mitochondrial
ST4.03ch03	50294700..50298052	PGSC0003DMG400015280	Conserved gene of unknown function
ST4.03ch03	50296177..50296648	PGSC0003DMG400015306	Gene of unknown function
ST4.03ch03	50298182..50299202	PGSC0003DMG400015214	Conserved gene of unknown function
ST4.03ch03	50299886..50302826	PGSC0003DMG400015213	Conserved gene of unknown function
ST4.03ch03	50305587..50313172	PGSC0003DMG400015212	Serine/threonine-protein phosphatase
ST4.03ch03	50314691..50324678	PGSC0003DMG400015211	Beta-galactosidase
ST4.03ch03	50329956..50334109	PGSC0003DMG400015210	UPF0426 protein, chloroplastic
ST4.03ch03	50339125..50342642	PGSC0003DMG400015278	Cytochrome P450
ST4.03ch03	50343898..50346888	PGSC0003DMG400015277	Conserved gene of unknown function
ST4.03ch03	50344166..50350920	PGSC0003DMG402015209	NPK2
ST4.03ch03	50351723..50357435	PGSC0003DMG401015209	Conserved gene of unknown function
ST4.03ch03	50362741..50366176	PGSC0003DMG400015276	Serine-threonine protein kinase, plant-type
ST4.03ch03	50367048..50368315	PGSC0003DMG400015275	Aquaporin, MIP family, TIP subfamily
ST4.03ch03	50368876..50373810	PGSC0003DMG400015208	Ser/Thr protein phosphatase family
ST4.03ch03	50374941..50378394	PGSC0003DMG400015274	DNA binding protein
ST4.03ch03	50381093..50388279	PGSC0003DMG400015273	Alpha-galactosidase
ST4.03ch03	50392706..50394025	PGSC0003DMG400015272	DNA binding protein
ST4.03ch03	50401614..50402275	PGSC0003DMG400015304	NAD(P)H-quinone oxidoreductase subunit 3, chloroplastic
ST4.03ch03	50403123..50405419	PGSC0003DMG400015271	P40
ST4.03ch03	50406273..50414267	PGSC0003DMG400015207	Conserved gene of unknown function
ST4.03ch03	50423170..50427354	PGSC0003DMG400015270	ATP-binding cassette transporter
ST4.03ch03	50429992..50431539	PGSC0003DMG400015269	GRP 2
ST4.03ch03	50431718..50432257	PGSC0003DMG400015206	Conserved gene of unknown function
ST4.03ch03	50436856..50443765	PGSC0003DMG400015268	Ubiquitin activating enzyme
ST4.03ch03	50449187..50455703	PGSC0003DMG402015205	Protein yrdA
ST4.03ch03	50457307..50460580	PGSC0003DMG401015205	TDR8 protein
ST4.03ch03	50470725..50471314	PGSC0003DMG400015204	Conserved gene of unknown function

ST4.03ch03	50477078..50477923	PGSC0003DMG400015267	Kunitz-type protease inhibitor
ST4.03ch03	50478770..50479219	PGSC0003DMG400015303	Conserved gene of unknown function
ST4.03ch03	50480853..50487293	PGSC0003DMG400015266	CBS domain containing protein
ST4.03ch03	50489074..50492190	PGSC0003DMG400015265	Phospholipase C 4
ST4.03ch03	50494186..50496764	PGSC0003DMG400015264	Thylakoid lumenal 17.9 kDa protein, chloroplast
ST4.03ch03	50502524..50507168	PGSC0003DMG400015263	Nodulin
ST4.03ch03	50509027..50511444	PGSC0003DMG400015262	Heat shock protein binding protein
ST4.03ch03	50520674..50521120	PGSC0003DMG400015261	Heat shock protein binding protein
ST4.03ch03	50526085..50526531	PGSC0003DMG400039636	Ccaat-binding transcription factor
ST4.03ch03	50535042..50535506	PGSC0003DMG400015260	CONSTANS interacting protein 2a
ST4.03ch03	50559294..50564194	PGSC0003DMG402015259	CONSTANS interacting protein 2a
ST4.03ch03	50565407..50565925	PGSC0003DMG401015259	Gene of unknown function
ST4.03ch03	50565407..50568184	PGSC0003DMG400015203	Protein transport protein SEC61 subunit gamma
ST4.03ch03	50578693..50579928	PGSC0003DMG400015258	DNA-directed RNA polymerase
ST4.03ch03	50581219..50582116	PGSC0003DMG400015302	Conserved gene of unknown function
ST4.03ch03	50584576..50587529	PGSC0003DMG402015202	Reverse transcriptase family member
ST4.03ch03	50610508..50614942	PGSC0003DMG401015202	Histone acetyltransferase
ST4.03ch03	50616754..50618798	PGSC0003DMG402015201	Receptor expression-enhancing protein
ST4.03ch03	50625581..50626068	PGSC0003DMG401015201	Receptor expression-enhancing protein
ST4.03ch03	50628919..50632108	PGSC0003DMG400015257	Ribosome
ST4.03ch03	50652743..50654344	PGSC0003DMG400015255	DELLA protein RGL1
ST4.03ch03	50655402..50657234	PGSC0003DMG400015254	Nucleoside diphosphate kinase 2, chloroplastic
ST4.03ch03	50658327..50662723	PGSC0003DMG400015200	GDP-mannose transporter
ST4.03ch03	50663176..50666491	PGSC0003DMG400015199	Conserved gene of unknown function
ST4.03ch03	50668234..50668746	PGSC0003DMG400015301	Conserved gene of unknown function
ST4.03ch03	50686141..50689501	PGSC0003DMG400015253	Glyceraldehyde-3-phosphate dehydrogenase
ST4.03ch03	50690497..50696246	PGSC0003DMG400015252	Conserved gene of unknown function
ST4.03ch03	50696476..50700303	PGSC0003DMG400015198	Amino acid transporter
ST4.03ch03	50702249..50714294	PGSC0003DMG400015251	Plastid RNA-binding protein
ST4.03ch03	50723631..50728564	PGSC0003DMG400015249	3-oxoacyl-(Acyl-carrier protein) reductase
ST4.03ch03	50728416..50735928	PGSC0003DMG400015197	Bromodomain-containing protein
ST4.03ch03	50747649..50749043	PGSC0003DMG400015196	Conserved gene of unknown function
ST4.03ch03	50794349..50796366	PGSC0003DMG400015195	Conserved gene of unknown function
ST4.03ch03	50799936..50800979	PGSC0003DMG400015248	Immediate early protein homolog
ST4.03ch03	50835985..50839603	PGSC0003DMG400015194	Malate synthase
ST4.03ch03	50843085..50843546	PGSC0003DMG402015193	Malate synthase, glyoxysomal
ST4.03ch03	50844030..50844353	PGSC0003DMG401015193	Malate synthase
ST4.03ch03	50846704..50849224	PGSC0003DMG400015192	Malate synthase, glyoxysomal
ST4.03ch03	50849874..50855089	PGSC0003DMG400015191	Conserved gene of unknown function
ST4.03ch03	50869963..50872171	PGSC0003DMG400015299	Gene of unknown function
ST4.03ch03	50875724..50885587	PGSC0003DMG400015246	Protein tyrosine phosphatase
ST4.03ch03	50885904..50891146	PGSC0003DMG400015190	AMP dependent CoA ligase
ST4.03ch03	50893836..50901875	PGSC0003DMG400015189	Aspartyl aminopeptidase
ST4.03ch03	50902946..50905075	PGSC0003DMG400015188	Auxin-independent growth promoter

ST4.03ch03	50905849..50909925	PGSC0003DMG400015187	Adenylate kinase B
ST4.03ch03	50913898..50915790	PGSC0003DMG400015186	Pentatricopeptide repeat-containing protein, chloroplastic
ST4.03ch03	50919866..50922316	PGSC0003DMG400015245	Ribosomal protein PETRP
ST4.03ch03	50939847..50943465	PGSC0003DMG403015244	Brg-1 associated factor
ST4.03ch03	50944271..50953745	PGSC0003DMG402015244	EMB2746
ST4.03ch03	50954189..50957371	PGSC0003DMG401015244	Conserved gene of unknown function
ST4.03ch03	50958078..50963959	PGSC0003DMG400015243	SAP domain-containing protein
ST4.03ch03	50982145..50983906	PGSC0003DMG400015185	Cytochrome P450
ST4.03ch03	50988577..50990118	PGSC0003DMG400015184	Cytochrome P450
ST4.03ch03	51005600..51007272	PGSC0003DMG400015183	Cytochrome P450
ST4.03ch03	51016453..51018063	PGSC0003DMG400015242	Cytochrome P450
ST4.03ch03	51025275..51029940	PGSC0003DMG400015241	AMP-activated protein kinase, gamma regulatory subunit
ST4.03ch03	51037214..51039217	PGSC0003DMG400039752	Exocyst complex component
ST4.03ch03	51044566..51049822	PGSC0003DMG402015240	Conserved gene of unknown function
ST4.03ch03	51050161..51058160	PGSC0003DMG401015240	Ubiquitin-activating enzyme E1 domain-containing protein
ST4.03ch03	51053171..51053969	PGSC0003DMG400015298	NAD(P)H-quinone oxidoreductase subunit 2 A, chloroplastic
ST4.03ch03	51058389..51062068	PGSC0003DMG400015182	Deoxycytidylate deaminase
ST4.03ch03	51062829..51067352	PGSC0003DMG400015239	Coproporphyrinigen III oxidase
ST4.03ch03	51066797..51070700	PGSC0003DMG400015181	Nucleic acid binding protein
ST4.03ch03	51071358..51079247	PGSC0003DMG400015180	Gamma tubulin
ST4.03ch03	51082104..51091873	PGSC0003DMG400015238	Kelch repeat protein
ST4.03ch03	51103949..51105853	PGSC0003DMG400015237	Purine permease
ST4.03ch03	51122654..51127262	PGSC0003DMG400015297	Conserved gene of unknown function
ST4.03ch03	51127315..51129996	PGSC0003DMG400015178	Heat shock protein
ST4.03ch03	51132926..51136036	PGSC0003DMG400015177	CONSTANS interacting protein 2a
ST4.03ch03	51139267..51141290	PGSC0003DMG400015176	Gene of unknown function
ST4.03ch03	51155179..51162521	PGSC0003DMG401015175	B3 domain-containing protein Os01g0905400
ST4.03ch03	51155205..51156460	PGSC0003DMG402015175	Gene of unknown function
ST4.03ch03	51158056..51158435	PGSC0003DMG400015294	Gene of unknown function
ST4.03ch03	51158674..51159340	PGSC0003DMG400015293	Gene of unknown function
ST4.03ch03	51164499..51165797	PGSC0003DMG400015236	Gene of unknown function
ST4.03ch03	51170046..51172608	PGSC0003DMG400015235	Ubiquitin-protein ligase
ST4.03ch03	51177387..51177785	PGSC0003DMG400015174	DUF26 domain-containing protein 2
ST4.03ch03	51192622..51195303	PGSC0003DMG400015173	ATP binding protein
ST4.03ch03	51211912..51212303	PGSC0003DMG400015292	ATP binding protein
ST4.03ch03	51224758..51228491	PGSC0003DMG400015171	ATP binding protein
ST4.03ch03	51230924..51234395	PGSC0003DMG400015170	ATP binding protein
ST4.03ch03	51237136..51239528	PGSC0003DMG400015169	Esterase
ST4.03ch03	51249900..51306775	PGSC0003DMG400015233	Transferase, transferring glycosyl groups
ST4.03ch03	51308561..51318350	PGSC0003DMG400015168	Nucleic acid binding protein
ST4.03ch03	51320232..51328435	PGSC0003DMG400015167	Zinc finger protein
ST4.03ch03	51344990..51349516	PGSC0003DMG400015165	2-deoxyglucose-6-phosphate phosphatase
ST4.03ch03	51352514..51353923	PGSC0003DMG400015164	Conserved gene of unknown function
ST4.03ch03	51386421..51389350	PGSC0003DMG400015232	Pentatricopeptide repeat-containing protein

ST4.03ch03	51390262..51396957	PGSC0003DMG400015231	Ufm1-specific protease
ST4.03ch03	51401507..51405421	PGSC0003DMG400015162	Nodulation receptor kinase
ST4.03ch03	51406268..51412317	PGSC0003DMG400015161	Replication factor C / DNA polymerase III gamma-tau subunit
ST4.03ch03	51432584..51434561	PGSC0003DMG400015230	Pectate lyase
ST4.03ch03	51465624..51468593	PGSC0003DMG400015229	BTB/POZ domain-containing protein
ST4.03ch03	51470885..51475042	PGSC0003DMG400015160	KDEL-tailed cysteine endopeptidase
ST4.03ch03	51471303..51474965	PGSC0003DMG400015228	Peptide methionine sulfoxide reductase
ST4.03ch03	51479433..51485736	PGSC0003DMG402015159	Protein tipD
ST4.03ch03	51499826..51500958	PGSC0003DMG401015159	Eukaryotic translation initiation factor 2c
ST4.03ch03	51505704..51510446	PGSC0003DMG400015158	Protein bem46
ST4.03ch03	51528931..51531438	PGSC0003DMG400015227	Conserved gene of unknown function
ST4.03ch03	51533595..51535855	PGSC0003DMG400015157	Receptor kinase
ST4.03ch03	51543477..51546492	PGSC0003DMG400015156	Conserved gene of unknown function
ST4.03ch03	51551313..51555625	PGSC0003DMG400015226	Protein-L-isoaspartate O-methyltransferase
ST4.03ch03	51556001..51560104	PGSC0003DMG400015155	28 kDa ribonucleoprotein, chloroplastic
ST4.03ch03	51561898..51566162	PGSC0003DMG400015154	Hydrolase, hydrolyzing O-glycosyl compounds
ST4.03ch03	51570170..51578078	PGSC0003DMG400046688	Gene of unknown function
ST4.03ch03	51581151..51583454	PGSC0003DMG400015225	Transposase
ST4.03ch03	51598600..51599166	PGSC0003DMG400015222	Transposase
ST4.03ch03	51601379..51606789	PGSC0003DMG400015153	Tryptophan biosynthesis protein, trpc
ST4.03ch03	51608762..51617317	PGSC0003DMG400015152	Copine
ST4.03ch03	51626123..51629028	PGSC0003DMG400015151	Cytochrome P450
ST4.03ch03	51633709..51635114	PGSC0003DMG400015150	Cytochrome P450 71A4
ST4.03ch03	51638104..51639500	PGSC0003DMG400015149	Cytochrome P450 71A4
ST4.03ch03	51641856..51643958	PGSC0003DMG400015221	Cytochrome P450 71A2
ST4.03ch03	51650729..51652620	PGSC0003DMG400015220	Cytochrome P450 71A4
ST4.03ch03	51745310..51745992	PGSC0003DMG400005554	Cytochrome P450 71A4
ST4.03ch03	51812217..51813743	PGSC0003DMG400018199	Cytochrome P450 71A4
ST4.03ch03	51814334..51816154	PGSC0003DMG400018141	Cytochrome P450 71A4
ST4.03ch03	51817344..51819661	PGSC0003DMG400018140	Cytochrome P450 71A4
ST4.03ch03	51821683..51824023	PGSC0003DMG400018139	Cytochrome P450 71A4
ST4.03ch03	51824105..51826186	PGSC0003DMG400018138	Cytochrome P450 71A4
ST4.03ch03	51848408..51849215	PGSC0003DMG400018136	Cytochrome P450 71A2
ST4.03ch03	51857417..51859571	PGSC0003DMG400018135	Cytochrome P450 71A4
ST4.03ch03	51883788..51884984	PGSC0003DMG400018198	70 kDa subunit of replication protein A
ST4.03ch03	51885505..51886302	PGSC0003DMG400018134	Cytochrome P450 71A4
ST4.03ch03	51893606..51894994	PGSC0003DMG400018133	Cytochrome P450 71A4
ST4.03ch03	51897826..51900459	PGSC0003DMG400018132	Cytochrome P450 71A2
ST4.03ch03	51908390..51908728	PGSC0003DMG400018197	Cytochrome P450 71A4
ST4.03ch03	51909751..51911623	PGSC0003DMG400018131	CYP71AU1
ST4.03ch03	51918881..51919990	PGSC0003DMG400018196	CYP71AU1
ST4.03ch03	51933988..51937884	PGSC0003DMG400018195	Afc
ST4.03ch03	51949423..51956787	PGSC0003DMG400018194	Conserved gene of unknown function
ST4.03ch03	51957161..51961663	PGSC0003DMG400018130	D-3-phosphoglycerate dehydrogenase

ST4.03ch03	51962141..51966797	PGSC0003DMG400018193	Hydrolase
ST4.03ch03	51966265..51973422	PGSC0003DMG400018129	High-affinity nitrate transport system component
ST4.03ch03	51984424..51988635	PGSC0003DMG400018192	Conserved gene of unknown function
ST4.03ch03	51989958..51991673	PGSC0003DMG400018128	Protein phosphatase 2c
ST4.03ch03	51998069..52005507	PGSC0003DMG400018191	Kinase
ST4.03ch03	52006116..52007990	PGSC0003DMG400018190	Elongation factor TuA, chloroplastic
ST4.03ch03	52010641..52013263	PGSC0003DMG400018127	Protein DEHYDRATION-INDUCED 19 homolog 6
ST4.03ch03	52023650..52024174	PGSC0003DMG400040957	Pectinesterase inhibitor
ST4.03ch03	52075082..52075546	PGSC0003DMG400034620	Pectinesterase inhibitor
ST4.03ch03	52079987..52080508	PGSC0003DMG400018189	Pectinesterase inhibitor
ST4.03ch03	52085642..52086187	PGSC0003DMG400018188	Ubiquitin-protein ligase
ST4.03ch03	52101613..52106705	PGSC0003DMG400018187	Pentatricopeptide repeat-containing protein
ST4.03ch03	52107190..52108048	PGSC0003DMG400018186	Conserved gene of unknown function
ST4.03ch03	52108928..52109738	PGSC0003DMG400018125	Gene of unknown function
ST4.03ch03	52111014..52111628	PGSC0003DMG400018124	Gene of unknown function
ST4.03ch03	52119132..52119801	PGSC0003DMG400018123	Gene of unknown function
ST4.03ch03	52121801..52126137	PGSC0003DMG400018122	Zinc ion binding protein
ST4.03ch03	52133169..52133993	PGSC0003DMG400018121	Conserved gene of unknown function
ST4.03ch03	52145382..52146343	PGSC0003DMG400042642	Conserved gene of unknown function
ST4.03ch03	52147910..52149090	PGSC0003DMG400018209	Gene of unknown function
ST4.03ch03	52151644..52154051	PGSC0003DMG400018120	TRANSPARENT TESTA 12 protein
ST4.03ch03	52160096..52162997	PGSC0003DMG400018119	TRANSPARENT TESTA 12 protein
ST4.03ch03	52176377..52179267	PGSC0003DMG400018118	MATE efflux family protein
ST4.03ch03	52180061..52184884	PGSC0003DMG400018117	Leucine-rich repeat-containing protein
ST4.03ch03	52185305..52190958	PGSC0003DMG400018116	C2 and GRAM domain-containing protein
ST4.03ch03	52194200..52199382	PGSC0003DMG400018185	Conserved gene of unknown function
ST4.03ch03	52202443..52204300	PGSC0003DMG400018115	ATP binding protein
ST4.03ch03	52212143..52214553	PGSC0003DMG400018184	Carbohydrate binding protein
ST4.03ch03	52215659..52218666	PGSC0003DMG400018114	ATFRO8/FRO8
ST4.03ch03	52222534..52225055	PGSC0003DMG400018183	ATP binding protein
ST4.03ch03	52228924..52230643	PGSC0003DMG400018182	Ring finger protein
ST4.03ch03	52233126..52242171	PGSC0003DMG400018181	DEAD-box ATP-dependent RNA helicase 30
ST4.03ch03	52242765..52243471	PGSC0003DMG400018180	Ribosomal protein L27a
ST4.03ch03	52247234..52250103	PGSC0003DMG400018179	Armadillo/beta-catenin repeat family protein
ST4.03ch03	52251311..52253379	PGSC0003DMG400018113	R2r3-myb transcription factor
ST4.03ch03	52276012..52276389	PGSC0003DMG400018207	Gene of unknown function
ST4.03ch03	52277098..52280098	PGSC0003DMG400018178	Histone deacetylase
ST4.03ch03	52281585..52284607	PGSC0003DMG400018177	Pentatricopeptide repeat-containing protein
ST4.03ch03	52299410..52301453	PGSC0003DMG400018112	Protein LATERAL ORGAN BOUNDARIES
ST4.03ch03	52311805..52312667	PGSC0003DMG400018176	Oleosin
ST4.03ch03	52333022..52338905	PGSC0003DMG400018110	Alliin lyase
ST4.03ch03	52344712..52345833	PGSC0003DMG400045225	Gene of unknown function
ST4.03ch03	52369689..52370336	PGSC0003DMG400037301	NB-ARC domain containing protein
ST4.03ch03	52379137..52380159	PGSC0003DMG400034404	Gene of unknown function

ST4.03ch03	52382294..52383250	PGSC0003DMG400045825	Gene of unknown function
ST4.03ch03	52385241..52385546	PGSC0003DMG400034803	Gene of unknown function
ST4.03ch03	52396390..52399121	PGSC0003DMG400018109	Raffionse synthase 2
ST4.03ch03	52400123..52405683	PGSC0003DMG400018175	Nucleic acid binding
ST4.03ch03	52405924..52413862	PGSC0003DMG400018108	FKBP12-interacting protein of 37 kDa
ST4.03ch03	52414815..52424083	PGSC0003DMG400018107	Notchless
ST4.03ch03	52432496..52437414	PGSC0003DMG400018174	Short-chain dehydrogenase
ST4.03ch03	52438388..52445179	PGSC0003DMG400018106	Conserved gene of unknown function
ST4.03ch03	52448904..52453290	PGSC0003DMG400018173	Pentatricopeptide repeat-containing protein, mitochondrial
ST4.03ch03	52455323..52465052	PGSC0003DMG400018172	Conserved gene of unknown function
ST4.03ch03	52473676..52478435	PGSC0003DMG400018171	Receptor protein kinase
ST4.03ch03	52481260..52482408	PGSC0003DMG400018105	Ubiquitin-protein ligase
ST4.03ch03	52483744..52487983	PGSC0003DMG400018104	Cell division cycle protein 48
ST4.03ch03	52489653..52490699	PGSC0003DMG400034990	F-box protein interaction domain containing protein
ST4.03ch03	52492633..52493652	PGSC0003DMG400018170	Conserved gene of unknown function
ST4.03ch03	52500043..52503055	PGSC0003DMG400018169	Cyclin D2.1 protein
ST4.03ch03	52503527..52508405	PGSC0003DMG400018168	Transcription factor
ST4.03ch03	52510915..52514983	PGSC0003DMG400018103	Carboxyl-terminal proteinase
ST4.03ch03	52524053..52526862	PGSC0003DMG400018167	UBX domain-containing protein 8-B
ST4.03ch03	52538882..52543092	PGSC0003DMG400018166	Transporter
ST4.03ch03	52544054..52546874	PGSC0003DMG400018165	Heme-binding protein
ST4.03ch03	52555424..52563443	PGSC0003DMG402018164	Protein MODIFIER OF SNC1 1
ST4.03ch03	52564527..52570400	PGSC0003DMG401018164	EMB2759
ST4.03ch03	52576899..52580137	PGSC0003DMG400018163	Serine-threonine protein kinase, plant-type
ST4.03ch03	52581838..52584136	PGSC0003DMG400018162	Trithorax
ST4.03ch03	52584257..52585523	PGSC0003DMG400018102	Calmodulin-binding protein
ST4.03ch03	52587339..52587794	PGSC0003DMG400040180	SKP1 1
ST4.03ch03	52589288..52592358	PGSC0003DMG400018161	Ubiquitin carrier protein
ST4.03ch03	52593703..52596962	PGSC0003DMG400018101	BRASSINOSTEROID INSENSITIVE 1-associated receptor kinase 1
ST4.03ch03	52597981..52598441	PGSC0003DMG400037011	Homeobox 9
ST4.03ch03	52600416..52602649	PGSC0003DMG400018100	Homeobox 9
ST4.03ch03	52611505..52613549	PGSC0003DMG400018160	Conserved gene of unknown function
ST4.03ch03	52616469..52623072	PGSC0003DMG400018099	Structure-specific recognition protein
ST4.03ch03	52624191..52626893	PGSC0003DMG400018098	Conserved gene of unknown function
ST4.03ch03	52628876..52633639	PGSC0003DMG402018159	Ubiquitin carrier protein
ST4.03ch03	52635181..52637606	PGSC0003DMG400018206	Conserved gene of unknown function
ST4.03ch03	52637713..52638405	PGSC0003DMG401018159	Conserved gene of unknown function
ST4.03ch03	52650396..52659108	PGSC0003DMG400018158	Conserved gene of unknown function
ST4.03ch03	52659498..52662549	PGSC0003DMG400018157	Glutaredoxin
ST4.03ch03	52662939..52664521	PGSC0003DMG400018156	Ubiquitin-protein ligase
ST4.03ch03	52670509..52671847	PGSC0003DMG400018155	Ubiquitin-protein ligase
ST4.03ch03	52672407..52674636	PGSC0003DMG400018097	Conserved gene of unknown function
ST4.03ch03	52679293..52682714	PGSC0003DMG400018154	Conserved gene of unknown function
ST4.03ch03	52682813..52688251	PGSC0003DMG400018096	U3 small nucleolar ribonucleoprotein protein mpp10

ST4.03ch03	52709732..52710076	PGSC0003DMG400037864	Raffionse synthase 2
ST4.03ch03	52720586..52721251	PGSC0003DMG400034843	Gene of unknown function
ST4.03ch03	52732972..52733406	PGSC0003DMG400039684	Gene of unknown function
ST4.03ch03	52736187..52740429	PGSC0003DMG400018153	Gibberellin receptor GID1
ST4.03ch03	52754214..52756452	PGSC0003DMG400018095	Conserved gene of unknown function
ST4.03ch03	52761138..52762995	PGSC0003DMG400018094	Ribosomal protein L41
ST4.03ch03	52767118..52770081	PGSC0003DMG401018151	Pre-mRNA-splicing factor cwc23
ST4.03ch03	52767195..52772875	PGSC0003DMG402018151	ATP binding protein
ST4.03ch03	52773840..52778005	PGSC0003DMG400018150	Phosphate/phosphoenolpyruvate translocator
ST4.03ch03	52778937..52780494	PGSC0003DMG400018093	Fasciclin-like arabinogalactan protein 9
ST4.03ch03	52782806..52785051	PGSC0003DMG400018092	Myb family transcription factor
ST4.03ch03	52790743..52794203	PGSC0003DMG400018149	Iron-sulfer cluster scaffold protein ISU1
ST4.03ch03	52795727..52815228	PGSC0003DMG400018148	ATPANK2 (PANTOTHENATE KINASE 2)
ST4.03ch03	52818309..52821055	PGSC0003DMG400018091	Nucleic acid binding protein
ST4.03ch03	52832337..52834480	PGSC0003DMG400018090	F-box family protein
ST4.03ch03	52904555..52905920	PGSC0003DMG400018147	Gene of unknown function
ST4.03ch03	52906784..52909093	PGSC0003DMG400018146	Pectinesterase
ST4.03ch03	52909271..52910258	PGSC0003DMG400018145	Enzyme inhibitor
ST4.03ch03	52923766..52930958	PGSC0003DMG400018089	Chromosome structural maintenance protein
ST4.03ch03	52940329..52944148	PGSC0003DMG400018088	F-box family protein
ST4.03ch03	52956526..52957985	PGSC0003DMG400018087	Conserved gene of unknown function
ST4.03ch03	52961160..52962359	PGSC0003DMG400018086	Ubiquitin-protein ligase
ST4.03ch03	52965443..52966779	PGSC0003DMG400018085	F-box domain containing protein
ST4.03ch03	52975177..52979518	PGSC0003DMG400018144	Vacuolar protein sorting
ST4.03ch03	52979750..52982411	PGSC0003DMG400018084	Aldose-1-epimerase
ST4.03ch03	53020278..53027861	PGSC0003DMG402018143	ABC transporter A family member 2
ST4.03ch03	53028781..53031719	PGSC0003DMG401018143	Conserved gene of unknown function
ST4.03ch03	53032298..53037916	PGSC0003DMG400018082	Ubiquitin carrier protein
ST4.03ch03	53041644..53045794	PGSC0003DMG400018081	WRKY72
ST4.03ch03	53057787..53061213	PGSC0003DMG400018080	Conserved gene of unknown function
ST4.03ch03	53062734..53066698	PGSC0003DMG400018079	Nuclear ribonuclease Z
ST4.03ch03	53068010..53070815	PGSC0003DMG400018142	Pectate lyase
ST4.03ch03	53072141..53073610	PGSC0003DMG400018078	Ubiquitin-protein ligase
ST4.03ch03	53074920..53076092	PGSC0003DMG400018077	Conserved gene of unknown function
ST4.03ch03	53138385..53139192	PGSC0003DMG400025350	Small heat-shock protein
ST4.03ch03	53140092..53149526	PGSC0003DMG400025349	ATP-dependent Clp protease proteolytic subunit
ST4.03ch03	53153671..53158806	PGSC0003DMG400025348	Quirky
ST4.03ch03	53159296..53160057	PGSC0003DMG400025347	ABR064Wp
ST4.03ch03	53160568..53162079	PGSC0003DMG400025346	Voltage-dependent anion channel
ST4.03ch03	53162164..53165206	PGSC0003DMG400025335	Hypersensitive-induced reaction protein
ST4.03ch03	53171883..53173398	PGSC0003DMG400025345	Conserved gene of unknown function
ST4.03ch03	53173772..53178316	PGSC0003DMG400025334	Nitrate transporter
ST4.03ch03	53184212..53185070	PGSC0003DMG400025344	Gene of unknown function
ST4.03ch03	53190265..53191554	PGSC0003DMG400025333	Short chain alcohol dehydrogenase

ST4.03ch03	53203041..53205307	PGSC0003DMG400025342	Homeobox
ST4.03ch03	53209658..53214943	PGSC0003DMG400025332	Integral membrane HPP family protein
ST4.03ch03	53219916..53220716	PGSC0003DMG400044826	Conserved gene of unknown function
ST4.03ch03	53226137..53226624	PGSC0003DMG400025341	Gene of unknown function
ST4.03ch03	53227052..53228846	PGSC0003DMG400025331	Short chain alcohol dehydrogenase
ST4.03ch03	53228956..53233993	PGSC0003DMG400025330	BRASSINOSTEROID INSENSITIVE 1-associated receptor kinase 1
ST4.03ch03	53235911..53238548	PGSC0003DMG400025339	Nitrate transporter
ST4.03ch03	53243878..53244450	PGSC0003DMG400025338	Gene of unknown function
ST4.03ch03	53260486..53262884	PGSC0003DMG400025337	Nitrate transporter
ST4.03ch03	53269753..53273630	PGSC0003DMG400025336	Nitrate transporter NRT1-5
ST4.03ch03	53276654..53283732	PGSC0003DMG400025328	Plasma membrane ATPase 1
ST4.03ch03	53299059..53299562	PGSC0003DMG400017117	Gene of unknown function
ST4.03ch03	53307178..53312264	PGSC0003DMG400017116	Nodulin family protein
ST4.03ch03	53312698..53316721	PGSC0003DMG400017115	Nodulin family protein
ST4.03ch03	53393151..53400243	PGSC0003DMG400013184	Zinc finger protein
ST4.03ch03	53401591..53404834	PGSC0003DMG400013182	LOB domain-containing protein
ST4.03ch03	53409812..53418049	PGSC0003DMG400013181	Ubiquitin thiolesterase/ zinc ion binding
ST4.03ch03	53421004..53425909	PGSC0003DMG400013180	Pentatricopeptide repeat-containing protein
ST4.03ch03	53433747..53439804	PGSC0003DMG400013183	Calcium-dependent protein kinase 1
ST4.03ch03	53496881..53497354	PGSC0003DMG400024668	Gene of unknown function
ST4.03ch03	53497641..53498274	PGSC0003DMG400024563	Gene of unknown function
ST4.03ch03	53507685..53510025	PGSC0003DMG400024458	HIPL1 protein
ST4.03ch03	53510985..53512068	PGSC0003DMG400024564	Conserved gene of unknown function
ST4.03ch03	53518677..53522587	PGSC0003DMG400024459	HIPL1 protein
ST4.03ch03	53536006..53538645	PGSC0003DMG400024460	TRNA ligase
ST4.03ch03	53538807..53540440	PGSC0003DMG400024566	Conserved gene of unknown function
ST4.03ch03	53540506..53544562	PGSC0003DMG400024567	Galactosyltransferase
ST4.03ch03	53549065..53550718	PGSC0003DMG400024461	Conserved gene of unknown function
ST4.03ch03	53552433..53554202	PGSC0003DMG400024462	Axonemal dynein light chain
ST4.03ch03	53560253..53564066	PGSC0003DMG401024463	DNA binding protein
ST4.03ch03	53562621..53564066	PGSC0003DMG402024463	Gene of unknown function
ST4.03ch03	53565261..53565923	PGSC0003DMG400024568	Rhicadhesin receptor
ST4.03ch03	53567366..53568413	PGSC0003DMG400024569	Rhicadhesin receptor
ST4.03ch03	53569914..53573217	PGSC0003DMG400024570	5-formyltetrahydrofolate cycloligase
ST4.03ch03	53574169..53577852	PGSC0003DMG400024464	Conserved gene of unknown function
ST4.03ch03	53578162..53583986	PGSC0003DMG400024571	Conserved gene of unknown function
ST4.03ch03	53594044..53595185	PGSC0003DMG400041939	Ubiquitin-protein ligase
ST4.03ch03	53602333..53603983	PGSC0003DMG400024572	MYB transcription factor
ST4.03ch03	53618233..53623994	PGSC0003DMG400024573	Pentatricopeptide repeat-containing protein, chloroplastic
ST4.03ch03	53624273..53638935	PGSC0003DMG400024574	Conserved gene of unknown function
ST4.03ch03	53653286..53663670	PGSC0003DMG400024465	Conserved gene of unknown function
ST4.03ch03	53664130..53668654	PGSC0003DMG400024575	Microtubule-associated protein TORTIFOLIA1
ST4.03ch03	53683860..53686179	PGSC0003DMG400024466	ATP-binding cassette transporter
ST4.03ch03	53694121..53695966	PGSC0003DMG400024467	Putative anti-virus transcriptional factor

ST4.03ch03	53699687..53700358	PGSC0003DMG400024576	Gene of unknown function
ST4.03ch03	53703782..53706897	PGSC0003DMG400024577	Actin binding protein
ST4.03ch03	53710389..53712417	PGSC0003DMG400024578	Type-A response regulator
ST4.03ch03	53717816..53719670	PGSC0003DMG400024468	B12D protein
ST4.03ch03	53719600..53725446	PGSC0003DMG400024579	Cell division cycle
ST4.03ch03	53729236..53733274	PGSC0003DMG401024469	Pentatricopeptide repeat-containing protein
ST4.03ch03	53732690..53737067	PGSC0003DMG402024469	E2F
ST4.03ch03	53737614..53742531	PGSC0003DMG400024580	Pyridoxamine 5-phosphate oxidase
ST4.03ch03	53743129..53750825	PGSC0003DMG401024581	Pyridoxamine 5-phosphate oxidase
ST4.03ch03	53751474..53756993	PGSC0003DMG402024581	Mannose-1-phosphate guanyltransferase
ST4.03ch03	53757940..53766241	PGSC0003DMG400024582	Aminoaldehyde dehydrogenase 2
ST4.03ch03	53773207..53773694	PGSC0003DMG400024583	Conserved gene of unknown function
ST4.03ch03	53775263..53778132	PGSC0003DMG400024584	Alpha-n-acetylglucosaminidase
ST4.03ch03	53797414..53798920	PGSC0003DMG400024470	F-box and wd40 domain protein
ST4.03ch03	53799509..53804520	PGSC0003DMG400024585	Methyltransferase gidB
ST4.03ch03	53807761..53808234	PGSC0003DMG400024670	Gene of unknown function
ST4.03ch03	53818291..53820652	PGSC0003DMG400024471	Ubiquitin-protein ligase
ST4.03ch03	53823505..53827469	PGSC0003DMG401024472	Endonuclease III
ST4.03ch03	53828218..53829118	PGSC0003DMG402024472	C2H2L domain class transcription factor
ST4.03ch03	53830836..53833876	PGSC0003DMG401024473	Mannosyl-oligosaccharide 1, 2-alpha-mannosidase IA
ST4.03ch03	53834245..53836806	PGSC0003DMG402024473	Gene of unknown function
ST4.03ch03	53841278..53844424	PGSC0003DMG400024474	Gonadotropin beta chain
ST4.03ch03	53866220..53872744	PGSC0003DMG400024475	Calmodulin binding protein
ST4.03ch03	53878612..53879610	PGSC0003DMG400024476	Heat-shock protein
ST4.03ch03	53883494..53884399	PGSC0003DMG400024671	Gene of unknown function
ST4.03ch03	53891239..53893065	PGSC0003DMG400024477	Calmodulin binding protein
ST4.03ch03	53897293..53901168	PGSC0003DMG400024478	Calmodulin-binding protein
ST4.03ch03	53911132..53916455	PGSC0003DMG400024479	Calmodulin-binding protein
ST4.03ch03	53926690..53931992	PGSC0003DMG401024480	Conserved gene of unknown function
ST4.03ch03	53957004..53960677	PGSC0003DMG402024480	Gene of unknown function
ST4.03ch03	53957004..53961462	PGSC0003DMG400024586	PBng143
ST4.03ch03	53970890..53973411	PGSC0003DMG400024481	Pollen specific LIM domain-containing protein
ST4.03ch03	53973615..53976517	PGSC0003DMG400024482	Pentatricopeptide repeat protein
ST4.03ch03	53980366..53981973	PGSC0003DMG400024587	Conserved gene of unknown function
ST4.03ch03	53992509..53999728	PGSC0003DMG400024588	Permease I
ST4.03ch03	54006159..54007939	PGSC0003DMG400024589	Protein PRD1
ST4.03ch03	54016250..54023228	PGSC0003DMG400024483	Structural constituent of ribosome
ST4.03ch03	54027001..54035855	PGSC0003DMG400024484	BAH domain-containing protein
ST4.03ch03	54027811..54028808	PGSC0003DMG400024673	Gene of unknown function
ST4.03ch03	54036158..54044679	PGSC0003DMG402024590	Rac protein
ST4.03ch03	54042117..54044321	PGSC0003DMG401024590	Rac protein
ST4.03ch03	54051726..54054216	PGSC0003DMG400024485	Serine-threonine protein kinase, plant-type
ST4.03ch03	54057013..54057951	PGSC0003DMG400040994	Ring finger protein
ST4.03ch03	54059590..54060328	PGSC0003DMG400024486	Conserved gene of unknown function

ST4.03ch03	54061000..54066772	PGSC0003DMG400024591	Cyclic nucleotide-gated ion channel 16
ST4.03ch03	54068745..54069866	PGSC0003DMG400024487	RNA binding protein
ST4.03ch03	54072511..54073496	PGSC0003DMG400024488	IS10 transposase
ST4.03ch03	54088348..54093737	PGSC0003DMG400024489	Sugar transporter
ST4.03ch03	54102305..54107157	PGSC0003DMG400024490	Aldehyde dehydrogenase
ST4.03ch03	54110767..54113181	PGSC0003DMG400024491	Spotted leaf protein
ST4.03ch03	54116300..54119783	PGSC0003DMG400024492	Deleted in split hand/splt foot protein 1
ST4.03ch03	54121762..54123763	PGSC0003DMG400024593	Glycosyltransferase UGT90A7
ST4.03ch03	54125552..54128245	PGSC0003DMG400024594	Glycosyltransferase UGT90A7
ST4.03ch03	54135534..54136366	PGSC0003DMG400024595	Zinc finger protein
ST4.03ch03	54139914..54140882	PGSC0003DMG400024493	Seven-transmembrane-domain protein 1
ST4.03ch03	54141229..54146751	PGSC0003DMG400024596	Protein kinase atmrk1
ST4.03ch03	54149532..54150359	PGSC0003DMG400046943	NAC transcription factor
ST4.03ch03	54170568..54171266	PGSC0003DMG400045571	NAC transcription factor
ST4.03ch03	54181378..54182621	PGSC0003DMG400024597	DNA binding protein
ST4.03ch03	54247606..54248172	PGSC0003DMG400024598	DNA binding protein
ST4.03ch03	54259488..54260780	PGSC0003DMG400024599	Polygalacturonase-1 non-catalytic subunit beta
ST4.03ch03	54266590..54268781	PGSC0003DMG400024494	Phosphoglycerate mutase
ST4.03ch03	54281887..54282522	PGSC0003DMG400024495	DNA binding protein
ST4.03ch03	54285706..54286826	PGSC0003DMG400043406	Ubiquitin-protein ligase
ST4.03ch03	54289906..54290359	PGSC0003DMG400024496	Ulp1 protease family, C-terminal catalytic domain containing protein
ST4.03ch03	54304129..54310985	PGSC0003DMG400024497	Zinc finger family protein
ST4.03ch03	54312619..54315441	PGSC0003DMG400024498	UPF0497 membrane protein
ST4.03ch03	54316402..54322812	PGSC0003DMG400024499	Para-hydroxybenzoate--polyprenyltransferase
ST4.03ch03	54329272..54335041	PGSC0003DMG400024500	HT1 protein kinase
ST4.03ch03	54348113..54351370	PGSC0003DMG400024501	Kinesin light chain
ST4.03ch03	54355980..54363363	PGSC0003DMG400024502	AtPex14p
ST4.03ch03	54368432..54374033	PGSC0003DMG400024503	1-deoxy-D-xylulose-5-phosphate reductoisomerase
ST4.03ch03	54398719..54399968	PGSC0003DMG400024504	Conserved gene of unknown function
ST4.03ch03	54417167..54419695	PGSC0003DMG400024600	Conserved gene of unknown function
ST4.03ch03	54440605..54441423	PGSC0003DMG400040550	NAC transcription factor
ST4.03ch03	54445376..54448068	PGSC0003DMG400024675	Gene of unknown function
ST4.03ch03	54460915..54461706	PGSC0003DMG400045663	NAC transcription factor
ST4.03ch03	54462729..54470280	PGSC0003DMG400024601	Dead box ATP-dependent RNA helicase
ST4.03ch03	54472475..54477646	PGSC0003DMG400024505	Chromosome-associated kinesin KIF4A
ST4.03ch03	54480475..54481506	PGSC0003DMG400024602	Conserved gene of unknown function
ST4.03ch03	54482662..54484036	PGSC0003DMG402024603	DR1
ST4.03ch03	54482662..54486225	PGSC0003DMG401024603	TATA-binding protein-associated phosphoprotein
ST4.03ch03	54485667..54486191	PGSC0003DMG400024506	Gene of unknown function
ST4.03ch03	54499470..54500427	PGSC0003DMG400024507	Gene of unknown function
ST4.03ch03	54508886..54509597	PGSC0003DMG400024604	Calmodulin
ST4.03ch03	54514242..54515501	PGSC0003DMG400024605	Ubiquitin-protein ligase
ST4.03ch03	54516634..54516957	PGSC0003DMG400041876	Conserved gene of unknown function
ST4.03ch03	54533650..54534364	PGSC0003DMG400024606	ERF transcription factor

ST4.03ch03	54537065..54540400	PGSC0003DMG400024508	Extracellular Ca2+ sensing receptor
ST4.03ch03	54542358..54546406	PGSC0003DMG401024509	Acetyl-CoA synthetase
ST4.03ch03	54546628..54553861	PGSC0003DMG402024509	Acetyl-CoA synthetase
ST4.03ch03	54554175..54558025	PGSC0003DMG403024509	Cell growth defect factor
ST4.03ch03	54558654..54565189	PGSC0003DMG402024607	Senescence-associated protein
ST4.03ch03	54561847..54562704	PGSC0003DMG400045477	Senescence-associated protein 5
ST4.03ch03	54563683..54567546	PGSC0003DMG401024607	Conserved gene of unknown function
ST4.03ch03	54572440..54573754	PGSC0003DMG400024608	Senescence-associated protein
ST4.03ch03	54580617..54585154	PGSC0003DMG401024510	Enolase
ST4.03ch03	54584634..54585092	PGSC0003DMG402024510	Gene of unknown function
ST4.03ch03	54585371..54590436	PGSC0003DMG400024609	Conserved gene of unknown function
ST4.03ch03	54592800..54599988	PGSC0003DMG400024511	MIS12 homologue
ST4.03ch03	54601440..54604655	PGSC0003DMG402024610	Conserved gene of unknown function
ST4.03ch03	54608969..54610252	PGSC0003DMG400024611	Conserved gene of unknown function
ST4.03ch03	54616709..54617351	PGSC0003DMG403024610	Conserved gene of unknown function
ST4.03ch03	54619356..54620651	PGSC0003DMG401024610	Conserved gene of unknown function
ST4.03ch03	54625568..54632815	PGSC0003DMG400024512	H/ACA ribonucleoprotein complex subunit
ST4.03ch03	54627728..54639244	PGSC0003DMG400024612	Uridine cytidine kinase I
ST4.03ch03	54653788..54658842	PGSC0003DMG400024513	Serine/threonine protein kinase
ST4.03ch03	54656234..54659205	PGSC0003DMG400024613	Protein ASC1
ST4.03ch03	54665923..54666739	PGSC0003DMG400024514	Gene of unknown function
ST4.03ch03	54668202..54668507	PGSC0003DMG400024515	Conserved gene of unknown function
ST4.03ch03	54671247..54671681	PGSC0003DMG400024516	Conserved gene of unknown function
ST4.03ch03	54691474..54694326	PGSC0003DMG401024517	Protease
ST4.03ch03	54696661..54697371	PGSC0003DMG400024518	Conserved gene of unknown function
ST4.03ch03	54698447..54702052	PGSC0003DMG402024517	Pentatricopeptide repeat-containing protein
ST4.03ch03	54709249..54710000	PGSC0003DMG400024676	Conserved gene of unknown function
ST4.03ch03	54710753..54714249	PGSC0003DMG400024519	Pentatricopeptide repeat-containing protein
ST4.03ch03	54714809..54719299	PGSC0003DMG400024520	Conserved gene of unknown function
ST4.03ch03	54720513..54724268	PGSC0003DMG400024614	Zinc finger protein
ST4.03ch03	54724838..54731754	PGSC0003DMG400024615	WD-repeat protein
ST4.03ch03	54742273..54745641	PGSC0003DMG400024521	Gene of unknown function
ST4.03ch03	54747304..54749116	PGSC0003DMG400024522	Conserved gene of unknown function
ST4.03ch03	54758892..54760665	PGSC0003DMG400024616	UDP-glucosyltransferase
ST4.03ch03	54771919..54773675	PGSC0003DMG400024617	UDP-glucosyltransferase
ST4.03ch03	54778153..54779955	PGSC0003DMG400024618	UDP-glucosyltransferase
ST4.03ch03	54796953..54803667	PGSC0003DMG400024523	Transcription factor BIM1
ST4.03ch03	54809796..54813578	PGSC0003DMG400024619	Conserved gene of unknown function
ST4.03ch03	54821626..54822091	PGSC0003DMG401024620	Gene of unknown function
ST4.03ch03	54823953..54825022	PGSC0003DMG402024620	Gene of unknown function
ST4.03ch03	54823953..54829101	PGSC0003DMG403024620	BSD domain containing protein
ST4.03ch03	54830434..54831584	PGSC0003DMG404024620	Conserved gene of unknown function
ST4.03ch03	54835543..54840071	PGSC0003DMG400024524	Microtubule-associated protein
ST4.03ch03	54840182..54841714	PGSC0003DMG400024621	Conserved gene of unknown function

ST4.03ch03	54853249..54860120	PGSC0003DMG400024525	Dead box ATP-dependent RNA helicase
ST4.03ch03	54860582..54863323	PGSC0003DMG400024526	L-asparaginase
ST4.03ch03	54865153..54866555	PGSC0003DMG400024622	Flavonol 4'-sulfotransferase
ST4.03ch03	54867980..54873469	PGSC0003DMG400024623	Glycosyltransferase QUASIMODO1
ST4.03ch03	54874088..54876489	PGSC0003DMG400024624	Conserved gene of unknown function
ST4.03ch03	54879384..54886641	PGSC0003DMG400024625	MADS box protein
ST4.03ch03	54896515..54901975	PGSC0003DMG400024626	MADS-box protein 1
ST4.03ch03	54926596..54932614	PGSC0003DMG400024527	LIGULELESS1 protein
ST4.03ch03	54934288..54936181	PGSC0003DMG400024627	GRP 2
ST4.03ch03	54941445..54943770	PGSC0003DMG400024528	Transposase Tam3
ST4.03ch03	54954975..54955675	PGSC0003DMG400024677	Gene of unknown function
ST4.03ch03	54961544..54963584	PGSC0003DMG400024530	Protein COBRA
ST4.03ch03	54963884..54966403	PGSC0003DMG400024628	COBRA 3
ST4.03ch03	54969629..54970481	PGSC0003DMG400024678	Gene of unknown function
ST4.03ch03	54970872..54972801	PGSC0003DMG400024629	Pentatricopeptide repeat-containing protein
ST4.03ch03	54979843..54986094	PGSC0003DMG400024531	Thylakoid lumenal 25.6 kDa protein, chloroplast
ST4.03ch03	54992291..54994660	PGSC0003DMG400024630	Cytochrome P450
ST4.03ch03	55020280..55031726	PGSC0003DMG400024532	Multidrug resistance protein
ST4.03ch03	55057180..55060002	PGSC0003DMG400024631	SP1L
ST4.03ch03	55066389..55071048	PGSC0003DMG400024534	Conserved gene of unknown function
ST4.03ch03	55080279..55086682	PGSC0003DMG400024535	Conserved gene of unknown function
ST4.03ch03	55087489..55088223	PGSC0003DMG400024679	Calcium ion binding protein
ST4.03ch03	55095032..55097193	PGSC0003DMG400024632	Transcription factor
ST4.03ch03	55106449..55107969	PGSC0003DMG400024633	Conserved gene of unknown function
ST4.03ch03	55108954..55112010	PGSC0003DMG400024536	Mitochondrial import receptor subunit TOM5 homolog
ST4.03ch03	55112112..55114301	PGSC0003DMG400024537	Basic 7S globulin 2 small subunit
ST4.03ch03	55114549..55115862	PGSC0003DMG400024538	Basic 7S globulin 2 small subunit
ST4.03ch03	55116413..55120021	PGSC0003DMG400024634	70 kDa subunit of replication protein A
ST4.03ch03	55120463..55124847	PGSC0003DMG400024635	Conserved gene of unknown function
ST4.03ch03	55126898..55130102	PGSC0003DMG400024539	ATEXO70E2
ST4.03ch03	55132684..55134160	PGSC0003DMG400024636	Presenilin
ST4.03ch03	55137994..55138407	PGSC0003DMG400036289	Gene of unknown function
ST4.03ch03	55140671..55145965	PGSC0003DMG400024637	Conserved gene of unknown function
ST4.03ch03	55148871..55153448	PGSC0003DMG400024638	ATP synthase subunit gamma, mitochondrial
ST4.03ch03	55158832..55159875	PGSC0003DMG400024540	Heat shock protein
ST4.03ch03	55167492..55170026	PGSC0003DMG400024541	Heat shock protein
ST4.03ch03	55170152..55172782	PGSC0003DMG400024639	Histone deacetylase
ST4.03ch03	55187626..55190646	PGSC0003DMG400024640	Zinc ion binding protein
ST4.03ch03	55207465..55207931	PGSC0003DMG400024542	Gene of unknown function
ST4.03ch03	55212848..55216845	PGSC0003DMG400024641	24 kDa seed maturation protein
ST4.03ch03	55220548..55224682	PGSC0003DMG400024642	Glucan endo-1, 3-beta-glucosidase 3
ST4.03ch03	55232191..55236265	PGSC0003DMG400024643	Flavonoid 3'-monooxygenase
ST4.03ch03	55248140..55252302	PGSC0003DMG400024644	101 kDa heat shock protein
ST4.03ch03	55254760..55255117	PGSC0003DMG400024645	Gene of unknown function

ST4.03ch03	55279369..55282340	PGSC0003DMG400024543	Protein tyrosine phosphatase
ST4.03ch03	55316389..55316718	PGSC0003DMG400024646	Expansin
ST4.03ch03	55320358..55324943	PGSC0003DMG400024647	Expansin
ST4.03ch03	55329575..55332586	PGSC0003DMG400024545	Gene of unknown function
ST4.03ch03	55333818..55338034	PGSC0003DMG400024648	Expansin
ST4.03ch03	55343022..55344967	PGSC0003DMG400024546	40S ribosomal protein S19
ST4.03ch03	55347770..55354797	PGSC0003DMG400024547	Diacylglycerol kinase, theta
ST4.03ch03	55351518..55352296	PGSC0003DMG400024682	Gene of unknown function
ST4.03ch03	55359886..55361328	PGSC0003DMG400036966	UDP-glucose 6-dehydrogenase
ST4.03ch03	55361783..55381426	PGSC0003DMG402024649	EMB2733/ESP3 (EMBRYO DEFECTIVE 2733)
ST4.03ch03	55379182..55379793	PGSC0003DMG401024649	EMB2733/ESP3 (EMBRYO DEFECTIVE 2733)
ST4.03ch03	55384580..55386047	PGSC0003DMG400024650	Ubiquitin-protein ligase
ST4.03ch03	55387412..55388225	PGSC0003DMG400024684	Gene of unknown function
ST4.03ch03	55397524..55398549	PGSC0003DMG400024651	Ubiquitin-protein ligase
ST4.03ch03	55408732..55414637	PGSC0003DMG401024548	Leucine-rich repeat containing protein
ST4.03ch03	55413716..55414630	PGSC0003DMG402024548	Gene of unknown function
ST4.03ch03	55415653..55419423	PGSC0003DMG401024549	ATP binding protein
ST4.03ch03	55421643..55425464	PGSC0003DMG402024549	ATP binding protein
ST4.03ch03	55433276..55435278	PGSC0003DMG400024550	ZPT4-3
ST4.03ch03	55439454..55441424	PGSC0003DMG400024551	Conserved gene of unknown function
ST4.03ch03	55443299..55443883	PGSC0003DMG400024652	RING-H2 finger protein ATL1M
ST4.03ch03	55445620..55449745	PGSC0003DMG400024552	Synaptosomal associated protein
ST4.03ch03	55451344..55451992	PGSC0003DMG400024653	Catalytic/ oxidoreductase, acting on NADH or NADPH
ST4.03ch03	55453167..55454136	PGSC0003DMG401024654	Heparanase 1
ST4.03ch03	55453167..55458448	PGSC0003DMG402024654	Heparanase-2
ST4.03ch03	55462578..55470574	PGSC0003DMG400024553	Conserved gene of unknown function
ST4.03ch03	55474349..55475978	PGSC0003DMG400024655	SNF2 domain-containing protein / helicase domain-containing protein / HNH endonuclease domain-containing protein
ST4.03ch03	55495567..55498080	PGSC0003DMG400024554	DNA binding protein
ST4.03ch03	55503952..55508294	PGSC0003DMG400024656	Conserved gene of unknown function
ST4.03ch03	55513446..55517088	PGSC0003DMG400024556	Dimethylaniline monooxygenase
ST4.03ch03	55517808..55520194	PGSC0003DMG400024657	Pentatricopeptide repeat-containing protein
ST4.03ch03	55520373..55524372	PGSC0003DMG400024658	Excision repair cross-complementing 1 ercc1
ST4.03ch03	55524895..55529535	PGSC0003DMG400024659	Conserved gene of unknown function
ST4.03ch03	55540500..55542597	PGSC0003DMG400024660	HCG23534, partial
ST4.03ch03	55544743..55548544	PGSC0003DMG400024661	Brassinosteroid LRR receptor kinase
ST4.03ch03	55559293..55561461	PGSC0003DMG400024662	Myosin heavy chain
ST4.03ch03	55564054..55569528	PGSC0003DMG401024557	Carbamoyl phosphate synthase small subunit
ST4.03ch03	55570479..55572685	PGSC0003DMG402024557	Conserved gene of unknown function
ST4.03ch03	55575040..55577312	PGSC0003DMG400024558	Eukaryotic translation initiation factor 5A-5
ST4.03ch03	55596037..55603079	PGSC0003DMG400024559	ATP binding protein
ST4.03ch03	55615061..55624395	PGSC0003DMG400024560	Formin 20
ST4.03ch03	55627919..55628320	PGSC0003DMG400041803	Gene of unknown function
ST4.03ch03	55631876..55633324	PGSC0003DMG400024664	Conserved gene of unknown function

ST4.03ch03	55641081..55645781	PGSC0003DMG400024561	Ser/Thr protein kinase
ST4.03ch03	55646289..55648550	PGSC0003DMG400024665	Kinase
ST4.03ch03	55666624..55668451	PGSC0003DMG400024562	Conserved gene of unknown function
ST4.03ch03	55668600..55672390	PGSC0003DMG400024667	Glycosyltransferase
ST4.03ch03	55730618..55734374	PGSC0003DMG400019502	Vesicle transport v-SNARE 11
ST4.03ch03	55734633..55741670	PGSC0003DMG400019518	Pseudo response regulator
ST4.03ch03	55745709..55749972	PGSC0003DMG400019519	Exonuclease
ST4.03ch03	55750123..55752218	PGSC0003DMG400019520	Conserved gene of unknown function
ST4.03ch03	55752344..55753969	PGSC0003DMG400019503	Pentatricopeptide repeat-containing protein
ST4.03ch03	55765639..55771810	PGSC0003DMG400019504	Sand family protein
ST4.03ch03	55775640..55782005	PGSC0003DMG400019521	Ribulose-phosphate 3-epimerase, chloroplastic
ST4.03ch03	55783142..55785146	PGSC0003DMG400019505	Alpha-1, 2-fucosyltransferase
ST4.03ch03	55785591..55789344	PGSC0003DMG400019522	Heat shock protein binding protein
ST4.03ch03	55815778..55817714	PGSC0003DMG400019523	NAC domain-containing protein 21/22
ST4.03ch03	55826686..55830767	PGSC0003DMG400019524	YGL010w
ST4.03ch03	55841662..55843149	PGSC0003DMG400019506	Thioredoxin II
ST4.03ch03	55854740..55856726	PGSC0003DMG400019507	Expansin
ST4.03ch03	55862891..55864115	PGSC0003DMG400019508	Chlorophyll a/b binding protein
ST4.03ch03	55865548..55867778	PGSC0003DMG400019525	MADS-box family protein
ST4.03ch03	55869333..55870654	PGSC0003DMG400019526	Zinc finger protein
ST4.03ch03	55872209..55873150	PGSC0003DMG400019527	Calcium ion binding protein
ST4.03ch03	55882564..55885296	PGSC0003DMG400019528	Dof zinc finger protein
ST4.03ch03	55899892..55900645	PGSC0003DMG400019509	Conserved gene of unknown function
ST4.03ch03	55901911..55903596	PGSC0003DMG400019529	Zinc finger protein
ST4.03ch03	55904950..55906678	PGSC0003DMG400019510	Geranylgeranyl diphosphate reductase, chloroplastic
ST4.03ch03	55907758..55912183	PGSC0003DMG400019511	NAD-malate dehydrogenase
ST4.03ch03	55912895..55913779	PGSC0003DMG400019530	Pentatricopeptide repeat-containing protein
ST4.03ch03	55919907..55921627	PGSC0003DMG400019531	EMB514
ST4.03ch03	55924195..55930016	PGSC0003DMG400019512	Membrane associated ring finger 1, 8
ST4.03ch03	55930637..55935866	PGSC0003DMG400019532	Conserved gene of unknown function
ST4.03ch03	55943853..55944385	PGSC0003DMG402019533	Gasa4
ST4.03ch03	55943853..55945734	PGSC0003DMG401019533	Gip1
ST4.03ch03	55959016..55959501	PGSC0003DMG400019534	Transcription factor
ST4.03ch03	55984819..55986615	PGSC0003DMG400019535	MYB domain class transcription factor
ST4.03ch03	55998988..56003542	PGSC0003DMG400019536	Conserved gene of unknown function
ST4.03ch03	56004967..56005756	PGSC0003DMG400019540	Glutathione s-transferase
ST4.03ch03	56013960..56015078	PGSC0003DMG400019537	Glutathione s-transferase
ST4.03ch03	56016933..56022364	PGSC0003DMG400019513	Activating signal cointegrator
ST4.03ch03	56022713..56032180	PGSC0003DMG400019514	ATP binding protein
ST4.03ch03	56036627..56043628	PGSC0003DMG400019515	SET protein, phospatase 2A inhibitor
ST4.03ch03	56045150..56046685	PGSC0003DMG400019516	Chitin-binding lectin
ST4.03ch03	56065422..56065940	PGSC0003DMG400019517	Chitin-binding lectin 1
ST4.03ch03	56080206..56081282	PGSC0003DMG400019538	42kDa chitin-binding protein
ST4.03ch03	56101471..56102139	PGSC0003DMG400019539	42kDa chitin-binding protein

ST4.03ch03	56182447..56183119	PGSC0003DMG400032524	Chitin-binding lectin 1
ST4.03ch03	56197017..56197678	PGSC0003DMG400032527	Chitin-binding lectin 1
ST4.03ch03	56282743..56283138	PGSC0003DMG400000645	42kDa chitin-binding protein
ST4.03ch03	56323681..56324169	PGSC0003DMG400000593	Flavonol synthase/flavanone 3-hydroxylase
ST4.03ch03	56325941..56329622	PGSC0003DMG401000594	Flavonol synthase/flavanone 3-hydroxylase
ST4.03ch03	56330296..56339009	PGSC0003DMG404000594	Flavonol synthase/flavanone 3-hydroxylase
ST4.03ch03	56335439..56336637	PGSC0003DMG400000595	Flavonol synthase/flavanone 3-hydroxylase
ST4.03ch03	56339468..56340945	PGSC0003DMG402000594	Flavonol synthase/flavanone 3-hydroxylase
ST4.03ch03	56342571..56343429	PGSC0003DMG403000594	Flavonol synthase/flavanone 3-hydroxylase
ST4.03ch03	56343918..56345959	PGSC0003DMG400000596	Flavonol synthase/flavanone 3-hydroxylase
ST4.03ch03	56360237..56362098	PGSC0003DMG400000597	Transcription factor
ST4.03ch03	56373271..56379378	PGSC0003DMG400000598	Conserved gene of unknown function
ST4.03ch03	56379966..56383979	PGSC0003DMG400000599	DNA-binding protein
ST4.03ch03	56388027..56390843	PGSC0003DMG400000600	Protein HVA22
ST4.03ch03	56396018..56403865	PGSC0003DMG400000601	ZR1 protein
ST4.03ch03	56404492..56409141	PGSC0003DMG400000627	AtEB1b
ST4.03ch03	56412282..56419831	PGSC0003DMG400000602	Conserved gene of unknown function
ST4.03ch03	56420226..56421126	PGSC0003DMG400000628	Late embryogenesis abundant protein
ST4.03ch03	56421696..56422663	PGSC0003DMG400000603	Conserved gene of unknown function
ST4.03ch03	56426550..56428641	PGSC0003DMG400000604	Zinc finger (CCCH-type) family protein
ST4.03ch03	56430657..56432021	PGSC0003DMG400038576	Conserved gene of unknown function
ST4.03ch03	56433707..56436222	PGSC0003DMG400000605	DNA binding protein
ST4.03ch03	56436555..56437339	PGSC0003DMG402000629	Stigma-specific protein Stig1
ST4.03ch03	56436747..56437316	PGSC0003DMG401000629	Stigma-specific protein Stig1
ST4.03ch03	56437399..56442899	PGSC0003DMG402000630	Racemase and epimerase, acting on amino acids and derivatives
ST4.03ch03	56441852..56442933	PGSC0003DMG401000630	Conserved gene of unknown function
ST4.03ch03	56445081..56446592	PGSC0003DMG400000606	Ubiquitin-protein ligase
ST4.03ch03	56453876..56454894	PGSC0003DMG400000607	Conserved gene of unknown function
ST4.03ch03	56461474..56466007	PGSC0003DMG400000608	Polygalacturonase
ST4.03ch03	56476942..56478370	PGSC0003DMG400000631	Lactoylglutathione lyase
ST4.03ch03	56487086..56490055	PGSC0003DMG400000611	Pentatricopeptide repeat-containing protein
ST4.03ch03	56490832..56494056	PGSC0003DMG400000612	AP2 domain-containing protein AP29
ST4.03ch03	56494225..56498356	PGSC0003DMG400000632	Pentatricopeptide repeat-containing protein
ST4.03ch03	56499164..56501938	PGSC0003DMG400000613	Nucleic acid binding protein
ST4.03ch03	56502402..56502712	PGSC0003DMG400000633	Glycoside hydrolase, family 28
ST4.03ch03	56506793..56508408	PGSC0003DMG400000634	CAPIP2
ST4.03ch03	56508806..56513240	PGSC0003DMG400000635	Longin
ST4.03ch03	56522242..56522732	PGSC0003DMG400000646	RNase H family protein
ST4.03ch03	56522932..56524108	PGSC0003DMG400000636	AP2 transcription factor
ST4.03ch03	56535683..56540302	PGSC0003DMG400000614	Phospholipase PLDa1
ST4.03ch03	56543497..56544650	PGSC0003DMG400000615	Conserved gene of unknown function
ST4.03ch03	56548515..56554248	PGSC0003DMG400000616	Conserved gene of unknown function
ST4.03ch03	56558525..56562589	PGSC0003DMG401000617	Conserved gene of unknown function
ST4.03ch03	56561181..56562589	PGSC0003DMG402000617	Gene of unknown function

ST4.03ch03	56568452..56570701	PGSC0003DMG400000618	Conserved gene of unknown function
ST4.03ch03	56571862..56574223	PGSC0003DMG400000619	Nucleosome-binding protein
ST4.03ch03	56574769..56575915	PGSC0003DMG400000620	Stellacyanin CASLP1
ST4.03ch03	56577846..56578716	PGSC0003DMG400000621	NtEIG-A1 protein
ST4.03ch03	56586492..56587506	PGSC0003DMG400000637	GTP binding protein
ST4.03ch03	56593561..56594955	PGSC0003DMG400000638	Histidine-containing phosphotransfer protein
ST4.03ch03	56602125..56605038	PGSC0003DMG400000639	Delta 9 desaturase
ST4.03ch03	56613085..56615641	PGSC0003DMG400000640	Conserved gene of unknown function
ST4.03ch03	56635358..56640706	PGSC0003DMG400000642	ATP binding protein
ST4.03ch03	56647451..56648584	PGSC0003DMG400000622	Conserved gene of unknown function
ST4.03ch03	56649989..56653346	PGSC0003DMG400000623	Conserved gene of unknown function
ST4.03ch03	56665415..56666306	PGSC0003DMG400000643	Conserved gene of unknown function
ST4.03ch03	56666693..56672595	PGSC0003DMG400000625	Signal recognition particle 54 kDa protein 1
ST4.03ch03	56672676..56681324	PGSC0003DMG400000644	Phox domain-containing protein
ST4.03ch03	56738617..56742872	PGSC0003DMG400019816	Conserved gene of unknown function
ST4.03ch03	56743356..56745821	PGSC0003DMG400019817	Pentatricopeptide repeat-containing protein
ST4.03ch03	56745855..56746399	PGSC0003DMG400019818	Conserved gene of unknown function
ST4.03ch03	56746680..56752798	PGSC0003DMG400019821	Cyclic nucleotide-gated ion channel
ST4.03ch03	56755917..56757865	PGSC0003DMG400019822	N-acetyltransferase
ST4.03ch03	56764519..56767403	PGSC0003DMG400019823	Conserved gene of unknown function
ST4.03ch03	56769711..56776369	PGSC0003DMG400019819	DNA-3-methyladenine glycosylase
ST4.03ch03	56777004..56780104	PGSC0003DMG400019824	JA-induced WRKY protein
ST4.03ch03	56814215..56816945	PGSC0003DMG400019825	Cinnamoyl CoA reductase
ST4.03ch03	56823535..56833474	PGSC0003DMG401019826	Nucleic acid binding protein
ST4.03ch03	56833367..56833831	PGSC0003DMG402019826	Gene of unknown function
ST4.03ch03	56839370..56843865	PGSC0003DMG400019827	Nucleic acid binding protein
ST4.03ch03	56848518..56849783	PGSC0003DMG400019828	Conserved gene of unknown function
ST4.03ch03	56862676..56870271	PGSC0003DMG400019829	Nucleic acid binding protein
ST4.03ch03	56877725..56879270	PGSC0003DMG400019830	Conserved gene of unknown function
ST4.03ch03	56881487..56883357	PGSC0003DMG400019831	Gene of unknown function
ST4.03ch03	56886710..56887250	PGSC0003DMG400019832	Transport protein
ST4.03ch03	56892955..56894017	PGSC0003DMG400014119	SFT2 domain containing 2
ST4.03ch03	56920573..56925568	PGSC0003DMG400014120	Stromal cell-derived factor 2
ST4.03ch03	56926874..56928354	PGSC0003DMG400014121	Ccr4-associated factor
ST4.03ch03	56931977..56937779	PGSC0003DMG402014189	XH/XS domain-containing protein
ST4.03ch03	56944480..56947012	PGSC0003DMG401014189	Conserved gene of unknown function
ST4.03ch03	56950476..56950820	PGSC0003DMG400014122	Conserved gene of unknown function
ST4.03ch03	56959060..56962258	PGSC0003DMG400014123	Aquaporin, MIP family, NIP subfamily
ST4.03ch03	56961255..56963431	PGSC0003DMG400014190	Conserved gene of unknown function
ST4.03ch03	56963696..56967940	PGSC0003DMG400014191	60S ribosomal protein L7
ST4.03ch03	56983543..56984571	PGSC0003DMG400014124	Zinc finger protein
ST4.03ch03	56988976..56991846	PGSC0003DMG400014192	Mitochondrial glycoprotein family protein
ST4.03ch03	56992178..56998247	PGSC0003DMG400014193	Conserved gene of unknown function
ST4.03ch03	57001818..57003706	PGSC0003DMG400014125	Conserved gene of unknown function

ST4.03ch03	57004942..57008028	PGSC0003DMG400014126	Conserved gene of unknown function
ST4.03ch03	57019240..57025146	PGSC0003DMG400014194	Defective in cullin neddylation protein
ST4.03ch03	57026302..57034757	PGSC0003DMG400014195	Nucleoporin
ST4.03ch03	57039987..57041187	PGSC0003DMG400014196	Ethylene response factor
ST4.03ch03	57042084..57043284	PGSC0003DMG400014127	DNA binding protein
ST4.03ch03	57050846..57054023	PGSC0003DMG400014128	Plasma membrane proton ATPase
ST4.03ch03	57054280..57061338	PGSC0003DMG403014197	Serine/threonine-protein kinase PBS1
ST4.03ch03	57055529..57056170	PGSC0003DMG402014197	Conserved gene of unknown function
ST4.03ch03	57057636..57060917	PGSC0003DMG401014197	Serine/threonine-protein kinase PBS1
ST4.03ch03	57066724..57068528	PGSC0003DMG400014129	Vegetative cell wall protein gp1
ST4.03ch03	57074634..57076797	PGSC0003DMG400014130	F-box/LRR-repeat protein
ST4.03ch03	57078465..57081047	PGSC0003DMG400014131	Leucine-rich repeat family protein
ST4.03ch03	57078465..57084441	PGSC0003DMG400014198	Conserved gene of unknown function
ST4.03ch03	57110681..57112638	PGSC0003DMG400014199	Nodulin
ST4.03ch03	57113395..57115435	PGSC0003DMG400014200	Flotillin-1
ST4.03ch03	57117491..57118750	PGSC0003DMG400014132	Conserved gene of unknown function
ST4.03ch03	57123593..57125091	PGSC0003DMG400014133	F-box family protein
ST4.03ch03	57134091..57138611	PGSC0003DMG400014134	Conserved gene of unknown function
ST4.03ch03	57155346..57155951	PGSC0003DMG400046334	Conserved gene of unknown function
ST4.03ch03	57159221..57164698	PGSC0003DMG400014135	Nodulin
ST4.03ch03	57164954..57166123	PGSC0003DMG400014201	Jp18
ST4.03ch03	57166577..57170997	PGSC0003DMG400014202	50S ribosomal protein L3
ST4.03ch03	57171533..57179260	PGSC0003DMG400014136	Prolyl-tRNA synthetase
ST4.03ch03	57178648..57179211	PGSC0003DMG400034349	Jp18
ST4.03ch03	57184598..57186764	PGSC0003DMG400014203	Amino acid transporter
ST4.03ch03	57191604..57192393	PGSC0003DMG400014204	Transcription factor TSRF1
ST4.03ch03	57209218..57211637	PGSC0003DMG400014137	Protein TOPLESS
ST4.03ch03	57212055..57221082	PGSC0003DMG401014138	WD-repeat protein
ST4.03ch03	57222159..57229453	PGSC0003DMG402014138	WD-repeat protein
ST4.03ch03	57232401..57241608	PGSC0003DMG401014139	WD-repeat protein
ST4.03ch03	57237010..57238131	PGSC0003DMG402014139	Beta transducin
ST4.03ch03	57239271..57241608	PGSC0003DMG403014139	WD-repeat protein
ST4.03ch03	57270931..57278996	PGSC0003DMG400014140	Prli-interacting factor l
ST4.03ch03	57279271..57282389	PGSC0003DMG400014141	DEAD-box protein abstrakt
ST4.03ch03	57283003..57283575	PGSC0003DMG400014142	Allergen Pru p 1.06B
ST4.03ch03	57285513..57286258	PGSC0003DMG400014143	Major allergen Pru ar
ST4.03ch03	57286634..57287334	PGSC0003DMG400014206	Conserved gene of unknown function
ST4.03ch03	57287784..57292017	PGSC0003DMG400014207	Calcineurin
ST4.03ch03	57293005..57297878	PGSC0003DMG400014208	Inorganic pyrophosphatase
ST4.03ch03	57309534..57312556	PGSC0003DMG400014144	Glycerol kinase

Table S4: Overview about the different mutation types and locations present in the genes under the DRYM QTL on LG3 (A)

ID	Name	synonymous	missense	downstream	upstream	intron	splice	stop	start	initiator	3'UTR	5'UTR	Summe
PGSC0003DMG401015193	Malate synthase	1	0	0	0	0	0	0	0	0	0	0	1
PGSC0003DMG400024516	Conserved gene of unknown function	0	1	0	0	0	0	0	0	0	0	0	1
PGSC0003DMG400019540	Glutathione s-transferase	0	0	0	0	0	0	0	0	0	0	1	1
PGSC0003DMG402019826	Gene of unknown function	0	1	0	0	0	0	0	0	0	0	0	1
PGSC0003DMG400000633	Glycoside hydrolase, family 28	0	0	0	0	0	0	0	0	0	1	0	1
PGSC0003DMG400018093	Fasciclin-like arabinogalactan protein 9	0	1	0	0	0	0	0	0	0	0	0	1
PGSC0003DMG400015258	DNA-directed RNA polymerase	1	0	0	0	0	0	0	0	0	0	0	1
PGSC0003DMG400019832	Transport protein	0	0	0	0	0	0	0	0	0	1	0	1
PGSC0003DMG400024583	Conserved gene of unknown function	0	1	0	0	0	0	0	0	0	0	0	1
PGSC0003DMG400025636	Receptor kinase	1	0	0	0	0	0	0	0	0	0	0	1
PGSC0003DMG400026498	Gene of unknown function	0	1	0	0	0	0	0	0	0	0	0	1
PGSC0003DMG400015294	Gene of unknown function	0	0	0	0	0	0	0	0	0	1	0	1
PGSC0003DMG400046943	NAC transcription factor	1	0	0	0	0	0	0	0	0	0	0	1
PGSC0003DMG402024548	Gene of unknown function	0	0	0	0	0	0	0	0	0	0	1	1
PGSC0003DMG400024668	Gene of unknown function	0	0	0	0	0	0	0	0	0	0	1	1
PGSC0003DMG400024651	Ubiquitin-protein ligase	0	1	0	0	0	0	0	0	0	0	0	1
PGSC0003DMG401015259	Gene of unknown function	0	0	0	0	0	0	0	0	0	1	0	1
PGSC0003DMG400019509	Conserved gene of unknown function	0	0	0	0	0	0	0	0	0	1	0	1
PGSC0003DMG400004345	Gene of unknown function	0	0	0	0	0	0	0	0	0	1	0	1
PGSC0003DMG402004331	Conserved gene of unknown function	0	1	0	0	0	0	0	0	0	0	0	1
PGSC0003DMG400010135	Ethylene-responsive element-binding family protein	0	1	0	0	0	0	0	0	0	0	0	1
PGSC0003DMG400019529	Zinc finger protein	1	0	0	0	0	0	0	0	0	0	0	1
PGSC0003DMG400031060	Gene of unknown function	0	0	0	0	0	0	0	0	0	1	0	1
PGSC0003DMG401018159	Conserved gene of unknown function	0	0	0	0	0	0	0	0	0	1	0	1
PGSC0003DMG400034620	Pectinesterase inhibitor	0	1	0	0	0	0	0	0	0	0	0	1
PGSC0003DMG400031059	Conserved gene of unknown function	0	0	0	0	1	0	0	0	0	0	0	1
PGSC0003DMG400018199	Cytochrome P450 71A4	1	0	0	0	0	0	0	0	0	0	0	1
PGSC0003DMG400034349	Jp18	0	1	0	0	0	0	0	0	0	0	0	1
PGSC0003DMG400025347	ABR064Wp	1	0	0	0	0	0	0	0	0	0	1	2
PGSC0003DMG400018197	Cytochrome P450 71A4	0	2	0	0	0	0	0	0	0	0	0	2
PGSC0003DMG400015302	Conserved gene of unknown function	2	0	0	0	0	0	0	0	0	0	0	2
PGSC0003DMG400019526	Zinc finger protein	1	1	0	0	0	0	0	0	0	0	0	2
PGSC0003DMG400018121	Conserved gene of unknown function	0	0	0	0	0	0	0	0	0	2	0	2
PGSC0003DMG400024468	B12D protein	0	0	2	0	0	0	0	0	0	0	0	2
PGSC0003DMG400015289	Proteinase inhibitor type-2 CEVI57	1	0	0	0	0	0	1	0	0	0	0	2
PGSC0003DMG400015214	Conserved gene of unknown function	1	0	0	0	0	0	0	0	0	1	0	2
PGSC0003DMG402019533	Gasa4	1	0	0	0	0	0	0	0	0	0	1	2
PGSC0003DMG400018145	Enzyme inhibitor	1	1	0	0	0	0	0	0	0	0	0	2
PGSC0003DMG400018188	Ubiquitin-protein ligase	1	1	0	0	0	0	0	0	0	0	0	2
PGSC0003DMG402031045	Argonaute 1	0	1	0	0	0	0	0	0	0	0	1	2
PGSC0003DMG400024616	UDP-glucosyltransferase	1	1	0	0	0	0	0	0	0	0	0	2
PGSC0003DMG400025333	Short chain alcohol dehydrogenase	0	0	0	0	0	0	0	0	0	2	0	2

PGSC0003DMG402000594	Flavonol synthase/flavanone 3-hydroxylase	0	0	0	0	0	0	0	0	0	2	0	2
PGSC0003DMG400024646	Expansin	2	0	0	0	0	0	0	0	0	0	0	2
PGSC0003DMG400000645	42kDa chitin-binding protein	0	2	0	0	0	0	0	0	0	0	0	2
PGSC0003DMG400000628	Late embryogenesis abundant protein	1	1	0	0	0	0	0	0	0	0	0	2
PGSC0003DMG400019534	Transcription factor	1	1	0	0	0	0	0	0	0	0	0	2
PGSC0003DMG400046334	Conserved gene of unknown function	2	0	0	0	0	0	0	0	0	0	0	2
PGSC0003DMG400025633	F-box protein interaction domain containing protein	2	1	0	0	0	0	0	0	0	0	0	3
PGSC0003DMG400014121	Ccr4-associated factor	0	0	0	0	0	0	0	0	0	0	3	3
PGSC0003DMG400036289	Gene of unknown function	0	3	0	0	0	0	0	0	0	0	0	3
PGSC0003DMG400015260	CONSTANS interacting protein 2a	1	2	0	0	0	0	0	0	0	0	0	3
PGSC0003DMG401024654	Heparanase 1	0	3	0	0	0	0	0	0	0	0	0	3
PGSC0003DMG400009512	Kunitz-type proteinase inhibitor	1	2	0	0	0	0	0	0	0	0	0	3
PGSC0003DMG400015292	ATP binding protein	0	1	0	0	0	0	0	0	0	0	2	3
PGSC0003DMG400024496	Ulp1 protease family, C-terminal catalytic domain containing p	1	1	0	0	0	1	0	0	0	0	0	3
PGSC0003DMG400018132	Cytochrome P450 71A2	0	1	0	2	0	0	0	0	0	0	0	3
PGSC0003DMG402024607	Senescence-associated protein	0	0	0	3	0	0	0	0	0	0	0	3
PGSC0003DMG400005554	Cytochrome P450 71A4	0	2	0	0	0	0	1	0	0	0	0	3
PGSC0003DMG400024645	Gene of unknown function	0	1	0	0	0	0	0	0	0	2	0	3
PGSC0003DMG400024670	Gene of unknown function	0	2	0	0	0	0	0	0	0	1	0	3
PGSC0003DMG400019863	RNase H family protein	1	1	0	0	0	0	0	0	0	1	0	3
PGSC0003DMG400019508	Chlorophyll a/b binding protein	0	1	0	0	0	1	0	0	0	1	0	3
PGSC0003DMG400037864	Raffionse synthase 2	0	3	0	0	0	0	0	0	0	0	0	3
PGSC0003DMG400024515	Conserved gene of unknown function	2	1	0	0	0	0	0	0	0	0	0	3
PGSC0003DMG400017117	Gene of unknown function	1	2	0	0	0	0	0	0	0	0	0	3
PGSC0003DMG400014142	Allergen Pru p 1.06B	2	1	0	0	0	0	0	0	0	0	0	3
PGSC0003DMG400018155	Ubiquitin-protein ligase	0	1	0	0	0	0	0	0	0	1	1	3
PGSC0003DMG400000593	Flavonol synthase/flavanone 3-hydroxylase	2	1	0	0	0	0	0	0	0	0	0	3
PGSC0003DMG401010182	Receptor kinase	0	0	0	0	0	0	0	0	0	2	1	3
PGSC0003DMG400014124	Zinc finger protein	2	1	0	0	0	0	0	0	0	0	0	3
PGSC0003DMG400024598	DNA binding protein	0	0	0	0	3	0	0	0	0	0	0	3
PGSC0003DMG400019506	Thioredoxin II	0	0	0	0	0	0	0	0	0	0	3	3
PGSC0003DMG400044826	Conserved gene of unknown function	1	2	0	0	0	0	0	0	0	0	0	3
PGSC0003DMG400037011	Homeobox 9	0	2	0	1	0	0	0	0	0	0	0	3
PGSC0003DMG400045366	Conserved gene of unknown function	1	2	0	0	0	0	0	0	0	0	0	3
PGSC0003DMG400015255	DELLA protein RGL1	2	1	0	0	0	0	0	0	0	0	0	3
PGSC0003DMG400000621	NtEIG-A1 protein	0	2	0	0	0	0	0	0	0	1	0	3
PGSC0003DMG400034803	Gene of unknown function	0	3	0	0	0	0	0	0	0	0	0	3
PGSC0003DMG400019831	Gene of unknown function	0	0	0	0	0	0	0	0	0	3	1	4
PGSC0003DMG400018180	Ribosomal protein L27a	3	0	0	0	0	0	0	0	0	1	0	4
PGSC0003DMG400010178	F-box family protein	3	1	0	0	0	0	0	0	0	0	0	4
PGSC0003DMG400015261	Heat shock protein binding protein	1	3	0	0	0	0	0	0	0	0	0	4
PGSC0003DMG400000595	Flavonol synthase/flavanone 3-hydroxylase	1	3	0	0	0	0	0	0	0	0	0	4
PGSC0003DMG400024653	Catalytic/ oxidoreductase, acting on NADH or NADPH	2	1	0	0	0	0	0	0	0	1	0	4
PGSC0003DMG401024620	Gene of unknown function	1	1	0	0	0	0	0	0	0	2	0	4

PGSC0003DMG400024671	Gene of unknown function	0	1	0	0	0	0	0	0	0	1	2	4
PGSC0003DMG400015204	Conserved gene of unknown function	0	0	0	0	0	0	0	0	0	4	0	4
PGSC0003DMG400045663	NAC transcription factor	0	4	0	0	0	0	0	0	0	0	0	4
PGSC0003DMG400040957	Pectinesterase inhibitor	0	4	0	0	0	0	0	0	0	0	0	4
PGSC0003DMG402015193	Malate synthase, glyoxysomal	3	1	0	0	0	0	0	0	0	0	0	4
PGSC0003DMG400010136	Stigma expressed protein	2	2	0	0	0	0	0	0	0	0	0	4
PGSC0003DMG400015301	Conserved gene of unknown function	0	0	0	0	0	0	0	0	0	4	0	4
PGSC0003DMG400024679	Calcium ion binding protein	0	1	0	0	0	0	1	0	0	2	0	4
PGSC0003DMG400024665	Kinase	3	1	0	0	0	0	0	0	0	0	0	4
PGSC0003DMG400039762	Proteinase inhibitor type-2 CEVI57	0	4	0	0	0	0	0	0	0	0	0	4
PGSC0003DMG400018176	Oleosin	1	2	0	0	0	0	0	0	0	0	1	4
PGSC0003DMG400010131	Nb cell deth marker	3	1	0	0	0	0	0	0	0	0	0	4
PGSC0003DMG401024610	Conserved gene of unknown function	1	3	0	0	0	0	0	0	0	0	0	4
PGSC0003DMG400024657	Pentatricopeptide repeat-containing protein	0	1	0	0	0	0	0	0	0	4	0	5
PGSC0003DMG400019503	Pentatricopeptide repeat-containing protein	0	4	0	0	0	0	0	0	0	0	1	5
PGSC0003DMG400033694	Zinc finger homeodomain protein SZF-HD1	2	2	0	0	0	0	0	0	0	1	0	5
PGSC0003DMG401015201	Receptor expression-enhancing protein	2	2	0	1	0	0	0	0	0	0	0	5
PGSC0003DMG400014137	Protein TOPLESS	1	0	0	4	0	0	0	0	0	0	0	5
PGSC0003DMG400015174	DUF26 domain-containing protein 2	3	2	0	0	0	0	0	0	0	0	0	5
PGSC0003DMG400039752	Exocyst complex component	4	1	0	0	0	0	0	0	0	0	0	5
PGSC0003DMG400010149	Conserved gene of unknown function	2	2	0	0	0	0	0	0	0	1	0	5
PGSC0003DMG400015206	Conserved gene of unknown function	1	4	0	0	0	0	0	0	0	0	0	5
PGSC0003DMG400031746	Gene of unknown function	0	2	0	0	0	0	0	0	0	0	3	5
PGSC0003DMG400026495	Gene of unknown function	1	2	1	0	0	0	0	0	0	1	0	5
PGSC0003DMG400019830	Conserved gene of unknown function	0	1	3	0	0	0	0	0	0	0	1	5
PGSC0003DMG401000594	Flavonol synthase/flavanone 3-hydroxylase	1	2	0	1	0	1	0	0	0	0	0	5
PGSC0003DMG400025338	Gene of unknown function	2	3	0	0	0	0	0	0	0	0	0	5
PGSC0003DMG400024506	Gene of unknown function	0	1	0	0	0	1	0	0	0	0	3	5
PGSC0003DMG400018133	Cytochrome P450 71A4	0	1	0	0	0	0	0	0	0	0	4	5
PGSC0003DMG400018170	Conserved gene of unknown function	4	1	0	0	0	0	0	0	0	0	0	5
PGSC0003DMG400024617	UDP-glucosyltransferase	0	2	0	0	0	0	0	0	0	1	2	5
PGSC0003DMG400015184	Cytochrome P450	5	0	0	0	0	0	0	0	0	0	0	5
PGSC0003DMG400014122	Conserved gene of unknown function	1	4	0	0	0	0	0	0	0	0	0	5
PGSC0003DMG402014197	Conserved gene of unknown function	2	3	0	0	0	0	0	0	0	0	0	5
PGSC0003DMG402024463	Gene of unknown function	0	0	0	0	0	0	0	0	0	0	5	5
PGSC0003DMG400024488	IS10 transposase	2	2	0	0	0	0	0	0	0	0	1	5
PGSC0003DMG400015186	Pentatricopeptide repeat-containing protein, chloroplastic	2	4	0	0	0	0	0	0	0	0	0	6
PGSC0003DMG400018207	Gene of unknown function	0	2	0	0	0	0	0	0	0	4	0	6
PGSC0003DMG400042642	Conserved gene of unknown function	0	4	1	0	0	0	1	0	0	0	0	6
PGSC0003DMG400024673	Gene of unknown function	3	1	0	0	0	0	0	0	0	2	0	6
PGSC0003DMG400041803	Gene of unknown function	3	3	0	0	0	0	0	0	0	0	0	6
PGSC0003DMG400024597	DNA binding protein	1	2	0	0	1	2	0	0	0	0	0	6
PGSC0003DMG400010183	Receptor kinase	3	2	0	1	0	0	0	0	0	0	0	6
PGSC0003DMG403004334	Gene of unknown function	0	0	0	0	0	0	0	0	0	5	1	6

PGSC0003DMG400000646	RNase H family protein	2	4	0	0	0	0	0	0	0	0	0	6
PGSC0003DMG402024510	Gene of unknown function	1	1	0	0	0	0	0	0	0	3	1	6
PGSC0003DMG400019538	42kDa chitin-binding protein	3	3	0	0	0	0	0	0	0	0	0	6
PGSC0003DMG400000615	Conserved gene of unknown function	0	3	0	0	0	1	0	0	0	0	2	6
PGSC0003DMG400004548	Proteinase inhibitor type-2 CEVI57	1	3	0	2	0	0	0	0	0	0	0	6
PGSC0003DMG400024493	Seven-transmembrane-domain protein 1	2	0	0	4	0	0	0	0	0	0	0	6
PGSC0003DMG400019537	Glutathione s-transferase	3	1	0	0	0	0	0	0	0	2	0	6
PGSC0003DMG400015267	Kunitz-type protease inhibitor	0	3	0	0	0	0	0	0	0	3	0	6
PGSC0003DMG400024542	Gene of unknown function	1	1	0	0	0	2	0	0	0	2	0	6
PGSC0003DMG400018147	Gene of unknown function	0	3	0	0	0	0	0	0	0	3	0	6
PGSC0003DMG400024655	SNF2 domain-containing protein / helicase domain-containing	0	3	0	0	0	0	0	0	0	3	0	6
PGSC0003DMG400018189	Pectinesterase inhibitor	1	3	0	0	0	0	0	0	0	1	1	6
PGSC0003DMG400024622	Flavonol 4'-sulfotransferase	1	0	0	0	0	0	0	0	0	5	0	6
PGSC0003DMG400015293	Gene of unknown function	0	2	0	0	0	0	0	0	0	3	1	6
PGSC0003DMG400004547	Proteinase inhibitor type-2 P303.51	1	1	0	0	0	0	0	0	0	4	0	6
PGSC0003DMG400019817	Pentatricopeptide repeat-containing protein	1	4	0	0	0	0	0	0	0	0	1	6
PGSC0003DMG400024678	Gene of unknown function	0	1	0	0	0	0	1	0	0	4	0	6
PGSC0003DMG400000637	GTP binding protein	2	1	0	0	2	0	0	0	0	1	0	6
PGSC0003DMG400012021	Armadillo/beta-catenin repeat family protein	3	2	0	0	0	0	0	0	0	2	0	7
PGSC0003DMG400018125	Gene of unknown function	1	4	0	0	0	0	0	0	0	0	2	7
PGSC0003DMG400024606	ERF transcription factor	1	2	0	0	0	0	0	0	0	3	1	7
PGSC0003DMG400015286	Miraculin	2	5	0	0	0	0	0	0	0	0	0	7
PGSC0003DMG401024649	EMB2733/ESP3 (EMBRYO DEFECTIVE 2733)	0	2	2	0	2	1	0	0	0	0	0	7
PGSC0003DMG400012024	O-methyltransferase	1	1	0	0	0	0	0	0	0	5	0	7
PGSC0003DMG400024602	Conserved gene of unknown function	4	2	0	0	0	0	0	0	0	0	1	7
PGSC0003DMG400010159	Conserved gene of unknown function	1	0	0	0	0	0	0	0	0	4	2	7
PGSC0003DMG401000629	Stigma-specific protein Stig1	3	3	0	0	0	0	1	0	0	0	0	7
PGSC0003DMG400018196	CYP71AU1	3	4	0	0	0	0	0	0	0	0	0	7
PGSC0003DMG400019818	Conserved gene of unknown function	1	3	2	0	0	1	0	0	0	0	0	7
PGSC0003DMG400010148	Miraculin	2	1	0	4	0	0	0	0	0	0	0	7
PGSC0003DMG400024650	Ubiquitin-protein ligase	2	5	0	0	0	0	0	0	0	0	0	7
PGSC0003DMG400024618	UDP-glucosyltransferase	3	1	0	0	0	0	0	0	0	1	2	7
PGSC0003DMG400015303	Conserved gene of unknown function	0	0	0	0	0	0	0	0	0	0	7	7
PGSC0003DMG400014201	Jp18	2	1	0	0	0	0	0	0	0	2	2	7
PGSC0003DMG400019511	NAD-malate dehydrogenase	1	0	6	0	0	0	0	0	0	0	0	7
PGSC0003DMG400037301	NB-ARC domain containing protein	1	5	0	0	0	0	1	0	0	0	0	7
PGSC0003DMG400014204	Transcription factor TSRF1	2	3	0	0	0	0	0	0	0	0	2	7
PGSC0003DMG404024620	Conserved gene of unknown function	2	5	0	0	0	0	0	0	0	0	0	7
PGSC0003DMG400040550	NAC transcription factor	2	5	0	0	0	0	0	0	0	0	0	7
PGSC0003DMG400010189	Gene of unknown function	2	4	0	0	0	0	0	0	0	1	0	7
PGSC0003DMG400018206	Conserved gene of unknown function	3	0	0	0	0	0	0	0	0	0	4	7
PGSC0003DMG400000603	Conserved gene of unknown function	2	3	0	1	0	0	0	0	0	1	0	7
PGSC0003DMG401031741	UPA16	1	1	0	0	5	1	0	0	0	0	0	8
PGSC0003DMG400015287	Proteinase inhibitor type-2 CEVI57	3	3	0	1	0	0	0	0	0	1	0	8

PGSC0003DMG400019535	MYB domain class transcription factor	3	1	0	0	0	0	0	0	0	2	2	8
PGSC0003DMG400015222	Transposase	2	5	1	0	0	0	0	0	0	0	0	8
PGSC0003DMG400014203	Amino acid transporter	5	1	0	0	0	0	0	0	0	1	1	8
PGSC0003DMG400010130	Conserved gene of unknown function	0	0	0	0	3	0	0	0	0	4	1	8
PGSC0003DMG400014132	Conserved gene of unknown function	1	2	0	0	0	0	0	0	0	5	0	8
PGSC0003DMG400024530	Protein COBRA	2	3	0	0	0	0	0	0	0	1	2	8
PGSC0003DMG400024518	Conserved gene of unknown function	2	5	0	0	0	0	1	0	0	0	0	8
PGSC0003DMG400019531	EMB514	1	3	0	4	0	0	0	0	0	0	0	8
PGSC0003DMG400024507	Gene of unknown function	0	0	0	0	0	0	0	0	0	7	1	8
PGSC0003DMG400010141	Stigma expressed protein	4	4	0	0	0	0	0	0	0	0	0	8
PGSC0003DMG403024610	Conserved gene of unknown function	2	2	2	0	0	0	0	0	0	2	0	8
PGSC0003DMG400014129	Vegetative cell wall protein gp1	5	3	0	0	0	0	0	0	0	0	0	8
PGSC0003DMG400009513	Aspartic protease inhibitor 5	2	6	0	0	0	0	0	0	0	0	0	8
PGSC0003DMG400043406	Ubiquitin-protein ligase	3	3	0	2	0	0	0	0	0	0	0	8
PGSC0003DMG400024605	Ubiquitin-protein ligase	5	3	0	0	0	0	0	0	0	0	0	8
PGSC0003DMG400010177	Pentatricopeptide	3	5	0	0	0	0	0	0	0	0	0	8
PGSC0003DMG400000631	Lactoylglutathione lyase	0	0	0	0	2	0	0	0	0	5	1	8
PGSC0003DMG400014143	Major allergen Pru ar	3	3	1	0	0	0	0	0	0	0	1	8
PGSC0003DMG400015306	Gene of unknown function	3	2	0	0	0	0	0	0	0	1	2	8
PGSC0003DMG401015159	Eukaryotic translation initiation factor 2c	3	0	0	0	4	1	0	0	0	0	0	8
PGSC0003DMG400019510	Geranylgeranyl diphosphate reductase, chloroplastic	8	0	0	0	0	0	0	0	0	0	0	8
PGSC0003DMG400015219	Miraculin	1	0	0	0	0	0	0	0	0	7	0	8
PGSC0003DMG400026482	Protein HVA22	0	3	0	0	5	0	0	0	0	0	0	8
PGSC0003DMG402015175	Gene of unknown function	2	2	0	0	0	0	0	1	0	0	3	8
PGSC0003DMG400033682	Potassium channel SKOR	0	0	0	0	6	0	0	0	0	3	0	9
PGSC0003DMG400024568	Rhicadhesin receptor	5	4	0	0	0	0	0	0	0	0	0	9
PGSC0003DMG400024462	Axonemal dynein light chain	2	6	0	0	0	0	0	0	0	1	0	9
PGSC0003DMG400024682	Gene of unknown function	1	0	0	0	0	0	0	0	0	1	7	9
PGSC0003DMG400018124	Gene of unknown function	1	1	0	0	0	0	0	0	0	5	2	9
PGSC0003DMG400024677	Gene of unknown function	1	3	0	0	0	0	1	0	0	3	1	9
PGSC0003DMG400025350	Small heat-shock protein	0	9	0	0	0	0	0	0	0	0	0	9
PGSC0003DMG400014130	F-box/LRR-repeat protein	5	2	0	0	0	0	0	0	0	2	0	9
PGSC0003DMG400010147	Kunitz-type enzyme inhibitor S9C11	2	3	0	0	0	0	0	0	0	3	1	9
PGSC0003DMG402000629	Stigma-specific protein Stig1	3	3	0	0	0	0	1	0	0	0	2	9
PGSC0003DMG402015201	Receptor expression-enhancing protein	0	0	0	9	0	0	0	0	0	0	0	9
PGSC0003DMG400019825	Cinnamoyl CoA reductase	0	1	0	0	7	0	0	0	0	0	1	9
PGSC0003DMG400019530	Pentatricopeptide repeat-containing protein	1	2	0	0	0	0	0	1	0	5	0	9
PGSC0003DMG400018087	Conserved gene of unknown function	3	3	0	0	0	1	0	0	0	2	0	9
PGSC0003DMG400024550	ZPT4-3	4	1	0	0	0	0	0	0	0	3	1	9
PGSC0003DMG400004330	Ulp1 protease family, C-terminal catalytic domain containing p	0	2	4	0	0	0	0	0	0	0	3	9
PGSC0003DMG400015173	ATP binding protein	6	3	0	0	0	0	0	0	0	0	0	9
PGSC0003DMG400000634	CAPIP2	1	1	0	0	0	1	0	0	0	3	3	9
PGSC0003DMG400010170	Miraculin	0	0	6	0	0	1	0	0	0	2	0	9
PGSC0003DMG401010181	Pentatricopeptide repeat domain containing protein	4	3	0	0	0	0	0	0	0	2	0	9

PGSC0003DMG401019860	Alpha-2, 8-sialyltransferase 8b	1	3	0	0	0	0	0	0	0	0	5	9
PGSC0003DMG400039636	Ccaat-binding transcription factor	3	5	0	0	0	0	1	0	0	0	0	9
PGSC0003DMG400041876	Conserved gene of unknown function	2	6	0	0	0	0	1	0	0	0	0	9
PGSC0003DMG400040994	Ring finger protein	4	5	0	0	0	0	0	0	0	0	0	9
PGSC0003DMG400000613	Nucleic acid binding protein	0	0	0	6	0	0	0	0	0	3	0	9
PGSC0003DMG400015269	GRP 2	8	0	2	0	0	0	0	0	0	0	0	10
PGSC0003DMG400019527	Calcium ion binding protein	1	1	0	0	0	0	0	0	0	0	8	10
PGSC0003DMG400014200	Flotillin-1	2	4	0	1	0	0	0	0	0	3	0	10
PGSC0003DMG400018134	Cytochrome P450 71A4	2	5	3	0	0	0	0	0	0	0	0	10
PGSC0003DMG400015203	Protein transport protein SEC61 subunit gamma	0	0	9	0	0	0	0	0	0	1	0	10
PGSC0003DMG400014206	Conserved gene of unknown function	4	2	0	0	0	1	1	0	0	0	2	10
PGSC0003DMG400019536	Conserved gene of unknown function	1	2	4	0	0	0	0	0	0	2	1	10
PGSC0003DMG400010155	Gene of unknown function	1	1	0	0	0	0	0	0	0	8	0	10
PGSC0003DMG400000622	Conserved gene of unknown function	2	2	0	6	0	0	0	0	0	0	0	10
PGSC0003DMG400031046	Sucrose synthase	0	1	0	0	0	0	0	0	0	9	0	10
PGSC0003DMG400015290	Proteinase inhibitor type-2 CEVI57	3	1	0	0	0	1	0	0	0	5	0	10
PGSC0003DMG400024495	DNA binding protein	6	4	0	0	0	0	0	0	0	0	0	10
PGSC0003DMG400018186	Conserved gene of unknown function	4	1	0	0	0	0	0	0	0	2	3	10
PGSC0003DMG400004335	MTN3 homolog	0	1	0	0	9	0	0	0	0	0	0	10
PGSC0003DMG400019816	Conserved gene of unknown function	3	2	0	5	0	0	0	0	0	0	0	10
PGSC0003DMG400024563	Gene of unknown function	4	4	0	0	0	1	1	0	0	0	0	10
PGSC0003DMG400019517	Chitin-binding lectin 1	2	8	0	0	0	0	0	0	0	0	0	10
PGSC0003DMG400024452	Pyridoxal-dependent decarboxylase, C-terminal sheet domain	4	6	0	0	0	0	0	0	0	0	0	10
PGSC0003DMG400018156	Ubiquitin-protein ligase	4	1	0	0	0	0	0	0	0	2	3	10
PGSC0003DMG400024600	Conserved gene of unknown function	2	2	0	0	0	0	0	0	0	2	4	10
PGSC0003DMG400018146	Pectinesterase	1	0	0	0	0	0	0	0	0	9	0	10
PGSC0003DMG400036966	UDP-glucose 6-dehydrogenase	6	4	0	0	0	0	0	0	0	0	0	10
PGSC0003DMG400024504	Conserved gene of unknown function	5	2	0	0	0	0	0	0	0	3	0	10
PGSC0003DMG400033695	DC1 domain containing protein	0	2	0	0	0	0	0	0	0	3	5	10
PGSC0003DMG400014128	Plasma membrane proton ATPase	5	0	2	0	0	1	0	0	0	3	0	11
PGSC0003DMG400024604	Calmodulin	7	2	0	0	0	0	0	0	0	0	2	11
PGSC0003DMG400024486	Conserved gene of unknown function	1	3	0	0	0	0	0	0	0	3	4	11
PGSC0003DMG400018085	F-box domain containing protein	0	6	0	0	0	0	0	0	0	4	1	11
PGSC0003DMG400018102	Calmodulin-binding protein	5	5	0	0	0	0	0	0	0	1	0	11
PGSC0003DMG400015227	Conserved gene of unknown function	0	0	0	11	0	0	0	0	0	0	0	11
PGSC0003DMG400014199	Nodulin	4	4	3	0	0	0	0	0	0	0	0	11
PGSC0003DMG400024540	Heat shock protein	1	9	0	0	1	0	0	0	0	0	0	11
PGSC0003DMG400024519	Pentatricopeptide repeat-containing protein	0	0	0	0	0	0	0	0	0	11	0	11
PGSC0003DMG400015282	F-box protein interaction domain containing protein	1	10	0	0	0	0	0	0	0	0	0	11
PGSC0003DMG400000604	Zinc finger (CCCH-type) family protein	0	2	0	9	0	0	0	0	0	0	0	11
PGSC0003DMG400019505	Alpha-1, 2-fucosyltransferase	4	6	0	1	0	0	0	0	0	0	0	11
PGSC0003DMG400024661	Brassinosteroid LRR receptor kinase	7	2	0	0	2	0	0	0	0	0	0	11
PGSC0003DMG401031052	Conserved gene of unknown function	1	2	0	0	0	0	0	0	0	4	4	11
PGSC0003DMG400012023	O-methyltransferase	4	6	0	0	1	0	0	0	0	0	0	11

PGSC0003DMG400015272	DNA binding protein	1	3	7	0	0	0	0	0	0	0	0	11
PGSC0003DMG400019822	N-acetyltransferase	2	2	0	7	0	0	0	0	0	0	0	11
PGSC0003DMG400015178	Heat shock protein	1	2	0	0	0	0	0	0	0	5	3	11
PGSC0003DMG400025341	Gene of unknown function	1	4	6	0	0	0	0	0	0	0	0	11
PGSC0003DMG400024467	Putative anti-virus transcriptional factor	3	4	0	0	1	0	0	0	0	0	4	12
PGSC0003DMG400014133	F-box family protein	1	7	0	0	0	0	0	0	0	4	0	12
PGSC0003DMG400000623	Conserved gene of unknown function	1	2	7	0	0	0	0	0	0	1	1	12
PGSC0003DMG400018123	Gene of unknown function	3	2	0	0	0	0	0	0	0	3	4	12
PGSC0003DMG400000618	Conserved gene of unknown function	1	1	0	4	0	0	0	0	0	4	2	12
PGSC0003DMG400000607	Conserved gene of unknown function	4	3	0	0	0	0	0	0	0	2	3	12
PGSC0003DMG400012022	MdSFBB3-alpha protein	3	5	0	0	0	0	0	0	0	4	0	12
PGSC0003DMG400004338	Mitochondrial import inner membrane translocase subunit Tir	0	0	12	0	0	0	0	0	0	0	0	12
PGSC0003DMG401014189	Conserved gene of unknown function	0	0	0	12	0	0	0	0	0	0	0	12
PGSC0003DMG400010146	Kunitz-type tuber invertase inhibitor	3	9	0	0	0	0	0	0	0	0	0	12
PGSC0003DMG400024514	Gene of unknown function	2	0	0	0	0	0	0	0	0	9	1	12
PGSC0003DMG400018112	Protein LATERAL ORGAN BOUNDARIES	3	1	0	0	8	0	0	0	0	0	0	12
PGSC0003DMG400031035	Conserved gene of unknown function	1	6	0	5	0	0	0	0	0	0	0	12
PGSC0003DMG400010172	Gene of unknown function	1	2	0	1	0	0	0	0	0	1	7	12
PGSC0003DMG400014190	Conserved gene of unknown function	0	0	7	0	0	0	0	0	0	4	1	12
PGSC0003DMG400026476	Transporter	2	1	0	0	8	0	0	0	0	0	1	12
PGSC0003DMG400009514	Kunitz-type protease inhibitor	3	2	0	0	0	0	0	0	0	7	0	12
PGSC0003DMG400024627	GRP 2	6	2	1	0	0	0	0	0	0	3	0	12
PGSC0003DMG400019513	Activating signal cointegrator	1	2	0	8	0	0	0	0	0	1	0	12
PGSC0003DMG400018098	Conserved gene of unknown function	2	4	0	0	0	0	0	0	0	5	2	13
PGSC0003DMG400025345	Conserved gene of unknown function	7	1	0	1	0	0	0	1	0	0	3	13
PGSC0003DMG400024656	Conserved gene of unknown function	3	3	0	0	0	0	0	0	0	5	2	13
PGSC0003DMG402014139	Beta transducin	0	1	11	0	0	0	0	0	0	0	1	13
PGSC0003DMG400019516	Chitin-binding lectin	0	5	0	0	0	0	0	0	0	0	8	13
PGSC0003DMG400018162	Trithorax	6	4	0	0	0	0	0	0	0	2	1	13
PGSC0003DMG402024620	Gene of unknown function	1	2	0	0	0	0	1	0	0	5	4	13
PGSC0003DMG400031731	Feruloyl transferase	1	1	0	7	0	0	0	0	0	4	0	13
PGSC0003DMG400015230	Pectate lyase	6	0	0	0	4	0	0	0	0	2	1	13
PGSC0003DMG400000638	Histidine-containing phosphotransfer protein	1	2	0	0	10	0	0	0	0	0	0	13
PGSC0003DMG400024546	40S ribosomal protein S19	1	1	0	11	0	0	0	0	0	0	0	13
PGSC0003DMG400024572	MYB transcription factor	6	2	0	0	0	1	0	0	0	3	1	13
PGSC0003DMG400026484	Gene of unknown function	6	7	0	0	0	0	0	0	0	0	0	13
PGSC0003DMG401015244	Conserved gene of unknown function	0	1	0	7	0	0	0	0	0	5	0	13
PGSC0003DMG400025331	Short chain alcohol dehydrogenase	2	4	0	0	0	0	0	0	0	6	1	13
PGSC0003DMG400015237	Purine permease	5	3	0	0	0	0	0	0	0	4	1	13
PGSC0003DMG400015149	Cytochrome P450 71A4	6	5	0	2	0	0	0	0	0	0	0	13
PGSC0003DMG400024564	Conserved gene of unknown function	5	3	0	0	0	0	0	0	0	5	0	13
PGSC0003DMG400024494	Phosphoglycerate mutase	6	1	0	0	1	0	0	0	0	4	1	13
PGSC0003DMG401014197	Serine/threonine-protein kinase PBS1	2	2	0	8	0	1	0	0	0	0	0	13
PGSC0003DMG400018179	Armadillo/beta-catenin repeat family protein	5	4	0	0	0	0	0	0	0	3	1	13

PGSC0003DMG400018166	Transporter	1	1	0	9	0	1	0	0	0	2	0	14
PGSC0003DMG400024466	ATP-binding cassette transporter	9	2	0	0	0	0	0	0	0	2	1	14
PGSC0003DMG401000630	Conserved gene of unknown function	4	1	0	3	0	1	0	0	0	0	5	14
PGSC0003DMG402024472	C2H2L domain class transcription factor	8	4	0	0	0	0	0	0	0	2	0	14
PGSC0003DMG400024460	TRNA ligase	1	0	0	4	0	0	0	0	0	4	5	14
PGSC0003DMG400015164	Conserved gene of unknown function	8	1	0	0	0	0	0	0	0	1	4	14
PGSC0003DMG400024664	Conserved gene of unknown function	5	3	0	0	0	0	0	0	0	6	0	14
PGSC0003DMG400000606	Ubiquitin-protein ligase	6	5	0	0	0	0	0	0	0	3	0	14
PGSC0003DMG400025342	Homeobox	5	2	0	0	0	0	0	0	0	1	6	14
PGSC0003DMG400026481	Inositol polyphosphate kinase	7	3	0	0	0	0	0	0	0	2	2	14
PGSC0003DMG400018169	Cyclin D2.1 protein	0	2	0	0	0	0	0	0	0	8	4	14
PGSC0003DMG400024526	L-asparaginase	3	2	0	9	0	0	0	0	0	0	0	14
PGSC0003DMG400010154	Conserved gene of unknown function	3	3	0	0	0	0	0	0	0	6	2	14
PGSC0003DMG400024587	Conserved gene of unknown function	1	1	4	0	0	0	0	0	0	6	2	14
PGSC0003DMG400031049	Nucleic acid binding protein	4	3	0	7	0	0	0	0	0	0	0	14
PGSC0003DMG400015242	Cytochrome P450	10	3	0	0	0	0	0	0	0	1	0	14
PGSC0003DMG400038576	Conserved gene of unknown function	2	4	0	8	0	0	0	0	0	0	0	14
PGSC0003DMG400018088	F-box family protein	1	3	0	3	0	0	0	0	0	3	4	14
PGSC0003DMG400000597	Transcription factor	4	3	0	0	0	0	0	0	0	3	4	14
PGSC0003DMG400025630	Conserved gene of unknown function	2	0	0	8	0	0	0	0	0	5	0	15
PGSC0003DMG400024630	Cytochrome P450	6	6	0	0	1	0	0	0	0	1	1	15
PGSC0003DMG400018150	Phosphate/phosphoenolpyruvate translocator	3	0	7	0	0	1	0	0	0	4	0	15
PGSC0003DMG402004334	Pyruvate dehydrogenase E1 component subunit beta	2	0	0	0	0	0	0	0	0	13	0	15
PGSC0003DMG400037684	Double-stranded RNA binding protein	0	8	0	0	4	3	0	0	0	0	0	15
PGSC0003DMG400031737	Conserved gene of unknown function	1	0	7	0	0	0	0	0	1	4	2	15
PGSC0003DMG400018135	Cytochrome P450 71A4	5	8	0	1	0	0	0	0	0	1	0	15
PGSC0003DMG400000605	DNA binding protein	5	6	0	4	0	0	0	0	0	0	0	15
PGSC0003DMG400026477	Transparent testa glabra 1	5	3	0	0	0	0	0	0	0	3	4	15
PGSC0003DMG400015236	Gene of unknown function	0	1	0	14	0	0	0	0	0	0	0	15
PGSC0003DMG400010152	Integral membrane protein	1	0	3	8	0	0	0	0	0	0	3	15
PGSC0003DMG400018105	Ubiquitin-protein ligase	7	8	0	0	0	0	0	0	0	0	0	15
PGSC0003DMG400019861	BHLH transcription factor Upa20	1	2	0	0	7	0	0	0	0	3	2	15
PGSC0003DMG400018198	70 kDa subunit of replication protein A	2	7	4	0	0	2	0	0	0	0	0	15
PGSC0003DMG400018128	Protein phosphatase 2c	9	1	0	0	0	0	0	0	0	4	1	15
PGSC0003DMG400024554	DNA binding protein	2	2	0	0	7	0	0	0	0	3	1	15
PGSC0003DMG400018097	Conserved gene of unknown function	4	0	10	0	0	1	0	0	0	0	0	15
PGSC0003DMG400000600	Protein HVA22	2	0	3	0	5	0	0	0	0	4	1	15
PGSC0003DMG400024556	Dimethylaniline monooxygenase	2	2	0	4	0	0	0	0	0	1	6	15
PGSC0003DMG400015220	Cytochrome P450 71A4	4	6	0	0	4	1	0	0	0	0	0	15
PGSC0003DMG400015156	Conserved gene of unknown function	7	1	0	0	3	1	0	0	0	2	1	15
PGSC0003DMG400015275	Aquaporin, MIP family, TIP subfamily	3	0	4	0	0	0	0	0	0	2	6	15
PGSC0003DMG400024619	Conserved gene of unknown function	2	1	0	0	6	0	0	0	0	2	4	15
PGSC0003DMG400018141	Cytochrome P450 71A4	8	3	0	0	0	0	0	0	0	3	1	15
PGSC0003DMG400024658	Excision repair cross-complementing 1 ercc1	2	2	0	3	0	2	0	0	0	2	5	16

PGSC0003DMG400024522	Conserved gene of unknown function	0	8	8	0	0	0	0	0	0	0	0	16
PGSC0003DMG400000611	Pentatricopeptide repeat-containing protein	3	2	0	0	0	0	0	0	0	10	1	16
PGSC0003DMG400024611	Conserved gene of unknown function	7	5	0	1	3	0	0	0	0	0	0	16
PGSC0003DMG400036195	Gene of unknown function	2	5	0	9	0	0	0	0	0	0	0	16
PGSC0003DMG400018095	Conserved gene of unknown function	3	1	0	0	5	0	0	0	0	7	0	16
PGSC0003DMG403015244	Brg-1 associated factor	0	5	0	7	0	0	0	0	0	1	3	16
PGSC0003DMG402024517	Pentatricopeptide repeat-containing protein	6	7	0	0	0	0	0	0	0	0	3	16
PGSC0003DMG400024470	F-box and wd40 domain protein	2	9	0	0	0	0	0	0	0	2	3	16
PGSC0003DMG400015217	F-box protein interaction domain containing protein	6	9	0	1	0	0	0	0	0	0	0	16
PGSC0003DMG400019520	Conserved gene of unknown function	1	3	0	0	0	0	0	0	0	6	6	16
PGSC0003DMG400019525	MADS-box family protein	3	2	11	0	0	0	0	0	0	0	0	16
PGSC0003DMG400034404	Gene of unknown function	6	10	0	0	0	0	0	0	0	0	0	16
PGSC0003DMG400026480	Mitotic-spindle organizing protein 1B	0	3	0	0	0	2	0	0	0	3	8	16
PGSC0003DMG400019859	Nuclear transcription factor, X-box binding	4	3	7	0	0	2	0	0	0	0	0	16
PGSC0003DMG400024471	Ubiquitin-protein ligase	6	3	0	0	0	0	0	0	0	3	4	16
PGSC0003DMG400024476	Heat-shock protein	1	3	0	0	0	0	0	0	0	12	0	16
PGSC0003DMG400015228	Peptide methionine sulfoxide reductase	4	1	0	0	0	0	1	0	0	9	1	16
PGSC0003DMG400024562	Conserved gene of unknown function	2	4	0	0	0	0	0	0	0	9	2	17
PGSC0003DMG401024557	Carbamoyl phosphate synthase small subunit	3	1	0	10	0	1	0	0	0	2	0	17
PGSC0003DMG400024501	Kinesin light chain	13	1	0	0	0	0	0	0	0	3	0	17
PGSC0003DMG400000614	Phospholipase PLDa1	4	2	0	0	0	0	0	0	0	8	3	17
PGSC0003DMG400018086	Ubiquitin-protein ligase	8	9	0	0	0	0	0	0	0	0	0	17
PGSC0003DMG400018092	Myb family transcription factor	5	4	0	8	0	0	0	0	0	0	0	17
PGSC0003DMG400019823	Conserved gene of unknown function	1	2	0	7	0	1	0	0	0	6	0	17
PGSC0003DMG400015254	Nucleoside diphosphate kinase 2, chloroplastic	5	0	12	0	0	0	0	0	0	0	0	17
PGSC0003DMG400024599	Polygalacturonase-1 non-catalytic subunit beta	12	5	0	0	0	0	0	0	0	0	0	17
PGSC0003DMG402031037	Kinase family protein	0	1	14	0	2	0	0	0	0	0	0	17
PGSC0003DMG400004337	MTN3	1	2	4	0	1	0	0	0	0	9	0	17
PGSC0003DMG400026479	Metal ion binding protein	2	1	0	0	6	1	0	0	0	2	5	17
PGSC0003DMG400018091	Nucleic acid binding protein	3	0	7	0	5	0	0	0	0	2	0	17
PGSC0003DMG400018078	Ubiquitin-protein ligase	4	11	0	0	0	0	0	0	0	1	1	17
PGSC0003DMG402024473	Gene of unknown function	2	1	0	9	0	1	0	0	0	1	3	17
PGSC0003DMG400015213	Conserved gene of unknown function	3	2	0	11	0	0	0	0	0	1	0	17
PGSC0003DMG400031732	ATP binding	3	0	7	0	3	1	0	0	0	3	0	17
PGSC0003DMG400025628	Pyridoxal-dependent decarboxylase, C-terminal sheet domain	8	5	0	0	0	0	0	0	0	0	4	17
PGSC0003DMG400024498	UPF0497 membrane protein	1	0	0	0	0	1	0	0	0	15	1	18
PGSC0003DMG400015196	Conserved gene of unknown function	12	4	0	0	0	0	1	0	0	1	0	18
PGSC0003DMG400018077	Conserved gene of unknown function	6	11	0	0	0	0	1	0	0	0	0	18
PGSC0003DMG400015160	KDEL-tailed cysteine endopeptidase	4	1	0	0	0	0	1	0	0	11	1	18
PGSC0003DMG400018161	Ubiquitin carrier protein	1	0	6	8	0	1	0	1	0	1	0	18
PGSC0003DMG400000619	Nucleosome-binding protein	1	7	0	1	0	0	0	0	0	3	6	18
PGSC0003DMG400000627	AtEB1b	1	1	9	6	0	0	0	0	0	0	1	18
PGSC0003DMG402031741	UPA16	3	3	0	0	7	0	0	0	0	4	1	18
PGSC0003DMG400019507	Expansin	5	0	0	3	0	0	0	0	0	7	3	18

PGSC0003DMG400024589	Protein PRD1	5	5	0	0	7	0	0	0	0	0	1	18
PGSC0003DMG400031742	UPA16	3	2	0	0	13	0	0	0	0	0	0	18
PGSC0003DMG400014208	Inorganic pyrophosphatase	7	0	0	8	0	0	0	0	0	2	1	18
PGSC0003DMG400033684	Cyc07	4	0	9	0	0	2	0	0	0	2	1	18
PGSC0003DMG400024538	Basic 7S globulin 2 small subunit	5	13	0	0	0	0	0	0	0	0	0	18
PGSC0003DMG400018163	Serine-threonine protein kinase, plant-type	7	6	0	0	0	0	0	0	0	3	2	18
PGSC0003DMG402000617	Gene of unknown function	2	1	0	0	0	0	0	0	0	14	1	18
PGSC0003DMG400033696	MAPKK	8	4	0	0	0	0	0	0	0	0	6	18
PGSC0003DMG400024628	COBRA 3	1	3	0	14	0	0	0	0	0	0	0	18
PGSC0003DMG400015150	Cytochrome P450 71A4	11	7	1	0	0	0	0	0	0	0	0	19
PGSC0003DMG401019533	Gip1	1	0	17	0	0	0	0	0	0	0	1	19
PGSC0003DMG400024577	Actin binding protein	9	8	0	1	0	0	0	0	0	0	1	19
PGSC0003DMG400015176	Gene of unknown function	0	0	0	0	0	0	0	0	0	19	0	19
PGSC0003DMG402024469	E2F	2	0	8	6	0	0	0	0	0	3	0	19
PGSC0003DMG400024487	RNA binding protein	9	3	0	3	0	0	0	0	0	3	1	19
PGSC0003DMG400000639	Delta 9 desaturase	2	2	0	0	11	0	0	0	0	2	2	19
PGSC0003DMG400031042	Glutamate decarboxylase isoform1	3	1	0	7	0	0	0	0	0	2	6	19
PGSC0003DMG400018165	Heme-binding protein	0	1	17	0	0	0	0	0	0	0	1	19
PGSC0003DMG400024614	Zinc finger protein	5	3	9	0	0	0	0	0	0	1	1	19
PGSC0003DMG400033691	50S ribosomal protein L29, chloroplastic	0	1	15	1	0	0	0	0	0	2	0	19
PGSC0003DMG400025635	Leucine rich repeat containing protein	0	1	0	4	0	1	0	0	0	0	13	19
PGSC0003DMG401018164	EMB2759	0	1	0	17	1	0	0	0	0	0	0	19
PGSC0003DMG400000620	Stellacyanin CASLP1	2	8	0	4	0	0	0	0	0	3	2	19
PGSC0003DMG400024636	Presenilin	9	4	0	0	0	0	0	0	0	2	5	20
PGSC0003DMG400024558	Eukaryotic translation initiation factor 5A-5	0	0	13	0	0	0	0	0	0	6	1	20
PGSC0003DMG400018094	Ribosomal protein L41	1	0	0	8	5	0	0	0	0	6	0	20
PGSC0003DMG400014191	60S ribosomal protein L7	1	0	0	18	0	0	0	0	0	1	0	20
PGSC0003DMG400019523	NAC domain-containing protein 21/22	4	1	0	0	1	0	0	0	0	14	0	20
PGSC0003DMG400014131	Leucine-rich repeat family protein	2	6	0	0	0	0	2	0	0	10	0	20
PGSC0003DMG400037321	UPF0497 membrane protein	1	9	0	0	10	0	0	0	0	0	0	20
PGSC0003DMG400018120	TRANSPARENT TESTA 12 protein	0	5	0	15	0	0	0	0	0	0	0	20
PGSC0003DMG400014125	Conserved gene of unknown function	3	2	0	7	0	1	0	0	0	1	6	20
PGSC0003DMG400000636	AP2 transcription factor	10	1	6	0	0	2	0	0	0	0	1	20
PGSC0003DMG402031036	Aldo-keto reductase	8	6	0	6	0	0	0	0	0	0	0	20
PGSC0003DMG400015183	Cytochrome P450	12	3	0	0	0	0	0	1	0	1	3	20
PGSC0003DMG400018149	Iron-sulfer cluster scaffold protein ISU1	0	0	0	16	0	0	0	0	0	3	1	20
PGSC0003DMG400033690	Conserved gene of unknown function	1	0	0	15	0	0	0	0	0	4	0	20
PGSC0003DMG400018084	Aldose-1-epimerase	2	4	13	0	0	1	0	0	0	0	0	20
PGSC0003DMG400024491	Spotted leaf protein	8	10	0	0	0	0	0	0	0	3	0	21
PGSC0003DMG400000625	Signal recognition particle 54 kDa protein 1	3	1	3	11	0	0	0	1	0	0	2	21
PGSC0003DMG400015277	Conserved gene of unknown function	3	3	0	0	0	1	0	0	0	12	2	21
PGSC0003DMG400024621	Conserved gene of unknown function	5	3	7	0	0	0	0	0	0	5	1	21
PGSC0003DMG400015181	Nucleic acid binding protein	2	1	13	0	0	1	0	0	0	2	2	21
PGSC0003DMG400000643	Conserved gene of unknown function	3	3	0	0	0	0	0	0	0	15	0	21

PGSC0003DMG402015202	Reverse transcriptase family member	0	0	0	0	0	0	0	0	0	11	10	21
PGSC0003DMG400018136	Cytochrome P450 71A2	8	1	0	0	0	0	0	0	0	7	6	22
PGSC0003DMG400024485	Serine-threonine protein kinase, plant-type	5	8	0	9	0	0	0	0	0	0	0	22
PGSC0003DMG400024566	Conserved gene of unknown function	4	1	0	0	0	0	0	0	0	12	5	22
PGSC0003DMG400018114	ATFRO8/FRO8	7	8	0	1	0	0	1	0	0	4	1	22
PGSC0003DMG400031738	UPA16	8	4	0	0	10	0	0	0	0	0	0	22
PGSC0003DMG400024482	Pentatricopeptide repeat protein	6	4	0	0	0	0	1	0	0	8	3	22
PGSC0003DMG400019528	Dof zinc finger protein	6	5	0	5	0	1	0	0	0	3	2	22
PGSC0003DMG400018131	CYP71AU1	15	3	0	0	0	0	0	0	0	2	2	22
PGSC0003DMG400018140	Cytochrome P450 71A4	12	7	0	3	0	0	0	0	0	0	0	22
PGSC0003DMG401031037	ATP binding protein	8	8	0	6	0	0	0	0	0	0	0	22
PGSC0003DMG402018164	Protein MODIFIER OF SNC1 1	3	6	0	0	7	1	0	0	0	4	1	22
PGSC0003DMG400034843	Gene of unknown function	7	13	0	0	0	0	2	0	0	0	0	22
PGSC0003DMG400024644	101 kDa heat shock protein	7	2	8	0	0	0	0	0	0	4	1	22
PGSC0003DMG400015185	Cytochrome P450	9	6	0	0	0	0	0	0	0	6	1	22
PGSC0003DMG400018182	Ring finger protein	6	3	0	0	0	0	0	0	0	7	6	22
PGSC0003DMG400018190	Elongation factor TuA, chloroplastic	12	4	0	0	0	0	0	0	0	4	2	22
PGSC0003DMG400018090	F-box family protein	9	2	0	0	6	0	1	0	0	4	0	22
PGSC0003DMG400015262	Heat shock protein binding protein	3	19	0	0	0	0	0	0	0	0	0	22
PGSC0003DMG400015299	Gene of unknown function	0	3	0	0	0	0	0	0	0	19	0	22
PGSC0003DMG400015151	Cytochrome P450	5	3	0	0	12	0	0	0	0	2	0	22
PGSC0003DMG400024629	Pentatricopeptide repeat-containing protein	13	9	0	0	0	0	0	0	0	0	0	22
PGSC0003DMG400045825	Gene of unknown function	10	13	0	0	0	0	0	0	0	0	0	23
PGSC0003DMG400018113	R2r3-myb transcription factor	3	8	2	0	0	0	0	0	0	10	0	23
PGSC0003DMG400018118	MATE efflux family protein	8	2	11	0	0	2	0	0	0	0	0	23
PGSC0003DMG402024557	Conserved gene of unknown function	2	0	0	12	0	0	0	0	0	1	8	23
PGSC0003DMG400015249	3-oxoacyl-(Acyl-carrier protein) reductase	0	5	15	0	0	3	0	0	0	0	0	23
PGSC0003DMG400024464	Conserved gene of unknown function	9	5	0	1	0	0	0	0	0	6	2	23
PGSC0003DMG400015271	P40	7	3	0	13	0	0	0	0	0	0	0	23
PGSC0003DMG400015194	Malate synthase	4	6	5	0	4	0	0	0	0	3	1	23
PGSC0003DMG400025634	F-box protein interaction domain containing protein	9	12	0	2	0	0	0	0	0	0	0	23
PGSC0003DMG400015274	DNA binding protein	5	8	0	8	0	0	0	0	0	0	2	23
PGSC0003DMG400024527	LIGULELESS1 protein	3	3	3	0	9	0	0	0	0	3	2	23
PGSC0003DMG400010137	Cysteine protease inhibitor 1	3	19	0	0	0	0	0	0	0	1	0	23
PGSC0003DMG400018109	Raffionse synthase 2	10	4	0	3	0	0	0	0	0	6	0	23
PGSC0003DMG400010132	Conserved gene of unknown function	6	14	0	0	0	0	0	0	0	0	3	23
PGSC0003DMG400024608	Senescence-associated protein	2	7	0	0	9	2	1	0	0	3	0	24
PGSC0003DMG400019502	Vesicle transport v-SNARE 11	0	0	18	4	0	0	0	0	0	1	1	24
PGSC0003DMG400018119	TRANSPARENT TESTA 12 protein	14	5	0	0	3	2	0	0	0	0	0	24
PGSC0003DMG400025339	Nitrate transporter	9	5	0	3	0	0	0	0	0	7	0	24
PGSC0003DMG400018184	Carbohydrate binding protein	8	6	0	0	0	0	0	0	0	7	3	24
PGSC0003DMG400045225	Gene of unknown function	12	11	0	0	0	0	1	0	0	0	0	24
PGSC0003DMG400004332	NAC domain protein	1	8	0	7	6	0	0	0	0	1	1	24
PGSC0003DMG400026494	Palmitoyl-acyl carrier protein thioesterase	6	1	0	0	15	0	0	0	0	2	0	24

PGSC0003DMG400024481	Pollen specific LIM domain-containing protein	0	1	0	17	0	0	0	0	0	3	3	24
PGSC0003DMG402010181	Xyloglucan endotransglucosylase/hydrolase protein 9	3	2	0	12	0	1	0	0	0	1	5	24
PGSC0003DMG400015235	Ubiquitin-protein ligase	3	1	3	0	14	0	0	0	0	3	0	24
PGSC0003DMG400013182	LOB domain-containing protein	4	2	18	0	0	0	0	0	0	0	0	24
PGSC0003DMG400000599	DNA-binding protein	0	1	12	0	0	0	0	0	0	9	2	24
PGSC0003DMG402014189	XH/XS domain-containing protein	2	2	0	18	0	0	0	0	0	1	1	24
PGSC0003DMG400025346	Voltage-dependent anion channel	7	7	0	0	0	2	0	0	0	4	4	24
PGSC0003DMG400000596	Flavonol synthase/flavanone 3-hydroxylase	3	2	15	0	0	0	0	0	0	2	2	24
PGSC0003DMG401024548	Leucine-rich repeat containing protein	2	4	7	5	0	0	0	0	0	0	6	24
PGSC0003DMG400024461	Conserved gene of unknown function	8	3	0	11	0	0	0	0	0	2	0	24
PGSC0003DMG400000640	Conserved gene of unknown function	0	2	0	12	0	0	0	0	0	6	4	24
PGSC0003DMG400015232	Pentatricopeptide repeat-containing protein	10	7	0	5	0	0	1	0	0	1	0	24
PGSC0003DMG400024667	Glycosyltransferase	8	4	4	0	0	0	0	0	0	6	2	24
PGSC0003DMG400024475	Calmodulin binding protein	2	4	0	0	14	0	0	0	0	3	2	25
PGSC0003DMG400034990	F-box protein interaction domain containing protein	3	21	0	0	0	0	1	0	0	0	0	25
PGSC0003DMG400018154	Conserved gene of unknown function	2	0	17	0	0	0	0	0	0	4	2	25
PGSC0003DMG400000644	Phox domain-containing protein	3	3	9	8	1	1	0	0	0	0	0	25
PGSC0003DMG400019519	Exonuclease	0	3	0	10	0	0	0	0	0	11	1	25
PGSC0003DMG400025631	F-box protein interaction domain containing protein	6	14	5	0	0	0	0	0	0	0	0	25
PGSC0003DMG400024528	Transposase Tam3	8	12	0	0	0	1	0	0	0	2	2	25
PGSC0003DMG400031057	Serine palmitoyltransferase	3	0	0	0	19	1	0	0	0	1	1	25
PGSC0003DMG400018177	Pentatricopeptide repeat-containing protein	6	7	0	0	0	0	0	0	0	7	5	25
PGSC0003DMG400014141	DEAD-box protein abstrakt	4	0	0	8	0	0	0	0	0	6	7	25
PGSC0003DMG401019826	Nucleic acid binding protein	3	5	0	10	7	0	0	0	0	0	0	25
PGSC0003DMG400024569	Rhicadhesin receptor	10	3	0	0	0	0	0	0	0	10	3	26
PGSC0003DMG400019532	Conserved gene of unknown function	6	4	6	0	0	0	0	0	0	9	1	26
PGSC0003DMG400024639	Histone deacetylase	5	4	17	0	0	0	0	0	0	0	0	26
PGSC0003DMG400018104	Cell division cycle protein 48	13	2	0	6	0	0	0	0	0	0	5	26
PGSC0003DMG400042259	Steroleosin	3	9	11	0	0	3	0	0	0	0	0	26
PGSC0003DMG400024638	ATP synthase subunit gamma, mitochondrial	1	0	0	8	13	0	0	0	0	2	2	26
PGSC0003DMG400015170	ATP binding protein	1	2	0	2	0	1	1	0	0	5	14	26
PGSC0003DMG400015187	Adenylate kinase B	0	0	0	19	0	1	0	0	0	6	0	26
PGSC0003DMG400033683	Dehydrogenase	4	1	0	19	0	0	0	0	0	0	2	26
PGSC0003DMG400024660	HCG23534, partial	2	2	22	0	0	0	0	0	0	0	0	26
PGSC0003DMG401018151	Pre-mRNA-splicing factor cwc23	1	1	0	22	0	0	1	0	0	0	1	26
PGSC0003DMG400018101	BRASSINOSTEROID INSENSITIVE 1-associated receptor kinase	6	10	5	0	0	1	1	0	0	1	2	26
PGSC0003DMG400012026	Conserved gene of unknown function	3	4	0	0	11	1	0	0	0	7	0	26
PGSC0003DMG400024631	SP1L	2	2	0	0	21	0	0	0	0	0	1	26
PGSC0003DMG400018167	UBX domain-containing protein 8-B	2	0	0	0	20	1	0	0	0	3	0	26
PGSC0003DMG404000594	Flavonol synthase/flavanone 3-hydroxylase	7	8	0	9	0	0	1	0	0	1	1	27
PGSC0003DMG402031052	Polysialic acid capsule expression protein kpsF	1	4	0	17	0	0	0	0	0	0	5	27
PGSC0003DMG400024594	Glycosyltransferase UGT90A7	9	2	0	14	0	0	0	0	0	2	0	27
PGSC0003DMG400014144	Glycerol kinase	4	0	0	0	19	2	0	0	0	0	2	27
PGSC0003DMG400004342	O-methyltransferase	3	2	13	0	0	0	0	0	0	9	0	27

PGSC0003DMG400033688	Tartrate-resistant acid phosphatase type 5	2	3	8	10	0	1	1	0	0	2	0	27
PGSC0003DMG402024549	ATP binding protein	2	5	15	0	0	0	0	0	0	3	2	27
PGSC0003DMG400024537	Basic 7S globulin 2 small subunit	10	6	0	0	0	0	0	0	0	10	1	27
PGSC0003DMG400019821	Cyclic nucleotide-gated ion channel	3	0	23	0	0	1	0	0	0	0	0	27
PGSC0003DMG400018157	Glutaredoxin	0	1	0	24	0	0	0	0	0	2	0	27
PGSC0003DMG400041939	Ubiquitin-protein ligase	10	17	0	0	0	0	0	0	0	0	0	27
PGSC0003DMG400015157	Receptor kinase	14	9	0	0	0	0	0	0	0	3	1	27
PGSC0003DMG400039684	Gene of unknown function	7	20	0	0	0	0	0	0	0	0	0	27
PGSC0003DMG400024539	ATEXO70E2	5	11	0	0	0	0	0	0	0	6	6	28
PGSC0003DMG400025632	Myb-like DNA-binding protein	2	4	6	0	13	0	0	0	0	3	0	28
PGSC0003DMG400015177	CONSTANS interacting protein 2a	3	2	0	23	0	0	0	0	0	0	0	28
PGSC0003DMG400015169	Esterase	4	5	0	14	0	0	0	0	0	1	4	28
PGSC0003DMG400014192	Mitochondrial glycoprotein family protein	3	3	10	0	0	0	0	0	0	12	0	28
PGSC0003DMG400024551	Conserved gene of unknown function	5	10	0	0	0	0	0	0	0	12	1	28
PGSC0003DMG400015264	Thylakoid lumenal 17.9 kDa protein, chloroplast	1	0	17	0	0	0	0	0	0	6	4	28
PGSC0003DMG400026483	HVA22 e	0	0	0	0	28	0	0	0	0	0	0	28
PGSC0003DMG400033693	UPA16	1	2	23	0	0	0	0	0	0	2	0	28
PGSC0003DMG400015198	Amino acid transporter	5	2	7	13	0	1	0	0	0	0	0	28
PGSC0003DMG400024633	Conserved gene of unknown function	2	5	0	4	0	0	1	0	0	14	2	28
PGSC0003DMG400019514	ATP binding protein	2	4	9	0	9	0	0	0	0	4	0	28
PGSC0003DMG400024593	Glycosyltransferase UGT90A7	8	14	0	0	0	0	0	0	0	0	6	28
PGSC0003DMG401031045	AGO1-1	2	2	4	14	0	0	0	0	0	1	6	29
PGSC0003DMG400015192	Malate synthase, glyoxysomal	12	4	0	10	0	2	0	0	0	1	0	29
PGSC0003DMG400024477	Calmodulin binding protein	5	16	0	2	0	0	0	0	0	3	3	29
PGSC0003DMG400024521	Gene of unknown function	0	4	0	24	0	1	0	0	0	0	0	29
PGSC0003DMG400015182	Deoxycytidylate deaminase	4	2	0	17	0	0	0	0	0	1	5	29
PGSC0003DMG400024579	Cell division cycle	5	0	20	4	0	0	0	0	0	0	0	29
PGSC0003DMG401024469	Pentatricopeptide repeat-containing protein	3	1	0	0	0	0	0	0	0	25	0	29
PGSC0003DMG400018160	Conserved gene of unknown function	7	13	0	0	0	0	1	0	0	8	0	29
PGSC0003DMG400025348	Quirky	15	4	0	0	0	0	0	0	0	6	4	29
PGSC0003DMG400015297	Conserved gene of unknown function	0	1	0	0	0	0	0	0	0	17	11	29
PGSC0003DMG402018159	Ubiquitin carrier protein	1	1	0	24	0	0	0	0	0	3	0	29
PGSC0003DMG400024662	Myosin heavy chain	10	10	0	9	0	0	0	0	0	0	0	29
PGSC0003DMG400015253	Glyceraldehyde-3-phosphate dehydrogenase	5	0	0	19	0	2	0	0	0	4	0	30
PGSC0003DMG400024458	HIPL1 protein	8	7	14	0	0	1	0	0	0	0	0	30
PGSC0003DMG400019524	YGL010w	0	0	0	0	19	0	0	0	0	10	1	30
PGSC0003DMG400015216	CDT1a protein	2	3	15	0	8	0	0	0	0	2	0	30
PGSC0003DMG401024517	Protease	3	2	0	16	0	0	0	0	0	6	3	30
PGSC0003DMG402015240	Conserved gene of unknown function	3	0	0	17	0	1	0	0	0	3	6	30
PGSC0003DMG400015280	Conserved gene of unknown function	5	4	0	9	0	0	0	0	0	7	5	30
PGSC0003DMG400019853	Sterile alpha motif domain-containing protein	1	4	0	0	0	0	0	0	0	16	9	30
PGSC0003DMG400019852	Translocon-associated protein, beta subunit	6	2	0	15	0	0	0	0	0	8	0	31
PGSC0003DMG400015212	Serine/threonine-protein phosphatase	2	1	13	7	3	0	0	0	0	4	1	31
PGSC0003DMG400033689	Conserved gene of unknown function	3	0	0	18	0	0	0	0	0	8	2	31

PGSC0003DMG400019858	Pentatricopeptide repeat-containing protein	11	2	0	2	0	1	0	0	0	15	0	31
PGSC0003DMG400019522	Heat shock protein binding protein	4	1	18	7	0	0	0	0	0	1	0	31
PGSC0003DMG400015188	Auxin-independent growth promoter	10	6	0	11	0	3	0	0	0	1	0	31
PGSC0003DMG400018115	ATP binding protein	5	1	0	0	0	0	1	0	0	20	4	31
PGSC0003DMG400000635	Longin	0	1	0	22	0	0	0	0	0	7	1	31
PGSC0003DMG400010151	ARC6H	6	2	0	18	4	0	0	0	0	0	1	31
PGSC0003DMG400015167	Zinc finger protein	3	1	0	21	0	0	0	0	0	0	6	31
PGSC0003DMG400024613	Protein ASC1	9	3	3	0	0	0	0	0	0	10	7	32
PGSC0003DMG400024459	HIPL1 protein	8	6	0	0	12	1	0	0	0	5	0	32
PGSC0003DMG400024640	Zinc ion binding protein	10	8	0	0	13	0	1	0	0	0	0	32
PGSC0003DMG400024632	Transcription factor	9	4	9	3	0	3	1	0	0	2	1	32
PGSC0003DMG401024590	Rac protein	1	3	0	24	0	4	0	0	0	0	0	32
PGSC0003DMG400026492	Protein kinase family protein	4	5	0	17	0	0	0	0	0	5	1	32
PGSC0003DMG400018178	Histone deacetylase	5	5	0	15	0	0	0	0	0	1	6	32
PGSC0003DMG401024463	DNA binding protein	3	5	12	0	0	1	0	0	0	0	11	32
PGSC0003DMG400019827	Nucleic acid binding protein	0	3	1	0	28	0	0	0	0	0	0	32
PGSC0003DMG400015225	Transposase	2	13	16	0	0	0	1	0	0	0	0	32
PGSC0003DMG400031044	MYB transcription factor	2	2	0	0	24	3	0	0	0	1	0	32
PGSC0003DMG400024648	Expansin	1	3	11	0	17	0	0	0	0	0	0	32
PGSC0003DMG400000612	AP2 domain-containing protein AP29	6	2	0	0	0	0	0	0	0	5	20	33
PGSC0003DMG403014139	WD-repeat protein	1	1	0	0	0	0	0	0	0	1	30	33
PGSC0003DMG400018127	Protein DEHYDRATION-INDUCED 19 homolog 6	4	7	5	0	0	2	0	0	0	11	4	33
PGSC0003DMG400024508	Extracellular Ca2+ sensing receptor	3	2	0	23	0	2	0	0	0	1	2	33
PGSC0003DMG400024484	BAH domain-containing protein	15	5	3	0	0	0	0	0	0	9	1	33
PGSC0003DMG400019824	JA-induced WRKY protein	5	4	0	14	0	0	0	0	0	9	1	33
PGSC0003DMG402024603	DR1	5	6	0	0	0	0	2	0	0	0	20	33
PGSC0003DMG400024624	Conserved gene of unknown function	2	0	0	22	0	1	0	0	0	3	5	33
PGSC0003DMG400015263	Nodulin	3	0	0	21	0	0	0	0	0	1	8	33
PGSC0003DMG400015229	BTB/POZ domain-containing protein	11	3	10	5	0	2	0	0	0	2	1	34
PGSC0003DMG400015270	ATP-binding cassette transporter	6	4	0	15	0	2	0	0	0	2	5	34
PGSC0003DMG400010153	ARF-GAP domain 15	0	0	1	0	26	4	0	0	0	3	0	34
PGSC0003DMG400014123	Aquaporin, MIP family, NIP subfamily	0	0	14	0	0	2	0	0	0	15	3	34
PGSC0003DMG400024573	Pentatricopeptide repeat-containing protein, chloroplastic	11	5	6	0	5	1	0	0	0	4	2	34
PGSC0003DMG400024601	Dead box ATP-dependent RNA helicase	5	2	8	8	7	3	0	0	0	1	1	35
PGSC0003DMG400024596	Protein kinase atmrk1	2	2	0	27	0	1	0	0	0	1	2	35
PGSC0003DMG400012025	UPF0497 membrane protein 8	0	1	34	0	0	0	0	0	0	0	0	35
PGSC0003DMG400000608	Polygalacturonase	2	3	9	0	8	0	0	0	0	13	0	35
PGSC0003DMG400015221	Cytochrome P450 71A2	10	14	0	10	0	1	0	0	0	0	0	35
PGSC0003DMG400025629	Conserved gene of unknown function	2	6	0	0	6	2	0	0	0	19	0	35
PGSC0003DMG400037483	Gene of unknown function	2	3	27	0	2	1	0	0	0	0	0	35
PGSC0003DMG400031744	SAUR family protein	1	4	0	0	0	0	0	0	0	28	3	36
PGSC0003DMG400025337	Nitrate transporter	8	7	0	0	8	2	0	0	0	11	0	36
PGSC0003DMG400024637	Conserved gene of unknown function	2	9	1	10	0	1	0	0	0	1	12	36
PGSC0003DMG400033681	GTP-binding protein alpha subunit, gna	7	0	24	0	0	0	0	0	0	4	1	36

PGSC0003DMG400000632	Pentatricopeptide repeat-containing protein	4	17	0	0	0	0	0	0	0	13	2	36
PGSC0003DMG400010156	Small nuclear ribonucleoprotein polypeptide	1	0	1	34	0	0	0	0	0	0	0	36
PGSC0003DMG402024581	Mannose-1-phosphate guanyltransferase	1	3	5	20	0	0	0	0	0	2	5	36
PGSC0003DMG400010188	Gene of unknown function	5	8	0	0	0	0	1	0	0	3	19	36
PGSC0003DMG402024610	Conserved gene of unknown function	9	7	13	0	0	1	0	0	0	6	0	36
PGSC0003DMG400019829	Nucleic acid binding protein	1	7	0	7	20	0	0	0	0	0	2	37
PGSC0003DMG400015208	Ser/Thr protein phosphatase family	0	5	5	18	0	0	0	0	0	1	8	37
PGSC0003DMG400018100	Homeobox 9	1	1	0	35	0	0	0	0	0	0	0	37
PGSC0003DMG400015283	Receptor kinase	13	24	0	0	0	0	0	0	0	0	0	37
PGSC0003DMG400010173	Avr9/Cf-9 rapidly elicited protein 140	1	1	35	0	0	0	0	0	0	0	0	37
PGSC0003DMG400018171	Receptor protein kinase	12	6	0	16	0	0	0	0	0	0	3	37
PGSC0003DMG401000617	Conserved gene of unknown function	3	4	14	0	0	0	0	0	0	15	1	37
PGSC0003DMG400014134	Conserved gene of unknown function	4	3	0	0	19	0	0	0	0	6	5	37
PGSC0003DMG400015207	Conserved gene of unknown function	5	3	8	0	18	0	0	0	0	3	0	37
PGSC0003DMG400015162	Nodulation receptor kinase	6	3	0	21	0	0	0	0	0	1	6	37
PGSC0003DMG400024641	24 kDa seed maturation protein	2	2	8	0	25	0	0	0	0	0	0	37
PGSC0003DMG400015189	Aspartyl aminopeptidase	2	3	14	8	4	1	0	0	0	0	5	37
PGSC0003DMG400004333	Conserved gene of unknown function	1	6	13	0	17	0	0	0	0	0	0	37
PGSC0003DMG402015205	Protein yrdA	2	0	14	0	16	2	0	0	0	3	1	38
PGSC0003DMG401015209	Conserved gene of unknown function	2	6	9	16	0	1	0	0	0	4	0	38
PGSC0003DMG400024474	Gonadotropin beta chain	1	2	2	0	22	2	0	0	0	5	4	38
PGSC0003DMG400018139	Cytochrome P450 71A4	11	15	0	4	0	1	1	0	0	2	4	38
PGSC0003DMG400010133	Sexual organ expressed protein	0	4	33	0	0	0	1	0	0	0	0	38
PGSC0003DMG400031054	Transcription factor	2	1	15	19	0	1	0	0	0	0	0	38
PGSC0003DMG402019860	Glycosyl transferase family 29 protein	6	4	12	1	0	1	0	0	0	1	13	38
PGSC0003DMG400024552	Synaptosomal associated protein	4	2	0	30	0	1	0	0	0	1	0	38
PGSC0003DMG401015202	Histone acetyltransferase	3	3	19	0	7	2	0	0	0	3	1	38
PGSC0003DMG400018089	Chromosome structural maintenance protein	8	4	0	20	0	0	0	0	0	1	5	38
PGSC0003DMG402018151	ATP binding protein	2	3	0	28	0	0	1	0	0	2	2	38
PGSC0003DMG400000616	Conserved gene of unknown function	0	0	8	2	26	0	0	0	0	0	3	39
PGSC0003DMG400015154	Hydrolase, hydrolyzing O-glycosyl compounds	7	4	0	8	0	0	1	0	0	9	10	39
PGSC0003DMG400024561	Ser/Thr protein kinase	3	4	17	4	0	1	0	0	0	7	3	39
PGSC0003DMG400024547	Diacylglycerol kinase, theta	8	5	14	4	0	0	0	0	0	1	7	39
PGSC0003DMG401004334	Pyruvate dehydrogenase	3	0	16	1	0	0	0	0	0	18	1	39
PGSC0003DMG400024520	Conserved gene of unknown function	3	0	28	0	0	1	0	0	0	5	2	39
PGSC0003DMG400024591	Cyclic nucleotide-gated ion channel 16	8	0	16	11	0	0	0	0	0	4	0	39
PGSC0003DMG400019512	Membrane associated ring finger 1, 8	0	1	0	0	0	0	0	0	0	34	4	39
PGSC0003DMG401024549	ATP binding protein	4	6	0	19	0	0	0	0	0	7	3	39
PGSC0003DMG400004340	DNA binding protein	5	3	0	12	17	0	0	0	0	0	2	39
PGSC0003DMG401024472	Endonuclease III	3	6	0	29	0	0	0	0	0	1	1	40
PGSC0003DMG400000642	ATP binding protein	2	3	0	0	30	0	0	0	0	3	2	40
PGSC0003DMG400025334	Nitrate transporter	4	4	0	5	0	1	0	0	0	11	15	40
PGSC0003DMG400018187	Pentatricopeptide repeat-containing protein	6	4	0	23	0	0	0	0	0	7	0	40
PGSC0003DMG400000601	ZR1 protein	1	3	22	0	7	0	0	0	0	5	2	40

PGSC0003DMG400015171	ATP binding protein	13	6	0	0	0	0	1	0	0	17	3	40
PGSC0003DMG403024620	BSD domain containing protein	5	6	3	1	0	0	1	0	0	18	6	40
PGSC0003DMG400024578	Type-A response regulator	1	2	0	4	1	0	0	0	0	27	5	40
PGSC0003DMG400025330	BRASSINOSTEROID INSENSITIVE 1-associated receptor kinase	5	2	0	25	0	1	0	0	0	2	5	40
PGSC0003DMG400015218	F-box protein interaction domain containing protein	10	13	0	17	0	0	0	0	0	0	0	40
PGSC0003DMG400018103	Carboxyl-terminal proteinase	1	0	0	34	0	1	0	0	0	2	2	40
PGSC0003DMG400033680	Protein SSU72	2	0	0	26	7	0	0	0	0	4	2	41
PGSC0003DMG400014207	Calcineurin	3	0	0	31	0	0	0	0	0	5	2	41
PGSC0003DMG402015159	Protein tipD	3	4	0	11	16	4	0	0	0	2	1	41
PGSC0003DMG400024545	Gene of unknown function	2	2	0	0	0	0	0	0	0	3	34	41
PGSC0003DMG400018148	ATPANK2 (PANTOTHENATE KINASE 2)	1	3	9	0	23	0	0	0	0	2	3	41
PGSC0003DMG401015205	TDR8 protein	2	2	0	35	0	1	0	0	0	0	1	41
PGSC0003DMG400015200	GDP-mannose transporter	8	4	18	0	0	1	0	0	0	8	2	41
PGSC0003DMG400018153	Gibberellin receptor GID1	0	1	0	19	6	1	0	0	0	0	14	41
PGSC0003DMG400015252	Conserved gene of unknown function	7	5	24	0	0	0	0	0	0	5	1	42
PGSC0003DMG400018122	Zinc ion binding protein	6	13	0	9	9	1	0	0	0	0	4	42
PGSC0003DMG400000598	Conserved gene of unknown function	5	2	0	20	14	1	0	0	0	0	0	42
PGSC0003DMG400017116	Nodulin family protein	3	5	12	0	0	0	0	0	0	18	4	42
PGSC0003DMG400025335	Hypersensitive-induced reaction protein	2	0	0	6	0	0	0	0	0	4	30	42
PGSC0003DMG400014198	Conserved gene of unknown function	4	8	18	0	0	0	2	0	0	10	0	42
PGSC0003DMG400024584	Alpha-n-acetylglucosaminidase	2	3	0	36	0	0	1	0	0	0	0	42
PGSC0003DMG402024654	Heparanase-2	1	9	0	27	0	0	0	0	0	0	5	42
PGSC0003DMG400024532	Multidrug resistance protein	6	1	2	3	23	1	0	0	0	5	1	42
PGSC0003DMG400019504	Sand family protein	2	2	0	0	27	0	0	0	0	10	2	43
PGSC0003DMG402015244	EMB2746	1	0	14	18	2	1	0	0	0	1	6	43
PGSC0003DMG400010150	Conserved gene of unknown function	8	13	9	10	0	0	0	0	0	3	0	43
PGSC0003DMG400024570	5-formyltetrahydrofolate cycloligase	7	5	0	25	0	0	0	0	0	6	0	43
PGSC0003DMG400024541	Heat shock protein	11	13	9	0	0	1	0	0	0	10	0	44
PGSC0003DMG400026478	Transcription regulator	0	0	27	0	9	0	0	0	0	7	1	44
PGSC0003DMG400018168	Transcription factor	0	7	8	10	0	1	0	0	0	8	10	44
PGSC0003DMG400024536	Mitochondrial import receptor subunit TOM5 homolog	1	0	0	28	0	0	0	0	0	15	0	44
PGSC0003DMG400024580	Pyridoxamine 5-phosphate oxidase	3	10	11	1	0	1	0	0	0	18	0	44
PGSC0003DMG400024534	Conserved gene of unknown function	4	11	0	0	13	3	1	0	0	2	10	44
PGSC0003DMG400024490	Aldehyde dehydrogenase	3	5	0	7	22	0	0	0	0	5	2	44
PGSC0003DMG401024473	Mannosyl-oligosaccharide 1, 2-alpha-mannosidase IA	1	2	0	33	0	2	0	0	0	4	2	44
PGSC0003DMG400024659	Conserved gene of unknown function	4	17	0	16	0	1	0	0	0	2	4	44
PGSC0003DMG400015278	Cytochrome P450	1	0	0	37	0	1	0	0	0	5	1	45
PGSC0003DMG400010158	Kinase interacting protein	1	0	0	34	0	0	0	0	0	8	2	45
PGSC0003DMG400014135	Nodulin	10	4	18	0	0	0	0	0	0	8	5	45
PGSC0003DMG400015191	Conserved gene of unknown function	5	5	0	20	5	0	0	0	0	9	1	45
PGSC0003DMG400019856	Poly(A) polymerase	2	2	11	0	27	1	1	0	0	1	0	45
PGSC0003DMG401004331	Zinc finger protein	2	1	0	42	0	0	0	0	0	0	0	45
PGSC0003DMG400015197	Bromodomain-containing protein	10	7	19	0	0	0	0	0	0	6	3	45
PGSC0003DMG400013180	Pentatricopeptide repeat-containing protein	8	5	0	24	0	0	0	0	0	7	1	45

PGSC0003DMG400024499	Para-hydroxybenzoate--polyprenyltransferase	4	6	15	2	0	2	0	0	0	16	0	45
PGSC0003DMG403014197	Serine/threonine-protein kinase PBS1	4	5	0	8	0	1	0	0	0	27	1	46
PGSC0003DMG403024509	Cell growth defect factor	1	2	40	0	0	0	0	0	0	2	1	46
PGSC0003DMG401015240	Ubiquitin-activating enzyme E1 domain-containing protein	1	3	3	33	0	2	0	0	0	3	1	46
PGSC0003DMG400015307	Gene of unknown function	0	1	0	0	0	0	0	0	0	0	45	46
PGSC0003DMG401014138	WD-repeat protein	4	2	19	19	2	1	0	0	0	0	0	47
PGSC0003DMG400015180	Gamma tubulin	3	0	0	36	6	1	0	0	0	0	1	47
PGSC0003DMG400018099	Structure-specific recognition protein	5	0	9	12	0	4	0	0	0	9	8	47
PGSC0003DMG400024553	Conserved gene of unknown function	8	12	5	0	16	1	0	0	0	4	1	47
PGSC0003DMG400018183	ATP binding protein	4	3	0	0	0	0	0	0	0	6	34	47
PGSC0003DMG400018175	Nucleic acid binding	4	5	8	21	0	3	0	0	0	0	6	47
PGSC0003DMG402018143	ABC transporter A family member 2	6	6	0	8	22	2	0	1	0	0	2	47
PGSC0003DMG401018143	Conserved gene of unknown function	4	10	28	0	0	4	0	0	0	2	0	48
PGSC0003DMG400024567	Galactosyltransferase	6	5	0	24	0	1	0	0	0	12	0	48
PGSC0003DMG400018080	Conserved gene of unknown function	1	2	13	7	0	1	0	0	0	4	20	48
PGSC0003DMG400024497	Zinc finger family protein	1	6	0	15	20	1	0	0	0	4	1	48
PGSC0003DMG400031047	Ornithine decarboxylase	4	11	0	0	32	1	0	0	0	0	0	48
PGSC0003DMG400013184	Zinc finger protein	13	9	7	0	13	1	0	0	0	6	0	49
PGSC0003DMG400031041	D-cysteine desulfhydrase	3	5	0	24	14	0	0	0	0	3	0	49
PGSC0003DMG400014120	Stromal cell-derived factor 2	0	2	0	39	0	0	0	0	0	5	3	49
PGSC0003DMG400010176	Zinc finger protein	4	3	33	0	0	0	0	0	0	7	2	49
PGSC0003DMG401024510	Enolase	5	4	21	12	0	2	0	0	0	3	2	49
PGSC0003DMG400024575	Microtubule-associated protein TORTIFOLIA1	16	3	16	0	0	0	0	0	0	15	0	50
PGSC0003DMG400015276	Serine-threonine protein kinase, plant-type	5	4	0	20	0	1	0	0	0	18	2	50
PGSC0003DMG400024492	Deleted in split hand/splt foot protein 1	1	0	43	0	0	0	0	0	0	4	2	50
PGSC0003DMG402000630	Racemase and epimerase, acting on amino acids and derivativ	6	2	0	15	0	1	0	0	0	9	17	50
PGSC0003DMG400015241	AMP-activated protein kinase, gamma regulatory subunit	7	0	0	0	29	2	0	0	0	10	2	50
PGSC0003DMG400024626	MADS-box protein 1	2	1	0	0	38	3	0	0	0	3	3	50
PGSC0003DMG400018174	Short-chain dehydrogenase	2	3	37	0	3	4	0	0	0	1	0	50
PGSC0003DMG400018138	Cytochrome P450 71A4	9	28	0	0	0	0	0	0	0	13	0	50
PGSC0003DMG400018195	Afc	5	4	0	0	3	1	0	0	0	6	32	51
PGSC0003DMG402015259	CONSTANS interacting protein 2a	0	0	0	45	3	0	0	0	0	2	1	51
PGSC0003DMG400015231	Ufm1-specific protease	6	4	20	0	15	3	0	0	0	3	0	51
PGSC0003DMG400031053	Tubulin-specific chaperone B	0	0	0	6	37	0	0	0	0	7	1	51
PGSC0003DMG400025332	Integral membrane HPP family protein	4	0	1	0	40	0	0	0	0	0	6	51
PGSC0003DMG400017115	Nodulin family protein	6	5	0	27	0	0	1	0	0	2	10	51
PGSC0003DMG400031051	Conserved gene of unknown function	0	0	0	47	0	0	0	0	0	1	3	51
PGSC0003DMG400024513	Serine/threonine protein kinase	11	3	27	0	0	0	0	0	0	10	1	52
PGSC0003DMG400018193	Hydrolase	4	5	0	28	0	2	0	0	0	4	9	52
PGSC0003DMG400014194	Defective in cullin neddylation protein	2	2	22	0	18	1	0	1	0	4	2	52
PGSC0003DMG400024642	Glucan endo-1, 3-beta-glucosidase 3	1	3	0	9	34	0	0	0	0	1	5	53
PGSC0003DMG400018117	Leucine-rich repeat-containing protein	6	7	0	29	0	1	0	0	0	9	1	53
PGSC0003DMG400015153	Tryptophan biosynthesis protein, trpc	7	2	42	0	0	0	0	0	0	1	1	53
PGSC0003DMG400015251	Plastid RNA-binding protein	5	4	0	36	7	1	0	0	0	0	0	53

PGSC0003DMG400019819	DNA-3-methyladenine glycosylase	3	0	24	24	0	0	0	0	0	3	0	54
PGSC0003DMG400024478	Calmodulin-binding protein	6	19	0	8	0	3	0	1	0	13	4	54
PGSC0003DMG400018106	Conserved gene of unknown function	2	5	19	4	0	0	0	0	0	17	8	55
PGSC0003DMG400018130	D-3-phosphoglycerate dehydrogenase	8	1	0	34	0	1	0	0	0	7	4	55
PGSC0003DMG400014193	Conserved gene of unknown function	0	1	0	37	0	0	0	0	0	3	14	55
PGSC0003DMG400015155	28 kDa ribonucleoprotein, chloroplastic	4	0	44	0	0	0	0	0	0	7	0	55
PGSC0003DMG400018079	Nuclear ribonuclease Z	4	5	0	39	0	0	0	0	0	8	0	56
PGSC0003DMG400015281	Conserved gene of unknown function	5	4	20	17	0	0	0	0	0	6	4	56
PGSC0003DMG400024647	Expansin	3	3	0	15	35	0	0	0	0	0	0	56
PGSC0003DMG400024500	HT1 protein kinase	7	5	0	0	38	2	0	0	0	3	2	57
PGSC0003DMG400015211	Beta-galactosidase	2	4	0	13	37	1	0	0	0	0	0	57
PGSC0003DMG400015245	Ribosomal protein PETRP	4	1	0	24	20	1	0	0	0	5	2	57
PGSC0003DMG402015209	NPK2	6	5	1	4	0	1	0	0	0	34	6	57
PGSC0003DMG400024635	Conserved gene of unknown function	1	9	0	37	0	1	0	0	0	8	1	57
PGSC0003DMG400024531	Thylakoid lumenal 25.6 kDa protein, chloroplast	0	0	0	0	57	0	0	0	0	0	0	57
PGSC0003DMG400010174	Peroxisomal membrane 22 kDa family protein	4	1	0	0	47	3	0	0	0	3	0	58
PGSC0003DMG400024512	H/ACA ribonucleoprotein complex subunit	3	2	17	0	0	1	0	0	0	25	10	58
PGSC0003DMG400015265	Phospholipase C 4	11	2	0	39	0	0	0	0	0	3	3	58
PGSC0003DMG400015238	Kelch repeat protein	4	2	0	7	45	0	0	0	0	0	0	58
PGSC0003DMG400015165	2-deoxyglucose-6-phosphate phosphatase	2	2	29	0	24	1	0	0	0	1	0	59
PGSC0003DMG401024603	TATA-binding protein-associated phosphoprotein	8	8	0	17	0	1	2	0	0	0	23	59
PGSC0003DMG400024582	Aminoaldehyde dehydrogenase 2	4	0	0	23	28	0	0	0	0	3	1	59
PGSC0003DMG400015243	SAP domain-containing protein	2	2	33	0	18	1	0	0	0	3	0	59
PGSC0003DMG400024571	Conserved gene of unknown function	3	2	46	0	5	0	0	0	0	3	0	59
PGSC0003DMG400031039	Conserved gene of unknown function	0	2	24	5	18	1	1	0	0	8	0	59
PGSC0003DMG400025336	Nitrate transporter NRT1-5	11	5	0	32	0	3	0	0	0	2	6	59
PGSC0003DMG400014202	50S ribosomal protein L3	2	0	0	52	0	1	0	0	0	4	1	60
PGSC0003DMG400018110	Alliin lyase	3	3	0	0	47	1	0	0	0	0	6	60
PGSC0003DMG401031036	Chalcone reductase	13	8	0	17	0	0	0	0	0	21	1	60
PGSC0003DMG400019521	Ribulose-phosphate 3-epimerase, chloroplastic	3	1	7	26	9	0	0	0	0	8	7	61
PGSC0003DMG400010175	DNA-binding protein RHL1	6	6	20	10	0	1	0	0	0	5	13	61
PGSC0003DMG402024480	Gene of unknown function	3	5	0	0	0	0	0	0	0	51	2	61
PGSC0003DMG400033685	Calmodulin	0	0	0	0	47	0	0	0	0	11	3	61
PGSC0003DMG400024609	Conserved gene of unknown function	2	6	0	48	0	1	0	0	0	0	4	61
PGSC0003DMG400018158	Conserved gene of unknown function	8	8	0	19	13	0	0	0	0	2	11	61
PGSC0003DMG400024625	MADS box protein	2	3	0	13	37	2	0	0	0	2	2	61
PGSC0003DMG400039813	Gene of unknown function	1	2	0	0	55	1	1	1	0	0	0	61
PGSC0003DMG400018185	Conserved gene of unknown function	2	1	13	18	27	0	0	0	0	1	0	62
PGSC0003DMG400024615	WD-repeat protein	10	11	0	36	4	1	0	0	0	0	0	62
PGSC0003DMG400015273	Alpha-galactosidase	0	2	22	3	29	1	0	0	0	3	2	62
PGSC0003DMG400025328	Plasma membrane ATPase 1	10	0	11	0	27	4	0	0	0	8	3	63
PGSC0003DMG401024581	Pyridoxamine 5-phosphate oxidase	3	2	29	19	0	3	0	0	0	5	2	63
PGSC0003DMG400026493	DNA-binding protein smubp-2	5	7	22	0	17	0	0	0	0	8	4	63
PGSC0003DMG400024524	Microtubule-associated protein	3	6	31	18	0	1	0	0	0	2	2	63

PGSC0003DMG400013181	Ubiquitin thiolesterase/ zinc ion binding	6	23	0	0	30	4	0	0	0	0	0	63
PGSC0003DMG400024634	70 kDa subunit of replication protein A	7	6	37	0	0	7	0	0	0	4	3	64
PGSC0003DMG400024559	ATP binding protein	9	5	0	19	14	0	0	0	0	10	7	64
PGSC0003DMG400015190	AMP dependent CoA ligase	7	6	48	0	0	0	0	0	0	1	2	64
PGSC0003DMG400019518	Pseudo response regulator	9	5	0	30	0	0	0	0	0	13	7	64
PGSC0003DMG400018081	WRKY72	8	6	0	5	45	0	0	0	0	0	0	64
PGSC0003DMG400013183	Calcium-dependent protein kinase 1	11	1	0	0	47	0	0	0	0	5	1	65
PGSC0003DMG400018142	Pectate lyase	4	5	0	37	0	1	0	0	0	17	1	65
PGSC0003DMG400024523	Transcription factor BIM1	1	8	25	24	0	1	0	0	0	0	6	65
PGSC0003DMG400000602	Conserved gene of unknown function	5	6	6	33	7	2	0	0	0	3	3	65
PGSC0003DMG400024623	Glycosyltransferase QUASIMODO1	9	0	10	32	0	0	0	0	0	3	11	65
PGSC0003DMG400015268	Ubiquitin activating enzyme	0	1	0	3	56	1	0	0	0	3	2	66
PGSC0003DMG400031050	Conserved gene of unknown function	5	5	0	30	0	0	0	0	0	22	4	66
PGSC0003DMG400026485	Steroleosin	4	5	0	56	0	1	0	0	0	0	0	66
PGSC0003DMG400031730	Protein DEK	3	10	0	7	38	1	0	0	0	6	2	67
PGSC0003DMG400018116	C2 and GRAM domain-containing protein	13	13	0	33	0	3	0	0	0	4	1	67
PGSC0003DMG400010179	Gene of unknown function	1	10	0	53	1	2	0	0	0	0	0	67
PGSC0003DMG400024535	Conserved gene of unknown function	25	10	0	3	5	0	0	0	0	19	5	67
PGSC0003DMG400015161	Replication factor C / DNA polymerase III gamma-tau subunit	9	13	0	39	0	0	0	0	0	6	0	67
PGSC0003DMG400025349	ATP-dependent Clp protease proteolytic subunit	3	2	32	5	5	1	0	0	0	7	12	67
PGSC0003DMG400015210	UPF0426 protein, chloroplastic	0	5	0	0	61	2	0	0	0	0	0	68
PGSC0003DMG400015226	Protein-L-isoaspartate O-methyltransferase	5	3	22	31	0	0	1	0	0	6	0	68
PGSC0003DMG400018173	Pentatricopeptide repeat-containing protein, mitochondrial	6	5	0	22	0	0	1	0	0	31	4	69
PGSC0003DMG400024585	Methyltransferase gidB	3	3	57	0	0	3	0	0	0	4	0	70
PGSC0003DMG400031043	DNA polymerase epsilon subunit B	10	1	0	46	12	1	0	0	0	1	0	71
PGSC0003DMG400024479	Calmodulin-binding protein	3	6	0	0	61	1	0	0	0	0	0	71
PGSC0003DMG400018107	Notchless	6	0	0	37	24	2	0	0	0	2	0	71
PGSC0003DMG401015175	B3 domain-containing protein Os01g0905400	7	9	0	43	0	2	0	1	0	6	4	72
PGSC0003DMG400024525	Dead box ATP-dependent RNA helicase	3	4	0	56	0	1	0	0	0	2	7	73
PGSC0003DMG400015152	Copine	12	4	0	16	28	6	0	0	0	6	1	73
PGSC0003DMG400024643	Flavonoid 3'-monooxygenase	13	3	0	25	0	0	0	0	0	0	33	74
PGSC0003DMG400018082	Ubiquitin carrier protein	1	0	68	0	0	2	0	0	0	2	1	74
PGSC0003DMG400015257	Ribosome	5	3	0	39	15	0	0	0	0	13	0	75
PGSC0003DMG400024612	Uridine cytidine kinase I	6	2	6	19	0	3	0	0	0	25	14	75
PGSC0003DMG400024586	PBng143	4	7	11	0	0	0	0	0	0	51	3	76
PGSC0003DMG401024480	Conserved gene of unknown function	10	14	0	0	51	1	0	0	0	0	0	76
PGSC0003DMG400024505	Chromosome-associated kinesin KIF4A	14	8	0	39	0	4	0	0	0	11	1	77
PGSC0003DMG401024509	Acetyl-CoA synthetase	10	4	0	34	0	1	0	0	0	25	5	79
PGSC0003DMG400018096	U3 small nucleolar ribonucleoprotein protein mpp10	9	13	0	49	0	4	0	0	0	1	3	79
PGSC0003DMG400015158	Protein bem46	6	1	0	0	69	0	0	0	0	1	3	80
PGSC0003DMG400024675	Gene of unknown function	0	0	0	0	0	0	0	0	0	6	75	81
PGSC0003DMG400014195	Nucleoporin	7	6	0	58	0	1	0	0	0	5	4	81
PGSC0003DMG400033687	Hydrolase, alpha/beta fold family protein	7	4	19	0	41	0	0	0	0	7	4	82
PGSC0003DMG400010180	TPR domain containing protein	2	8	33	0	0	1	0	0	0	35	3	82

PGSC0003DMG400015215	Asparaginyl-tRNA synthetase, chloroplastic/mitochondrial	8	4	26	39	1	2	0	0	0	2	0	82
PGSC0003DMG400024502	AtPex14p	7	15	13	33	4	3	0	1	0	4	3	83
PGSC0003DMG400018191	Kinase	6	13	0	39	21	0	0	0	0	3	1	83
PGSC0003DMG402014138	WD-repeat protein	5	16	42	12	7	2	0	0	0	0	0	84
PGSC0003DMG400018144	Vacuolar protein sorting	10	0	71	0	0	0	0	0	0	2	1	84
PGSC0003DMG400015239	Coproporphyrinigen III oxidase	16	9	0	33	0	1	0	0	0	12	14	85
PGSC0003DMG400014140	Prli-interacting factor l	5	4	0	48	24	3	0	0	0	2	0	86
PGSC0003DMG400015199	Conserved gene of unknown function	5	16	0	49	0	0	0	0	0	9	7	86
PGSC0003DMG400024483	Structural constituent of ribosome	1	1	0	4	72	0	0	0	0	8	3	89
PGSC0003DMG400018181	DEAD-box ATP-dependent RNA helicase 30	4	6	9	22	21	2	0	0	0	29	2	95
PGSC0003DMG400024503	1-deoxy-D-xylulose-5-phosphate reductoisomerase	16	2	0	0	64	4	0	0	0	5	6	97
PGSC0003DMG400035007	Conserved gene of unknown function	0	1	0	15	82	0	0	0	0	0	0	98
PGSC0003DMG400018129	High-affinity nitrate transport system component	3	5	64	0	19	2	0	0	0	2	3	98
PGSC0003DMG400018172	Conserved gene of unknown function	0	0	30	0	68	1	0	0	0	0	1	100
PGSC0003DMG400014136	Prolyl-tRNA synthetase	1	3	20	67	0	4	0	0	0	5	0	100
PGSC0003DMG400024588	Permease I	4	4	0	0	85	4	0	0	0	2	3	102
PGSC0003DMG400026490	Glutamine-rich protein	12	3	0	0	65	0	0	0	0	23	1	104
PGSC0003DMG400015266	CBS domain containing protein	7	4	0	88	0	0	0	0	0	2	4	105
PGSC0003DMG402024590	Rac protein	1	3	36	51	0	5	0	0	0	9	0	105
PGSC0003DMG400024574	Conserved gene of unknown function	4	4	0	48	32	1	0	0	0	6	13	108
PGSC0003DMG400019515	SET protein, phospatase 2A inhibitor	7	3	2	59	28	2	1	0	0	7	0	109
PGSC0003DMG400024465	Conserved gene of unknown function	4	5	35	38	18	1	0	0	0	6	2	109
PGSC0003DMG400024560	Formin 20	7	1	11	52	2	1	1	0	0	13	23	111
PGSC0003DMG400046688	Gene of unknown function	4	14	0	37	56	3	0	0	0	0	0	114
PGSC0003DMG402024509	Acetyl-CoA synthetase	8	6	35	52	0	0	0	0	0	3	17	121
PGSC0003DMG400015168	Nucleic acid binding protein	2	2	58	0	51	2	0	0	0	10	0	125
PGSC0003DMG400018194	Conserved gene of unknown function	14	11	61	0	29	4	0	0	0	6	2	127
PGSC0003DMG400010157	Forever young oxidoreductase	4	6	72	36	3	2	1	0	0	2	2	128
PGSC0003DMG400026491	Sigma factor	9	13	27	33	14	2	0	0	0	10	29	137
PGSC0003DMG400018108	FKBP12-interacting protein of 37 kDa	7	2	114	0	0	2	0	0	0	10	2	137
PGSC0003DMG400024511	MIS12 homologue	6	9	46	49	23	2	0	0	0	7	1	143
PGSC0003DMG401014139	WD-repeat protein	3	8	13	46	0	1	3	0	0	11	63	148
PGSC0003DMG400031735	Conserved gene of unknown function	8	5	0	5	102	0	0	1	0	7	23	151
PGSC0003DMG400015246	Protein tyrosine phosphatase	7	13	91	0	48	1	0	0	0	1	1	162
PGSC0003DMG400031733	Ribosomal pseudouridine synthase	7	4	0	98	49	5	1	0	0	17	24	205
PGSC0003DMG402024649	EMB2733/ESP3 (EMBRYO DEFECTIVE 2733)	36	17	89	97	24	12	2	0	0	6	10	293
PGSC0003DMG400015233	Transferase, transferring glycosyl groups	3	1	18	0	419	10	0	0	0	4	25	480
	**Sums**	3114	3030	3716	5633	4172	404	71	13	1	3045	2006	25205

Table S5: Transcript data used for eQTL analysis in the F1 population A×R.

Normalised transcript data (2−ΔCt values) for 43 marker genes were used from two trials, MPI Golm foil greenhouse [61711] and JKI shelter [68015], in the year 2014 measuring 95 A×R lines. For further details of the experiments see Haas et al. [38] and for details on the measurements see Sprenger *et al.* [14]. Missing data are presented by -99.

Location_ Treatment	MPI_FGH_Co [61711]																					Location_ Treatment
Ranking	11	12	27	47	38	23	25	54	45	1	66	22	35	17	57	21	6	8	3	24	33	Ranking
Gene product	Glutathione-S-transferase	Bacterial_spot_ disease_ resistance_ protein_4	Glutamyl-tRNA(Gln)_amidotransferase_subunit_A	(S)-norcoclaurine synthase	Cytochrome_ P450	PR1_protein	Beta-D-glucan_ exohydrolase	SBT4B_protein	Ethylene-inducing_ xylanase	Glucosyltransferase	Nbs-lrr_ resistance_ protein	Acidic_class_II_ 1_3-beta-glucanase	Hcr9-OR2A	Serine-threonine protein kinase, plant-type	Gamma_amino butyrate_ transaminase_ isoform1	O-methyltransferase	Multidrug_resistance_protein_ABC_transporter_ family	BED_finger-nbs-lrr_ resistance_ protein	S-locus-specific_ glycoprotein_ S6	NADPH-dependent_ codeinone_ reductase	Desacetoxyvindoline_4-hydroxylase	Gene product
Transcript ID F1-Individuals	PGSC0003 DMT400004539	PGSC0003 DMT400006231	PGSC0003 DMT400008092	PGSC0003 DMT400008278	PGSC0003 DMT400008547	PGSC0003 DMT400013094	PGSC0003 DMT400015224	PGSC0003 DMT400017633	PGSC0003 DMT400020366	PGSC0003 DMT400021019	PGSC0003 DMT400021608	PGSC0003 DMT400027201	PGSC0003 DMT400027792	PGSC0003 DMT400028434	PGSC0003 DMT400030682	PGSC0003 DMT400031370	PGSC0003 DMT400031714	PGSC0003 DMT400035714	PGSC0003 DMT400036026	PGSC0003 DMT400037483	PGSC0003 DMT400041989	Transcript ID F1-Individuals
1	2,23225215	0,24123314	0,26990058	0,47714511	0,32499738	0,49913432	9,09768826	1,18961933	0,05257424	0,01471570	0,02372415	0,08336005	0,00575687	0,03198413	0,99206049	0,80134756	0,09588839	0,02111629	0,05697607	1,36178599	0,05450378	1
2	-99	-99	-99	-99	-99	-99	-99	-99	-99	-99	-99	-99	-99	-99	-99	-99	-99	-99	-99	-99	-99	2
3	-99	-99	-99	-99	-99	-99	-99	-99	-99	-99	-99	-99	-99	-99	-99	-99	-99	-99	-99	-99	-99	3
4	-99	-99	-99	-99	-99	-99	-99	-99	-99	-99	-99	-99	-99	-99	-99	-99	-99	-99	-99	-99	-99	4
5	-99	-99	-99	-99	-99	-99	-99	-99	-99	-99	-99	-99	-99	-99	-99	-99	-99	-99	-99	-99	-99	5
6	0,06042335	0,10375480	0,10483919	0,29735330	0,13776201	0,46692088	10,39090927	4,88463242	0,15552841	0,02886070	0,01284410	0,03180175	0,00029526	0,43204395	1,54461790	0,67654134	0,04582409	0,03893604	0,02834523	0,56536325	0,19468991	6
7	-99	-99	-99	-99	-99	-99	-99	-99	-99	-99	-99	-99	-99	-99	-99	-99	-99	-99	-99	-99	-99	7
8	-99	-99	-99	-99	-99	-99	-99	-99	-99	-99	-99	-99	-99	-99	-99	-99	-99	-99	-99	-99	-99	8
9	-99	-99	-99	-99	-99	-99	-99	-99	-99	-99	-99	-99	-99	-99	-99	-99	-99	-99	-99	-99	-99	9
10	-99	-99	-99	-99	-99	-99	-99	-99	-99	-99	-99	-99	-99	-99	-99	-99	-99	-99	-99	-99	-99	10
11	-99	-99	-99	-99	-99	-99	-99	-99	-99	-99	-99	-99	-99	-99	-99	-99	-99	-99	-99	-99	-99	11
12	-99	-99	-99	-99	-99	-99	-99	-99	-99	-99	-99	-99	-99	-99	-99	-99	-99	-99	-99	-99	-99	12
13	-99	-99	-99	-99	-99	-99	-99	-99	-99	-99	-99	-99	-99	-99	-99	-99	-99	-99	-99	-99	-99	13
14	-99	-99	-99	-99	-99	-99	-99	-99	-99	-99	-99	-99	-99	-99	-99	-99	-99	-99	-99	-99	-99	14
15	-99	-99	-99	-99	-99	-99	-99	-99	-99	-99	-99	-99	-99	-99	-99	-99	-99	-99	-99	-99	-99	15
16	5,83304822	0,32381686	0,21692226	0,57087627	0,43114648	0,27135436	25,05887948	3,08709526	0,21587229	0,22286624	0,03347551	0,11275396	0,00224239	1,19188910	1,79408718	0,66032584	0,19590824	0,01683083	0,01486692	0,75904115	0,30698711	16
17	5,36006316	0,24557745	0,25283183	0,49974014	0,40451076	3,55352291	0,32269654	7,21625340	0,09370371	0,01498071	0,06453609	0,37794547	0,00835728	0,53858651	1,64518520	0,52823434	0,09494593	0,03540876	0,02586688	0,62211362	0,31737271	17
18	0,41509113	0,48013100	0,25587303	0,61153204	0,16442482	1,81315216	9,80488018	5,39828071	0,29108250	0,02693265	0,06308759	0,38090417	0,01109843	0,60730788	2,36280407	0,45454656	0,20783348	0,04329967	0,02061017	0,64505765	0,26710891	18
19	2,96904714	0,26152123	0,21183311	0,32646495	0,18569412	0,35453501	21,28846466	2,83431479	0,08591179	0,02240555	0,02950289	0,03159850	0,00913107	0,78349719	1,17202228	0,77916458	0,25384153	0,01039476	0,16828751	0,20993287	0,04772867	19
20	-99	-99	-99	-99	-99	-99	-99	-99	-99	-99	-99	-99	-99	-99	-99	-99	-99	-99	-99	-99	-99	20
21	-99	-99	-99	-99	-99	-99	-99	-99	-99	-99	-99	-99	-99	-99	-99	-99	-99	-99	-99	-99	-99	21
22	-99	-99	-99	-99	-99	-99	-99	-99	-99	-99	-99	-99	-99	-99	-99	-99	-99	-99	-99	-99	-99	22
23	-99	-99	-99	-99	-99	-99	-99	-99	-99	-99	-99	-99	-99	-99	-99	-99	-99	-99	-99	-99	-99	23
24	3,95108806	0,26558582	0,26265665	0,52933392	0,13051160	0,23573698	20,70991892	1,47759409	0,08022802	0,02768503	0,00950070	0,03768802	0,00300223	0,21378759	0,79402151	0,29103206	0,05105691	0,02385193	0,04541303	0,42846522	0,08486127	24
25	-99	-99	-99	-99	-99	-99	-99	-99	-99	-99	-99	-99	-99	-99	-99	-99	-99	-99	-99	-99	-99	25
26	-99	-99	-99	-99	-99	-99	-99	-99	-99	-99	-99	-99	-99	-99	-99	-99	-99	-99	-99	-99	-99	26
27	-99	-99	-99	-99	-99	-99	-99	-99	-99	-99	-99	-99	-99	-99	-99	-99	-99	-99	-99	-99	-99	27
28	-99	-99	-99	-99	-99	-99	-99	-99	-99	-99	-99	-99	-99	-99	-99	-99	-99	-99	-99	-99	-99	28
29	-99	-99	-99	-99	-99	-99	-99	-99	-99	-99	-99	-99	-99	-99	-99	-99	-99	-99	-99	-99	-99	29
30	-99	-99	-99	-99	-99	-99	-99	-99	-99	-99	-99	-99	-99	-99	-99	-99	-99	-99	-99	-99	-99	30
31	-99	-99	-99	-99	-99	-99	-99	-99	-99	-99	-99	-99	-99	-99	-99	-99	-99	-99	-99	-99	-99	31
32	-99	-99	-99	-99	-99	-99	-99	-99	-99	-99	-99	-99	-99	-99	-99	-99	-99	-99	-99	-99	-99	32
33	3,68138848	0,22833913	0,18858019	0,70771971	0,35459645	1,09524225	14,75620672	4,23760760	0,00001997	0,02848310	0,02791627	0,10190170	0,00617755	0,63739076	2,07519945	0,26947998	0,06760389	0,02117125	0,04170170	0,36608486	0,57484703	33
34	-99	-99	-99	-99	-99	-99	-99	-99	-99	-99	-99	-99	-99	-99	-99	-99	-99	-99	-99	-99	-99	34
35	1,91487471	0,28862136	0,23427095	0,20295756	0,18897273	0,08592668	17,62131885	2,70616536	0,01798889	0,01792665	0,01982205	0,07052360	0,00048432	0,56890119	1,11903100	0,55449640	0,10310956	0,00002118	0,08527402	0,71759911	0,19536582	35
36	-99	-99	-99	-99	-99	-99	-99	-99	-99	-99	-99	-99	-99	-99	-99	-99	-99	-99	-99	-99	-99	36
37	-99	-99	-99	-99	-99	-99	-99	-99	-99	-99	-99	-99	-99	-99	-99	-99	-99	-99	-99	-99	-99	37
38	-99	-99	-99	-99	-99	-99	-99	-99	-99	-99	-99	-99	-99	-99	-99	-99	-99	-99	-99	-99	-99	38
39	-99	-99	-99	-99	-99	-99	-99	-99	-99	-99	-99	-99	-99	-99	-99	-99	-99	-99	-99	-99	-99	39
40	1,55347605	0,01550095	0,17683797	0,49499985	0,34162844	0,74354914	5,85431348	4,22075182	0,15523223	0,04878225	0,03856671	0,17586009	0,01675227	0,48447697	1,58831824	1,18797131	0,09307258	0,02387261	0,03566122	1,19044420	0,20159056	40
41	-99	-99	-99	-99	-99	-99	-99	-99	-99	-99	-99	-99	-99	-99	-99	-99	-99	-99	-99	-99	-99	41
42	-99	-99	-99	-99	-99	-99	-99	-99	-99	-99	-99	-99	-99	-99	-99	-99	-99	-99	-99	-99	-99	42
43	2,93223351	0,21824190	0,16887176	0,42513708	0,38156480	1,28520634	4,95883080	3,91225389	0,11606461	0,02378177	0,05648455	0,10431366	0,00785050	0,60625641	3,43426175	1,14949485	0,05621114	0,01462925	0,01589812	0,22313674	0,36602142	43
44	-99	-99	-99	-99	-99	-99	-99	-99	-99	-99	-99	-99	-99	-99	-99	-99	-99	-99	-99	-99	-99	44
45	-99	-99	-99	-99	-99	-99	-99	-99	-99	-99	-99	-99	-99	-99	-99	-99	-99	-99	-99	-99	-99	45
46	2,83677160	0,15370649	0,06400152	0,29184009	0,17102155	0,07135953	13,94088264	4,50413478	0,00214806	0,01613402	0,00418734	0,14134081	0,00548326	0,37119521	1,44018086	0,74084827	0,07205538	0,01821474	0,04429384	1,10668911	0,48506500	46
47	-99	-99	-99	-99	-99	-99	-99	-99	-99	-99	-99	-99	-99	-99	-99	-99	-99	-99	-99	-99	-99	47
48	-99	-99	-99	-99	-99	-99	-99	-99	-99	-99	-99	-99	-99	-99	-99	-99	-99	-99	-99	-99	-99	48
49	0,60920513	0,30061731	0,23750010	0,72196460	0,19025417	0,55133458	8,51496146	5,23431229	0,00888754	0,35601255	0,01177605	0,06100199	0,00740133	1,06142321	1,42109204	0,46911060	0,24553490	0,03071314	0,00476932	0,80440837	0,22144188	49
50	4,18506491	0,32742809	0,18662964	0,35780613	0,17437317	0,22071399	10,98337856	4,45445830	0,00544539	0,03538422	0,03453612	0,14981459	0,00636003	1,08617006	1,49200255	0,63606672	0,16247053	0,03432135	0,04104215	0,69267476	0,16045604	50
51	1,54247809	0,15275061	0,13738058	0,55066621	0,17902711	0,54346160	15,83725065	2,12615975	0,11058672	0,03069718	0,02230097	0,01080056	0,00557911	0,59925835	1,35495975	0,35954643	0,07190570	0,02807150	0,04607890	0,19754456	0,15359999	51
52	-99	-99	-99	-99	-99	-99	-99	-99	-99	-99	-99	-99	-99	-99	-99	-99	-99	-99	-99	-99	-99	52
53	-99	-99	-99	-99	-99	-99	-99	-99	-99	-99	-99	-99	-99	-99	-99	-99	-99	-99	-99	-99	-99	53
54	-99	-99	-99	-99	-99	-99	-99	-99	-99	-99	-99	-99	-99	-99	-99	-99	-99	-99	-99	-99	-99	54
55	-99	-99	-99	-99	-99	-99	-99	-99	-99	-99	-99	-99	-99	-99	-99	-99	-99	-99	-99	-99	-99	55
56	-99	-99	-99	-99	-99	-99	-99	-99	-99	-99	-99	-99	-99	-99	-99	-99	-99	-99	-99	-99	-99	56
57	3,99930691	0,23000705	0,22636927	0,23483999	0,28474650	0,23176747	9,67156931	1,65950158	0,19207611	0,18386898	0,01051615	0,04455558	0,00861908	0,02609198	1,50185965	0,68956096	0,06371381	0,01714284	0,02636467	0,06776809	0,18943174	57
58	5,44808798	0,38951479	0,68622329	0,45842258	0,18310588	0,87463319	28,81503766	1,98308979	0,17516058	0,81549617	0,06296745	0,06446903	0,00975089	0,74522604	3,08602554	0,55353636	0,21896163	0,02359704	0,03913898	0,25485525	0,09942483	58
59	-99	-99	-99	-99	-99	-99	-99	-99	-99	-99	-99	-99	-99	-99	-99	-99	-99	-99	-99	-99	-99	59
60	-99	-99	-99	-99	-99	-99	-99	-99	-99	-99	-99	-99	-99	-99	-99	-99	-99	-99	-99	-99	-99	60
61	0,39474694	0,16031708	0,18854751	0,37709502	0,14865089	0,18061630	1,18017434	2,59008600	0,07829153	0,31100291	0,03920007	0,08082806	0,00786139	0,36551436	0,88086937	0,29320874	0,10280626	0,01600870	0,03515808	0,40444067	0,13754733	61
62	-99	-99	-99	-99	-99	-99	-99	-99	-99	-99	-99	-99	-99	-99	-99	-99	-99	-99	-99	-99	-99	62
63	-99	-99	-99	-99	-99	-99	-99	-99	-99	-99	-99	-99	-99	-99	-99	-99	-99	-99	-99	-99	-99	63
64	-99	-99	-99	-99	-99	-99	-99	-99	-99	-99	-99	-99	-99	-99	-99	-99	-99	-99	-99	-99	-99	64
65	2,84119923	0,14503771	0,15959640	0,38196173	0,17745191	0,13411105	10,22125188	2,53063598	0,16419704	0,02831087	0,01917672	0,13617181	0,00702396	0,54695726	1,13563562	0,68896376	0,05159947	0,02365846	0,05677895	0,87145616	0,08007524	65
66	-99	-99	-99	-99	-99	-99	-99	-99	-99	-99	-99	-99	-99	-99	-99	-99	-99	-99	-99	-99	-99	66
67	-99	-99	-99	-99	-99	-99	-99	-99	-99	-99	-99	-99	-99	-99	-99	-99	-99	-99	-99	-99	-99	67
68	-99	-99	-99	-99	-99	-99	-99	-99	-99	-99	-99	-99	-99	-99	-99	-99	-99	-99	-99	-99	-99	68
69	-99	-99	-99	-99	-99	-99	-99	-99	-99	-99	-99	-99	-99	-99	-99	-99	-99	-99	-99	-99	-99	69
70	-99	-99	-99	-99	-99	-99	-99	-99	-99	-99	-99	-99	-99	-99	-99	-99	-99	-99	-99	-99	-99	70

71	-99	-99	-99	-99	-99	-99	-99	-99	-99	-99	-99	-99	-99	-99	-99	-99	-99	-99	-99	-99	-99	71
72	-99	-99	-99	-99	-99	-99	-99	-99	-99	-99	-99	-99	-99	-99	-99	-99	-99	-99	-99	-99	-99	72
73	-99	-99	-99	-99	-99	-99	-99	-99	-99	-99	-99	-99	-99	-99	-99	-99	-99	-99	-99	-99	-99	73
74	-99	-99	-99	-99	-99	-99	-99	-99	-99	-99	-99	-99	-99	-99	-99	-99	-99	-99	-99	-99	-99	74
75	3,91089824	0,16533912	0,20103241	0,56350488	0,10817957	0,11037612	14,49516650	2,80402653	0,08534793	0,23463660	0,02051041	0,09824311	0,00915960	0,03851329	1,32914638	0,43271829	0,11838022	0,01462418	0,04754706	0,77458743	0,17093266	75
76	-99	-99	-99	-99	-99	-99	-99	-99	-99	-99	-99	-99	-99	-99	-99	-99	-99	-99	-99	-99	-99	76
77	-99	-99	-99	-99	-99	-99	-99	-99	-99	-99	-99	-99	-99	-99	-99	-99	-99	-99	-99	-99	-99	77
78	-99	-99	-99	-99	-99	-99	-99	-99	-99	-99	-99	-99	-99	-99	-99	-99	-99	-99	-99	-99	-99	78
79	-99	-99	-99	-99	-99	-99	-99	-99	-99	-99	-99	-99	-99	-99	-99	-99	-99	-99	-99	-99	-99	79
80	-99	-99	-99	-99	-99	-99	-99	-99	-99	-99	-99	-99	-99	-99	-99	-99	-99	-99	-99	-99	-99	80
81	-99	-99	-99	-99	-99	-99	-99	-99	-99	-99	-99	-99	-99	-99	-99	-99	-99	-99	-99	-99	-99	81
82	-99	-99	-99	-99	-99	-99	-99	-99	-99	-99	-99	-99	-99	-99	-99	-99	-99	-99	-99	-99	-99	82
83	-99	-99	-99	-99	-99	-99	-99	-99	-99	-99	-99	-99	-99	-99	-99	-99	-99	-99	-99	-99	-99	83
84	0,45007895	0,20058005	0,22488354	0,24987007	0,29427767	0,33570129	4,27004123	6,39635495	0,10469395	0,05792179	0,01439039	0,15990090	0,02046426	0,69411663	2,57264073	1,05062659	0,10001235	0,00601696	0,04576061	0,83349763	0,23329867	84
85	-99	-99	-99	-99	-99	-99	-99	-99	-99	-99	-99	-99	-99	-99	-99	-99	-99	-99	-99	-99	-99	85
86	-99	-99	-99	-99	-99	-99	-99	-99	-99	-99	-99	-99	-99	-99	-99	-99	-99	-99	-99	-99	-99	86
87	3,68457955	0,23414920	0,05138532	0,61450643	0,14544040	0,26379699	2,86691673	4,27078124	0,08971543	0,29310714	0,00998861	0,05654330	0,00104992	0,57454827	1,45145516	0,55844999	0,08475839	0,01225488	0,06039195	0,00000569	0,24375437	87
88	3,39762116	0,17923509	0,19263276	0,31666405	0,26141702	0,12808958	8,52455781	2,94042563	0,11311209	0,25444487	0,00766991	0,07235944	0,00273361	0,47140044	1,46980524	0,32981410	0,23535738	0,00400863	0,00002547	0,22847766	0,39521922	88
89	4,93482868	0,23948380	0,36023244	0,60123462	0,17182359	1,04246576	5,38893431	3,90683412	0,08379454	0,04021841	0,03980242	0,07971527	0,00822938	0,47302882	2,38171370	0,98828565	0,12285257	0,01018084	0,03457804	0,62330060	0,38130041	89
90	-99	-99	-99	-99	-99	-99	-99	-99	-99	-99	-99	-99	-99	-99	-99	-99	-99	-99	-99	-99	-99	90
91	-99	-99	-99	-99	-99	-99	-99	-99	-99	-99	-99	-99	-99	-99	-99	-99	-99	-99	-99	-99	-99	91
92	-99	-99	-99	-99	-99	-99	-99	-99	-99	-99	-99	-99	-99	-99	-99	-99	-99	-99	-99	-99	-99	92
93	-99	-99	-99	-99	-99	-99	-99	-99	-99	-99	-99	-99	-99	-99	-99	-99	-99	-99	-99	-99	-99	93
94	-99	-99	-99	-99	-99	-99	-99	-99	-99	-99	-99	-99	-99	-99	-99	-99	-99	-99	-99	-99	-99	94
95	-99	-99	-99	-99	-99	-99	-99	-99	-99	-99	-99	-99	-99	-99	-99	-99	-99	-99	-99	-99	-99	95
96	-99	-99	-99	-99	-99	-99	-99	-99	-99	-99	-99	-99	-99	-99	-99	-99	-99	-99	-99	-99	-99	96
97	-99	-99	-99	-99	-99	-99	-99	-99	-99	-99	-99	-99	-99	-99	-99	-99	-99	-99	-99	-99	-99	97
98	-99	-99	-99	-99	-99	-99	-99	-99	-99	-99	-99	-99	-99	-99	-99	-99	-99	-99	-99	-99	-99	98
99	2,40452179	0,10480286	0,17735968	0,46065226	0,06545966	0,44557803	8,74676783	2,00520537	0,00704468	0,19114641	0,01787082	0,09302421	0,00584229	0,75667726	0,73496630	0,76088483	0,16640261	0,01312763	0,01455592	0,68101100	0,08471434	99
100	2,24310942	0,14973672	0,20568379	0,37722573	0,15652880	0,04998018	11,71674558	5,63923709	0,00309789	0,50785903	0,02574614	0,02385607	0,00869559	0,47747596	1,46357837	0,38622140	0,13598317	0,02574614	0,07266981	0,76657561	0,04384329	100
101	-99	-99	-99	-99	-99	-99	-99	-99	-99	-99	-99	-99	-99	-99	-99	-99	-99	-99	-99	-99	-99	101
102	-99	-99	-99	-99	-99	-99	-99	-99	-99	-99	-99	-99	-99	-99	-99	-99	-99	-99	-99	-99	-99	102
103	1,10726458	0,30928312	0,28558656	0,29259966	0,16232983	0,10746956	18,29021337	4,25748073	0,11234439	0,03996830	0,02627801	0,12422264	0,00132114	1,19416319	1,11419365	1,07698638	0,12736140	0,01225063	0,05226540	0,76577900	0,11172315	103
104	5,50502837	0,16860860	0,34478048	0,55468861	0,29869604	1,73357470	11,69443292	2,37141803	0,22668330	0,01609493	0,03234642	0,06218671	0,01620688	0,16354392	1,96939159	0,49611621	0,09970088	0,01864264	0,08064618	0,06358146	0,12428723	104
105	-99	-99	-99	-99	-99	-99	-99	-99	-99	-99	-99	-99	-99	-99	-99	-99	-99	-99	-99	-99	-99	105
106	0,51015233	0,20292240	0,15283003	0,40640780	0,12736140	0,66572581	19,21285484	2,53151319	0,08071608	0,21095395	0,00745798	0,12439497	0,00164808	0,60920513	1,75807912	0,79774524	0,12815840	0,02223537	0,05962247	0,98897092	0,20278179	106
107	6,41078044	0,26655405	0,25801020	0,67947859	0,06170367	0,09947653	9,66989350	1,56319743	0,00005015	0,31154231	0,05213876	0,12829172	0,00414762	1,39136410	0,92114489	0,35786814	0,09892644	0,04255581	0,05537966	0,51139147	0,07058473	107
108	-99	-99	-99	-99	-99	-99	-99	-99	-99	-99	-99	-99	-99	-99	-99	-99	-99	-99	-99	-99	-99	108
109	-99	-99	-99	-99	-99	-99	-99	-99	-99	-99	-99	-99	-99	-99	-99	-99	-99	-99	-99	-99	-99	109
110	2,43724283	0,28822153	0,29062888	0,29062888	0,15912694	0,25636123	8,31807677	3,70699457	0,00134727	0,02642871	0,02922304	0,13502047	0,00617755	1,13386588	1,51808229	0,56068019	0,22209518	0,02155625	0,06033964	0,24371214	0,17631780	110
111	-99	-99	-99	-99	-99	-99	-99	-99	-99	-99	-99	-99	-99	-99	-99	-99	-99	-99	-99	-99	-99	111
112	-99	-99	-99	-99	-99	-99	-99	-99	-99	-99	-99	-99	-99	-99	-99	-99	-99	-99	-99	-99	-99	112
113	-99	-99	-99	-99	-99	-99	-99	-99	-99	-99	-99	-99	-99	-99	-99	-99	-99	-99	-99	-99	-99	113
114	4,64281566	0,24081547	0,64171295	0,53551541	0,16379918	0,12024093	7,63696811	2,57397849	0,12439497	0,03103414	0,07683993	0,33448189	0,00879561	0,46329403	1,76785506	1,04246576	0,12107727	0,06448020	0,00000768	0,69688862	0,42720487	114
115	-99	-99	-99	-99	-99	-99	-99	-99	-99	-99	-99	-99	-99	-99	-99	-99	-99	-99	-99	-99	-99	115
116	-99	-99	-99	-99	-99	-99	-99	-99	-99	-99	-99	-99	-99	-99	-99	-99	-99	-99	-99	-99	-99	116
117	-99	-99	-99	-99	-99	-99	-99	-99	-99	-99	-99	-99	-99	-99	-99	-99	-99	-99	-99	-99	-99	117
118	-99	-99	-99	-99	-99	-99	-99	-99	-99	-99	-99	-99	-99	-99	-99	-99	-99	-99	-99	-99	-99	118
119	-99	-99	-99	-99	-99	-99	-99	-99	-99	-99	-99	-99	-99	-99	-99	-99	-99	-99	-99	-99	-99	119
120	3,62818998	0,25725125	0,16796703	0,45132857	0,12151868	0,34850470	14,72555393	5,02195813	0,10425945	0,33968043	0,02734176	0,05917986	0,04332219	0,66215919	1,44818910	0,72610495	0,11472486	0,01504314	0,05176964	0,39921837	0,10861159	120
121	2,65461014	0,19485867	0,21501363	0,32522273	0,06756875	0,48280081	11,20056807	2,93121746	0,24647274	0,01999108	0,05217491	0,09689057	0,00347084	0,25658345	1,75868853	0,23561446	0,11797066	0,01824317	0,05837522	0,39379044	0,08594157	121
122	3,02513599	0,25419367	0,31316611	0,38742797	0,05366034	0,31338326	0,00003062	2,51925996	0,08408545	0,21553589	0,03462601	0,09572237	0,00572702	0,47434216	1,62225431	1,14551790	0,20632635	0,01929004	0,02479168	0,16334566	0,19792147	122
123	-99	-99	-99	-99	-99	-99	-99	-99	-99	-99	-99	-99	-99	-99	-99	-99	-99	-99	-99	-99	-99	123
124	-99	-99	-99	-99	-99	-99	-99	-99	-99	-99	-99	-99	-99	-99	-99	-99	-99	-99	-99	-99	-99	124
125	-99	-99	-99	-99	-99	-99	-99	-99	-99	-99	-99	-99	-99	-99	-99	-99	-99	-99	-99	-99	-99	125
126	-99	-99	-99	-99	-99	-99	-99	-99	-99	-99	-99	-99	-99	-99	-99	-99	-99	-99	-99	-99	-99	126
127	1,31517064	0,15424012	0,19028714	0,82774024	0,26613866	0,69171517	0,36583119	3,08709526	0,11058672	0,28524035	0,00548326	0,25056382	0,01598929	0,63916043	1,78292992	1,12837765	0,11544279	0,02234739	0,02844364	0,15127547	0,24506732	127
128	-99	-99	-99	-99	-99	-99	-99	-99	-99	-99	-99	-99	-99	-99	-99	-99	-99	-99	-99	-99	-99	128
129	-99	-99	-99	-99	-99	-99	-99	-99	-99	-99	-99	-99	-99	-99	-99	-99	-99	-99	-99	-99	-99	129
130	-99	-99	-99	-99	-99	-99	-99	-99	-99	-99	-99	-99	-99	-99	-99	-99	-99	-99	-99	-99	-99	130
131	-99	-99	-99	-99	-99	-99	-99	-99	-99	-99	-99	-99	-99	-99	-99	-99	-99	-99	-99	-99	-99	131
132	-99	-99	-99	-99	-99	-99	-99	-99	-99	-99	-99	-99	-99	-99	-99	-99	-99	-99	-99	-99	-99	132
133	-99	-99	-99	-99	-99	-99	-99	-99	-99	-99	-99	-99	-99	-99	-99	-99	-99	-99	-99	-99	-99	133
134	-99	-99	-99	-99	-99	-99	-99	-99	-99	-99	-99	-99	-99	-99	-99	-99	-99	-99	-99	-99	-99	134
135	-99	-99	-99	-99	-99	-99	-99	-99	-99	-99	-99	-99	-99	-99	-99	-99	-99	-99	-99	-99	-99	135
136	-99	-99	-99	-99	-99	-99	-99	-99	-99	-99	-99	-99	-99	-99	-99	-99	-99	-99	-99	-99	-99	136
137	-99	-99	-99	-99	-99	-99	-99	-99	-99	-99	-99	-99	-99	-99	-99	-99	-99	-99	-99	-99	-99	137
138	6,07548676	0,20208022	0,22546884	0,37035995	0,11446671	0,23536962	35,30987968	3,88522998	0,01429842	0,26590817	0,00568746	0,05783153	0,00427458	0,59666787	2,10234582	0,60878301	0,13425056	0,03386644	0,00262221	0,39474694	0,10273502	138
139	-99	-99	-99	-99	-99	-99	-99	-99	-99	-99	-99	-99	-99	-99	-99	-99	-99	-99	-99	-99	-99	139
140	-99	-99	-99	-99	-99	-99	-99	-99	-99	-99	-99	-99	-99	-99	-99	-99	-99	-99	-99	-99	-99	140
141	3,80527311	0,14813660	0,16817090	0,72749034	0,17410144	0,14289229	9,62642421	3,06311599	0,00001662	0,00804886	0,02321176	0,13985464	0,01257760	0,45754958	1,22688498	0,35948413	0,08379454	0,00518658	0,01395572	0,52668052	0,12293776	141
142	1,11168650	0,12911692	0,26957339	0,91051204	0,19665653	0,62232927	5,52797082	5,62459805	0,14090063	0,05750176	0,07079297	0,40549331	0,01061134	1,15648776	1,69672176	0,63233019	0,13422730	0,03616530	0,12291646	0,69003909	0,13905717	142
143	4,83243390	0,24532225	0,17072545	0,28336824	0,15461476	0,10407893	13,80146969	1,69085155	0,08788434	0,30139972	0,03170271	0,11209159	0,01219768	0,66284801	1,37339822	0,78826357	0,17037080	0,02233964	0,06582366	0,72284088	0,15874136	143
144	-99	-99	-99	-99	-99	-99	-99	-99	-99	-99	-99	-99	-99	-99	-99	-99	-99	-99	-99	-99	-99	144
145	2,44189303	0,34555805	0,16724094	0,37945481	0,10467581	0,12534705	8,89503326	4,50023394	0,06434626	0,37241937	0,03983002	0,31468929	0,00859671	0,66711158	1,84676462	0,64126830	0,21330652	0,02701679	0,09368748	0,76260083	0,30061731	145
146	-99	-99	-99	-99	-99	-99	-99	-99	-99	-99	-99	-99	-99	-99	-99	-99	-99	-99	-99	-99	-99	146
147	6,50703359	0,59336840	0,25560713	0,39721720	0,20632635	0,22344629	36,30257552	2,98142077	0,10013373	0,30735972	0,03363250	0,14721535	0,01760343	0,26407141	1,47222686	0,92274249	0,15690900	0,03479443	0,11003238	0,44135150	0,55825648	147
148	-99	-99	-99	-99	-99	-99	-99	-99	-99	-99	-99	-99	-99	-99	-99	-99	-99	-99	-99	-99	-99	148
149	-99	-99	-99	-99	-99	-99	-99	-99	-99	-99	-99	-99	-99	-99	-99	-99	-99	-99	-99	-99	-99	149
150	-99	-99	-99	-99	-99	-99	-99	-99	-99	-99	-99	-99	-99	-99	-99	-99	-99	-99	-99	-99	-99	150
151	-99	-99	-99	-99	-99	-99	-99	-99	-99	-99	-99	-99	-99	-99	-99	-99	-99	-99	-99	-99	-99	151
152	0,90940825	0,25863690	0,16574072	0,62330060	0,10821707	0,70466038	6,07127701	4,82323131	0,00571908	0,02103594	0,02758447	0,18530838	0,01403332	0,49620219	1,53155800	0,47565914	0,12683282	0,01418982	0,02688602	0,59915451	0,19902202	152
153	-99	-99	-99	-99	-99	-99	-99	-99	-99	-99	-99	-99	-99	-99	-99	-99	-99	-99	-99	-99	-99	153

154	-99	-99	-99	-99	-99	-99	-99	-99	-99	-99	-99	-99	-99	-99	-99	-99	-99	-99	-99	-99	-99	154
155	-99	-99	-99	-99	-99	-99	-99	-99	-99	-99	-99	-99	-99	-99	-99	-99	-99	-99	-99	-99	-99	155
156	-99	-99	-99	-99	-99	-99	-99	-99	-99	-99	-99	-99	-99	-99	-99	-99	-99	-99	-99	-99	-99	156
157	-99	-99	-99	-99	-99	-99	-99	-99	-99	-99	-99	-99	-99	-99	-99	-99	-99	-99	-99	-99	-99	157
158	-99	-99	-99	-99	-99	-99	-99	-99	-99	-99	-99	-99	-99	-99	-99	-99	-99	-99	-99	-99	-99	158
159	2,52319202	0,00277413	0,20211524	0,80176425	0,29427767	1,95375559	13,98928187	5,65783459	0,12641591	0,31869538	0,06548235	0,16531047	0,00257047	0,04694938	1,32431838	0,67373353	0,06173575	0,01421197	0,00006207	0,62905148	0,64898183	159
160	2,86658555	0,19429670	0,30956909	0,83992553	0,24103814	1,27839470	9,99587828	5,80514995	0,00673238	0,02251973	0,04302543	0,60681697	0,03899231	0,00938995	1,87947970	0,81413127	0,09661114	0,02544160	0,10601449	0,42819306	0,13224942	160
161	2,24855785	0,00001677	0,18556545	0,62373279	0,21703506	0,99792272	12,33768660	5,62167480	0,11320425	0,26242917	0,03043763	0,10503922	0,00009704	0,02834032	1,14234625	0,53737459	0,08962220	0,00878952	0,00613807	0,19710004	0,45375958	161
162	-99	-99	-99	-99	-99	-99	-99	-99	-99	-99	-99	-99	-99	-99	-99	-99	-99	-99	-99	-99	-99	162
163	-99	-99	-99	-99	-99	-99	-99	-99	-99	-99	-99	-99	-99	-99	-99	-99	-99	-99	-99	-99	-99	163
164	6,19347888	0,26734044	0,32527909	0,28832143	0,18399647	0,20078870	6,11668508	0,28199666	0,08554041	0,27428545	0,01535391	0,03517636	0,00662323	0,52513127	1,48118311	0,41573900	0,21805289	0,00002800	0,06753363	0,31616509	0,01981518	164
165	-99	-99	-99	-99	-99	-99	-99	-99	-99	-99	-99	-99	-99	-99	-99	-99	-99	-99	-99	-99	-99	165
166	-99	-99	-99	-99	-99	-99	-99	-99	-99	-99	-99	-99	-99	-99	-99	-99	-99	-99	-99	-99	-99	166
167	2,75585503	0,28038868	0,27385801	0,33973930	0,24240645	0,85352185	0,53310810	4,32739856	0,23044590	0,04761302	0,02461190	0,10100510	0,00206377	0,41768869	1,11149388	1,39040001	0,16683571	0,01781826	0,11014684	0,68800931	0,22837870	167
168	-99	-99	-99	-99	-99	-99	-99	-99	-99	-99	-99	-99	-99	-99	-99	-99	-99	-99	-99	-99	-99	168
169	-99	-99	-99	-99	-99	-99	-99	-99	-99	-99	-99	-99	-99	-99	-99	-99	-99	-99	-99	-99	-99	169
170	-99	-99	-99	-99	-99	-99	-99	-99	-99	-99	-99	-99	-99	-99	-99	-99	-99	-99	-99	-99	-99	170
171	7,78663499	0,27243803	0,19778433	0,33240178	0,09827716	0,10482102	13,04115732	5,13726324	0,13898489	0,01574459	0,04759652	0,04413295	0,00221113	0,60374030	2,01810327	0,55478474	0,25809963	0,01779974	0,05013632	0,58601114	0,31819873	171
172	1,79813333	0,30513086	0,21561060	0,63003331	0,07404263	0,92050662	13,43104823	2,03848876	0,00487122	0,02514648	0,03442260	0,15373313	0,00516327	0,59193062	1,40980927	0,47122892	0,06450255	0,02963611	0,03666380	0,84527929	0,13244819	172
173	-99	-99	-99	-99	-99	-99	-99	-99	-99	-99	-99	-99	-99	-99	-99	-99	-99	-99	-99	-99	-99	173
174	5,45092096	0,10698645	0,27423792	0,46474138	0,17259948	1,38367009	5,98146965	1,84228977	0,00001354	0,26729412	0,04288152	0,07596612	0,01203600	0,59480967	2,19847023	0,87206042	0,15555536	0,02932958	0,05642585	0,32342430	0,23140629	174
175	4,19377657	0,24540729	0,28014584	0,83871351	0,16960493	0,22897310	28,50711230	3,30637417	0,13151051	0,02095226	0,04231315	0,16123647	0,00580395	0,10105762	1,25897532	0,38857096	0,10022053	0,04429384	0,00203501	0,84337724	0,20895295	175
176	3,53571012	0,13621901	0,19560295	0,67548704	0,11095142	0,29792064	31,03878113	3,88253788	0,10111017	0,31057207	0,01033728	0,06346038	0,00935530	0,44104568	1,09961615	0,35973340	0,15954110	0,01079121	0,00010152	0,47929972	0,27130734	176
177	2,46058269	0,13937078	0,21673439	0,40025741	0,08379454	0,12949785	6,32909841	2,45717396	0,03321549	0,22251893	0,02284465	0,06848824	0,00908688	0,54299093	1,40444488	0,51192345	0,09930430	0,02037579	0,02441650	0,77003717	0,14131632	177
178	-99	-99	-99	-99	-99	-99	-99	-99	-99	-99	-99	-99	-99	-99	-99	-99	-99	-99	-99	-99	-99	178
179	-99	-99	-99	-99	-99	-99	-99	-99	-99	-99	-99	-99	-99	-99	-99	-99	-99	-99	-99	-99	-99	179
180	-99	-99	-99	-99	-99	-99	-99	-99	-99	-99	-99	-99	-99	-99	-99	-99	-99	-99	-99	-99	-99	180
181	-99	-99	-99	-99	-99	-99	-99	-99	-99	-99	-99	-99	-99	-99	-99	-99	-99	-99	-99	-99	-99	181
182	2,15397275	0,27433298	0,21718555	0,50662845	0,25139015	0,61813763	7,56321693	3,66293475	0,25702846	0,00953864	0,07065816	0,20891674	0,00670754	1,08222464	1,46307122	0,48971015	0,17604303	0,01739721	0,21345443	0,49723508	0,48599049	182
183	-99	-99	-99	-99	-99	-99	-99	-99	-99	-99	-99	-99	-99	-99	-99	-99	-99	-99	-99	-99	-99	183
184	-99	-99	-99	-99	-99	-99	-99	-99	-99	-99	-99	-99	-99	-99	-99	-99	-99	-99	-99	-99	-99	184
185	-99	-99	-99	-99	-99	-99	-99	-99	-99	-99	-99	-99	-99	-99	-99	-99	-99	-99	-99	-99	-99	185
186	-99	-99	-99	-99	-99	-99	-99	-99	-99	-99	-99	-99	-99	-99	-99	-99	-99	-99	-99	-99	-99	186
187	-99	-99	-99	-99	-99	-99	-99	-99	-99	-99	-99	-99	-99	-99	-99	-99	-99	-99	-99	-99	-99	187
188	-99	-99	-99	-99	-99	-99	-99	-99	-99	-99	-99	-99	-99	-99	-99	-99	-99	-99	-99	-99	-99	188
189	2,93324993	0,24307948	0,35960874	0,28331915	0,12204626	0,26747946	21,72824881	2,91905218	0,01129243	0,46377598	0,03376097	0,10551354	0,00248507	0,63661806	0,96761162	0,99068616	0,09642159	0,01585960	0,02267899	0,38461848	0,09346046	189
190	-99	-99	-99	-99	-99	-99	-99	-99	-99	-99	-99	-99	-99	-99	-99	-99	-99	-99	-99	-99	-99	190
191	-99	-99	-99	-99	-99	-99	-99	-99	-99	-99	-99	-99	-99	-99	-99	-99	-99	-99	-99	-99	-99	191
192	-99	-99	-99	-99	-99	-99	-99	-99	-99	-99	-99	-99	-99	-99	-99	-99	-99	-99	-99	-99	-99	192
193	-99	-99	-99	-99	-99	-99	-99	-99	-99	-99	-99	-99	-99	-99	-99	-99	-99	-99	-99	-99	-99	193
194	-99	-99	-99	-99	-99	-99	-99	-99	-99	-99	-99	-99	-99	-99	-99	-99	-99	-99	-99	-99	-99	194
195	-99	-99	-99	-99	-99	-99	-99	-99	-99	-99	-99	-99	-99	-99	-99	-99	-99	-99	-99	-99	-99	195
196	-99	-99	-99	-99	-99	-99	-99	-99	-99	-99	-99	-99	-99	-99	-99	-99	-99	-99	-99	-99	-99	196
197	-99	-99	-99	-99	-99	-99	-99	-99	-99	-99	-99	-99	-99	-99	-99	-99	-99	-99	-99	-99	-99	197
198	3,46775025	0,20208022	0,23068562	0,61429349	0,24707141	0,41293883	16,12246017	3,78948028	0,00170620	0,28538868	0,02802775	0,20152071	0,02798893	0,82759682	1,55724938	0,93174043	0,15198491	0,02179287	0,05172480	1,22858698	0,28204553	198
199	6,04607971	0,29874780	0,21360244	0,29178953	0,15041288	1,28966825	15,71423352	3,42475314	0,18415596	0,00735020	0,04106349	0,11203334	0,00234045	0,06707877	1,52308874	0,77378250	0,09375244	0,01822421	0,01199020	0,23116582	0,17974207	199
200	-99	-99	-99	-99	-99	-99	-99	-99	-99	-99	-99	-99	-99	-99	-99	-99	-99	-99	-99	-99	-99	200
201	-99	-99	-99	-99	-99	-99	-99	-99	-99	-99	-99	-99	-99	-99	-99	-99	-99	-99	-99	-99	-99	201
202	2,65921422	0,03203960	0,07320064	0,35551935	0,21345443	1,00625782	3,19319355	3,57761961	0,04694125	0,28717459	0,01549557	0,21271593	0,01049612	0,93562350	1,62788637	0,62981499	0,17702193	0,01634508	0,13956412	0,52015013	0,19304378	202
203	0,73713461	0,31100291	0,08711108	0,41208104	0,10174290	0,25139015	9,08193682	5,55582054	0,08218392	0,42454812	0,02942120	0,12007436	0,00799326	0,29381908	1,56049096	0,72749034	0,09889216	0,00190367	0,01709834	0,66989180	0,27262693	203
204	-99	-99	-99	-99	-99	-99	-99	-99	-99	-99	-99	-99	-99	-99	-99	-99	-99	-99	-99	-99	-99	204
205	-99	-99	-99	-99	-99	-99	-99	-99	-99	-99	-99	-99	-99	-99	-99	-99	-99	-99	-99	-99	-99	205
206	-99	-99	-99	-99	-99	-99	-99	-99	-99	-99	-99	-99	-99	-99	-99	-99	-99	-99	-99	-99	-99	206
207	-99	-99	-99	-99	-99	-99	-99	-99	-99	-99	-99	-99	-99	-99	-99	-99	-99	-99	-99	-99	-99	207
208	-99	-99	-99	-99	-99	-99	-99	-99	-99	-99	-99	-99	-99	-99	-99	-99	-99	-99	-99	-99	-99	208
209	1,96768598	0,18588729	0,31567239	0,32074529	0,40430053	0,37462004	18,28387556	4,36656858	0,16857938	0,05231977	0,03357427	0,01604758	0,00394844	0,62135945	1,76846786	0,75812098	0,20525653	0,03447036	0,02835015	0,59070102	0,09736182	209
210	-99	-99	-99	-99	-99	-99	-99	-99	-99	-99	-99	-99	-99	-99	-99	-99	-99	-99	-99	-99	-99	210
211	-99	-99	-99	-99	-99	-99	-99	-99	-99	-99	-99	-99	-99	-99	-99	-99	-99	-99	-99	-99	-99	211
212	0,34746751	0,15919313	0,15754654	0,48432588	0,16433936	0,49371487	5,03136551	4,90907089	0,13321699	0,02447156	0,01860134	0,03157989	0,00794548	0,03377852	0,98503712	0,41568137	0,06772114	0,00412197	0,04964355	0,20155563	0,17964865	212
213	-99	-99	-99	-99	-99	-99	-99	-99	-99	-99	-99	-99	-99	-99	-99	-99	-99	-99	-99	-99	-99	213
214	4,58445425	0,32867873	0,22778584	0,36927052	0,21062521	0,19981683	31,77345346	3,22153887	0,18553329	0,24345888	0,01269804	0,06948041	0,00254477	0,62405712	1,51966150	0,32035646	0,09308871	0,02027365	0,00391099	0,39032560	0,21400998	214
215	-99	-99	-99	-99	-99	-99	-99	-99	-99	-99	-99	-99	-99	-99	-99	-99	-99	-99	-99	-99	-99	215
216	6,37753981	0,14539001	0,21183311	0,41094009	0,14299137	0,11924494	3,37760355	3,35194854	0,10927233	0,30650872	0,01868468	0,04129183	0,00678235	0,52741116	2,10818285	0,92916067	0,13231055	0,01695671	0,02591624	0,82131070	0,22893342	216
217	-99	-99	-99	-99	-99	-99	-99	-99	-99	-99	-99	-99	-99	-99	-99	-99	-99	-99	-99	-99	-99	217
218	-99	-99	-99	-99	-99	-99	-99	-99	-99	-99	-99	-99	-99	-99	-99	-99	-99	-99	-99	-99	-99	218
219	-99	-99	-99	-99	-99	-99	-99	-99	-99	-99	-99	-99	-99	-99	-99	-99	-99	-99	-99	-99	-99	219
220	2,47040915	0,26494228	0,24127494	0,21460417	0,19597615	0,25984983	10,26562813	3,62190825	0,11897662	0,19692934	0,03094285	0,01620688	0,00065362	0,77579641	1,42281688	0,07822372	0,12283129	0,00983234	0,02885070	0,04212294	0,06253250	220
221	3,73730939	0,17739042	0,21613431	0,49277467	0,07726720	0,58967830	37,27147477	4,10960576	0,09433912	0,35601255	0,04322471	0,16199262	0,00792705	1,14155471	1,49796093	0,48733982	0,10904534	0,02317961	0,01143024	0,27700809	0,25899569	221
222	2,05729688	0,19502757	0,22387262	0,38017880	0,18133760	0,56165262	6,99221720	4,68727682	0,01045075	0,02656646	0,04391172	0,09048046	0,00162398	0,26457525	1,28320352	0,58631587	0,07860419	0,01028546	0,02191025	0,52951740	0,22984768	222
223	2,35585378	0,32856484	0,21662175	0,72409455	0,23836594	1,39692058	18,79464812	6,28646940	0,09494593	0,16303459	0,04924940	0,18089821	0,01169674	0,22992735	2,19504421	1,06825051	0,10454892	0,01615640	0,13473999	0,33107959	0,30826650	223
224	-99	-99	-99	-99	-99	-99	-99	-99	-99	-99	-99	-99	-99	-99	-99	-99	-99	-99	-99	-99	-99	224
225	3,53387253	0,18291560	0,14981459	0,50812311	0,23394641	0,53970763	9,85769265	3,26537798	0,09640488	0,00598783	0,06259755	0,63518555	0,01044713	0,69027828	2,08240402	0,66491877	0,07744146	0,03612772	0,04969519	0,84865495	0,25073755	225
226	0,68669911	0,33675003	0,15852145	0,38044241	0,21062521	0,74059155	0,32370465	5,22615531	0,00626595	0,03096431	0,04831952	0,14376157	0,00804746	0,99705846	1,78231211	0,80482666	0,06266267	0,04735794	0,11750141	0,91620986	0,22542978	226
227	-99	-99	-99	-99	-99	-99	-99	-99	-99	-99	-99	-99	-99	-99	-99	-99	-99	-99	-99	-99	-99	227
228	-99	-99	-99	-99	-99	-99	-99	-99	-99	-99	-99	-99	-99	-99	-99	-99	-99	-99	-99	-99	-99	228
229	-99	-99	-99	-99	-99	-99	-99	-99	-99	-99	-99	-99	-99	-99	-99	-99	-99	-99	-99	-99	-99	229
230	-99	-99	-99	-99	-99	-99	-99	-99	-99	-99	-99	-99	-99	-99	-99	-99	-99	-99	-99	-99	-99	230
231	-99	-99	-99	-99	-99	-99	-99	-99	-99	-99	-99	-99	-99	-99	-99	-99	-99	-99	-99	-99	-99	231
232	2,26812476	0,14156142	0,25112891	0,22539072	0,21816627	0,13711896	7,45650722	2,66013599	0,00002661	0,03362085	0,02283673	0,09502823	0,00716161	0,86904332	1,38943659	0,26840808	0,13153330	0,01218711	0,01861359	0,58865735	0,17794459	232
233	6,07759273	0,14165957	0,25998495	0,46377598	0,17069586	0,07122364	12,41919709	1,86025449	0,18168359	0,02129266	0,01905746	0,21650916	0,00461805	0,48179790	1,15069062	1,16999308	0,12340733	0,02342999	0,09077885	0,99896082	0,21175971	233
234	2,00972764	0,14034018	0,21809068	0,49482833	0,17301871	1,21672236	13,00504963	5,43771297	0,11990801	0,22531262	0,05609438	0,08038109	0,01101795	0,06978206	0,96727633	0,69447757	0,12175053	0,02705427	0,01678132	1,22264028	0,09150536	234
235	1,98377719	0,09673957	0,12450279	0,55835323	0,11885298	0,22754913	14,48261283	5,48788405	0,18165211	0,22040823	0,03329040	0,05864899	0,00775450	1,05573665	1,24941261	0,44142799	0,06135182	0,00325134	0,01342671	0,31300335	0,13814450	235
236	-99	-99	-99	-99	-99	-99	-99	-99	-99	-99	-99	-99	-99	-99	-99	-99	-99	-99	-99	-99	-99	236

237	-99	-99	-99	-99	-99	-99	-99	-99	-99	-99	-99	-99	-99	-99	-99	-99	-99	-99	-99	-99	-99	237
238	-99	-99	-99	-99	-99	-99	-99	-99	-99	-99	-99	-99	-99	-99	-99	-99	-99	-99	-99	-99	-99	238
239	-99	-99	-99	-99	-99	-99	-99	-99	-99	-99	-99	-99	-99	-99	-99	-99	-99	-99	-99	-99	-99	239
240	-99	-99	-99	-99	-99	-99	-99	-99	-99	-99	-99	-99	-99	-99	-99	-99	-99	-99	-99	-99	-99	240
241	-99	-99	-99	-99	-99	-99	-99	-99	-99	-99	-99	-99	-99	-99	-99	-99	-99	-99	-99	-99	-99	241
242	-99	-99	-99	-99	-99	-99	-99	-99	-99	-99	-99	-99	-99	-99	-99	-99	-99	-99	-99	-99	-99	242
243	2,98400510	0,10906424	0,22441640	0,38163093	0,13662089	0,47673188	17,63353725	4,02015186	0,00324684	0,00559848	0,02963098	0,08474370	0,00427236	0,55951550	2,37388492	0,34275509	0,14311532	0,01692442	0,01567110	0,02768503	0,28189895	243
244	3,14759517	0,25265664	0,29529933	0,60259057	0,27419041	0,07290947	9,06464173	3,04248468	0,14321455	0,01509537	0,02030882	0,03605267	0,01314128	1,07642664	1,44417943	0,40199515	0,14055922	0,01092858	0,03572926	0,51557349	0,18805805	244
245	-99	-99	-99	-99	-99	-99	-99	-99	-99	-99	-99	-99	-99	-99	-99	-99	-99	-99	-99	-99	-99	245
246	4,23173710	0,32993415	0,09183895	0,50286742	0,08340340	0,15117065	6,02203044	4,93568389	0,08236927	0,02345843	0,02294383	0,14971078	0,00157903	0,04611085	1,36627699	0,59470660	0,16034486	0,01083806	0,07787208	0,63606672	0,20536326	246
247	-99	-99	-99	-99	-99	-99	-99	-99	-99	-99	-99	-99	-99	-99	-99	-99	-99	-99	-99	-99	-99	247
248	2,82401940	0,42418044	0,27657641	0,43519986	0,12778142	0,19912552	9,35508006	3,29836258	0,00024802	0,35743430	0,02384367	0,05182349	0,00341654	0,02309541	1,22412425	0,69003909	0,16241424	0,01552514	0,00330473	0,39852717	0,08812835	248
249	0,69279480	0,33973930	0,26785052	0,39873441	0,15077823	0,27557177	7,33857526	4,50803901	0,09084179	0,01679878	0,00488814	0,19771579	0,00283095	0,62568135	1,92185568	0,49844285	0,09968360	0,00923610	0,00148482	0,31111072	0,07821017	249
250	-99	-99	-99	-99	-99	-99	-99	-99	-99	-99	-99	-99	-99	-99	-99	-99	-99	-99	-99	-99	-99	250
251	2,46784195	0,32516638	0,24002390	0,37068098	0,19754456	0,02685343	0,67701045	2,63947317	0,07819662	0,06290201	0,02106877	0,04588766	0,02131850	0,62384088	1,33723220	0,98845692	0,18102364	0,02145191	0,01820212	0,43686214	0,10098760	251
252	-99	-99	-99	-99	-99	-99	-99	-99	-99	-99	-99	-99	-99	-99	-99	-99	-99	-99	-99	-99	-99	252
253	-99	-99	-99	-99	-99	-99	-99	-99	-99	-99	-99	-99	-99	-99	-99	-99	-99	-99	-99	-99	-99	253
254	-99	-99	-99	-99	-99	-99	-99	-99	-99	-99	-99	-99	-99	-99	-99	-99	-99	-99	-99	-99	-99	254
255	-99	-99	-99	-99	-99	-99	-99	-99	-99	-99	-99	-99	-99	-99	-99	-99	-99	-99	-99	-99	-99	255
256	1,63750578	0,16545376	0,17271916	0,27385801	0,07353118	0,02379002	0,91795797	2,54823795	0,16115267	0,23220968	0,03055389	0,02435733	0,00204243	0,18796032	1,02420180	0,51068302	0,14146333	0,00764377	0,02015805	0,19922906	0,03534133	256
257	-99	-99	-99	-99	-99	-99	-99	-99	-99	-99	-99	-99	-99	-99	-99	-99	-99	-99	-99	-99	-99	257
258	-99	-99	-99	-99	-99	-99	-99	-99	-99	-99	-99	-99	-99	-99	-99	-99	-99	-99	-99	-99	-99	258
259	3,81782242	0,25520880	0,24668639	0,75615296	0,10307383	0,08530357	15,93083521	1,93287722	0,09225366	0,35793016	0,08673451	0,37598578	0,01633092	1,75290766	1,04662890	1,05976912	0,14872819	0,02915223	0,03697644	0,42066684	0,29356462	259
260	-99	-99	-99	-99	-99	-99	-99	-99	-99	-99	-99	-99	-99	-99	-99	-99	-99	-99	-99	-99	-99	260
261	0,61015598	0,20607623	0,16948741	0,24780033	0,23216944	0,44357501	16,50979155	3,09352137	0,04942038	0,02398456	0,03499397	0,07579518	0,01379462	0,52240840	1,40761227	0,91462357	0,10425945	0,00733112	0,07003646	0,10704208	0,25547429	261
262	3,04828966	0,14488699	0,24810109	0,35551935	0,24587552	0,31643915	5,44903214	3,46294626	0,08820474	0,02351948	0,03794359	0,12116123	0,01410157	1,09809281	1,86606598	0,41322516	0,07284632	0,02097770	0,07489431	0,29219431	0,31014182	262
263	-99	-99	-99	-99	-99	-99	-99	-99	-99	-99	-99	-99	-99	-99	-99	-99	-99	-99	-99	-99	-99	263
264	2,22645739	0,14965890	0,15709945	0,32936291	0,20037161	0,20902538	17,16918913	1,08956329	0,08880286	0,01121637	0,00628335	0,02853250	0,00179942	0,51610982	1,31380394	0,44219358	0,23877935	0,01191357	0,00004168	0,37886348	0,13581832	264
265	3,51311304	0,21926539	0,23337954	0,34959344	0,17674606	0,24702860	5,08324582	1,57107276	0,13953994	0,03279796	0,01988397	0,02424364	0,00804189	0,07410681	1,43172062	0,39331306	0,09484727	0,01478984	0,03358009	0,01386652	0,17662360	265

MPI_FGH_Co [61711]																						Location_ Treatment
15	4	50	32	31	28	19	26	5	36	20	29	14	30	9	13	2	18	16	10	7	34	Ranking
Kinase	Receptor_ protein_kinase	Reticuline_ oxidase	Serine- threonine_ protein_kinase _plant-type	TMV_ resistance_ protein_N	Homoserine_d ehydrogenase _family_protei n	Cc-nbs-lrr resistance protein	UDP-glucose: glucosyltrans ferase	Methionyl- tRNA_ synthetase	70_kDa_ subunit_of_ replication_ protein_A	Gene_of_ unknown_ function	RING_finger_ protein	Flagellin- sensing 2	Serine- threonine protein kinase, plant-type	TIR-NBS- LRR_disease_ resistance	LRR receptor- like serine/ threonine- protein kinase	Poly(ADP- ribose)_glyco hydrolase	Bacterial_spot _disease_ resistance_ protein_4	Extensin	Lipoxygenase	Betaine_ aldehyde_ dehyrogenase	Fatty_acid_ desaturase	Gene product
PGSC0003 DMT400042362	PGSC0003 DMT400045689	PGSC0003 DMT40004630	PGSC0003 DMT40004644	PGSC0003 DMT40004689	PGSC0003 DMT40004811	PGSC0003 DMT40004909	PGSC0003 DMT40004912	PGSC0003 DMT40004917	PGSC0003 DMT40005251	PGSC0003 DMT40006237	PGSC0003 DMT40006687	PGSC0003 DMT40006877	PGSC0003 DMT40006878	PGSC0003 DMT4000714	PGSC0003 DMT40007188	PGSC0003 DMT40007551	PGSC0003 DMT40008081	PGSC0003 DMT40008201	PGSC0003 DMT40008202	PGSC0003 DMT40008302	PGSC0003 DMT40008385	Transcript ID F1-Individuals
0,08903399	0,02334893	6,20529587	0,11970041	0,02870608	0,11083612	0,48750874	1,84228977	0,06014131	0,77405072	0,00051025	1,15548618	0,05943679	0,04564182	0,76923697	0,14921867	0,36920654	0,19244259	8,95379942	3,63133493	0,75707073	0,06686986	1
-99	-99	-99	-99	-99	-99	-99	-99	-99	-99	-99	-99	-99	-99	-99	-99	-99	-99	-99	-99	-99	-99	2
-99	-99	-99	-99	-99	-99	-99	-99	-99	-99	-99	-99	-99	-99	-99	-99	-99	-99	-99	-99	-99	-99	3
-99	-99	-99	-99	-99	-99	-99	-99	-99	-99	-99	-99	-99	-99	-99	-99	-99	-99	-99	-99	-99	-99	4
-99	-99	-99	-99	-99	-99	-99	-99	-99	-99	-99	-99	-99	-99	-99	-99	-99	-99	-99	-99	-99	-99	5
0,05645519	0,04636725	4,16480803	0,02318363	0,06059111	0,03850661	0,26576997	1,20936522	0,08527402	0,09966633	0,04344247	2,04946876	0,02514212	0,00964001	0,15824699	0,08908029	0,71859460	0,03136393	2,58694611	3,02775821	0,32292029	0,07475169	6
-99	-99	-99	-99	-99	-99	-99	-99	-99	-99	-99	-99	-99	-99	-99	-99	-99	-99	-99	-99	-99	-99	7
-99	-99	-99	-99	-99	-99	-99	-99	-99	-99	-99	-99	-99	-99	-99	-99	-99	-99	-99	-99	-99	-99	8
-99	-99	-99	-99	-99	-99	-99	-99	-99	-99	-99	-99	-99	-99	-99	-99	-99	-99	-99	-99	-99	-99	9
-99	-99	-99	-99	-99	-99	-99	-99	-99	-99	-99	-99	-99	-99	-99	-99	-99	-99	-99	-99	-99	-99	10
-99	-99	-99	-99	-99	-99	-99	-99	-99	-99	-99	-99	-99	-99	-99	-99	-99	-99	-99	-99	-99	-99	11
-99	-99	-99	-99	-99	-99	-99	-99	-99	-99	-99	-99	-99	-99	-99	-99	-99	-99	-99	-99	-99	-99	12
-99	-99	-99	-99	-99	-99	-99	-99	-99	-99	-99	-99	-99	-99	-99	-99	-99	-99	-99	-99	-99	-99	13
-99	-99	-99	-99	-99	-99	-99	-99	-99	-99	-99	-99	-99	-99	-99	-99	-99	-99	-99	-99	-99	-99	14
-99	-99	-99	-99	-99	-99	-99	-99	-99	-99	-99	-99	-99	-99	-99	-99	-99	-99	-99	-99	-99	-99	15
0,05423056	0,17173429	3,01728296	0,00200143	0,05260158	0,01802634	0,46692088	0,37820753	0,02872100	0,02120062	0,01443035	1,09448334	0,04181748	0,04011401	1,01273028	0,15748103	0,47376713	0,17619562	7,17634874	6,38749386	0,41617148	0,06736999	16
0,02619164	0,26411717	1,96053854	0,15857640	0,06580085	0,14981459	0,13087396	1,01343250	0,14877974	0,00599614	0,03475225	2,19656623	0,10425945	0,10289537	0,91145921	0,43444637	0,93513723	0,01744854	5,38613354	8,71348592	0,47180088	0,24421945	17
0,09588839	0,03967846	3,36941936	0,26361420	0,05841570	0,09709225	0,39873441	0,95495192	0,05435287	0,00810765	0,01348501	1,68587784	0,15448086	0,08464097	0,15533986	0,60815037	0,65270364	0,03965097	2,55177301	4,27670590	0,37125954	0,23791201	18
0,08759547	0,11825721	5,39641014	0,05925169	0,03455408	0,10562330	0,38050835	0,95660816	0,02905640	0,50768305	0,01628853	0,80162533	0,04677884	0,03228482	0,17051848	0,19117953	0,45313097	0,07075618	10,57070725	7,78123958	0,70027816	0,11336129	19
-99	-99	-99	-99	-99	-99	-99	-99	-99	-99	-99	-99	-99	-99	-99	-99	-99	-99	-99	-99	-99	-99	20
-99	-99	-99	-99	-99	-99	-99	-99	-99	-99	-99	-99	-99	-99	-99	-99	-99	-99	-99	-99	-99	-99	21
-99	-99	-99	-99	-99	-99	-99	-99	-99	-99	-99	-99	-99	-99	-99	-99	-99	-99	-99	-99	-99	-99	22
-99	-99	-99	-99	-99	-99	-99	-99	-99	-99	-99	-99	-99	-99	-99	-99	-99	-99	-99	-99	-99	-99	23
0,06182139	0,05426817	4,93568389	0,05066910	0,07874052	0,11291038	0,15542064	1,28009422	0,03688684	0,11043352	0,04158624	0,70821043	0,02797438	0,01480522	0,23362232	0,26892019	0,38136649	0,12093050	5,05338466	1,84708467	0,44790044	0,15033470	24
-99	-99	-99	-99	-99	-99	-99	-99	-99	-99	-99	-99	-99	-99	-99	-99	-99	-99	-99	-99	-99	-99	25
-99	-99	-99	-99	-99	-99	-99	-99	-99	-99	-99	-99	-99	-99	-99	-99	-99	-99	-99	-99	-99	-99	26
-99	-99	-99	-99	-99	-99	-99	-99	-99	-99	-99	-99	-99	-99	-99	-99	-99	-99	-99	-99	-99	-99	27
-99	-99	-99	-99	-99	-99	-99	-99	-99	-99	-99	-99	-99	-99	-99	-99	-99	-99	-99	-99	-99	-99	28
-99	-99	-99	-99	-99	-99	-99	-99	-99	-99	-99	-99	-99	-99	-99	-99	-99	-99	-99	-99	-99	-99	29
-99	-99	-99	-99	-99	-99	-99	-99	-99	-99	-99	-99	-99	-99	-99	-99	-99	-99	-99	-99	-99	-99	30
-99	-99	-99	-99	-99	-99	-99	-99	-99	-99	-99	-99	-99	-99	-99	-99	-99	-99	-99	-99	-99	-99	31
-99	-99	-99	-99	-99	-99	-99	-99	-99	-99	-99	-99	-99	-99	-99	-99	-99	-99	-99	-99	-99	-99	32
0,08634461	0,08622500	2,02546057	0,02996142	0,03233521	0,11213045	0,28862136	1,82039307	0,03142921	0,45478293	0,01551976	0,95346375	0,05525504	0,01169674	0,27840364	0,21026054	0,50671625	0,08492011	2,75346830	4,85089180	0,26595425	0,08456767	33
-99	-99	-99	-99	-99	-99	-99	-99	-99	-99	-99	-99	-99	-99	-99	-99	-99	-99	-99	-99	-99	-99	34
0,10556840	0,09455188	2,84267664	0,04028119	0,09070023	0,06484998	0,42110444	1,37768876	0,07658735	0,14351267	0,00053146	1,08016372	0,04141365	0,03640423	0,57048071	0,13399490	0,30741298	0,15033470	3,18490425	3,65849427	0,24797215	0,08761065	35
-99	-99	-99	-99	-99	-99	-99	-99	-99	-99	-99	-99	-99	-99	-99	-99	-99	-99	-99	-99	-99	-99	36
-99	-99	-99	-99	-99	-99	-99	-99	-99	-99	-99	-99	-99	-99	-99	-99	-99	-99	-99	-99	-99	-99	37
-99	-99	-99	-99	-99	-99	-99	-99	-99	-99	-99	-99	-99	-99	-99	-99	-99	-99	-99	-99	-99	-99	38
-99	-99	-99	-99	-99	-99	-99	-99	-99	-99	-99	-99	-99	-99	-99	-99	-99	-99	-99	-99	-99	-99	39
0,05473092	0,06747515	1,31084763	0,07466107	0,07864506	0,34091878	0,28233894	1,42059962	0,07034053	0,21000564	0,02169490	2,06837819	0,06956474	0,05869982	0,22259607	0,16916465	0,48280081	0,15151158	1,98825114	4,53310632	0,33529433	0,11285170	40
-99	-99	-99	-99	-99	-99	-99	-99	-99	-99	-99	-99	-99	-99	-99	-99	-99	-99	-99	-99	-99	-99	41
-99	-99	-99	-99	-99	-99	-99	-99	-99	-99	-99	-99	-99	-99	-99	-99	-99	-99	-99	-99	-99	-99	42
0,07376090	0,11999116	2,16444929	0,00870464	0,05474989	0,17640948	0,11574325	1,23713248	0,11858554	0,19888412	0,02474018	1,07251662	0,02841901	0,02983191	0,17677670	0,11226655	0,90689033	0,11118237	1,42503761	1,16715810	0,42631745	0,09632139	43
-99	-99	-99	-99	-99	-99	-99	-99	-99	-99	-99	-99	-99	-99	-99	-99	-99	-99	-99	-99	-99	-99	44
-99	-99	-99	-99	-99	-99	-99	-99	-99	-99	-99	-99	-99	-99	-99	-99	-99	-99	-99	-99	-99	-99	45
0,06612089	0,08404175	1,65433339	0,09448637	0,04959196	0,06177856	0,13334633	1,54568892	0,05355815	0,42876231	0,00001543	0,76432070	0,03950694	0,00488729	0,03745365	0,23590044	0,19618002	0,16519592	4,17347752	6,39192287	0,40032677	0,10368291	46
-99	-99	-99	-99	-99	-99	-99	-99	-99	-99	-99	-99	-99	-99	-99	-99	-99	-99	-99	-99	-99	-99	47
-99	-99	-99	-99	-99	-99	-99	-99	-99	-99	-99	-99	-99	-99	-99	-99	-99	-99	-99	-99	-99	-99	48
0,07484242	0,10746956	7,52660886	0,11454608	0,01617601	0,14269434	0,08094019	0,86573657	0,09539120	1,20748059	0,08949805	1,31039340	0,04487327	0,03906445	1,16150873	0,16143217	0,87843047	0,05904669	2,29421405	5,45281043	0,46522483	0,14599592	49
0,09527556	0,08340340	2,77454269	0,04541303	0,07695986	0,17607354	0,18291560	0,59553161	0,04275539	0,09038643	0,01436050	1,32248375	0,03217870	0,02631903	0,13929834	0,16280873	0,63386609	0,15466836	2,80159808	1,87157129	0,45699491	0,08305725	50
0,03622803	0,02554616	1,44018086	0,05472144	0,05877107	0,05155478	0,16034486	0,54686248	0,02902118	0,40535280	0,00786820	2,00868313	0,04081519	0,02508989	0,54534836	0,13852805	0,51485925	0,09534162	5,64608168	7,89670064	0,22025551	0,07101413	51
-99	-99	-99	-99	-99	-99	-99	-99	-99	-99	-99	-99	-99	-99	-99	-99	-99	-99	-99	-99	-99	-99	52
-99	-99	-99	-99	-99	-99	-99	-99	-99	-99	-99	-99	-99	-99	-99	-99	-99	-99	-99	-99	-99	-99	53
-99	-99	-99	-99	-99	-99	-99	-99	-99	-99	-99	-99	-99	-99	-99	-99	-99	-99	-99	-99	-99	-99	54
-99	-99	-99	-99	-99	-99	-99	-99	-99	-99	-99	-99	-99	-99	-99	-99	-99	-99	-99	-99	-99	-99	55
-99	-99	-99	-99	-99	-99	-99	-99	-99	-99	-99	-99	-99	-99	-99	-99	-99	-99	-99	-99	-99	-99	56
0,03327886	0,06487246	2,13428080	0,13973352	0,04057542	0,08354805	0,06981835	0,87402715	0,06702067	0,13367022	0,02197109	0,56438439	0,03416706	0,02629168	0,07025526	0,09697455	0,23370330	0,02735124	6,57846049	2,58963721	0,31682322	0,06952858	57
0,05643563	0,14536481	3,81517703	0,15101356	0,18501960	0,12147657	0,38255789	1,61860393	0,10165478	0,33512007	0,02458207	1,00468971	0,11047180	0,05222014	0,15461476	0,10465767	0,56321201	0,08536272	7,50446903	6,64260902	0,74007839	0,16709610	58
-99	-99	-99	-99	-99	-99	-99	-99	-99	-99	-99	-99	-99	-99	-99	-99	-99	-99	-99	-99	-99	-99	59
-99	-99	-99	-99	-99	-99	-99	-99	-99	-99	-99	-99	-99	-99	-99	-99	-99	-99	-99	-99	-99	-99	60
0,04521672	0,01770131	4,00000000	0,04623086	0,03412564	0,02378177	0,36780169	0,53403270	0,06280399	0,36627522	0,01929004	1,05848439	0,01876255	0,03033233	0,29606790	0,08699040	0,25121596	0,08573333	6,11774511	2,59367911	0,38608757	0,06606363	61
-99	-99	-99	-99	-99	-99	-99	-99	-99	-99	-99	-99	-99	-99	-99	-99	-99	-99	-99	-99	-99	-99	62
-99	-99	-99	-99	-99	-99	-99	-99	-99	-99	-99	-99	-99	-99	-99	-99	-99	-99	-99	-99	-99	-99	63
-99	-99	-99	-99	-99	-99	-99	-99	-99	-99	-99	-99	-99	-99	-99	-99	-99	-99	-99	-99	-99	-99	64
0,05943679	0,09096781	4,33941330	0,06700906	0,10878111	0,05290324	0,16951678	1,84868574	0,04660085	0,58216500	0,02719528	0,69039791	0,06379115	0,05039764	0,65044546	0,25658345	0,21131982	0,15915452	1,76234943	6,00639769	0,41250971	0,07191816	65
-99	-99	-99	-99	-99	-99	-99	-99	-99	-99	-99	-99	-99	-99	-99	-99	-99	-99	-99	-99	-99	-99	66
-99	-99	-99	-99	-99	-99	-99	-99	-99	-99	-99	-99	-99	-99	-99	-99	-99	-99	-99	-99	-99	-99	67
-99	-99	-99	-99	-99	-99	-99	-99	-99	-99	-99	-99	-99	-99	-99	-99	-99	-99	-99	-99	-99	-99	68
-99	-99	-99	-99	-99	-99	-99	-99	-99	-99	-99	-99	-99	-99	-99	-99	-99	-99	-99	-99	-99	-99	69
-99	-99	-99	-99	-99	-99	-99	-99	-99	-99	-99	-99	-99	-99	-99	-99	-99	-99	-99	-99	-99	-99	70

-99	-99	-99	-99	-99	-99	-99	-99	-99	-99	-99	-99	-99	-99	-99	-99	-99	-99	-99	-99	-99	-99	71
-99	-99	-99	-99	-99	-99	-99	-99	-99	-99	-99	-99	-99	-99	-99	-99	-99	-99	-99	-99	-99	-99	72
-99	-99	-99	-99	-99	-99	-99	-99	-99	-99	-99	-99	-99	-99	-99	-99	-99	-99	-99	-99	-99	-99	73
-99	-99	-99	-99	-99	-99	-99	-99	-99	-99	-99	-99	-99	-99	-99	-99	-99	-99	-99	-99	-99	-99	74
0,09021430	0,01605871	3,07908138	0,09033945	0,06330661	0,12003275	0,13401812	1,87449243	0,02189128	0,35123293	0,02440804	1,90990384	0,07272020	0,03553784	0,15036076	0,27903152	0,51494848	0,06942023	5,07532427	5,63142487	0,51210090	0,09198229	75
-99	-99	-99	-99	-99	-99	-99	-99	-99	-99	-99	-99	-99	-99	-99	-99	-99	-99	-99	-99	-99	-99	76
-99	-99	-99	-99	-99	-99	-99	-99	-99	-99	-99	-99	-99	-99	-99	-99	-99	-99	-99	-99	-99	-99	77
-99	-99	-99	-99	-99	-99	-99	-99	-99	-99	-99	-99	-99	-99	-99	-99	-99	-99	-99	-99	-99	-99	78
-99	-99	-99	-99	-99	-99	-99	-99	-99	-99	-99	-99	-99	-99	-99	-99	-99	-99	-99	-99	-99	-99	79
-99	-99	-99	-99	-99	-99	-99	-99	-99	-99	-99	-99	-99	-99	-99	-99	-99	-99	-99	-99	-99	-99	80
-99	-99	-99	-99	-99	-99	-99	-99	-99	-99	-99	-99	-99	-99	-99	-99	-99	-99	-99	-99	-99	-99	81
-99	-99	-99	-99	-99	-99	-99	-99	-99	-99	-99	-99	-99	-99	-99	-99	-99	-99	-99	-99	-99	-99	82
-99	-99	-99	-99	-99	-99	-99	-99	-99	-99	-99	-99	-99	-99	-99	-99	-99	-99	-99	-99	-99	-99	83
0,04042103	0,17328876	2,02125312	0,09114138	0,09627133	0,01941412	0,00003612	0,96409686	0,11778682	0,41243824	0,00003612	2,72121321	0,04901102	0,01664520	0,81521359	0,12536878	0,54876105	0,12545571	6,05551642	9,41036055	0,69507955	0,18534049	84
-99	-99	-99	-99	-99	-99	-99	-99	-99	-99	-99	-99	-99	-99	-99	-99	-99	-99	-99	-99	-99	-99	85
-99	-99	-99	-99	-99	-99	-99	-99	-99	-99	-99	-99	-99	-99	-99	-99	-99	-99	-99	-99	-99	-99	86
0,07414535	0,14503771	2,92918639	0,19485867	0,06463682	0,15298902	0,45549275	1,12622884	0,13281592	0,35985809	0,00855213	0,65861168	0,09027686	0,05541806	0,25962478	0,16556849	0,46506362	0,20986013	7,12801316	8,69689249	0,77190756	0,20257106	87
0,05847748	0,06226326	2,41818564	0,06471415	0,04004386	0,10242395	0,50028600	0,64497941	0,13936353	0,03883026	0,02690140	1,83938694	0,06242315	0,02945640	0,15018629	0,15859564	0,49073804	0,00973452	3,25086805	3,11649630	0,62175797	0,07597797	88
0,07578205	0,14269434	2,77213977	0,15485609	0,12413656	0,08420210	0,00005949	1,81001293	0,15821957	0,14249666	0,02078952	2,02932509	0,05767141	0,02754625	0,15528604	0,23020642	0,57434918	0,11614508	5,44525647	5,36285037	0,31468929	0,11990801	89
-99	-99	-99	-99	-99	-99	-99	-99	-99	-99	-99	-99	-99	-99	-99	-99	-99	-99	-99	-99	-99	-99	90
-99	-99	-99	-99	-99	-99	-99	-99	-99	-99	-99	-99	-99	-99	-99	-99	-99	-99	-99	-99	-99	-99	91
-99	-99	-99	-99	-99	-99	-99	-99	-99	-99	-99	-99	-99	-99	-99	-99	-99	-99	-99	-99	-99	-99	92
-99	-99	-99	-99	-99	-99	-99	-99	-99	-99	-99	-99	-99	-99	-99	-99	-99	-99	-99	-99	-99	-99	93
-99	-99	-99	-99	-99	-99	-99	-99	-99	-99	-99	-99	-99	-99	-99	-99	-99	-99	-99	-99	-99	-99	94
-99	-99	-99	-99	-99	-99	-99	-99	-99	-99	-99	-99	-99	-99	-99	-99	-99	-99	-99	-99	-99	-99	95
-99	-99	-99	-99	-99	-99	-99	-99	-99	-99	-99	-99	-99	-99	-99	-99	-99	-99	-99	-99	-99	-99	96
-99	-99	-99	-99	-99	-99	-99	-99	-99	-99	-99	-99	-99	-99	-99	-99	-99	-99	-99	-99	-99	-99	97
-99	-99	-99	-99	-99	-99	-99	-99	-99	-99	-99	-99	-99	-99	-99	-99	-99	-99	-99	-99	-99	-99	98
0,07566396	0,07103875	3,71085081	0,08565908	0,07143377	0,11024232	0,19981683	1,60630962	0,04536583	0,83436468	0,00060062	1,61860393	0,01725012	0,02784863	0,52658926	0,25309484	0,25099839	0,14821363	2,94190365	2,50489477	0,71784785	0,11342024	99
0,11715578	0,10938600	2,61443645	0,01709241	0,09065309	0,10355722	0,16272412	1,85767741	0,06365863	0,32771191	0,01826847	0,80748088	0,06026650	0,02061017	0,15394640	0,18256726	0,52069123	0,03595908	6,47778237	8,14265908	0,25165166	0,11658871	100
-99	-99	-99	-99	-99	-99	-99	-99	-99	-99	-99	-99	-99	-99	-99	-99	-99	-99	-99	-99	-99	-99	101
-99	-99	-99	-99	-99	-99	-99	-99	-99	-99	-99	-99	-99	-99	-99	-99	-99	-99	-99	-99	-99	-99	102
0,05204849	0,16863782	3,57514065	0,06792093	0,04316483	0,13048899	0,14043749	1,97520172	0,08449443	0,00003048	0,00721392	0,77808518	0,05811281	0,03783853	0,71747477	0,25261286	0,60290391	0,16042824	8,28786211	6,47105079	0,40360054	0,04869779	103
0,06828677	0,21415837	3,01414746	0,00433050	0,08120712	0,11790935	0,19981683	2,69400028	0,05168000	0,00001001	0,02974415	0,84425458	0,04995421	0,02509859	0,15038682	0,44066371	0,39168071	0,11271489	11,38253508	8,23489355	0,79926732	0,33932744	104
-99	-99	-99	-99	-99	-99	-99	-99	-99	-99	-99	-99	-99	-99	-99	-99	-99	-99	-99	-99	-99	-99	105
0,06620115	0,07753546	5,64901762	0,19888412	0,02568376	0,04863032	0,28697560	1,40541870	0,06202527	0,00945308	0,01622093	0,69544099	0,05048505	0,06129869	0,86753869	0,27224925	0,60836118	0,17146666	0,12200397	2,48629934	0,36831192	0,13157889	106
0,08884904	0,11490393	3,81980767	0,00858777	0,09865254	0,04949752	0,12170835	1,79688739	0,02980091	0,60646656	0,02051041	0,82788369	0,04918970	0,03180726	0,19084853	0,13980618	0,59398566	0,11199452	1,70231734	3,14377943	0,25165166	0,11014684	107
-99	-99	-99	-99	-99	-99	-99	-99	-99	-99	-99	-99	-99	-99	-99	-99	-99	-99	-99	-99	-99	-99	108
-99	-99	-99	-99	-99	-99	-99	-99	-99	-99	-99	-99	-99	-99	-99	-99	-99	-99	-99	-99	-99	-99	109
0,07469989	0,13520777	5,39360549	0,07215534	0,04432456	0,13427382	0,59842817	1,74140286	0,12694276	0,22833913	0,03394281	1,66468591	0,03572926	0,03901709	0,80958249	0,28405654	0,37689903	0,10549525	2,85254570	2,86840751	0,69027828	0,06017258	110
-99	-99	-99	-99	-99	-99	-99	-99	-99	-99	-99	-99	-99	-99	-99	-99	-99	-99	-99	-99	-99	-99	111
-99	-99	-99	-99	-99	-99	-99	-99	-99	-99	-99	-99	-99	-99	-99	-99	-99	-99	-99	-99	-99	-99	112
-99	-99	-99	-99	-99	-99	-99	-99	-99	-99	-99	-99	-99	-99	-99	-99	-99	-99	-99	-99	-99	-99	113
0,13860008	0,03768149	2,01670491	0,17075503	0,03584709	0,01146993	0,04502905	0,26535580	0,09099935	0,07335301	0,01546339	1,37936092	0,10540389	0,06474892	0,84206295	0,36831192	0,57196549	0,05823378	3,96961750	6,06286627	0,32782550	0,23931786	114
-99	-99	-99	-99	-99	-99	-99	-99	-99	-99	-99	-99	-99	-99	-99	-99	-99	-99	-99	-99	-99	-99	115
-99	-99	-99	-99	-99	-99	-99	-99	-99	-99	-99	-99	-99	-99	-99	-99	-99	-99	-99	-99	-99	-99	116
-99	-99	-99	-99	-99	-99	-99	-99	-99	-99	-99	-99	-99	-99	-99	-99	-99	-99	-99	-99	-99	-99	117
-99	-99	-99	-99	-99	-99	-99	-99	-99	-99	-99	-99	-99	-99	-99	-99	-99	-99	-99	-99	-99	-99	118
-99	-99	-99	-99	-99	-99	-99	-99	-99	-99	-99	-99	-99	-99	-99	-99	-99	-99	-99	-99	-99	-99	119
0,18190410	0,03942488	1,69613382	0,05728297	0,08670446	0,10063820	0,19645217	1,27920723	0,16023375	0,34705832	0,00569634	2,21798560	0,04691685	0,05610410	0,15328092	0,10303811	0,35312479	0,21467856	1,81283799	3,60812670	0,64673653	0,03439279	120
0,08774739	0,03229602	1,95816183	0,00535276	0,06196082	0,07282108	0,17140725	1,04282711	0,03409018	0,22633004	0,00595163	1,51204387	0,04196993	0,02900610	0,66734283	0,46249190	0,57375233	0,09865254	4,33340176	2,73871663	0,30051314	0,11846231	121
0,14680775	0,00476272	3,27614679	0,10380875	0,06754534	0,05843595	0,44596426	2,15546629	0,15072598	0,63595650	0,01123388	0,83973149	0,07325139	0,03088393	0,34317111	0,19833346	0,59708159	0,16852097	1,38991822	1,85060886	0,33821206	0,07345477	122
-99	-99	-99	-99	-99	-99	-99	-99	-99	-99	-99	-99	-99	-99	-99	-99	-99	-99	-99	-99	-99	-99	123
-99	-99	-99	-99	-99	-99	-99	-99	-99	-99	-99	-99	-99	-99	-99	-99	-99	-99	-99	-99	-99	-99	124
-99	-99	-99	-99	-99	-99	-99	-99	-99	-99	-99	-99	-99	-99	-99	-99	-99	-99	-99	-99	-99	-99	125
-99	-99	-99	-99	-99	-99	-99	-99	-99	-99	-99	-99	-99	-99	-99	-99	-99	-99	-99	-99	-99	-99	126
0,08174362	0,13224178	3,19817748	0,11584358	0,05548532	0,12126625	0,35706286	1,49096873	0,05080978	0,40647823	0,05760149	1,09296712	0,08011688	0,04061763	0,55874038	0,27648057	0,68456052	0,06307666	2,20878041	3,43247687	0,61567889	0,11959674	127
-99	-99	-99	-99	-99	-99	-99	-99	-99	-99	-99	-99	-99	-99	-99	-99	-99	-99	-99	-99	-99	-99	128
-99	-99	-99	-99	-99	-99	-99	-99	-99	-99	-99	-99	-99	-99	-99	-99	-99	-99	-99	-99	-99	-99	129
-99	-99	-99	-99	-99	-99	-99	-99	-99	-99	-99	-99	-99	-99	-99	-99	-99	-99	-99	-99	-99	-99	130
-99	-99	-99	-99	-99	-99	-99	-99	-99	-99	-99	-99	-99	-99	-99	-99	-99	-99	-99	-99	-99	-99	131
-99	-99	-99	-99	-99	-99	-99	-99	-99	-99	-99	-99	-99	-99	-99	-99	-99	-99	-99	-99	-99	-99	132
-99	-99	-99	-99	-99	-99	-99	-99	-99	-99	-99	-99	-99	-99	-99	-99	-99	-99	-99	-99	-99	-99	133
-99	-99	-99	-99	-99	-99	-99	-99	-99	-99	-99	-99	-99	-99	-99	-99	-99	-99	-99	-99	-99	-99	134
-99	-99	-99	-99	-99	-99	-99	-99	-99	-99	-99	-99	-99	-99	-99	-99	-99	-99	-99	-99	-99	-99	135
-99	-99	-99	-99	-99	-99	-99	-99	-99	-99	-99	-99	-99	-99	-99	-99	-99	-99	-99	-99	-99	-99	136
-99	-99	-99	-99	-99	-99	-99	-99	-99	-99	-99	-99	-99	-99	-99	-99	-99	-99	-99	-99	-99	-99	137
0,13593605	0,08525924	2,01391110	0,17087343	0,10518494	0,08632965	0,41552293	1,18345102	0,12753808	0,05233791	0,02060303	0,90563398	0,05855759	0,09848174	0,26152123	0,13688156	0,76897042	0,08496427	3,26028933	4,32290161	0,47106564	0,07199298	138
-99	-99	-99	-99	-99	-99	-99	-99	-99	-99	-99	-99	-99	-99	-99	-99	-99	-99	-99	-99	-99	-99	139
-99	-99	-99	-99	-99	-99	-99	-99	-99	-99	-99	-99	-99	-99	-99	-99	-99	-99	-99	-99	-99	-99	140
0,14508799	0,12336457	3,74249399	0,08150317	0,05896489	0,05941619	0,32896363	1,65061082	0,02962584	0,54714685	0,01664232	0,89378516	0,02465459	0,03698284	0,74329149	0,19025417	0,37061675	0,05295828	2,69633546	1,91719888	0,65383567	0,15051717	141
0,09367124	0,19394910	3,20372430	0,29745637	0,07035272	0,21164965	0,07529772	1,75047930	0,05012764	1,63099233	0,13983040	1,69672176	0,38282316	0,23144640	0,31441675	0,22527358	0,65826938	0,18721267	3,15086949	5,78272702	0,44496075	0,40437060	142
0,13857606	0,18071022	3,07321777	0,07645475	0,06057012	0,08314366	0,26549379	1,23263871	0,02931433	0,67303340	0,01970561	0,88132742	0,04838655	0,03965784	0,76352643	0,23032613	0,41430065	0,08751960	4,97863414	4,38856720	0,49783860	0,07822372	143
-99	-99	-99	-99	-99	-99	-99	-99	-99	-99	-99	-99	-99	-99	-99	-99	-99	-99	-99	-99	-99	-99	144
0,07174390	0,09759831	2,40327210	0,06867839	0,06848824	0,14875396	0,17099191	1,81252388	0,07355667	0,14650279	0,02300354	1,48143980	0,12293776	0,04876534	0,15898913	0,31057207	0,38689125	0,15658305	2,38336515	4,39846460	0,49448546	0,08105247	145
-99	-99	-99	-99	-99	-99	-99	-99	-99	-99	-99	-99	-99	-99	-99	-99	-99	-99	-99	-99	-99	-99	146
0,06934809	0,02707303	4,35296977	0,25756349	0,13471664	0,12090954	0,09252583	1,30314715	0,08796052	0,26646168	0,04143518	1,75807912	0,15669163	0,09375244	0,16597064	0,34484023	0,74638919	0,09944206	6,75520710	12,19315864	0,42868802	0,30460257	147
-99	-99	-99	-99	-99	-99	-99	-99	-99	-99	-99	-99	-99	-99	-99	-99	-99	-99	-99	-99	-99	-99	148
-99	-99	-99	-99	-99	-99	-99	-99	-99	-99	-99	-99	-99	-99	-99	-99	-99	-99	-99	-99	-99	-99	149
-99	-99	-99	-99	-99	-99	-99	-99	-99	-99	-99	-99	-99	-99	-99	-99	-99	-99	-99	-99	-99	-99	150
-99	-99	-99	-99	-99	-99	-99	-99	-99	-99	-99	-99	-99	-99	-99	-99	-99	-99	-99	-99	-99	-99	151
0,06629298	0,00949247	1,98894034	0,01783679	0,04903651	0,13378609	0,20124153	0,90125046	0,05570691	0,35404386	0,01525050	0,97198099	0,03609643	0,02577292	0,16539643	0,29709578	0,52960917	0,09658882	1,99169951	3,25577274	0,55133458	0,07515433	152
-99	-99	-99	-99	-99	-99	-99	-99	-99	-99	-99	-99	-99	-99	-99	-99	-99	-99	-99	-99	-99	-99	153

-99	-99	-99	-99	-99	-99	-99	-99	-99	-99	-99	-99	-99	-99	-99	-99	-99	-99	-99	-99	-99	-99	154
-99	-99	-99	-99	-99	-99	-99	-99	-99	-99	-99	-99	-99	-99	-99	-99	-99	-99	-99	-99	-99	-99	155
-99	-99	-99	-99	-99	-99	-99	-99	-99	-99	-99	-99	-99	-99	-99	-99	-99	-99	-99	-99	-99	-99	156
-99	-99	-99	-99	-99	-99	-99	-99	-99	-99	-99	-99	-99	-99	-99	-99	-99	-99	-99	-99	-99	-99	157
-99	-99	-99	-99	-99	-99	-99	-99	-99	-99	-99	-99	-99	-99	-99	-99	-99	-99	-99	-99	-99	-99	158
0,06395717	0,07250629	5,53755837	0,08908029	0,06878558	0,09455188	0,00006207	1,75108607	0,09177532	0,90140665	0,03587817	2,02686500	0,08311485	0,03989219	0,20016339	0,25056382	0,40760679	0,40339078	6,58986998	7,59078979	0,51987980	0,08106652	159
0,07082160	0,34206285	2,89253283	0,38832415	0,05319127	0,08067879	0,15968861	1,80291744	0,05638024	0,04429640	0,00848619	0,73577335	0,05537326	0,06476388	0,23656906	0,55069802	0,68460006	0,22787797	1,92964216	6,17454716	0,45544013	0,15307741	160
0,08333117	0,07494624	5,05951817	0,04716956	0,06224061	0,06232695	0,00001677	1,95612695	0,06796802	0,35576587	0,00691526	2,14057740	0,03340019	0,02041821	0,20292240	0,18174656	0,56370021	0,29017598	6,74117464	7,50576957	0,65519670	0,10302026	161
-99	-99	-99	-99	-99	-99	-99	-99	-99	-99	-99	-99	-99	-99	-99	-99	-99	-99	-99	-99	-99	-99	162
-99	-99	-99	-99	-99	-99	-99	-99	-99	-99	-99	-99	-99	-99	-99	-99	-99	-99	-99	-99	-99	-99	163
0,07915092	0,03775339	4,15183756	0,03362668	0,08536272	0,01510060	0,41458792	1,40614951	0,06781508	0,34501955	0,06031874	0,95511741	0,01827164	0,01709538	0,13886453	0,09563947	0,52695439	0,09887503	2,90140160	1,35542943	0,40409040	0,03783198	164
-99	-99	-99	-99	-99	-99	-99	-99	-99	-99	-99	-99	-99	-99	-99	-99	-99	-99	-99	-99	-99	-99	165
-99	-99	-99	-99	-99	-99	-99	-99	-99	-99	-99	-99	-99	-99	-99	-99	-99	-99	-99	-99	-99	-99	166
0,04139212	0,01656750	5,03677400	0,00819239	0,03258835	0,07559843	0,19675879	0,59936220	0,10522140	0,55536186	0,01063158	0,94572982	0,04205730	0,02663561	0,94572982	0,20568379	0,36261236	0,03490312	4,55515454	4,01806249	0,68042120	0,13980618	167
-99	-99	-99	-99	-99	-99	-99	-99	-99	-99	-99	-99	-99	-99	-99	-99	-99	-99	-99	-99	-99	-99	168
-99	-99	-99	-99	-99	-99	-99	-99	-99	-99	-99	-99	-99	-99	-99	-99	-99	-99	-99	-99	-99	-99	169
-99	-99	-99	-99	-99	-99	-99	-99	-99	-99	-99	-99	-99	-99	-99	-99	-99	-99	-99	-99	-99	-99	170
0,11518302	0,04753059	3,84237946	0,07229301	0,10097010	0,08116491	0,16840420	0,56019461	0,08292782	0,40416043	0,01674646	1,00069339	0,08789957	0,00955188	0,57434918	0,13067001	0,77700727	0,07489431	2,43175857	7,66348175	0,49004971	0,06863080	171
0,09837940	0,09384996	2,69727010	0,10086517	0,06495120	0,10128553	0,41250971	1,52573034	0,09021430	0,13836011	0,01675227	1,05738444	0,03274685	0,05210263	0,46121137	0,12740555	0,45203300	0,07302327	5,25066444	4,84837067	0,41913880	0,14333869	172
-99	-99	-99	-99	-99	-99	-99	-99	-99	-99	-99	-99	-99	-99	-99	-99	-99	-99	-99	-99	-99	-99	173
0,12864792	0,09033945	1,52467315	0,09865254	0,03785165	0,14224995	0,15437382	1,75017599	0,08108057	0,28747333	0,02468024	0,88976731	0,06617821	0,04139212	0,90469287	0,22075224	0,40598547	0,06832228	2,67863870	3,95999882	0,32793914	0,14849641	174
0,09007372	0,02203972	4,37186848	0,11664934	0,05804236	0,21843107	0,00000990	1,36722435	0,12844744	0,14713884	0,03660666	1,63155769	0,10883768	0,10944288	0,69604380	0,29062888	0,53821332	0,05126969	2,19048449	4,26708249	0,26448357	0,12925124	175
0,06997581	0,13850404	1,38510947	0,15030866	0,09923549	0,05791175	0,36349313	2,24077843	0,01739721	0,02849791	0,00520459	1,03598276	0,04707158	0,04903651	0,11558291	0,20819394	0,52522227	0,04364620	5,90935225	2,29739671	0,31035687	0,13860008	176
0,05578419	0,07124833	2,01810327	0,05640630	0,05474989	0,08408545	0,18972741	1,02740144	0,05835500	0,20589775	0,00202200	0,67548704	0,03133676	0,02501609	0,73764573	0,12967749	0,47467106	0,08894147	3,02513599	3,75809100	0,41841312	0,10694938	177
-99	-99	-99	-99	-99	-99	-99	-99	-99	-99	-99	-99	-99	-99	-99	-99	-99	-99	-99	-99	-99	-99	178
-99	-99	-99	-99	-99	-99	-99	-99	-99	-99	-99	-99	-99	-99	-99	-99	-99	-99	-99	-99	-99	-99	179
-99	-99	-99	-99	-99	-99	-99	-99	-99	-99	-99	-99	-99	-99	-99	-99	-99	-99	-99	-99	-99	-99	180
-99	-99	-99	-99	-99	-99	-99	-99	-99	-99	-99	-99	-99	-99	-99	-99	-99	-99	-99	-99	-99	-99	181
0,05069545	0,16875475	4,75682846	0,14053487	0,08467031	0,12183495	0,00008269	1,36793530	0,05392133	0,00594957	0,00463248	2,06622878	0,08304286	0,05880163	0,22829956	0,29895495	0,45344516	0,18505166	7,35130307	8,08921665	0,42395998	0,19957460	182
-99	-99	-99	-99	-99	-99	-99	-99	-99	-99	-99	-99	-99	-99	-99	-99	-99	-99	-99	-99	-99	-99	183
-99	-99	-99	-99	-99	-99	-99	-99	-99	-99	-99	-99	-99	-99	-99	-99	-99	-99	-99	-99	-99	-99	184
-99	-99	-99	-99	-99	-99	-99	-99	-99	-99	-99	-99	-99	-99	-99	-99	-99	-99	-99	-99	-99	-99	185
-99	-99	-99	-99	-99	-99	-99	-99	-99	-99	-99	-99	-99	-99	-99	-99	-99	-99	-99	-99	-99	-99	186
-99	-99	-99	-99	-99	-99	-99	-99	-99	-99	-99	-99	-99	-99	-99	-99	-99	-99	-99	-99	-99	-99	187
-99	-99	-99	-99	-99	-99	-99	-99	-99	-99	-99	-99	-99	-99	-99	-99	-99	-99	-99	-99	-99	-99	188
0,06742839	0,09255790	4,46141081	0,02840916	0,03973351	0,10044656	0,12539050	2,07412091	0,04182473	0,59646112	0,00004535	0,83827761	0,04320973	0,02658028	0,71029981	0,14068106	0,38302222	0,20768947	10,37831266	5,71795791	0,38729372	0,08108057	189
-99	-99	-99	-99	-99	-99	-99	-99	-99	-99	-99	-99	-99	-99	-99	-99	-99	-99	-99	-99	-99	-99	190
-99	-99	-99	-99	-99	-99	-99	-99	-99	-99	-99	-99	-99	-99	-99	-99	-99	-99	-99	-99	-99	-99	191
-99	-99	-99	-99	-99	-99	-99	-99	-99	-99	-99	-99	-99	-99	-99	-99	-99	-99	-99	-99	-99	-99	192
-99	-99	-99	-99	-99	-99	-99	-99	-99	-99	-99	-99	-99	-99	-99	-99	-99	-99	-99	-99	-99	-99	193
-99	-99	-99	-99	-99	-99	-99	-99	-99	-99	-99	-99	-99	-99	-99	-99	-99	-99	-99	-99	-99	-99	194
-99	-99	-99	-99	-99	-99	-99	-99	-99	-99	-99	-99	-99	-99	-99	-99	-99	-99	-99	-99	-99	-99	195
-99	-99	-99	-99	-99	-99	-99	-99	-99	-99	-99	-99	-99	-99	-99	-99	-99	-99	-99	-99	-99	-99	196
-99	-99	-99	-99	-99	-99	-99	-99	-99	-99	-99	-99	-99	-99	-99	-99	-99	-99	-99	-99	-99	-99	197
0,07041370	0,01451814	1,69231719	0,07340387	0,05362316	0,13735678	0,00008590	2,08204320	0,10467581	0,84615860	0,01107154	2,25167717	0,05609438	0,04233516	0,68968046	0,15810994	0,51121427	0,03802257	1,24573742	2,48113460	0,39229205	0,08241210	198
0,09592163	0,06721840	3,01258093	0,04334472	0,09258999	0,02664484	0,11446671	0,38904259	0,04052624	0,22782532	0,07716016	0,81281560	0,05126081	0,04852930	0,63068870	0,15865886	0,47139227	0,09401273	4,46605185	7,32586949	0,21139307	0,18556545	199
-99	-99	-99	-99	-99	-99	-99	-99	-99	-99	-99	-99	-99	-99	-99	-99	-99	-99	-99	-99	-99	-99	200
-99	-99	-99	-99	-99	-99	-99	-99	-99	-99	-99	-99	-99	-99	-99	-99	-99	-99	-99	-99	-99	-99	201
0,08626983	0,13039857	2,13761198	0,12166617	0,07031616	0,13879235	0,18212489	2,36198532	0,11391266	0,64752150	0,01420950	1,10956947	0,04493552	0,03887536	0,32623874	0,24672914	0,50522572	0,18492344	4,59479342	4,54096841	0,55671081	0,09678988	202
0,09944206	0,00846074	2,36198532	0,03441067	0,10851753	0,17592105	0,23981603	1,04971668	0,13276990	0,48733982	0,06095972	1,18755967	0,02234352	0,03572307	0,19398271	0,17028225	0,42278614	0,15251256	3,01049349	3,69609029	0,47237352	0,07124833	203
-99	-99	-99	-99	-99	-99	-99	-99	-99	-99	-99	-99	-99	-99	-99	-99	-99	-99	-99	-99	-99	-99	204
-99	-99	-99	-99	-99	-99	-99	-99	-99	-99	-99	-99	-99	-99	-99	-99	-99	-99	-99	-99	-99	-99	205
-99	-99	-99	-99	-99	-99	-99	-99	-99	-99	-99	-99	-99	-99	-99	-99	-99	-99	-99	-99	-99	-99	206
-99	-99	-99	-99	-99	-99	-99	-99	-99	-99	-99	-99	-99	-99	-99	-99	-99	-99	-99	-99	-99	-99	207
-99	-99	-99	-99	-99	-99	-99	-99	-99	-99	-99	-99	-99	-99	-99	-99	-99	-99	-99	-99	-99	-99	208
0,14984055	0,10802971	1,89934239	0,07231807	0,07455764	0,11122091	0,33459783	1,96632255	0,08588202	0,02860676	0,01208616	1,06547741	0,02747951	0,03814136	1,18304094	0,08170114	0,53607249	0,12487010	3,41882363	3,48099562	0,45834314	0,10984187	209
-99	-99	-99	-99	-99	-99	-99	-99	-99	-99	-99	-99	-99	-99	-99	-99	-99	-99	-99	-99	-99	-99	210
-99	-99	-99	-99	-99	-99	-99	-99	-99	-99	-99	-99	-99	-99	-99	-99	-99	-99	-99	-99	-99	-99	211
0,05488859	0,05704918	2,63508591	0,09165453	0,01878012	0,04453397	0,27625070	0,55357473	0,06283665	0,03859479	0,01300987	0,86967604	0,03513250	0,03494307	0,16220048	0,13997586	0,40319510	0,12216052	4,65748138	7,48006082	0,32863317	0,07409140	212
-99	-99	-99	-99	-99	-99	-99	-99	-99	-99	-99	-99	-99	-99	-99	-99	-99	-99	-99	-99	-99	-99	213
0,09380119	0,08331673	3,78619837	0,02966180	0,07741463	0,09308871	0,00002282	1,36202199	0,11139451	0,29973303	0,02446308	0,66422780	0,05086263	0,03579123	0,62709243	0,16640261	0,50865170	0,05798204	6,15069740	4,66135697	0,48254989	0,08530357	214
-99	-99	-99	-99	-99	-99	-99	-99	-99	-99	-99	-99	-99	-99	-99	-99	-99	-99	-99	-99	-99	-99	215
0,05656290	0,03407837	3,27841843	0,04221794	0,08937406	0,02531261	0,00002375	1,37458870	0,07960484	0,77110541	0,02450126	1,12272142	0,05628912	0,01582117	0,16643145	0,14803395	0,61599904	0,13621901	3,08014870	6,93069485	0,30291817	0,07453180	216
-99	-99	-99	-99	-99	-99	-99	-99	-99	-99	-99	-99	-99	-99	-99	-99	-99	-99	-99	-99	-99	-99	217
-99	-99	-99	-99	-99	-99	-99	-99	-99	-99	-99	-99	-99	-99	-99	-99	-99	-99	-99	-99	-99	-99	218
-99	-99	-99	-99	-99	-99	-99	-99	-99	-99	-99	-99	-99	-99	-99	-99	-99	-99	-99	-99	-99	-99	219
0,01992537	0,06568692	3,95245764	0,02591175	0,09328248	0,17760573	0,18310588	1,53660888	0,06210056	0,18943174	0,01387614	1,19893214	0,05718379	0,03126625	0,63014249	0,02972354	0,40577447	0,06541431	3,20372430	6,33678031	0,07493326	0,08036716	220
0,05958116	0,06319701	2,91197910	0,06045477	0,04839494	0,10799227	0,37683373	2,10526231	0,08321573	0,15304205	0,00001269	1,31312113	0,03778611	0,03637270	1,10956947	0,18036609	0,59666787	0,15712667	5,50980019	8,84584523	0,21763764	0,03994061	221
0,06660388	0,02835506	4,05232533	0,11988724	0,07865869	0,19008940	0,13137385	1,47605860	0,09263813	2,20190160	0,09928709	1,89441184	0,05065154	0,05376272	0,10004701	0,19926359	0,36041976	0,27927339	2,02335575	2,45504590	0,39604877	0,18083552	222
0,05205751	0,03597778	1,54998043	0,13418079	0,02696534	0,06281487	0,41473163	1,28275887	0,03789759	0,08225516	0,03660666	2,79190529	0,04417120	0,05393068	0,58774000	0,24625928	0,57604363	0,16936997	2,25832011	6,38306792	0,39618605	0,24371214	223
-99	-99	-99	-99	-99	-99	-99	-99	-99	-99	-99	-99	-99	-99	-99	-99	-99	-99	-99	-99	-99	-99	224
0,09741245	0,05975694	1,26861121	0,15381307	0,08574819	0,04129898	0,22379505	1,19851669	0,08951356	0,14421069	0,03518855	1,05354359	0,12364279	0,07362043	0,11401140	0,50671625	0,70088517	0,06503003	3,02356375	8,65929757	0,16984021	0,25283183	225
0,14266961	0,04004456	1,97759911	0,09041776	0,04967797	0,09812401	0,23977448	1,45775675	0,10172527	0,00932778	0,01295587	1,88002259	0,07698654	0,05612355	1,00329788	0,42653913	0,96443105	0,24857446	0,10336001	1,08956329	0,37546491	0,04664124	226
-99	-99	-99	-99	-99	-99	-99	-99	-99	-99	-99	-99	-99	-99	-99	-99	-99	-99	-99	-99	-99	-99	227
-99	-99	-99	-99	-99	-99	-99	-99	-99	-99	-99	-99	-99	-99	-99	-99	-99	-99	-99	-99	-99	-99	228
-99	-99	-99	-99	-99	-99	-99	-99	-99	-99	-99	-99	-99	-99	-99	-99	-99	-99	-99	-99	-99	-99	229
-99	-99	-99	-99	-99	-99	-99	-99	-99	-99	-99	-99	-99	-99	-99	-99	-99	-99	-99	-99	-99	-99	230
-99	-99	-99	-99	-99	-99	-99	-99	-99	-99	-99	-99	-99	-99	-99	-99	-99	-99	-99	-99	-99	-99	231
0,09052750	0,06946837	2,45291969	0,03461401	0,10002968	0,15729012	0,16963432	0,47450658	0,04188275	0,00193225	0,00002661	1,64775300	0,02819337	0,02457781	0,21456698	0,15309510	0,45171978	0,15320125	4,20906562	4,95711250	0,56781781	0,10938600	232
0,23269305	0,13116912	2,94956050	0,02967722	0,12829172	0,03338861	0,14655357	1,06621619	0,07053583	0,64238050	0,00004595	1,94194185	0,03714984	0,02606938	0,67759729	0,12357853	0,41336840	0,08085608	3,86776462	2,76542267	0,40739495	0,08153142	233
0,05233791	0,04313492	4,41067665	0,10511205	0,06041288	0,13481005	0,17775968	1,48041330	0,16654685	0,64349462	0,01596438	1,68646222	0,05872017	0,07845450	0,67128625	0,17899609	0,34531861	0,08391078	1,39764697	9,65315136	0,38501859	0,10709774	234
0,10469395	0,04880761	2,42376529	0,02751286	0,07464813	0,18936610	0,00004623	1,34280517	0,12084670	0,29797227	0,00004623	0,79292153	0,04129898	0,02531700	0,65304305	0,15531295	0,36254953	0,11885298	2,51446243	4,05654080	0,22881444	0,04907901	235
-99	-99	-99	-99	-99	-99	-99	-99	-99	-99	-99	-99	-99	-99	-99	-99	-99	-99	-99	-99	-99	-99	236

-99	-99	-99	-99	-99	-99	-99	-99	-99	-99	-99	-99	-99	-99	-99	-99	-99	-99	-99	-99	-99	-99	237
-99	-99	-99	-99	-99	-99	-99	-99	-99	-99	-99	-99	-99	-99	-99	-99	-99	-99	-99	-99	-99	-99	238
-99	-99	-99	-99	-99	-99	-99	-99	-99	-99	-99	-99	-99	-99	-99	-99	-99	-99	-99	-99	-99	-99	239
-99	-99	-99	-99	-99	-99	-99	-99	-99	-99	-99	-99	-99	-99	-99	-99	-99	-99	-99	-99	-99	-99	240
-99	-99	-99	-99	-99	-99	-99	-99	-99	-99	-99	-99	-99	-99	-99	-99	-99	-99	-99	-99	-99	-99	241
-99	-99	-99	-99	-99	-99	-99	-99	-99	-99	-99	-99	-99	-99	-99	-99	-99	-99	-99	-99	-99	-99	242
0,04986772	0,02015456	3,11935987	0,09448637	0,07674678	0,13776201	0,75275380	1,73417561	0,04061763	0,03373173	0,00007857	1,33815942	0,07225544	0,02601072	0,76114858	0,04389651	0,50286742	0,07326409	5,46132116	2,50402679	0,33176877	0,08282729	243
0,10936705	0,13690528	3,98685186	0,04469478	0,05852716	0,09007372	0,31041066	0,56536325	0,09613796	0,06355943	0,01543394	0,88899672	0,03363833	0,03191216	0,16056730	0,25565143	0,44051102	0,03645473	3,59315208	4,77748055	0,55296114	0,07744146	244
-99	-99	-99	-99	-99	-99	-99	-99	-99	-99	-99	-99	-99	-99	-99	-99	-99	-99	-99	-99	-99	-99	245
0,09600478	0,15148533	3,08495619	0,17292879	0,10771193	0,28563605	0,00005676	1,00364565	0,07301061	1,18612002	0,02666794	1,54998043	0,07131009	0,09700817	0,58692579	0,14075421	0,54308503	0,04734153	2,42376529	4,14464920	0,21617176	0,12101435	246
-99	-99	-99	-99	-99	-99	-99	-99	-99	-99	-99	-99	-99	-99	-99	-99	-99	-99	-99	-99	-99	-99	247
0,09826013	0,02362978	6,45648967	0,08861839	0,07948079	0,09737870	0,23663740	2,42796900	0,08343231	0,23977448	0,00854177	0,69339532	0,02550193	0,08685484	0,79595016	0,19167711	0,32550464	0,17262939	2,88735801	3,37234001	0,81211166	0,18285221	248
0,06980625	0,07886342	2,70288476	0,13217305	0,08301409	0,12400756	0,29412473	1,01010131	0,09529207	0,04165115	0,00725152	1,08861966	0,03019597	0,02988365	0,71276579	0,21650916	0,52905881	0,08446515	8,00832209	9,30818574	0,57534531	0,09115717	249
-99	-99	-99	-99	-99	-99	-99	-99	-99	-99	-99	-99	-99	-99	-99	-99	-99	-99	-99	-99	-99	-99	250
0,07008502	0,11259776	3,84837663	0,18624195	0,07220537	0,03494549	0,22071399	1,65777705	0,02508989	0,74703617	0,04016966	1,72219678	0,07501121	0,06025605	0,78038070	0,18165211	0,42550559	0,06075934	4,50101384	3,44200693	0,36104486	0,09222170	251
-99	-99	-99	-99	-99	-99	-99	-99	-99	-99	-99	-99	-99	-99	-99	-99	-99	-99	-99	-99	-99	-99	252
-99	-99	-99	-99	-99	-99	-99	-99	-99	-99	-99	-99	-99	-99	-99	-99	-99	-99	-99	-99	-99	-99	253
-99	-99	-99	-99	-99	-99	-99	-99	-99	-99	-99	-99	-99	-99	-99	-99	-99	-99	-99	-99	-99	-99	254
-99	-99	-99	-99	-99	-99	-99	-99	-99	-99	-99	-99	-99	-99	-99	-99	-99	-99	-99	-99	-99	-99	255
0,07414535	0,02780523	1,26356510	0,03932253	0,09249377	0,09230163	0,56233432	0,98248004	0,01955254	0,30981953	0,02334893	1,90461579	0,03891580	0,01410646	0,21308486	0,12927364	1,00660663	0,00912791	5,87463819	1,88622267	0,73892508	0,05341912	256
-99	-99	-99	-99	-99	-99	-99	-99	-99	-99	-99	-99	-99	-99	-99	-99	-99	-99	-99	-99	-99	-99	257
-99	-99	-99	-99	-99	-99	-99	-99	-99	-99	-99	-99	-99	-99	-99	-99	-99	-99	-99	-99	-99	-99	258
0,07410681	0,08222666	3,38874246	0,06976997	0,09731122	0,08081405	0,19462244	1,16211271	0,15418668	0,89735454	0,00600654	2,17610776	0,05265630	0,04693311	0,79100022	0,18825368	0,44403645	0,25751886	2,76781976	14,25849691	0,49200675	0,13896081	259
-99	-99	-99	-99	-99	-99	-99	-99	-99	-99	-99	-99	-99	-99	-99	-99	-99	-99	-99	-99	-99	-99	260
0,10652397	0,04659277	2,92664955	0,14104721	0,14602123	0,08050655	0,09481440	0,94294792	0,08492011	0,80009876	0,01046887	1,85092957	0,04407945	0,03548246	0,17114013	0,10600836	0,37093801	0,05231070	7,30685207	9,30012431	0,10001235	0,13251706	261
0,12621891	0,05038018	3,85571923	0,02568376	0,05027552	0,15062154	0,35013908	1,00765376	0,09233363	0,60290391	0,01955932	1,12349990	0,03316948	0,01802321	0,59790990	0,13773814	0,53403270	0,10160195	4,77003549	5,55197088	0,68254686	0,06801515	262
-99	-99	-99	-99	-99	-99	-99	-99	-99	-99	-99	-99	-99	-99	-99	-99	-99	-99	-99	-99	-99	-99	263
0,06772114	0,05523590	3,40994862	0,04327717	0,06823946	0,07571642	0,40297158	1,82481473	0,03767497	0,08733780	0,00277701	0,44712496	0,03741473	0,02620072	0,70161427	0,18259890	0,40605583	0,16140420	7,75297493	5,96180836	0,27753661	0,11124019	264
0,05899555	0,01603924	3,95245764	0,06996368	0,08818946	0,11733863	0,35471937	0,58105636	0,05075698	0,64472239	0,02306342	0,84075071	0,03836009	0,04221062	0,63452548	0,13676301	0,35867522	0,03943854	3,73407266	5,74278281	0,29052818	0,06948041	265

MPI_FGH_Ds [61711]																					Location_ Treatment	
11	12	27	47	38	23	25	54	45	1	66	22	35	17	57	21	6	8	3	24	33	**Ranking**	15
**Glutathione-S-transferase**	**Bacterial_spot_disease_resistance_protein_4**	**Glutamyl-tRNA(Gln)_amidotransferase_subunit_A**	**(S)-norcoclaurine synthase**	**Cytochrome_P450**	**PR1_protein**	**Beta-D-glucan_exohydrolase**	**SBT4B_protein**	**Ethylene-inducing_xylanase**	**Glucosyltransferase**	**Nbs-lrr_resistance_protein**	**Acidic_class_II_1_3-beta-glucanase**	**Hcr9-OR2A**	**Serine-threonine protein kinase, plant-type**	**Gamma_aminobutyrate_transaminase_isoform1**	**O-methyltransferase**	**Multidrug_resistance_protein_ABC_transporter_family**	**BED_finger-nbs-lrr_resistance_protein**	**S-locus-specific_glycoprotein_S6**	**NADPH-dependent_codeinone_reductase**	**Desacetoxyvindoline_4-hydroxylase**	**Gene product**	**Kinase**
PGSC0003 DMT400004539	PGSC0003 DMT400006231	PGSC0003 DMT400008092	PGSC0003 DMT400008278	PGSC0003 DMT40000854	PGSC0003 DMT400013094	PGSC0003 DMT400015224	PGSC0003 DMT400017633	PGSC0003 DMT400020366	PGSC0003 DMT400021019	PGSC0003 DMT40002160	PGSC0003 DMT40002720	PGSC0003 DMT400027792	PGSC0003 DMT400028434	PGSC0003 DMT40003068	PGSC0003 DMT400031370	PGSC0003 DMT400031714	PGSC0003 DMT40003571	PGSC0003 DMT400036026	PGSC0003 DMT40003748	PGSC0003 DMT400041989	**Transcript ID F1-Individuals**	PGSC0003 DMT40004236
0,90500646	0,23898633	0,11430814	0,65747138	0,34651747	0,60415892	8,42688829	3,07588166	0,07536299	0,02681158	0,04859663	0,08189959	0,00150815	0,04450157	0,88148016	1,07251662	0,18466726	0,01909052	0,08105247	0,83914964	0,08735294	1	0,09632139
-99	-99	-99	-99	-99	-99	-99	-99	-99	-99	-99	-99	-99	-99	-99	-99	-99	-99	-99	-99	-99	2	-99
-99	-99	-99	-99	-99	-99	-99	-99	-99	-99	-99	-99	-99	-99	-99	-99	-99	-99	-99	-99	-99	3	-99
-99	-99	-99	-99	-99	-99	-99	-99	-99	-99	-99	-99	-99	-99	-99	-99	-99	-99	-99	-99	-99	4	-99
-99	-99	-99	-99	-99	-99	-99	-99	-99	-99	-99	-99	-99	-99	-99	-99	-99	-99	-99	-99	-99	5	-99
0,05311452	0,14652817	0,25230663	0,23184781	0,19618002	0,10353927	13,46600500	4,00346724	0,17304870	0,05502572	0,03758368	0,17280897	0,00454108	0,41158149	0,81183025	0,60804500	0,04320224	0,03231281	0,03655595	0,85042151	0,30423330	6	0,06462562
-99	-99	-99	-99	-99	-99	-99	-99	-99	-99	-99	-99	-99	-99	-99	-99	-99	-99	-99	-99	-99	7	-99
-99	-99	-99	-99	-99	-99	-99	-99	-99	-99	-99	-99	-99	-99	-99	-99	-99	-99	-99	-99	-99	8	-99
-99	-99	-99	-99	-99	-99	-99	-99	-99	-99	-99	-99	-99	-99	-99	-99	-99	-99	-99	-99	-99	9	-99
-99	-99	-99	-99	-99	-99	-99	-99	-99	-99	-99	-99	-99	-99	-99	-99	-99	-99	-99	-99	-99	10	-99
-99	-99	-99	-99	-99	-99	-99	-99	-99	-99	-99	-99	-99	-99	-99	-99	-99	-99	-99	-99	-99	11	-99
-99	-99	-99	-99	-99	-99	-99	-99	-99	-99	-99	-99	-99	-99	-99	-99	-99	-99	-99	-99	-99	12	-99
-99	-99	-99	-99	-99	-99	-99	-99	-99	-99	-99	-99	-99	-99	-99	-99	-99	-99	-99	-99	-99	13	-99
-99	-99	-99	-99	-99	-99	-99	-99	-99	-99	-99	-99	-99	-99	-99	-99	-99	-99	-99	-99	-99	14	-99
-99	-99	-99	-99	-99	-99	-99	-99	-99	-99	-99	-99	-99	-99	-99	-99	-99	-99	-99	-99	-99	15	-99
3,53693572	0,25026007	0,12349290	0,42205415	0,09702498	0,11846231	26,27283538	1,06179113	0,11903849	0,34856509	0,02537409	0,28155721	0,00801268	0,89719905	0,60353109	0,84821388	0,15235408	0,00676122	0,03098041	0,89161945	0,59193062	16	0,04460966
1,38799272	0,21763764	0,18946457	0,37113089	0,11399165	0,33915108	0,40276215	3,33341283	0,02956430	0,01557094	0,01935701	0,38877302	0,00625402	0,52123288	0,45977502	0,49175104	0,08705072	0,01831285	0,05908763	0,62894249	0,35898613	17	0,03577263
0,36450234	0,31775792	0,25971478	0,50417623	0,08508213	1,47222686	18,77837085	3,06311599	0,27149546	0,03721428	0,04401076	0,38931235	0,01685126	0,46619324	1,73868912	0,32578679	0,19371398	0,03384297	0,02319568	0,69159531	0,32420989	18	0,08729241
3,73925277	0,29973303	0,11636669	0,31836420	0,18374157	0,31116463	19,26285981	1,84005642	0,09155294	0,01462672	0,00080471	0,09880652	0,01224002	0,35324719	0,99636759	1,16938501	0,33698353	0,00845928	0,10842355	0,16207686	0,21077125	19	0,12911692
-99	-99	-99	-99	-99	-99	-99	-99	-99	-99	-99	-99	-99	-99	-99	-99	-99	-99	-99	-99	-99	20	-99
-99	-99	-99	-99	-99	-99	-99	-99	-99	-99	-99	-99	-99	-99	-99	-99	-99	-99	-99	-99	-99	21	-99
-99	-99	-99	-99	-99	-99	-99	-99	-99	-99	-99	-99	-99	-99	-99	-99	-99	-99	-99	-99	-99	22	-99
-99	-99	-99	-99	-99	-99	-99	-99	-99	-99	-99	-99	-99	-99	-99	-99	-99	-99	-99	-99	-99	23	-99
8,80150356	0,14597063	0,14903778	0,37991537	0,19154430	0,10910204	13,21175302	0,66376755	0,01142826	0,01394363	0,00424727	0,10328839	0,00247347	0,31289489	0,91304002	0,26256563	0,01974663	0,01682500	0,06019344	0,59244371	0,16365732	24	0,05722344
-99	-99	-99	-99	-99	-99	-99	-99	-99	-99	-99	-99	-99	-99	-99	-99	-99	-99	-99	-99	-99	25	-99
-99	-99	-99	-99	-99	-99	-99	-99	-99	-99	-99	-99	-99	-99	-99	-99	-99	-99	-99	-99	-99	26	-99
-99	-99	-99	-99	-99	-99	-99	-99	-99	-99	-99	-99	-99	-99	-99	-99	-99	-99	-99	-99	-99	27	-99
-99	-99	-99	-99	-99	-99	-99	-99	-99	-99	-99	-99	-99	-99	-99	-99	-99	-99	-99	-99	-99	28	-99
-99	-99	-99	-99	-99	-99	-99	-99	-99	-99	-99	-99	-99	-99	-99	-99	-99	-99	-99	-99	-99	29	-99
-99	-99	-99	-99	-99	-99	-99	-99	-99	-99	-99	-99	-99	-99	-99	-99	-99	-99	-99	-99	-99	30	-99
-99	-99	-99	-99	-99	-99	-99	-99	-99	-99	-99	-99	-99	-99	-99	-99	-99	-99	-99	-99	-99	31	-99
-99	-99	-99	-99	-99	-99	-99	-99	-99	-99	-99	-99	-99	-99	-99	-99	-99	-99	-99	-99	-99	32	-99
1,91122814	0,13071531	0,18524416	0,84997953	0,15298902	0,26088755	41,74408842	2,31097235	0,00002937	0,01275759	0,03009150	0,15352016	0,01158579	0,36236110	0,44859953	0,74872094	0,09675634	0,00890913	0,01154570	0,50610197	0,41595518	33	0,07596612
-99	-99	-99	-99	-99	-99	-99	-99	-99	-99	-99	-99	-99	-99	-99	-99	-99	-99	-99	-99	-99	34	-99
3,81253346	0,19871188	0,19912552	0,24978349	0,25913037	0,01442535	9,70514656	0,61121421	0,00002773	0,01390502	0,02301551	0,29093121	0,00258208	0,41545093	0,98606182	0,40888017	0,08053446	0,00711831	0,13666825	1,02296019	0,78881014	35	0,09022994
-99	-99	-99	-99	-99	-99	-99	-99	-99	-99	-99	-99	-99	-99	-99	-99	-99	-99	-99	-99	-99	36	-99
-99	-99	-99	-99	-99	-99	-99	-99	-99	-99	-99	-99	-99	-99	-99	-99	-99	-99	-99	-99	-99	37	-99
-99	-99	-99	-99	-99	-99	-99	-99	-99	-99	-99	-99	-99	-99	-99	-99	-99	-99	-99	-99	-99	38	-99
-99	-99	-99	-99	-99	-99	-99	-99	-99	-99	-99	-99	-99	-99	-99	-99	-99	-99	-99	-99	-99	39	-99
0,89595613	0,08744381	0,18562977	0,24274273	0,12179274	0,12238511	17,64882216	1,74654038	0,06432396	0,03286054	0,03623430	0,19621402	0,00035633	0,47352090	0,76498322	1,22816126	0,18562977	0,02678372	0,06608653	0,72673435	0,40767743	40	0,13778588
-99	-99	-99	-99	-99	-99	-99	-99	-99	-99	-99	-99	-99	-99	-99	-99	-99	-99	-99	-99	-99	41	-99
-99	-99	-99	-99	-99	-99	-99	-99	-99	-99	-99	-99	-99	-99	-99	-99	-99	-99	-99	-99	-99	42	-99
3,10856770	0,15704501	0,26012014	0,35582752	0,23655540	0,92418271	8,25489577	1,26861121	0,11361695	0,02069247	0,01752126	0,24320588	0,02131850	0,53746771	1,49096873	0,86468706	0,06662697	0,00455368	0,02954894	0,10008169	0,15715390	43	0,10868690
-99	-99	-99	-99	-99	-99	-99	-99	-99	-99	-99	-99	-99	-99	-99	-99	-99	-99	-99	-99	-99	44	-99
-99	-99	-99	-99	-99	-99	-99	-99	-99	-99	-99	-99	-99	-99	-99	-99	-99	-99	-99	-99	-99	45	-99
2,20037589	0,36901465	0,13311546	0,33512007	0,16331735	0,12911692	30,05951861	2,05160074	0,00184233	0,02871105	0,02871105	0,30773277	0,00598576	0,45747030	1,37054529	0,55047540	0,08239782	0,02485621	0,04177403	0,38176321	0,23994073	46	0,05072181
-99	-99	-99	-99	-99	-99	-99	-99	-99	-99	-99	-99	-99	-99	-99	-99	-99	-99	-99	-99	-99	47	-99
-99	-99	-99	-99	-99	-99	-99	-99	-99	-99	-99	-99	-99	-99	-99	-99	-99	-99	-99	-99	-99	48	-99
0,54186298	0,24655818	0,23981603	0,62243712	0,35576587	0,40866766	12,72858374	3,47737824	0,00003966	0,29586276	0,03356264	0,06100199	0,01085121	0,94343825	1,50942597	0,56058304	0,20661258	0,02281300	0,00701180	1,05044454	0,91319825	49	0,05847647
2,89386977	0,25971478	0,22515649	0,55593958	0,09672281	0,09632139	32,76310081	2,24855785	0,00198314	0,01587610	0,02775709	0,05664137	0,00698270	1,08072540	0,69063722	0,43497368	0,21583489	0,02324397	0,03269015	0,70027816	0,16188037	50	0,11518302
3,13779261	0,14506285	0,17589056	0,65553740	0,16490991	0,09846467	24,51767041	1,26817162	0,01524785	0,19995538	0,01618443	0,10407893	0,00875152	0,82287774	0,90547706	0,38789820	0,10088265	0,04134194	0,06197156	0,28672707	0,69339532	51	0,01365667
-99	-99	-99	-99	-99	-99	-99	-99	-99	-99	-99	-99	-99	-99	-99	-99	-99	-99	-99	-99	-99	52	-99
-99	-99	-99	-99	-99	-99	-99	-99	-99	-99	-99	-99	-99	-99	-99	-99	-99	-99	-99	-99	-99	53	-99
-99	-99	-99	-99	-99	-99	-99	-99	-99	-99	-99	-99	-99	-99	-99	-99	-99	-99	-99	-99	-99	54	-99
-99	-99	-99	-99	-99	-99	-99	-99	-99	-99	-99	-99	-99	-99	-99	-99	-99	-99	-99	-99	-99	55	-99
-99	-99	-99	-99	-99	-99	-99	-99	-99	-99	-99	-99	-99	-99	-99	-99	-99	-99	-99	-99	-99	56	-99
3,41231303	0,19435283	0,22033186	0,32913469	0,26512599	0,19529812	15,60298336	1,36013514	0,20472370	0,18617742	0,00851221	0,08806728	0,00533147	0,03554400	1,11941889	1,24207303	0,06609798	0,01389539	0,04171615	0,09530859	0,37338866	57	0,07529772
6,46880848	0,18396459	0,29678704	0,77727661	0,29885136	0,28687616	26,00108641	0,43062381	0,07963244	0,46025331	0,00719146	0,21175971	0,01510846	0,63529563	1,44944441	0,68467916	0,11986647	0,02996662	0,08440662	0,53718838	0,55690378	58	0,05131413
-99	-99	-99	-99	-99	-99	-99	-99	-99	-99	-99	-99	-99	-99	-99	-99	-99	-99	-99	-99	-99	59	-99
-99	-99	-99	-99	-99	-99	-99	-99	-99	-99	-99	-99	-99	-99	-99	-99	-99	-99	-99	-99	-99	60	-99
0,51745310	0,21352842	0,17464533	0,49809747	0,22758857	0,29189067	0,80804077	1,14036843	0,04679506	0,29331037	0,03981622	0,09582195	0,00916595	0,35539616	1,00034663	0,50156202	0,14523892	0,01769518	0,04970380	0,50017332	0,76763905	61	0,06243505
-99	-99	-99	-99	-99	-99	-99	-99	-99	-99	-99	-99	-99	-99	-99	-99	-99	-99	-99	-99	-99	62	-99
-99	-99	-99	-99	-99	-99	-99	-99	-99	-99	-99	-99	-99	-99	-99	-99	-99	-99	-99	-99	-99	63	-99
-99	-99	-99	-99	-99	-99	-99	-99	-99	-99	-99	-99	-99	-99	-99	-99	-99	-99	-99	-99	-99	64	-99
0,63079800	0,08988661	0,18266220	0,49388601	0,19415086	0,16808350	9,42994921	1,29168116	0,05860835	0,01505358	0,01408448	0,10462141	0,00094085	0,48674903	1,12525346	0,98845692	0,09573896	0,01505358	0,09337952	0,94098915	0,33130916	65	0,03220102
-99	-99	-99	-99	-99	-99	-99	-99	-99	-99	-99	-99	-99	-99	-99	-99	-99	-99	-99	-99	-99	66	-99
-99	-99	-99	-99	-99	-99	-99	-99	-99	-99	-99	-99	-99	-99	-99	-99	-99	-99	-99	-99	-99	67	-99
-99	-99	-99	-99	-99	-99	-99	-99	-99	-99	-99	-99	-99	-99	-99	-99	-99	-99	-99	-99	-99	68	-99
-99	-99	-99	-99	-99	-99	-99	-99	-99	-99	-99	-99	-99	-99	-99	-99	-99	-99	-99	-99	-99	69	-99
-99	-99	-99	-99	-99	-99	-99	-99	-99	-99	-99	-99	-99	-99	-99	-99	-99	-99	-99	-99	-99	70	-99

-99	-99	-99	-99	-99	-99	-99	-99	-99	-99	-99	-99	-99	-99	-99	-99	-99	-99	-99	-99	-99	71	-99
-99	-99	-99	-99	-99	-99	-99	-99	-99	-99	-99	-99	-99	-99	-99	-99	-99	-99	-99	-99	-99	72	-99
-99	-99	-99	-99	-99	-99	-99	-99	-99	-99	-99	-99	-99	-99	-99	-99	-99	-99	-99	-99	-99	73	-99
-99	-99	-99	-99	-99	-99	-99	-99	-99	-99	-99	-99	-99	-99	-99	-99	-99	-99	-99	-99	-99	74	-99
4,65409287	0,19204283	0,18692093	0,51139147	0,11594399	0,06414586	15,92807484	1,27588650	0,04884992	0,18653264	0,02500742	0,09783537	0,01336404	0,03435110	1,18632558	0,39763041	0,10051620	0,01723518	0,02850779	1,00800305	0,38461848	75	0,10587985
-99	-99	-99	-99	-99	-99	-99	-99	-99	-99	-99	-99	-99	-99	-99	-99	-99	-99	-99	-99	-99	76	-99
-99	-99	-99	-99	-99	-99	-99	-99	-99	-99	-99	-99	-99	-99	-99	-99	-99	-99	-99	-99	-99	77	-99
-99	-99	-99	-99	-99	-99	-99	-99	-99	-99	-99	-99	-99	-99	-99	-99	-99	-99	-99	-99	-99	78	-99
-99	-99	-99	-99	-99	-99	-99	-99	-99	-99	-99	-99	-99	-99	-99	-99	-99	-99	-99	-99	-99	79	-99
-99	-99	-99	-99	-99	-99	-99	-99	-99	-99	-99	-99	-99	-99	-99	-99	-99	-99	-99	-99	-99	80	-99
-99	-99	-99	-99	-99	-99	-99	-99	-99	-99	-99	-99	-99	-99	-99	-99	-99	-99	-99	-99	-99	81	-99
-99	-99	-99	-99	-99	-99	-99	-99	-99	-99	-99	-99	-99	-99	-99	-99	-99	-99	-99	-99	-99	82	-99
-99	-99	-99	-99	-99	-99	-99	-99	-99	-99	-99	-99	-99	-99	-99	-99	-99	-99	-99	-99	-99	83	-99
0,87645383	0,26845459	0,13780976	0,19354622	0,38070621	0,07193063	8,62634849	1,07456295	0,03330194	0,03230161	0,02589379	0,43249339	0,01800760	0,62752724	1,54729684	1,17263173	0,10812335	0,02187990	0,01122415	1,27965065	0,78444815	84	0,04564973
-99	-99	-99	-99	-99	-99	-99	-99	-99	-99	-99	-99	-99	-99	-99	-99	-99	-99	-99	-99	-99	85	-99
-99	-99	-99	-99	-99	-99	-99	-99	-99	-99	-99	-99	-99	-99	-99	-99	-99	-99	-99	-99	-99	86	-99
2,65967506	0,12001195	0,08129159	0,25353380	0,16157210	0,04853771	14,90009888	1,69143765	0,08017243	0,30550121	0,00747998	0,08011688	0,00004840	0,29550409	0,99740407	0,46951723	0,05517850	0,00926656	0,05905692	0,42256641	0,81408424	87	0,08328786
2,27836675	0,21538654	0,26480458	0,14660437	0,25384153	0,08455301	20,32241572	1,84548499	0,17739042	0,12065838	0,02762274	0,10132064	0,00465501	0,58357905	0,87417862	0,34364717	0,19117953	0,01326713	0,00007801	0,23455530	0,19778433	88	0,13231055
4,09113175	0,12011598	0,21441831	0,43603021	0,22980785	0,18345524	7,42041565	1,72967382	0,09642159	0,02639667	0,01840830	0,09709225	0,00553002	0,52540433	1,33468566	1,33191314	0,10802971	0,01681042	0,06966124	0,80412963	0,51281132	89	0,01633941
-99	-99	-99	-99	-99	-99	-99	-99	-99	-99	-99	-99	-99	-99	-99	-99	-99	-99	-99	-99	-99	90	-99
-99	-99	-99	-99	-99	-99	-99	-99	-99	-99	-99	-99	-99	-99	-99	-99	-99	-99	-99	-99	-99	91	-99
-99	-99	-99	-99	-99	-99	-99	-99	-99	-99	-99	-99	-99	-99	-99	-99	-99	-99	-99	-99	-99	92	-99
-99	-99	-99	-99	-99	-99	-99	-99	-99	-99	-99	-99	-99	-99	-99	-99	-99	-99	-99	-99	-99	93	-99
-99	-99	-99	-99	-99	-99	-99	-99	-99	-99	-99	-99	-99	-99	-99	-99	-99	-99	-99	-99	-99	94	-99
-99	-99	-99	-99	-99	-99	-99	-99	-99	-99	-99	-99	-99	-99	-99	-99	-99	-99	-99	-99	-99	95	-99
-99	-99	-99	-99	-99	-99	-99	-99	-99	-99	-99	-99	-99	-99	-99	-99	-99	-99	-99	-99	-99	96	-99
-99	-99	-99	-99	-99	-99	-99	-99	-99	-99	-99	-99	-99	-99	-99	-99	-99	-99	-99	-99	-99	97	-99
-99	-99	-99	-99	-99	-99	-99	-99	-99	-99	-99	-99	-99	-99	-99	-99	-99	-99	-99	-99	-99	98	-99
3,60312824	0,13123733	0,14411076	0,51059453	0,20380340	0,42935711	9,14035374	2,50229174	0,01089832	0,26595425	0,01324186	0,10253938	0,00872427	1,28542907	1,22031196	1,04120201	0,15075210	0,02920279	0,01620126	0,88102203	0,43625694	99	0,09164818
4,12244430	0,17452432	0,24358547	0,51924956	0,21953152	0,02227393	16,96512318	1,15869431	0,00007496	0,56155530	0,02700743	0,04844528	0,00627791	0,42824253	1,60491846	0,41394184	0,24630196	0,02951311	0,15235408	1,15468553	0,36513453	100	0,08570362
-99	-99	-99	-99	-99	-99	-99	-99	-99	-99	-99	-99	-99	-99	-99	-99	-99	-99	-99	-99	-99	101	-99
-99	-99	-99	-99	-99	-99	-99	-99	-99	-99	-99	-99	-99	-99	-99	-99	-99	-99	-99	-99	-99	102	-99
3,42119420	0,32930584	0,34856509	0,36843959	0,19164390	0,08232646	35,61714568	1,74533019	0,11075932	0,04491995	0,03759019	0,34591752	0,01516091	1,14909653	0,93594782	1,48916127	0,11870890	0,01332704	0,06477137	0,97840250	0,41308197	103	0,05319742
5,89299074	0,10889427	0,15582515	0,50592660	0,52522227	1,06289567	3,79210786	1,32500702	0,05974658	0,03649898	0,00417502	0,12985739	0,02406366	0,43077308	1,24314967	0,46781175	0,06578945	0,00567958	0,07748173	0,12293776	0,23848989	104	0,08668944
-99	-99	-99	-99	-99	-99	-99	-99	-99	-99	-99	-99	-99	-99	-99	-99	-99	-99	-99	-99	-99	105	-99
0,72585335	0,17262939	0,12532534	0,44898838	0,15493661	0,26439192	17,93554899	1,22922584	0,05146552	0,28238787	0,02718114	0,08389624	0,00470611	0,42742702	1,11245733	0,91557502	0,18133760	0,01985643	0,12198283	0,80259830	0,56243177	106	0,07592664
2,51795064	0,26836157	0,23410863	0,46049264	0,08951356	0,03403705	28,25139105	0,90704749	0,00002474	0,30592502	0,03340598	0,11393240	0,00677412	0,90956585	0,87858270	0,28504271	0,15835672	0,03732407	0,03215641	0,50043340	0,18002261	107	0,07884975
-99	-99	-99	-99	-99	-99	-99	-99	-99	-99	-99	-99	-99	-99	-99	-99	-99	-99	-99	-99	-99	108	-99
-99	-99	-99	-99	-99	-99	-99	-99	-99	-99	-99	-99	-99	-99	-99	-99	-99	-99	-99	-99	-99	109	-99
3,76656650	0,31561770	0,33292059	0,27648057	0,23557364	0,34133257	16,99749217	1,06751031	0,00001475	0,02987847	0,01908060	0,12321502	0,00698391	0,61653299	1,18365612	0,65033276	0,19002353	0,03110413	0,10310956	0,29062888	0,16090154	110	0,07382483
-99	-99	-99	-99	-99	-99	-99	-99	-99	-99	-99	-99	-99	-99	-99	-99	-99	-99	-99	-99	-99	111	-99
-99	-99	-99	-99	-99	-99	-99	-99	-99	-99	-99	-99	-99	-99	-99	-99	-99	-99	-99	-99	-99	112	-99
-99	-99	-99	-99	-99	-99	-99	-99	-99	-99	-99	-99	-99	-99	-99	-99	-99	-99	-99	-99	-99	113	-99
3,08121638	0,29802391	0,24904874	1,07065970	0,26785052	0,50575129	26,78772207	2,13835295	0,06789739	0,02001881	0,02236676	0,12011598	0,00332253	1,30721822	2,07268374	1,09391451	0,21102707	0,06119256	0,08522970	0,94704179	0,31003436	114	0,22837870
-99	-99	-99	-99	-99	-99	-99	-99	-99	-99	-99	-99	-99	-99	-99	-99	-99	-99	-99	-99	-99	115	-99
-99	-99	-99	-99	-99	-99	-99	-99	-99	-99	-99	-99	-99	-99	-99	-99	-99	-99	-99	-99	-99	116	-99
-99	-99	-99	-99	-99	-99	-99	-99	-99	-99	-99	-99	-99	-99	-99	-99	-99	-99	-99	-99	-99	117	-99
-99	-99	-99	-99	-99	-99	-99	-99	-99	-99	-99	-99	-99	-99	-99	-99	-99	-99	-99	-99	-99	118	-99
-99	-99	-99	-99	-99	-99	-99	-99	-99	-99	-99	-99	-99	-99	-99	-99	-99	-99	-99	-99	-99	119	-99
3,90142185	0,18946457	0,21375054	0,57594382	0,17837681	0,23553282	21,69062923	2,91399823	0,07330218	0,37267760	0,00291154	0,02629623	0,00589620	0,07204290	1,98756219	0,48063046	0,07938444	0,00946619	0,05215683	0,18893999	0,42307930	120	0,07861781
2,59907812	0,22640850	0,22672258	0,50208377	0,12074204	0,24553490	23,05487598	1,03454758	0,13840807	0,01587610	0,02185338	0,12789218	0,01119501	0,56213946	1,08748839	0,50944560	0,16735691	0,02103594	0,02229710	0,49757986	0,24333234	121	0,08431891
2,30337611	0,17772888	0,20037161	0,30751954	0,10827334	0,12223675	23,50262508	1,50811872	0,05100385	0,26530982	0,03974039	0,18158916	0,01199644	0,68908316	1,30744477	0,98333166	0,17286887	0,01663943	0,08262659	0,72083951	0,22371750	122	0,08691506
-99	-99	-99	-99	-99	-99	-99	-99	-99	-99	-99	-99	-99	-99	-99	-99	-99	-99	-99	-99	-99	123	-99
-99	-99	-99	-99	-99	-99	-99	-99	-99	-99	-99	-99	-99	-99	-99	-99	-99	-99	-99	-99	-99	124	-99
-99	-99	-99	-99	-99	-99	-99	-99	-99	-99	-99	-99	-99	-99	-99	-99	-99	-99	-99	-99	-99	125	-99
-99	-99	-99	-99	-99	-99	-99	-99	-99	-99	-99	-99	-99	-99	-99	-99	-99	-99	-99	-99	-99	126	-99
3,68777337	0,12368565	0,17250977	0,53431040	0,25117243	0,76352643	0,24127494	1,02864844	0,04306770	0,23144640	0,02974415	0,25931005	0,00778952	0,34192457	1,48426634	1,67917198	0,14356241	0,01856527	0,04755530	0,44805570	0,75930426	127	0,12463231
-99	-99	-99	-99	-99	-99	-99	-99	-99	-99	-99	-99	-99	-99	-99	-99	-99	-99	-99	-99	-99	128	-99
-99	-99	-99	-99	-99	-99	-99	-99	-99	-99	-99	-99	-99	-99	-99	-99	-99	-99	-99	-99	-99	129	-99
-99	-99	-99	-99	-99	-99	-99	-99	-99	-99	-99	-99	-99	-99	-99	-99	-99	-99	-99	-99	-99	130	-99
-99	-99	-99	-99	-99	-99	-99	-99	-99	-99	-99	-99	-99	-99	-99	-99	-99	-99	-99	-99	-99	131	-99
-99	-99	-99	-99	-99	-99	-99	-99	-99	-99	-99	-99	-99	-99	-99	-99	-99	-99	-99	-99	-99	132	-99
-99	-99	-99	-99	-99	-99	-99	-99	-99	-99	-99	-99	-99	-99	-99	-99	-99	-99	-99	-99	-99	133	-99
-99	-99	-99	-99	-99	-99	-99	-99	-99	-99	-99	-99	-99	-99	-99	-99	-99	-99	-99	-99	-99	134	-99
-99	-99	-99	-99	-99	-99	-99	-99	-99	-99	-99	-99	-99	-99	-99	-99	-99	-99	-99	-99	-99	135	-99
-99	-99	-99	-99	-99	-99	-99	-99	-99	-99	-99	-99	-99	-99	-99	-99	-99	-99	-99	-99	-99	136	-99
-99	-99	-99	-99	-99	-99	-99	-99	-99	-99	-99	-99	-99	-99	-99	-99	-99	-99	-99	-99	-99	137	-99
3,35311044	0,25428178	0,14146333	0,40234360	0,15004842	0,13235641	75,40038579	2,01880281	0,00002763	0,16081791	0,01702148	0,27272143	0,00921053	0,65406232	1,37411238	0,74509692	0,19635007	0,04501345	0,02397210	0,49568654	0,29617053	138	0,18294730
-99	-99	-99	-99	-99	-99	-99	-99	-99	-99	-99	-99	-99	-99	-99	-99	-99	-99	-99	-99	-99	139	-99
3,67756286	0,16422549	0,14924453	0,32169156	0,16559718	0,24345888	63,37096150	1,71802377	0,04619082	0,01590639	0,01550363	0,13182994	0,00025358	0,43974833	0,93222493	0,77902957	0,14257076	0,00003537	0,04926647	0,81606162	0,13752349	140	0,09997769
5,06214910	0,21297411	0,25948985	0,60699225	0,24345888	0,05554304	14,35767260	1,74321438	0,00002131	0,01346866	0,01740626	0,08003362	0,00934720	0,78608106	1,34048028	0,35349213	0,09283097	0,00617969	0,00631391	0,31419889	0,52476740	141	0,13857606
3,38111714	0,17622616	0,14696046	0,42735295	0,27595406	0,23512502	26,65806205	3,90006995	0,04150704	0,02519882	0,02555058	0,09147365	0,00984427	0,50295457	1,30450276	0,40711266	0,18627423	0,02807637	0,08399807	0,51210090	0,18731002	142	0,12530362
5,45186561	0,14316493	0,12645973	0,22232622	0,34406427	0,09617128	9,20074064	0,76617720	0,03083580	0,26003000	0,02536090	0,10879997	0,00393274	0,35570422	1,50707373	1,05537082	0,10250385	0,01418736	0,03187900	0,69291486	0,60952192	143	0,06833412
-99	-99	-99	-99	-99	-99	-99	-99	-99	-99	-99	-99	-99	-99	-99	-99	-99	-99	-99	-99	-99	144	-99
3,56524198	0,33010571	0,23100564	0,14865089	0,19357976	0,35973340	35,92708551	3,40581483	0,06939618	0,54487606	0,03052214	0,20819394	0,00685798	0,42839098	1,57680035	0,76207242	0,30821308	0,03008108	0,13103281	0,95395955	0,57874511	145	0,06624705
-99	-99	-99	-99	-99	-99	-99	-99	-99	-99	-99	-99	-99	-99	-99	-99	-99	-99	-99	-99	-99	146	-99
7,46038457	0,26247465	0,19550129	0,45352375	0,42432747	0,10793615	19,17626705	2,06801980	0,06476014	0,27004093	0,02914213	0,18910377	0,02023855	0,66032584	1,57707361	1,79533118	0,17413161	0,04161507	0,12210972	0,24069032	1,60798060	147	0,08078605
-99	-99	-99	-99	-99	-99	-99	-99	-99	-99	-99	-99	-99	-99	-99	-99	-99	-99	-99	-99	-99	148	-99
-99	-99	-99	-99	-99	-99	-99	-99	-99	-99	-99	-99	-99	-99	-99	-99	-99	-99	-99	-99	-99	149	-99
-99	-99	-99	-99	-99	-99	-99	-99	-99	-99	-99	-99	-99	-99	-99	-99	-99	-99	-99	-99	-99	150	-99
-99	-99	-99	-99	-99	-99	-99	-99	-99	-99	-99	-99	-99	-99	-99	-99	-99	-99	-99	-99	-99	151	-99
1,75746992	0,17586009	0,10788005	0,61621256	0,18231435	0,49775234	5,95664508	4,39084924	0,00003437	0,01603647	0,05022328	0,21983607	0,00921692	0,61365513	1,34303788	1,29011529	0,16776341	0,02505947	0,09096781	1,29369722	0,38408566	152	0,06733498
-99	-99	-99	-99	-99	-99	-99	-99	-99	-99	-99	-99	-99	-99	-99	-99	-99	-99	-99	-99	-99	153	-99

-99	-99	-99	-99	-99	-99	-99	-99	-99	-99	-99	-99	-99	-99	-99	-99	-99	-99	-99	-99	-99	154	-99
-99	-99	-99	-99	-99	-99	-99	-99	-99	-99	-99	-99	-99	-99	-99	-99	-99	-99	-99	-99	-99	155	-99
-99	-99	-99	-99	-99	-99	-99	-99	-99	-99	-99	-99	-99	-99	-99	-99	-99	-99	-99	-99	-99	156	-99
-99	-99	-99	-99	-99	-99	-99	-99	-99	-99	-99	-99	-99	-99	-99	-99	-99	-99	-99	-99	-99	157	-99
-99	-99	-99	-99	-99	-99	-99	-99	-99	-99	-99	-99	-99	-99	-99	-99	-99	-99	-99	-99	-99	158	-99
2,08132174	0,00603471	0,11490393	0,94115223	0,28987443	0,48046392	10,24963053	1,92452178	0,12635021	0,23758242	0,02014408	0,24089895	0,00526081	0,03759019	0,91098550	0,54809581	0,06572108	0,00076117	0,01619284	0,28509211	1,07810672	159	0,08052050
3,40994862	0,32236120	0,38870565	0,20715033	0,27239082	0,87100325	42,24620957	3,31670339	0,00529832	0,02128159	0,04467929	0,47524719	0,00719270	0,10538563	0,84894912	1,74079944	0,08165868	0,01473867	0,08886444	0,54402694	0,28004877	160	0,08601607
2,11917099	0,00484765	0,19284318	0,93919719	0,20639787	1,13721103	14,68732747	1,12155471	0,09989110	0,30428603	0,04023236	0,15620364	0,00099605	0,09756449	0,92690925	0,69135567	0,07502421	0,00413901	0,00566582	0,39188439	0,66873197	161	0,07815598
-99	-99	-99	-99	-99	-99	-99	-99	-99	-99	-99	-99	-99	-99	-99	-99	-99	-99	-99	-99	-99	162	-99
-99	-99	-99	-99	-99	-99	-99	-99	-99	-99	-99	-99	-99	-99	-99	-99	-99	-99	-99	-99	-99	163	-99
10,42156473	0,17992905	0,28727413	0,15512467	0,17745191	0,11022321	11,25504563	0,13990312	0,12999247	0,15620364	0,02434046	0,03571069	0,01266947	0,77351437	1,25223037	0,47220984	0,17392051	0,02277350	0,06553911	0,33552682	0,05923115	164	0,05777143
-99	-99	-99	-99	-99	-99	-99	-99	-99	-99	-99	-99	-99	-99	-99	-99	-99	-99	-99	-99	-99	165	-99
-99	-99	-99	-99	-99	-99	-99	-99	-99	-99	-99	-99	-99	-99	-99	-99	-99	-99	-99	-99	-99	166	-99
11,44979609	0,40845527	0,39673566	0,33430805	0,29346289	0,81577884	0,86768903	14,88977450	0,27079068	0,01157575	0,04770387	0,10622903	0,00192791	0,51450250	1,02189715	63,61301231	0,21113681	0,02203972	0,11448655	0,45415290	0,30719997	167	0,04347259
-99	-99	-99	-99	-99	-99	-99	-99	-99	-99	-99	-99	-99	-99	-99	-99	-99	-99	-99	-99	-99	168	-99
-99	-99	-99	-99	-99	-99	-99	-99	-99	-99	-99	-99	-99	-99	-99	-99	-99	-99	-99	-99	-99	169	-99
-99	-99	-99	-99	-99	-99	-99	-99	-99	-99	-99	-99	-99	-99	-99	-99	-99	-99	-99	-99	-99	170	-99
6,40079010	0,19280976	0,21512543	0,22770691	0,15857640	0,06342740	17,30659496	1,02260572	0,09019867	0,02347469	0,02679764	0,14991847	0,00467846	0,39317677	0,39563720	1,31699512	0,26836157	0,02830597	0,02799378	0,90767643	0,69797632	171	0,05868965
4,10889368	0,18553329	0,26660024	0,75092983	0,12884871	0,42891093	8,32096009	1,97485948	0,01605037	0,01590639	0,03841331	0,18184107	0,00502295	0,41660441	1,61860393	0,41258120	0,09295975	0,02665870	0,07524555	0,90171911	0,43701357	172	0,07876781
-99	-99	-99	-99	-99	-99	-99	-99	-99	-99	-99	-99	-99	-99	-99	-99	-99	-99	-99	-99	-99	173	-99
7,98753307	0,12325773	0,11758289	0,38951479	0,22094359	0,78065120	7,43199742	0,91748088	0,00005100	0,21896163	0,02847323	0,33396064	0,01288423	0,45368095	1,33306765	1,14135691	0,23160688	0,02243275	0,09928709	0,45842258	0,40074322	174	0,26220189
3,97305840	0,29529933	0,31409002	0,40507192	0,23803572	0,31605554	17,11571908	6,55797317	0,13862410	0,03953434	0,03967159	0,11385346	0,01041820	0,01858458	2,04379430	0,49115490	0,19523045	0,03470410	0,02672345	1,02615595	0,49217729	175	0,13949159
2,78562296	0,11125947	0,14518859	0,60248616	0,08609063	0,10511205	34,27298634	1,52520165	0,06168228	0,18724512	0,02326008	0,10569654	0,00343613	0,44411340	0,88884268	0,24965367	0,10294888	0,01789872	0,00593721	0,59336840	0,68396765	176	0,09732809
3,10587550	0,14092505	0,22988751	0,21778855	0,08264091	0,12082576	7,45909189	1,47836243	0,00002396	0,20718623	0,02046071	0,12736140	0,00284767	0,56409107	1,33145161	0,37216131	0,20208022	0,02548867	0,03836674	0,84557229	0,49175104	177	0,03572307
-99	-99	-99	-99	-99	-99	-99	-99	-99	-99	-99	-99	-99	-99	-99	-99	-99	-99	-99	-99	-99	178	-99
-99	-99	-99	-99	-99	-99	-99	-99	-99	-99	-99	-99	-99	-99	-99	-99	-99	-99	-99	-99	-99	179	-99
-99	-99	-99	-99	-99	-99	-99	-99	-99	-99	-99	-99	-99	-99	-99	-99	-99	-99	-99	-99	-99	180	-99
-99	-99	-99	-99	-99	-99	-99	-99	-99	-99	-99	-99	-99	-99	-99	-99	-99	-99	-99	-99	-99	181	-99
4,10035835	0,30139972	0,23224992	0,28376135	0,37833863	0,43731659	11,96501251	1,32477743	0,15525913	0,01322810	0,02817872	0,14456097	0,00880629	1,11786812	0,90109430	0,44219358	0,15101356	0,02417651	0,21594712	0,31485293	0,73293136	182	0,03569213
4,50569607	0,04455558	0,18196716	0,17772888	0,13516092	0,05678879	6,27558521	1,79098096	0,07369702	0,06991520	0,01672036	0,15174806	0,00346903	0,40661913	0,85485403	0,39550011	0,06532369	0,01074456	0,00006222	0,85248715	0,18903824	183	0,10715343
-99	-99	-99	-99	-99	-99	-99	-99	-99	-99	-99	-99	-99	-99	-99	-99	-99	-99	-99	-99	-99	184	-99
-99	-99	-99	-99	-99	-99	-99	-99	-99	-99	-99	-99	-99	-99	-99	-99	-99	-99	-99	-99	-99	185	-99
-99	-99	-99	-99	-99	-99	-99	-99	-99	-99	-99	-99	-99	-99	-99	-99	-99	-99	-99	-99	-99	186	-99
-99	-99	-99	-99	-99	-99	-99	-99	-99	-99	-99	-99	-99	-99	-99	-99	-99	-99	-99	-99	-99	187	-99
-99	-99	-99	-99	-99	-99	-99	-99	-99	-99	-99	-99	-99	-99	-99	-99	-99	-99	-99	-99	-99	188	-99
0,85071630	0,13516092	0,12105629	0,08064618	0,11255874	0,10081275	15,90876568	2,04025574	0,00516058	0,32959128	0,04063171	0,16582690	0,00013788	0,64338312	0,79982152	1,20978443	0,14516343	0,02546660	0,05387463	0,15280355	0,25769742	189	0,09570579
-99	-99	-99	-99	-99	-99	-99	-99	-99	-99	-99	-99	-99	-99	-99	-99	-99	-99	-99	-99	-99	190	-99
-99	-99	-99	-99	-99	-99	-99	-99	-99	-99	-99	-99	-99	-99	-99	-99	-99	-99	-99	-99	-99	191	-99
-99	-99	-99	-99	-99	-99	-99	-99	-99	-99	-99	-99	-99	-99	-99	-99	-99	-99	-99	-99	-99	192	-99
-99	-99	-99	-99	-99	-99	-99	-99	-99	-99	-99	-99	-99	-99	-99	-99	-99	-99	-99	-99	-99	193	-99
-99	-99	-99	-99	-99	-99	-99	-99	-99	-99	-99	-99	-99	-99	-99	-99	-99	-99	-99	-99	-99	194	-99
-99	-99	-99	-99	-99	-99	-99	-99	-99	-99	-99	-99	-99	-99	-99	-99	-99	-99	-99	-99	-99	195	-99
-99	-99	-99	-99	-99	-99	-99	-99	-99	-99	-99	-99	-99	-99	-99	-99	-99	-99	-99	-99	-99	196	-99
-99	-99	-99	-99	-99	-99	-99	-99	-99	-99	-99	-99	-99	-99	-99	-99	-99	-99	-99	-99	-99	197	-99
0,82602079	0,22960883	0,27495168	0,38348711	0,13446010	0,12773714	36,91793620	1,04336938	0,00001137	0,25868172	0,02954894	0,14602123	0,02127422	0,22117343	0,96476535	0,83871351	0,31170431	0,01371834	0,10440408	0,77177381	0,33873994	198	0,07052360
7,67012452	0,32113460	0,25993990	0,58085501	0,22691911	0,22691911	23,70714455	1,63382108	0,13814450	0,03387231	0,04014182	0,13196708	0,00662093	0,09653862	1,14254422	0,76062117	0,15857640	0,02998220	0,03874758	0,17619562	0,17340892	199	0,06816854
-99	-99	-99	-99	-99	-99	-99	-99	-99	-99	-99	-99	-99	-99	-99	-99	-99	-99	-99	-99	-99	200	-99
-99	-99	-99	-99	-99	-99	-99	-99	-99	-99	-99	-99	-99	-99	-99	-99	-99	-99	-99	-99	-99	201	-99
3,91768119	0,12586944	0,23439277	0,53886657	0,19237590	0,27874155	0,95992926	2,82450881	0,00002245	0,21197999	0,01073897	0,21080778	0,01059847	0,85500218	1,21419488	0,77003717	0,18326459	0,00243477	0,04123462	0,47008710	0,16199262	202	0,07536299
0,90000193	0,18920210	0,09258999	0,40668960	0,17899609	0,07753546	10,93589962	1,23970770	0,04283696	0,33355579	0,01754252	0,18162063	0,00587987	0,23341999	0,85737604	0,62286871	0,04553122	0,00382586	0,04796082	0,49861563	0,68586664	203	0,08049260
-99	-99	-99	-99	-99	-99	-99	-99	-99	-99	-99	-99	-99	-99	-99	-99	-99	-99	-99	-99	-99	204	-99
-99	-99	-99	-99	-99	-99	-99	-99	-99	-99	-99	-99	-99	-99	-99	-99	-99	-99	-99	-99	-99	205	-99
-99	-99	-99	-99	-99	-99	-99	-99	-99	-99	-99	-99	-99	-99	-99	-99	-99	-99	-99	-99	-99	206	-99
-99	-99	-99	-99	-99	-99	-99	-99	-99	-99	-99	-99	-99	-99	-99	-99	-99	-99	-99	-99	-99	207	-99
-99	-99	-99	-99	-99	-99	-99	-99	-99	-99	-99	-99	-99	-99	-99	-99	-99	-99	-99	-99	-99	208	-99
0,82759682	0,12578223	0,23618676	0,30249852	0,46748760	0,24844527	9,65315136	1,86606598	0,08443588	0,01552783	0,03248686	0,07705327	0,00298925	0,31057207	1,22433639	1,00625782	0,09855002	0,02564818	0,07376090	0,70808772	0,27662434	209	0,11646755
-99	-99	-99	-99	-99	-99	-99	-99	-99	-99	-99	-99	-99	-99	-99	-99	-99	-99	-99	-99	-99	210	-99
-99	-99	-99	-99	-99	-99	-99	-99	-99	-99	-99	-99	-99	-99	-99	-99	-99	-99	-99	-99	-99	211	-99
2,08710040	0,12323637	0,15151158	0,64909430	0,22774638	0,07875416	10,11552996	1,92185568	0,05825396	0,03063872	0,01721130	0,22585989	0,00178174	0,32342430	1,05226640	0,55613229	0,09365501	0,00244576	0,01307994	0,48784678	0,42735295	212	0,07559843
-99	-99	-99	-99	-99	-99	-99	-99	-99	-99	-99	-99	-99	-99	-99	-99	-99	-99	-99	-99	-99	213	-99
10,64976728	0,17871715	0,17013478	0,23032613	0,28712483	0,02691865	4,25969460	0,40549331	0,07975673	0,36317832	0,01186413	0,12698676	0,00028001	0,25680585	1,04300784	0,22155703	0,10400682	0,02306342	0,00413112	1,27699245	1,46484701	214	0,06175715
-99	-99	-99	-99	-99	-99	-99	-99	-99	-99	-99	-99	-99	-99	-99	-99	-99	-99	-99	-99	-99	215	-99
4,81988926	0,15920968	0,30778610	0,28697560	0,20320390	0,09394759	10,71826882	0,94737007	0,07812889	0,31490749	0,01995646	0,10409697	0,01176789	0,55057080	1,18755967	0,90187538	0,28538868	0,01423908	0,03127167	0,51691537	0,26443774	216	0,10020317
-99	-99	-99	-99	-99	-99	-99	-99	-99	-99	-99	-99	-99	-99	-99	-99	-99	-99	-99	-99	-99	217	-99
-99	-99	-99	-99	-99	-99	-99	-99	-99	-99	-99	-99	-99	-99	-99	-99	-99	-99	-99	-99	-99	218	-99
-99	-99	-99	-99	-99	-99	-99	-99	-99	-99	-99	-99	-99	-99	-99	-99	-99	-99	-99	-99	-99	219	-99
3,10103541	0,28063172	0,26734044	0,28435203	0,26530982	0,17096228	25,55883193	1,05463955	0,05493045	0,25291947	0,02332062	0,04660892	0,01195493	0,58835141	0,75667726	0,43459696	0,22294349	0,00751897	0,03720783	0,18864555	0,17528203	220	0,05413667
0,96126093	0,22082876	0,06787387	0,33471381	0,12405055	0,02305143	37,53071838	2,06766147	0,05759151	0,29320874	0,02853745	0,09226965	0,00910579	1,03383074	1,43494953	0,47929972	0,19012234	0,01732501	0,04143518	0,13221887	0,28519093	221	0,07726720
3,55721950	0,11373514	0,21759993	0,52078146	0,13657355	1,46789625	10,38730868	2,47212210	0,00558878	0,02703084	0,04430920	0,20218530	0,00193259	0,36317832	1,62534953	0,50269317	0,18773246	0,00179693	0,04303786	0,59863561	0,40269236	222	0,05948831
0,74161894	0,20288723	0,24261657	0,63189204	0,16479564	0,83668124	13,23925467	3,09030664	0,02487344	0,39385869	0,04667358	0,13780976	0,00450190	0,07876781	1,08504133	0,60029768	0,11914167	0,02271438	0,02394304	0,61974645	0,39113811	223	0,11644737
-99	-99	-99	-99	-99	-99	-99	-99	-99	-99	-99	-99	-99	-99	-99	-99	-99	-99	-99	-99	-99	224	-99
3,39461980	0,09600478	0,24286896	0,59967386	0,37637690	0,23088558	14,02812222	1,05720122	0,02572384	0,01611167	0,02182688	0,17729823	0,00663931	0,09076312	1,22031196	0,42285941	0,05227446	0,01237224	0,04426315	1,10439021	0,33315143	225	0,10147878
0,54252067	0,22937022	0,22109679	0,32392910	0,17809884	0,25538576	0,40859685	4,18941847	0,01837324	0,01912694	0,03068654	0,09977001	0,00417430	1,26993090	1,21314332	1,48838731	0,09092054	0,02763710	0,09866964	0,78011029	1,19478415	226	0,11147175
-99	-99	-99	-99	-99	-99	-99	-99	-99	-99	-99	-99	-99	-99	-99	-99	-99	-99	-99	-99	-99	227	-99
-99	-99	-99	-99	-99	-99	-99	-99	-99	-99	-99	-99	-99	-99	-99	-99	-99	-99	-99	-99	-99	228	-99
-99	-99	-99	-99	-99	-99	-99	-99	-99	-99	-99	-99	-99	-99	-99	-99	-99	-99	-99	-99	-99	229	-99
-99	-99	-99	-99	-99	-99	-99	-99	-99	-99	-99	-99	-99	-99	-99	-99	-99	-99	-99	-99	-99	230	-99
-99	-99	-99	-99	-99	-99	-99	-99	-99	-99	-99	-99	-99	-99	-99	-99	-99	-99	-99	-99	-99	231	-99
4,44212503	0,28072900	0,17607354	0,18546900	0,55912781	0,10498463	7,08736819	2,41036228	0,00010541	0,05897511	0,06017258	0,37794547	0,01616761	1,05354359	2,31698708	1,45120366	0,17461507	0,01201724	0,05752170	0,68077502	0,66861610	232	0,12295906
3,32245578	0,15117065	0,21260538	0,23459595	0,30933672	0,06050717	8,54600405	0,72913103	0,09870384	0,03106104	0,04911304	0,15499032	0,00081171	0,42580063	1,13386588	0,17425235	0,10454892	0,02870110	0,11028053	0,29387000	0,68979998	233	0,14411076
2,62396777	0,06748684	0,16942868	0,59780630	0,16196455	0,28199666	28,12439194	1,58639276	0,06188570	0,31770286	0,02468451	0,06609798	0,00669709	0,04533440	0,59739207	0,87039972	0,19260940	0,01744249	0,04424781	0,84894912	0,19301033	234	0,03865369
1,52864139	0,13492691	0,19809303	0,46951723	0,24405023	0,31213672	17,34262046	1,46028504	0,07506322	0,30444426	0,02066381	0,06807411	0,00513739	0,85633666	1,17222540	0,37533481	0,10578815	0,00798357	0,06974579	0,33991596	0,46337432	235	0,10944288
-99	-99	-99	-99	-99	-99	-99	-99	-99	-99	-99	-99	-99	-99	-99	-99	-99	-99	-99	-99	-99	236	-99

-99	-99	-99	-99	-99	-99	-99	-99	-99	-99	-99	-99	-99	-99	-99	-99	-99	-99	-99	-99	-99	237	-99
-99	-99	-99	-99	-99	-99	-99	-99	-99	-99	-99	-99	-99	-99	-99	-99	-99	-99	-99	-99	-99	238	-99
-99	-99	-99	-99	-99	-99	-99	-99	-99	-99	-99	-99	-99	-99	-99	-99	-99	-99	-99	-99	-99	239	-99
-99	-99	-99	-99	-99	-99	-99	-99	-99	-99	-99	-99	-99	-99	-99	-99	-99	-99	-99	-99	-99	240	-99
-99	-99	-99	-99	-99	-99	-99	-99	-99	-99	-99	-99	-99	-99	-99	-99	-99	-99	-99	-99	-99	241	-99
-99	-99	-99	-99	-99	-99	-99	-99	-99	-99	-99	-99	-99	-99	-99	-99	-99	-99	-99	-99	-99	242	-99
0,84264683	0,12985739	0,18049115	0,27452320	0,05288491	0,18402836	16,80711270	1,86218964	0,00389274	0,01019496	0,01984611	0,23848989	0,00072286	0,49827013	1,58776786	0,27566729	0,21419549	0,02158241	0,02662638	0,08808255	1,02882671	243	0,08900314
1,76051803	0,16898885	0,21330652	0,37319460	0,19874631	0,02881572	6,16884330	0,78676245	0,02013710	0,04512279	0,02582657	0,04230582	0,00506227	0,20949678	1,21587928	0,21508816	0,24948068	0,01865880	0,04668167	0,72698626	0,38850363	244	0,10533085
-99	-99	-99	-99	-99	-99	-99	-99	-99	-99	-99	-99	-99	-99	-99	-99	-99	-99	-99	-99	-99	245	-99
4,19014451	0,27643266	0,14823931	0,29098163	0,28068036	0,05003218	4,11245531	0,97942030	0,06083309	0,01450808	0,01887997	0,17800627	0,00174084	0,07694653	1,40152745	0,40668960	0,16934062	0,00736550	0,13194421	0,45786684	0,64662447	246	0,16448182
-99	-99	-99	-99	-99	-99	-99	-99	-99	-99	-99	-99	-99	-99	-99	-99	-99	-99	-99	-99	-99	247	-99
4,09609734	0,18976029	0,29694137	0,39646076	0,34513914	0,10425945	10,58720598	1,47554713	0,00005712	0,33039185	0,03745365	0,13051160	0,00189610	0,01446039	0,75589094	0,53970763	0,12398607	0,02730388	0,00655699	0,35214707	0,51772217	248	0,05824387
0,63573614	0,25498778	0,23076559	0,50052013	0,15172177	0,14195445	16,59296730	3,84904356	0,08805203	0,01240014	0,02372415	0,21937941	0,01105237	0,55420821	0,86244239	0,59563482	0,09217377	0,01288200	0,00005217	0,33529433	0,14757293	249	0,04841171
-99	-99	-99	-99	-99	-99	-99	-99	-99	-99	-99	-99	-99	-99	-99	-99	-99	-99	-99	-99	-99	250	-99
2,45717396	0,36882286	0,30249852	0,35038186	0,28165481	0,05204849	0,72648252	1,90527600	0,06176785	0,02251453	0,01692149	0,04061060	0,00875304	0,58641747	0,70759708	0,49209201	0,38103621	0,01826214	0,01500929	0,51655719	0,24485507	251	0,10965169
-99	-99	-99	-99	-99	-99	-99	-99	-99	-99	-99	-99	-99	-99	-99	-99	-99	-99	-99	-99	-99	252	-99
-99	-99	-99	-99	-99	-99	-99	-99	-99	-99	-99	-99	-99	-99	-99	-99	-99	-99	-99	-99	-99	253	-99
-99	-99	-99	-99	-99	-99	-99	-99	-99	-99	-99	-99	-99	-99	-99	-99	-99	-99	-99	-99	-99	254	-99
-99	-99	-99	-99	-99	-99	-99	-99	-99	-99	-99	-99	-99	-99	-99	-99	-99	-99	-99	-99	-99	255	-99
3,05834254	0,11397189	0,22109679	0,23128602	0,15504404	0,03241376	0,62970586	1,57652713	0,07263204	0,34382587	0,02569711	0,11178124	0,00927620	0,64293732	1,33306765	0,90422267	0,13404135	0,01452568	0,03194536	0,41979300	0,33815346	256	0,14852215
-99	-99	-99	-99	-99	-99	-99	-99	-99	-99	-99	-99	-99	-99	-99	-99	-99	-99	-99	-99	-99	257	-99
-99	-99	-99	-99	-99	-99	-99	-99	-99	-99	-99	-99	-99	-99	-99	-99	-99	-99	-99	-99	-99	258	-99
2,13243239	0,23956682	0,22476667	0,48146406	0,12145552	0,07373534	33,88324164	2,57665611	0,15352016	0,38809991	0,04303040	0,25729583	0,01310717	1,67888103	1,11844941	1,52361669	0,20654099	0,02658028	0,05124304	0,15849399	0,44704749	259	0,02111629
-99	-99	-99	-99	-99	-99	-99	-99	-99	-99	-99	-99	-99	-99	-99	-99	-99	-99	-99	-99	-99	260	-99
0,80051481	0,19371398	0,16981079	0,28797191	0,24299525	0,19778433	28,46268779	2,22221746	0,06666161	0,02293985	0,01776277	0,08573333	0,00187226	0,51619927	1,16958766	1,05995278	0,12217322	0,00632377	0,08753477	0,12833619	0,48363816	261	0,11478452
2,78224603	0,10719058	0,21512543	0,29776581	0,05796195	0,03693801	9,33888298	2,18744994	0,10084769	0,03511546	0,03021168	0,12026177	0,01127483	0,60342652	1,08843104	0,48204843	0,06264095	0,00876670	0,06264095	0,26265665	0,84337724	262	0,09761523
-99	-99	-99	-99	-99	-99	-99	-99	-99	-99	-99	-99	-99	-99	-99	-99	-99	-99	-99	-99	-99	263	-99
3,72308873	0,19418451	0,26288432	0,19840221	0,32748483	0,10384474	29,15153608	2,34323232	0,11238333	0,04576854	0,03017505	0,16579817	0,00051273	0,44119856	1,40395822	0,94245785	0,37023161	0,02034051	0,02674661	0,33552682	0,79526082	264	0,08629974
4,63558045	0,28732392	0,12463231	0,31289489	0,22542978	0,11162639	10,42337081	0,86318997	0,15143284	0,00994199	0,00883074	0,02177400	0,01065556	0,04268136	0,64204664	2,19428360	0,06692783	0,02804233	0,07524555	0,02686273	0,69821826	265	0,01740626

MPI_FGH_Ds [61711]																					Location_ Treatment	
4	50	32	31	28	19	26	5	36	20	29	14	30	9	13	2	18	16	10	7	34	Ranking	11
**Receptor_ protein_ kinase**	**Reticuline_ oxidase**	**Serine-threonine_ protein_kinase _plant-type**	**TMV_ resistance_ protein_N**	**Homoserine_ dehydro genase_family _protein**	**Cc-nbs-lrr resistance protein**	**UDP-glucose: glucosyltrans ferase**	**Methionyl-tRNA_ synthetase**	**70_kDa_ subunit_of_ replication_ protein_A**	**Gene_of_ unknown_ function**	**RING_finger_ protein**	**Flagellin-sensing 2**	**Serine-threonine protein kinase, plant-type**	**TIR-NBS-LRR_disease_ resistance**	**LRR receptor-like serine/ threonine-protein kinase**	**Poly(ADP-ribose)_glyco hydrolase**	**Bacterial_spot _disease_ resistance_ protein_4**	**Extensin**	**Lipoxygenase**	**Betaine_ aldehyde_ dehyrogenase**	**Fatty_acid_ desaturase**	**Gene product**	**Glutathione-S-transferase**
PGSC0003 DMT40004568	PGSC0003 DMT40004630	PGSC0003 DMT40004644	PGSC0003 DMT40004689	PGSC0003 DMT40004811	PGSC0003 DMT40004909	PGSC0003 DMT40004912	PGSC0003 DMT40004917	PGSC0003 DMT40005251	PGSC0003 DMT40006237	PGSC0003 DMT40006687	PGSC0003 DMT40006877	PGSC0003 DMT40006878	PGSC0003 DMT40007144	PGSC0003 DMT40007188	PGSC0003 DMT40007551	PGSC0003 DMT40008081	PGSC0003 DMT40008201	PGSC0003 DMT40008202	PGSC0003 DMT40008302	PGSC0003 DMT40008385	**Transcript ID F1-Individuals**	PGSC0003 DMT400004539
0,02050330	4,66539748	0,10806715	0,04010706	0,20250087	0,52340514	1,80125196	0,04152143	0,72648252	0,04543664	0,97063447	0,05605551	0,02653426	0,72146434	0,16210494	0,55286533	0,28204553	4,77003549	9,82869584	0,48903174	0,07850890	1	12,81933927
-99	-99	-99	-99	-99	-99	-99	-99	-99	-99	-99	-99	-99	-99	-99	-99	-99	-99	-99	-99	-99	2	-99
-99	-99	-99	-99	-99	-99	-99	-99	-99	-99	-99	-99	-99	-99	-99	-99	-99	-99	-99	-99	-99	3	-99
-99	-99	-99	-99	-99	-99	-99	-99	-99	-99	-99	-99	-99	-99	-99	-99	-99	-99	-99	-99	-99	4	-99
-99	-99	-99	-99	-99	-99	-99	-99	-99	-99	-99	-99	-99	-99	-99	-99	-99	-99	-99	-99	-99	5	-99
0,06940820	5,31935013	0,06530105	0,04924940	0,04362352	0,11552284	0,56262673	0,03856003	0,37873220	0,03171370	2,93070956	0,03802916	0,02745571	0,36659271	0,16235796	0,68124706	0,05260158	4,14464920	5,50312081	0,30150420	0,16738591	6	9,56490076
-99	-99	-99	-99	-99	-99	-99	-99	-99	-99	-99	-99	-99	-99	-99	-99	-99	-99	-99	-99	-99	7	-99
-99	-99	-99	-99	-99	-99	-99	-99	-99	-99	-99	-99	-99	-99	-99	-99	-99	-99	-99	-99	-99	8	-99
-99	-99	-99	-99	-99	-99	-99	-99	-99	-99	-99	-99	-99	-99	-99	-99	-99	-99	-99	-99	-99	9	-99
-99	-99	-99	-99	-99	-99	-99	-99	-99	-99	-99	-99	-99	-99	-99	-99	-99	-99	-99	-99	-99	10	-99
-99	-99	-99	-99	-99	-99	-99	-99	-99	-99	-99	-99	-99	-99	-99	-99	-99	-99	-99	-99	-99	11	-99
-99	-99	-99	-99	-99	-99	-99	-99	-99	-99	-99	-99	-99	-99	-99	-99	-99	-99	-99	-99	-99	12	-99
-99	-99	-99	-99	-99	-99	-99	-99	-99	-99	-99	-99	-99	-99	-99	-99	-99	-99	-99	-99	-99	13	-99
-99	-99	-99	-99	-99	-99	-99	-99	-99	-99	-99	-99	-99	-99	-99	-99	-99	-99	-99	-99	-99	14	-99
-99	-99	-99	-99	-99	-99	-99	-99	-99	-99	-99	-99	-99	-99	-99	-99	-99	-99	-99	-99	-99	15	-99
0,17918230	1,45750416	0,01095323	0,06513153	0,01307994	0,35050332	0,52358657	0,05545649	0,01209454	0,00003735	1,38462951	0,02059589	0,01055083	0,57256048	0,12221557	0,33739254	0,11895600	12,85715913	9,80488018	0,18953025	0,09824311	16	15,27923069
0,28068036	2,83431479	0,07320064	0,08626983	0,13332322	0,15261831	0,96727633	0,14269434	0,00530291	0,04822751	1,31312113	0,04565764	0,03775993	0,44503787	0,37397144	0,49380043	0,03195089	8,43857854	6,92589252	0,39584293	0,15432033	17	10,26384938
0,03654961	3,17333537	0,18646800	0,06519929	0,07453180	0,36123260	0,92402257	0,11226655	0,00747868	0,00860863	2,15995312	0,11550282	0,07894545	0,17800627	0,47731051	0,51227841	0,05872017	8,04448464	8,47374668	0,34796160	0,34651747	18	17,56644072
0,14597063	6,30610853	0,03908476	0,05710457	0,04772040	0,29295480	0,98196942	0,02592971	0,19775005	0,04542877	1,27787790	0,03388405	0,02354803	0,24312160	0,19354622	0,29560652	0,07524555	11,80028665	7,52009037	0,76458563	0,06406810	19	6,82580878
-99	-99	-99	-99	-99	-99	-99	-99	-99	-99	-99	-99	-99	-99	-99	-99	-99	-99	-99	-99	-99	20	-99
-99	-99	-99	-99	-99	-99	-99	-99	-99	-99	-99	-99	-99	-99	-99	-99	-99	-99	-99	-99	-99	21	-99
-99	-99	-99	-99	-99	-99	-99	-99	-99	-99	-99	-99	-99	-99	-99	-99	-99	-99	-99	-99	-99	22	-99
-99	-99	-99	-99	-99	-99	-99	-99	-99	-99	-99	-99	-99	-99	-99	-99	-99	-99	-99	-99	-99	23	-99
0,02740817	2,25753757	0,08661436	0,11644737	0,08274122	0,10243282	1,27610762	0,08691506	0,13348504	0,02925344	0,92706988	0,02456503	0,07400415	0,36267520	0,20902538	0,42388652	0,15122305	8,13278796	8,56082598	0,18425172	0,14426068	24	7,35895035
-99	-99	-99	-99	-99	-99	-99	-99	-99	-99	-99	-99	-99	-99	-99	-99	-99	-99	-99	-99	-99	25	-99
-99	-99	-99	-99	-99	-99	-99	-99	-99	-99	-99	-99	-99	-99	-99	-99	-99	-99	-99	-99	-99	26	-99
-99	-99	-99	-99	-99	-99	-99	-99	-99	-99	-99	-99	-99	-99	-99	-99	-99	-99	-99	-99	-99	27	-99
-99	-99	-99	-99	-99	-99	-99	-99	-99	-99	-99	-99	-99	-99	-99	-99	-99	-99	-99	-99	-99	28	-99
-99	-99	-99	-99	-99	-99	-99	-99	-99	-99	-99	-99	-99	-99	-99	-99	-99	-99	-99	-99	-99	29	-99
-99	-99	-99	-99	-99	-99	-99	-99	-99	-99	-99	-99	-99	-99	-99	-99	-99	-99	-99	-99	-99	30	-99
-99	-99	-99	-99	-99	-99	-99	-99	-99	-99	-99	-99	-99	-99	-99	-99	-99	-99	-99	-99	-99	31	-99
-99	-99	-99	-99	-99	-99	-99	-99	-99	-99	-99	-99	-99	-99	-99	-99	-99	-99	-99	-99	-99	32	-99
0,04864718	4,79489758	0,00840521	0,02129266	0,09179122	0,49534308	2,17874900	0,04513843	0,53052771	0,02169490	1,11612608	0,05821360	0,02556830	0,25658345	0,13759500	0,48280081	0,08272688	10,41434355	6,18811497	0,27347863	0,17331879	33	12,26520974
-99	-99	-99	-99	-99	-99	-99	-99	-99	-99	-99	-99	-99	-99	-99	-99	-99	-99	-99	-99	-99	34	-99
0,08837303	2,16707638	0,03203405	0,09785233	0,09354148	0,25698392	1,21146271	0,22794379	0,26771131	0,00002773	2,68281951	0,04477229	0,02434467	0,47261915	0,14708785	0,32169156	0,24228047	5,22615531	8,60842914	0,24583292	0,06069620	35	8,92900860
-99	-99	-99	-99	-99	-99	-99	-99	-99	-99	-99	-99	-99	-99	-99	-99	-99	-99	-99	-99	-99	36	-99
-99	-99	-99	-99	-99	-99	-99	-99	-99	-99	-99	-99	-99	-99	-99	-99	-99	-99	-99	-99	-99	37	-99
-99	-99	-99	-99	-99	-99	-99	-99	-99	-99	-99	-99	-99	-99	-99	-99	-99	-99	-99	-99	-99	38	-99
-99	-99	-99	-99	-99	-99	-99	-99	-99	-99	-99	-99	-99	-99	-99	-99	-99	-99	-99	-99	-99	39	-99
0,06865459	1,38462951	0,09300809	0,09831123	0,05149228	0,31808847	1,73688231	0,05177861	0,13309239	0,01622655	1,60158459	0,04099239	0,04402601	0,21205347	0,15330748	0,51637820	0,16729892	11,86384706	3,66674516	0,40178622	0,13664457	40	0,74846149
-99	-99	-99	-99	-99	-99	-99	-99	-99	-99	-99	-99	-99	-99	-99	-99	-99	-99	-99	-99	-99	41	-99
-99	-99	-99	-99	-99	-99	-99	-99	-99	-99	-99	-99	-99	-99	-99	-99	-99	-99	-99	-99	-99	42	-99
0,18203023	2,88235893	0,02830597	0,08864911	0,10310956	0,13446010	1,32615555	0,11860609	0,12143448	0,00566484	0,92226292	0,02280114	0,02108338	0,15715390	0,16045604	0,79017823	0,17078462	4,80404612	4,59877623	0,40116009	0,15499032	43	11,28433894
-99	-99	-99	-99	-99	-99	-99	-99	-99	-99	-99	-99	-99	-99	-99	-99	-99	-99	-99	-99	-99	44	-99
-99	-99	-99	-99	-99	-99	-99	-99	-99	-99	-99	-99	-99	-99	-99	-99	-99	-99	-99	-99	-99	45	-99
0,07059697	1,97485948	0,03576643	0,06065414	0,06660388	0,17084382	1,94632145	0,05439998	0,38709243	0,00296809	2,68096057	0,10986091	0,06353741	0,41602727	0,36167104	0,53468088	0,23500282	9,85427682	14,36762803	0,44992299	0,16445332	46	32,63843552
-99	-99	-99	-99	-99	-99	-99	-99	-99	-99	-99	-99	-99	-99	-99	-99	-99	-99	-99	-99	-99	47	-99
-99	-99	-99	-99	-99	-99	-99	-99	-99	-99	-99	-99	-99	-99	-99	-99	-99	-99	-99	-99	-99	48	-99
0,11949316	7,22501208	0,09466664	0,04425548	0,04587971	0,18339167	1,12896440	0,07189324	0,38395257	0,18351883	1,80750545	0,05340061	0,03206182	0,86693756	0,13443680	0,68396765	0,03280365	6,43972910	21,42168411	0,37866658	0,14711334	49	19,93525287
0,10927233	4,31990624	0,05456047	0,05509251	0,10323471	0,26115894	1,25006230	0,04210105	0,19157750	0,01703918	0,95065910	0,03133676	0,03275820	0,13021793	0,11250024	0,37892914	0,17016426	4,58207159	3,78685452	0,56722774	0,10836720	50	24,74388280
0,04631907	1,91421118	0,09341189	0,02478739	0,06358146	0,18463526	0,80259830	0,04111333	0,69676787	0,00366683	2,46356921	0,04274057	0,02023154	0,65010741	0,16433936	0,73496630	0,09432278	7,59868620	9,49883116	0,34910913	0,09977001	51	11,16181705
-99	-99	-99	-99	-99	-99	-99	-99	-99	-99	-99	-99	-99	-99	-99	-99	-99	-99	-99	-99	-99	52	-99
-99	-99	-99	-99	-99	-99	-99	-99	-99	-99	-99	-99	-99	-99	-99	-99	-99	-99	-99	-99	-99	53	-99
-99	-99	-99	-99	-99	-99	-99	-99	-99	-99	-99	-99	-99	-99	-99	-99	-99	-99	-99	-99	-99	54	-99
-99	-99	-99	-99	-99	-99	-99	-99	-99	-99	-99	-99	-99	-99	-99	-99	-99	-99	-99	-99	-99	55	-99
-99	-99	-99	-99	-99	-99	-99	-99	-99	-99	-99	-99	-99	-99	-99	-99	-99	-99	-99	-99	-99	56	-99
0,02453100	2,98297110	0,08465564	0,08530357	0,04798575	0,16989909	1,06566205	0,06192862	0,05037145	0,03353357	1,10095080	0,03254884	0,04174508	0,16802526	0,21805289	0,42183479	0,04858821	8,53712321	5,38053638	0,47393135	0,06116075	57	9,52190345
0,17343897	2,32382270	0,15140660	0,07348023	0,07236822	0,09242968	1,87189563	0,11426853	0,43663510	0,02029826	1,06769531	0,07348023	0,03546402	0,12678887	0,24510979	0,66044027	0,08552559	7,44101795	8,65479711	0,35269670	0,23382482	58	17,96043018
-99	-99	-99	-99	-99	-99	-99	-99	-99	-99	-99	-99	-99	-99	-99	-99	-99	-99	-99	-99	-99	59	-99
-99	-99	-99	-99	-99	-99	-99	-99	-99	-99	-99	-99	-99	-99	-99	-99	-99	-99	-99	-99	-99	60	-99
0,03483062	3,12856266	0,07691986	0,04056839	0,10065565	0,57854457	1,12700975	0,21590971	0,97840250	0,01368273	2,28865500	0,01967490	0,05595846	0,22398903	0,11482431	0,41451609	0,31154231	13,81822114	4,05162317	0,38302222	0,03263356	61	1,70941182
-99	-99	-99	-99	-99	-99	-99	-99	-99	-99	-99	-99	-99	-99	-99	-99	-99	-99	-99	-99	-99	62	-99
-99	-99	-99	-99	-99	-99	-99	-99	-99	-99	-99	-99	-99	-99	-99	-99	-99	-99	-99	-99	-99	63	-99
-99	-99	-99	-99	-99	-99	-99	-99	-99	-99	-99	-99	-99	-99	-99	-99	-99	-99	-99	-99	-99	64	-99
0,13169295	3,67883762	0,02169114	0,09409423	0,03441664	0,14361218	1,67626472	0,12244875	0,74600127	0,00901317	1,00992629	0,06984255	0,03285485	0,67513597	0,13690528	0,56301685	0,09299197	6,49464199	4,57017687	0,34753977	0,09587178	65	32,50297795
-99	-99	-99	-99	-99	-99	-99	-99	-99	-99	-99	-99	-99	-99	-99	-99	-99	-99	-99	-99	-99	66	-99
-99	-99	-99	-99	-99	-99	-99	-99	-99	-99	-99	-99	-99	-99	-99	-99	-99	-99	-99	-99	-99	67	-99
-99	-99	-99	-99	-99	-99	-99	-99	-99	-99	-99	-99	-99	-99	-99	-99	-99	-99	-99	-99	-99	68	-99
-99	-99	-99	-99	-99	-99	-99	-99	-99	-99	-99	-99	-99	-99	-99	-99	-99	-99	-99	-99	-99	69	-99
-99	-99	-99	-99	-99	-99	-99	-99	-99	-99	-99	-99	-99	-99	-99	-99	-99	-99	-99	-99	-99	70	-99

-99	-99	-99	-99	-99	-99	-99	-99	-99	-99	-99	-99	-99	-99	-99	-99	-99	-99	-99	-99	-99	71	-99
-99	-99	-99	-99	-99	-99	-99	-99	-99	-99	-99	-99	-99	-99	-99	-99	-99	-99	-99	-99	-99	72	-99
-99	-99	-99	-99	-99	-99	-99	-99	-99	-99	-99	-99	-99	-99	-99	-99	-99	-99	-99	-99	-99	73	-99
-99	-99	-99	-99	-99	-99	-99	-99	-99	-99	-99	-99	-99	-99	-99	-99	-99	-99	-99	-99	-99	74	-99
0,03976106	2,91097006	0,15394640	0,07713343	0,07221789	0,09865254	2,25558241	0,09682343	0,09736182	0,03512154	2,35462939	0,04637529	0,05446601	0,17893407	0,39270014	0,74613056	0,05029295	5,69028140	7,48239433	0,40514212	0,13598317	75	6,93790459
-99	-99	-99	-99	-99	-99	-99	-99	-99	-99	-99	-99	-99	-99	-99	-99	-99	-99	-99	-99	-99	76	-99
-99	-99	-99	-99	-99	-99	-99	-99	-99	-99	-99	-99	-99	-99	-99	-99	-99	-99	-99	-99	-99	77	-99
-99	-99	-99	-99	-99	-99	-99	-99	-99	-99	-99	-99	-99	-99	-99	-99	-99	-99	-99	-99	-99	78	-99
-99	-99	-99	-99	-99	-99	-99	-99	-99	-99	-99	-99	-99	-99	-99	-99	-99	-99	-99	-99	-99	79	-99
-99	-99	-99	-99	-99	-99	-99	-99	-99	-99	-99	-99	-99	-99	-99	-99	-99	-99	-99	-99	-99	80	-99
-99	-99	-99	-99	-99	-99	-99	-99	-99	-99	-99	-99	-99	-99	-99	-99	-99	-99	-99	-99	-99	81	-99
-99	-99	-99	-99	-99	-99	-99	-99	-99	-99	-99	-99	-99	-99	-99	-99	-99	-99	-99	-99	-99	82	-99
-99	-99	-99	-99	-99	-99	-99	-99	-99	-99	-99	-99	-99	-99	-99	-99	-99	-99	-99	-99	-99	83	-99
0,18669433	2,83972259	0,12088859	0,10137333	0,00985280	0,00003291	1,10630563	0,17455457	0,22002662	0,02414302	3,53020020	0,04137061	0,04111333	0,47065767	0,09717641	0,39440507	0,21987416	6,87686012	10,49587108	0,33885736	0,17576869	84	1,14353459
-99	-99	-99	-99	-99	-99	-99	-99	-99	-99	-99	-99	-99	-99	-99	-99	-99	-99	-99	-99	-99	85	-99
-99	-99	-99	-99	-99	-99	-99	-99	-99	-99	-99	-99	-99	-99	-99	-99	-99	-99	-99	-99	-99	86	-99
0,09829419	2,33958071	0,10246833	0,07126068	0,11673022	0,83003841	1,62478632	0,13987887	0,51987980	0,00036401	1,52970133	0,02390158	0,01473356	0,21084431	0,12853650	0,44388258	0,22897310	15,96953057	12,59038068	0,51307797	0,11640702	87	15,85922097
0,01823685	5,29635564	0,10972772	0,03356264	0,10489370	0,69303494	0,68112902	0,20068435	0,07386322	0,03749911	2,63353384	0,06629298	0,03379609	0,20920656	0,10416915	0,55363229	0,02653426	11,21221959	5,89707687	0,76207242	0,10323471	88	9,95554296
0,13263193	3,11557835	0,07181853	0,08623994	0,03074509	0,00256380	1,47580284	0,09729436	0,08653934	0,03055389	2,45122004	0,05845621	0,02425624	0,20412149	0,18575848	0,29719876	0,13636072	4,49867455	3,43307173	0,19431915	0,07670689	89	24,81688267
-99	-99	-99	-99	-99	-99	-99	-99	-99	-99	-99	-99	-99	-99	-99	-99	-99	-99	-99	-99	-99	90	-99
-99	-99	-99	-99	-99	-99	-99	-99	-99	-99	-99	-99	-99	-99	-99	-99	-99	-99	-99	-99	-99	91	-99
-99	-99	-99	-99	-99	-99	-99	-99	-99	-99	-99	-99	-99	-99	-99	-99	-99	-99	-99	-99	-99	92	-99
-99	-99	-99	-99	-99	-99	-99	-99	-99	-99	-99	-99	-99	-99	-99	-99	-99	-99	-99	-99	-99	93	-99
-99	-99	-99	-99	-99	-99	-99	-99	-99	-99	-99	-99	-99	-99	-99	-99	-99	-99	-99	-99	-99	94	-99
-99	-99	-99	-99	-99	-99	-99	-99	-99	-99	-99	-99	-99	-99	-99	-99	-99	-99	-99	-99	-99	95	-99
-99	-99	-99	-99	-99	-99	-99	-99	-99	-99	-99	-99	-99	-99	-99	-99	-99	-99	-99	-99	-99	96	-99
-99	-99	-99	-99	-99	-99	-99	-99	-99	-99	-99	-99	-99	-99	-99	-99	-99	-99	-99	-99	-99	97	-99
-99	-99	-99	-99	-99	-99	-99	-99	-99	-99	-99	-99	-99	-99	-99	-99	-99	-99	-99	-99	-99	98	-99
0,07890442	3,85638743	0,05983983	0,10454892	0,07792608	0,16901814	1,38343034	0,09114138	0,64093502	0,00634682	2,06372394	0,03169173	0,02737969	0,63474543	0,19055112	0,49457115	0,12441652	2,87836590	2,74774857	0,38561952	0,14075421	99	4,95969017
0,10072544	2,33512536	0,03566122	0,10698645	0,08434814	0,14185609	2,00763917	0,13532497	0,56350488	0,05217491	1,28209219	0,05275677	0,05004952	0,23725329	0,23512502	0,53273870	0,12740555	12,52292787	18,61637149	0,22996720	0,18030359	100	7,77045996
-99	-99	-99	-99	-99	-99	-99	-99	-99	-99	-99	-99	-99	-99	-99	-99	-99	-99	-99	-99	-99	101	-99
-99	-99	-99	-99	-99	-99	-99	-99	-99	-99	-99	-99	-99	-99	-99	-99	-99	-99	-99	-99	-99	102	-99
0,18614516	3,24563328	0,09892644	0,06432396	0,21486464	0,17176405	1,80938573	0,13336944	0,00001903	0,00658546	1,25483702	0,05646497	0,04323969	0,88239713	0,15598725	0,49499985	0,22398903	15,43892075	8,73313717	0,40067378	0,11091297	103	12,77056102
0,02533016	2,32946717	0,06381326	0,12345011	0,01534593	0,14478660	2,65185152	0,13956412	0,00365985	0,03351614	1,43395525	0,03966472	0,05045007	0,19250929	0,09971815	0,43982454	0,06670783	4,74365800	4,04180579	0,37552998	0,09265419	104	8,67581927
-99	-99	-99	-99	-99	-99	-99	-99	-99	-99	-99	-99	-99	-99	-99	-99	-99	-99	-99	-99	-99	105	-99
0,06069620	3,94151428	0,16640261	0,04706342	0,04788608	0,35693914	1,89178744	0,11389292	0,02456503	0,04250422	1,18078803	0,06623557	0,05054632	0,64651242	0,09583856	0,56282176	0,26329463	0,18890725	3,24169869	0,34959344	0,10067309	106	7,10458313
0,06307666	3,49005555	0,01081554	0,08822003	0,05052881	0,06699745	1,90824975	0,06242423	0,54800084	0,02739868	1,07941527	0,04857137	0,02942630	0,20823002	0,09911519	0,60510189	0,07972909	4,52290596	2,20878041	0,29123386	0,06598354	107	6,38970798
-99	-99	-99	-99	-99	-99	-99	-99	-99	-99	-99	-99	-99	-99	-99	-99	-99	-99	-99	-99	-99	108	-99
-99	-99	-99	-99	-99	-99	-99	-99	-99	-99	-99	-99	-99	-99	-99	-99	-99	-99	-99	-99	-99	109	-99
0,13872022	3,83506226	0,08084207	0,04484994	0,06013088	1,03472687	2,79190529	0,10951877	0,19227592	0,02540489	2,52144368	0,02431938	0,03688684	1,00573485	0,07454472	0,51915959	0,04287409	2,84070693	3,95382769	0,69846029	0,17042986	110	17,13352390
-99	-99	-99	-99	-99	-99	-99	-99	-99	-99	-99	-99	-99	-99	-99	-99	-99	-99	-99	-99	-99	111	-99
-99	-99	-99	-99	-99	-99	-99	-99	-99	-99	-99	-99	-99	-99	-99	-99	-99	-99	-99	-99	-99	112	-99
-99	-99	-99	-99	-99	-99	-99	-99	-99	-99	-99	-99	-99	-99	-99	-99	-99	-99	-99	-99	-99	113	-99
0,04464059	3,97650229	0,11961747	0,08244067	0,08353358	0,46057244	2,21529680	0,17093266	0,26252014	0,02195206	1,46459320	0,02352763	0,02864645	1,41764863	0,32230534	0,30981953	0,04457875	8,13137878	8,68484439	0,35172018	0,13336944	114	13,37298850
-99	-99	-99	-99	-99	-99	-99	-99	-99	-99	-99	-99	-99	-99	-99	-99	-99	-99	-99	-99	-99	115	-99
-99	-99	-99	-99	-99	-99	-99	-99	-99	-99	-99	-99	-99	-99	-99	-99	-99	-99	-99	-99	-99	116	-99
-99	-99	-99	-99	-99	-99	-99	-99	-99	-99	-99	-99	-99	-99	-99	-99	-99	-99	-99	-99	-99	117	-99
-99	-99	-99	-99	-99	-99	-99	-99	-99	-99	-99	-99	-99	-99	-99	-99	-99	-99	-99	-99	-99	118	-99
-99	-99	-99	-99	-99	-99	-99	-99	-99	-99	-99	-99	-99	-99	-99	-99	-99	-99	-99	-99	-99	119	-99
0,06886907	1,42010736	0,04487327	0,10034217	0,03508504	0,15767218	1,04826248	0,18162063	0,01910375	0,00007045	3,12531152	0,04540516	0,03713697	0,17579915	0,10957572	0,46683997	0,11289081	3,66293475	1,56157299	0,40192550	0,14279328	120	14,19686029
0,05735249	2,27363395	0,00804886	0,05933388	0,06315322	0,14782888	0,81225240	0,05244685	0,38182937	0,02284465	1,84165139	0,05133192	0,02365026	0,71400199	0,15357337	0,38262419	0,11141381	12,50991429	9,00670869	0,29730178	0,10216692	121	8,06681964
0,01488239	4,17492419	0,09590501	0,07379925	0,03015937	0,34669766	0,41979300	0,07752202	0,52367731	0,02006396	0,96376279	0,19327809	0,04477229	0,69003909	0,34935120	0,47261915	0,12013680	3,54245616	3,29379325	0,47393135	0,09458466	122	16,64192019
-99	-99	-99	-99	-99	-99	-99	-99	-99	-99	-99	-99	-99	-99	-99	-99	-99	-99	-99	-99	-99	123	-99
-99	-99	-99	-99	-99	-99	-99	-99	-99	-99	-99	-99	-99	-99	-99	-99	-99	-99	-99	-99	-99	124	-99
-99	-99	-99	-99	-99	-99	-99	-99	-99	-99	-99	-99	-99	-99	-99	-99	-99	-99	-99	-99	-99	125	-99
-99	-99	-99	-99	-99	-99	-99	-99	-99	-99	-99	-99	-99	-99	-99	-99	-99	-99	-99	-99	-99	126	-99
0,08800626	3,81782242	0,08087009	0,11668977	0,08477308	0,33535244	1,96121813	0,06036056	0,43069844	0,05424936	1,25244739	0,06678880	0,04183198	0,69967168	0,20600482	0,63101666	0,09751379	5,44808798	4,51820585	0,59822081	0,12584763	127	1,94160536
-99	-99	-99	-99	-99	-99	-99	-99	-99	-99	-99	-99	-99	-99	-99	-99	-99	-99	-99	-99	-99	128	-99
-99	-99	-99	-99	-99	-99	-99	-99	-99	-99	-99	-99	-99	-99	-99	-99	-99	-99	-99	-99	-99	129	-99
-99	-99	-99	-99	-99	-99	-99	-99	-99	-99	-99	-99	-99	-99	-99	-99	-99	-99	-99	-99	-99	130	-99
-99	-99	-99	-99	-99	-99	-99	-99	-99	-99	-99	-99	-99	-99	-99	-99	-99	-99	-99	-99	-99	131	-99
-99	-99	-99	-99	-99	-99	-99	-99	-99	-99	-99	-99	-99	-99	-99	-99	-99	-99	-99	-99	-99	132	-99
-99	-99	-99	-99	-99	-99	-99	-99	-99	-99	-99	-99	-99	-99	-99	-99	-99	-99	-99	-99	-99	133	-99
-99	-99	-99	-99	-99	-99	-99	-99	-99	-99	-99	-99	-99	-99	-99	-99	-99	-99	-99	-99	-99	134	-99
-99	-99	-99	-99	-99	-99	-99	-99	-99	-99	-99	-99	-99	-99	-99	-99	-99	-99	-99	-99	-99	135	-99
-99	-99	-99	-99	-99	-99	-99	-99	-99	-99	-99	-99	-99	-99	-99	-99	-99	-99	-99	-99	-99	136	-99
-99	-99	-99	-99	-99	-99	-99	-99	-99	-99	-99	-99	-99	-99	-99	-99	-99	-99	-99	-99	-99	137	-99
0,21680952	2,79238913	0,18900548	0,12426570	0,12221557	0,43724082	1,82576363	0,24784327	0,20711443	0,03886189	1,24962914	0,06432396	0,04498226	0,17045939	0,32907766	1,30359886	0,13208147	12,65381058	17,64882216	0,47516484	0,06352640	138	7,86120243
-99	-99	-99	-99	-99	-99	-99	-99	-99	-99	-99	-99	-99	-99	-99	-99	-99	-99	-99	-99	-99	139	-99
0,11240281	3,56462423	0,13137385	0,11823672	0,07597929	0,20801363	1,28765848	0,05196738	0,08649437	0,02354803	1,79844495	0,08667441	0,05604580	0,18071022	0,31594602	0,62839778	0,17455457	6,21067468	7,25386552	0,29848907	0,09016742	140	11,37464804
0,12902746	5,39547509	0,05132302	0,07736099	0,04732512	0,18916932	1,48529552	0,08843431	0,42183479	0,02381064	0,95976293	0,02742718	0,01726208	0,62883351	0,18695332	0,33605051	0,06986676	4,80238145	3,00163801	0,45810493	0,11548281	141	15,39617426
0,00677060	3,38815529	0,11387319	0,06585789	0,09749689	0,00009102	1,39040001	0,04408709	0,88668895	0,01780591	1,12857320	0,07368425	0,05371616	0,21058871	0,13513750	0,26952668	0,14614780	5,22162916	5,59252577	0,29658139	0,07946702	142	10,98718577
0,12867021	1,31380394	0,06600641	0,08536272	0,02047135	0,21684709	0,99982673	0,04899404	0,55009397	0,01606150	1,19644162	0,05895467	0,03529848	0,41950212	0,11342024	0,48759323	0,02665870	2,78707147	6,05341810	0,64875695	0,16297810	143	7,40500136
-99	-99	-99	-99	-99	-99	-99	-99	-99	-99	-99	-99	-99	-99	-99	-99	-99	-99	-99	-99	-99	144	-99
0,13621901	3,09084220	0,19957460	0,11242229	0,10643171	0,18763489	1,97794183	0,09944206	0,10223776	0,03075575	1,53687518	0,09246172	0,06407920	0,22531262	0,48937083	0,48229909	0,20054529	5,19455441	5,29635564	0,45881994	0,13574773	145	6,06812162
-99	-99	-99	-99	-99	-99	-99	-99	-99	-99	-99	-99	-99	-99	-99	-99	-99	-99	-99	-99	-99	146	-99
0,02307141	2,19656623	0,18779753	0,14491210	0,13795312	0,15671878	0,99740407	0,16866704	0,68598551	0,01209245	2,53722243	0,09101512	0,06046525	0,17233050	0,31322038	0,88163292	0,09987379	9,87136779	16,60159557	0,32584325	0,12943054	147	10,36213946
-99	-99	-99	-99	-99	-99	-99	-99	-99	-99	-99	-99	-99	-99	-99	-99	-99	-99	-99	-99	-99	148	-99
-99	-99	-99	-99	-99	-99	-99	-99	-99	-99	-99	-99	-99	-99	-99	-99	-99	-99	-99	-99	-99	149	-99
-99	-99	-99	-99	-99	-99	-99	-99	-99	-99	-99	-99	-99	-99	-99	-99	-99	-99	-99	-99	-99	150	-99
-99	-99	-99	-99	-99	-99	-99	-99	-99	-99	-99	-99	-99	-99	-99	-99	-99	-99	-99	-99	-99	151	-99
0,01722324	1,72010901	0,03674012	0,05412729	0,01988053	0,14880553	0,68848637	0,04895161	0,23973293	0,01919002	1,29100984	0,06682353	0,03519465	0,14747067	0,23906917	0,60815037	0,12272491	3,20761280	4,52055529	0,57774309	0,11434776	152	11,43195312
-99	-99	-99	-99	-99	-99	-99	-99	-99	-99	-99	-99	-99	-99	-99	-99	-99	-99	-99	-99	-99	153	-99

-99	-99	-99	-99	-99	-99	-99	-99	-99	-99	-99	-99	-99	-99	-99	-99	-99	-99	-99	-99	-99	154	-99
-99	-99	-99	-99	-99	-99	-99	-99	-99	-99	-99	-99	-99	-99	-99	-99	-99	-99	-99	-99	-99	155	-99
-99	-99	-99	-99	-99	-99	-99	-99	-99	-99	-99	-99	-99	-99	-99	-99	-99	-99	-99	-99	-99	156	-99
-99	-99	-99	-99	-99	-99	-99	-99	-99	-99	-99	-99	-99	-99	-99	-99	-99	-99	-99	-99	-99	157	-99
-99	-99	-99	-99	-99	-99	-99	-99	-99	-99	-99	-99	-99	-99	-99	-99	-99	-99	-99	-99	-99	158	-99
0,10334210	3,89196841	0,11277350	0,08388171	0,03502430	0,00005450	2,70288476	0,11506333	0,86603666	0,01899153	2,69914036	0,04692498	0,04217407	0,15926487	0,20257106	0,42498976	0,28253471	6,17526051	10,75175303	0,40909279	0,13153330	159	6,52396933
0,25503197	4,64201119	0,20960572	0,06218671	0,08211275	0,13164731	1,72757698	0,12249120	0,04795251	0,01283965	1,02864844	0,06692783	0,06437971	0,16241424	0,27390547	0,55815975	0,22017919	8,53712321	7,48369105	0,41172416	0,16767622	160	8,89657479
0,06761560	5,26159425	0,06870219	0,06081201	0,09391504	0,00005997	2,02440789	0,10565991	0,84762615	0,02145563	2,25558241	0,03420853	0,05275677	0,21338046	0,21546120	0,53384765	0,36462869	12,55769698	10,06656798	0,63485543	0,20103241	161	4,64522990
-99	-99	-99	-99	-99	-99	-99	-99	-99	-99	-99	-99	-99	-99	-99	-99	-99	-99	-99	-99	-99	162	-99
-99	-99	-99	-99	-99	-99	-99	-99	-99	-99	-99	-99	-99	-99	-99	-99	-99	-99	-99	-99	-99	163	-99
0,02509424	3,18545620	0,03641054	0,07317527	0,01201100	0,18358244	0,57614346	0,11514311	0,28312283	0,13429709	1,23756131	0,03948641	0,04104926	0,18562977	0,13126007	0,58256866	0,07622986	3,38580761	3,95451290	0,31897163	0,08024192	164	25,29008534
-99	-99	-99	-99	-99	-99	-99	-99	-99	-99	-99	-99	-99	-99	-99	-99	-99	-99	-99	-99	-99	165	-99
-99	-99	-99	-99	-99	-99	-99	-99	-99	-99	-99	-99	-99	-99	-99	-99	-99	-99	-99	-99	-99	166	-99
0,01424155	11,61767926	0,03046402	0,09607135	0,08294219	0,31235315	0,65123494	0,11664934	0,35558096	0,01225276	1,87676758	0,02902118	0,03627828	1,07195920	0,32449092	0,64449899	0,03073977	11,93602039	5,81286740	0,82088384	0,14341323	167	10,91885749
-99	-99	-99	-99	-99	-99	-99	-99	-99	-99	-99	-99	-99	-99	-99	-99	-99	-99	-99	-99	-99	168	-99
-99	-99	-99	-99	-99	-99	-99	-99	-99	-99	-99	-99	-99	-99	-99	-99	-99	-99	-99	-99	-99	169	-99
-99	-99	-99	-99	-99	-99	-99	-99	-99	-99	-99	-99	-99	-99	-99	-99	-99	-99	-99	-99	-99	170	-99
0,07749516	3,51189570	0,09241366	0,08410002	0,10361107	0,17766729	0,93513723	0,10967070	0,09396387	0,02950800	1,25548953	0,07200545	0,05231070	0,63167308	0,16348725	0,63960362	0,07280846	13,58789434	18,33146269	0,45953607	0,14232391	171	17,68556004
0,16252685	2,35177493	0,20316869	0,06065414	0,09180713	0,26715520	1,96393884	0,15122305	0,38070621	0,04474127	0,86438743	0,05940590	0,02392645	0,62232927	0,03187900	0,16331735	0,25591737	11,30391016	6,70273548	0,42066684	0,15623071	172	15,40684977
-99	-99	-99	-99	-99	-99	-99	-99	-99	-99	-99	-99	-99	-99	-99	-99	-99	-99	-99	-99	-99	173	-99
0,15786355	1,41200970	0,17601253	0,07865869	0,10307383	0,14129183	2,07017108	0,14607184	0,23745894	0,00817679	1,21819915	0,10538563	0,07608469	0,86558656	0,25432585	0,50689189	0,07431257	10,60556812	10,14537298	0,26311219	0,25984983	174	6,37864505
0,04016966	2,63399024	0,22178751	0,13270089	0,09344427	0,00005344	1,43420376	0,21677195	0,13006007	0,07537605	2,09979720	0,03492127	0,04601506	0,78744442	0,13929834	0,76750604	0,04685186	7,82044119	5,59543386	0,30976585	0,12312964	175	10,53778686
0,05059014	1,20831784	0,23536962	0,05354887	0,07309923	0,35135468	1,33792755	0,02716702	0,00872881	0,01692149	1,35191083	0,10195469	0,06501877	0,22484458	0,20804968	0,40612620	0,06421259	10,17530405	12,12573253	0,22221067	0,17265931	176	36,65657274
0,02037579	1,67365249	0,07289684	0,08999571	0,12015762	0,32533546	1,11961289	0,10402484	0,14004865	0,00973907	1,11806185	0,05317898	0,02508554	0,94605765	0,16597064	0,39887262	0,14170868	11,81870451	13,94813183	0,30228892	0,09855002	177	14,78692332
-99	-99	-99	-99	-99	-99	-99	-99	-99	-99	-99	-99	-99	-99	-99	-99	-99	-99	-99	-99	-99	178	-99
-99	-99	-99	-99	-99	-99	-99	-99	-99	-99	-99	-99	-99	-99	-99	-99	-99	-99	-99	-99	-99	179	-99
-99	-99	-99	-99	-99	-99	-99	-99	-99	-99	-99	-99	-99	-99	-99	-99	-99	-99	-99	-99	-99	180	-99
-99	-99	-99	-99	-99	-99	-99	-99	-99	-99	-99	-99	-99	-99	-99	-99	-99	-99	-99	-99	-99	181	-99
0,14158595	3,66483946	0,17431275	0,13001500	0,06393501	0,00021368	1,41200970	0,10834842	0,00003102	0,01009476	2,38956829	0,04366890	0,04241593	0,37755272	0,24566258	0,53061965	0,15354676	13,97474449	9,86110966	0,27032184	0,10752544	182	9,64311999
0,05305932	3,22600796	0,02419328	0,05160841	0,07773726	0,10473024	1,55374527	0,13610104	0,18982606	0,04960915	2,12689675	0,03749261	0,03447633	0,47656668	0,05631839	0,46740660	0,12232151	4,60356016	5,65587408	0,28024295	0,19234257	183	22,70203939
-99	-99	-99	-99	-99	-99	-99	-99	-99	-99	-99	-99	-99	-99	-99	-99	-99	-99	-99	-99	-99	184	-99
-99	-99	-99	-99	-99	-99	-99	-99	-99	-99	-99	-99	-99	-99	-99	-99	-99	-99	-99	-99	-99	185	-99
-99	-99	-99	-99	-99	-99	-99	-99	-99	-99	-99	-99	-99	-99	-99	-99	-99	-99	-99	-99	-99	186	-99
-99	-99	-99	-99	-99	-99	-99	-99	-99	-99	-99	-99	-99	-99	-99	-99	-99	-99	-99	-99	-99	187	-99
-99	-99	-99	-99	-99	-99	-99	-99	-99	-99	-99	-99	-99	-99	-99	-99	-99	-99	-99	-99	-99	188	-99
0,12249120	3,76004519	0,01870736	0,08036716	0,00437273	0,12515172	0,40661913	0,05047630	0,82116839	0,00674835	1,16614727	0,05324353	0,03884170	0,63716989	0,03746663	0,30307568	0,18259890	15,18684203	11,71065609	0,28992467	0,11070176	189	14,67206469
-99	-99	-99	-99	-99	-99	-99	-99	-99	-99	-99	-99	-99	-99	-99	-99	-99	-99	-99	-99	-99	190	-99
-99	-99	-99	-99	-99	-99	-99	-99	-99	-99	-99	-99	-99	-99	-99	-99	-99	-99	-99	-99	-99	191	-99
-99	-99	-99	-99	-99	-99	-99	-99	-99	-99	-99	-99	-99	-99	-99	-99	-99	-99	-99	-99	-99	192	-99
-99	-99	-99	-99	-99	-99	-99	-99	-99	-99	-99	-99	-99	-99	-99	-99	-99	-99	-99	-99	-99	193	-99
-99	-99	-99	-99	-99	-99	-99	-99	-99	-99	-99	-99	-99	-99	-99	-99	-99	-99	-99	-99	-99	194	-99
-99	-99	-99	-99	-99	-99	-99	-99	-99	-99	-99	-99	-99	-99	-99	-99	-99	-99	-99	-99	-99	195	-99
-99	-99	-99	-99	-99	-99	-99	-99	-99	-99	-99	-99	-99	-99	-99	-99	-99	-99	-99	-99	-99	196	-99
-99	-99	-99	-99	-99	-99	-99	-99	-99	-99	-99	-99	-99	-99	-99	-99	-99	-99	-99	-99	-99	197	-99
0,02996142	1,22285216	0,13042117	0,06369174	0,11020412	0,00001137	1,60575301	0,14491210	0,76591171	0,00489746	2,11586855	0,02064949	0,02227007	0,78799042	0,11528287	0,46530545	0,05095085	7,28662123	3,78751079	0,30046107	0,13652622	198	8,39627838
0,14262018	3,52653168	0,15726287	0,11795022	0,03555632	0,11520298	0,72058973	0,06118195	0,51414600	0,12597855	1,21272295	0,10239733	0,05897511	0,90704749	0,31980181	1,06825051	0,13169295	11,86179139	12,48609117	0,29694137	0,27248524	199	20,48860707
-99	-99	-99	-99	-99	-99	-99	-99	-99	-99	-99	-99	-99	-99	-99	-99	-99	-99	-99	-99	-99	200	-99
-99	-99	-99	-99	-99	-99	-99	-99	-99	-99	-99	-99	-99	-99	-99	-99	-99	-99	-99	-99	-99	201	-99
0,14813660	1,88556907	0,13792922	0,09499530	0,11446671	0,19157750	2,02791896	0,10474839	0,31100291	0,00965840	1,20413738	0,05351177	0,04543664	0,19601011	0,19805870	0,49277467	0,13406458	5,74377804	5,47174122	0,59749560	0,13518435	202	20,12615955
0,01510322	2,85007523	0,02327621	0,05987095	0,04982453	0,15680028	1,24919613	0,20461730	0,48801588	0,02948245	1,26751252	0,02726133	0,03403116	0,21110022	0,11949316	0,35601255	0,15336062	7,51097398	5,46416103	0,30354872	0,04559439	203	17,43603380
-99	-99	-99	-99	-99	-99	-99	-99	-99	-99	-99	-99	-99	-99	-99	-99	-99	-99	-99	-99	-99	204	-99
-99	-99	-99	-99	-99	-99	-99	-99	-99	-99	-99	-99	-99	-99	-99	-99	-99	-99	-99	-99	-99	205	-99
-99	-99	-99	-99	-99	-99	-99	-99	-99	-99	-99	-99	-99	-99	-99	-99	-99	-99	-99	-99	-99	206	-99
-99	-99	-99	-99	-99	-99	-99	-99	-99	-99	-99	-99	-99	-99	-99	-99	-99	-99	-99	-99	-99	207	-99
-99	-99	-99	-99	-99	-99	-99	-99	-99	-99	-99	-99	-99	-99	-99	-99	-99	-99	-99	-99	-99	208	-99
0,12887104	3,26934131	0,07002433	0,07406830	0,08579278	0,21022410	2,39329793	0,12268238	0,07609788	0,02102136	1,20413738	0,01575551	0,01706282	0,39584293	0,06820399	0,63816438	0,19091469	6,74117464	3,06736532	0,45093769	0,08327343	209	6,91949460
-99	-99	-99	-99	-99	-99	-99	-99	-99	-99	-99	-99	-99	-99	-99	-99	-99	-99	-99	-99	-99	210	-99
-99	-99	-99	-99	-99	-99	-99	-99	-99	-99	-99	-99	-99	-99	-99	-99	-99	-99	-99	-99	-99	211	-99
0,07161969	2,68607577	0,07502421	0,09411053	0,10551354	0,13654988	1,68004514	0,06576665	0,56389560	0,01536189	2,07699825	0,03623430	0,03558714	0,63617695	0,11466524	0,27442808	0,10847993	5,08236504	6,46432620	0,55114354	0,07935693	212	3,48945082
-99	-99	-99	-99	-99	-99	-99	-99	-99	-99	-99	-99	-99	-99	-99	-99	-99	-99	-99	-99	-99	213	-99
0,11938968	4,09467799	0,15536678	0,10236185	0,04271096	0,00039544	1,91288481	0,27032184	0,37261302	0,02976478	1,63552068	0,09104667	0,03924765	0,46675908	0,20643364	0,51718416	0,11612496	7,57239674	11,19474685	0,33257462	0,13676301	214	24,95055496
-99	-99	-99	-99	-99	-99	-99	-99	-99	-99	-99	-99	-99	-99	-99	-99	-99	-99	-99	-99	-99	215	-99
0,03289472	3,44618464	0,05711447	0,12175053	0,03239691	0,00002355	1,20915568	0,13406458	1,11187916	0,02084724	1,46003201	0,04671404	0,04251159	0,19971298	0,08662937	0,41928409	0,18136903	8,66980773	7,77045996	0,29545289	0,14299137	216	22,08507765
-99	-99	-99	-99	-99	-99	-99	-99	-99	-99	-99	-99	-99	-99	-99	-99	-99	-99	-99	-99	-99	217	-99
-99	-99	-99	-99	-99	-99	-99	-99	-99	-99	-99	-99	-99	-99	-99	-99	-99	-99	-99	-99	-99	218	-99
-99	-99	-99	-99	-99	-99	-99	-99	-99	-99	-99	-99	-99	-99	-99	-99	-99	-99	-99	-99	-99	219	-99
0,05157265	3,83373336	0,08571848	0,12223675	0,09731122	0,18527627	1,69085155	0,11988724	0,89177397	0,01471825	1,98446484	0,05455102	0,02704958	0,63628720	0,16617209	0,35080714	0,14586948	14,91559888	9,88163659	0,37055254	0,06107603	220	5,34151875
0,13966089	3,98339901	0,12456753	0,03552553	0,08711108	0,43286828	2,01950260	0,03866039	0,08987103	0,00005136	1,08748839	0,07340387	0,06934809	0,81563749	0,32443469	0,58479383	0,30971217	16,57572422	10,15416709	0,47830408	0,10769327	221	7,54881400
0,02763710	4,34317475	0,18773246	0,05189538	0,10350340	0,13010515	1,63665473	0,09798808	2,54338524	0,17589056	3,68011283	0,08081405	0,03212299	0,12013680	0,05171584	0,27581064	0,27523770	4,95453616	5,37680817	0,51041761	0,27985472	222	5,36842913
0,03702773	1,07829356	0,15059544	0,04333721	0,08548114	0,27485640	1,86315797	0,11294952	0,13219596	0,04855454	2,16707638	0,09425742	0,01541790	0,29952535	0,38843632	0,85189646	0,09295975	11,92361671	8,20071670	0,31507125	0,11717608	223	6,06812162
-99	-99	-99	-99	-99	-99	-99	-99	-99	-99	-99	-99	-99	-99	-99	-99	-99	-99	-99	-99	-99	224	-99
0,02751286	1,75960304	0,14311532	0,08724704	0,03158208	0,21797733	1,67510322	0,12818061	0,17437317	0,01000767	1,46738760	0,04199176	0,04857137	0,12364279	0,24438879	0,50112763	0,05326198	2,62669738	7,46555750	0,14950338	0,24866063	225	7,48498798
0,02296770	2,53810191	0,05372547	0,06380220	0,09123619	0,13320776	1,40712452	0,16040044	0,03510329	0,01361886	2,72215648	0,05229258	0,04716139	0,83263147	0,29356462	0,66838442	0,19489244	0,19194302	0,47360296	0,36850344	0,07752202	226	3,47978941
-99	-99	-99	-99	-99	-99	-99	-99	-99	-99	-99	-99	-99	-99	-99	-99	-99	-99	-99	-99	-99	227	-99
-99	-99	-99	-99	-99	-99	-99	-99	-99	-99	-99	-99	-99	-99	-99	-99	-99	-99	-99	-99	-99	228	-99
-99	-99	-99	-99	-99	-99	-99	-99	-99	-99	-99	-99	-99	-99	-99	-99	-99	-99	-99	-99	-99	229	-99
-99	-99	-99	-99	-99	-99	-99	-99	-99	-99	-99	-99	-99	-99	-99	-99	-99	-99	-99	-99	-99	230	-99
-99	-99	-99	-99	-99	-99	-99	-99	-99	-99	-99	-99	-99	-99	-99	-99	-99	-99	-99	-99	-99	231	-99
0,06400152	1,86897854	0,07200545	0,08146081	0,16972253	0,12528191	0,64226919	0,27324178	0,03572926	0,00010541	1,08466535	0,01443035	0,01894880	0,16808350	0,24303736	0,72459663	0,29776581	11,96915998	24,22206569	0,49594430	0,09627133	232	16,74896451
0,05740221	2,24738922	0,03635380	0,23362232	0,15759023	0,13051160	0,43324350	0,21334349	0,91589238	0,00809502	3,21150602	0,05483535	0,03912542	0,91209121	0,01968513	0,49422846	0,04560229	7,37299102	5,09206205	0,48439303	0,07485539	233	-99,00000000
0,06819217	4,38552633	0,13752349	0,08412917	0,03611520	0,14099833	1,75047930	0,13534842	0,55468861	0,03857340	2,67724654	0,05398678	0,03903061	0,64293732	0,09458466	0,43279328	0,08788434	10,97196485	6,34997099	0,36901465	0,11556288	234	8,40792616
0,06658080	2,79771693	0,08945153	0,11151039	0,19795577	0,00003615	1,68558572	0,21172301	0,40032677	0,00731589	1,19685634	0,04566555	0,01705395	0,69604380	0,18114916	0,49217729	0,25904058	10,00916695	6,70505887	0,22426090	0,07961864	235	11,93477944
-99	-99	-99	-99	-99	-99	-99	-99	-99	-99	-99	-99	-99	-99	-99	-99	-99	-99	-99	-99	-99	236	-99

-99	-99	-99	-99	-99	-99	-99	-99	-99	-99	-99	-99	-99	-99	-99	-99	-99	-99	-99	-99	-99	237	-99
-99	-99	-99	-99	-99	-99	-99	-99	-99	-99	-99	-99	-99	-99	-99	-99	-99	-99	-99	-99	-99	238	-99
-99	-99	-99	-99	-99	-99	-99	-99	-99	-99	-99	-99	-99	-99	-99	-99	-99	-99	-99	-99	-99	239	-99
-99	-99	-99	-99	-99	-99	-99	-99	-99	-99	-99	-99	-99	-99	-99	-99	-99	-99	-99	-99	-99	240	-99
-99	-99	-99	-99	-99	-99	-99	-99	-99	-99	-99	-99	-99	-99	-99	-99	-99	-99	-99	-99	-99	241	-99
-99	-99	-99	-99	-99	-99	-99	-99	-99	-99	-99	-99	-99	-99	-99	-99	-99	-99	-99	-99	-99	242	-99
0,01661926	3,13398875	0,12648165	0,08567393	0,08875671	0,48229909	1,66324420	0,06219748	0,05628912	0,00291154	1,32684514	0,07668031	0,04629499	0,65975396	0,30020086	0,54601027	0,18646800	11,53543097	15,54091993	0,44782284	0,21330652	243	2,10489753
0,12015762	3,41053957	0,05787163	0,09638818	0,04450157	0,31035687	0,70417211	0,12107727	0,02174760	0,02436578	1,07103082	0,04066693	0,01306635	0,20761751	0,17134785	0,36475509	0,06003718	9,08823412	6,78806321	0,39584293	0,16585564	244	15,46837779
-99	-99	-99	-99	-99	-99	-99	-99	-99	-99	-99	-99	-99	-99	-99	-99	-99	-99	-99	-99	-99	245	-99
0,13956412	3,01466982	0,18225117	0,10791744	0,11502346	0,00005222	1,25962998	0,08579278	0,75838377	0,02960531	1,63353798	0,09565605	0,07971527	0,51548416	0,13378609	0,47041305	0,04979001	3,89601707	8,20782516	0,21286343	0,23948380	246	7,76911356
-99	-99	-99	-99	-99	-99	-99	-99	-99	-99	-99	-99	-99	-99	-99	-99	-99	-99	-99	-99	-99	247	-99
0,01978087	5,07444486	0,09114138	0,15531295	0,03753161	0,19094777	1,48169653	0,19536582	0,52640679	0,00674601	1,30789797	0,07250629	0,06503003	0,54383843	0,14652817	0,56418883	0,15802777	18,28070748	6,94151227	0,72812094	0,09133110	248	10,56997457
0,08136206	2,04698425	0,09015179	0,06352640	0,09065309	0,27883818	1,39522713	0,17561646	0,02124843	0,01193630	1,14432750	0,06396826	0,02676516	0,85826793	0,07591349	0,48146406	0,08364946	11,15408295	7,37938200	0,50575129	0,18550115	249	12,35908472
-99	-99	-99	-99	-99	-99	-99	-99	-99	-99	-99	-99	-99	-99	-99	-99	-99	-99	-99	-99	-99	250	-99
0,06867839	4,45368647	0,07850890	0,08561456	0,02308341	0,20877198	1,76051803	0,06206828	0,83335321	0,01275317	2,22530024	0,03129335	0,05486386	0,90877812	0,09585517	0,34868592	0,05664137	8,33972609	4,86014717	0,42249319	0,09284706	251	7,80690114
-99	-99	-99	-99	-99	-99	-99	-99	-99	-99	-99	-99	-99	-99	-99	-99	-99	-99	-99	-99	-99	252	-99
-99	-99	-99	-99	-99	-99	-99	-99	-99	-99	-99	-99	-99	-99	-99	-99	-99	-99	-99	-99	-99	253	-99
-99	-99	-99	-99	-99	-99	-99	-99	-99	-99	-99	-99	-99	-99	-99	-99	-99	-99	-99	-99	-99	254	-99
-99	-99	-99	-99	-99	-99	-99	-99	-99	-99	-99	-99	-99	-99	-99	-99	-99	-99	-99	-99	-99	255	-99
0,02933466	2,47898579	0,01523729	0,09901219	0,01802009	0,23811823	1,51755626	0,05100385	0,39385869	0,01512155	3,02670905	0,03757065	0,03728528	0,26752581	0,06678880	0,31485293	0,02873096	4,14321303	1,63892519	0,53061965	0,09751379	256	19,34347381
-99	-99	-99	-99	-99	-99	-99	-99	-99	-99	-99	-99	-99	-99	-99	-99	-99	-99	-99	-99	-99	257	-99
-99	-99	-99	-99	-99	-99	-99	-99	-99	-99	-99	-99	-99	-99	-99	-99	-99	-99	-99	-99	-99	258	-99
0,07241839	3,62630432	0,04454786	0,09033945	0,03811493	0,14932214	0,84469359	0,07777768	0,26508005	0,01008601	2,83529726	0,05492094	0,03292894	0,70539341	0,24613129	0,63090732	0,14818795	5,27986119	10,25673749	0,33552682	0,17271916	259	10,14537298
-99	-99	-99	-99	-99	-99	-99	-99	-99	-99	-99	-99	-99	-99	-99	-99	-99	-99	-99	-99	-99	260	-99
0,06198230	3,77636968	0,10125043	0,14670602	0,04626292	0,12824727	0,83393104	0,09558977	0,30608410	0,00980002	2,78369278	0,02324397	0,01501970	0,10323471	0,13792922	0,51799138	0,04407181	4,96570997	7,51618199	0,09106244	0,18263055	261	6,47306953
0,03737584	4,15615656	0,03768802	0,05563937	0,05820351	0,32113460	1,31608257	0,01166435	0,42521076	0,03538422	1,18447685	0,02741767	0,03732407	0,60762368	0,14281803	0,37119521	0,10652397	7,91313845	5,49549715	0,55757972	0,13343879	262	9,53346060
-99	-99	-99	-99	-99	-99	-99	-99	-99	-99	-99	-99	-99	-99	-99	-99	-99	-99	-99	-99	-99	263	-99
0,10795485	3,47858362	0,06486122	0,07363319	0,18939892	0,28233894	2,23689882	0,11075932	0,04751412	0,04542090	3,03669076	0,04915561	0,03108797	0,72522472	0,08928119	0,23140629	0,20725804	17,69782299	16,84793651	0,29740483	0,03956860	264	5,73482714
0,01570916	4,70681125	0,09035511	0,07045032	0,06244587	0,30987322	0,52150392	0,05762146	0,33932744	0,03066528	1,10477303	0,02100316	0,04333721	0,61845906	0,13752349	0,35226914	0,05936474	6,48789288	7,74223447	0,21654669	0,05093319	265	-99,00000000

JKI Shelter_Co [68015]																				Location_ Treatment		
12	27	47	38	23	25	54	45	1	66	22	35	17	57	21	6	8	3	24	33	**Ranking**	15	4
**Bacterial_spot_ disease_ resistance_ protein_4**	**Glutamyl-tRNA(Gln)_amidotransferase_ subunit_A**	**(S)-norcoclaurine synthase**	**Cytochrome_ P450**	**PR1_protein**	**Beta-D-glucan_ exohydrolase**	**SBT4B_protein**	**Ethylene-inducing_ xylanase**	**Glucosyltransferase**	**Nbs-lrr_ resistance_ protein**	**Acidic_class_II_ 1_3-beta-glucanase**	**Hcr9-OR2A**	**Serine-threonine protein kinase, plant-type**	**Gamma_amino butyrate_ transaminase_ isoform1**	**O-methyl transferase**	**Multidrug_resistance _protein_ABC_transporter_family**	**BED_finger-nbs-lrr_ resistance_ protein**	**S-locus-specific_glycoprotein_S6**	**NADPH-dependent_ codeinone_ reductase**	**Desacetoxyvindoline_4-hydroxylase**	**Gene product**	**Kinase**	**Receptor_ protein_ kinase**
PGSC0003 DMT400006231	PGSC0003 DMT40000809	PGSC0003 DMT400008278	PGSC0003 DMT400008547	PGSC0003 DMT400013094	PGSC0003 DMT400015224	PGSC0003 DMT400017633	PGSC0003 DMT400020366	PGSC0003 DMT400021019	PGSC0003 DMT40002160	PGSC0003 DMT400027201	PGSC0003 DMT40002779	PGSC0003 DMT40002843	PGSC0003 DMT400030682	PGSC0003 DMT400031370	PGSC0003 DMT400031714	PGSC0003 DMT400035714	PGSC0003 DMT400036026	PGSC0003 DMT400037483	PGSC0003 DMT40004198	**Transcript ID F1-Individuals**	PGSC0003 DMT40004236	PGSC0003 DMT40004568
0,49217729	0,56654011	0,22739146	5,42735772	0,45195467	21,17441197	0,60259057	0,00487714	0,00396421	0,02814944	0,51343374	0,00206056	0,11512316	5,35263768	0,87433012	0,01752126	0,02874092	0,16034486	0,49835648	1,46433942	1	0,33016292	0,20938790
-99	-99	-99	-99	-99	-99	-99	-99	-99	-99	-99	-99	-99	-99	-99	-99	-99	-99	-99	-99	2	-99	-99
-99	-99	-99	-99	-99	-99	-99	-99	-99	-99	-99	-99	-99	-99	-99	-99	-99	-99	-99	-99	3	-99	-99
-99	-99	-99	-99	-99	-99	-99	-99	-99	-99	-99	-99	-99	-99	-99	-99	-99	-99	-99	-99	4	-99	-99
-99	-99	-99	-99	-99	-99	-99	-99	-99	-99	-99	-99	-99	-99	-99	-99	-99	-99	-99	-99	5	-99	-99
0,03641685	0,64293732	0,15482926	12,17415724	0,03569213	8,10465065	0,80874119	0,10827334	0,00418299	0,02322383	0,32936291	0,00347626	0,17822233	4,40075178	2,97471200	0,04845368	0,04285924	0,04654435	0,91494061	1,18078803	6	0,44311406	0,11980417
-99	-99	-99	-99	-99	-99	-99	-99	-99	-99	-99	-99	-99	-99	-99	-99	-99	-99	-99	-99	7	-99	-99
-99	-99	-99	-99	-99	-99	-99	-99	-99	-99	-99	-99	-99	-99	-99	-99	-99	-99	-99	-99	8	-99	-99
-99	-99	-99	-99	-99	-99	-99	-99	-99	-99	-99	-99	-99	-99	-99	-99	-99	-99	-99	-99	9	-99	-99
-99	-99	-99	-99	-99	-99	-99	-99	-99	-99	-99	-99	-99	-99	-99	-99	-99	-99	-99	-99	10	-99	-99
-99	-99	-99	-99	-99	-99	-99	-99	-99	-99	-99	-99	-99	-99	-99	-99	-99	-99	-99	-99	11	-99	-99
-99	-99	-99	-99	-99	-99	-99	-99	-99	-99	-99	-99	-99	-99	-99	-99	-99	-99	-99	-99	12	-99	-99
-99	-99	-99	-99	-99	-99	-99	-99	-99	-99	-99	-99	-99	-99	-99	-99	-99	-99	-99	-99	13	-99	-99
-99	-99	-99	-99	-99	-99	-99	-99	-99	-99	-99	-99	-99	-99	-99	-99	-99	-99	-99	-99	14	-99	-99
-99	-99	-99	-99	-99	-99	-99	-99	-99	-99	-99	-99	-99	-99	-99	-99	-99	-99	-99	-99	15	-99	-99
0,20173034	0,34233958	0,31501665	5,84620330	0,19909101	11,51545890	0,29947345	0,23012665	0,00004205	0,01231448	0,97299210	0,00390490	0,07287158	5,19995811	4,42139001	0,01560876	0,03017505	0,03910508	0,25165166	1,19292224	16	0,28430276	0,18537261
0,42557933	0,15480243	0,09642159	6,75286633	0,01336404	6,15816277	0,91414821	0,00001283	0,02418908	0,02965666	0,61408063	0,01355059	0,24291105	4,06287224	1,79191226	0,02988365	0,03383124	0,16204877	0,74975960	0,88730377	17	0,31720777	0,15620364
0,15493661	0,27144842	0,72736429	2,83382369	0,47755871	23,48633995	0,99913394	0,12506500	0,03547017	0,03030606	0,19816169	0,04495888	0,11872947	5,57414295	1,33769573	0,08948254	0,06011005	0,12584763	0,69051756	2,92766402	18	0,33073554	0,11980417
0,23229017	0,47863573	0,88699631	6,00847971	0,14014576	6,62995924	0,37474990	0,00058228	0,20026747	0,02460337	0,20068435	0,00906800	0,11079771	5,18376386	0,44319085	0,11590382	0,04304531	0,00475942	0,70612720	4,23687334	19	0,44104568	0,24165152
-99	-99	-99	-99	-99	-99	-99	-99	-99	-99	-99	-99	-99	-99	-99	-99	-99	-99	-99	-99	20	-99	-99
-99	-99	-99	-99	-99	-99	-99	-99	-99	-99	-99	-99	-99	-99	-99	-99	-99	-99	-99	-99	21	-99	-99
-99	-99	-99	-99	-99	-99	-99	-99	-99	-99	-99	-99	-99	-99	-99	-99	-99	-99	-99	-99	22	-99	-99
-99	-99	-99	-99	-99	-99	-99	-99	-99	-99	-99	-99	-99	-99	-99	-99	-99	-99	-99	-99	23	-99	-99
0,31370926	0,28019439	0,18231435	1,56103188	0,04073746	0,07171904	2,16820325	0,00001498	0,10009904	0,01623780	0,63265900	0,00068221	0,28331915	3,12856266	0,03998215	0,08811308	0,00089582	0,07152047	0,38890778	1,27677119	24	0,23253181	0,01919002
-99	-99	-99	-99	-99	-99	-99	-99	-99	-99	-99	-99	-99	-99	-99	-99	-99	-99	-99	-99	25	-99	-99
-99	-99	-99	-99	-99	-99	-99	-99	-99	-99	-99	-99	-99	-99	-99	-99	-99	-99	-99	-99	26	-99	-99
-99	-99	-99	-99	-99	-99	-99	-99	-99	-99	-99	-99	-99	-99	-99	-99	-99	-99	-99	-99	27	-99	-99
-99	-99	-99	-99	-99	-99	-99	-99	-99	-99	-99	-99	-99	-99	-99	-99	-99	-99	-99	-99	28	-99	-99
-99	-99	-99	-99	-99	-99	-99	-99	-99	-99	-99	-99	-99	-99	-99	-99	-99	-99	-99	-99	29	-99	-99
-99	-99	-99	-99	-99	-99	-99	-99	-99	-99	-99	-99	-99	-99	-99	-99	-99	-99	-99	-99	30	-99	-99
-99	-99	-99	-99	-99	-99	-99	-99	-99	-99	-99	-99	-99	-99	-99	-99	-99	-99	-99	-99	31	-99	-99
-99	-99	-99	-99	-99	-99	-99	-99	-99	-99	-99	-99	-99	-99	-99	-99	-99	-99	-99	-99	32	-99	-99
0,00937153	0,32096769	0,18067891	0,31436227	0,00084985	0,58337683	0,30323328	0,01320749	0,23561446	0,01341974	0,09542427	0,00150242	0,40095160	1,95409418	0,21146635	0,11207217	0,00278617	0,00515611	0,23890352	0,86483691	33	0,20768947	0,00295116
-99	-99	-99	-99	-99	-99	-99	-99	-99	-99	-99	-99	-99	-99	-99	-99	-99	-99	-99	-99	34	-99	-99
0,14165957	0,17116979	0,17671544	4,76177681	0,02331658	7,55012223	0,82673679	0,13749966	0,00909633	0,09588839	0,41972026	0,00293078	0,05050255	3,25915960	5,63142487	0,06295654	0,03867379	0,18030359	0,93789609	0,27423792	35	0,27729625	0,10327049
-99	-99	-99	-99	-99	-99	-99	-99	-99	-99	-99	-99	-99	-99	-99	-99	-99	-99	-99	-99	36	-99	-99
-99	-99	-99	-99	-99	-99	-99	-99	-99	-99	-99	-99	-99	-99	-99	-99	-99	-99	-99	-99	37	-99	-99
-99	-99	-99	-99	-99	-99	-99	-99	-99	-99	-99	-99	-99	-99	-99	-99	-99	-99	-99	-99	38	-99	-99
-99	-99	-99	-99	-99	-99	-99	-99	-99	-99	-99	-99	-99	-99	-99	-99	-99	-99	-99	-99	39	-99	-99
0,04684374	0,30757284	0,10643171	1,86735989	0,02911689	4,23393758	0,46813612	0,07159487	0,00530291	0,01700379	0,42425395	0,00334680	0,14180694	3,02094517	1,75078266	0,00116617	0,02798893	0,04549967	0,48196490	0,42987824	40	0,15145908	0,06578945
-99	-99	-99	-99	-99	-99	-99	-99	-99	-99	-99	-99	-99	-99	-99	-99	-99	-99	-99	-99	41	-99	-99
-99	-99	-99	-99	-99	-99	-99	-99	-99	-99	-99	-99	-99	-99	-99	-99	-99	-99	-99	-99	42	-99	-99
0,29407377	0,24711423	0,52750256	5,80481464	0,01712503	24,08812175	0,32856484	0,21423261	0,19988609	0,03704057	0,31235315	0,00359013	0,22117343	2,68188988	1,10899280	0,09007372	0,03880133	0,08580764	0,61953170	1,58364613	43	0,22271181	0,39591153
-99	-99	-99	-99	-99	-99	-99	-99	-99	-99	-99	-99	-99	-99	-99	-99	-99	-99	-99	-99	44	-99	-99
-99	-99	-99	-99	-99	-99	-99	-99	-99	-99	-99	-99	-99	-99	-99	-99	-99	-99	-99	-99	45	-99	-99
0,13017281	0,44395951	0,49294548	4,88209375	0,32544824	0,02413884	0,55844999	0,08635958	0,00603471	0,02285257	0,61195607	0,00928746	0,03394870	4,81821910	1,35331717	0,00388061	0,08318689	0,16340228	0,63441553	1,38175324	46	0,44457539	0,48179790
-99	-99	-99	-99	-99	-99	-99	-99	-99	-99	-99	-99	-99	-99	-99	-99	-99	-99	-99	-99	47	-99	-99
-99	-99	-99	-99	-99	-99	-99	-99	-99	-99	-99	-99	-99	-99	-99	-99	-99	-99	-99	-99	48	-99	-99
0,44357501	0,79182307	0,48877757	5,80481464	0,08574819	18,66482400	0,50531328	0,13015025	0,00862805	0,00616472	0,14202827	0,00590949	0,17803712	5,24248199	2,44740015	0,08718659	0,02696534	0,08045077	0,31278647	0,89022998	49	0,37873220	0,14017005
0,29957726	0,54036270	0,52814281	9,70682848	0,23052578	18,30289557	0,24383887	0,00266435	0,00902411	0,03148918	0,23423036	0,00937478	0,06592639	4,64603493	0,29017598	0,06224061	0,02950289	0,10209612	0,72046487	2,51228476	50	0,30885466	0,19184326
0,14333869	0,39984147	0,26896680	3,40463467	0,02230483	14,10857134	0,09662230	0,07196803	0,03392517	0,00307223	0,19526428	0,00219510	0,00875607	3,90006995	0,11838022	0,00629971	0,02623252	0,12426570	0,09172762	1,24703331	51	0,39488378	0,04861347
-99	-99	-99	-99	-99	-99	-99	-99	-99	-99	-99	-99	-99	-99	-99	-99	-99	-99	-99	-99	52	-99	-99
-99	-99	-99	-99	-99	-99	-99	-99	-99	-99	-99	-99	-99	-99	-99	-99	-99	-99	-99	-99	53	-99	-99
-99	-99	-99	-99	-99	-99	-99	-99	-99	-99	-99	-99	-99	-99	-99	-99	-99	-99	-99	-99	54	-99	-99
-99	-99	-99	-99	-99	-99	-99	-99	-99	-99	-99	-99	-99	-99	-99	-99	-99	-99	-99	-99	55	-99	-99
-99	-99	-99	-99	-99	-99	-99	-99	-99	-99	-99	-99	-99	-99	-99	-99	-99	-99	-99	-99	56	-99	-99
0,09422476	0,23557364	0,18266220	4,21417434	0,01550901	3,82179395	0,35263559	0,23787079	0,17102155	0,02791627	0,35979574	0,00131589	0,02253404	2,85650291	2,61942472	0,00926656	0,01850745	0,05031910	0,05126969	0,52060100	57	0,15650167	0,18431559
0,19421816	0,42359281	0,22449419	5,28810198	0,01159583	7,36022566	0,32482847	0,08643444	0,40046554	0,01566567	0,73547591	0,00366937	0,14934802	4,33715800	1,60742341	0,01839873	0,03130962	0,10364698	0,34766024	0,55353636	58	0,13506727	0,20204520
-99	-99	-99	-99	-99	-99	-99	-99	-99	-99	-99	-99	-99	-99	-99	-99	-99	-99	-99	-99	59	-99	-99
-99	-99	-99	-99	-99	-99	-99	-99	-99	-99	-99	-99	-99	-99	-99	-99	-99	-99	-99	-99	60	-99	-99
0,12990240	0,16126441	0,19553517	3,59128463	0,01515040	0,00861162	0,89595613	0,11037612	0,18447536	0,03725299	0,28707508	0,00100785	0,16465292	1,91919327	1,10228707	0,01338258	0,03875429	0,04306023	0,30176555	0,28410576	61	0,13071531	0,04211564
-99	-99	-99	-99	-99	-99	-99	-99	-99	-99	-99	-99	-99	-99	-99	-99	-99	-99	-99	-99	62	-99	-99
-99	-99	-99	-99	-99	-99	-99	-99	-99	-99	-99	-99	-99	-99	-99	-99	-99	-99	-99	-99	63	-99	-99
-99	-99	-99	-99	-99	-99	-99	-99	-99	-99	-99	-99	-99	-99	-99	-99	-99	-99	-99	-99	64	-99	-99
0,00756995	0,36895071	0,95429023	3,38111714	0,05261070	12,47960183	0,17188315	0,03918649	0,00027706	0,00207237	0,31764781	0,05138532	0,09160055	3,51493985	2,55177301	0,00027706	0,00778277	0,17200233	1,72727764	2,76159164	65	0,23610492	0,13626623
-99	-99	-99	-99	-99	-99	-99	-99	-99	-99	-99	-99	-99	-99	-99	-99	-99	-99	-99	-99	66	-99	-99
-99	-99	-99	-99	-99	-99	-99	-99	-99	-99	-99	-99	-99	-99	-99	-99	-99	-99	-99	-99	67	-99	-99
-99	-99	-99	-99	-99	-99	-99	-99	-99	-99	-99	-99	-99	-99	-99	-99	-99	-99	-99	-99	68	-99	-99
-99	-99	-99	-99	-99	-99	-99	-99	-99	-99	-99	-99	-99	-99	-99	-99	-99	-99	-99	-99	69	-99	-99
-99	-99	-99	-99	-99	-99	-99	-99	-99	-99	-99	-99	-99	-99	-99	-99	-99	-99	-99	-99	70	-99	-99

-99	-99	-99	-99	-99	-99	-99	-99	-99	-99	-99	-99	-99	-99	-99	-99	-99	-99	-99	-99	71	-99	-99
-99	-99	-99	-99	-99	-99	-99	-99	-99	-99	-99	-99	-99	-99	-99	-99	-99	-99	-99	-99	72	-99	-99
-99	-99	-99	-99	-99	-99	-99	-99	-99	-99	-99	-99	-99	-99	-99	-99	-99	-99	-99	-99	73	-99	-99
-99	-99	-99	-99	-99	-99	-99	-99	-99	-99	-99	-99	-99	-99	-99	-99	-99	-99	-99	-99	74	-99	-99
0,07979820	0,12061657	0,09223768	1,71653586	0,00361572	1,75260393	0,45929723	0,05956051	0,16857938	0,02732755	0,51996989	0,00172822	0,02499009	2,84908764	0,44365188	0,01085497	0,03025883	0,04208646	0,43906304	0,28214330	75	0,13980618	0,02181554
-99	-99	-99	-99	-99	-99	-99	-99	-99	-99	-99	-99	-99	-99	-99	-99	-99	-99	-99	-99	76	-99	-99
-99	-99	-99	-99	-99	-99	-99	-99	-99	-99	-99	-99	-99	-99	-99	-99	-99	-99	-99	-99	77	-99	-99
-99	-99	-99	-99	-99	-99	-99	-99	-99	-99	-99	-99	-99	-99	-99	-99	-99	-99	-99	-99	78	-99	-99
-99	-99	-99	-99	-99	-99	-99	-99	-99	-99	-99	-99	-99	-99	-99	-99	-99	-99	-99	-99	79	-99	-99
-99	-99	-99	-99	-99	-99	-99	-99	-99	-99	-99	-99	-99	-99	-99	-99	-99	-99	-99	-99	80	-99	-99
-99	-99	-99	-99	-99	-99	-99	-99	-99	-99	-99	-99	-99	-99	-99	-99	-99	-99	-99	-99	81	-99	-99
-99	-99	-99	-99	-99	-99	-99	-99	-99	-99	-99	-99	-99	-99	-99	-99	-99	-99	-99	-99	82	-99	-99
-99	-99	-99	-99	-99	-99	-99	-99	-99	-99	-99	-99	-99	-99	-99	-99	-99	-99	-99	-99	83	-99	-99
0,10135576	0,06659234	0,13588895	3,83838653	0,01037676	4,68971418	0,17708329	0,08714127	0,00972895	0,00615298	0,25305099	0,00895867	0,08952907	1,61944559	1,34396913	0,00317396	0,01341044	0,02523378	0,51139147	1,12077758	84	0,05634768	0,21771308
-99	-99	-99	-99	-99	-99	-99	-99	-99	-99	-99	-99	-99	-99	-99	-99	-99	-99	-99	-99	85	-99	-99
-99	-99	-99	-99	-99	-99	-99	-99	-99	-99	-99	-99	-99	-99	-99	-99	-99	-99	-99	-99	86	-99	-99
0,13728539	0,25725125	0,09802204	4,44212503	0,10161956	4,02293938	0,56850699	0,10571485	0,13215015	0,00752157	0,63650776	0,00134074	0,09158467	3,42534665	0,68030330	0,01078560	0,02551077	0,08129159	0,22040823	1,07344628	87	0,34323058	0,14341323
0,19648621	0,22727328	0,06733498	2,67492789	0,00578086	7,31318572	0,50856356	0,25658345	0,14463614	0,01896522	0,38249161	0,00576485	0,06544832	3,25915960	0,19799007	0,06383538	0,02516391	0,01045618	0,15437382	0,77405072	88	0,18809064	0,16718299
0,07945325	0,53449561	0,28248575	6,72833715	0,20366218	6,64491157	0,48945563	0,22086703	0,00324459	0,05708478	0,76963697	0,00279536	0,14271907	5,29727351	4,00069321	0,08033932	0,03553169	0,10615542	0,76167635	1,26773218	89	0,25814436	0,24591813
-99	-99	-99	-99	-99	-99	-99	-99	-99	-99	-99	-99	-99	-99	-99	-99	-99	-99	-99	-99	90	-99	-99
-99	-99	-99	-99	-99	-99	-99	-99	-99	-99	-99	-99	-99	-99	-99	-99	-99	-99	-99	-99	91	-99	-99
-99	-99	-99	-99	-99	-99	-99	-99	-99	-99	-99	-99	-99	-99	-99	-99	-99	-99	-99	-99	92	-99	-99
-99	-99	-99	-99	-99	-99	-99	-99	-99	-99	-99	-99	-99	-99	-99	-99	-99	-99	-99	-99	93	-99	-99
-99	-99	-99	-99	-99	-99	-99	-99	-99	-99	-99	-99	-99	-99	-99	-99	-99	-99	-99	-99	94	-99	-99
-99	-99	-99	-99	-99	-99	-99	-99	-99	-99	-99	-99	-99	-99	-99	-99	-99	-99	-99	-99	95	-99	-99
-99	-99	-99	-99	-99	-99	-99	-99	-99	-99	-99	-99	-99	-99	-99	-99	-99	-99	-99	-99	96	-99	-99
-99	-99	-99	-99	-99	-99	-99	-99	-99	-99	-99	-99	-99	-99	-99	-99	-99	-99	-99	-99	97	-99	-99
-99	-99	-99	-99	-99	-99	-99	-99	-99	-99	-99	-99	-99	-99	-99	-99	-99	-99	-99	-99	98	-99	-99
0,16726993	0,46789282	0,13178426	2,72687771	0,05684787	6,43192240	0,25406156	0,00459490	0,03928848	0,02498576	0,09389877	0,00346303	0,10586151	3,02356375	0,46789282	0,07884975	0,02747475	0,06788563	0,29002517	0,42609588	99	0,26558582	0,03958918
0,16910603	0,58277060	0,56097174	10,10501809	0,00646112	15,95570002	0,81281560	0,00001662	0,29771421	0,01826214	0,17924441	0,00920096	0,10323471	5,23794169	0,65112209	0,08555524	0,03326157	0,13631347	0,87417862	1,29145735	100	0,46748760	0,40444067
-99	-99	-99	-99	-99	-99	-99	-99	-99	-99	-99	-99	-99	-99	-99	-99	-99	-99	-99	-99	101	-99	-99
-99	-99	-99	-99	-99	-99	-99	-99	-99	-99	-99	-99	-99	-99	-99	-99	-99	-99	-99	-99	102	-99	-99
0,79816006	0,65326941	0,32686119	8,43127023	0,03089999	47,79374788	0,67024014	0,21549854	0,02014757	0,02123739	0,52367731	0,00479834	0,11124019	3,41231303	2,71085886	0,02366256	0,08014465	0,08818946	1,07904124	1,46383201	103	0,23647343	0,77579641
0,07625628	0,52704571	0,31121856	10,09101927	0,05165314	12,80823697	0,24948068	0,19902202	0,02745095	0,02456929	0,13707145	0,00481917	0,01171093	2,77021894	0,25243782	0,01556015	0,02322786	0,07041370	0,12345011	0,72046487	104	0,30333839	0,24434644
-99	-99	-99	-99	-99	-99	-99	-99	-99	-99	-99	-99	-99	-99	-99	-99	-99	-99	-99	-99	105	-99	-99
0,12637211	0,49234790	0,46643566	3,80197752	1,40907656	11,32744044	0,68337530	0,09060597	0,18695332	0,00399594	0,68195573	0,01504836	0,01135719	5,28077620	2,36321354	0,07714679	0,03081443	0,29994087	1,65261424	1,70143260	106	0,27850015	0,12013680
0,32573034	0,32981982	0,27050928	4,14608588	0,00962999	9,86794723	0,16617209	0,00006401	0,10760000	0,04712871	0,34838393	0,00805863	0,07784510	2,88335805	0,37055254	0,07133481	0,03302609	0,08843431	0,27088455	0,33488786	107	0,34121430	0,25609482
-99	-99	-99	-99	-99	-99	-99	-99	-99	-99	-99	-99	-99	-99	-99	-99	-99	-99	-99	-99	108	-99	-99
-99	-99	-99	-99	-99	-99	-99	-99	-99	-99	-99	-99	-99	-99	-99	-99	-99	-99	-99	-99	109	-99	-99
0,01868145	0,54933191	0,45874044	5,83102699	0,02769463	2,97677463	0,40888017	0,00002402	0,01697729	0,00995578	0,98537857	0,00847689	0,08003362	4,73626564	0,94917763	0,05265630	0,02349911	0,13293105	0,40831373	1,63438741	110	0,12698676	0,38843632
-99	-99	-99	-99	-99	-99	-99	-99	-99	-99	-99	-99	-99	-99	-99	-99	-99	-99	-99	-99	111	-99	-99
-99	-99	-99	-99	-99	-99	-99	-99	-99	-99	-99	-99	-99	-99	-99	-99	-99	-99	-99	-99	112	-99	-99
-99	-99	-99	-99	-99	-99	-99	-99	-99	-99	-99	-99	-99	-99	-99	-99	-99	-99	-99	-99	113	-99	-99
0,11544279	0,38991998	0,16067863	5,64216947	0,01154370	5,07092874	0,37300064	0,18367791	0,00593824	0,02565262	0,50078040	0,01315040	0,05479735	3,23160306	2,25988600	0,02086531	0,04398788	0,05059891	0,26012014	0,57764298	114	0,31452574	0,10898866
-99	-99	-99	-99	-99	-99	-99	-99	-99	-99	-99	-99	-99	-99	-99	-99	-99	-99	-99	-99	115	-99	-99
-99	-99	-99	-99	-99	-99	-99	-99	-99	-99	-99	-99	-99	-99	-99	-99	-99	-99	-99	-99	116	-99	-99
-99	-99	-99	-99	-99	-99	-99	-99	-99	-99	-99	-99	-99	-99	-99	-99	-99	-99	-99	-99	117	-99	-99
-99	-99	-99	-99	-99	-99	-99	-99	-99	-99	-99	-99	-99	-99	-99	-99	-99	-99	-99	-99	118	-99	-99
-99	-99	-99	-99	-99	-99	-99	-99	-99	-99	-99	-99	-99	-99	-99	-99	-99	-99	-99	-99	119	-99	-99
0,14624914	0,19045209	0,73739012	5,67059452	0,01552245	11,86384706	0,38729372	0,25998495	0,35245232	0,00022598	0,42646522	0,01054352	0,16340228	4,38780679	2,16970665	0,00003025	0,05745196	0,11610484	0,50470070	0,80023742	120	0,22197975	0,07450598
0,20978741	0,23229017	0,29833394	10,43963958	0,02606035	18,25221945	0,33634180	0,17567734	0,02438267	0,05636721	0,16031708	0,00290550	0,11999116	4,32290161	0,27990322	0,05819343	0,02748903	0,02679300	0,26224733	0,66526452	121	0,29402281	0,06224061
0,27792163	0,38628833	0,36167104	6,80101465	0,06952858	11,43789768	0,31116463	0,06952858	0,09257394	0,01075946	0,59408860	0,00125661	0,05350250	5,08324582	0,72083951	0,10151396	0,02564373	0,01966467	0,18450733	0,98196942	122	0,49337277	0,02589379
-99	-99	-99	-99	-99	-99	-99	-99	-99	-99	-99	-99	-99	-99	-99	-99	-99	-99	-99	-99	123	-99	-99
-99	-99	-99	-99	-99	-99	-99	-99	-99	-99	-99	-99	-99	-99	-99	-99	-99	-99	-99	-99	124	-99	-99
-99	-99	-99	-99	-99	-99	-99	-99	-99	-99	-99	-99	-99	-99	-99	-99	-99	-99	-99	-99	125	-99	-99
-99	-99	-99	-99	-99	-99	-99	-99	-99	-99	-99	-99	-99	-99	-99	-99	-99	-99	-99	-99	126	-99	-99
0,14311532	0,28603230	0,26873386	5,63435318	0,04630302	0,03345232	0,57325542	0,18753737	0,17268923	0,01437046	1,01554207	0,01505358	0,15945818	5,56449206	2,04379430	0,06897656	0,04161507	0,06126683	0,38803266	0,83118988	127	0,32947708	0,27821074
-99	-99	-99	-99	-99	-99	-99	-99	-99	-99	-99	-99	-99	-99	-99	-99	-99	-99	-99	-99	128	-99	-99
-99	-99	-99	-99	-99	-99	-99	-99	-99	-99	-99	-99	-99	-99	-99	-99	-99	-99	-99	-99	129	-99	-99
-99	-99	-99	-99	-99	-99	-99	-99	-99	-99	-99	-99	-99	-99	-99	-99	-99	-99	-99	-99	130	-99	-99
-99	-99	-99	-99	-99	-99	-99	-99	-99	-99	-99	-99	-99	-99	-99	-99	-99	-99	-99	-99	131	-99	-99
-99	-99	-99	-99	-99	-99	-99	-99	-99	-99	-99	-99	-99	-99	-99	-99	-99	-99	-99	-99	132	-99	-99
-99	-99	-99	-99	-99	-99	-99	-99	-99	-99	-99	-99	-99	-99	-99	-99	-99	-99	-99	-99	133	-99	-99
-99	-99	-99	-99	-99	-99	-99	-99	-99	-99	-99	-99	-99	-99	-99	-99	-99	-99	-99	-99	134	-99	-99
-99	-99	-99	-99	-99	-99	-99	-99	-99	-99	-99	-99	-99	-99	-99	-99	-99	-99	-99	-99	135	-99	-99
-99	-99	-99	-99	-99	-99	-99	-99	-99	-99	-99	-99	-99	-99	-99	-99	-99	-99	-99	-99	136	-99	-99
-99	-99	-99	-99	-99	-99	-99	-99	-99	-99	-99	-99	-99	-99	-99	-99	-99	-99	-99	-99	137	-99	-99
0,35917280	0,67630691	0,73343957	15,63546261	0,27069685	51,72357181	0,61376148	0,00003651	0,59946607	0,04739077	0,51575221	0,01086438	0,27182499	9,50541753	1,51650473	0,11997037	0,06665006	0,22731267	1,24465853	2,62942984	138	0,38628833	0,55893406
-99	-99	-99	-99	-99	-99	-99	-99	-99	-99	-99	-99	-99	-99	-99	-99	-99	-99	-99	-99	139	-99	-99
0,18323284	0,29828224	0,28972378	6,94391843	0,02806177	23,81419704	0,67116994	0,00002885	0,01873331	0,02116391	0,19408358	0,00305153	0,08165868	3,34672498	0,52258948	0,01564396	0,09347666	0,15059544	0,43369418	0,32890663	140	0,23549201	0,40661913
0,12802523	0,29658139	0,13254002	3,98477979	0,00470529	3,95725491	0,39488378	0,00265605	0,00221958	0,01192803	0,27234362	0,00429389	0,12670102	5,92781326	0,49775234	0,01469531	0,01247774	0,04110621	0,13845605	1,10305139	141	0,16340228	0,27538082
0,20571943	0,24144224	0,30858717	8,15536812	0,04128467	7,13913855	0,62883351	0,14486189	0,02917244	0,04635118	0,82917585	0,02295178	0,03738880	2,99747973	0,73956559	0,09432278	0,02625525	0,15808255	0,42861374	0,33349800	142	0,26678510	0,07334030
0,17188315	0,13008261	0,16204877	1,71891714	0,00652639	2,29978660	0,11482431	0,04621484	0,09268631	0,01229742	0,42059395	0,00073473	0,17647063	3,06417777	1,52573034	0,01264315	0,02354395	0,09172762	0,63617695	0,71474474	143	0,20582641	0,37332396
-99	-99	-99	-99	-99	-99	-99	-99	-99	-99	-99	-99	-99	-99	-99	-99	-99	-99	-99	-99	144	-99	-99
0,41101131	0,28563605	0,19919454	3,43485691	0,01226125	7,77180660	0,62905148	0,13399490	0,23313702	0,03129877	0,17137755	0,00195753	0,15596022	2,25362895	0,71909286	0,10389874	0,02286445	0,06063313	0,34466101	0,48271715	145	0,16382756	0,23919349
-99	-99	-99	-99	-99	-99	-99	-99	-99	-99	-99	-99	-99	-99	-99	-99	-99	-99	-99	-99	146	-99	-99
0,16565458	0,33245938	0,21843107	2,83480598	0,02828635	15,10809651	1,16979036	0,13891266	0,13015025	0,02698404	0,84045938	0,01133753	0,03642947	4,15327673	2,70429024	0,05824387	0,02890074	0,13548922	0,20522096	0,86468706	147	0,15585215	0,04302294
-99	-99	-99	-99	-99	-99	-99	-99	-99	-99	-99	-99	-99	-99	-99	-99	-99	-99	-99	-99	148	-99	-99
-99	-99	-99	-99	-99	-99	-99	-99	-99	-99	-99	-99	-99	-99	-99	-99	-99	-99	-99	-99	149	-99	-99
-99	-99	-99	-99	-99	-99	-99	-99	-99	-99	-99	-99	-99	-99	-99	-99	-99	-99	-99	-99	150	-99	-99
-99	-99	-99	-99	-99	-99	-99	-99	-99	-99	-99	-99	-99	-99	-99	-99	-99	-99	-99	-99	151	-99	-99
0,20689920	0,23504355	0,17850050	6,32909841	0,04937758	4,67835066	0,65022007	0,00003479	0,00279488	0,01207360	0,29874780	0,01011051	0,09037077	3,14704978	1,07400447	0,03118508	0,02259269	0,06394609	0,50697974	0,48029743	152	0,26831507	0,04063875
-99	-99	-99	-99	-99	-99	-99	-99	-99	-99	-99	-99	-99	-99	-99	-99	-99	-99	-99	-99	153	-99	-99

-99	-99	-99	-99	-99	-99	-99	-99	-99	-99	-99	-99	-99	-99	-99	-99	-99	-99	-99	-99	154	-99	-99
-99	-99	-99	-99	-99	-99	-99	-99	-99	-99	-99	-99	-99	-99	-99	-99	-99	-99	-99	-99	155	-99	-99
-99	-99	-99	-99	-99	-99	-99	-99	-99	-99	-99	-99	-99	-99	-99	-99	-99	-99	-99	-99	156	-99	-99
-99	-99	-99	-99	-99	-99	-99	-99	-99	-99	-99	-99	-99	-99	-99	-99	-99	-99	-99	-99	157	-99	-99
-99	-99	-99	-99	-99	-99	-99	-99	-99	-99	-99	-99	-99	-99	-99	-99	-99	-99	-99	-99	158	-99	-99
0,00003876	0,35202505	0,22778584	4,99245699	0,03713054	2,09180737	1,40128460	0,04080105	0,11070176	0,00860118	0,40269236	0,00091480	0,01268045	5,83507015	0,98537857	0,05504480	0,00855955	0,01778432	0,39249604	1,70379292	159	0,19981683	0,08047865
0,21692226	0,35607425	0,37171015	4,53860835	0,07047474	12,93537524	0,66537981	0,00001834	0,00383715	0,01291552	0,71909286	0,01347333	0,02958993	3,71986432	2,97780648	0,01957627	0,02098133	0,11632636	0,33176877	1,07642664	160	0,17877910	0,15857640
0,00138851	0,25397352	0,28892161	3,88725028	0,04312744	5,67550985	0,73905314	0,14466121	0,23192818	0,01463686	0,43519986	0,00347626	0,03825388	2,99332722	0,27333649	0,08003362	0,00788732	0,03232401	0,54252067	2,23728648	161	0,22048463	0,14446080
-99	-99	-99	-99	-99	-99	-99	-99	-99	-99	-99	-99	-99	-99	-99	-99	-99	-99	-99	-99	162	-99	-99
-99	-99	-99	-99	-99	-99	-99	-99	-99	-99	-99	-99	-99	-99	-99	-99	-99	-99	-99	-99	163	-99	-99
0,26324901	0,29189067	0,29658139	8,48256159	0,12582583	3,73601436	0,02528630	0,40430053	0,19018825	0,04787778	0,54961756	0,02417232	0,05170688	6,88520686	0,34281449	0,16511007	0,03811493	0,19177679	0,36869506	0,60604633	164	0,41797831	0,13401812
-99	-99	-99	-99	-99	-99	-99	-99	-99	-99	-99	-99	-99	-99	-99	-99	-99	-99	-99	-99	165	-99	-99
-99	-99	-99	-99	-99	-99	-99	-99	-99	-99	-99	-99	-99	-99	-99	-99	-99	-99	-99	-99	166	-99	-99
0,30773277	0,65191239	0,32708783	4,37641636	0,17491792	0,04868935	0,64606445	0,13469330	0,01419720	0,02040052	0,43309337	0,00343553	0,15017848	3,70571004	2,53458580	0,07613745	0,05489239	0,19207611	0,58186243	1,12798666	167	0,28593319	0,03440471
-99	-99	-99	-99	-99	-99	-99	-99	-99	-99	-99	-99	-99	-99	-99	-99	-99	-99	-99	-99	168	-99	-99
-99	-99	-99	-99	-99	-99	-99	-99	-99	-99	-99	-99	-99	-99	-99	-99	-99	-99	-99	-99	169	-99	-99
-99	-99	-99	-99	-99	-99	-99	-99	-99	-99	-99	-99	-99	-99	-99	-99	-99	-99	-99	-99	170	-99	-99
0,32163582	0,39680442	0,38249161	6,79512457	0,00933910	19,95253293	0,26034561	0,25711755	0,02636011	0,01581569	0,23940082	0,00838775	0,05199440	2,49925828	1,65806435	0,18030359	0,03845993	0,08318689	0,32589972	1,10228707	171	0,31611031	0,19351268
0,11137521	0,39324491	0,38435198	2,77117919	0,02454376	9,30818574	0,17893407	0,00560333	0,01039116	0,00963500	0,22090531	0,00446074	0,07102644	2,50272539	0,34257695	0,00786411	0,02086892	0,03878117	0,64909430	1,76234943	172	0,17561646	0,10551354
-99	-99	-99	-99	-99	-99	-99	-99	-99	-99	-99	-99	-99	-99	-99	-99	-99	-99	-99	-99	173	-99	-99
0,04853771	0,18884179	0,50812311	4,66297275	0,11893539	8,48697248	0,21707267	0,00104684	0,12907218	0,03301465	0,18457128	0,01201724	0,12764863	1,95240182	1,07717302	0,07155766	0,06630447	0,06118195	0,24591813	0,65986829	174	0,21245806	0,18923489
0,20497218	0,25908547	0,19005646	4,59001860	0,01058746	7,77854327	0,24698580	0,06387964	0,00801823	0,03132591	0,22075224	0,00463730	0,05246503	4,66054929	0,57894572	0,07533687	0,04854613	0,04781145	0,38515205	0,43815098	175	0,13364706	0,03154379
0,24913507	0,28578458	0,48430909	4,82992237	0,35453501	34,58321287	0,34388545	0,06324083	0,10302026	0,00938778	0,81563749	0,00492384	0,01832555	6,31157473	0,17519093	0,06652314	0,04799407	0,02249893	0,42425395	3,54798522	176	0,32828028	0,01862004
0,10346753	0,48004781	0,32697449	2,39371269	0,01792665	2,21952352	0,26430031	0,00274543	0,23297548	0,01831602	0,59306001	0,00663471	0,10906424	5,21349195	0,79292153	0,07923326	0,07367148	0,03335970	0,90767643	1,14017083	177	0,20693506	0,01421197
-99	-99	-99	-99	-99	-99	-99	-99	-99	-99	-99	-99	-99	-99	-99	-99	-99	-99	-99	-99	178	-99	-99
-99	-99	-99	-99	-99	-99	-99	-99	-99	-99	-99	-99	-99	-99	-99	-99	-99	-99	-99	-99	179	-99	-99
-99	-99	-99	-99	-99	-99	-99	-99	-99	-99	-99	-99	-99	-99	-99	-99	-99	-99	-99	-99	180	-99	-99
-99	-99	-99	-99	-99	-99	-99	-99	-99	-99	-99	-99	-99	-99	-99	-99	-99	-99	-99	-99	181	-99	-99
0,39928755	0,25165166	0,29885136	3,54184235	0,10720915	3,25464457	0,46089180	0,26526385	0,00629534	0,02320372	0,65225138	0,01033369	0,19635007	1,14670954	0,62222143	0,06098085	0,04127752	0,35613595	0,15182697	1,36556691	182	0,21058871	0,61322992
0,15547451	0,56712946	0,38415222	7,75297493	0,04680317	7,09966031	0,95181296	0,38388604	0,02966180	0,02520319	0,66699599	0,00525079	0,21744915	5,55100888	1,18160677	0,01499630	0,04772040	0,00468983	0,80371171	0,46321376	183	0,31660370	0,11460564
-99	-99	-99	-99	-99	-99	-99	-99	-99	-99	-99	-99	-99	-99	-99	-99	-99	-99	-99	-99	184	-99	-99
-99	-99	-99	-99	-99	-99	-99	-99	-99	-99	-99	-99	-99	-99	-99	-99	-99	-99	-99	-99	185	-99	-99
-99	-99	-99	-99	-99	-99	-99	-99	-99	-99	-99	-99	-99	-99	-99	-99	-99	-99	-99	-99	186	-99	-99
-99	-99	-99	-99	-99	-99	-99	-99	-99	-99	-99	-99	-99	-99	-99	-99	-99	-99	-99	-99	187	-99	-99
-99	-99	-99	-99	-99	-99	-99	-99	-99	-99	-99	-99	-99	-99	-99	-99	-99	-99	-99	-99	188	-99	-99
0,11883239	0,20504323	0,31121856	2,84612692	2,71132865	10,71084206	0,44689258	0,00291760	0,09401273	0,00276980	0,34651747	0,00323056	0,05715407	3,46534743	1,31859361	0,00580696	0,02913708	0,07630916	0,12024093	1,42405020	189	0,39914919	0,11866777
-99	-99	-99	-99	-99	-99	-99	-99	-99	-99	-99	-99	-99	-99	-99	-99	-99	-99	-99	-99	190	-99	-99
-99	-99	-99	-99	-99	-99	-99	-99	-99	-99	-99	-99	-99	-99	-99	-99	-99	-99	-99	-99	191	-99	-99
-99	-99	-99	-99	-99	-99	-99	-99	-99	-99	-99	-99	-99	-99	-99	-99	-99	-99	-99	-99	192	-99	-99
-99	-99	-99	-99	-99	-99	-99	-99	-99	-99	-99	-99	-99	-99	-99	-99	-99	-99	-99	-99	193	-99	-99
-99	-99	-99	-99	-99	-99	-99	-99	-99	-99	-99	-99	-99	-99	-99	-99	-99	-99	-99	-99	194	-99	-99
-99	-99	-99	-99	-99	-99	-99	-99	-99	-99	-99	-99	-99	-99	-99	-99	-99	-99	-99	-99	195	-99	-99
-99	-99	-99	-99	-99	-99	-99	-99	-99	-99	-99	-99	-99	-99	-99	-99	-99	-99	-99	-99	196	-99	-99
-99	-99	-99	-99	-99	-99	-99	-99	-99	-99	-99	-99	-99	-99	-99	-99	-99	-99	-99	-99	197	-99	-99
0,24396566	0,34910913	0,18943174	2,50315912	0,21150300	4,51194662	0,80148643	0,00001668	0,07822372	0,01354355	0,30035696	0,00461245	0,09491303	4,70354987	0,50234485	0,06384644	0,02153385	0,08794528	0,43610577	0,65735746	198	0,19912552	0,02727551
0,59326558	0,32867873	0,63761170	7,68342736	0,03922045	39,55395358	0,31770286	0,41430065	0,03749261	0,07816952	0,41372671	0,00583016	0,08256934	3,60687644	2,57174928	0,11524292	0,05565866	0,09751379	0,08806728	0,36267520	199	0,28317190	0,30391715
-99	-99	-99	-99	-99	-99	-99	-99	-99	-99	-99	-99	-99	-99	-99	-99	-99	-99	-99	-99	200	-99	-99
-99	-99	-99	-99	-99	-99	-99	-99	-99	-99	-99	-99	-99	-99	-99	-99	-99	-99	-99	-99	201	-99	-99
0,08032540	0,41725464	0,69592320	6,21605815	0,70076372	0,03980242	0,51441335	0,00897421	0,24570515	0,02424784	2,85798828	0,00076249	0,10874342	5,23068539	0,61386784	0,02468879	0,00256291	0,02180798	0,35749625	1,55617035	202	0,43077308	0,20124153
0,18946457	0,24878993	0,79829839	4,60755057	0,04713688	9,76080408	1,18099266	0,19792147	0,38342066	0,03022739	1,45699911	0,00004364	0,18530838	5,94633194	2,37676621	0,00223115	0,00080429	0,15135414	3,94493082	15,03236399	203	0,58196327	0,00101733
-99	-99	-99	-99	-99	-99	-99	-99	-99	-99	-99	-99	-99	-99	-99	-99	-99	-99	-99	-99	204	-99	-99
-99	-99	-99	-99	-99	-99	-99	-99	-99	-99	-99	-99	-99	-99	-99	-99	-99	-99	-99	-99	205	-99	-99
-99	-99	-99	-99	-99	-99	-99	-99	-99	-99	-99	-99	-99	-99	-99	-99	-99	-99	-99	-99	206	-99	-99
-99	-99	-99	-99	-99	-99	-99	-99	-99	-99	-99	-99	-99	-99	-99	-99	-99	-99	-99	-99	207	-99	-99
-99	-99	-99	-99	-99	-99	-99	-99	-99	-99	-99	-99	-99	-99	-99	-99	-99	-99	-99	-99	208	-99	-99
0,20973894	0,50686262	0,34452168	8,14359981	0,10421730	30,81725949	0,54815913	0,39850415	0,00719229	0,01379861	0,07093624	0,00098517	0,00844707	3,23477791	3,36280860	0,00447985	0,01438458	0,05577130	0,51251519	1,81461900	209	0,18334930	0,14187248
-99	-99	-99	-99	-99	-99	-99	-99	-99	-99	-99	-99	-99	-99	-99	-99	-99	-99	-99	-99	210	-99	-99
-99	-99	-99	-99	-99	-99	-99	-99	-99	-99	-99	-99	-99	-99	-99	-99	-99	-99	-99	-99	211	-99	-99
0,36198454	0,34627737	0,53588673	8,28786211	0,05551417	20,80704234	0,95263800	0,44658293	0,05024069	0,03001859	0,27912824	0,00700208	0,07742804	1,39184639	1,29235283	0,01018790	0,01721727	0,09012055	0,10482102	0,76207242	212	0,32691784	0,38635527
-99	-99	-99	-99	-99	-99	-99	-99	-99	-99	-99	-99	-99	-99	-99	-99	-99	-99	-99	-99	213	-99	-99
0,19051810	0,28400732	0,19971298	4,20469164	0,07075618	11,90091010	0,16817090	0,06028739	0,21479019	0,00258611	0,56058304	0,00028873	0,05321586	1,88295693	0,73509367	0,00120814	0,03018551	0,00021416	0,71598437	0,74638919	214	0,30757284	0,02256139
-99	-99	-99	-99	-99	-99	-99	-99	-99	-99	-99	-99	-99	-99	-99	-99	-99	-99	-99	-99	215	-99	-99
0,08802151	0,31121856	0,19411722	1,90791910	0,00585547	1,16958766	0,31035687	0,12448122	0,26720150	0,00814991	0,21036987	0,00059410	0,15346696	3,94493082	0,82302035	0,05314214	0,02938044	0,01288646	0,91763988	0,49930733	216	0,15041288	0,00760941
-99	-99	-99	-99	-99	-99	-99	-99	-99	-99	-99	-99	-99	-99	-99	-99	-99	-99	-99	-99	217	-99	-99
-99	-99	-99	-99	-99	-99	-99	-99	-99	-99	-99	-99	-99	-99	-99	-99	-99	-99	-99	-99	218	-99	-99
-99	-99	-99	-99	-99	-99	-99	-99	-99	-99	-99	-99	-99	-99	-99	-99	-99	-99	-99	-99	219	-99	-99
0,36786543	0,46789282	0,30741298	6,46768762	0,32674793	36,28370815	0,65576463	0,17939978	0,49217729	0,01036059	0,18495549	0,00192924	0,07967384	5,54139804	0,76538100	0,08492011	0,02739868	0,05241959	0,19267616	0,95677394	220	0,33477182	0,07945325
0,37222581	0,45509827	0,37325927	4,89480030	0,04717774	37,56325036	0,56184730	0,25423772	0,08033932	0,02502042	0,04928355	0,00176545	0,09215779	3,43723860	0,49183626	0,02969266	0,04308263	0,07165693	0,03912542	1,06234326	221	0,33453986	0,53597960
0,20061481	0,32680456	0,16160010	5,16762005	0,01892582	3,03879636	0,78922032	0,00001032	0,03816780	0,06022474	0,52105227	0,00551853	0,10355722	4,89564857	0,35416658	0,10493006	0,01129243	0,04868091	0,40880932	0,52649802	222	0,17464533	0,08965327
0,15033470	0,44852180	0,34561794	5,88584683	0,01615640	1,71623844	1,47657025	0,09139443	0,32584325	0,04513061	0,38428538	0,00422817	0,05216587	1,70674793	2,22260257	0,02652047	0,02866134	0,05430580	0,39317677	0,63079800	223	0,19002353	0,02222381
-99	-99	-99	-99	-99	-99	-99	-99	-99	-99	-99	-99	-99	-99	-99	-99	-99	-99	-99	-99	224	-99	-99
0,12789218	0,34174686	0,39147715	6,40855902	0,02938044	13,73704698	0,80832087	0,19438651	0,01891927	0,02030530	0,67736249	0,01702738	0,13021793	2,93833727	1,10190512	0,02989400	0,04487327	0,08514113	0,64215790	0,36933452	225	0,32623874	0,11907975
0,15571718	0,18199869	0,19224260	2,64634288	1,89342726	0,01775046	0,70514898	0,00238797	0,01167851	0,02989400	0,25261286	0,00115331	0,10980381	4,92116538	1,45800938	0,00848423	0,05601667	0,14813660	0,30166098	1,12505848	226	0,31709785	0,04710422
-99	-99	-99	-99	-99	-99	-99	-99	-99	-99	-99	-99	-99	-99	-99	-99	-99	-99	-99	-99	227	-99	-99
-99	-99	-99	-99	-99	-99	-99	-99	-99	-99	-99	-99	-99	-99	-99	-99	-99	-99	-99	-99	228	-99	-99
-99	-99	-99	-99	-99	-99	-99	-99	-99	-99	-99	-99	-99	-99	-99	-99	-99	-99	-99	-99	229	-99	-99
-99	-99	-99	-99	-99	-99	-99	-99	-99	-99	-99	-99	-99	-99	-99	-99	-99	-99	-99	-99	230	-99	-99
-99	-99	-99	-99	-99	-99	-99	-99	-99	-99	-99	-99	-99	-99	-99	-99	-99	-99	-99	-99	231	-99	-99
0,11534281	0,45691573	0,09951101	6,22468141	0,03090534	5,56352789	0,33401852	0,00001836	0,02080393	0,03952749	0,37474990	0,00403281	0,08127751	4,24569284	2,44189303	0,03014369	0,04195539	0,04471802	0,35281896	0,84674531	232	0,32152437	0,06066465
-99,00000000	-99,00000000	-99,00000000	-99,00000000	-99,00000000	-99,00000000	-99,00000000	-99,00000000	-99,00000000	-99,00000000	-99,00000000	-99,00000000	-99,00000000	-99,00000000	-99,00000000	-99,00000000	-99,00000000	-99,00000000	-99,00000000	-99,00000000	233	-99,00000000	-99,00000000
0,19898754	0,40297158	0,46578949	7,27400542	4,04671150	18,63250834	1,91819581	0,10307383	0,19287660	0,03355682	0,92322231	0,00022076	0,03862691	5,44808798	2,30817082	0,07629593	0,10656089	0,11750141	0,75249297	0,76724008	234	0,60029768	0,30286568
0,18070396	0,44590244	0,68896376	5,42040256	0,04107488	23,64232479	0,55072347	0,27583931	0,24409252	0,00204852	1,06629010	0,00084803	0,07528467	5,44601139	0,87115419	0,00727115	0,00627399	0,03016878	0,37916560	1,55164659	235	0,54923672	0,11218876
-99	-99	-99	-99	-99	-99	-99	-99	-99	-99	-99	-99	-99	-99	-99	-99	-99	-99	-99	-99	236	-99	-99

-99	-99	-99	-99	-99	-99	-99	-99	-99	-99	-99	-99	-99	-99	-99	-99	-99	-99	-99	-99	237	-99	-99
-99	-99	-99	-99	-99	-99	-99	-99	-99	-99	-99	-99	-99	-99	-99	-99	-99	-99	-99	-99	238	-99	-99
-99	-99	-99	-99	-99	-99	-99	-99	-99	-99	-99	-99	-99	-99	-99	-99	-99	-99	-99	-99	239	-99	-99
-99	-99	-99	-99	-99	-99	-99	-99	-99	-99	-99	-99	-99	-99	-99	-99	-99	-99	-99	-99	240	-99	-99
-99	-99	-99	-99	-99	-99	-99	-99	-99	-99	-99	-99	-99	-99	-99	-99	-99	-99	-99	-99	241	-99	-99
-99	-99	-99	-99	-99	-99	-99	-99	-99	-99	-99	-99	-99	-99	-99	-99	-99	-99	-99	-99	242	-99	-99
0,16919396	0,35693914	0,09597151	5,27711711	0,01297385	4,23613921	0,07714679	0,00000894	0,00896022	0,02024557	0,21729848	0,00102565	0,13302322	4,58127765	0,34121430	0,13055684	0,02451400	0,01857814	0,02508120	0,69387611	243	0,20902538	0,04007232
0,26706263	0,53412525	0,38375302	15,63004471	0,02193305	19,81127739	0,41329677	0,25283183	0,01408448	0,01671457	0,20408612	0,00095464	0,23573698	6,57162428	0,64405241	0,07521947	0,02284861	0,05533170	0,78689879	1,55967993	244	0,41415709	0,12597855
-99	-99	-99	-99	-99	-99	-99	-99	-99	-99	-99	-99	-99	-99	-99	-99	-99	-99	-99	-99	245	-99	-99
0,42713085	0,59946607	0,32822340	12,44720570	0,10502102	8,39046054	0,36875896	0,10685675	0,00375167	0,01414808	0,30286568	0,00607774	0,00855362	6,05761547	0,37729111	0,06646552	0,01799513	0,16399799	0,27985472	1,46687913	246	0,30223654	0,44434433
-99	-99	-99	-99	-99	-99	-99	-99	-99	-99	-99	-99	-99	-99	-99	-99	-99	-99	-99	-99	247	-99	-99
0,33394907	0,31264557	0,27027500	4,08858037	0,01220317	7,07877637	0,32583195	0,00001161	0,32268535	0,05346357	0,29027656	0,00901411	0,16999922	2,09812408	1,01557726	0,06570286	0,02746237	0,02247710	0,28530956	0,63437156	248	0,52631558	0,09177214
0,17500888	0,32342430	0,14685863	6,05656585	0,04595928	2,04840360	0,33022014	0,04922381	0,01005112	0,03954119	0,35367594	0,00347565	0,08249783	4,21490467	0,55305697	0,02236676	0,03032182	0,00498048	0,20243070	0,53905336	249	0,37332396	0,12882638
-99	-99	-99	-99	-99	-99	-99	-99	-99	-99	-99	-99	-99	-99	-99	-99	-99	-99	-99	-99	250	-99	-99
0,68717526	0,42742702	0,14021863	8,72859833	0,00366683	0,01836051	0,47360296	0,12867021	0,02643787	0,01956949	0,12911692	0,01055266	0,03286623	4,72642709	1,76021298	0,09887503	0,05096851	0,09004250	0,50865170	1,12955146	251	0,41143887	0,38709243
-99	-99	-99	-99	-99	-99	-99	-99	-99	-99	-99	-99	-99	-99	-99	-99	-99	-99	-99	-99	252	-99	-99
-99	-99	-99	-99	-99	-99	-99	-99	-99	-99	-99	-99	-99	-99	-99	-99	-99	-99	-99	-99	253	-99	-99
-99	-99	-99	-99	-99	-99	-99	-99	-99	-99	-99	-99	-99	-99	-99	-99	-99	-99	-99	-99	254	-99	-99
-99	-99	-99	-99	-99	-99	-99	-99	-99	-99	-99	-99	-99	-99	-99	-99	-99	-99	-99	-99	255	-99	-99
0,07048573	0,38039627	0,33921574	5,78122411	0,03639477	0,02249542	0,11494973	0,19466629	0,19549112	0,02386558	0,79398024	0,00983523	0,08334705	7,98601067	3,26385055	0,00204112	0,01761411	0,04568534	0,40971000	0,72035252	256	0,42518865	0,18480491
-99	-99	-99	-99	-99	-99	-99	-99	-99	-99	-99	-99	-99	-99	-99	-99	-99	-99	-99	-99	257	-99	-99
-99	-99	-99	-99	-99	-99	-99	-99	-99	-99	-99	-99	-99	-99	-99	-99	-99	-99	-99	-99	258	-99	-99
0,47755871	0,54478164	0,31376362	6,82935817	0,02913203	16,87423274	0,15742647	0,09367124	0,13790532	0,03884170	0,59039401	0,00897887	0,20571943	5,22977906	0,70698426	0,10993708	0,03919328	0,06048621	0,16399799	1,45372058	259	0,37390664	0,14566741
-99	-99	-99	-99	-99	-99	-99	-99	-99	-99	-99	-99	-99	-99	-99	-99	-99	-99	-99	-99	260	-99	-99
0,16391843	0,28119639	0,22635358	8,52884275	0,03263242	12,53986577	0,66298586	0,20240965	0,01188141	0,02919267	0,26037268	0,01416083	0,12846524	2,98028438	3,64608956	0,00791854	0,01281387	0,13900898	0,07770493	1,22616234	261	0,37575130	0,39328580
0,17899609	0,46716367	0,52558645	7,63696811	0,02399704	10,83779982	0,83973149	0,40276215	0,03417298	0,01737311	0,18633880	0,00569930	0,17924441	5,74776071	0,96861819	0,02293985	0,04313492	0,23667841	0,33962157	3,15141554	262	0,35330841	0,17111048
-99	-99	-99	-99	-99	-99	-99	-99	-99	-99	-99	-99	-99	-99	-99	-99	-99	-99	-99	-99	263	-99	-99
0,17262939	0,22294349	0,22048463	1,51440387	0,07472579	6,99706552	0,20643364	0,13228762	0,01256670	0,01307314	0,08818946	0,00210167	0,09136276	2,28588052	0,59822081	0,07915092	0,01872033	0,03825388	0,12663517	0,66792129	264	0,13155610	0,09819205
-99,00000000	-99,00000000	-99,00000000	-99,00000000	-99,00000000	-99,00000000	-99,00000000	-99,00000000	-99,00000000	-99,00000000	-99,00000000	-99,00000000	-99,00000000	-99,00000000	-99,00000000	-99,00000000	-99,00000000	-99,00000000	-99,00000000	-99,00000000	265	-99,00000000	-99,00000000

JKI Shelter_Co [68015]																				Location_ Treatment		
50	32	31	28	19	26	5	36	20	29	14	30	9	13	2	18	16	10	7	34	**Ranking**	11	12
**Reticuline_ oxidase**	**Serine-threonine_ protein_kinase _plant-type**	**TMV_ resistance_ protein_N**	**Homoserine_ dehydroge nase_family_ protein**	**Cc-nbs-lrr resistance protein**	**UDP-glucose: glucosyltransfe rase**	**Methionyl-tRNA_ synthetase**	**70_kDa_ subunit_of_ replication_ protein_A**	**Gene_of_ unknown_ function**	**RING_finger_ protein**	**Flagellin-sensing 2**	**Serine-threonine protein kinase, plant-type**	**TIR-NBS-LRR_disease_ resistance**	**LRR receptor-like serine/ threonine-protein kinase**	**Poly(ADP-ribose)_glyco hydrolase**	**Bacterial_spot _disease_ resistance_ protein_4**	**Extensin**	**Lipoxygenase**	**Betaine_aldeh yde_ dehyrogenase**	**Fatty_acid_ desaturase**	**Gene product**	**Glutathione-S-transferase**	**Bacterial_spot _disease_ resistance_ protein_4**
PGSC0003 DMT40004630	PGSC0003 DMT40004644	PGSC0003 DMT40004689	PGSC0003 DMT40004811	PGSC0003 DMT40004909	PGSC0003 DMT40004912	PGSC0003 DMT40004917	PGSC0003 DMT40005251	PGSC0003 DMT40006237	PGSC0003 DMT40006687	PGSC0003 DMT40006877	PGSC0003 DMT40006878	PGSC0003 DMT40007144	PGSC0003 DMT40007188	PGSC0003 DMT40007551	PGSC0003 DMT40008081	PGSC0003 DMT40008201	PGSC0003 DMT40008202	PGSC0003 DMT40008302	PGSC0003 DMT40008385	**Transcript ID F1-Individuals**	PGSC0003 DMT400004539	PGSC0003 DMT40000623
4,80071736	0,06080147	0,10808588	0,11058672	0,15127547	3,01101522	0,16202069	0,14898614	0,00001309	1,14969406	0,04207917	0,01984955	0,84337724	0,17902711	1,00643221	0,15509779	2,23496153	3,68394111	0,57285821	0,16247053	1	4,60755057	0,09855002
-99	-99	-99	-99	-99	-99	-99	-99	-99	-99	-99	-99	-99	-99	-99	-99	-99	-99	-99	-99	2	-99	-99
-99	-99	-99	-99	-99	-99	-99	-99	-99	-99	-99	-99	-99	-99	-99	-99	-99	-99	-99	-99	3	-99	-99
-99	-99	-99	-99	-99	-99	-99	-99	-99	-99	-99	-99	-99	-99	-99	-99	-99	-99	-99	-99	4	-99	-99
-99	-99	-99	-99	-99	-99	-99	-99	-99	-99	-99	-99	-99	-99	-99	-99	-99	-99	-99	-99	5	-99	-99
1,50707373	0,05128746	0,06688145	0,06541431	0,22921129	4,17492419	0,10745094	0,12602222	0,02296770	1,09258839	0,03223451	0,03651796	0,17334883	0,15185328	1,13504540	0,12929604	2,62396777	2,74679644	0,92066614	0,03754462	6	0,42868802	0,12049123
-99	-99	-99	-99	-99	-99	-99	-99	-99	-99	-99	-99	-99	-99	-99	-99	-99	-99	-99	-99	7	-99	-99
-99	-99	-99	-99	-99	-99	-99	-99	-99	-99	-99	-99	-99	-99	-99	-99	-99	-99	-99	-99	8	-99	-99
-99	-99	-99	-99	-99	-99	-99	-99	-99	-99	-99	-99	-99	-99	-99	-99	-99	-99	-99	-99	9	-99	-99
-99	-99	-99	-99	-99	-99	-99	-99	-99	-99	-99	-99	-99	-99	-99	-99	-99	-99	-99	-99	10	-99	-99
-99	-99	-99	-99	-99	-99	-99	-99	-99	-99	-99	-99	-99	-99	-99	-99	-99	-99	-99	-99	11	-99	-99
-99	-99	-99	-99	-99	-99	-99	-99	-99	-99	-99	-99	-99	-99	-99	-99	-99	-99	-99	-99	12	-99	-99
-99	-99	-99	-99	-99	-99	-99	-99	-99	-99	-99	-99	-99	-99	-99	-99	-99	-99	-99	-99	13	-99	-99
-99	-99	-99	-99	-99	-99	-99	-99	-99	-99	-99	-99	-99	-99	-99	-99	-99	-99	-99	-99	14	-99	-99
-99	-99	-99	-99	-99	-99	-99	-99	-99	-99	-99	-99	-99	-99	-99	-99	-99	-99	-99	-99	15	-99	-99
3,67183187	0,04984180	0,05163525	0,14849641	0,22151864	4,08829698	0,16718299	0,11928628	0,03808852	1,40883241	0,01056546	0,01386412	0,23285439	0,19458872	1,42849897	0,20215027	4,64120686	6,05236922	1,47171672	0,06153282	16	7,85167251	0,31930344
1,93656510	0,15109209	0,06295654	0,05634768	0,16741492	3,94083132	0,09430643	0,37487980	0,00687941	1,18632558	0,07538911	0,04912155	0,20131129	0,43062381	0,70686176	0,25112891	6,09446677	10,14361508	0,63794325	0,03042709	17	7,13666474	0,21767536
3,67246820	0,05269281	0,03068654	0,04835303	0,18579068	6,46992954	0,09491303	0,05750176	0,01514253	2,79480960	0,01219768	0,00959002	0,73293136	0,29254896	0,69243473	0,45588758	14,86399483	5,37680817	0,34959344	0,19584036	18	0,98299092	0,13939493
5,06302638	0,01327633	0,04812732	0,06497371	0,17361939	1,03168318	0,12090954	0,35551935	0,32331223	1,19499120	0,03224010	0,02122635	0,62243712	0,23915204	0,93238649	0,03922725	3,90683412	2,94649537	0,99102956	0,03700849	19	3,50035198	0,12078389
-99	-99	-99	-99	-99	-99	-99	-99	-99	-99	-99	-99	-99	-99	-99	-99	-99	-99	-99	-99	20	-99	-99
-99	-99	-99	-99	-99	-99	-99	-99	-99	-99	-99	-99	-99	-99	-99	-99	-99	-99	-99	-99	21	-99	-99
-99	-99	-99	-99	-99	-99	-99	-99	-99	-99	-99	-99	-99	-99	-99	-99	-99	-99	-99	-99	22	-99	-99
-99	-99	-99	-99	-99	-99	-99	-99	-99	-99	-99	-99	-99	-99	-99	-99	-99	-99	-99	-99	23	-99	-99
2,06981238	0,06650009	0,14706237	0,00001498	0,09824311	2,55000487	0,14058358	1,40785622	0,10878111	3,91089824	0,03970598	0,06187498	0,40937645	0,11183937	0,40262258	0,16465292	2,71414914	2,93731909	0,00001498	0,00837033	24	11,07703607	0,10878111
-99	-99	-99	-99	-99	-99	-99	-99	-99	-99	-99	-99	-99	-99	-99	-99	-99	-99	-99	-99	25	-99	-99
-99	-99	-99	-99	-99	-99	-99	-99	-99	-99	-99	-99	-99	-99	-99	-99	-99	-99	-99	-99	26	-99	-99
-99	-99	-99	-99	-99	-99	-99	-99	-99	-99	-99	-99	-99	-99	-99	-99	-99	-99	-99	-99	27	-99	-99
-99	-99	-99	-99	-99	-99	-99	-99	-99	-99	-99	-99	-99	-99	-99	-99	-99	-99	-99	-99	28	-99	-99
-99	-99	-99	-99	-99	-99	-99	-99	-99	-99	-99	-99	-99	-99	-99	-99	-99	-99	-99	-99	29	-99	-99
-99	-99	-99	-99	-99	-99	-99	-99	-99	-99	-99	-99	-99	-99	-99	-99	-99	-99	-99	-99	30	-99	-99
-99	-99	-99	-99	-99	-99	-99	-99	-99	-99	-99	-99	-99	-99	-99	-99	-99	-99	-99	-99	31	-99	-99
-99	-99	-99	-99	-99	-99	-99	-99	-99	-99	-99	-99	-99	-99	-99	-99	-99	-99	-99	-99	32	-99	-99
2,71414914	0,04685998	0,14314012	0,04153582	0,04653629	2,41579829	0,08266956	0,62741851	0,02840916	2,03848876	0,04387369	0,02876583	0,57137111	0,16261137	0,60941630	0,19164390	0,97164418	1,94733353	0,66411270	0,03027981	33	5,09735913	0,13110095
-99	-99	-99	-99	-99	-99	-99	-99	-99	-99	-99	-99	-99	-99	-99	-99	-99	-99	-99	-99	34	-99	-99
3,73601436	0,25801020	0,03926806	0,06265181	0,30260338	4,01527834	0,05382798	0,31414445	0,03292894	0,89161945	0,05371616	0,04424015	0,76710714	1,14195041	2,51141422	0,76817132	33,30114783	26,88072231	1,74291233	0,06517669	35	4,48000394	0,20554127
-99	-99	-99	-99	-99	-99	-99	-99	-99	-99	-99	-99	-99	-99	-99	-99	-99	-99	-99	-99	36	-99	-99
-99	-99	-99	-99	-99	-99	-99	-99	-99	-99	-99	-99	-99	-99	-99	-99	-99	-99	-99	-99	37	-99	-99
-99	-99	-99	-99	-99	-99	-99	-99	-99	-99	-99	-99	-99	-99	-99	-99	-99	-99	-99	-99	38	-99	-99
-99	-99	-99	-99	-99	-99	-99	-99	-99	-99	-99	-99	-99	-99	-99	-99	-99	-99	-99	-99	39	-99	-99
1,26137741	0,05683802	0,05194037	0,03340019	0,19533197	2,84021472	0,12560798	0,13276990	0,01803571	1,33329868	0,00468414	0,00875304	0,09239765	0,45691573	3,98339901	0,14299137	5,63337691	11,69645959	1,02740144	0,13378609	40	2,80888975	0,00652865
-99	-99	-99	-99	-99	-99	-99	-99	-99	-99	-99	-99	-99	-99	-99	-99	-99	-99	-99	-99	41	-99	-99
-99	-99	-99	-99	-99	-99	-99	-99	-99	-99	-99	-99	-99	-99	-99	-99	-99	-99	-99	-99	42	-99	-99
2,12027295	0,02484759	0,05544688	0,02884070	0,28862136	2,02686500	0,03675922	0,00072399	0,00277605	0,52677179	0,01887015	0,01441036	0,41301040	0,33546868	1,27213343	0,23985759	8,31231312	12,73078963	0,81295646	0,04773694	43	5,72688249	0,29663279
-99	-99	-99	-99	-99	-99	-99	-99	-99	-99	-99	-99	-99	-99	-99	-99	-99	-99	-99	-99	44	-99	-99
-99	-99	-99	-99	-99	-99	-99	-99	-99	-99	-99	-99	-99	-99	-99	-99	-99	-99	-99	-99	45	-99	-99
2,32543401	0,06481628	0,10370088	0,11253924	0,12722905	4,28857990	0,18042861	0,00704346	0,09668930	1,12466864	0,01547947	0,02572830	0,53384765	1,17242855	3,11557835	0,19580642	23,62921834	20,48505697	1,54274541	0,34615738	46	5,94118207	0,17286887
-99	-99	-99	-99	-99	-99	-99	-99	-99	-99	-99	-99	-99	-99	-99	-99	-99	-99	-99	-99	47	-99	-99
-99	-99	-99	-99	-99	-99	-99	-99	-99	-99	-99	-99	-99	-99	-99	-99	-99	-99	-99	-99	48	-99	-99
3,74833527	0,07121130	0,13872022	0,10303811	0,40704212	2,34770315	0,07972909	0,23152662	0,07978437	0,91970941	0,01158378	0,00683070	0,34156925	0,47508251	1,54354763	0,11600428	11,22971960	7,95162654	1,97691385	0,12185607	49	0,38850363	0,22656548
3,39167986	0,08071608	0,05900578	0,08338895	0,19546741	2,45547137	0,07796660	0,03711124	0,02520755	0,66434291	0,01487465	0,01448798	0,21975989	0,29730178	0,93952275	0,41812320	9,78112222	4,03061504	1,19333574	0,08863375	50	6,29628131	0,11691241
1,72010901	0,05729289	0,06119256	0,05188639	0,07848169	2,07555908	0,03437492	0,26489637	0,00834137	1,01501426	0,03216198	0,01039836	0,39598015	0,22275041	1,81566746	0,12600038	2,73113381	0,88300897	0,71623255	0,02405532	51	4,31766107	0,08033932
-99	-99	-99	-99	-99	-99	-99	-99	-99	-99	-99	-99	-99	-99	-99	-99	-99	-99	-99	-99	52	-99	-99
-99	-99	-99	-99	-99	-99	-99	-99	-99	-99	-99	-99	-99	-99	-99	-99	-99	-99	-99	-99	53	-99	-99
-99	-99	-99	-99	-99	-99	-99	-99	-99	-99	-99	-99	-99	-99	-99	-99	-99	-99	-99	-99	54	-99	-99
-99	-99	-99	-99	-99	-99	-99	-99	-99	-99	-99	-99	-99	-99	-99	-99	-99	-99	-99	-99	55	-99	-99
-99	-99	-99	-99	-99	-99	-99	-99	-99	-99	-99	-99	-99	-99	-99	-99	-99	-99	-99	-99	56	-99	-99
2,30577222	0,13078328	0,05245594	0,10190170	0,12659129	2,15136155	0,03920686	0,06602929	0,02828635	0,61738828	0,03858677	0,02050685	0,16001178	0,68266515	1,21188265	0,03934298	7,04329251	14,45252838	0,92996608	0,07160728	57	7,96817869	0,15509779
2,15957886	0,09785233	0,06460323	0,07379925	0,22542978	4,09467799	0,10663478	0,24857446	0,02560821	0,76458563	0,01803258	0,03051685	0,13497368	0,25013000	1,63099233	0,11524292	4,81905411	13,53619170	0,99982673	0,11428833	58	11,00242780	0,23598221
-99	-99	-99	-99	-99	-99	-99	-99	-99	-99	-99	-99	-99	-99	-99	-99	-99	-99	-99	-99	59	-99	-99
-99	-99	-99	-99	-99	-99	-99	-99	-99	-99	-99	-99	-99	-99	-99	-99	-99	-99	-99	-99	60	-99	-99
2,03002853	0,11610484	0,05697607	0,08990219	0,38222658	1,82070855	0,10199003	0,41308197	0,00838194	0,61450643	0,00865950	0,01717555	0,15871386	1,02491197	1,61496176	0,31240728	8,92900860	14,74853755	0,92242275	0,03853999	61	1,08053814	0,09302421
-99	-99	-99	-99	-99	-99	-99	-99	-99	-99	-99	-99	-99	-99	-99	-99	-99	-99	-99	-99	62	-99	-99
-99	-99	-99	-99	-99	-99	-99	-99	-99	-99	-99	-99	-99	-99	-99	-99	-99	-99	-99	-99	63	-99	-99
-99	-99	-99	-99	-99	-99	-99	-99	-99	-99	-99	-99	-99	-99	-99	-99	-99	-99	-99	-99	64	-99	-99
3,10695210	0,08799101	0,04970380	0,00027706	0,21471576	2,75776591	0,06889294	0,17930654	0,00027706	1,16999308	0,03134763	0,01442285	0,55728993	0,71276579	5,09294452	1,43147254	28,97024539	7,41013589	2,65093262	0,07783161	65	4,04671150	0,06871410
-99	-99	-99	-99	-99	-99	-99	-99	-99	-99	-99	-99	-99	-99	-99	-99	-99	-99	-99	-99	66	-99	-99
-99	-99	-99	-99	-99	-99	-99	-99	-99	-99	-99	-99	-99	-99	-99	-99	-99	-99	-99	-99	67	-99	-99
-99	-99	-99	-99	-99	-99	-99	-99	-99	-99	-99	-99	-99	-99	-99	-99	-99	-99	-99	-99	68	-99	-99
-99	-99	-99	-99	-99	-99	-99	-99	-99	-99	-99	-99	-99	-99	-99	-99	-99	-99	-99	-99	69	-99	-99
-99	-99	-99	-99	-99	-99	-99	-99	-99	-99	-99	-99	-99	-99	-99	-99	-99	-99	-99	-99	70	-99	-99

-99	-99	-99	-99	-99	-99	-99	-99	-99	-99	-99	-99	-99	-99	-99	-99	-99	-99	-99	-99	71	-99	-99
-99	-99	-99	-99	-99	-99	-99	-99	-99	-99	-99	-99	-99	-99	-99	-99	-99	-99	-99	-99	72	-99	-99
-99	-99	-99	-99	-99	-99	-99	-99	-99	-99	-99	-99	-99	-99	-99	-99	-99	-99	-99	-99	73	-99	-99
-99	-99	-99	-99	-99	-99	-99	-99	-99	-99	-99	-99	-99	-99	-99	-99	-99	-99	-99	-99	74	-99	-99
1,46459320	0,08971543	0,05777143	0,03613398	0,05060767	2,46826963	0,04255581	0,03357427	0,04020448	0,73280436	0,02046780	0,02163484	0,14574315	1,39232885	2,64542589	0,15458797	6,25712532	14,11835404	1,35050595	0,07127303	75	12,40199235	0,11498360
-99	-99	-99	-99	-99	-99	-99	-99	-99	-99	-99	-99	-99	-99	-99	-99	-99	-99	-99	-99	76	-99	-99
-99	-99	-99	-99	-99	-99	-99	-99	-99	-99	-99	-99	-99	-99	-99	-99	-99	-99	-99	-99	77	-99	-99
-99	-99	-99	-99	-99	-99	-99	-99	-99	-99	-99	-99	-99	-99	-99	-99	-99	-99	-99	-99	78	-99	-99
-99	-99	-99	-99	-99	-99	-99	-99	-99	-99	-99	-99	-99	-99	-99	-99	-99	-99	-99	-99	79	-99	-99
-99	-99	-99	-99	-99	-99	-99	-99	-99	-99	-99	-99	-99	-99	-99	-99	-99	-99	-99	-99	80	-99	-99
-99	-99	-99	-99	-99	-99	-99	-99	-99	-99	-99	-99	-99	-99	-99	-99	-99	-99	-99	-99	81	-99	-99
-99	-99	-99	-99	-99	-99	-99	-99	-99	-99	-99	-99	-99	-99	-99	-99	-99	-99	-99	-99	82	-99	-99
-99	-99	-99	-99	-99	-99	-99	-99	-99	-99	-99	-99	-99	-99	-99	-99	-99	-99	-99	-99	83	-99	-99
1,43743827	0,05103037	0,04592743	0,00443915	0,00002187	1,58611788	0,06085417	0,17355923	0,01354121	0,81875289	0,02597919	0,00704834	0,32163582	0,50750713	1,73688231	0,31897163	11,34118905	12,19738520	1,07960233	0,07196803	84	0,83250163	0,18572308
-99	-99	-99	-99	-99	-99	-99	-99	-99	-99	-99	-99	-99	-99	-99	-99	-99	-99	-99	-99	85	-99	-99
-99	-99	-99	-99	-99	-99	-99	-99	-99	-99	-99	-99	-99	-99	-99	-99	-99	-99	-99	-99	86	-99	-99
1,38151383	0,04614282	0,09673957	0,15190592	0,32879266	2,54603103	0,14877974	0,25335812	0,01526372	1,05427410	0,03866709	0,02199394	0,26558582	0,32471591	1,54461790	0,24677190	6,66798103	8,88425006	1,69261047	0,05017978	87	3,05696493	0,13823549
2,18782903	0,16351558	0,04037203	0,05231977	0,22554700	2,29819307	0,09715958	0,03748611	0,02092686	0,64640040	0,07251886	0,03840665	0,23092560	0,45203300	1,30721822	0,04279245	2,58201969	2,97007631	1,27677119	0,06700906	88	4,55341830	0,05131591
3,43485691	0,07362043	0,10147878	0,03920686	0,00003249	5,52988700	0,14836781	0,11656851	0,10147878	1,25027894	0,03368500	0,01468259	0,21497637	0,48708653	1,33815942	0,56771942	8,77409301	12,46015405	0,68598551	0,12093050	89	5,29846698	0,10842542
-99	-99	-99	-99	-99	-99	-99	-99	-99	-99	-99	-99	-99	-99	-99	-99	-99	-99	-99	-99	90	-99	-99
-99	-99	-99	-99	-99	-99	-99	-99	-99	-99	-99	-99	-99	-99	-99	-99	-99	-99	-99	-99	91	-99	-99
-99	-99	-99	-99	-99	-99	-99	-99	-99	-99	-99	-99	-99	-99	-99	-99	-99	-99	-99	-99	92	-99	-99
-99	-99	-99	-99	-99	-99	-99	-99	-99	-99	-99	-99	-99	-99	-99	-99	-99	-99	-99	-99	93	-99	-99
-99	-99	-99	-99	-99	-99	-99	-99	-99	-99	-99	-99	-99	-99	-99	-99	-99	-99	-99	-99	94	-99	-99
-99	-99	-99	-99	-99	-99	-99	-99	-99	-99	-99	-99	-99	-99	-99	-99	-99	-99	-99	-99	95	-99	-99
-99	-99	-99	-99	-99	-99	-99	-99	-99	-99	-99	-99	-99	-99	-99	-99	-99	-99	-99	-99	96	-99	-99
-99	-99	-99	-99	-99	-99	-99	-99	-99	-99	-99	-99	-99	-99	-99	-99	-99	-99	-99	-99	97	-99	-99
-99	-99	-99	-99	-99	-99	-99	-99	-99	-99	-99	-99	-99	-99	-99	-99	-99	-99	-99	-99	98	-99	-99
1,82797964	0,00943508	0,17890306	0,10808588	0,06960091	2,22568589	0,05893425	0,28603230	0,00685442	0,57724273	0,02437000	0,01291552	0,59141798	0,34729896	1,14254422	0,37768359	2,85650291	2,25675531	1,69026564	0,08889524	99	3,33531957	0,06524110
2,01391110	0,03377267	0,08662937	0,09958001	0,16031708	4,62675274	0,08490539	0,18815584	0,02526003	1,15829281	0,00519738	0,01328553	0,17470587	0,44906619	1,47938751	0,08269821	5,31106063	10,15416709	0,34080065	0,09030815	100	4,09254988	0,10245058
-99	-99	-99	-99	-99	-99	-99	-99	-99	-99	-99	-99	-99	-99	-99	-99	-99	-99	-99	-99	101	-99	-99
-99	-99	-99	-99	-99	-99	-99	-99	-99	-99	-99	-99	-99	-99	-99	-99	-99	-99	-99	-99	102	-99	-99
3,72373395	0,18747238	0,09511060	0,14080300	0,30965851	4,45291477	0,20472370	0,00001588	0,01305503	1,14770351	0,02040052	0,03058038	1,24121239	0,69532049	1,26905096	0,41315356	8,80150356	9,61142272	0,87645383	0,08973098	103	3,43723860	0,35879955
2,28152743	0,03579743	0,09074739	0,07921953	0,13802486	4,05302760	0,12090954	0,00000724	0,00157985	0,73407532	0,01030866	0,01078373	0,18061630	0,34484023	0,94278454	0,08603098	3,73730939	1,49381346	2,02370640	0,08049260	104	9,60143472	0,10868690
-99	-99	-99	-99	-99	-99	-99	-99	-99	-99	-99	-99	-99	-99	-99	-99	-99	-99	-99	-99	105	-99	-99
4,39161018	0,11428833	0,04291869	0,05846634	0,10004701	3,33052590	0,05562009	0,00952378	0,04825258	1,63099233	0,02426045	0,01268924	0,92258261	0,34621737	1,60630962	0,26256563	0,04815235	2,87139140	1,47401377	0,38362004	106	1,00260268	0,07001219
2,53282956	0,04520888	0,08837303	0,03459602	0,06866649	3,34904555	0,06772114	0,15852145	0,00932132	0,50548844	0,01477959	0,00863702	0,14366196	0,43640817	0,90673319	0,49098471	0,52222738	1,50707373	0,29173897	0,08008912	107	6,84357420	0,14617313
-99	-99	-99	-99	-99	-99	-99	-99	-99	-99	-99	-99	-99	-99	-99	-99	-99	-99	-99	-99	108	-99	-99
-99	-99	-99	-99	-99	-99	-99	-99	-99	-99	-99	-99	-99	-99	-99	-99	-99	-99	-99	-99	109	-99	-99
3,13127453	0,06948041	0,02358069	0,01847541	0,36978279	2,27323999	0,11807292	0,25152087	0,04315735	1,26466037	0,02601973	0,04517755	0,50724337	0,33073554	2,97677463	0,06786211	1,12330523	4,32814850	1,08579369	0,10046396	110	5,96077534	0,42853947
-99	-99	-99	-99	-99	-99	-99	-99	-99	-99	-99	-99	-99	-99	-99	-99	-99	-99	-99	-99	111	-99	-99
-99	-99	-99	-99	-99	-99	-99	-99	-99	-99	-99	-99	-99	-99	-99	-99	-99	-99	-99	-99	112	-99	-99
-99	-99	-99	-99	-99	-99	-99	-99	-99	-99	-99	-99	-99	-99	-99	-99	-99	-99	-99	-99	113	-99	-99
1,58804302	0,04627093	0,01446039	0,11197511	0,11393240	1,76082313	0,09396387	0,01382334	0,01139267	0,54008186	0,04350274	0,07939819	0,31649399	0,85219176	1,45220991	0,08006136	9,02702123	6,77044190	1,45019811	0,32674793	114	6,48227398	0,17488761
-99	-99	-99	-99	-99	-99	-99	-99	-99	-99	-99	-99	-99	-99	-99	-99	-99	-99	-99	-99	115	-99	-99
-99	-99	-99	-99	-99	-99	-99	-99	-99	-99	-99	-99	-99	-99	-99	-99	-99	-99	-99	-99	116	-99	-99
-99	-99	-99	-99	-99	-99	-99	-99	-99	-99	-99	-99	-99	-99	-99	-99	-99	-99	-99	-99	117	-99	-99
-99	-99	-99	-99	-99	-99	-99	-99	-99	-99	-99	-99	-99	-99	-99	-99	-99	-99	-99	-99	118	-99	-99
-99	-99	-99	-99	-99	-99	-99	-99	-99	-99	-99	-99	-99	-99	-99	-99	-99	-99	-99	-99	119	-99	-99
2,12652822	0,05103037	0,04491995	0,03924085	0,07979820	2,76159164	0,17488761	0,00729690	0,00603471	0,88730377	0,00091591	0,01635074	0,21546120	0,25165166	1,85381850	0,29067925	5,06829325	6,51380260	1,09847345	0,17819145	120	5,48123124	0,10780530
1,61776270	0,00633254	0,06224061	0,14793138	0,12508667	2,49147483	0,05593907	0,57355351	0,02754625	1,21503679	0,01321207	0,02313146	0,35330841	0,12630643	1,33700050	0,36729216	10,14010018	0,91193317	0,37971792	0,04968658	121	3,33514619	0,06128807
2,59143283	0,19775005	0,07640178	0,03897654	0,17552519	3,16400088	0,07871323	0,72887837	0,02885070	0,96643861	0,06957679	0,06871410	0,98333166	1,49873987	2,29222712	0,46546674	2,65507019	5,94118207	1,07233078	0,11476463	122	1,25527199	0,20618339
-99	-99	-99	-99	-99	-99	-99	-99	-99	-99	-99	-99	-99	-99	-99	-99	-99	-99	-99	-99	123	-99	-99
-99	-99	-99	-99	-99	-99	-99	-99	-99	-99	-99	-99	-99	-99	-99	-99	-99	-99	-99	-99	124	-99	-99
-99	-99	-99	-99	-99	-99	-99	-99	-99	-99	-99	-99	-99	-99	-99	-99	-99	-99	-99	-99	125	-99	-99
-99	-99	-99	-99	-99	-99	-99	-99	-99	-99	-99	-99	-99	-99	-99	-99	-99	-99	-99	-99	126	-99	-99
2,45589691	0,19686110	0,13624262	0,14431068	0,31803336	2,77262019	0,06122438	0,48038067	0,03046402	1,02615595	0,07301061	0,03166977	0,31343756	1,32248375	5,10974041	0,23770596	3,76134855	7,39858819	1,32156740	0,06484998	127	4,92713841	0,07746831
-99	-99	-99	-99	-99	-99	-99	-99	-99	-99	-99	-99	-99	-99	-99	-99	-99	-99	-99	-99	128	-99	-99
-99	-99	-99	-99	-99	-99	-99	-99	-99	-99	-99	-99	-99	-99	-99	-99	-99	-99	-99	-99	129	-99	-99
-99	-99	-99	-99	-99	-99	-99	-99	-99	-99	-99	-99	-99	-99	-99	-99	-99	-99	-99	-99	130	-99	-99
-99	-99	-99	-99	-99	-99	-99	-99	-99	-99	-99	-99	-99	-99	-99	-99	-99	-99	-99	-99	131	-99	-99
-99	-99	-99	-99	-99	-99	-99	-99	-99	-99	-99	-99	-99	-99	-99	-99	-99	-99	-99	-99	132	-99	-99
-99	-99	-99	-99	-99	-99	-99	-99	-99	-99	-99	-99	-99	-99	-99	-99	-99	-99	-99	-99	133	-99	-99
-99	-99	-99	-99	-99	-99	-99	-99	-99	-99	-99	-99	-99	-99	-99	-99	-99	-99	-99	-99	134	-99	-99
-99	-99	-99	-99	-99	-99	-99	-99	-99	-99	-99	-99	-99	-99	-99	-99	-99	-99	-99	-99	135	-99	-99
-99	-99	-99	-99	-99	-99	-99	-99	-99	-99	-99	-99	-99	-99	-99	-99	-99	-99	-99	-99	136	-99	-99
-99	-99	-99	-99	-99	-99	-99	-99	-99	-99	-99	-99	-99	-99	-99	-99	-99	-99	-99	-99	137	-99	-99
12,13203786	0,12884871	0,04145673	0,07364595	0,19274295	3,18380064	0,18773246	0,00441231	0,01999454	0,68290178	0,04775349	0,05523590	0,19101396	0,73090206	2,00937941	0,20600482	8,50758700	11,01005675	1,02580037	0,22371750	138	7,13171969	0,26375128
-99	-99	-99	-99	-99	-99	-99	-99	-99	-99	-99	-99	-99	-99	-99	-99	-99	-99	-99	-99	139	-99	-99
3,71085081	0,25751886	0,19995538	0,04021145	0,15429359	3,57452118	0,09425742	0,35300243	0,01194665	1,03724018	0,07308656	0,01588435	0,17407127	0,83668124	1,46079122	0,32303223	3,96892968	8,26921282	0,92194334	0,13714273	140	4,03271094	0,11823672
3,46895229	0,09892644	0,11379428	0,01884728	0,12521680	4,14824183	0,06361452	0,09287924	0,01867821	0,82903218	0,01361650	0,03921366	0,57415016	0,41797831	0,76339413	0,06686986	2,01740397	1,58063033	1,20539000	0,04226186	141	7,58027401	0,11104759
3,66738061	0,38229282	0,06069620	0,06842892	0,00001910	2,64405099	0,03581605	0,05481635	0,01047976	0,74007839	0,12240632	0,03672102	0,25099839	0,71536429	0,93093349	0,05128746	3,04143042	3,40286520	0,69243473	0,08165868	142	5,32857585	0,16802526
2,43597614	0,07544139	0,10277063	0,04663316	0,21308486	2,44104688	0,06149019	0,59894690	0,01218711	0,53089557	0,01020557	0,02153012	0,57574425	0,63705949	1,07437676	0,06064363	4,75847734	2,46655935	1,14670954	0,04220331	143	6,54775345	0,09565605
-99	-99	-99	-99	-99	-99	-99	-99	-99	-99	-99	-99	-99	-99	-99	-99	-99	-99	-99	-99	144	-99	-99
1,93488792	0,12835843	0,07658735	0,09748000	0,14775204	4,12458795	0,05661193	0,02886070	0,02313547	0,74807250	0,02828635	0,03926125	0,12907218	0,69604380	0,70673928	0,26466696	4,09325913	9,68834339	0,74911027	0,04506809	145	5,27163323	0,22395022
-99	-99	-99	-99	-99	-99	-99	-99	-99	-99	-99	-99	-99	-99	-99	-99	-99	-99	-99	-99	146	-99	-99
2,54426686	0,13141938	0,12597855	0,05579385	0,05913885	2,63581662	0,08503791	0,12441652	0,01001461	0,78038070	0,04780317	0,04359329	0,16588439	0,51628873	1,84324776	0,07170661	2,95518818	14,91043043	0,80958249	0,16496707	147	10,00569864	0,26976031
-99	-99	-99	-99	-99	-99	-99	-99	-99	-99	-99	-99	-99	-99	-99	-99	-99	-99	-99	-99	148	-99	-99
-99	-99	-99	-99	-99	-99	-99	-99	-99	-99	-99	-99	-99	-99	-99	-99	-99	-99	-99	-99	149	-99	-99
-99	-99	-99	-99	-99	-99	-99	-99	-99	-99	-99	-99	-99	-99	-99	-99	-99	-99	-99	-99	150	-99	-99
-99	-99	-99	-99	-99	-99	-99	-99	-99	-99	-99	-99	-99	-99	-99	-99	-99	-99	-99	-99	151	-99	-99
1,57243458	0,02246776	0,09125200	0,03153286	0,11102835	2,62624225	0,06159683	0,19860860	0,00302049	0,90500646	0,02499875	0,01191564	0,18724512	0,51799138	1,24056730	0,25173889	1,22858698	4,59797939	1,16070391	0,09368748	152	3,61501091	0,00003142
-99	-99	-99	-99	-99	-99	-99	-99	-99	-99	-99	-99	-99	-99	-99	-99	-99	-99	-99	-99	153	-99	-99

-99	-99	-99	-99	-99	-99	-99	-99	-99	-99	-99	-99	-99	-99	-99	-99	-99	-99	-99	-99	154	-99	-99
-99	-99	-99	-99	-99	-99	-99	-99	-99	-99	-99	-99	-99	-99	-99	-99	-99	-99	-99	-99	155	-99	-99
-99	-99	-99	-99	-99	-99	-99	-99	-99	-99	-99	-99	-99	-99	-99	-99	-99	-99	-99	-99	156	-99	-99
-99	-99	-99	-99	-99	-99	-99	-99	-99	-99	-99	-99	-99	-99	-99	-99	-99	-99	-99	-99	157	-99	-99
-99	-99	-99	-99	-99	-99	-99	-99	-99	-99	-99	-99	-99	-99	-99	-99	-99	-99	-99	-99	158	-99	-99
3,60187971	0,05251051	0,08979320	0,02315953	0,00003876	4,43136143	0,13394847	0,34478048	0,02808123	1,08203713	0,01330166	0,00875152	0,18234594	0,11596409	1,30563352	0,31638432	2,76973894	1,71445496	1,04156292	0,04593539	159	3,83971704	0,00073549
2,60493974	0,10959470	0,05329892	0,05776142	0,18228276	1,79782177	0,15748103	0,00013333	0,02892077	0,71660499	0,02163109	0,03177972	0,17570778	0,38722661	0,73014252	0,26521789	1,66122789	5,25339477	0,88469373	0,07377368	160	4,92457765	0,35379854
3,69033043	0,15143284	0,14976267	0,02114925	0,00000656	2,58963721	0,06505258	0,52150392	0,02257313	0,93935996	0,01238511	0,03611520	0,25859208	0,31682322	1,02936169	0,57824388	5,15777915	3,80197752	0,72837333	0,05600696	161	4,33039912	0,00082432
-99	-99	-99	-99	-99	-99	-99	-99	-99	-99	-99	-99	-99	-99	-99	-99	-99	-99	-99	-99	162	-99	-99
-99	-99	-99	-99	-99	-99	-99	-99	-99	-99	-99	-99	-99	-99	-99	-99	-99	-99	-99	-99	163	-99	-99
4,11103029	0,16637378	0,09090478	0,03336548	0,17573823	4,26190962	0,14890871	0,22648698	0,24140040	0,83711631	0,05231977	0,01853634	0,21620923	0,46185119	1,93656510	0,15761754	0,72221485	3,72050898	0,48079707	0,28136212	164	10,85283462	0,06759217
-99	-99	-99	-99	-99	-99	-99	-99	-99	-99	-99	-99	-99	-99	-99	-99	-99	-99	-99	-99	165	-99	-99
-99	-99	-99	-99	-99	-99	-99	-99	-99	-99	-99	-99	-99	-99	-99	-99	-99	-99	-99	-99	166	-99	-99
2,90744117	0,02304743	0,07288420	0,09167995	0,06957679	2,84563377	0,09537467	0,31116463	0,02520319	0,78990442	0,00571017	0,00630080	0,45778751	0,53542262	2,23728648	0,03248123	3,06470880	9,27116091	0,69725100	0,17167478	167	4,56226421	0,26678510
-99	-99	-99	-99	-99	-99	-99	-99	-99	-99	-99	-99	-99	-99	-99	-99	-99	-99	-99	-99	168	-99	-99
-99	-99	-99	-99	-99	-99	-99	-99	-99	-99	-99	-99	-99	-99	-99	-99	-99	-99	-99	-99	169	-99	-99
-99	-99	-99	-99	-99	-99	-99	-99	-99	-99	-99	-99	-99	-99	-99	-99	-99	-99	-99	-99	170	-99	-99
3,60625147	0,15685463	0,09776758	0,24341669	0,20426303	2,40577213	0,13180710	0,36286379	0,01423415	0,73026905	0,02947223	0,04595928	0,37827308	0,37827308	1,76234943	0,12170835	11,68430486	8,52381925	0,89223769	0,07327678	171	9,94864469	0,18718023
2,08132174	0,03397224	0,03385470	0,02542691	0,17164503	1,67075478	0,07409397	0,11731830	0,02084001	0,65315622	0,00842271	0,00432375	0,41480351	0,17955528	1,11072371	0,19351268	4,10818172	3,25013580	1,43743827	0,04834465	172	5,94942400	0,32415371
-99	-99	-99	-99	-99	-99	-99	-99	-99	-99	-99	-99	-99	-99	-99	-99	-99	-99	-99	-99	173	-99	-99
1,38631009	0,11416956	0,05684787	0,09700817	0,09363878	3,52653168	0,09415947	0,35979574	0,00916436	0,46886679	0,05205751	0,04075865	0,41530697	1,07568078	2,20266486	0,19227592	8,56973148	23,13892604	0,73216971	0,47673188	174	6,76926877	0,07304858
2,16970665	0,23333910	0,10639483	0,05837522	0,00001019	2,55000487	0,10384474	0,03661301	0,02078231	0,78268299	0,03573546	0,04807731	0,49775234	0,51387879	1,62281664	0,03623430	4,30346877	4,27078124	0,55152569	0,07533687	175	7,29799418	0,16092942
1,44392920	0,08461164	0,02726133	0,04506028	0,27951546	2,04344017	0,02416395	0,00375232	0,00459729	0,54148752	0,02787277	0,02089063	0,12534705	0,11010867	0,98282060	0,11924494	5,77972159	2,87188902	0,54942711	0,33797771	176	6,35878002	0,06040241
1,78169452	0,04649599	0,05052881	0,08334561	0,33803628	2,36731223	0,13260895	0,00896954	0,02737969	0,81295646	0,01110421	0,00845049	0,64853215	0,19618002	1,05573665	0,16750197	5,80079245	7,60132016	0,94753425	0,04843689	177	5,59543386	0,17019375
-99	-99	-99	-99	-99	-99	-99	-99	-99	-99	-99	-99	-99	-99	-99	-99	-99	-99	-99	-99	178	-99	-99
-99	-99	-99	-99	-99	-99	-99	-99	-99	-99	-99	-99	-99	-99	-99	-99	-99	-99	-99	-99	179	-99	-99
-99	-99	-99	-99	-99	-99	-99	-99	-99	-99	-99	-99	-99	-99	-99	-99	-99	-99	-99	-99	180	-99	-99
-99	-99	-99	-99	-99	-99	-99	-99	-99	-99	-99	-99	-99	-99	-99	-99	-99	-99	-99	-99	181	-99	-99
2,94343343	0,27423792	0,09982189	0,16328906	0,00002226	3,43783428	0,08147493	0,00124512	0,00586562	0,82788369	0,10384474	0,05360458	0,24630196	0,76977035	0,84821388	0,15383973	2,77310069	5,79777763	1,01220394	0,27461836	182	2,85056915	0,14046183
4,10035835	0,17383012	0,16779248	0,13925007	0,17946196	4,87617533	0,09231763	0,09873805	0,09432278	1,42973721	0,10465767	0,06411252	1,00608347	0,75197156	1,45977903	0,27013454	6,39857214	8,95535113	0,81719371	0,19843659	183	7,70342489	0,03995445
-99	-99	-99	-99	-99	-99	-99	-99	-99	-99	-99	-99	-99	-99	-99	-99	-99	-99	-99	-99	184	-99	-99
-99	-99	-99	-99	-99	-99	-99	-99	-99	-99	-99	-99	-99	-99	-99	-99	-99	-99	-99	-99	185	-99	-99
-99	-99	-99	-99	-99	-99	-99	-99	-99	-99	-99	-99	-99	-99	-99	-99	-99	-99	-99	-99	186	-99	-99
-99	-99	-99	-99	-99	-99	-99	-99	-99	-99	-99	-99	-99	-99	-99	-99	-99	-99	-99	-99	187	-99	-99
-99	-99	-99	-99	-99	-99	-99	-99	-99	-99	-99	-99	-99	-99	-99	-99	-99	-99	-99	-99	188	-99	-99
2,59547753	0,07427394	0,03278092	0,03457804	0,07355667	2,47769740	0,06470406	0,23406806	0,01868468	1,04318859	0,01878858	0,01323040	0,47139227	0,25827860	1,35003799	0,16597064	17,36366995	9,11978618	0,74020665	0,57874511	189	5,03764689	0,09238164
-99	-99	-99	-99	-99	-99	-99	-99	-99	-99	-99	-99	-99	-99	-99	-99	-99	-99	-99	-99	190	-99	-99
-99	-99	-99	-99	-99	-99	-99	-99	-99	-99	-99	-99	-99	-99	-99	-99	-99	-99	-99	-99	191	-99	-99
-99	-99	-99	-99	-99	-99	-99	-99	-99	-99	-99	-99	-99	-99	-99	-99	-99	-99	-99	-99	192	-99	-99
-99	-99	-99	-99	-99	-99	-99	-99	-99	-99	-99	-99	-99	-99	-99	-99	-99	-99	-99	-99	193	-99	-99
-99	-99	-99	-99	-99	-99	-99	-99	-99	-99	-99	-99	-99	-99	-99	-99	-99	-99	-99	-99	194	-99	-99
-99	-99	-99	-99	-99	-99	-99	-99	-99	-99	-99	-99	-99	-99	-99	-99	-99	-99	-99	-99	195	-99	-99
-99	-99	-99	-99	-99	-99	-99	-99	-99	-99	-99	-99	-99	-99	-99	-99	-99	-99	-99	-99	196	-99	-99
-99	-99	-99	-99	-99	-99	-99	-99	-99	-99	-99	-99	-99	-99	-99	-99	-99	-99	-99	-99	197	-99	-99
0,71387827	0,08182866	0,10229092	0,05380000	0,00001668	2,54162291	0,10545870	0,34886723	0,01191357	0,66376755	0,01727405	0,03616530	0,76830444	0,31267808	1,38679064	0,07030397	2,67910291	2,66798397	1,27787790	0,28474650	198	1,99515385	0,11994958
2,95826235	0,11881180	0,16502426	0,05247412	0,22778584	3,33745874	0,09004250	0,60909957	0,05026681	0,67677585	0,04628697	0,04858821	0,61036748	0,94786270	1,62760430	0,34074160	21,92872459	46,64821590	0,58388251	0,12240632	199	9,53180872	0,19141158
-99	-99	-99	-99	-99	-99	-99	-99	-99	-99	-99	-99	-99	-99	-99	-99	-99	-99	-99	-99	200	-99	-99
-99	-99	-99	-99	-99	-99	-99	-99	-99	-99	-99	-99	-99	-99	-99	-99	-99	-99	-99	-99	201	-99	-99
1,41225440	0,15701780	0,07742804	0,09985649	0,24098245	3,65786036	0,13490353	0,30502513	0,01579925	0,69979293	0,03312352	0,02598819	0,20862732	0,22500048	1,41715740	0,16020599	6,16029739	2,18252775	0,87296759	0,31731772	202	6,01681501	0,01622093
5,84822979	0,02979058	0,03802257	0,05691686	0,19655432	3,06099354	0,09440454	0,21464136	0,06915608	1,11728714	0,01362122	0,00934882	0,25121596	0,26370558	1,47222686	0,25489942	12,44073657	7,58421572	1,07103082	0,13030822	203	1,96870917	0,14441075
-99	-99	-99	-99	-99	-99	-99	-99	-99	-99	-99	-99	-99	-99	-99	-99	-99	-99	-99	-99	204	-99	-99
-99	-99	-99	-99	-99	-99	-99	-99	-99	-99	-99	-99	-99	-99	-99	-99	-99	-99	-99	-99	205	-99	-99
-99	-99	-99	-99	-99	-99	-99	-99	-99	-99	-99	-99	-99	-99	-99	-99	-99	-99	-99	-99	206	-99	-99
-99	-99	-99	-99	-99	-99	-99	-99	-99	-99	-99	-99	-99	-99	-99	-99	-99	-99	-99	-99	207	-99	-99
-99	-99	-99	-99	-99	-99	-99	-99	-99	-99	-99	-99	-99	-99	-99	-99	-99	-99	-99	-99	208	-99	-99
3,13543729	0,09053796	0,03068477	0,05312986	0,07364170	2,94785726	0,11539612	0,01148319	0,02721728	0,66973704	0,01303620	0,01109972	0,49540031	0,16801555	1,43064593	0,14535642	3,27312039	1,81839632	1,02361037	0,16139487	209	4,59558971	0,06038148
-99	-99	-99	-99	-99	-99	-99	-99	-99	-99	-99	-99	-99	-99	-99	-99	-99	-99	-99	-99	210	-99	-99
-99	-99	-99	-99	-99	-99	-99	-99	-99	-99	-99	-99	-99	-99	-99	-99	-99	-99	-99	-99	211	-99	-99
3,50156532	0,29668420	0,02771863	0,09827716	0,45344516	3,17553572	0,11695294	0,10330629	0,02171747	0,78785389	0,12940812	0,08765620	0,40951835	0,23164702	1,26487954	0,19860860	19,95944915	12,04197398	0,64215790	0,12630643	212	1,16231411	0,18313761
-99	-99	-99	-99	-99	-99	-99	-99	-99	-99	-99	-99	-99	-99	-99	-99	-99	-99	-99	-99	213	-99	-99
3,47737824	0,06934809	0,06267353	0,08004749	0,00003895	2,24699981	0,09753069	0,00770494	0,05791175	0,70319660	0,01850424	0,01617601	0,64841978	0,17063671	1,92518889	0,13764270	7,07142025	3,76069681	1,08975211	0,05186841	214	7,87893154	0,30418059
-99	-99	-99	-99	-99	-99	-99	-99	-99	-99	-99	-99	-99	-99	-99	-99	-99	-99	-99	-99	215	-99	-99
2,25949443	0,05664137	0,08937406	0,01776277	0,00137724	2,26576777	0,09746311	0,59749560	0,01026587	0,84968500	0,01458875	0,01157375	0,21212698	0,00002450	1,46205745	0,22861627	7,69008743	8,85197882	1,07251662	0,04450157	216	12,45799506	0,11049094
-99	-99	-99	-99	-99	-99	-99	-99	-99	-99	-99	-99	-99	-99	-99	-99	-99	-99	-99	-99	217	-99	-99
-99	-99	-99	-99	-99	-99	-99	-99	-99	-99	-99	-99	-99	-99	-99	-99	-99	-99	-99	-99	218	-99	-99
-99	-99	-99	-99	-99	-99	-99	-99	-99	-99	-99	-99	-99	-99	-99	-99	-99	-99	-99	-99	219	-99	-99
5,13459327	0,01984955	0,08724704	0,21452980	0,11885298	3,79802657	0,11020412	0,34015165	0,01338026	0,80399030	0,02683482	0,02717173	0,17280897	0,07018225	1,64063010	0,27571506	7,85847841	5,70113830	1,06307988	0,10035956	220	4,62194470	0,33390278
2,87238672	0,20722213	0,11918297	0,02108338	0,29285329	3,95108806	0,06242423	0,11267583	0,01417262	0,67280018	0,12219439	0,08427509	0,66537981	0,13051160	1,54783319	0,31213672	9,98145408	9,85769265	1,04554126	0,14785449	221	5,46037487	0,13995161
2,47512262	0,42381307	0,08928119	0,28747333	0,13457665	6,96681860	0,19635007	1,82829643	0,14921867	1,37316025	0,03576023	0,07886342	0,23285439	0,94441967	1,10764840	0,33950389	4,07132954	4,27670590	0,60772898	0,25640566	222	2,23341291	0,04623887
1,29796367	0,20281693	0,06892877	0,09344427	0,29022627	4,33565511	0,08456767	0,00978135	0,05355815	1,47350300	0,04646377	0,04810231	0,21527460	0,39181648	1,94564702	0,13316160	19,75642494	33,08831683	0,86408791	0,04681939	223	2,19656623	0,11632636
-99	-99	-99	-99	-99	-99	-99	-99	-99	-99	-99	-99	-99	-99	-99	-99	-99	-99	-99	-99	224	-99	-99
2,11403608	0,14528926	0,13908127	0,07609788	0,31208264	3,69096997	0,15399976	0,08327343	0,01606428	0,81056512	0,10866807	0,07345477	0,17170453	0,72900469	1,64946710	0,12007436	5,15152652	6,64376019	0,31447124	0,07169419	225	4,75353242	0,06963710
2,32946717	0,12560798	0,10942392	0,06958885	0,06968539	1,92385491	0,08887984	0,01586510	0,00859075	0,92018765	0,02849791	0,04334472	0,76418826	0,78458410	2,10672207	0,28637947	0,07694653	0,70857870	0,55786966	0,86573657	226	0,91509917	0,17087343
-99	-99	-99	-99	-99	-99	-99	-99	-99	-99	-99	-99	-99	-99	-99	-99	-99	-99	-99	-99	227	-99	-99
-99	-99	-99	-99	-99	-99	-99	-99	-99	-99	-99	-99	-99	-99	-99	-99	-99	-99	-99	-99	228	-99	-99
-99	-99	-99	-99	-99	-99	-99	-99	-99	-99	-99	-99	-99	-99	-99	-99	-99	-99	-99	-99	229	-99	-99
-99	-99	-99	-99	-99	-99	-99	-99	-99	-99	-99	-99	-99	-99	-99	-99	-99	-99	-99	-99	230	-99	-99
-99	-99	-99	-99	-99	-99	-99	-99	-99	-99	-99	-99	-99	-99	-99	-99	-99	-99	-99	-99	231	-99	-99
1,81755623	0,01138281	0,15378642	0,05747188	0,17653180	2,08060053	0,12090954	0,04789437	0,00326206	1,24660119	0,02428148	0,02486052	0,30481377	0,24638733	0,29771421	0,25243782	7,07142025	4,21636569	1,32225461	0,02944160	232	6,00639769	0,15739919
-99,00000000	-99,00000000	-99,00000000	-99,00000000	-99,00000000	-99,00000000	-99,00000000	-99,00000000	-99,00000000	-99,00000000	-99,00000000	-99,00000000	-99,00000000	-99,00000000	-99,00000000	-99,00000000	-99,00000000	-99,00000000	-99,00000000	-99,00000000	233	-99,00000000	0,12015762
3,30752027	0,29519701	0,10102260	0,10053362	0,08325900	3,02461182	0,05674944	0,19529812	0,03481252	1,35825090	0,03096431	0,04635118	0,79926732	0,38123434	1,83496176	0,11556288	3,96892968	6,38970798	1,12798666	0,49680445	234	3,28467356	0,04974688
3,49501828	0,07432544	0,02000355	0,06973854	0,00011830	3,57018789	0,13164275	0,09734833	0,00011830	0,85678195	0,02159588	0,01510217	0,66149401	0,22676973	1,24582377	0,18360790	7,52139362	2,30361560	0,27232475	0,08358570	235	3,70763699	0,06106544
-99	-99	-99	-99	-99	-99	-99	-99	-99	-99	-99	-99	-99	-99	-99	-99	-99	-99	-99	-99	236	-99	-99

-99	-99	-99	-99	-99	-99	-99	-99	-99	-99	-99	-99	-99	-99	-99	-99	-99	-99	-99	-99	237	-99	-99
-99	-99	-99	-99	-99	-99	-99	-99	-99	-99	-99	-99	-99	-99	-99	-99	-99	-99	-99	-99	238	-99	-99
-99	-99	-99	-99	-99	-99	-99	-99	-99	-99	-99	-99	-99	-99	-99	-99	-99	-99	-99	-99	239	-99	-99
-99	-99	-99	-99	-99	-99	-99	-99	-99	-99	-99	-99	-99	-99	-99	-99	-99	-99	-99	-99	240	-99	-99
-99	-99	-99	-99	-99	-99	-99	-99	-99	-99	-99	-99	-99	-99	-99	-99	-99	-99	-99	-99	241	-99	-99
-99	-99	-99	-99	-99	-99	-99	-99	-99	-99	-99	-99	-99	-99	-99	-99	-99	-99	-99	-99	242	-99	-99
2,49277039	0,02703084	0,02562596	0,08782345	0,10465767	4,43136143	0,06532369	0,05899555	0,00295372	0,78336143	0,03022215	0,02744620	0,44836638	0,14168412	0,85307826	0,30858717	3,93332664	7,61450366	0,70210076	0,05383730	243	4,99159194	0,13932248
4,99072704	0,03389579	0,07511527	0,04959196	0,27667228	2,92462165	0,11552284	0,02319970	0,03195643	0,72208972	0,01657612	0,01274654	0,25004333	0,17964865	0,90893561	0,03923405	3,70185914	2,15583984	0,68313850	0,01122804	244	5,88278781	0,16637378
-99	-99	-99	-99	-99	-99	-99	-99	-99	-99	-99	-99	-99	-99	-99	-99	-99	-99	-99	-99	245	-99	-99
3,49610860	0,11271489	0,06141564	0,14997043	0,00003339	2,10489753	0,05738232	0,19652027	0,01686002	0,78881014	0,03474020	0,03952064	0,36978279	0,17589056	1,18160677	0,01865557	0,00003339	1,67220300	0,34838393	0,07339115	246	3,62441963	0,25362168
-99	-99	-99	-99	-99	-99	-99	-99	-99	-99	-99	-99	-99	-99	-99	-99	-99	-99	-99	-99	247	-99	-99
3,27342290	0,24732843	0,08071608	0,10353568	0,17547045	4,75056794	0,09625465	0,23569613	0,01696141	0,71251881	0,06446232	0,07098215	0,43627206	0,59687470	0,70495350	0,29594480	8,47844682	6,10165242	1,05870452	0,04805399	248	6,30173899	0,38070621
1,59936586	0,15642034	0,21278967	0,02082557	0,20668420	1,23071781	0,11546280	0,02382302	0,04185373	2,28706917	0,01804196	0,05257424	0,41250971	0,09509412	0,89100164	0,04874844	1,67191326	3,05569384	0,70344035	0,06898851	249	2,40077467	0,16003951
-99	-99	-99	-99	-99	-99	-99	-99	-99	-99	-99	-99	-99	-99	-99	-99	-99	-99	-99	-99	250	-99	-99
4,18361473	0,21209022	0,09901219	0,05075698	0,10465767	3,80989173	0,07283370	0,28141088	0,01572005	1,07307432	0,08583739	0,04139929	0,54101856	0,30181784	1,00329788	0,05928250	1,94902149	2,93579249	0,56634379	0,04412530	251	4,13174151	0,18221959
-99	-99	-99	-99	-99	-99	-99	-99	-99	-99	-99	-99	-99	-99	-99	-99	-99	-99	-99	-99	252	-99	-99
-99	-99	-99	-99	-99	-99	-99	-99	-99	-99	-99	-99	-99	-99	-99	-99	-99	-99	-99	-99	253	-99	-99
-99	-99	-99	-99	-99	-99	-99	-99	-99	-99	-99	-99	-99	-99	-99	-99	-99	-99	-99	-99	254	-99	-99
-99	-99	-99	-99	-99	-99	-99	-99	-99	-99	-99	-99	-99	-99	-99	-99	-99	-99	-99	-99	255	-99	-99
1,58950219	0,07748039	0,09513203	0,01936473	0,27631293	3,53564886	0,05295736	0,21188818	0,01982102	1,51306608	0,05561335	0,02851125	0,22197591	0,08382794	1,47383498	0,01388311	1,53068243	1,42037808	0,91635277	0,09500024	256	6,19992172	0,07600562
-99	-99	-99	-99	-99	-99	-99	-99	-99	-99	-99	-99	-99	-99	-99	-99	-99	-99	-99	-99	257	-99	-99
-99	-99	-99	-99	-99	-99	-99	-99	-99	-99	-99	-99	-99	-99	-99	-99	-99	-99	-99	-99	258	-99	-99
3,72373395	0,06331758	0,18158916	0,06433511	0,10932915	1,29123358	0,11685165	0,39714837	0,01744249	0,95843334	0,03626571	0,01750305	0,68242859	0,26457525	0,70502680	0,19167711	3,34672498	5,95354926	0,63938198	0,06446903	259	3,23496477	0,19502757
-99	-99	-99	-99	-99	-99	-99	-99	-99	-99	-99	-99	-99	-99	-99	-99	-99	-99	-99	-99	260	-99	-99
4,76987018	0,12987089	0,06553230	0,04299313	0,08690904	2,41990431	0,11643123	0,48874370	0,03931844	1,07754640	0,05362130	0,07109786	0,17143695	0,31851870	1,05602940	0,08244924	7,94556604	4,39861704	0,14788524	0,07919483	261	1,86833091	0,07904127
4,15255708	0,09645501	0,03450621	0,06712528	0,27376312	4,34693945	0,09665579	0,24299525	0,08808255	1,12661923	0,02463751	0,03550091	0,81620305	0,35650643	1,12272142	0,09453550	5,63337691	3,80000153	1,60213976	0,06973371	262	5,67157725	0,13394847
-99	-99	-99	-99	-99	-99	-99	-99	-99	-99	-99	-99	-99	-99	-99	-99	-99	-99	-99	-99	263	-99	-99
1,57543475	0,07385042	0,08436275	0,06057012	0,25134659	2,36813282	0,03641685	0,02027365	0,00466713	0,59697814	0,01879835	0,00770361	0,26789694	1,21230272	3,23945249	0,59615112	32,08328586	13,09323782	0,52331445	0,06509768	264	4,01249613	0,07419676
-99,00000000	-99,00000000	-99,00000000	-99,00000000	-99,00000000	-99,00000000	-99,00000000	-99,00000000	-99,00000000	-99,00000000	-99,00000000	-99,00000000	-99,00000000	-99,00000000	-99,00000000	-99,00000000	-99,00000000	-99,00000000	-99,00000000	-99,00000000	265	-99,00000000	0,30939033

Location_ Treatment	JKI Shelter_Ds [68015]										
Ranking	27	47	38	23	25	54	45	1	66	22	35
Gene product	Glutamyl-tRNA(Gln)_amidotransferase_subunit_A	(S)-norcoclaurine synthase	Cytochrome_ P450	PR1_protein	Beta-D-glucan_ exohydrolase	SBT4B_protein	Ethylene-inducing_ xylanase	Glucosyltransferase	Nbs-lrr_resistance_ protein	Acidic_class_II _1_3-beta-glucanase	Hcr9-OR2A
Transcript ID F1-Individuals	PGSC0003 DMT400008092	PGSC0003 DMT400008278	PGSC0003 DMT400008547	PGSC0003 DMT400013094	PGSC0003 DMT400015224	PGSC0003 DMT400017633	PGSC0003 DMT400020366	PGSC0003 DMT400021019	PGSC0003 DMT400021608	PGSC0003 DMT40002720	PGSC0003 DMT40002779
1	0,03836674	0,08777780	0,60248616	0,01256017	0,05831456	1,98068574	0,02580868	0,05675928	0,00888754	0,01254277	0,00172762
2	-99	-99	-99	-99	-99	-99	-99	-99	-99	-99	-99
3	-99	-99	-99	-99	-99	-99	-99	-99	-99	-99	-99
4	-99	-99	-99	-99	-99	-99	-99	-99	-99	-99	-99
5	-99	-99	-99	-99	-99	-99	-99	-99	-99	-99	-99
6	0,26261114	0,14141431	0,92466328	0,01042362	0,09106244	2,23302592	0,05908763	0,05904669	0,02812506	0,03855335	0,00247561
7	-99	-99	-99	-99	-99	-99	-99	-99	-99	-99	-99
8	-99	-99	-99	-99	-99	-99	-99	-99	-99	-99	-99
9	-99	-99	-99	-99	-99	-99	-99	-99	-99	-99	-99
10	-99	-99	-99	-99	-99	-99	-99	-99	-99	-99	-99
11	-99	-99	-99	-99	-99	-99	-99	-99	-99	-99	-99
12	-99	-99	-99	-99	-99	-99	-99	-99	-99	-99	-99
13	-99	-99	-99	-99	-99	-99	-99	-99	-99	-99	-99
14	-99	-99	-99	-99	-99	-99	-99	-99	-99	-99	-99
15	-99	-99	-99	-99	-99	-99	-99	-99	-99	-99	-99
16	0,06782684	0,25278802	0,57315609	0,01061317	0,02653426	1,55078641	0,02221996	0,22877479	0,01797331	0,05695633	0,00041546
17	0,20479466	0,12109826	0,57285821	0,01808892	0,04806898	6,35657662	0,00212879	0,09802204	0,00344627	0,02046426	0,00183914
18	0,10433174	0,12321502	0,98230980	0,04786948	0,01430090	3,29493499	0,03470410	0,09870384	0,02319970	0,00908215	0,00115351
19	0,05431521	0,11411023	0,66595657	0,00427014	0,05353032	1,70941182	0,00002661	0,11308661	0,01298959	0,00461485	0,00020156
20	-99	-99	-99	-99	-99	-99	-99	-99	-99	-99	-99
21	-99	-99	-99	-99	-99	-99	-99	-99	-99	-99	-99
22	-99	-99	-99	-99	-99	-99	-99	-99	-99	-99	-99
23	-99	-99	-99	-99	-99	-99	-99	-99	-99	-99	-99
24	0,25818910	0,12767076	0,38971733	0,01667118	0,02374060	1,62957980	0,01871709	0,08102439	0,01675227	0,00401746	0,00177557
25	-99	-99	-99	-99	-99	-99	-99	-99	-99	-99	-99
26	-99	-99	-99	-99	-99	-99	-99	-99	-99	-99	-99
27	-99	-99	-99	-99	-99	-99	-99	-99	-99	-99	-99
28	-99	-99	-99	-99	-99	-99	-99	-99	-99	-99	-99
29	-99	-99	-99	-99	-99	-99	-99	-99	-99	-99	-99
30	-99	-99	-99	-99	-99	-99	-99	-99	-99	-99	-99
31	-99	-99	-99	-99	-99	-99	-99	-99	-99	-99	-99
32	-99	-99	-99	-99	-99	-99	-99	-99	-99	-99	-99
33	0,09792018	0,13237935	0,21790180	0,00877582	0,01606150	2,56996729	0,00001546	0,07597929	0,01357174	0,00594441	0,00159277
34	-99	-99	-99	-99	-99	-99	-99	-99	-99	-99	-99
35	0,26471283	0,11253924	0,49706279	0,01067589	0,03119589	1,42059962	0,00024832	0,07450598	0,01953899	0,02868619	0,00087314
36	-99	-99	-99	-99	-99	-99	-99	-99	-99	-99	-99
37	-99	-99	-99	-99	-99	-99	-99	-99	-99	-99	-99
38	-99	-99	-99	-99	-99	-99	-99	-99	-99	-99	-99
39	-99	-99	-99	-99	-99	-99	-99	-99	-99	-99	-99
40	0,28977399	0,07314992	0,66388258	0,01532467	0,04010706	2,13465068	0,01231022	0,08310044	0,01275317	0,00638101	0,00053469
41	-99	-99	-99	-99	-99	-99	-99	-99	-99	-99	-99
42	-99	-99	-99	-99	-99	-99	-99	-99	-99	-99	-99
43	0,21941742	0,12454595	1,10860852	0,01136507	0,04032309	1,56238500	0,02546660	0,07203041	0,01774738	0,00489237	0,00331161
44	-99	-99	-99	-99	-99	-99	-99	-99	-99	-99	-99
45	-99	-99	-99	-99	-99	-99	-99	-99	-99	-99	-99
46	0,07926073	0,07313724	0,78553638	0,00607774	0,08285600	1,69202396	0,00261994	0,10264605	0,01357174	0,06628150	0,00266204
47	-99	-99	-99	-99	-99	-99	-99	-99	-99	-99	-99
48	-99	-99	-99	-99	-99	-99	-99	-99	-99	-99	-99
49	0,13130557	0,36984688	0,26061644	0,02631447	0,03377267	2,11403608	0,00002061	0,24114955	0,01420950	0,00613381	0,00113822
50	0,28628024	0,16893030	0,78105714	0,00429389	0,01072038	2,10307456	0,00001563	0,05420238	0,02365846	0,01187030	0,00337827
51	0,08254073	0,12641591	0,44265358	0,01148783	0,02216228	0,59141798	0,01413338	0,18521207	0,01262345	0,00477015	0,00016312
52	-99	-99	-99	-99	-99	-99	-99	-99	-99	-99	-99
53	-99	-99	-99	-99	-99	-99	-99	-99	-99	-99	-99
54	-99	-99	-99	-99	-99	-99	-99	-99	-99	-99	-99
55	-99	-99	-99	-99	-99	-99	-99	-99	-99	-99	-99
56	-99	-99	-99	-99	-99	-99	-99	-99	-99	-99	-99
57	0,25423772	0,10119781	0,57964841	0,01610051	0,10876226	0,82259260	0,05227446	0,31343756	0,01594502	0,01771665	0,00023133
58	0,37860097	0,16849177	1,47913117	0,01370409	0,05920037	1,59300412	0,02813969	0,60113045	0,02119327	0,01886362	0,00325247
59	-99	-99	-99	-99	-99	-99	-99	-99	-99	-99	-99
60	-99	-99	-99	-99	-99	-99	-99	-99	-99	-99	-99
61	0,05714416	0,12867021	1,18735390	0,03183483	0,03309484	2,02055273	0,04174508	0,25256909	0,01936708	0,15472197	0,00228712
62	-99	-99	-99	-99	-99	-99	-99	-99	-99	-99	-99
63	-99	-99	-99	-99	-99	-99	-99	-99	-99	-99	-99
64	-99	-99	-99	-99	-99	-99	-99	-99	-99	-99	-99
65	0,16536777	0,22094359	0,44158100	0,00666467	0,05895467	0,88132742	0,01551438	0,06266267	0,01350605	0,01622936	0,00289896
66	-99	-99	-99	-99	-99	-99	-99	-99	-99	-99	-99
67	-99	-99	-99	-99	-99	-99	-99	-99	-99	-99	-99
68	-99	-99	-99	-99	-99	-99	-99	-99	-99	-99	-99
69	-99	-99	-99	-99	-99	-99	-99	-99	-99	-99	-99
70	-99	-99	-99	-99	-99	-99	-99	-99	-99	-99	-99

Location_ Treatment	JKI Shelter_Ds [68015]										
Ranking	17	57	21	6	8	3	24	33	15	4	50
Gene product	Serine-threonine protein kinase, plant-type	Gamma_ aminobutyrate _transaminase _isoform1	O-methyltransferase	Multidrug_resistance _protein_ABC_transporter_family	BED_finger-nbs-lrr_ resistance_ protein	S-locus-specific_glycoprotein_S6	NADPH-dependent_ codeinone_ reductase	Desacetoxyvindoline_4-hydroxylase	Kinase	Receptor_ protein_ kinase	Reticuline_ oxidase
Transcript ID F1-Individuals	PGSC0003 DMT40002843	PGSC0003 DMT40003068	PGSC0003 DMT400031370	PGSC0003 DMT400031714	PGSC0003 DMT400035714	PGSC0003 DMT40003602	PGSC0003 DMT40003748	PGSC0003 DMT400041989	PGSC0003 DMT40004236	PGSC0003 DMT40004568	PGSC0003 DMT40004630
1	0,03481855	3,57266341	0,51978972	0,15410654	0,01917008	0,20632635	0,96259444	0,24861754	0,26407141	0,00082119	2,59008600
2	-99	-99	-99	-99	-99	-99	-99	-99	-99	-99	-99
3	-99	-99	-99	-99	-99	-99	-99	-99	-99	-99	-99
4	-99	-99	-99	-99	-99	-99	-99	-99	-99	-99	-99
5	-99	-99	-99	-99	-99	-99	-99	-99	-99	-99	-99
6	0,19737347	4,16986304	0,57038186	0,04575268	0,02927880	0,08161624	0,66434291	0,61132014	0,20518540	0,01655029	3,93946577
7	-99	-99	-99	-99	-99	-99	-99	-99	-99	-99	-99
8	-99	-99	-99	-99	-99	-99	-99	-99	-99	-99	-99
9	-99	-99	-99	-99	-99	-99	-99	-99	-99	-99	-99
10	-99	-99	-99	-99	-99	-99	-99	-99	-99	-99	-99
11	-99	-99	-99	-99	-99	-99	-99	-99	-99	-99	-99
12	-99	-99	-99	-99	-99	-99	-99	-99	-99	-99	-99
13	-99	-99	-99	-99	-99	-99	-99	-99	-99	-99	-99
14	-99	-99	-99	-99	-99	-99	-99	-99	-99	-99	-99
15	-99	-99	-99	-99	-99	-99	-99	-99	-99	-99	-99
16	0,42750109	4,55042091	0,16098520	0,21538654	0,02270258	0,13985464	0,74123350	0,99792272	0,26535580	0,02791143	2,26419780
17	0,26211104	3,58568810	0,16451032	0,06625853	0,01123583	0,19947087	0,70821043	0,67607256	0,33338244	0,02256530	2,00034660
18	0,23040597	6,26906375	0,02256530	0,27286325	0,01624624	0,11005145	1,02331478	0,68836708	0,30066941	0,00041640	3,68138848
19	0,10788005	3,36009029	0,35613595	0,26215646	0,00397866	0,28390890	0,19730508	0,31589128	0,18498754	0,00033385	4,22367843
20	-99	-99	-99	-99	-99	-99	-99	-99	-99	-99	-99
21	-99	-99	-99	-99	-99	-99	-99	-99	-99	-99	-99
22	-99	-99	-99	-99	-99	-99	-99	-99	-99	-99	-99
23	-99	-99	-99	-99	-99	-99	-99	-99	-99	-99	-99
24	0,15288301	4,55515454	0,08720170	0,06365863	0,01750002	0,10065565	0,62092891	0,49878846	0,31111072	0,00241544	2,01460919
25	-99	-99	-99	-99	-99	-99	-99	-99	-99	-99	-99
26	-99	-99	-99	-99	-99	-99	-99	-99	-99	-99	-99
27	-99	-99	-99	-99	-99	-99	-99	-99	-99	-99	-99
28	-99	-99	-99	-99	-99	-99	-99	-99	-99	-99	-99
29	-99	-99	-99	-99	-99	-99	-99	-99	-99	-99	-99
30	-99	-99	-99	-99	-99	-99	-99	-99	-99	-99	-99
31	-99	-99	-99	-99	-99	-99	-99	-99	-99	-99	-99
32	-99	-99	-99	-99	-99	-99	-99	-99	-99	-99	-99
33	0,12038687	5,35820582	0,09478155	0,05527420	0,00360884	0,03137480	0,64115719	1,86315797	0,20009403	0,00156540	3,28695111
34	-99	-99	-99	-99	-99	-99	-99	-99	-99	-99	-99
35	0,18966166	4,17999148	0,17843864	0,09844761	0,01722324	0,17319870	0,92370239	0,61195607	0,26933992	0,01737913	2,91500832
36	-99	-99	-99	-99	-99	-99	-99	-99	-99	-99	-99
37	-99	-99	-99	-99	-99	-99	-99	-99	-99	-99	-99
38	-99	-99	-99	-99	-99	-99	-99	-99	-99	-99	-99
39	-99	-99	-99	-99	-99	-99	-99	-99	-99	-99	-99
40	0,17158555	3,47978941	0,36729216	0,20949678	0,03755113	0,07609788	0,79498525	0,34340906	0,25720668	0,00974582	0,93692145
41	-99	-99	-99	-99	-99	-99	-99	-99	-99	-99	-99
42	-99	-99	-99	-99	-99	-99	-99	-99	-99	-99	-99
43	0,13647892	4,15471639	0,42742702	0,10207843	0,00897887	0,06491744	0,27182499	0,51646769	0,35619767	0,00377777	3,06258524
44	-99	-99	-99	-99	-99	-99	-99	-99	-99	-99	-99
45	-99	-99	-99	-99	-99	-99	-99	-99	-99	-99	-99
46	0,21004204	2,89939120	0,26789694	0,08613539	0,01349670	0,13516092	0,75983075	0,60909957	0,19747610	0,01180670	1,50081900
47	-99	-99	-99	-99	-99	-99	-99	-99	-99	-99	-99
48	-99	-99	-99	-99	-99	-99	-99	-99	-99	-99	-99
49	0,28361387	5,00718583	0,02849791	0,18111777	0,03629715	0,03328463	1,24142749	1,39958587	0,24587552	0,00157329	3,28069164
50	0,18874365	4,81488053	0,03401936	0,21726083	0,02323591	0,19716837	1,41274394	0,78648982	0,27710411	0,01770745	2,81181173
51	0,12067929	4,46992306	0,01690097	0,08236927	0,03274118	0,22209518	0,19495999	0,82487648	0,28642910	0,00584837	2,38543107
52	-99	-99	-99	-99	-99	-99	-99	-99	-99	-99	-99
53	-99	-99	-99	-99	-99	-99	-99	-99	-99	-99	-99
54	-99	-99	-99	-99	-99	-99	-99	-99	-99	-99	-99
55	-99	-99	-99	-99	-99	-99	-99	-99	-99	-99	-99
56	-99	-99	-99	-99	-99	-99	-99	-99	-99	-99	-99
57	0,04585586	3,42534665	0,64628840	0,09196636	0,00645776	0,07956347	0,04636725	0,59100818	0,15682745	0,00193460	2,16632545
58	0,14729190	5,12215177	0,23000705	0,13173860	0,04151424	0,14607184	0,52150392	0,59450053	0,21490188	0,00741031	2,02335575
59	-99	-99	-99	-99	-99	-99	-99	-99	-99	-99	-99
60	-99	-99	-99	-99	-99	-99	-99	-99	-99	-99	-99
61	0,09914955	2,48586853	0,15090892	0,06491744	0,00907901	0,05631839	0,33142400	0,44929970	0,12637211	0,00263816	2,29699864
62	-99	-99	-99	-99	-99	-99	-99	-99	-99	-99	-99
63	-99	-99	-99	-99	-99	-99	-99	-99	-99	-99	-99
64	-99	-99	-99	-99	-99	-99	-99	-99	-99	-99	-99
65	0,13046638	3,36533471	0,45525602	0,14406083	0,01847541	0,29376817	1,21651153	0,56087454	0,28474650	0,02052819	4,25084602
66	-99	-99	-99	-99	-99	-99	-99	-99	-99	-99	-99
67	-99	-99	-99	-99	-99	-99	-99	-99	-99	-99	-99
68	-99	-99	-99	-99	-99	-99	-99	-99	-99	-99	-99
69	-99	-99	-99	-99	-99	-99	-99	-99	-99	-99	-99
70	-99	-99	-99	-99	-99	-99	-99	-99	-99	-99	-99

-99	-99	-99	-99	-99	-99	-99	-99	-99	-99	-99	-99	-99	-99	-99	-99	-99	-99	-99	71	-99	-99	-99
-99	-99	-99	-99	-99	-99	-99	-99	-99	-99	-99	-99	-99	-99	-99	-99	-99	-99	-99	72	-99	-99	-99
-99	-99	-99	-99	-99	-99	-99	-99	-99	-99	-99	-99	-99	-99	-99	-99	-99	-99	-99	73	-99	-99	-99
-99	-99	-99	-99	-99	-99	-99	-99	-99	-99	-99	-99	-99	-99	-99	-99	-99	-99	-99	74	-99	-99	-99
0,31808847	0,08946703	0,68278345	0,01060215	0,03270149	1,81944697	0,01956610	0,13383246	0,00217239	0,09695775	0,00022551	0,04640744	5,43582873	0,07724043	0,09404532	0,03117428	0,09749689	0,79968293	0,49775234	75	0,24223849	0,00092356	1,88230446
-99	-99	-99	-99	-99	-99	-99	-99	-99	-99	-99	-99	-99	-99	-99	-99	-99	-99	-99	76	-99	-99	-99
-99	-99	-99	-99	-99	-99	-99	-99	-99	-99	-99	-99	-99	-99	-99	-99	-99	-99	-99	77	-99	-99	-99
-99	-99	-99	-99	-99	-99	-99	-99	-99	-99	-99	-99	-99	-99	-99	-99	-99	-99	-99	78	-99	-99	-99
-99	-99	-99	-99	-99	-99	-99	-99	-99	-99	-99	-99	-99	-99	-99	-99	-99	-99	-99	79	-99	-99	-99
-99	-99	-99	-99	-99	-99	-99	-99	-99	-99	-99	-99	-99	-99	-99	-99	-99	-99	-99	80	-99	-99	-99
-99	-99	-99	-99	-99	-99	-99	-99	-99	-99	-99	-99	-99	-99	-99	-99	-99	-99	-99	81	-99	-99	-99
-99	-99	-99	-99	-99	-99	-99	-99	-99	-99	-99	-99	-99	-99	-99	-99	-99	-99	-99	82	-99	-99	-99
-99	-99	-99	-99	-99	-99	-99	-99	-99	-99	-99	-99	-99	-99	-99	-99	-99	-99	-99	83	-99	-99	-99
0,06529992	0,12153342	0,49043199	0,00149382	0,08738473	1,19277754	0,00332029	0,08426194	0,00976628	0,00373371	0,00227444	0,26365532	2,51215416	0,43218623	0,10023964	0,00733049	0,02915779	0,80291824	0,94162531	84	0,13599495	0,00322189	2,22950742
-99	-99	-99	-99	-99	-99	-99	-99	-99	-99	-99	-99	-99	-99	-99	-99	-99	-99	-99	85	-99	-99	-99
-99	-99	-99	-99	-99	-99	-99	-99	-99	-99	-99	-99	-99	-99	-99	-99	-99	-99	-99	86	-99	-99	-99
0,05650021	0,09825673	0,23436028	0,00361197	0,02887170	3,20028414	0,01381137	0,19960227	0,00812228	0,01291955	0,00038716	0,13096017	3,49962418	0,09077256	0,06940580	0,01282409	0,08605483	0,18165840	0,62585485	87	0,11073629	0,00900474	1,50326567
0,15206394	0,05609243	0,36061967	0,00151912	0,05393816	0,93325937	0,01578666	0,19684746	0,00616771	0,02347144	0,00165615	0,10606716	2,86125881	0,04042664	0,20769667	0,00927427	0,00907083	0,19801066	0,52728322	88	0,15750287	0,00517240	2,47383624
0,13758308	0,06325288	0,42032437	0,00940455	0,01185201	1,14019059	0,02247827	0,07148454	0,03213468	0,01498409	0,00203251	0,15167708	2,94297441	0,26055774	0,10070275	0,01223939	0,08290914	0,33007711	0,37714076	89	0,13658538	0,00946275	1,49980506
-99	-99	-99	-99	-99	-99	-99	-99	-99	-99	-99	-99	-99	-99	-99	-99	-99	-99	-99	90	-99	-99	-99
-99	-99	-99	-99	-99	-99	-99	-99	-99	-99	-99	-99	-99	-99	-99	-99	-99	-99	-99	91	-99	-99	-99
-99	-99	-99	-99	-99	-99	-99	-99	-99	-99	-99	-99	-99	-99	-99	-99	-99	-99	-99	92	-99	-99	-99
-99	-99	-99	-99	-99	-99	-99	-99	-99	-99	-99	-99	-99	-99	-99	-99	-99	-99	-99	93	-99	-99	-99
-99	-99	-99	-99	-99	-99	-99	-99	-99	-99	-99	-99	-99	-99	-99	-99	-99	-99	-99	94	-99	-99	-99
-99	-99	-99	-99	-99	-99	-99	-99	-99	-99	-99	-99	-99	-99	-99	-99	-99	-99	-99	95	-99	-99	-99
-99	-99	-99	-99	-99	-99	-99	-99	-99	-99	-99	-99	-99	-99	-99	-99	-99	-99	-99	96	-99	-99	-99
-99	-99	-99	-99	-99	-99	-99	-99	-99	-99	-99	-99	-99	-99	-99	-99	-99	-99	-99	97	-99	-99	-99
-99	-99	-99	-99	-99	-99	-99	-99	-99	-99	-99	-99	-99	-99	-99	-99	-99	-99	-99	98	-99	-99	-99
0,11142347	0,11417550	0,52001495	0,02096352	0,03537135	1,81494396	0,00002384	0,20445071	0,01303040	0,01720921	0,00081113	0,22680510	5,11390372	0,37026369	0,21894266	0,01308470	0,08273405	1,00634501	0,36712035	99	0,26508464	0,00693122	2,85823592
0,21419549	0,12913930	0,70612720	0,00412040	0,01116402	1,26312726	0,00002997	0,40640780	0,01808578	0,01966808	0,00662897	0,13203570	4,01110574	0,01214916	0,15103973	0,02317961	0,27452320	0,61047326	0,53144784	100	0,44043469	0,02762274	2,41831135
-99	-99	-99	-99	-99	-99	-99	-99	-99	-99	-99	-99	-99	-99	-99	-99	-99	-99	-99	101	-99	-99	-99
-99	-99	-99	-99	-99	-99	-99	-99	-99	-99	-99	-99	-99	-99	-99	-99	-99	-99	-99	102	-99	-99	-99
0,23072560	0,14124288	0,70185748	0,01086062	0,06088582	1,89375540	0,01831602	0,05393068	0,02211624	0,04395740	0,00025846	0,32336826	3,50703055	0,19523045	0,09788625	0,02593870	0,29022627	0,79237211	0,92996608	103	0,33710034	0,01525314	2,52144368
0,28882149	0,25213180	1,16332161	0,00952048	0,06521059	2,18593424	0,02882072	0,12528191	0,02329639	0,02219302	0,00039476	0,22271181	6,40966964	0,06498497	0,10733928	0,01158378	0,17558603	0,35879955	0,72610495	104	0,35484232	0,00932455	3,16290451
-99	-99	-99	-99	-99	-99	-99	-99	-99	-99	-99	-99	-99	-99	-99	-99	-99	-99	-99	105	-99	-99	-99
0,08211275	0,17921335	0,65146068	0,01521618	0,01406009	2,80840305	0,03720783	0,25503197	0,01673196	0,00914849	0,00242256	0,13953994	5,75074952	0,44588699	0,19368042	0,01752733	0,29315793	1,06344838	0,51041761	106	0,30370657	0,02090150	3,70827953
0,27907988	0,18348703	0,51112569	0,00386250	0,00641093	0,87706155	0,00004998	0,18734248	0,03245872	0,08143258	0,00282360	0,23435216	4,95453616	0,03751861	0,20401541	0,02069965	0,13761885	0,42183479	0,35398251	107	0,26826858	0,01789562	2,49969140
-99	-99	-99	-99	-99	-99	-99	-99	-99	-99	-99	-99	-99	-99	-99	-99	-99	-99	-99	108	-99	-99	-99
-99	-99	-99	-99	-99	-99	-99	-99	-99	-99	-99	-99	-99	-99	-99	-99	-99	-99	-99	109	-99	-99	-99
0,26655405	0,17367957	2,31899547	0,16904743	0,05960181	4,73216376	0,00004734	0,12635021	0,01955254	0,32252882	0,00563449	0,30260338	6,29628131	0,46894805	0,20869964	0,02689534	0,43997700	0,30239370	0,74303393	110	0,39025797	0,16306285	4,11103029
-99	-99	-99	-99	-99	-99	-99	-99	-99	-99	-99	-99	-99	-99	-99	-99	-99	-99	-99	111	-99	-99	-99
-99	-99	-99	-99	-99	-99	-99	-99	-99	-99	-99	-99	-99	-99	-99	-99	-99	-99	-99	112	-99	-99	-99
-99	-99	-99	-99	-99	-99	-99	-99	-99	-99	-99	-99	-99	-99	-99	-99	-99	-99	-99	113	-99	-99	-99
0,38729372	0,15882391	1,62394188	0,03970598	0,13692901	1,69760405	0,06273872	0,11903849	0,02799863	0,28987443	0,00253904	0,19311070	4,44597551	0,37722573	0,09729436	0,03628457	0,17488761	0,47648411	0,79636405	114	0,34904864	0,02278929	2,54294454
-99	-99	-99	-99	-99	-99	-99	-99	-99	-99	-99	-99	-99	-99	-99	-99	-99	-99	-99	115	-99	-99	-99
-99	-99	-99	-99	-99	-99	-99	-99	-99	-99	-99	-99	-99	-99	-99	-99	-99	-99	-99	116	-99	-99	-99
-99	-99	-99	-99	-99	-99	-99	-99	-99	-99	-99	-99	-99	-99	-99	-99	-99	-99	-99	117	-99	-99	-99
-99	-99	-99	-99	-99	-99	-99	-99	-99	-99	-99	-99	-99	-99	-99	-99	-99	-99	-99	118	-99	-99	-99
-99	-99	-99	-99	-99	-99	-99	-99	-99	-99	-99	-99	-99	-99	-99	-99	-99	-99	-99	119	-99	-99	-99
0,04054028	0,13826424	0,46894805	0,00326319	0,04363108	1,18222120	0,03376097	0,13951576	0,00467279	0,00025040	0,00343494	0,09384996	4,33640649	0,19757879	0,08848029	0,02648832	0,18118055	0,23188799	0,51068302	120	0,34210237	0,00398418	1,41961527
0,12300169	0,19775005	0,94589372	0,06331758	0,04385089	1,16857473	0,07079297	0,10834842	0,02468451	0,13450671	0,00015036	0,04872311	5,68338323	0,22652623	0,28180127	0,02209326	0,15375977	0,39304053	0,64160176	121	0,36041976	0,01839873	1,67220300
0,26701635	0,04152143	0,93303299	0,06363657	0,04049816	1,16715810	0,00604099	0,12674493	0,00656495	0,42103148	0,00326206	0,13103281	5,19815625	0,14670602	0,18880907	0,02729915	0,17726750	0,00001380	0,68968046	122	0,22845786	0,00796561	3,13616182
-99	-99	-99	-99	-99	-99	-99	-99	-99	-99	-99	-99	-99	-99	-99	-99	-99	-99	-99	123	-99	-99	-99
-99	-99	-99	-99	-99	-99	-99	-99	-99	-99	-99	-99	-99	-99	-99	-99	-99	-99	-99	124	-99	-99	-99
-99	-99	-99	-99	-99	-99	-99	-99	-99	-99	-99	-99	-99	-99	-99	-99	-99	-99	-99	125	-99	-99	-99
-99	-99	-99	-99	-99	-99	-99	-99	-99	-99	-99	-99	-99	-99	-99	-99	-99	-99	-99	126	-99	-99	-99
0,05302255	0,16084578	0,37312993	0,01310944	0,02937535	1,94767100	0,03908476	0,21033342	0,00514630	0,03759670	0,00299288	0,06154349	5,44054055	0,42037536	0,12420111	0,01432571	0,02964125	0,12072112	0,86020356	127	0,23402750	0,01159583	2,05160074
-99	-99	-99	-99	-99	-99	-99	-99	-99	-99	-99	-99	-99	-99	-99	-99	-99	-99	-99	128	-99	-99	-99
-99	-99	-99	-99	-99	-99	-99	-99	-99	-99	-99	-99	-99	-99	-99	-99	-99	-99	-99	129	-99	-99	-99
-99	-99	-99	-99	-99	-99	-99	-99	-99	-99	-99	-99	-99	-99	-99	-99	-99	-99	-99	130	-99	-99	-99
-99	-99	-99	-99	-99	-99	-99	-99	-99	-99	-99	-99	-99	-99	-99	-99	-99	-99	-99	131	-99	-99	-99
-99	-99	-99	-99	-99	-99	-99	-99	-99	-99	-99	-99	-99	-99	-99	-99	-99	-99	-99	132	-99	-99	-99
-99	-99	-99	-99	-99	-99	-99	-99	-99	-99	-99	-99	-99	-99	-99	-99	-99	-99	-99	133	-99	-99	-99
-99	-99	-99	-99	-99	-99	-99	-99	-99	-99	-99	-99	-99	-99	-99	-99	-99	-99	-99	134	-99	-99	-99
-99	-99	-99	-99	-99	-99	-99	-99	-99	-99	-99	-99	-99	-99	-99	-99	-99	-99	-99	135	-99	-99	-99
-99	-99	-99	-99	-99	-99	-99	-99	-99	-99	-99	-99	-99	-99	-99	-99	-99	-99	-99	136	-99	-99	-99
-99	-99	-99	-99	-99	-99	-99	-99	-99	-99	-99	-99	-99	-99	-99	-99	-99	-99	-99	137	-99	-99	-99
0,33338244	0,15211665	1,31334869	0,02236288	0,21275280	1,30337299	0,00003010	0,28822153	0,01926666	0,03834680	0,00262085	0,32720121	7,25135196	0,10733928	0,26356852	0,05080978	0,28111844	0,28702534	0,69363567	138	0,36965466	0,06695103	3,10641375
-99	-99	-99	-99	-99	-99	-99	-99	-99	-99	-99	-99	-99	-99	-99	-99	-99	-99	-99	139	-99	-99	-99
0,10067309	0,12813620	0,50935733	0,01888978	0,35619767	2,69400028	0,00328874	0,10589820	0,02041467	0,03152740	0,00231744	0,14070544	4,21271407	0,20009403	0,15069986	0,04035105	0,20444008	1,16453177	0,35105038	140	0,33027736	0,01188059	4,48388725
0,11081691	0,07642826	0,52060100	0,00303151	0,00857439	1,57488884	0,00002184	0,01228677	0,02751286	0,02886070	0,00227369	0,03432135	4,35976387	0,12773714	0,09350906	0,01151972	0,03693801	0,52458556	0,54120610	141	0,29673562	0,01458117	2,65783216
0,08285600	0,03512763	0,62796236	0,01062606	0,06437971	2,23883778	0,02247944	0,04363864	0,00638433	0,04542877	0,00494693	0,04722681	5,92062714	0,07203041	0,16162811	0,02304743	0,29807556	0,44526928	0,52915049	142	0,38255789	0,00218788	3,69033043
0,05843595	0,12225793	1,17446197	0,00988187	0,05108346	0,67830216	0,03346971	0,27854841	0,01235295	0,00773169	0,00466793	0,23148651	4,08121864	0,16887176	0,12824727	0,01616481	0,23602310	0,73407532	0,87236271	143	0,31709785	0,02679300	2,41161566
-99	-99	-99	-99	-99	-99	-99	-99	-99	-99	-99	-99	-99	-99	-99	-99	-99	-99	-99	144	-99	-99	-99
0,10891315	0,12676690	0,86648699	0,00951388	0,26066161	3,23832998	0,03008629	0,41101131	0,02291204	0,06369174	0,00157247	0,07145853	4,46373073	0,21423261	0,27898317	0,02179665	0,25125949	0,81239316	0,97214943	145	0,49835648	0,01506401	2,48673022
-99	-99	-99	-99	-99	-99	-99	-99	-99	-99	-99	-99	-99	-99	-99	-99	-99	-99	-99	146	-99	-99	-99
0,34741935	0,06781508	0,15269767	0,04148547	0,54933191	4,15759722	0,08583739	0,18463526	0,02652966	0,19733927	0,00120208	0,03386057	2,19732763	1,46180412	0,22186438	0,02400951	0,17383012	0,56243177	0,80874119	147	0,05189538	0,00761337	3,43604755
-99	-99	-99	-99	-99	-99	-99	-99	-99	-99	-99	-99	-99	-99	-99	-99	-99	-99	-99	148	-99	-99	-99
-99	-99	-99	-99	-99	-99	-99	-99	-99	-99	-99	-99	-99	-99	-99	-99	-99	-99	-99	149	-99	-99	-99
-99	-99	-99	-99	-99	-99	-99	-99	-99	-99	-99	-99	-99	-99	-99	-99	-99	-99	-99	150	-99	-99	-99
-99	-99	-99	-99	-99	-99	-99	-99	-99	-99	-99	-99	-99	-99	-99	-99	-99	-99	-99	151	-99	-99	-99
0,06620115	0,12922884	0,29219431	0,11574325	0,07085434	2,37841423	0,03685489	0,21095395	0,02140364	0,06886907	0,00003142	0,00955188	4,93825043	0,02826186	0,10942392	0,00901161	0,05161735	0,31425334	0,76154437	152	0,26962011	0,00899912	3,38228915
-99	-99	-99	-99	-99	-99	-99	-99	-99	-99	-99	-99	-99	-99	-99	-99	-99	-99	-99	153	-99	-99	-99

-99	-99	-99	-99	-99	-99	-99	-99	-99	-99	-99	-99	-99	-99	-99	-99	-99	-99	-99	154	-99	-99	-99
-99	-99	-99	-99	-99	-99	-99	-99	-99	-99	-99	-99	-99	-99	-99	-99	-99	-99	-99	155	-99	-99	-99
-99	-99	-99	-99	-99	-99	-99	-99	-99	-99	-99	-99	-99	-99	-99	-99	-99	-99	-99	156	-99	-99	-99
-99	-99	-99	-99	-99	-99	-99	-99	-99	-99	-99	-99	-99	-99	-99	-99	-99	-99	-99	157	-99	-99	-99
-99	-99	-99	-99	-99	-99	-99	-99	-99	-99	-99	-99	-99	-99	-99	-99	-99	-99	-99	158	-99	-99	-99
0,15030866	0,15346696	0,60457784	0,05388397	0,05399614	2,09216988	0,01036598	0,19999003	0,02332466	0,03073444	0,00269033	0,01234440	4,66216479	0,11041438	0,11056755	0,01487465	0,00002722	0,27452320	1,00277644	159	0,36933452	0,03153286	3,56030293
0,29463485	0,11049094	0,76207242	0,04886685	0,04515407	2,92614244	0,00004072	0,04143518	0,01868468	0,04478005	0,00167456	0,03284915	4,75353242	0,52376806	0,05395872	0,01678132	0,05186841	0,27074376	0,64037993	160	0,29750792	0,01745761	1,98480875
0,14284278	0,31392678	0,34808221	0,03878117	0,01322581	2,95160569	0,03308910	0,26600034	0,00252500	0,07968765	0,00238053	0,01561959	6,91869527	0,03512154	0,20243070	0,00838775	0,00743475	0,18731002	1,07661319	161	0,32589972	0,00747091	4,54569220
-99	-99	-99	-99	-99	-99	-99	-99	-99	-99	-99	-99	-99	-99	-99	-99	-99	-99	-99	162	-99	-99	-99
-99	-99	-99	-99	-99	-99	-99	-99	-99	-99	-99	-99	-99	-99	-99	-99	-99	-99	-99	163	-99	-99	-99
0,18608066	0,22940997	0,50662845	0,00232106	0,19833346	0,09909802	0,02217380	0,49106980	0,00752548	0,00875911	0,00242803	0,10904534	4,26634312	0,21449263	0,33541056	0,02923823	0,15636614	0,52558645	0,54073738	164	0,50000000	0,00462606	5,90525762
-99	-99	-99	-99	-99	-99	-99	-99	-99	-99	-99	-99	-99	-99	-99	-99	-99	-99	-99	165	-99	-99	-99
-99	-99	-99	-99	-99	-99	-99	-99	-99	-99	-99	-99	-99	-99	-99	-99	-99	-99	-99	166	-99	-99	-99
0,30645561	0,11287125	0,51503772	0,01956949	0,01627442	3,01205894	0,04327717	0,11070176	0,01191357	0,01833508	0,00036794	0,25877139	2,37306234	1,91819581	0,24278480	0,01688341	0,18982606	0,50408887	0,51148009	167	0,19381471	0,00058542	3,72889972
-99	-99	-99	-99	-99	-99	-99	-99	-99	-99	-99	-99	-99	-99	-99	-99	-99	-99	-99	168	-99	-99	-99
-99	-99	-99	-99	-99	-99	-99	-99	-99	-99	-99	-99	-99	-99	-99	-99	-99	-99	-99	169	-99	-99	-99
-99	-99	-99	-99	-99	-99	-99	-99	-99	-99	-99	-99	-99	-99	-99	-99	-99	-99	-99	170	-99	-99	-99
0,25801020	0,19867744	0,67759729	0,01061685	0,06093859	1,67539352	0,01318919	0,03907799	0,02884570	0,00422891	0,00056264	0,22523454	4,33941330	0,26197481	0,44488365	0,02186095	0,07523251	1,40104180	1,36462069	171	0,33903356	0,00253904	2,48888574
0,16286517	0,13733298	0,55777300	0,00892922	0,01906077	1,53767435	0,00164665	0,00508601	0,02187990	0,01603924	0,00147175	0,10429559	6,74935669	0,01752733	0,02571493	0,01860391	0,03930209	1,07307432	0,63101666	172	0,39276819	0,00540215	2,27797198
-99	-99	-99	-99	-99	-99	-99	-99	-99	-99	-99	-99	-99	-99	-99	-99	-99	-99	-99	173	-99	-99	-99
0,20646942	0,36123260	0,35923504	0,03305472	0,04846208	0,85856544	0,00580696	0,12327909	0,04038603	0,02338942	0,00470366	0,45470413	3,57018789	0,71697762	0,38421880	0,04540516	0,02981124	0,54412122	0,80552429	174	0,32646495	0,01414073	0,85975649
0,17343897	0,10580649	0,75969909	0,00497013	0,00865950	1,17649892	0,02212007	0,06387964	0,02247555	0,00780979	0,00076434	0,05894446	4,69296597	0,07337844	0,17831500	0,04144954	0,05642585	0,51387879	0,56506941	175	0,24460063	0,00252150	3,10910642
0,13367022	0,19747610	0,13192135	0,00941222	0,02618256	2,64955486	0,01592845	0,29153682	0,02171371	0,00625294	0,00294146	0,13666825	5,53180384	0,01193010	0,19448759	0,02210858	0,04388130	0,78935709	0,89486999	176	0,35669181	0,00456158	1,76510010
0,17964865	0,13087396	0,53006824	0,01597821	0,05355815	1,97144027	0,00013231	0,34323058	0,02004311	0,04317231	0,00530751	0,22271181	4,10462380	0,14361218	0,24270067	0,03499397	0,07145853	1,31517064	0,56223688	177	0,12615331	0,00013231	1,91487471
-99	-99	-99	-99	-99	-99	-99	-99	-99	-99	-99	-99	-99	-99	-99	-99	-99	-99	-99	178	-99	-99	-99
-99	-99	-99	-99	-99	-99	-99	-99	-99	-99	-99	-99	-99	-99	-99	-99	-99	-99	-99	179	-99	-99	-99
-99	-99	-99	-99	-99	-99	-99	-99	-99	-99	-99	-99	-99	-99	-99	-99	-99	-99	-99	180	-99	-99	-99
-99	-99	-99	-99	-99	-99	-99	-99	-99	-99	-99	-99	-99	-99	-99	-99	-99	-99	-99	181	-99	-99	-99
0,08328786	0,16901814	1,55859922	0,02872100	0,73573085	0,93061091	0,11786849	0,03134219	0,03294607	0,23820077	0,00611365	0,26650786	2,63764426	0,53746771	0,21157631	0,02095226	0,40535280	0,45889946	1,08617006	182	0,21557324	0,05700570	3,73019228
0,31676833	0,08665940	1,12857320	0,02058161	0,10514849	1,75381916	0,05401485	0,18005381	0,01305277	0,02398872	0,00250062	0,16408326	5,16762005	0,36236110	0,14839352	0,03397224	0,05103037	1,20789914	0,39652947	183	0,50891619	0,01522409	3,70506795
-99	-99	-99	-99	-99	-99	-99	-99	-99	-99	-99	-99	-99	-99	-99	-99	-99	-99	-99	184	-99	-99	-99
-99	-99	-99	-99	-99	-99	-99	-99	-99	-99	-99	-99	-99	-99	-99	-99	-99	-99	-99	185	-99	-99	-99
-99	-99	-99	-99	-99	-99	-99	-99	-99	-99	-99	-99	-99	-99	-99	-99	-99	-99	-99	186	-99	-99	-99
-99	-99	-99	-99	-99	-99	-99	-99	-99	-99	-99	-99	-99	-99	-99	-99	-99	-99	-99	187	-99	-99	-99
-99	-99	-99	-99	-99	-99	-99	-99	-99	-99	-99	-99	-99	-99	-99	-99	-99	-99	-99	188	-99	-99	-99
0,20729396	0,15216938	0,37755272	0,00967012	0,01408936	0,99774981	0,00014176	0,22109679	0,00975765	0,03629086	0,00489237	0,16129236	4,82239558	0,23565529	0,07103875	0,02591175	0,11016593	0,25662791	0,51361171	189	0,30901526	0,01319148	3,00997186
-99	-99	-99	-99	-99	-99	-99	-99	-99	-99	-99	-99	-99	-99	-99	-99	-99	-99	-99	190	-99	-99	-99
-99	-99	-99	-99	-99	-99	-99	-99	-99	-99	-99	-99	-99	-99	-99	-99	-99	-99	-99	191	-99	-99	-99
-99	-99	-99	-99	-99	-99	-99	-99	-99	-99	-99	-99	-99	-99	-99	-99	-99	-99	-99	192	-99	-99	-99
-99	-99	-99	-99	-99	-99	-99	-99	-99	-99	-99	-99	-99	-99	-99	-99	-99	-99	-99	193	-99	-99	-99
-99	-99	-99	-99	-99	-99	-99	-99	-99	-99	-99	-99	-99	-99	-99	-99	-99	-99	-99	194	-99	-99	-99
-99	-99	-99	-99	-99	-99	-99	-99	-99	-99	-99	-99	-99	-99	-99	-99	-99	-99	-99	195	-99	-99	-99
-99	-99	-99	-99	-99	-99	-99	-99	-99	-99	-99	-99	-99	-99	-99	-99	-99	-99	-99	196	-99	-99	-99
-99	-99	-99	-99	-99	-99	-99	-99	-99	-99	-99	-99	-99	-99	-99	-99	-99	-99	-99	197	-99	-99	-99
0,26361420	0,10199003	1,38751176	0,03940439	0,21983607	1,61384274	0,00011521	0,24562001	0,02641498	0,11068257	0,00402582	0,22648698	2,13095481	0,09109401	0,24240645	0,01559795	0,09595488	0,54092482	0,62222143	198	0,40796011	0,01179648	1,78695091
0,26147592	0,17946196	0,96979384	0,02074274	0,72635665	1,02722342	0,09597151	0,08291345	0,01906077	0,21744915	0,00137796	0,04065989	4,77251589	0,57704271	0,27946703	0,03751861	0,15290951	0,18838422	0,72938377	199	0,29848907	0,03963036	3,69033043
-99	-99	-99	-99	-99	-99	-99	-99	-99	-99	-99	-99	-99	-99	-99	-99	-99	-99	-99	200	-99	-99	-99
-99	-99	-99	-99	-99	-99	-99	-99	-99	-99	-99	-99	-99	-99	-99	-99	-99	-99	-99	201	-99	-99	-99
0,32556105	0,23915204	2,90391656	0,02950289	0,06095972	2,50185816	0,00002102	0,33240178	0,02555944	0,08808255	0,00128591	0,28165481	6,40411849	0,11164573	0,23100564	0,00868054	0,12692076	0,17099191	0,45187636	202	0,44319085	0,02773785	1,87514219
0,07495923	0,11762364	0,41588311	0,01382334	0,05374409	2,93884649	0,02655726	0,14867665	0,01416280	0,01829065	0,00164494	0,18585508	6,15282944	0,11934831	0,06916807	0,00450346	0,07280846	0,34299275	0,31170431	203	0,20030218	0,00001769	1,71267333
-99	-99	-99	-99	-99	-99	-99	-99	-99	-99	-99	-99	-99	-99	-99	-99	-99	-99	-99	204	-99	-99	-99
-99	-99	-99	-99	-99	-99	-99	-99	-99	-99	-99	-99	-99	-99	-99	-99	-99	-99	-99	205	-99	-99	-99
-99	-99	-99	-99	-99	-99	-99	-99	-99	-99	-99	-99	-99	-99	-99	-99	-99	-99	-99	206	-99	-99	-99
-99	-99	-99	-99	-99	-99	-99	-99	-99	-99	-99	-99	-99	-99	-99	-99	-99	-99	-99	207	-99	-99	-99
-99	-99	-99	-99	-99	-99	-99	-99	-99	-99	-99	-99	-99	-99	-99	-99	-99	-99	-99	208	-99	-99	-99
0,19468991	0,12160294	0,62730980	0,01088323	0,05411791	2,32342005	0,06409031	0,13473999	0,01658761	0,00853881	0,00087663	0,26375128	3,91836013	0,81070559	0,16292162	0,03091070	0,05934417	1,09980671	0,49013463	209	0,25335812	0,00377384	1,87676758
-99	-99	-99	-99	-99	-99	-99	-99	-99	-99	-99	-99	-99	-99	-99	-99	-99	-99	-99	210	-99	-99	-99
-99	-99	-99	-99	-99	-99	-99	-99	-99	-99	-99	-99	-99	-99	-99	-99	-99	-99	-99	211	-99	-99	-99
0,05578419	0,24536477	0,97603176	0,05186841	0,14579367	1,93992382	0,05921063	0,11289081	0,01640182	0,01444786	0,00102369	0,03501216	4,56938499	0,11502346	0,06820399	0,01067219	0,10181345	0,16666233	0,91193317	212	0,31908219	0,01032296	2,23457428
-99	-99	-99	-99	-99	-99	-99	-99	-99	-99	-99	-99	-99	-99	-99	-99	-99	-99	-99	213	-99	-99	-99
0,28757297	0,09896073	0,70222244	0,01117950	0,02202064	1,23029134	0,03614651	0,21643414	0,01742135	0,00720393	0,00442840	0,07878146	4,03340982	0,05168896	0,13341567	0,01327633	0,02365026	0,73204285	0,81337920	214	0,36831192	0,01882769	2,00833509
-99	-99	-99	-99	-99	-99	-99	-99	-99	-99	-99	-99	-99	-99	-99	-99	-99	-99	-99	215	-99	-99	-99
0,18685616	0,09214183	0,32941999	0,02752717	0,02200538	1,43494953	0,04776177	0,26757217	0,01137492	0,01059113	0,00047198	0,15410654	3,81319418	0,12024093	0,16922328	0,01939730	0,03431540	0,70661682	0,33378708	216	0,24299525	0,00816688	2,13909418
-99	-99	-99	-99	-99	-99	-99	-99	-99	-99	-99	-99	-99	-99	-99	-99	-99	-99	-99	217	-99	-99	-99
-99	-99	-99	-99	-99	-99	-99	-99	-99	-99	-99	-99	-99	-99	-99	-99	-99	-99	-99	218	-99	-99	-99
-99	-99	-99	-99	-99	-99	-99	-99	-99	-99	-99	-99	-99	-99	-99	-99	-99	-99	-99	219	-99	-99	-99
0,25463453	0,18042861	1,26094032	0,01575005	0,07565084	1,65003886	0,05960181	0,31963560	0,01302566	0,00667739	0,00403420	0,20826611	4,72888481	0,09210990	0,19826474	0,01289093	0,14175780	0,15566322	0,47516484	220	0,32657811	0,00466308	2,84119923
0,06745176	0,13966089	0,56683471	0,00642094	0,05966381	1,65978917	0,01739721	0,15093507	0,00891840	0,00862655	0,00101311	0,30207946	8,21351637	0,05215683	0,11850337	0,03742121	0,18339167	0,16758907	0,94082611	221	0,35281896	0,00002785	2,07915887
0,34947230	0,10711630	0,56850699	0,01244966	0,02022453	2,48673022	0,00392865	0,10268163	0,02403449	0,01263220	0,00002916	0,06662697	6,39635495	0,08323015	0,17840773	0,01007728	0,06723005	0,49663230	0,37611611	222	0,15477560	0,00031975	2,78610571
0,26283877	0,12458912	1,03043250	0,00667161	0,04491216	2,82304084	0,03164782	0,15148533	0,01208407	0,02894083	0,00303151	0,03204515	4,66620600	0,22613403	0,14991847	0,01787702	0,13279291	0,44945544	0,60133882	223	0,31980181	0,00595885	1,32340075
-99	-99	-99	-99	-99	-99	-99	-99	-99	-99	-99	-99	-99	-99	-99	-99	-99	-99	-99	224	-99	-99	-99
0,20447551	0,11801155	0,68729435	0,01417999	0,05154585	1,47733806	0,02002575	0,07484242	0,01926332	0,05006687	0,00024397	0,19641813	3,72696172	0,16065079	0,06193935	0,02489500	0,07829153	0,64082396	0,22360123	225	0,30971217	0,00404961	1,18263100
0,07249373	0,07525859	1,28609748	0,04697380	0,04382810	2,70757256	0,00425980	0,09284706	0,02005353	0,09923549	0,00103368	0,44196377	4,21052462	0,64752150	0,08026974	0,04462512	0,14875396	0,79004131	0,98077900	226	0,41667660	0,00963834	1,35566433
-99	-99	-99	-99	-99	-99	-99	-99	-99	-99	-99	-99	-99	-99	-99	-99	-99	-99	-99	227	-99	-99	-99
-99	-99	-99	-99	-99	-99	-99	-99	-99	-99	-99	-99	-99	-99	-99	-99	-99	-99	-99	228	-99	-99	-99
-99	-99	-99	-99	-99	-99	-99	-99	-99	-99	-99	-99	-99	-99	-99	-99	-99	-99	-99	229	-99	-99	-99
-99	-99	-99	-99	-99	-99	-99	-99	-99	-99	-99	-99	-99	-99	-99	-99	-99	-99	-99	230	-99	-99	-99
-99	-99	-99	-99	-99	-99	-99	-99	-99	-99	-99	-99	-99	-99	-99	-99	-99	-99	-99	231	-99	-99	-99
0,28390890	0,09595488	1,60269511	0,02359296	0,05681832	2,20916320	0,00003418	0,07815598	0,01604758	0,03704699	0,00275544	0,31111072	5,02282844	0,66457319	0,09483083	0,02014408	0,07638854	0,64999476	0,69039791	232	0,33274756	0,01403818	1,84996760
0,19682699	0,09440454	0,33564313	0,00720393	0,09532510	1,43395525	0,06350438	0,08310044	0,02653426	0,02192925	0,00187746	0,15072598	3,11449876	0,13048899	0,13975773	0,01738516	0,18711537	0,84733243	0,24810109	233	0,26079715	0,00506578	1,07251662
0,06375799	0,09225366	0,31181236	0,01376120	0,06192862	3,93878317	0,03330194	0,29012570	0,02627346	0,00475200	0,00130816	0,02037226	5,48218115	0,39113811	0,19035310	0,03372004	0,07025526	0,94851993	0,45023496	234	0,16628731	0,00593206	1,84005642
0,11887358	0,10602674	0,57814369	0,02170995	0,04523239	2,66013599	0,04104926	0,21175971	0,00822653	0,03843328	0,00097893	0,34115517	5,34985578	0,04332970	0,06663851	0,00596815	0,13235641	0,37306528	0,47057611	235	0,33529433	0,00388869	2,22915974
-99	-99	-99	-99	-99	-99	-99	-99	-99	-99	-99	-99	-99	-99	-99	-99	-99	-99	-99	236	-99	-99	-99

-99	-99	-99	-99	-99	-99	-99	-99	-99	-99	-99	-99	-99	-99	-99	-99	-99	-99	-99	237	-99	-99	-99
-99	-99	-99	-99	-99	-99	-99	-99	-99	-99	-99	-99	-99	-99	-99	-99	-99	-99	-99	238	-99	-99	-99
-99	-99	-99	-99	-99	-99	-99	-99	-99	-99	-99	-99	-99	-99	-99	-99	-99	-99	-99	239	-99	-99	-99
-99	-99	-99	-99	-99	-99	-99	-99	-99	-99	-99	-99	-99	-99	-99	-99	-99	-99	-99	240	-99	-99	-99
-99	-99	-99	-99	-99	-99	-99	-99	-99	-99	-99	-99	-99	-99	-99	-99	-99	-99	-99	241	-99	-99	-99
-99	-99	-99	-99	-99	-99	-99	-99	-99	-99	-99	-99	-99	-99	-99	-99	-99	-99	-99	242	-99	-99	-99
0,23124595	0,09320169	0,79802177	0,06752193	0,00986476	1,85896551	0,00004834	0,07518038	0,00850484	0,00773974	0,00199347	0,12162401	5,73780922	0,02181554	0,15172177	0,01567381	0,08382358	0,07429969	0,95164804	243	0,21968374	0,00076381	2,93731909
0,27864497	0,14839352	0,68278345	0,00194435	0,03663839	1,57734692	0,02124843	0,11098988	0,01667118	0,00351198	0,00021965	0,34880678	4,23540520	0,05638675	0,21846893	0,02423944	0,07528467	0,80412963	0,52141356	244	0,21456698	0,00199486	3,65405918
-99	-99	-99	-99	-99	-99	-99	-99	-99	-99	-99	-99	-99	-99	-99	-99	-99	-99	-99	245	-99	-99	-99
0,37938906	0,10842355	1,65261424	0,01650160	0,01879835	1,04300784	0,01389539	0,06036056	0,01300085	0,00675303	0,00319881	0,03379023	4,81237811	0,07446726	0,17686862	0,00668318	0,22248038	0,30098218	0,69918687	246	0,24685743	0,02333679	2,90744117
-99	-99	-99	-99	-99	-99	-99	-99	-99	-99	-99	-99	-99	-99	-99	-99	-99	-99	-99	247	-99	-99	-99
0,28004877	0,23273337	0,55893406	0,00374907	0,06006840	2,28588052	0,00002541	0,24362769	0,01139662	0,01861681	0,00197730	0,03390754	5,47838251	0,05928250	0,12769288	0,01885054	0,05550456	0,30412788	0,84075071	248	0,18437948	0,00697665	2,12542301
0,07445435	0,10206075	0,90031390	0,00937153	0,02975962	3,17443536	0,02412211	0,06352640	0,01269584	0,02235126	0,00016002	0,14215138	4,81821910	0,19716837	0,10795485	0,02670956	0,03322700	0,34091878	0,33950389	249	0,26471283	0,00747091	1,59936586
-99	-99	-99	-99	-99	-99	-99	-99	-99	-99	-99	-99	-99	-99	-99	-99	-99	-99	-99	250	-99	-99	-99
0,24295314	0,11596409	0,97248641	0,00214583	0,12411505	1,55590071	0,02825696	0,13110095	0,00760809	0,01024987	0,00187194	0,17822233	3,07108831	1,06862080	0,37338866	0,02806177	0,07477760	0,70845592	0,58712924	251	0,28415500	0,02482177	3,90345057
-99	-99	-99	-99	-99	-99	-99	-99	-99	-99	-99	-99	-99	-99	-99	-99	-99	-99	-99	252	-99	-99	-99
-99	-99	-99	-99	-99	-99	-99	-99	-99	-99	-99	-99	-99	-99	-99	-99	-99	-99	-99	253	-99	-99	-99
-99	-99	-99	-99	-99	-99	-99	-99	-99	-99	-99	-99	-99	-99	-99	-99	-99	-99	-99	254	-99	-99	-99
-99	-99	-99	-99	-99	-99	-99	-99	-99	-99	-99	-99	-99	-99	-99	-99	-99	-99	-99	255	-99	-99	-99
0,23557364	0,08797577	0,20823002	0,00487038	0,03808192	1,84836541	0,01030330	0,23200857	0,01993227	0,00293434	0,00261903	0,19550129	4,65328645	0,57365291	0,12641591	0,02166110	0,09481440	0,37585549	0,24831615	256	0,24694300	0,00043559	1,31243866
-99	-99	-99	-99	-99	-99	-99	-99	-99	-99	-99	-99	-99	-99	-99	-99	-99	-99	-99	257	-99	-99	-99
-99	-99	-99	-99	-99	-99	-99	-99	-99	-99	-99	-99	-99	-99	-99	-99	-99	-99	-99	258	-99	-99	-99
0,20316869	0,14158595	0,56282176	0,01155571	0,09104667	0,87645383	0,06270612	0,19665653	0,02641956	0,14396101	0,00097437	0,33698353	4,63558045	0,20458184	0,28752315	0,02513341	0,09079458	0,15143284	0,57944756	259	0,23630958	0,01490304	2,90542658
-99	-99	-99	-99	-99	-99	-99	-99	-99	-99	-99	-99	-99	-99	-99	-99	-99	-99	-99	260	-99	-99	-99
0,15731738	0,07909608	0,66607198	0,00482167	0,04533440	2,48242477	0,02356435	0,05742210	0,00534628	0,00938615	0,00187064	0,12334319	3,86575445	0,63672839	0,08806728	0,00672034	0,08430430	0,06145823	1,09790255	261	0,19898754	0,00490256	3,82576962
0,26660024	0,19611204	1,71802377	0,01091722	0,13247114	2,13428080	0,03561799	0,08115085	0,02268292	0,02137029	0,00332081	0,43399490	3,75223452	0,10480286	0,15375977	0,03809512	0,35545775	0,24754282	1,09866382	262	0,39852717	0,01084933	4,21563512
-99	-99	-99	-99	-99	-99	-99	-99	-99	-99	-99	-99	-99	-99	-99	-99	-99	-99	-99	263	-99	-99	-99
0,09320169	0,13807270	0,52832588	0,01027655	0,00666814	1,48812942	0,03954119	0,05845621	0,01569556	0,03756414	0,00198658	0,12504333	4,96743125	0,06127745	0,21576010	0,01915016	0,15130169	0,22822045	0,84177117	264	0,31852974	0,01807952	2,70475890
0,26070678	0,20341529	0,40880932	0,02010224	0,01404792	0,46506362	0,00256558	0,02225850	0,02676516	0,00353396	0,00062994	0,04583203	3,52714283	0,07848169	0,08683979	0,02732755	0,19950544	0,17598203	1,23584687	265	0,33575947	0,00202130	2,70101191

JKI Shelter_Ds [68015]																		
32	31	28	19	26	5	36	20	29	14	30	9	13	2	18	16	10	7	34
**Serine-threonine_protein_kinase_plant-type**	**TMV_resistance_protein_N**	**Homoserine_dehydrogenase_family_protein**	**Cc-nbs-lrr resistance protein**	**UDP-glucose: glucosyltransferase**	**Methionyl-tRNA_synthetase**	**70_kDa_subunit_of_replication_protein_A**	**Gene_of_unknown_function**	**RING_finger_protein**	**Flagellin-sensing 2**	**Serine-threonine protein kinase, plant-type**	**TIR-NBS-LRR_disease_resistance**	**LRR receptor-like serine/threonine-protein kinase**	**Poly(ADP-ribose)_glycohydrolase**	**Bacterial_spot_disease_resistance_protein_4**	**Extensin**	**Lipoxygenase**	**Betaine_aldehyde_dehyrogenase**	**Fatty_acid_desaturase**
GSC0003 DMT40004644	PGSC0003 DMT40004689	PGSC0003 DMT40004811	PGSC0003 DMT40004909	PGSC0003 DMT40004912	PGSC0003 DMT40004917	PGSC0003 DMT40005251	PGSC0003 DMT40006237	PGSC0003 DMT40006687	PGSC0003 DMT40006877	PGSC0003 DMT40006878	PGSC0003 DMT40007144	PGSC0003 DMT40007188	PGSC0003 DMT40007551	PGSC0003 DMT40008081	PGSC0003 DMT40008201	PGSC0003 DMT40008202	PGSC0003 DMT40008302	PGSC0003 DMT40008385
0,04484217	0,11454608	0,06796802	0,10995613	1,61552156	0,12639401	0,60541654	0,23229017	1,86218964	0,02332466	0,13231055	0,31753775	0,09793715	0,50208377	0,24502485	1,13366941	2,87786716	0,53218509	0,00186838
-99	-99	-99	-99	-99	-99	-99	-99	-99	-99	-99	-99	-99	-99	-99	-99	-99	-99	-99
-99	-99	-99	-99	-99	-99	-99	-99	-99	-99	-99	-99	-99	-99	-99	-99	-99	-99	-99
-99	-99	-99	-99	-99	-99	-99	-99	-99	-99	-99	-99	-99	-99	-99	-99	-99	-99	-99
-99	-99	-99	-99	-99	-99	-99	-99	-99	-99	-99	-99	-99	-99	-99	-99	-99	-99	-99
0,02871603	0,22988751	0,01147788	0,11711518	2,67214816	0,25000000	0,80496614	0,09565605	2,54382601	0,08127751	0,11383373	0,23536962	0,04948037	0,55133458	0,04478005	0,68634222	4,48155686	0,35898613	0,00794355
-99	-99	-99	-99	-99	-99	-99	-99	-99	-99	-99	-99	-99	-99	-99	-99	-99	-99	-99
-99	-99	-99	-99	-99	-99	-99	-99	-99	-99	-99	-99	-99	-99	-99	-99	-99	-99	-99
-99	-99	-99	-99	-99	-99	-99	-99	-99	-99	-99	-99	-99	-99	-99	-99	-99	-99	-99
-99	-99	-99	-99	-99	-99	-99	-99	-99	-99	-99	-99	-99	-99	-99	-99	-99	-99	-99
-99	-99	-99	-99	-99	-99	-99	-99	-99	-99	-99	-99	-99	-99	-99	-99	-99	-99	-99
-99	-99	-99	-99	-99	-99	-99	-99	-99	-99	-99	-99	-99	-99	-99	-99	-99	-99	-99
-99	-99	-99	-99	-99	-99	-99	-99	-99	-99	-99	-99	-99	-99	-99	-99	-99	-99	-99
-99	-99	-99	-99	-99	-99	-99	-99	-99	-99	-99	-99	-99	-99	-99	-99	-99	-99	-99
-99	-99	-99	-99	-99	-99	-99	-99	-99	-99	-99	-99	-99	-99	-99	-99	-99	-99	-99
0,02189887	0,22221067	0,02701679	0,27018135	3,13398875	0,16009498	0,04100660	0,08573333	2,05480288	0,10555011	0,15854892	0,37500975	0,05823378	0,47302882	0,14701141	0,67501899	1,27987241	0,34796160	0,00329844
0,04196266	0,28229001	0,10491188	0,09120457	3,14977767	0,28722436	0,01337099	0,58045253	2,58336232	0,17233050	0,27840364	0,50671625	0,11143312	0,52422207	0,05922089	0,48506500	1,98377719	0,59511897	0,00003912
0,04763778	0,15054326	0,05614300	0,22739146	2,57442457	0,21542387	0,02092324	0,01813914	3,50703055	0,09481440	0,09640488	0,17364948	0,09273450	0,52531329	0,05625987	0,30719997	1,87416763	0,47838697	0,00004245
0,04702266	0,17905814	0,01819266	0,10220233	2,69540115	0,19045209	0,88853469	0,02652506	2,65093262	0,29596531	0,33459783	0,22398903	0,07853611	0,29658139	0,03126083	0,68278345	1,49019384	0,46281258	0,00318057
-99	-99	-99	-99	-99	-99	-99	-99	-99	-99	-99	-99	-99	-99	-99	-99	-99	-99	-99
-99	-99	-99	-99	-99	-99	-99	-99	-99	-99	-99	-99	-99	-99	-99	-99	-99	-99	-99
-99	-99	-99	-99	-99	-99	-99	-99	-99	-99	-99	-99	-99	-99	-99	-99	-99	-99	-99
-99	-99	-99	-99	-99	-99	-99	-99	-99	-99	-99	-99	-99	-99	-99	-99	-99	-99	-99
0,03956860	0,27442808	0,06627001	0,05541806	2,20152008	0,16963432	0,69858133	0,20187022	2,41747337	0,12539050	0,15620364	0,16092942	0,09122038	0,31392678	0,06860702	1,61608155	1,92318827	0,51637820	0,00323168
-99	-99	-99	-99	-99	-99	-99	-99	-99	-99	-99	-99	-99	-99	-99	-99	-99	-99	-99
-99	-99	-99	-99	-99	-99	-99	-99	-99	-99	-99	-99	-99	-99	-99	-99	-99	-99	-99
-99	-99	-99	-99	-99	-99	-99	-99	-99	-99	-99	-99	-99	-99	-99	-99	-99	-99	-99
-99	-99	-99	-99	-99	-99	-99	-99	-99	-99	-99	-99	-99	-99	-99	-99	-99	-99	-99
-99	-99	-99	-99	-99	-99	-99	-99	-99	-99	-99	-99	-99	-99	-99	-99	-99	-99	-99
-99	-99	-99	-99	-99	-99	-99	-99	-99	-99	-99	-99	-99	-99	-99	-99	-99	-99	-99
-99	-99	-99	-99	-99	-99	-99	-99	-99	-99	-99	-99	-99	-99	-99	-99	-99	-99	-99
-99	-99	-99	-99	-99	-99	-99	-99	-99	-99	-99	-99	-99	-99	-99	-99	-99	-99	-99
0,04815235	0,14945157	0,03358009	0,14012148	1,12876879	0,10737649	0,57624331	0,03490917	2,16107629	0,11201393	0,15558232	0,13896081	0,01430586	0,31748273	0,07904127	0,22294349	0,64115719	0,28199666	0,00190466
-99	-99	-99	-99	-99	-99	-99	-99	-99	-99	-99	-99	-99	-99	-99	-99	-99	-99	-99
0,03816780	0,29719876	0,07019441	0,20826611	2,32865998	0,26952668	0,45612464	0,07297267	2,53942172	0,09191856	0,09940760	0,32567390	0,03684212	0,31786807	0,11748105	0,51423510	1,16756268	0,32703116	0,00001211
-99	-99	-99	-99	-99	-99	-99	-99	-99	-99	-99	-99	-99	-99	-99	-99	-99	-99	-99
-99	-99	-99	-99	-99	-99	-99	-99	-99	-99	-99	-99	-99	-99	-99	-99	-99	-99	-99
-99	-99	-99	-99	-99	-99	-99	-99	-99	-99	-99	-99	-99	-99	-99	-99	-99	-99	-99
-99	-99	-99	-99	-99	-99	-99	-99	-99	-99	-99	-99	-99	-99	-99	-99	-99	-99	-99
0,04893464	0,20320390	0,05719370	0,10576982	2,93020175	0,10547697	0,12430877	0,07259430	2,77213977	0,08925025	0,10445837	0,18985896	0,03823400	0,59666787	0,14640127	0,33891608	1,06363267	0,28048587	0,00305418
-99	-99	-99	-99	-99	-99	-99	-99	-99	-99	-99	-99	-99	-99	-99	-99	-99	-99	-99
-99	-99	-99	-99	-99	-99	-99	-99	-99	-99	-99	-99	-99	-99	-99	-99	-99	-99	-99
0,04391172	0,25186979	0,07113730	0,07730738	2,04167043	0,26752581	0,38789820	0,08542191	2,27323999	0,30987322	0,37912618	0,28932241	0,09771677	0,46675908	0,15775416	0,36041976	2,47555156	0,36901465	0,00486195
-99	-99	-99	-99	-99	-99	-99	-99	-99	-99	-99	-99	-99	-99	-99	-99	-99	-99	-99
-99	-99	-99	-99	-99	-99	-99	-99	-99	-99	-99	-99	-99	-99	-99	-99	-99	-99	-99
0,03785821	0,16028930	0,04218138	0,08199899	2,46527741	0,16721197	0,82573456	0,01081180	2,81229902	0,02454801	0,03488498	0,26057128	0,03113649	0,38978487	0,16286517	2,88136015	5,82294907	0,39195230	0,00479834
-99	-99	-99	-99	-99	-99	-99	-99	-99	-99	-99	-99	-99	-99	-99	-99	-99	-99	-99
-99	-99	-99	-99	-99	-99	-99	-99	-99	-99	-99	-99	-99	-99	-99	-99	-99	-99	-99
0,04005150	0,13946742	0,02231256	0,05987095	1,97794183	0,22484458	1,13288389	0,40248307	1,99446250	0,05094202	0,04349520	0,40053494	0,04866404	0,45501941	0,02335702	0,18288390	0,26134003	0,21212698	0,00186320
0,04390412	0,17464533	0,03449426	0,10691232	2,53414662	0,20383872	0,08063221	0,09040209	2,56063212	0,03397224	0,03218428	0,16431089	0,06423485	0,44797807	0,16294986	0,24291105	0,26785052	0,44922185	0,00225212
0,03615277	0,24455824	0,02433624	0,06195008	2,50055788	0,06489495	0,54233268	0,03683574	2,43555405	0,03691242	0,03080375	0,40507192	0,07301061	0,52204642	0,06493995	0,18805805	0,39646076	0,18152624	0,00002213
-99	-99	-99	-99	-99	-99	-99	-99	-99	-99	-99	-99	-99	-99	-99	-99	-99	-99	-99
-99	-99	-99	-99	-99	-99	-99	-99	-99	-99	-99	-99	-99	-99	-99	-99	-99	-99	-99
-99	-99	-99	-99	-99	-99	-99	-99	-99	-99	-99	-99	-99	-99	-99	-99	-99	-99	-99
-99	-99	-99	-99	-99	-99	-99	-99	-99	-99	-99	-99	-99	-99	-99	-99	-99	-99	-99
-99	-99	-99	-99	-99	-99	-99	-99	-99	-99	-99	-99	-99	-99	-99	-99	-99	-99	-99
0,06783859	0,14341323	0,03874758	0,06203602	1,88197831	0,15596022	0,09925269	0,23104568	1,81158186	0,10368291	0,09822608	0,23836594	0,04420183	0,37507474	0,04585586	3,42772175	7,97370372	0,36710127	0,00203078
0,06637345	0,23549201	0,06406810	0,12022010	3,03721702	0,18514789	0,54971281	0,02600170	2,00242749	0,06847637	0,09663904	0,15547451	0,20989650	0,51432422	0,09880652	3,32821815	13,61146078	0,34311165	0,04657663
-99	-99	-99	-99	-99	-99	-99	-99	-99	-99	-99	-99	-99	-99	-99	-99	-99	-99	-99
-99	-99	-99	-99	-99	-99	-99	-99	-99	-99	-99	-99	-99	-99	-99	-99	-99	-99	-99
0,04852090	0,11170379	0,03631602	0,19022121	2,82989789	0,31880585	1,11400059	0,03236885	2,76781976	0,05093319	0,07550678	0,18604841	0,02309541	0,36192182	0,10531260	10,68674060	11,80028665	0,34645743	0,01015793
-99	-99	-99	-99	-99	-99	-99	-99	-99	-99	-99	-99	-99	-99	-99	-99	-99	-99	-99
-99	-99	-99	-99	-99	-99	-99	-99	-99	-99	-99	-99	-99	-99	-99	-99	-99	-99	-99
-99	-99	-99	-99	-99	-99	-99	-99	-99	-99	-99	-99	-99	-99	-99	-99	-99	-99	-99
0,03639162	0,24413482	0,02069965	0,07656082	2,12689675	0,25204443	1,13268759	0,06393501	1,89572540	0,05135861	0,05600696	0,49234790	0,04696566	0,50618968	0,13283894	1,55374527	1,81850135	0,66514925	0,00396284
-99	-99	-99	-99	-99	-99	-99	-99	-99	-99	-99	-99	-99	-99	-99	-99	-99	-99	-99
-99	-99	-99	-99	-99	-99	-99	-99	-99	-99	-99	-99	-99	-99	-99	-99	-99	-99	-99
-99	-99	-99	-99	-99	-99	-99	-99	-99	-99	-99	-99	-99	-99	-99	-99	-99	-99	-99
-99	-99	-99	-99	-99	-99	-99	-99	-99	-99	-99	-99	-99	-99	-99	-99	-99	-99	-99
-99	-99	-99	-99	-99	-99	-99	-99	-99	-99	-99	-99	-99	-99	-99	-99	-99	-99	-99

-99	-99	-99	-99	-99	-99	-99	-99	-99	-99	-99	-99	-99	-99	-99	-99	-99	-99	-99
-99	-99	-99	-99	-99	-99	-99	-99	-99	-99	-99	-99	-99	-99	-99	-99	-99	-99	-99
-99	-99	-99	-99	-99	-99	-99	-99	-99	-99	-99	-99	-99	-99	-99	-99	-99	-99	-99
-99	-99	-99	-99	-99	-99	-99	-99	-99	-99	-99	-99	-99	-99	-99	-99	-99	-99	-99
0,06357045	0,16695139	0,04433224	0,04821079	2,63079713	0,01519247	0,15663733	0,18080419	3,64141710	0,10086517	0,11797066	0,15182697	0,13874426	0,68610439	0,04821079	0,36665624	1,34303788	0,32432227	0,00540122
-99	-99	-99	-99	-99	-99	-99	-99	-99	-99	-99	-99	-99	-99	-99	-99	-99	-99	-99
-99	-99	-99	-99	-99	-99	-99	-99	-99	-99	-99	-99	-99	-99	-99	-99	-99	-99	-99
-99	-99	-99	-99	-99	-99	-99	-99	-99	-99	-99	-99	-99	-99	-99	-99	-99	-99	-99
-99	-99	-99	-99	-99	-99	-99	-99	-99	-99	-99	-99	-99	-99	-99	-99	-99	-99	-99
-99	-99	-99	-99	-99	-99	-99	-99	-99	-99	-99	-99	-99	-99	-99	-99	-99	-99	-99
-99	-99	-99	-99	-99	-99	-99	-99	-99	-99	-99	-99	-99	-99	-99	-99	-99	-99	-99
-99	-99	-99	-99	-99	-99	-99	-99	-99	-99	-99	-99	-99	-99	-99	-99	-99	-99	-99
-99	-99	-99	-99	-99	-99	-99	-99	-99	-99	-99	-99	-99	-99	-99	-99	-99	-99	-99
0,02689021	0,27110526	0,01606901	0,00083817	3,06869444	0,19110335	0,63906076	0,19228592	3,07316451	0,05657173	0,08294076	0,38098999	0,12848083	0,51374523	0,12045156	0,85005318	1,16177042	0,26650324	0,00990055
-99	-99	-99	-99	-99	-99	-99	-99	-99	-99	-99	-99	-99	-99	-99	-99	-99	-99	-99
-99	-99	-99	-99	-99	-99	-99	-99	-99	-99	-99	-99	-99	-99	-99	-99	-99	-99	-99
0,02065307	0,17863665	0,03981070	0,15346696	2,04769380	0,17803095	0,52748428	0,10280626	1,47928497	0,04459111	0,06997581	0,15023574	0,10718686	0,42628790	0,12460208	1,47222686	5,48579230	0,33800114	0,00459570
0,03726203	0,12563845	0,06878081	0,17297674	2,25769406	0,27736353	0,06362555	0,03683829	2,08413683	0,01570045	0,01148385	0,12867021	0,02212391	0,49470830	0,01904888	1,22497304	1,65387477	0,71675402	0,00383024
0,01643112	0,19432925	0,01448773	0,00002334	1,20369927	0,16711058	0,11003810	0,49191297	2,04567222	0,07813837	0,08411897	0,16871674	0,09163706	0,32306022	0,25654343	2,49869533	9,17288148	0,44555487	0,00192193
-99	-99	-99	-99	-99	-99	-99	-99	-99	-99	-99	-99	-99	-99	-99	-99	-99	-99	-99
-99	-99	-99	-99	-99	-99	-99	-99	-99	-99	-99	-99	-99	-99	-99	-99	-99	-99	-99
-99	-99	-99	-99	-99	-99	-99	-99	-99	-99	-99	-99	-99	-99	-99	-99	-99	-99	-99
-99	-99	-99	-99	-99	-99	-99	-99	-99	-99	-99	-99	-99	-99	-99	-99	-99	-99	-99
-99	-99	-99	-99	-99	-99	-99	-99	-99	-99	-99	-99	-99	-99	-99	-99	-99	-99	-99
-99	-99	-99	-99	-99	-99	-99	-99	-99	-99	-99	-99	-99	-99	-99	-99	-99	-99	-99
-99	-99	-99	-99	-99	-99	-99	-99	-99	-99	-99	-99	-99	-99	-99	-99	-99	-99	-99
-99	-99	-99	-99	-99	-99	-99	-99	-99	-99	-99	-99	-99	-99	-99	-99	-99	-99	-99
-99	-99	-99	-99	-99	-99	-99	-99	-99	-99	-99	-99	-99	-99	-99	-99	-99	-99	-99
0,01441011	0,17162421	0,03267713	0,04763365	1,90544108	0,14255840	0,65321282	0,01715562	2,41299512	0,03223172	0,03762212	0,32351959	0,04261559	0,36486255	0,13860248	0,18958609	0,57683276	0,56806385	0,00463641
0,07463520	0,34484023	0,04806065	0,03911864	1,71475207	0,22113511	1,35848628	0,20949678	1,63127499	0,09786929	0,08826590	0,27951546	0,13707145	0,39721720	0,08993335	0,56919701	1,90000077	0,43921523	0,00392797
-99	-99	-99	-99	-99	-99	-99	-99	-99	-99	-99	-99	-99	-99	-99	-99	-99	-99	-99
-99	-99	-99	-99	-99	-99	-99	-99	-99	-99	-99	-99	-99	-99	-99	-99	-99	-99	-99
0,04850408	0,20866348	0,20436924	0,07071941	2,91248375	0,29776581	0,00855658	0,08846496	2,09688828	0,06476014	0,08123527	0,44914401	0,20967838	0,35830250	0,14652817	2,66705948	5,72093122	0,43114648	0,00547567
0,06779158	0,24660091	0,00625077	0,05563937	2,23496153	0,24102421	0,00563351	0,10035956	3,06577113	0,29632454	0,34753977	0,31781299	0,05426817	0,38348711	0,09714274	0,37093801	0,61269883	0,32359248	0,01742437
-99	-99	-99	-99	-99	-99	-99	-99	-99	-99	-99	-99	-99	-99	-99	-99	-99	-99	-99
0,04511497	0,13676301	0,03009672	0,12911692	2,67539146	0,18399647	0,06231615	0,11054840	1,95985918	0,01113118	0,01722622	0,51077152	0,12030346	0,63408581	0,12342872	0,00524715	0,88745754	0,40437060	0,00612425
0,03109335	0,21850679	0,01752733	0,09511060	2,12395029	0,23680148	0,96176078	0,03075042	1,78726059	0,05686757	0,09731122	0,25591737	0,12013680	0,52258948	0,18272551	0,26238370	0,73090206	0,45148501	0,01008776
-99	-99	-99	-99	-99	-99	-99	-99	-99	-99	-99	-99	-99	-99	-99	-99	-99	-99	-99
-99	-99	-99	-99	-99	-99	-99	-99	-99	-99	-99	-99	-99	-99	-99	-99	-99	-99	-99
0,07559843	0,13932248	0,02450976	0,13598317	3,62379162	0,32477219	1,09923512	0,11538279	4,26190962	0,05264718	0,05074818	0,69955045	0,21426974	0,61195607	0,17893407	1,37983905	7,83807838	0,70686176	0,01131594
-99	-99	-99	-99	-99	-99	-99	-99	-99	-99	-99	-99	-99	-99	-99	-99	-99	-99	-99
-99	-99	-99	-99	-99	-99	-99	-99	-99	-99	-99	-99	-99	-99	-99	-99	-99	-99	-99
-99	-99	-99	-99	-99	-99	-99	-99	-99	-99	-99	-99	-99	-99	-99	-99	-99	-99	-99
0,06617821	0,30344354	0,03147827	0,06221904	3,56153705	0,22523454	0,16556849	0,00754376	2,27127123	0,05221109	0,04963494	0,51566284	0,19164390	0,37023161	0,06286932	5,62362346	16,06111337	0,58297261	0,02021402
-99	-99	-99	-99	-99	-99	-99	-99	-99	-99	-99	-99	-99	-99	-99	-99	-99	-99	-99
-99	-99	-99	-99	-99	-99	-99	-99	-99	-99	-99	-99	-99	-99	-99	-99	-99	-99	-99
-99	-99	-99	-99	-99	-99	-99	-99	-99	-99	-99	-99	-99	-99	-99	-99	-99	-99	-99
-99	-99	-99	-99	-99	-99	-99	-99	-99	-99	-99	-99	-99	-99	-99	-99	-99	-99	-99
-99	-99	-99	-99	-99	-99	-99	-99	-99	-99	-99	-99	-99	-99	-99	-99	-99	-99	-99
0,06700906	0,29494135	0,00907743	0,05793183	2,38253928	0,36997508	0,05480685	0,04168003	2,52713021	0,04692498	0,13495029	0,18383712	0,04357064	0,30576602	0,12547745	0,27442808	0,14175780	0,20271152	0,00134005
0,05562009	0,23712998	0,03030606	0,10130308	2,14614864	0,14456097	0,61418705	0,03532296	2,38460449	0,07405546	0,02859189	0,42653913	0,22278901	0,49887490	0,12672297	7,39089972	11,99823260	0,37886348	0,01097032
0,14151236	0,16551111	0,01159784	0,07791257	1,74230838	0,13898489	1,33792755	0,03453014	1,26225203	0,02259269	0,03682936	0,46137125	0,16109683	0,71598437	0,21227406	1,33145161	2,59188193	0,47434216	0,00992994
-99	-99	-99	-99	-99	-99	-99	-99	-99	-99	-99	-99	-99	-99	-99	-99	-99	-99	-99
-99	-99	-99	-99	-99	-99	-99	-99	-99	-99	-99	-99	-99	-99	-99	-99	-99	-99	-99
-99	-99	-99	-99	-99	-99	-99	-99	-99	-99	-99	-99	-99	-99	-99	-99	-99	-99	-99
-99	-99	-99	-99	-99	-99	-99	-99	-99	-99	-99	-99	-99	-99	-99	-99	-99	-99	-99
0,08395441	0,22125010	0,05538926	0,11070176	2,29540703	0,21253171	0,10745094	0,17540357	2,68096057	0,37235484	0,37468497	0,43913913	0,01142034	0,47855280	0,00539093	0,41115378	1,44869109	0,55854677	0,00003483
-99	-99	-99	-99	-99	-99	-99	-99	-99	-99	-99	-99	-99	-99	-99	-99	-99	-99	-99
-99	-99	-99	-99	-99	-99	-99	-99	-99	-99	-99	-99	-99	-99	-99	-99	-99	-99	-99
-99	-99	-99	-99	-99	-99	-99	-99	-99	-99	-99	-99	-99	-99	-99	-99	-99	-99	-99
-99	-99	-99	-99	-99	-99	-99	-99	-99	-99	-99	-99	-99	-99	-99	-99	-99	-99	-99
-99	-99	-99	-99	-99	-99	-99	-99	-99	-99	-99	-99	-99	-99	-99	-99	-99	-99	-99
-99	-99	-99	-99	-99	-99	-99	-99	-99	-99	-99	-99	-99	-99	-99	-99	-99	-99	-99
-99	-99	-99	-99	-99	-99	-99	-99	-99	-99	-99	-99	-99	-99	-99	-99	-99	-99	-99
-99	-99	-99	-99	-99	-99	-99	-99	-99	-99	-99	-99	-99	-99	-99	-99	-99	-99	-99
-99	-99	-99	-99	-99	-99	-99	-99	-99	-99	-99	-99	-99	-99	-99	-99	-99	-99	-99
-99	-99	-99	-99	-99	-99	-99	-99	-99	-99	-99	-99	-99	-99	-99	-99	-99	-99	-99
0,16348725	0,25178251	0,09741245	0,13390205	4,17058569	0,77123905	0,09761523	0,10921554	2,39371269	0,06063313	0,05393068	0,26724780	0,30592502	1,04264642	0,13614822	0,79567435	4,44520514	0,41215246	0,01098935
-99	-99	-99	-99	-99	-99	-99	-99	-99	-99	-99	-99	-99	-99	-99	-99	-99	-99	-99
0,05523590	0,43099708	0,03796332	0,08904942	1,44468003	0,19747610	0,70307476	0,02057092	2,67724654	0,02798408	0,04995421	0,18463526	0,06720675	0,52006001	0,11116311	3,20594572	3,00163801	0,51325582	0,01639898
0,03425005	0,15650167	0,01601147	0,07135953	1,50968755	0,24303736	0,70771971	0,04161507	1,89769745	0,03869390	0,02610102	0,46177117	0,03701490	0,17114013	0,09667254	0,23313702	0,82373375	0,40395038	0,00508425
0,11001331	0,12716292	0,08577791	0,00032164	2,54691357	0,10611864	0,96777931	0,02516827	2,28113211	0,01952884	0,02557273	0,26808269	0,07666702	0,36722852	0,12506500	0,35007841	0,93157898	0,28474650	0,01017907
0,04132046	0,24638733	0,00878343	0,11203334	1,92785962	0,17702193	1,07474917	0,03431540	1,61552156	0,03289472	0,03417298	0,40081266	0,13850404	0,48835426	0,07791257	1,47631441	4,08121864	0,38077218	0,00351807
-99	-99	-99	-99	-99	-99	-99	-99	-99	-99	-99	-99	-99	-99	-99	-99	-99	-99	-99
0,03813475	0,28248575	0,05464563	0,05963280	2,99436481	0,31913749	0,05517850	0,09514357	1,94160536	0,02958993	0,03071847	0,33062094	0,02942630	0,27724820	0,09843055	1,45826205	1,29347305	0,54346160	0,00817962
-99	-99	-99	-99	-99	-99	-99	-99	-99	-99	-99	-99	-99	-99	-99	-99	-99	-99	-99
0,09697455	0,36545103	0,03163686	0,11062505	2,07879861	0,24044019	0,49749364	0,05398678	2,55044679	0,09412684	0,14607184	0,19061717	0,08554041	0,65690197	0,15461476	7,57239674	26,47850934	0,45969536	0,00427681
-99	-99	-99	-99	-99	-99	-99	-99	-99	-99	-99	-99	-99	-99	-99	-99	-99	-99	-99
-99	-99	-99	-99	-99	-99	-99	-99	-99	-99	-99	-99	-99	-99	-99	-99	-99	-99	-99
-99	-99	-99	-99	-99	-99	-99	-99	-99	-99	-99	-99	-99	-99	-99	-99	-99	-99	-99
-99	-99	-99	-99	-99	-99	-99	-99	-99	-99	-99	-99	-99	-99	-99	-99	-99	-99	-99
0,05644541	0,18124335	0,01512417	0,00003142	3,37058732	0,17961752	0,64215790	0,01100269	2,34404457	0,04559439	0,05448489	0,17604303	0,06958885	0,46490247	0,63949279	0,34651747	0,62720110	0,28204553	0,00003142
-99	-99	-99	-99	-99	-99	-99	-99	-99	-99	-99	-99	-99	-99	-99	-99	-99	-99	-99

-99	-99	-99	-99	-99	-99	-99	-99	-99	-99	-99	-99	-99	-99	-99	-99	-99	-99	-99
-99	-99	-99	-99	-99	-99	-99	-99	-99	-99	-99	-99	-99	-99	-99	-99	-99	-99	-99
-99	-99	-99	-99	-99	-99	-99	-99	-99	-99	-99	-99	-99	-99	-99	-99	-99	-99	-99
-99	-99	-99	-99	-99	-99	-99	-99	-99	-99	-99	-99	-99	-99	-99	-99	-99	-99	-99
-99	-99	-99	-99	-99	-99	-99	-99	-99	-99	-99	-99	-99	-99	-99	-99	-99	-99	-99
0,05962247	0,19277635	0,03928167	0,00002722	2,73018744	0,28479584	1,28788163	0,03112030	3,10157283	0,02849791	0,04607091	0,25560713	0,11242229	0,39694197	0,54563194	0,29463485	0,62503115	0,25104189	0,01200684
0,03333081	0,22925101	0,02353579	0,06289111	1,91189064	0,19519662	0,01893239	0,04694125	1,96700415	0,00519738	0,08711108	0,18815584	0,17788293	0,34198383	0,05459831	0,43316843	0,45375958	0,31665856	0,00004072
0,07378646	0,25481109	0,06636195	0,00004747	2,86294509	0,35343088	2,04273209	0,16869627	2,59278036	0,20898916	0,16431089	0,26106845	0,13607746	0,48582209	0,68705619	0,29027656	0,32252882	0,42059395	0,00004747
-99	-99	-99	-99	-99	-99	-99	-99	-99	-99	-99	-99	-99	-99	-99	-99	-99	-99	-99
-99	-99	-99	-99	-99	-99	-99	-99	-99	-99	-99	-99	-99	-99	-99	-99	-99	-99	-99
0,04819409	0,29402281	0,07060920	0,17665421	4,61394242	0,38103621	0,10754408	0,56019461	1,91454292	0,14741957	0,02660793	0,24131676	0,09362256	0,58438862	0,13406458	0,95793522	0,07274541	0,56959169	0,00006323
-99	-99	-99	-99	-99	-99	-99	-99	-99	-99	-99	-99	-99	-99	-99	-99	-99	-99	-99
-99	-99	-99	-99	-99	-99	-99	-99	-99	-99	-99	-99	-99	-99	-99	-99	-99	-99	-99
0,00887984	0,21987416	0,02441227	0,05879144	3,05198950	0,15206394	1,53023158	0,14841924	2,33229455	0,19035310	0,13339255	0,24261657	0,04300803	0,43279328	0,02120796	4,87955641	14,01354447	0,32258472	0,00165695
-99	-99	-99	-99	-99	-99	-99	-99	-99	-99	-99	-99	-99	-99	-99	-99	-99	-99	-99
-99	-99	-99	-99	-99	-99	-99	-99	-99	-99	-99	-99	-99	-99	-99	-99	-99	-99	-99
-99	-99	-99	-99	-99	-99	-99	-99	-99	-99	-99	-99	-99	-99	-99	-99	-99	-99	-99
0,03295178	0,21015126	0,04605495	0,10646861	2,02440789	0,17893407	0,34929067	0,13053422	2,37429632	0,09824311	0,20725804	0,18874365	0,05297663	0,25375357	0,03492733	0,07029179	0,42528445	0,36818430	0,00814709
0,05211166	0,14556647	0,03697644	0,13857606	1,41494895	0,15290951	0,04762127	0,08314366	1,01942102	0,02372826	0,03591549	0,40577447	0,10364698	0,41805075	0,08228367	0,06702067	0,64740930	0,21386169	0,00947112
-99	-99	-99	-99	-99	-99	-99	-99	-99	-99	-99	-99	-99	-99	-99	-99	-99	-99	-99
0,04521672	0,20166044	0,06289111	0,06717182	1,26050339	0,11858554	0,95926412	0,01199852	1,39088197	0,18275718	0,24232245	0,64037993	0,04619883	0,44627349	0,04140647	0,83335321	2,70757256	0,21110022	0,00266251
0,09249377	0,26016522	0,05246503	0,00003984	3,25238940	0,25872655	0,17573823	0,04191179	3,10264793	0,05650412	0,09430643	0,45517714	0,07373534	0,34424319	0,05213876	0,45675740	1,70113779	0,23545120	0,00335029
0,05107461	0,17711398	0,05899555	0,15601428	1,93958769	0,11365633	0,04742363	0,01868145	2,22954605	0,01249721	0,02829616	0,19516280	0,02980607	0,43640817	0,07714679	0,25823384	0,77741131	0,19394910	0,00353457
0,04880761	0,19740768	0,06017258	0,15106591	1,78416618	0,21995038	0,31452574	0,15477560	2,18290599	0,04582409	0,03617784	0,57444871	0,11081691	0,35410522	0,16473854	1,33630562	8,32961607	0,42787165	0,00013231
-99	-99	-99	-99	-99	-99	-99	-99	-99	-99	-99	-99	-99	-99	-99	-99	-99	-99	-99
-99	-99	-99	-99	-99	-99	-99	-99	-99	-99	-99	-99	-99	-99	-99	-99	-99	-99	-99
-99	-99	-99	-99	-99	-99	-99	-99	-99	-99	-99	-99	-99	-99	-99	-99	-99	-99	-99
-99	-99	-99	-99	-99	-99	-99	-99	-99	-99	-99	-99	-99	-99	-99	-99	-99	-99	-99
0,03044291	0,19428548	0,05808261	0,00010688	3,61814443	0,18089821	0,00797804	0,03136393	1,82291842	0,02430252	0,02584896	0,27286325	0,11127875	0,40060435	0,14481169	13,39154026	16,36169151	0,50182283	0,00391777
0,04273317	0,18537261	0,04977275	0,13551270	3,36941936	0,19799007	0,26052613	0,23398695	2,90089887	0,06544832	0,05305013	0,42528445	0,03819427	0,49192149	0,13198995	1,55455321	8,51201091	0,64371768	0,00580898
-99	-99	-99	-99	-99	-99	-99	-99	-99	-99	-99	-99	-99	-99	-99	-99	-99	-99	-99
-99	-99	-99	-99	-99	-99	-99	-99	-99	-99	-99	-99	-99	-99	-99	-99	-99	-99	-99
-99	-99	-99	-99	-99	-99	-99	-99	-99	-99	-99	-99	-99	-99	-99	-99	-99	-99	-99
-99	-99	-99	-99	-99	-99	-99	-99	-99	-99	-99	-99	-99	-99	-99	-99	-99	-99	-99
-99	-99	-99	-99	-99	-99	-99	-99	-99	-99	-99	-99	-99	-99	-99	-99	-99	-99	-99
0,04725956	0,18184107	0,03146191	0,04875689	2,35014537	0,30837335	1,17915224	0,22387262	2,09616168	0,22794379	0,29683847	0,40633738	0,10902644	0,38736084	0,06362555	0,19816169	0,79926732	0,33651670	0,02618256
-99	-99	-99	-99	-99	-99	-99	-99	-99	-99	-99	-99	-99	-99	-99	-99	-99	-99	-99
-99	-99	-99	-99	-99	-99	-99	-99	-99	-99	-99	-99	-99	-99	-99	-99	-99	-99	-99
-99	-99	-99	-99	-99	-99	-99	-99	-99	-99	-99	-99	-99	-99	-99	-99	-99	-99	-99
-99	-99	-99	-99	-99	-99	-99	-99	-99	-99	-99	-99	-99	-99	-99	-99	-99	-99	-99
-99	-99	-99	-99	-99	-99	-99	-99	-99	-99	-99	-99	-99	-99	-99	-99	-99	-99	-99
-99	-99	-99	-99	-99	-99	-99	-99	-99	-99	-99	-99	-99	-99	-99	-99	-99	-99	-99
-99	-99	-99	-99	-99	-99	-99	-99	-99	-99	-99	-99	-99	-99	-99	-99	-99	-99	-99
-99	-99	-99	-99	-99	-99	-99	-99	-99	-99	-99	-99	-99	-99	-99	-99	-99	-99	-99
0,02892579	0,22492252	0,04003762	0,00011521	2,59457817	0,19230924	0,78540027	0,01664808	2,47855625	0,02330042	0,03002899	0,72271563	0,11887358	0,52504028	0,02189128	1,86541937	3,25238940	0,49878846	0,00609778
0,05357672	0,31332895	0,02968237	0,10648706	2,47898579	0,21490188	0,32731463	0,29911040	2,21261126	0,12411505	0,14739403	0,73039561	0,27428545	0,50935733	0,10895090	8,05424858	29,44090266	0,25467866	0,01282187
-99	-99	-99	-99	-99	-99	-99	-99	-99	-99	-99	-99	-99	-99	-99	-99	-99	-99	-99
-99	-99	-99	-99	-99	-99	-99	-99	-99	-99	-99	-99	-99	-99	-99	-99	-99	-99	-99
0,08105247	0,37164575	0,06528974	0,18711537	3,21094955	0,39093482	1,00975130	0,06583506	2,15397275	0,00568352	0,01192390	0,29078001	0,08116491	0,35824041	0,15051717	1,15268635	0,93822120	0,56604945	0,00280264
0,05712436	0,14703688	0,05134081	0,05340986	4,95969017	0,25653899	0,99051450	0,07923326	2,12615975	0,16890103	0,21026054	0,15737191	0,03653062	0,49013463	0,12312964	1,43619336	6,03038453	0,61781637	0,00001769
-99	-99	-99	-99	-99	-99	-99	-99	-99	-99	-99	-99	-99	-99	-99	-99	-99	-99	-99
-99	-99	-99	-99	-99	-99	-99	-99	-99	-99	-99	-99	-99	-99	-99	-99	-99	-99	-99
-99	-99	-99	-99	-99	-99	-99	-99	-99	-99	-99	-99	-99	-99	-99	-99	-99	-99	-99
-99	-99	-99	-99	-99	-99	-99	-99	-99	-99	-99	-99	-99	-99	-99	-99	-99	-99	-99
-99	-99	-99	-99	-99	-99	-99	-99	-99	-99	-99	-99	-99	-99	-99	-99	-99	-99	-99
0,07501121	0,20507876	0,02445461	0,09057458	1,20184430	0,21782629	0,08864911	0,07701322	1,88982153	0,09527556	0,14202827	0,44357501	0,07367148	0,47706244	0,04386609	0,29002517	1,62478632	0,50426360	0,00352600
-99	-99	-99	-99	-99	-99	-99	-99	-99	-99	-99	-99	-99	-99	-99	-99	-99	-99	-99
-99	-99	-99	-99	-99	-99	-99	-99	-99	-99	-99	-99	-99	-99	-99	-99	-99	-99	-99
0,12379286	0,08054841	0,01291329	0,14043749	2,07340220	0,25578437	0,08826590	0,13888859	2,02090289	0,13434365	0,17230064	0,25156446	0,05933388	0,51799138	0,11399165	2,41831135	4,07556479	0,49517144	0,00281237
-99	-99	-99	-99	-99	-99	-99	-99	-99	-99	-99	-99	-99	-99	-99	-99	-99	-99	-99
0,07144615	0,21389876	0,07785859	0,00002974	2,23612370	0,33125175	0,02644246	0,12771501	2,07340220	0,07921953	0,05373478	0,42425395	0,08414375	0,56097174	0,11825721	0,25881623	1,20831784	0,37216131	0,00002974
-99	-99	-99	-99	-99	-99	-99	-99	-99	-99	-99	-99	-99	-99	-99	-99	-99	-99	-99
0,05908763	0,22877479	0,01803571	0,00022497	1,93187266	0,18828631	1,73387513	0,28837140	2,33755450	0,26720150	0,28029152	0,19157750	0,06254334	0,46943587	0,15821957	1,43993132	6,00015596	0,55980644	0,00945308
-99	-99	-99	-99	-99	-99	-99	-99	-99	-99	-99	-99	-99	-99	-99	-99	-99	-99	-99
-99	-99	-99	-99	-99	-99	-99	-99	-99	-99	-99	-99	-99	-99	-99	-99	-99	-99	-99
-99	-99	-99	-99	-99	-99	-99	-99	-99	-99	-99	-99	-99	-99	-99	-99	-99	-99	-99
0,04124892	0,31392678	0,07226796	0,08307165	2,64542589	0,18231435	1,24530575	0,18030359	3,02408774	0,04142082	0,03451817	0,53570104	0,14942568	0,39598015	0,14645202	1,65691547	2,64359285	0,44642818	0,00364087
0,03530460	0,16516730	0,01784916	0,09062168	1,64262140	0,13360075	0,21389876	0,44689258	1,76051803	0,07588718	0,09665579	0,30757284	0,03991293	0,54412122	0,09820906	0,49106980	0,80051481	0,22236475	0,00384980
0,03779266	0,10622903	0,04662508	0,09032380	4,56701016	0,27801796	2,93070956	0,22178751	2,58873986	0,01735205	0,01901458	0,08646440	0,11275396	0,32720121	0,17245000	0,48843890	1,03759972	0,52494930	0,00407566
0,06320796	0,16936997	0,05472144	0,13353131	3,22042256	0,19686110	0,11778682	0,15628487	2,84070693	0,12720700	0,13380927	0,20479466	0,14877974	0,46401714	0,08828120	0,82602079	3,92651661	0,55680729	0,01436050
-99	-99	-99	-99	-99	-99	-99	-99	-99	-99	-99	-99	-99	-99	-99	-99	-99	-99	-99
0,06524450	0,20848276	0,02186853	0,10628427	2,88986080	0,20862732	0,27932178	0,04658470	1,73628046	0,17386025	0,20906160	0,14438572	0,08918841	0,37500975	0,04948037	0,44135150	4,26930135	0,40276215	0,02388088
0,02503343	0,21315872	0,04160786	0,08609063	2,03778239	0,17398080	0,03892929	0,05978801	2,94241349	0,08229793	0,05158159	0,38689125	0,13840807	0,65022007	0,12032431	0,00740133	0,21271593	0,40781875	0,00749425
-99	-99	-99	-99	-99	-99	-99	-99	-99	-99	-99	-99	-99	-99	-99	-99	-99	-99	-99
-99	-99	-99	-99	-99	-99	-99	-99	-99	-99	-99	-99	-99	-99	-99	-99	-99	-99	-99
-99	-99	-99	-99	-99	-99	-99	-99	-99	-99	-99	-99	-99	-99	-99	-99	-99	-99	-99
-99	-99	-99	-99	-99	-99	-99	-99	-99	-99	-99	-99	-99	-99	-99	-99	-99	-99	-99
-99	-99	-99	-99	-99	-99	-99	-99	-99	-99	-99	-99	-99	-99	-99	-99	-99	-99	-99
0,03227364	0,27234362	0,10030740	0,06055962	3,14160108	0,26143061	0,06085417	0,00667276	2,84908764	0,10500282	0,11403116	0,17081422	0,13235641	0,63090732	0,14798266	0,71276579	4,51429281	0,60311290	0,02326814
0,06666161	0,23423036	0,06421259	0,06829861	1,98618499	0,18685616	1,37363623	0,16414014	3,39403161	0,19304378	0,15124926	0,33962157	0,02927880	0,39776824	0,12015762	1,07400447	1,84932656	0,39832005	0,00011032
0,05875071	0,14486189	0,03830695	0,06309852	1,86703633	0,28672707	0,55009397	0,07163210	2,85155725	0,07865869	0,07263204	0,33675003	0,03723363	0,43189425	0,05726312	0,48355436	0,90673319	0,54971281	0,00004514
0,02283673	0,21456698	0,05349323	0,00004837	2,34973816	0,38917744	0,50017332	0,06540297	1,75746992	0,03578503	0,02553288	0,51745310	0,06410141	0,54430983	0,10051620	0,32862178	0,41108254	0,28430276	0,00908373
-99	-99	-99	-99	-99	-99	-99	-99	-99	-99	-99	-99	-99	-99	-99	-99	-99	-99	-99

-99	-99	-99	-99	-99	-99	-99	-99	-99	-99	-99	-99	-99	-99	-99	-99	-99	-99	-99
-99	-99	-99	-99	-99	-99	-99	-99	-99	-99	-99	-99	-99	-99	-99	-99	-99	-99	-99
-99	-99	-99	-99	-99	-99	-99	-99	-99	-99	-99	-99	-99	-99	-99	-99	-99	-99	-99
-99	-99	-99	-99	-99	-99	-99	-99	-99	-99	-99	-99	-99	-99	-99	-99	-99	-99	-99
-99	-99	-99	-99	-99	-99	-99	-99	-99	-99	-99	-99	-99	-99	-99	-99	-99	-99	-99
-99	-99	-99	-99	-99	-99	-99	-99	-99	-99	-99	-99	-99	-99	-99	-99	-99	-99	-99
0,03437492	0,17647063	0,03653695	0,23060569	0,83422011	0,15871386	0,12003275	0,01404792	2,47855625	0,08113679	0,09002690	0,44211697	0,16340228	0,47417779	0,11395215	0,25747424	0,97704709	0,49603025	0,00572503
0,03989910	0,23659640	0,04491995	0,07902758	3,06205458	0,29270109	0,04888379	0,15256543	2,53239070	0,06339443	0,09090478	0,17659299	0,02196728	0,36462869	0,03302037	0,25287565	0,72673435	0,45234643	0,00245085
-99	-99	-99	-99	-99	-99	-99	-99	-99	-99	-99	-99	-99	-99	-99	-99	-99	-99	-99
0,07860419	0,34669766	0,06895266	0,00001243	1,94227839	0,17896508	1,10784036	0,07168177	1,75290766	0,03124459	0,05928250	0,44557803	0,12283129	0,56752270	0,03783198	0,23927640	0,64785821	0,46514422	0,00337885
-99	-99	-99	-99	-99	-99	-99	-99	-99	-99	-99	-99	-99	-99	-99	-99	-99	-99	-99
0,07953590	0,35496532	0,05569725	0,08691506	1,89047661	0,27523770	0,70649439	0,29458380	1,43768738	0,08583739	0,07975673	0,36167104	0,24430410	0,75092983	0,23549201	0,48894700	0,60405424	0,61036748	0,00002541
0,04345000	0,22869552	0,05345616	0,11191691	1,85639022	0,38196173	0,04605495	0,13903307	2,10016110	0,02051041	0,03915933	0,46409755	0,10044656	0,48447697	0,06590355	0,25729583	1,51834538	0,72976304	0,00451049
-99	-99	-99	-99	-99	-99	-99	-99	-99	-99	-99	-99	-99	-99	-99	-99	-99	-99	-99
0,03614024	0,21415837	0,01620688	0,12822505	3,07748111	0,15143284	0,90862065	0,04798575	2,93172544	0,02831578	0,02104688	0,47294686	0,04071629	0,48422518	0,07258172	0,72635665	1,61524163	0,40046554	0,00002740
-99	-99	-99	-99	-99	-99	-99	-99	-99	-99	-99	-99	-99	-99	-99	-99	-99	-99	-99
-99	-99	-99	-99	-99	-99	-99	-99	-99	-99	-99	-99	-99	-99	-99	-99	-99	-99	-99
-99	-99	-99	-99	-99	-99	-99	-99	-99	-99	-99	-99	-99	-99	-99	-99	-99	-99	-99
-99	-99	-99	-99	-99	-99	-99	-99	-99	-99	-99	-99	-99	-99	-99	-99	-99	-99	-99
0,02340970	0,20621912	0,02465886	0,14242260	2,07376153	0,14222530	0,86289086	0,13105552	3,45395671	0,19294345	0,22944973	0,18740742	0,10084769	0,63960362	0,04843689	0,19028714	0,56850699	0,45541383	0,00643096
-99	-99	-99	-99	-99	-99	-99	-99	-99	-99	-99	-99	-99	-99	-99	-99	-99	-99	-99
-99	-99	-99	-99	-99	-99	-99	-99	-99	-99	-99	-99	-99	-99	-99	-99	-99	-99	-99
0,05496854	0,26093276	0,03157114	0,11209159	1,85285502	0,16825835	0,49132515	0,10307383	1,53023158	0,01737010	0,02332062	0,28494394	0,08075806	0,54705205	0,14416072	3,30293825	6,24088229	0,39358578	0,00197730
-99	-99	-99	-99	-99	-99	-99	-99	-99	-99	-99	-99	-99	-99	-99	-99	-99	-99	-99
0,04007232	0,22873515	0,04950610	0,07431257	2,64221891	0,20815787	0,57784321	0,11532283	3,04987476	0,01780900	0,02492522	0,15386639	0,01904756	0,55123905	0,04590356	1,02722342	2,19124379	0,37416590	0,00512139
0,05604580	0,26202021	0,06309852	0,14316493	1,90362592	0,21744915	0,71387827	0,06336149	2,01216693	0,02727551	0,06137309	0,49680445	0,14759850	0,70307476	0,03085718	1,19644162	0,92899968	0,70210076	0,00233680
-99	-99	-99	-99	-99	-99	-99	-99	-99	-99	-99	-99	-99	-99	-99	-99	-99	-99	-99
0,05841570	0,19111329	0,03341176	0,17140725	1,82197100	0,18118055	0,03959604	0,04402601	2,41747337	0,08091214	0,07171904	0,49465686	0,14215138	0,44426734	0,14304094	0,65861168	1,07885427	0,42943152	0,00510278
0,04442452	0,19867744	0,03267883	0,13411105	0,97772456	0,16442482	1,02207425	0,07633561	1,53954069	0,12145552	0,20061481	0,37254846	0,06889294	0,38064024	0,02483468	0,20327434	0,75812098	0,32680456	0,00314113

Table S6 Transcripts derived from a set of marker candidates for drought tolerance [14] and respective eQTL overlapping with DRYM QTL.

Gene product[1]	Transcript ID	LG1[2]		LG2		LG3		LG4		LG5		LG6		LG7		LG8		LG9		LG10		LG11		LG12		Number of overlaps
		1A	1R	2A	2R	3A	3R	4A	4R	5A	5R	6A	6R	7A	7R	8A	8R	9A	9R	10A	10R	11A	11R	12A	12R	
Ethylene-inducing xylanase	PGSC0003DMT400020366					1Ds, 1Co			1Co															2Ds, 2Co	2Ds,1Co	10
Bacterial spot disease resistance protein 4	PGSC0003DMT400080813														1Ds, 1Co								2Ds	2Co		6
Cytochrome P450	PGSC0003DMT400008547		1Ds					1Ds	1Co			1Co			1Co											5
Desacetoxyvindoline 4-hydroxylase	PGSC0003DMT400041989											2Ds, 1Co											1Ds, 1Co			5
Reticuline oxidase	PGSC0003DMT400046308				1Ds, 1Co	1Ds, 1Co						1Co														5
UDP-glucose:glucosyltransferase	PGSC0003DMT400049125		1Ds																				2Ds, 1Co	1Ds		5
O-methyltransferase	PGSC0003DMT400031370					1Ds, 2Co											1Ds, 1Co									5
Acidic class II 1,3-beta-glucanase	PGSC0003DMT400027201		1Ds, 1Co									1Ds			1Ds										1 Ds	5
Beta-D-glucan exohydrolase	PGSC0003DMT400015224											1Ds, 2Co					1Ds									4
Cc-nbs-lrr resistance protein	PGSC0003DMT400049097											2Ds					1Co								1Co	4
Lipoxygenase	PGSC0003DMT400082023	1Co			1Co							1Ds, 1Co														4
(S)-norcoclaurine synthase	PGSC0003DMT400008278								1Co						1Ds, 1Co		1Co									4
Kinase	PGSC0003DMT400042362	1Ds, 1Co	1Co		1Co																					4
NADPH-dependent codeinone reductase	PGSC0003DMT400037483																								2Ds, 2Co	4
Poly(ADP-ribose) glycohydrolase	PGSC0003DMT400075512	1Co				1Co		1Ds									1Co									4
TIR-NBS-LRR disease resistance	PGSC0003DMT400071445																						2Ds, 2Co			4
Glutathione s-transferase	PGSC0003DMT400004539		2Ds, 1Co																							3
PR1 protein	PGSC0003DMT400013094		1Ds					1Ds							1Ds											3
BED finger-nbs-lrr resistance protein	PGSC0003DMT400035714								1Ds																1Ds, 1Co	3
Betaine aldehyde dehyrogenase	PGSC0003DMT400083025											2Ds, 1Co														3
Glucosyltransferase	PGSC0003DMT400021019		1Ds					1Co																	1Co	3
LRR receptor-like serine/threonine-protein kinase	PGSC0003DMT400071885							1Ds	1Ds																1Co	3
Receptor protein kinase	PGSC0003DMT400045689				1Co												1Ds, 1Co									3
S-locus-specific glycoprotein S6	PGSC0003DMT400036026				1Ds, 2Co																					3
SBT4B protein	PGSC0003DMT400017633	1Co	1Ds, 1Co																							3
Fatty acid desaturase	PGSC0003DMT400083859																				1Ds		1Co			2
Glutamyl-tRNA(Gln) amidotransferase subunit A	PGSC0003DMT400008092																1Co						1Ds			2
Extensin	PGSC0003DMT400082012		1Ds, 1Co																							2
Gamma aminobutyrate transaminase isoform1	PGSC0003DMT400030682				1Co										1Ds											2
Hcr9-OR2A	PGSC0003DMT400027792				1Co				1Co																	2
Homoserine dehydrogenase family protein	PGSC0003DMT400048114											1Ds											1Co			2
TMV resistance protein N	PGSC0003DMT400046899		1Co																						1Ds	2
Gene of unknown function	PGSC0003DMT400062379				1Co																					1
Multidrug resistance protein ABC transporter family	PGSC0003DMT400031714				1Co																					1
Serine-threonine protein kinase, plant-type	PGSC0003DMT400068787				1Co																					1
RING finger protein	PGSC0003DMT400066873	1Co																								1
Methionyl-tRNA synthetase	PGSC0003DMT400049172														1Co											1
Nbs-lrr resistance protein	PGSC0003DMT400021608				1Co																					1
70 kDa subunit of replication protein A	PGSC0003DMT400052517											1Co														1
Serine-threonine protein kinase plant-type	PGSC0003DMT400046445											1Ds														1
Flagellin-sensing 2	PGSC0003DMT400068776																									0
Bacterial spot disease resistance protein 4	PGSC0003DMT400006231																									0
Serine-threonine protein kinase, plant-type	PGSC0003DMT400028434																									0
																										127

[1]Gene products are sorted according to the number of eQTL overlapping with DRYM QTL in descending order.

[2]grey, no DRYM QTL on the linkage group (LG); Co, overlapping eQTL under control conditions; Ds, overlapping eQTL under drought stress

Table S7 Metabolite data used for mQTL analysis in the F1-popuation A×R.

Normalised metabolite data for 15 metabolites were used from two trials, 61711 (MPI Golm foil green house) and 68015 (JKI shelter), in the year 2014 measuring 95 A×R lines. For details of the drought experiments s Haas et al. [38].

Original intensities of each metabolite were normalized to the average original intensity (response) of all annotated analytes in a sample and log10-transformed.

Transformed data were corrected for analytical batch and sequence effects according to the procedure described in the methods section (missing data are given as -99).

	1	2	3	4	5	6	7	8	9	10	11	12	13	14	15
**Location_ Treatment**	JKI_S_14_ Co _M1	JKI_S_14_Co _M2	JKI_S_14_Co _M3	JKI_S_14_Co _M4	JKI_S_14_Co _M5	JKI_S_14_Co _M6	JKI_S_14_Co _M7	JKI_S_14_Co_ M8	JKI_S_14_Co _M9	JKI_S_14_Co _M10	JKI_S_14_Co _M11	JKI_S_14_Co _M12	JKI_S_14_Co _M13	JKI_S_14_Co _M14	JKI_S_14_Co _M15
**Metabolites F1 individuals**	Salicylic acid-glucopyranoside	A148006-10:	Threonic acic	Ribitol	Malic acid	A250002-10:	Galactinol	Fumaric acid	Quinic acid, 3-caffeoyl-, trans-	A237001-10:	A217004-10:	A174001-10:	A197007-101	Raffinose	Galactaric acid
1	3,523756631	4,6111517	5,2237315	4,3713766	4,9330216	3,523391	5,3191509	3,636046047	5,5623605	4,2329113	3,932992	3,9238031	5,100347038	4,284504786	3,71684058
2	-99	-99	-99	-99	-99	-99	-99	-99	-99	-99	-99	-99	-99	-99	-99
3	-99	-99	-99	-99	-99	-99	-99	-99	-99	-99	-99	-99	-99	-99	-99
4	-99	-99	-99	-99	-99	-99	-99	-99	-99	-99	-99	-99	-99	-99	-99
5	-99	-99	-99	-99	-99	-99	-99	-99	-99	-99	-99	-99	-99	-99	-99
6	3,270801713	4,1616793	5,3819443	4,267251	5,4008856	3,5846793	5,3730833	3,183000807	5,4692411	4,0951212	3,9212923	4,3244119	5,260709869	4,359004222	3,10067756
7	-99	-99	-99	-99	-99	-99	-99	-99	-99	-99	-99	-99	-99	-99	-99
8	-99	-99	-99	-99	-99	-99	-99	-99	-99	-99	-99	-99	-99	-99	-99
9	-99	-99	-99	-99	-99	-99	-99	-99	-99	-99	-99	-99	-99	-99	-99
10	-99	-99	-99	-99	-99	-99	-99	-99	-99	-99	-99	-99	-99	-99	-99
11	-99	-99	-99	-99	-99	-99	-99	-99	-99	-99	-99	-99	-99	-99	-99
12	-99	-99	-99	-99	-99	-99	-99	-99	-99	-99	-99	-99	-99	-99	-99
13	-99	-99	-99	-99	-99	-99	-99	-99	-99	-99	-99	-99	-99	-99	-99
14	-99	-99	-99	-99	-99	-99	-99	-99	-99	-99	-99	-99	-99	-99	-99
15	-99	-99	-99	-99	-99	-99	-99	-99	-99	-99	-99	-99	-99	-99	-99
16	3,358540777	4,6162578	5,3472665	4,4105694	5,0655159	3,1533917	5,414502	-99	5,5624232	4,2769207	3,8652963	4,3557531	5,312731998	4,159658424	-99
17	3,805128221	4,6203074	5,268362	4,4198271	5,3543384	3,8035553	5,3090332	3,889427644	5,5604136	3,9898686	3,8681165	4,3490551	5,327882196	4,536273724	2,50159614
18	3,590365221	3,7766584	4,3955902	4,3214129	5,3075298	3,8042149	5,4467285	4,052723499	5,5606238	4,4740371	4,0962991	4,6247871	5,421369648	4,772790273	3,76927919
19	3,132072197	3,9956802	5,3666221	4,2155142	5,6809771	3,6335607	5,2471293	3,791572049	5,4364674	4,1604376	4,1121447	4,1724518	5,329172064	4,278498608	2,57383180
20	-99	-99	-99	-99	-99	-99	-99	-99	-99	-99	-99	-99	-99	-99	-99
21	-99	-99	-99	-99	-99	-99	-99	-99	-99	-99	-99	-99	-99	-99	-99
22	-99	-99	-99	-99	-99	-99	-99	-99	-99	-99	-99	-99	-99	-99	-99
23	-99	-99	-99	-99	-99	-99	-99	-99	-99	-99	-99	-99	-99	-99	-99
24	3,351951026	3,8366873	4,5052389	4,4981639	5,5610417	3,4774979	5,4177185	3,893986568	5,3999416	4,0846817	4,0928812	4,3526347	5,316545534	5,211168354	-99
25	-99	-99	-99	-99	-99	-99	-99	-99	-99	-99	-99	-99	-99	-99	-99
26	-99	-99	-99	-99	-99	-99	-99	-99	-99	-99	-99	-99	-99	-99	-99
27	-99	-99	-99	-99	-99	-99	-99	-99	-99	-99	-99	-99	-99	-99	-99
28	-99	-99	-99	-99	-99	-99	-99	-99	-99	-99	-99	-99	-99	-99	-99
29	-99	-99	-99	-99	-99	-99	-99	-99	-99	-99	-99	-99	-99	-99	-99
30	-99	-99	-99	-99	-99	-99	-99	-99	-99	-99	-99	-99	-99	-99	-99
31	-99	-99	-99	-99	-99	-99	-99	-99	-99	-99	-99	-99	-99	-99	-99
32	-99	-99	-99	-99	-99	-99	-99	-99	-99	-99	-99	-99	-99	-99	-99
33	2,60949963	3,7700197	4,4851126	4,3923028	5,805428	3,4359141	5,3244596	4,086912894	5,5034452	3,9745764	4,0323335	4,5227028	5,367076454	5,018731999	3,20704516
34	-99	-99	-99	-99	-99	-99	-99	-99	-99	-99	-99	-99	-99	-99	-99
35	3,27863018	3,7523179	4,4319899	4,4181758	5,4924391	3,0109957	5,4390724	3,77405822	5,4797291	4,1128929	4,0982089	4,3195291	5,35938039	4,966548968	-99
36	-99	-99	-99	-99	-99	-99	-99	-99	-99	-99	-99	-99	-99	-99	-99
37	-99	-99	-99	-99	-99	-99	-99	-99	-99	-99	-99	-99	-99	-99	-99
38	-99	-99	-99	-99	-99	-99	-99	-99	-99	-99	-99	-99	-99	-99	-99
39	-99	-99	-99	-99	-99	-99	-99	-99	-99	-99	-99	-99	-99	-99	-99
40	3,3134191	3,6745589	4,1834906	4,464015	5,8497834	3,3169262	5,3588433	3,967168487	5,4329489	4,3377863	4,0526384	4,4005219	5,386273067	4,804435115	-99
41	-99	-99	-99	-99	-99	-99	-99	-99	-99	-99	-99	-99	-99	-99	-99
42	-99	-99	-99	-99	-99	-99	-99	-99	-99	-99	-99	-99	-99	-99	-99
43	3,549804515	3,8924204	4,5859355	4,5693371	5,4287988	3,4685513	5,3968678	3,248160533	5,5662486	4,062772	3,9915	4,1423178	5,422271656	3,559951528	3,21665145
44	-99	-99	-99	-99	-99	-99	-99	-99	-99	-99	-99	-99	-99	-99	-99
45	-99	-99	-99	-99	-99	-99	-99	-99	-99	-99	-99	-99	-99	-99	-99
46	3,363839962	4,0017006	4,7642571	4,221074	5,6912805	3,2551889	5,3322829	3,840595698	5,5617571	4,193226	3,992499	4,3460532	5,316655644	4,669926978	-99
47	-99	-99	-99	-99	-99	-99	-99	-99	-99	-99	-99	-99	-99	-99	-99
48	-99	-99	-99	-99	-99	-99	-99	-99	-99	-99	-99	-99	-99	-99	-99
49	3,199799365	3,6508301	4,1774486	4,3287916	5,700717	3,6019622	5,4072733	4,121275367	5,4227374	3,9643452	3,938022	4,2257799	5,27365917	4,788891051	3,64862551
50	3,441168	4,019138	4,6396079	4,3982618	5,4361978	3,0121851	5,3305542	3,870563237	5,4733623	4,2349181	4,01001	4,1734238	5,250298457	4,902812732	-99
51	3,169712731	4,3763368	5,1277995	4,101345	5,7834319	3,6171777	5,3392347	3,813654553	5,368867	3,8110683	4,0575736	4,2781907	5,383158545	4,993237016	-99
52	-99	-99	-99	-99	-99	-99	-99	-99	-99	-99	-99	-99	-99	-99	-99
53	-99	-99	-99	-99	-99	-99	-99	-99	-99	-99	-99	-99	-99	-99	-99
54	-99	-99	-99	-99	-99	-99	-99	-99	-99	-99	-99	-99	-99	-99	-99
55	-99	-99	-99	-99	-99	-99	-99	-99	-99	-99	-99	-99	-99	-99	-99
56	-99	-99	-99	-99	-99	-99	-99	-99	-99	-99	-99	-99	-99	-99	-99
57	3,454831044	4,4526325	5,0303728	4,194795	4,8886261	3,4347472	5,3318987	-99	5,5540488	4,1070007	3,9228349	3,9301175	5,121705954	3,535652281	3,60393997
58	3,475074758	4,0878779	4,5875765	4,275685	5,3017697	3,4072908	5,4383084	3,183235374	5,5619146	4,1979831	4,0714137	3,8341029	5,125125722	4,828325921	-99
59	-99	-99	-99	-99	-99	-99	-99	-99	-99	-99	-99	-99	-99	-99	-99
60	-99	-99	-99	-99	-99	-99	-99	-99	-99	-99	-99	-99	-99	-99	-99
61	3,467438061	4,0380021	4,6824654	4,5170984	5,2742923	3,5239949	5,3653546	3,937617971	5,5981183	4,4103404	3,9351846	4,3455697	5,287365287	4,194918273	3,61244365
62	-99	-99	-99	-99	-99	-99	-99	-99	-99	-99	-99	-99	-99	-99	-99
63	-99	-99	-99	-99	-99	-99	-99	-99	-99	-99	-99	-99	-99	-99	-99
64	-99	-99	-99	-99	-99	-99	-99	-99	-99	-99	-99	-99	-99	-99	-99
65	3,151914557	3,7599115	4,4377611	4,4961887	5,3951008	3,4013486	5,3555011	3,270320986	5,4670673	4,0516413	4,0056349	4,1560549	5,347848744	5,09716121	-99
66	-99	-99	-99	-99	-99	-99	-99	-99	-99	-99	-99	-99	-99	-99	-99
67	-99	-99	-99	-99	-99	-99	-99	-99	-99	-99	-99	-99	-99	-99	-99
68	-99	-99	-99	-99	-99	-99	-99	-99	-99	-99	-99	-99	-99	-99	-99
69	-99	-99	-99	-99	-99	-99	-99	-99	-99	-99	-99	-99	-99	-99	-99
70	-99	-99	-99	-99	-99	-99	-99	-99	-99	-99	-99	-99	-99	-99	-99
71	-99	-99	-99	-99	-99	-99	-99	-99	-99	-99	-99	-99	-99	-99	-99
72	-99	-99	-99	-99	-99	-99	-99	-99	-99	-99	-99	-99	-99	-99	-99
73	-99	-99	-99	-99	-99	-99	-99	-99	-99	-99	-99	-99	-99	-99	-99
74	-99	-99	-99	-99	-99	-99	-99	-99	-99	-99	-99	-99	-99	-99	-99
75	3,21206344	3,8361342	4,5771959	4,3427154	5,6416033	3,5842647	5,3590742	3,934368978	5,4711725	4,2496116	4,0493768	4,43534	5,313707874	4,305027487	-99
76	-99	-99	-99	-99	-99	-99	-99	-99	-99	-99	-99	-99	-99	-99	-99
77	-99	-99	-99	-99	-99	-99	-99	-99	-99	-99	-99	-99	-99	-99	-99
78	-99	-99	-99	-99	-99	-99	-99	-99	-99	-99	-99	-99	-99	-99	-99
79	-99	-99	-99	-99	-99	-99	-99	-99	-99	-99	-99	-99	-99	-99	-99
80	-99	-99	-99	-99	-99	-99	-99	-99	-99	-99	-99	-99	-99	-99	-99
81	-99	-99	-99	-99	-99	-99	-99	-99	-99	-99	-99	-99	-99	-99	-99
82	-99	-99	-99	-99	-99	-99	-99	-99	-99	-99	-99	-99	-99	-99	-99
83	-99	-99	-99	-99	-99	-99	-99	-99	-99	-99	-99	-99	-99	-99	-99
84	3,272819879	3,8556513	4,53473	4,3675035	4,9158654	3,6967345	5,4409775	3,784481756	5,5100632	4,2272442	4,0470335	4,2917174	5,25971967	4,317430415	-99
85	-99	-99	-99	-99	-99	-99	-99	-99	-99	-99	-99	-99	-99	-99	-99
86	-99	-99	-99	-99	-99	-99	-99	-99	-99	-99	-99	-99	-99	-99	-99
87	2,813722452	3,9137207	4,4950175	4,3217537	5,6264953	3,3797555	5,4320483	3,338930028	5,4532777	3,3954551	3,9664841	4,1278006	5,034631951	4,598309117	-99
88	3,22600624	3,6196767	4,3524559	4,4414938	5,1264854	3,6697931	5,4313896	3,921026582	5,5093408	4,1566056	3,9839655	4,2963098	5,322321706	4,514760878	3,16646119
89	3,456322747	3,6823358	4,2885511	4,3516185	5,4093049	3,4801017	5,4995212	3,9121248	5,5574805	4,6862485	3,9578522	4,2320921	5,152723757	4,254896404	-99
90	-99	-99	-99	-99	-99	-99	-99	-99	-99	-99	-99	-99	-99	-99	-99
91	-99	-99	-99	-99	-99	-99	-99	-99	-99	-99	-99	-99	-99	-99	-99
92	-99	-99	-99	-99	-99	-99	-99	-99	-99	-99	-99	-99	-99	-99	-99
93	-99	-99	-99	-99	-99	-99	-99	-99	-99	-99	-99	-99	-99	-99	-99
94	-99	-99	-99	-99	-99	-99	-99	-99	-99	-99	-99	-99	-99	-99	-99
95	-99	-99	-99	-99	-99	-99	-99	-99	-99	-99	-99	-99	-99	-99	-99
96	-99	-99	-99	-99	-99	-99	-99	-99	-99	-99	-99	-99	-99	-99	-99
97	-99	-99	-99	-99	-99	-99	-99	-99	-99	-99	-99	-99	-99	-99	-99
98	-99	-99	-99	-99	-99	-99	-99	-99	-99	-99	-99	-99	-99	-99	-99
99	2,975964089	4,0812889	4,8176922	4,1483092	5,7304661	3,0762218	5,4572029	3,956942359	5,4058268	4,0161561	3,9946982	4,1237972	5,279434489	4,736749949	-99
100	3,300127977	4,1876105	4,2711695	4,4172364	4,6337326	3,9052592	5,3643687	2,690657793	5,4123984	3,9847793	4,1171919	3,6349791	5,221920502	4,686754857	-99

101	-99	-99	-99	-99	-99	-99	-99	-99	-99	-99	-99	-99	-99	-99	-99
102	-99	-99	-99	-99	-99	-99	-99	-99	-99	-99	-99	-99	-99	-99	-99
103	3,335924142	4,6473429	5,257595	4,2773728	5,8041601	3,2107132	5,3799425	3,989020474	5,5062068	3,9591663	3,7720372	4,2293269	5,301134366	4,316421536	2,94437523
104	3,322616125	3,8575792	4,4960506	4,3387206	5,7966975	3,4646745	5,3801303	3,931994424	5,4218568	3,865661	3,8887974	4,367985	5,306263497	4,345089767	-99
105	-99	-99	-99	-99	-99	-99	-99	-99	-99	-99	-99	-99	-99	-99	-99
106	3,164727642	4,1547038	5,299735	4,4827227	5,786179	3,3957903	5,3568847	3,947809644	5,4668342	4,0893887	3,9950486	4,5172982	5,391691893	4,098864192	3,56320235
107	3,415108207	3,7095619	4,4325006	4,4346545	5,5059917	3,4994427	5,3257735	3,821911012	5,5153768	4,355747	4,0231203	4,2378283	5,246668358	4,437085832	-99
108	-99	-99	-99	-99	-99	-99	-99	-99	-99	-99	-99	-99	-99	-99	-99
109	-99	-99	-99	-99	-99	-99	-99	-99	-99	-99	-99	-99	-99	-99	-99
110	3,171889745	4,307831	5,0416063	4,2000434	5,8586566	3,5848386	5,3695177	4,067579913	5,5099989	4,085688	3,7865163	4,0820957	4,94393268	4,846288179	-99
111	-99	-99	-99	-99	-99	-99	-99	-99	-99	-99	-99	-99	-99	-99	-99
112	-99	-99	-99	-99	-99	-99	-99	-99	-99	-99	-99	-99	-99	-99	-99
113	-99	-99	-99	-99	-99	-99	-99	-99	-99	-99	-99	-99	-99	-99	-99
114	3,368895856	4,7680973	5,2964623	4,2132201	4,8660619	3,3324041	5,3855052	3,728178244	5,5220253	4,0787261	3,8705312	4,0543796	5,10331184	4,685233872	-99
115	-99	-99	-99	-99	-99	-99	-99	-99	-99	-99	-99	-99	-99	-99	-99
116	-99	-99	-99	-99	-99	-99	-99	-99	-99	-99	-99	-99	-99	-99	-99
117	-99	-99	-99	-99	-99	-99	-99	-99	-99	-99	-99	-99	-99	-99	-99
118	-99	-99	-99	-99	-99	-99	-99	-99	-99	-99	-99	-99	-99	-99	-99
119	-99	-99	-99	-99	-99	-99	-99	-99	-99	-99	-99	-99	-99	-99	-99
120	3,139981412	4,2695812	5,432275	4,4618399	5,569986	3,1379769	5,3557609	3,871615956	5,5461124	3,9886065	3,8751159	4,0219113	5,236201497	4,659759877	-99
121	3,454096086	3,7341455	4,3596895	4,2906419	4,4304326	3,5925022	5,365223	3,129258489	5,5429745	4,2038059	4,0724186	4,1213319	5,118476742	4,940908969	-99
122	3,37034418	3,6974609	4,2527541	4,4889638	5,5183541	3,5676726	5,4052929	4,184719472	5,5412994	4,0496262	3,8936796	3,9649635	5,159619999	4,984928624	3,05503151
123	-99	-99	-99	-99	-99	-99	-99	-99	-99	-99	-99	-99	-99	-99	-99
124	-99	-99	-99	-99	-99	-99	-99	-99	-99	-99	-99	-99	-99	-99	-99
125	-99	-99	-99	-99	-99	-99	-99	-99	-99	-99	-99	-99	-99	-99	-99
126	-99	-99	-99	-99	-99	-99	-99	-99	-99	-99	-99	-99	-99	-99	-99
127	3,310204106	3,8493481	4,4779168	4,4899371	5,6269581	4,1380639	5,4039669	3,990285978	5,4812085	4,253975	4,0144062	4,375973	5,327738594	5,025123577	3,75980532
128	-99	-99	-99	-99	-99	-99	-99	-99	-99	-99	-99	-99	-99	-99	-99
129	-99	-99	-99	-99	-99	-99	-99	-99	-99	-99	-99	-99	-99	-99	-99
130	-99	-99	-99	-99	-99	-99	-99	-99	-99	-99	-99	-99	-99	-99	-99
131	-99	-99	-99	-99	-99	-99	-99	-99	-99	-99	-99	-99	-99	-99	-99
132	-99	-99	-99	-99	-99	-99	-99	-99	-99	-99	-99	-99	-99	-99	-99
133	-99	-99	-99	-99	-99	-99	-99	-99	-99	-99	-99	-99	-99	-99	-99
134	-99	-99	-99	-99	-99	-99	-99	-99	-99	-99	-99	-99	-99	-99	-99
135	-99	-99	-99	-99	-99	-99	-99	-99	-99	-99	-99	-99	-99	-99	-99
136	-99	-99	-99	-99	-99	-99	-99	-99	-99	-99	-99	-99	-99	-99	-99
137	-99	-99	-99	-99	-99	-99	-99	-99	-99	-99	-99	-99	-99	-99	-99
138	3,150927165	3,8358259	4,4543878	4,3816931	5,5516825	3,2465476	5,3645658	3,659941424	5,5408311	4,1566629	3,9920728	4,3681638	5,220395111	4,291003235	2,61815315
139	-99	-99	-99	-99	-99	-99	-99	-99	-99	-99	-99	-99	-99	-99	-99
140	3,314702938	4,0451884	4,6284762	4,5019653	5,6013301	3,3077173	5,3873922	3,802434302	5,4816311	4,1811453	3,977501	4,2415683	5,305123785	3,84318608	-99
141	3,275195686	3,8820694	4,585837	4,466272	5,6790723	3,7222403	5,4501951	3,928194156	5,5008984	4,1294978	4,0301605	4,2098273	5,28191258	4,678230452	3,08327994
142	3,271733551	4,3056262	4,8303676	4,2083791	5,8181161	3,5011082	5,2693744	4,313297797	5,5300876	4,1584028	3,8333687	4,0494431	5,070497539	4,578249989	3,00181321
143	3,545212812	3,9069518	5,2254301	4,3143792	5,4233427	3,7513277	5,3295191	3,619695433	5,5596415	4,2007048	3,9380451	4,0101329	5,229938423	4,673462961	2,88337758
144	-99	-99	-99	-99	-99	-99	-99	-99	-99	-99	-99	-99	-99	-99	-99
145	3,361702474	3,8770323	5,2715368	4,3290978	5,4463383	3,0913819	5,2977928	3,922970687	5,558937	4,3595084	3,8385072	4,1865306	5,276227089	4,509725641	3,29162860
146	-99	-99	-99	-99	-99	-99	-99	-99	-99	-99	-99	-99	-99	-99	-99
147	3,636216267	3,7200794	4,397921	4,3267793	5,4059028	3,8979266	5,392359	3,774414924	5,5590624	4,2184296	4,0813786	4,4589511	5,29225381	4,786818228	3,00678045
148	-99	-99	-99	-99	-99	-99	-99	-99	-99	-99	-99	-99	-99	-99	-99
149	-99	-99	-99	-99	-99	-99	-99	-99	-99	-99	-99	-99	-99	-99	-99
150	-99	-99	-99	-99	-99	-99	-99	-99	-99	-99	-99	-99	-99	-99	-99
151	-99	-99	-99	-99	-99	-99	-99	-99	-99	-99	-99	-99	-99	-99	-99
152	3,462413194	3,8834366	4,5064244	4,5484998	5,5684606	3,3514492	5,4071637	3,643498412	5,4718797	3,9566544	3,908802	4,3769515	5,382322444	4,993270399	3,41534741
153	-99	-99	-99	-99	-99	-99	-99	-99	-99	-99	-99	-99	-99	-99	-99
154	-99	-99	-99	-99	-99	-99	-99	-99	-99	-99	-99	-99	-99	-99	-99
155	-99	-99	-99	-99	-99	-99	-99	-99	-99	-99	-99	-99	-99	-99	-99
156	-99	-99	-99	-99	-99	-99	-99	-99	-99	-99	-99	-99	-99	-99	-99
157	-99	-99	-99	-99	-99	-99	-99	-99	-99	-99	-99	-99	-99	-99	-99
158	-99	-99	-99	-99	-99	-99	-99	-99	-99	-99	-99	-99	-99	-99	-99
159	2,995730962	3,7825018	4,5123864	4,3094735	5,6088083	3,8812505	5,5213115	4,021563743	5,4080689	4,1217575	3,9701374	4,5271375	5,310169314	3,736050125	-99
160	3,12729086	3,8984296	4,4772618	4,3345203	5,6388229	3,5198289	5,3229988	3,821314919	5,5442442	4,0396407	3,9660218	4,2348822	5,224773476	4,526845113	3,18604876
161	3,126039729	3,785164	4,4873587	4,26575	5,6613618	3,9014824	5,477934	3,904471768	5,4039814	4,1215059	3,9516945	4,4245419	5,364213144	3,623701931	3,02576258
162	-99	-99	-99	-99	-99	-99	-99	-99	-99	-99	-99	-99	-99	-99	-99
163	-99	-99	-99	-99	-99	-99	-99	-99	-99	-99	-99	-99	-99	-99	-99
164	2,957733044	4,3704959	5,2232396	4,46271	5,6771251	3,5366991	5,6332198	4,022172366	5,4045708	4,0915288	4,0997591	4,5716238	5,304186692	4,647406287	-99
165	-99	-99	-99	-99	-99	-99	-99	-99	-99	-99	-99	-99	-99	-99	-99
166	-99	-99	-99	-99	-99	-99	-99	-99	-99	-99	-99	-99	-99	-99	-99
167	3,571837264	3,5175489	4,1764295	4,4398689	5,7111955	3,7249945	5,3353071	3,770576005	5,5286252	4,1045397	3,9708921	4,2256288	5,235805798	4,675575802	-99
168	-99	-99	-99	-99	-99	-99	-99	-99	-99	-99	-99	-99	-99	-99	-99
169	-99	-99	-99	-99	-99	-99	-99	-99	-99	-99	-99	-99	-99	-99	-99
170	-99	-99	-99	-99	-99	-99	-99	-99	-99	-99	-99	-99	-99	-99	-99
171	3,41622904	4,1505211	5,4733447	4,2732363	5,1382239	3,6828404	5,4007937	3,592051354	5,5602813	4,382389	3,8886958	4,1936768	5,300669426	4,6064409	-99
172	3,129621501	3,8782891	4,6394444	4,4267202	4,3556476	3,6124403	5,3051203	-99	5,5942263	4,0483086	3,5405124	4,1556868	5,286574639	5,155068478	2,57466098
173	-99	-99	-99	-99	-99	-99	-99	-99	-99	-99	-99	-99	-99	-99	-99
174	3,417789288	3,9481231	5,4476514	4,3471643	4,7918331	3,6494968	5,3287994	3,013931286	5,5613926	4,1113678	3,9665895	3,8040312	5,08704518	4,851544167	-99
175	3,482890905	4,0594717	4,6082126	4,4158748	5,2871162	3,516683	5,428712	3,33389183	5,5146317	4,2173722	3,830925	4,1083541	5,029618208	4,196625005	-99
176	2,848029542	3,5376963	4,1400073	4,1911755	5,9238814	3,4344237	5,4705284	4,011617423	5,3740919	4,3805179	4,0857289	4,7926936	5,384713152	4,589476572	-99
177	3,507052413	3,7920792	4,2854556	4,3325104	5,4015563	3,4608072	5,3640812	3,420637243	5,5197605	4,2363181	4,0589004	4,352173	5,339684085	4,405351679	2,57061952
178	-99	-99	-99	-99	-99	-99	-99	-99	-99	-99	-99	-99	-99	-99	-99
179	-99	-99	-99	-99	-99	-99	-99	-99	-99	-99	-99	-99	-99	-99	-99
180	-99	-99	-99	-99	-99	-99	-99	-99	-99	-99	-99	-99	-99	-99	-99
181	-99	-99	-99	-99	-99	-99	-99	-99	-99	-99	-99	-99	-99	-99	-99
182	3,269641402	3,8981089	4,6623207	4,0519579	5,8522676	3,2573179	5,3344447	4,09937017	5,5584338	3,5714789	3,9390695	4,1481196	4,999182034	4,361307016	-99
183	3,435142163	3,7505248	4,3814549	4,2605423	4,8411744	3,749766	5,4353248	-99	5,5950437	3,9116797	-99	4,0297513	5,105611714	4,631642378	-99
184	-99	-99	-99	-99	-99	-99	-99	-99	-99	-99	-99	-99	-99	-99	-99
185	-99	-99	-99	-99	-99	-99	-99	-99	-99	-99	-99	-99	-99	-99	-99
186	-99	-99	-99	-99	-99	-99	-99	-99	-99	-99	-99	-99	-99	-99	-99
187	-99	-99	-99	-99	-99	-99	-99	-99	-99	-99	-99	-99	-99	-99	-99
188	-99	-99	-99	-99	-99	-99	-99	-99	-99	-99	-99	-99	-99	-99	-99
189	3,146221316	3,9636701	4,6728263	4,2648852	5,2848861	3,1745559	5,3316323	3,858776189	5,5485397	4,0384407	4,1224655	4,5003126	5,373585866	4,308368706	3,39951383
190	-99	-99	-99	-99	-99	-99	-99	-99	-99	-99	-99	-99	-99	-99	-99
191	-99	-99	-99	-99	-99	-99	-99	-99	-99	-99	-99	-99	-99	-99	-99
192	-99	-99	-99	-99	-99	-99	-99	-99	-99	-99	-99	-99	-99	-99	-99
193	-99	-99	-99	-99	-99	-99	-99	-99	-99	-99	-99	-99	-99	-99	-99
194	-99	-99	-99	-99	-99	-99	-99	-99	-99	-99	-99	-99	-99	-99	-99
195	-99	-99	-99	-99	-99	-99	-99	-99	-99	-99	-99	-99	-99	-99	-99
196	-99	-99	-99	-99	-99	-99	-99	-99	-99	-99	-99	-99	-99	-99	-99
197	-99	-99	-99	-99	-99	-99	-99	-99	-99	-99	-99	-99	-99	-99	-99
198	3,374764786	4,0521302	5,3676134	4,3125272	5,6076181	3,6908335	5,2842799	4,104404594	5,5460511	4,2974477	3,9860188	4,4219963	5,321954952	4,956453452	-99
199	3,656278546	4,0080336	4,59999	4,2648097	4,8848827	3,5666443	5,397323	3,67753329	5,5663263	4,4198137	3,8585115	4,3163189	5,074442989	3,693290144	2,87220782
200	-99	-99	-99	-99	-99	-99	-99	-99	-99	-99	-99	-99	-99	-99	-99
201	-99	-99	-99	-99	-99	-99	-99	-99	-99	-99	-99	-99	-99	-99	-99
202	3,170357689	3,7314344	4,3435236	4,5504708	5,482463	3,0870292	5,3737119	3,715415186	5,5017248	4,2666595	4,1731427	4,2386248	5,342241423	4,766341318	2,47808516
203	3,10733387	4,5514559	5,2899083	4,1983226	5,3206847	3,6492087	5,3456042	3,380841739	5,5048468	3,8378003	3,892388	4,1270109	5,25244429	4,54735651	-99
204	-99	-99	-99	-99	-99	-99	-99	-99	-99	-99	-99	-99	-99	-99	-99
205	-99	-99	-99	-99	-99	-99	-99	-99	-99	-99	-99	-99	-99	-99	-99
206	-99	-99	-99	-99	-99	-99	-99	-99	-99	-99	-99	-99	-99	-99	-99
207	-99	-99	-99	-99	-99	-99	-99	-99	-99	-99	-99	-99	-99	-99	-99
208	-99	-99	-99	-99	-99	-99	-99	-99	-99	-99	-99	-99	-99	-99	-99
209	3,262150288	4,0614673	4,8579186	4,5233576	5,5844158	3,9183923	5,4719721	3,468347964	5,3285837	3,9941249	4,1188855	4,2210224	5,177815999	4,933679374	2,73831173
210	-99	-99	-99	-99	-99	-99	-99	-99	-99	-99	-99	-99	-99	-99	-99
211	-99	-99	-99	-99	-99	-99	-99	-99	-99	-99	-99	-99	-99	-99	-99
212	3,538209295	4,4073488	5,1611313	4,15172	5,0594199	3,5123696	5,3419353	3,429148555	5,56192	4,2056914	4,0125908	4,1021546	5,175646572	4,667829353	3,51914437

213	-99	-99	-99	-99	-99	-99	-99	-99	-99	-99	-99	-99	-99	-99	-99
214	3,656711916	3,8728097	4,4695628	4,5111731	5,7875595	4,0461335	5,3885034	3,981708026	5,5423654	4,0449354	4,1184416	4,492371	5,362687192	4,471820615	2,62754656
215	-99	-99	-99	-99	-99	-99	-99	-99	-99	-99	-99	-99	-99	-99	-99
216	3,098599923	3,751285	4,4750949	4,2895433	5,7164678	3,2097842	5,4935923	3,835009927	5,4909983	4,3392695	4,0403675	4,3675513	5,326806277	4,799774555	2,99933225
217	-99	-99	-99	-99	-99	-99	-99	-99	-99	-99	-99	-99	-99	-99	-99
218	-99	-99	-99	-99	-99	-99	-99	-99	-99	-99	-99	-99	-99	-99	-99
219	-99	-99	-99	-99	-99	-99	-99	-99	-99	-99	-99	-99	-99	-99	-99
220	3,332660861	3,623408	4,2470117	4,3428052	5,045989	3,6304539	5,3835358	3,549342811	5,5469991	4,2437836	4,1648067	3,9568252	5,308392373	4,348477125	2,86794499
221	3,161905631	4,0541713	4,7615853	4,2652036	5,5869192	3,1816852	5,3845577	3,868359515	5,5351194	4,1308276	3,9993686	4,2536601	5,297778835	4,189902747	3,32987387
222	3,246280093	3,904527	5,3731047	4,4895488	5,3689187	3,410578	5,3490238	3,838575633	5,5680443	4,3003139	3,9062098	3,9321689	5,051776182	4,301372557	2,84722569
223	3,539176632	3,9667365	5,4114854	4,1270336	5,2764578	3,5222986	5,4155651	3,424943471	5,5698139	4,1809663	3,9357765	4,2380323	5,305142873	4,410445204	2,72226367
224	-99	-99	-99	-99	-99	-99	-99	-99	-99	-99	-99	-99	-99	-99	-99
225	3,530317333	3,968139	4,6950093	4,4823016	5,3356648	3,6603296	5,429348	3,438099148	5,5640812	4,0751109	4,0898822	4,419969	5,382076838	4,272396931	-99
226	3,583915866	3,7137595	4,2851457	4,6023518	5,6700802	3,4645564	5,2947856	3,780741156	5,554594	3,9132838	3,9804394	4,0925408	5,259371337	4,96790788	-99
227	-99	-99	-99	-99	-99	-99	-99	-99	-99	-99	-99	-99	-99	-99	-99
228	-99	-99	-99	-99	-99	-99	-99	-99	-99	-99	-99	-99	-99	-99	-99
229	-99	-99	-99	-99	-99	-99	-99	-99	-99	-99	-99	-99	-99	-99	-99
230	-99	-99	-99	-99	-99	-99	-99	-99	-99	-99	-99	-99	-99	-99	-99
231	-99	-99	-99	-99	-99	-99	-99	-99	-99	-99	-99	-99	-99	-99	-99
232	3,280921219	3,8402011	4,2797674	4,5047835	5,6509333	3,3326448	5,4254634	3,896299495	5,4323203	3,6934383	3,9555833	4,0437	5,227355717	4,870987341	-99
233	3,340246172	3,7732313	4,6259335	4,4912484	5,0326136	3,3133741	5,4618302	3,293482953	5,5836606	4,458845	4,129581	4,4220014	5,345972425	4,730113757	2,96519589
234	3,397963285	3,9356889	4,6545863	4,3138876	5,5358604	3,5883657	5,313101	3,786673382	5,5396794	4,2853613	3,9648713	4,1535301	5,254081619	4,489229591	3,32690280
235	3,350957359	4,0079252	4,6233614	4,4159009	5,4171253	3,3552178	5,386405	3,687108281	5,5336791	3,9518254	4,0718889	4,3955558	5,409267756	4,676809478	2,44933460
236	-99	-99	-99	-99	-99	-99	-99	-99	-99	-99	-99	-99	-99	-99	-99
237	-99	-99	-99	-99	-99	-99	-99	-99	-99	-99	-99	-99	-99	-99	-99
238	-99	-99	-99	-99	-99	-99	-99	-99	-99	-99	-99	-99	-99	-99	-99
239	-99	-99	-99	-99	-99	-99	-99	-99	-99	-99	-99	-99	-99	-99	-99
240	-99	-99	-99	-99	-99	-99	-99	-99	-99	-99	-99	-99	-99	-99	-99
241	-99	-99	-99	-99	-99	-99	-99	-99	-99	-99	-99	-99	-99	-99	-99
242	-99	-99	-99	-99	-99	-99	-99	-99	-99	-99	-99	-99	-99	-99	-99
243	3,40918038	3,7862339	4,4715447	4,2224791	5,6145207	3,0373642	5,3593001	3,620245693	5,5335589	4,2827453	4,1169553	4,1997645	5,368201909	4,448169005	2,81538009
244	3,159878769	3,7095152	4,2877362	4,4369636	5,7009105	3,4841929	5,4582673	3,70813	5,4164647	4,2000848	4,1260965	4,3282822	5,326119006	4,379171924	2,65724050
245	-99	-99	-99	-99	-99	-99	-99	-99	-99	-99	-99	-99	-99	-99	-99
246	3,335154314	4,3015975	5,1717535	4,3552152	5,7907008	3,7522357	5,3836161	4,039843042	5,3721276	4,0905847	3,8967998	4,3209899	5,218314541	4,645091272	-99
247	-99	-99	-99	-99	-99	-99	-99	-99	-99	-99	-99	-99	-99	-99	-99
248	3,516773803	3,9961658	5,3653288	4,3003755	5,51742	3,0583691	5,2033017	3,776162188	5,5748319	4,0538069	3,8730256	4,0504637	5,262328395	4,073808635	3,19279184
249	3,367451385	4,4236533	5,346395	4,4366045	5,3488971	3,716934	5,4156704	3,601521424	5,4879057	3,9914584	3,8612252	4,4753749	5,249997474	4,857496493	3,42281888
250	-99	-99	-99	-99	-99	-99	-99	-99	-99	-99	-99	-99	-99	-99	-99
251	3,216995683	3,8597271	5,3907411	4,4444261	5,7538294	3,4147804	5,3928269	4,017548901	5,4841912	4,0804463	4,0683836	3,9412935	5,220119431	4,453196732	-99
252	-99	-99	-99	-99	-99	-99	-99	-99	-99	-99	-99	-99	-99	-99	-99
253	-99	-99	-99	-99	-99	-99	-99	-99	-99	-99	-99	-99	-99	-99	-99
254	-99	-99	-99	-99	-99	-99	-99	-99	-99	-99	-99	-99	-99	-99	-99
255	-99	-99	-99	-99	-99	-99	-99	-99	-99	-99	-99	-99	-99	-99	-99
256	3,00831801	4,483617	5,2018248	4,425286	5,8541208	3,5359598	5,3250182	3,959163569	5,4026774	3,9899615	3,9589989	4,5038699	5,335577232	4,531731063	-99
257	-99	-99	-99	-99	-99	-99	-99	-99	-99	-99	-99	-99	-99	-99	-99
258	-99	-99	-99	-99	-99	-99	-99	-99	-99	-99	-99	-99	-99	-99	-99
259	3,298040253	3,8452692	5,3027571	4,1736023	5,6806123	3,2652171	5,2908804	3,76298961	5,5644106	4,312387	3,9656407	4,3128681	5,325987676	4,849248312	2,65644770
260	-99	-99	-99	-99	-99	-99	-99	-99	-99	-99	-99	-99	-99	-99	-99
261	2,806193366	4,1470981	5,3274096	4,2372177	5,6173693	3,621144	5,3772331	3,915785651	5,4735668	3,7896633	3,9270561	3,9892471	4,855534845	4,761963763	-99
262	3,164432715	4,1051959	4,8786615	4,3962849	5,1858927	3,786668	5,3839071	3,264074531	5,5558384	4,1640799	4,1374873	4,2634316	5,380303867	4,054352974	3,28166315
263	-99	-99	-99	-99	-99	-99	-99	-99	-99	-99	-99	-99	-99	-99	-99
264	3,276941992	3,7751623	4,4507488	4,1075917	5,6188645	3,6081295	5,4429806	3,480385948	5,4728012	4,1833564	4,0484989	4,2296911	5,325437064	4,743173153	-99
265	3,293615282	4,3901369	5,2241965	4,1296296	5,3281743	3,6571892	5,2934262	3,575843037	5,5447398	4,0219247	3,9747933	4,1589006	5,273872069	4,702115323	3,02787592
	16	17	18	19	20	21	22	23	24	25	26	27	28	29	30

**Location_ Treatment**	JKI_S_14_Ds_M1	JKI_S_14_Ds_M2	JKI_S_14_Ds_M3	JKI_S_14_Ds_M4	JKI_S_14_Ds_M5	JKI_S_14_Ds_M6	JKI_S_14_Ds_M7	JKI_S_14_Ds_M8	JKI_S_14_Ds_M9	JKI_S_14_Ds_M10	JKI_S_14_Ds_M11	JKI_S_14_Ds_M12	JKI_S_14_Ds_M13	JKI_S_14_Ds_M14	JKI_S_14_Ds_M15
**Metabolites F1 individuals**	Salicylic acid-glucopyranoside	A148006-10:	Threonic acic	Ribitol	Malic acid	A250002-10:	Galactinol	Fumaric acid	Quinic acid, 3-caffeoyl-, trans-	A237001-10:	A217004-10:	A174001-10:	A197007-101	Raffinose	Galactaric acid
1	3,346830662	3,84558	5,1522584	4,2460192	5,5004873	3,7719005	5,1962939	4,269602409	5,5150836	4,3939547	3,9448515	4,6089487	5,210010274	4,35408990	3,89312520
2	-99	-99	-99	-99	-99	-99	-99	-99	-99	-99	-99	-99	-99	-99	-99
3	-99	-99	-99	-99	-99	-99	-99	-99	-99	-99	-99	-99	-99	-99	-99
4	-99	-99	-99	-99	-99	-99	-99	-99	-99	-99	-99	-99	-99	-99	-99
5	-99	-99	-99	-99	-99	-99	-99	-99	-99	-99	-99	-99	-99	-99	-99
6	3,401833829	3,7617894	4,6225949	4,4735605	5,7460028	3,7160248	5,2983673	4,209716332	5,3844212	4,2537087	3,996901	4,845378	5,372834604	4,87451995	3,34941948
7	-99	-99	-99	-99	-99	-99	-99	-99	-99	-99	-99	-99	-99	-99	-99
8	-99	-99	-99	-99	-99	-99	-99	-99	-99	-99	-99	-99	-99	-99	-99
9	-99	-99	-99	-99	-99	-99	-99	-99	-99	-99	-99	-99	-99	-99	-99
10	-99	-99	-99	-99	-99	-99	-99	-99	-99	-99	-99	-99	-99	-99	-99
11	-99	-99	-99	-99	-99	-99	-99	-99	-99	-99	-99	-99	-99	-99	-99
12	-99	-99	-99	-99	-99	-99	-99	-99	-99	-99	-99	-99	-99	-99	-99
13	-99	-99	-99	-99	-99	-99	-99	-99	-99	-99	-99	-99	-99	-99	-99
14	-99	-99	-99	-99	-99	-99	-99	-99	-99	-99	-99	-99	-99	-99	-99
15	-99	-99	-99	-99	-99	-99	-99	-99	-99	-99	-99	-99	-99	-99	-99
16	3,097768237	3,7428811	5,166864	4,130891	5,6405005	2,910341	5,1812931	3,900732912	5,5349831	4,2975376	3,9669969	4,6747221	5,225278884	4,66318160	3,05936620
17	-99	-99	-99	-99	-99	-99	-99	-99	-99	-99	-99	-99	-99	-99	-99
18	3,338069241	4,4355802	5,0099893	3,982022	5,4827647	3,892847	5,0681471	4,447491363	5,4860109	4,2661858	4,0535794	4,5082059	5,140930806	4,44528339	4,01115469
19	3,075028144	3,9140371	5,1613564	4,1244709	5,6039985	3,7638705	5,1023624	4,254584166	5,334469	4,3021172	4,1983874	4,5466581	5,24247203	4,46270945	3,25505077
20	-99	-99	-99	-99	-99	-99	-99	-99	-99	-99	-99	-99	-99	-99	-99
21	-99	-99	-99	-99	-99	-99	-99	-99	-99	-99	-99	-99	-99	-99	-99
22	-99	-99	-99	-99	-99	-99	-99	-99	-99	-99	-99	-99	-99	-99	-99
23	-99	-99	-99	-99	-99	-99	-99	-99	-99	-99	-99	-99	-99	-99	-99
24	3,215178633	3,9149875	5,104694	4,1221537	5,6308311	3,5547298	5,0436206	4,468386153	5,3566362	3,9610084	4,0583669	4,4849924	5,144212045	4,14945878	3,27628750
25	-99	-99	-99	-99	-99	-99	-99	-99	-99	-99	-99	-99	-99	-99	-99
26	-99	-99	-99	-99	-99	-99	-99	-99	-99	-99	-99	-99	-99	-99	-99
27	-99	-99	-99	-99	-99	-99	-99	-99	-99	-99	-99	-99	-99	-99	-99
28	-99	-99	-99	-99	-99	-99	-99	-99	-99	-99	-99	-99	-99	-99	-99
29	-99	-99	-99	-99	-99	-99	-99	-99	-99	-99	-99	-99	-99	-99	-99
30	-99	-99	-99	-99	-99	-99	-99	-99	-99	-99	-99	-99	-99	-99	-99
31	-99	-99	-99	-99	-99	-99	-99	-99	-99	-99	-99	-99	-99	-99	-99
32	-99	-99	-99	-99	-99	-99	-99	-99	-99	-99	-99	-99	-99	-99	-99
33	2,830476355	3,7211561	5,1138902	3,9893845	5,7220088	3,720751	5,1277576	4,566069738	5,359823	4,1231299	4,0904106	4,845823	5,288984362	4,04382415	3,56013153
34	-99	-99	-99	-99	-99	-99	-99	-99	-99	-99	-99	-99	-99	-99,00000000	-99,00000000
35	3,160837593	4,2058948	5,0586167	4,3710634	5,6446411	2,9403113	5,143257	4,281410573	5,4701752	4,2809533	4,0492355	4,7641458	5,32337201	4,28300388	3,23381311
36	-99	-99	-99	-99	-99	-99	-99	-99	-99	-99	-99	-99	-99	-99	-99
37	-99	-99	-99	-99	-99	-99	-99	-99	-99	-99	-99	-99	-99	-99	-99
38	-99	-99	-99	-99	-99	-99	-99	-99	-99	-99	-99	-99	-99	-99	-99
39	-99	-99	-99	-99	-99	-99	-99	-99	-99	-99	-99	-99	-99	-99	-99
40	3,159220059	4,250798	4,9788586	4,175044	5,7154395	3,5768014	5,0739988	4,449785251	5,362942	4,3995802	3,92966	4,6039487	5,209216305	4,66950087	3,17144127
41	-99	-99	-99	-99	-99	-99	-99	-99	-99	-99	-99	-99	-99	-99	-99
42	-99	-99	-99	-99	-99	-99	-99	-99	-99	-99	-99	-99	-99	-99	-99
43	3,406236204	3,8878812	5,0987757	4,2624395	5,4071925	3,6406342	5,0490849	3,988245802	5,4775844	4,1019696	4,0694157	4,5804484	5,157131409	4,07016348	3,50918598
44	-99	-99	-99	-99	-99	-99	-99	-99	-99	-99	-99	-99	-99	-99	-99
45	-99	-99	-99	-99	-99	-99	-99	-99	-99	-99	-99	-99	-99	-99	-99
46	3,138958428	4,0774276	5,1550561	4,2737968	5,5600782	3,1460861	5,1805244	4,257934448	5,4924038	4,1846528	3,9268813	4,531241	5,183247584	4,68238535	2,57600468
47	-99	-99	-99	-99	-99	-99	-99	-99	-99	-99	-99	-99	-99	-99	-99
48	-99	-99	-99	-99	-99	-99	-99	-99	-99	-99	-99	-99	-99	-99	-99
49	3,327361874	3,8932323	5,1742149	4,0377783	5,6015143	3,6641181	5,0846678	4,600196918	5,3978998	4,272403	4,0664875	4,6693494	5,236090239	4,32470136	3,94316818
50	3,333376579	3,9231185	4,7672469	4,1752089	5,6170832	3,0599948	5,1365407	4,589373886	5,392855	4,3665219	4,0247631	4,6294294	5,137367532	4,43743459	2,81062803
51	3,209455047	4,0010835	5,1777085	4,0648057	5,670681	3,5359449	5,0020509	4,087843163	5,3201817	3,8501707	4,1403228	4,7282422	5,190717372	4,37640178	2,21002897
52	-99	-99	-99	-99	-99	-99	-99	-99	-99	-99	-99	-99	-99	-99	-99

53	-99	-99	-99	-99	-99	-99	-99	-99	-99	-99	-99	-99	-99	-99	-99
54	-99	-99	-99	-99	-99	-99	-99	-99	-99	-99	-99	-99	-99	-99	-99
55	-99	-99	-99	-99	-99	-99	-99	-99	-99	-99	-99	-99	-99	-99	-99
56	-99	-99	-99	-99	-99	-99	-99	-99	-99	-99	-99	-99	-99	-99	-99
57	3,606235368	3,7546286	4,3903481	4,3005834	5,6363023	3,8115241	5,2045552	4,371504783	5,531731	4,2075516	4,030242	4,6602039	5,275136386	4,25386464	3,89556004
58	3,464102759	3,7318389	4,813002	3,9950833	5,4738683	3,5875378	5,1219649	3,881508922	5,5291306	4,1073682	3,9489516	4,4681151	5,189557144	4,53393845	2,84763921
59	-99	-99	-99	-99	-99	-99	-99	-99	-99	-99	-99	-99	-99	-99	-99
60	-99	-99	-99	-99	-99	-99	-99	-99	-99	-99	-99	-99	-99	-99	-99
61	3,17562611	4,651811	5,211769	4,456672	5,6122942	3,7046536	5,2091775	4,284823412	5,5331655	4,3242308	3,9665089	4,6380672	5,325369566	4,17509243	3,48200720
62	-99	-99	-99	-99	-99	-99	-99	-99	-99	-99	-99	-99	-99	-99	-99
63	-99	-99	-99	-99	-99	-99	-99	-99	-99	-99	-99	-99	-99	-99	-99
64	-99	-99	-99	-99	-99	-99	-99	-99	-99	-99	-99	-99	-99	-99	-99
65	3,230873884	3,8505962	4,6354442	4,3555951	5,5832523	3,819578	5,2495541	4,347184788	5,3949312	4,2537087	4,0202985	4,599933	5,27803414	4,74806964	2,88427655
66	-99	-99	-99	-99	-99	-99	-99	-99	-99	-99	-99	-99	-99	-99	-99
67	-99	-99	-99	-99	-99	-99	-99	-99	-99	-99	-99	-99	-99	-99	-99
68	-99	-99	-99	-99	-99	-99	-99	-99	-99	-99	-99	-99	-99	-99	-99
69	-99	-99	-99	-99	-99	-99	-99	-99	-99	-99	-99	-99	-99	-99	-99
70	-99	-99	-99	-99	-99	-99	-99	-99	-99	-99	-99	-99	-99	-99	-99
71	-99	-99	-99	-99	-99	-99	-99	-99	-99	-99	-99	-99	-99	-99	-99
72	-99	-99	-99	-99	-99	-99	-99	-99	-99	-99	-99	-99	-99	-99	-99
73	-99	-99	-99	-99	-99	-99	-99	-99	-99	-99	-99	-99	-99	-99	-99
74	-99	-99	-99	-99	-99	-99	-99	-99	-99	-99	-99	-99	-99	-99	-99
75	3,276648584	3,8803363	4,5173896	4,248242	5,539176	3,7676742	5,1657277	4,305335898	5,5200086	4,1635202	3,9945916	4,58732	5,243301504	4,32182681	2,99944984
76	-99	-99	-99	-99	-99	-99	-99	-99	-99	-99	-99	-99	-99	-99	-99
77	-99	-99	-99	-99	-99	-99	-99	-99	-99	-99	-99	-99	-99	-99	-99
78	-99	-99	-99	-99	-99	-99	-99	-99	-99	-99	-99	-99	-99	-99	-99
79	-99	-99	-99	-99	-99	-99	-99	-99	-99	-99	-99	-99	-99	-99	-99
80	-99	-99	-99	-99	-99	-99	-99	-99	-99	-99	-99	-99	-99	-99	-99
81	-99	-99	-99	-99	-99	-99	-99	-99	-99	-99	-99	-99	-99	-99	-99
82	-99	-99	-99	-99	-99	-99	-99	-99	-99	-99	-99	-99	-99	-99	-99
83	-99	-99	-99	-99	-99	-99	-99	-99	-99	-99	-99	-99	-99	-99	-99
84	3,251643405	4,1175278	4,9123703	4,1994347	5,549607	3,9900644	5,172645	4,240530653	5,5306644	4,3282267	3,9743553	4,6568306	5,226763617	4,39321962	3,43316190
85	-99	-99	-99	-99	-99	-99	-99	-99	-99	-99	-99	-99	-99	-99	-99
86	-99	-99	-99	-99	-99	-99	-99	-99	-99	-99	-99	-99	-99	-99	-99
87	3,09905097	3,8865156	4,5886548	3,9918924	5,5639782	3,6070101	5,1118223	4,490571809	5,4593126	4,2057582	4,0293218	4,7309427	5,229980735	4,19446944	3,00480842
88	3,267069338	3,9042599	4,5952223	4,3016896	5,7151213	3,9871896	5,2057785	4,410185745	5,5181291	4,3893094	4,0311347	4,8041342	5,316306416	4,33352591	3,69128953
89	3,357519685	4,4685389	5,0702384	4,2177542	5,5577904	3,9326642	5,1189695	4,315673614	5,4413212	4,4687238	3,9567682	4,4995185	5,249047877	4,26010993	3,05566192
90	-99	-99	-99	-99	-99	-99	-99	-99	-99	-99	-99	-99	-99	-99	-99
91	-99	-99	-99	-99	-99	-99	-99	-99	-99	-99	-99	-99	-99	-99	-99
92	-99	-99	-99	-99	-99	-99	-99	-99	-99	-99	-99	-99	-99	-99	-99
93	-99	-99	-99	-99	-99	-99	-99	-99	-99	-99	-99	-99	-99	-99	-99
94	-99	-99	-99	-99	-99	-99	-99	-99	-99	-99	-99	-99	-99	-99	-99
95	-99	-99	-99	-99	-99	-99	-99	-99	-99	-99	-99	-99	-99	-99	-99
96	-99	-99	-99	-99	-99	-99	-99	-99	-99	-99	-99	-99	-99	-99	-99
97	-99	-99	-99	-99	-99	-99	-99	-99	-99	-99	-99	-99	-99	-99	-99
98	-99	-99	-99	-99	-99	-99	-99	-99	-99	-99	-99	-99	-99	-99	-99
99	3,178246045	3,7727236	4,644462	4,1265242	5,6758907	3,0467646	5,1049847	4,117378055	5,4740217	4,244367	4,0744172	4,5912463	5,181999753	4,34269420	3,22983224
100	3,371419974	4,4103018	5,1213056	3,9033033	5,6484208	3,6937368	5,1251175	4,105653644	5,3301984	4,0265697	3,9919712	4,6202466	5,23001447	4,59179407	2,42373302
101	-99	-99	-99	-99	-99	-99	-99	-99	-99	-99	-99	-99	-99	-99	-99
102	-99	-99	-99	-99	-99	-99	-99	-99	-99	-99	-99	-99	-99	-99	-99
103	3,413385223	3,8421192	5,0854726	4,1226402	5,5250352	3,3808736	5,1017731	4,172698078	5,5239788	4,2871823	3,9422178	4,493217	5,195120992	4,67819715	3,33609992
104	3,071025477	4,4031975	5,0739907	4,0733092	5,5956364	3,6636354	5,1290324	4,182472767	5,290077	3,9955216	4,0053958	4,6272696	5,178562968	4,78283557	2,14487436
105	-99	-99	-99	-99	-99	-99	-99	-99	-99	-99	-99	-99	-99	-99	-99
106	3,245400628	3,9503608	4,6589377	4,30593	5,5406539	3,6556844	5,17218	4,052059917	5,5223222	4,2188255	3,9342116	4,6021264	5,190658544	3,97617806	4,06905908
107	3,318087886	4,1097742	4,822689	4,0233506	5,4706228	3,8944395	5,1122788	4,151145182	5,4779392	4,3171418	4,0299819	4,4145826	5,17229012	4,46850827	2,97292473
108	-99	-99	-99	-99	-99	-99	-99	-99	-99	-99	-99	-99	-99	-99	-99
109	-99	-99	-99	-99	-99	-99	-99	-99	-99	-99	-99	-99	-99	-99	-99
110	3,592973219	3,8850573	4,5408491	4,2934562	5,6932406	3,8938236	5,0913111	4,362231678	5,5313521	4,509818	3,9531314	4,4901478	5,278475537	4,60749787	3,15900250
111	-99	-99	-99	-99	-99	-99	-99	-99	-99	-99	-99	-99	-99	-99	-99
112	-99	-99	-99	-99	-99	-99	-99	-99	-99	-99	-99	-99	-99	-99	-99
113	-99	-99	-99	-99	-99	-99	-99	-99	-99	-99	-99	-99	-99	-99	-99
114	3,553651084	4,04762	4,8803788	4,2893818	5,6325066	3,7240864	5,2264238	4,255067609	5,4665061	4,2538145	3,9437175	4,6067901	5,264654147	4,83823323	-99
115	-99	-99	-99	-99	-99	-99	-99	-99	-99	-99	-99	-99	-99	-99	-99
116	-99	-99	-99	-99	-99	-99	-99	-99	-99	-99	-99	-99	-99	-99	-99
117	-99	-99	-99	-99	-99	-99	-99	-99	-99	-99	-99	-99	-99	-99	-99
118	-99	-99	-99	-99	-99	-99	-99	-99	-99	-99	-99	-99	-99	-99	-99
119	-99	-99	-99	-99	-99	-99	-99	-99	-99	-99	-99	-99	-99	-99	-99
120	2,970249703	4,6146651	5,1779564	4,3019181	5,6547227	3,1503792	5,1449508	4,423332749	5,4194926	4,1687266	3,965942	4,4410151	5,218483604	4,89457442	-99
121	3,398164894	4,3077192	4,95861	3,9518373	5,385946	3,8731284	5,1917054	4,102222159	5,505428	4,3196917	3,9620148	4,6576192	5,183584794	4,82062721	2,60130461
122	3,415083448	4,4896933	5,1754997	4,341053	5,6884263	3,8055863	5,2159984	4,368557444	5,4803339	4,3349216	3,9022603	4,6007747	5,298454969	4,89753038	3,63830452
123	-99	-99	-99	-99	-99	-99	-99	-99	-99	-99	-99	-99	-99	-99	-99
124	-99	-99	-99	-99	-99	-99	-99	-99	-99	-99	-99	-99	-99	-99	-99
125	-99	-99	-99	-99	-99	-99	-99	-99	-99	-99	-99	-99	-99	-99	-99
126	-99	-99	-99	-99	-99	-99	-99	-99	-99	-99	-99	-99	-99	-99	-99
127	3,660893691	3,941547	4,6227024	4,2510342	5,5396701	4,2085499	5,1265928	4,349098516	5,5265436	4,4130297	4,0133826	4,7539632	5,259837573	4,39634885	3,89517468
128	-99	-99	-99	-99	-99	-99	-99	-99	-99	-99	-99	-99	-99	-99	-99
129	-99	-99	-99	-99	-99	-99	-99	-99	-99	-99	-99	-99	-99	-99	-99
130	-99	-99	-99	-99	-99	-99	-99	-99	-99	-99	-99	-99	-99	-99	-99
131	-99	-99	-99	-99	-99	-99	-99	-99	-99	-99	-99	-99	-99	-99	-99
132	-99	-99	-99	-99	-99	-99	-99	-99	-99	-99	-99	-99	-99	-99	-99
133	-99	-99	-99	-99	-99	-99	-99	-99	-99	-99	-99	-99	-99	-99	-99
134	-99	-99	-99	-99	-99	-99	-99	-99	-99	-99	-99	-99	-99	-99	-99
135	-99	-99	-99	-99	-99	-99	-99	-99	-99	-99	-99	-99	-99	-99	-99
136	-99	-99	-99	-99	-99	-99	-99	-99	-99	-99	-99	-99	-99	-99	-99
137	-99	-99	-99	-99	-99	-99	-99	-99	-99	-99	-99	-99	-99	-99	-99
138	3,199378747	4,4765658	5,1330836	4,3266112	5,6140412	3,4226421	5,2214698	4,105880051	5,4631411	4,1725529	4,0154375	4,6662177	5,177674058	4,83577393	3,43035378
139	-99	-99	-99	-99	-99	-99	-99	-99	-99	-99	-99	-99	-99	-99	-99
140	3,22867321	4,5696057	5,1034679	4,3058895	5,62076	3,4778331	5,1126706	4,283136219	5,3547801	4,0157334	4,0444648	4,6010576	5,245177912	4,14423262	2,66324398
141	3,349045874	4,0662091	4,9083025	4,2075023	5,6619087	4,0439882	5,1454858	4,340803555	5,4741998	4,2345195	4,0207236	4,6650857	5,292104898	4,75774023	2,66346625
142	3,229835031	3,8115942	4,4900703	4,1117205	5,7499437	3,8948665	5,1796784	4,517024448	5,4416155	4,4027058	4,0422761	4,7191384	5,276229146	4,14881138	3,40275413
143	3,351515443	3,9034081	4,9117243	4,1786712	5,5061991	4,0918097	5,2361082	3,977796139	5,5635989	4,2966785	3,9506228	4,5124198	5,178487424	4,49325208	3,23032283
144	-99	-99	-99	-99	-99	-99	-99	-99	-99	-99	-99	-99	-99	-99	-99
145	3,166600626	3,7522708	4,5351287	4,3351861	5,6665476	3,1519006	5,1223385	4,424930808	5,4891697	4,3581257	4,0562542	4,7746664	5,30515821	4,29825751	3,51499456
146	-99	-99	-99	-99	-99	-99	-99	-99	-99	-99	-99	-99	-99	-99	-99
147	3,514087765	4,5996732	5,1585959	4,3196803	5,6244425	4,2328493	5,2612938	3,985506203	5,5339547	4,4016672	3,9518592	4,7398787	5,336282291	4,50928491	3,39118386
148	-99	-99	-99	-99	-99	-99	-99	-99	-99	-99	-99	-99	-99	-99	-99
149	-99	-99	-99	-99	-99	-99	-99	-99	-99	-99	-99	-99	-99	-99	-99
150	-99	-99	-99	-99	-99	-99	-99	-99	-99	-99	-99	-99	-99	-99	-99
151	-99	-99	-99	-99	-99	-99	-99	-99	-99	-99	-99	-99	-99	-99	-99
152	3,15674878	3,8956939	4,5654625	4,2249885	5,6525575	3,8697538	5,2212596	4,646177532	5,4592924	4,1634268	3,8817862	4,4839449	5,233612104	4,62289220	3,36052378
153	-99	-99	-99	-99	-99	-99	-99	-99	-99	-99	-99	-99	-99	-99	-99
154	-99	-99	-99	-99	-99	-99	-99	-99	-99	-99	-99	-99	-99	-99	-99
155	-99	-99	-99	-99	-99	-99	-99	-99	-99	-99	-99	-99	-99	-99	-99
156	-99	-99	-99	-99	-99	-99	-99	-99	-99	-99	-99	-99	-99	-99	-99
157	-99	-99	-99	-99	-99	-99	-99	-99	-99	-99	-99	-99	-99	-99	-99
158	-99	-99	-99	-99	-99	-99	-99	-99	-99	-99	-99	-99	-99	-99	-99
159	3,085918461	4,5073583	5,1009706	4,0169732	5,5243175	3,8702718	5,1193307	4,397944803	5,4500829	4,1289056	3,8678684	4,3274749	5,155057835	4,17144946	3,30641918
160	3,244143154	3,7354323	4,4019298	4,0751034	5,6189452	3,8649193	5,0757747	4,324512047	5,5136826	4,2529483	3,9452264	4,5650551	5,242223777	4,61679746	3,45784627
161	3,082444939	3,9892351	4,7126258	4,1081888	5,553286	3,791462	5,142074	4,387958663	5,4943914	4,1575361	3,874371	4,3995089	5,165018885	4,51276045	3,31719621
162	-99	-99	-99	-99	-99	-99	-99	-99	-99	-99	-99	-99	-99	-99	-99
163	-99	-99	-99	-99	-99	-99	-99	-99	-99	-99	-99	-99	-99	-99	-99
164	3,150791167	3,7613864	4,7956382	4,4579748	5,7197501	3,6761698	5,326042	4,072412278	5,3813353	4,3611185	4,1261601	4,9726211	5,352901698	4,81093423	3,31921070

165	-99	-99	-99	-99	-99	-99	-99	-99	-99	-99	-99	-99	-99	-99	-99
166	-99	-99	-99	-99	-99	-99	-99	-99	-99	-99	-99	-99	-99	-99	-99
167	3,538144258	3,7104819	5,2061395	4,2737232	5,5114798	4,0537439	5,2084874	3,797197584	5,5387268	4,3136295	3,8900527	4,5096664	5,232415871	4,26716594	2,80122350
168	-99	-99	-99	-99	-99	-99	-99	-99	-99	-99	-99	-99	-99	-99	-99
169	-99	-99	-99	-99	-99	-99	-99	-99	-99	-99	-99	-99	-99	-99	-99
170	-99	-99	-99	-99	-99	-99	-99	-99	-99	-99	-99	-99	-99	-99	-99
171	3,305552986	3,825135	5,2180303	4,0026475	5,7229433	3,8441508	5,0853162	4,438045614	5,4126273	4,5615051	4,1733504	4,819937	5,30089859	4,29180393	3,14137821
172	3,097081961	3,7918888	4,8967694	4,0112738	5,5593128	3,3213751	5,1556399	4,561960229	5,4283155	4,1475471	3,92192	4,5907936	5,211071549	4,28581487	3,41180155
173	-99	-99	-99	-99	-99	-99	-99	-99	-99	-99	-99	-99	-99	-99	-99
174	3,371219171	4,0192386	4,7155405	4,1899583	5,6893433	3,9277521	5,1801363	4,062365413	5,5316229	4,426348	4,0545998	4,9309957	5,384383042	4,78671754	3,35749694
175	3,15374218	4,0737023	4,8950894	4,2791362	5,5770156	3,4543535	5,1285495	3,965307328	5,5276552	4,2810483	4,0548742	4,4651202	5,055870218	4,54384112	3,03833874
176	2,881774718	4,04776	5,0978123	3,8046798	5,5129076	3,4592799	5,0630691	4,504314443	5,4957898	4,2634335	3,9767432	4,5979626	5,150364837	4,34624965	3,09070963
177	3,485004222	3,8155651	4,5547176	4,0170653	5,2045617	3,878796	5,11328	4,141272	5,5358685	4,3842763	4,015613	4,6560653	5,250943023	3,80528637	3,40637188
178	-99	-99	-99	-99	-99	-99	-99	-99	-99	-99	-99	-99	-99	-99	-99
179	-99	-99	-99	-99	-99	-99	-99	-99	-99	-99	-99	-99	-99	-99	-99
180	-99	-99	-99	-99	-99	-99	-99	-99	-99	-99	-99	-99	-99	-99	-99
181	-99	-99	-99	-99	-99	-99	-99	-99	-99	-99	-99	-99	-99	-99	-99
182	3,335785488	4,7284263	5,1696347	4,2185064	5,6197632	3,3065157	5,2676706	4,00746089	5,580107	4,3428832	3,9535183	4,7152902	5,253141056	4,69429402	3,81530656
183	3,560946191	3,6888281	4,472906	4,3355361	5,7735118	3,942021	5,2610191	4,250372782	5,4521809	4,264523	4,1050177	4,8213098	5,319872758	4,17087684	3,38705248
184	-99	-99	-99	-99	-99	-99	-99	-99	-99	-99	-99	-99	-99	-99	-99
185	-99	-99	-99	-99	-99	-99	-99	-99	-99	-99	-99	-99	-99	-99	-99
186	-99	-99	-99	-99	-99	-99	-99	-99	-99	-99	-99	-99	-99	-99	-99
187	-99	-99	-99	-99	-99	-99	-99	-99	-99	-99	-99	-99	-99	-99	-99
188	-99	-99	-99	-99	-99	-99	-99	-99	-99	-99	-99	-99	-99	-99	-99
189	3,113597628	3,9916419	4,8486558	3,9631961	5,4534784	3,0920258	5,1892207	3,880416208	5,5113685	4,0148588	4,0935416	4,7319004	5,169475981	4,25257815	3,36958812
190	-99	-99	-99	-99	-99	-99	-99	-99	-99	-99	-99	-99	-99	-99	-99
191	-99	-99	-99	-99	-99	-99	-99	-99	-99	-99	-99	-99	-99	-99	-99
192	-99	-99	-99	-99	-99	-99	-99	-99	-99	-99	-99	-99	-99	-99	-99
193	-99	-99	-99	-99	-99	-99	-99	-99	-99	-99	-99	-99	-99	-99	-99
194	-99	-99	-99	-99	-99	-99	-99	-99	-99	-99	-99	-99	-99	-99	-99
195	-99	-99	-99	-99	-99	-99	-99	-99	-99	-99	-99	-99	-99	-99	-99
196	-99	-99	-99	-99	-99	-99	-99	-99	-99	-99	-99	-99	-99	-99	-99
197	-99	-99	-99	-99	-99	-99	-99	-99	-99	-99	-99	-99	-99	-99	-99
198	3,404944136	4,0012854	4,5486864	4,2577476	5,4734364	3,9144394	5,1457724	4,115448688	5,5572315	4,3920247	3,8203316	4,541095	5,190473525	4,85935008	2,77694694
199	3,452450996	3,9421207	4,7155166	4,1146306	5,6751316	3,6402839	5,2388493	4,166145422	5,566296	4,3295692	3,8096254	4,6518484	5,184154861	4,09081038	3,05040840
200	-99	-99	-99	-99	-99	-99	-99	-99	-99	-99	-99	-99	-99	-99	-99
201	-99	-99	-99	-99	-99	-99	-99	-99	-99	-99	-99	-99	-99	-99	-99
202	3,087381669	3,7331419	5,1264205	4,2169843	5,5078485	3,0714839	5,082081	4,151169192	5,4364981	4,1425989	4,0664849	4,5515735	5,136612303	4,51890694	2,98273475
203	3,28470582	3,7894495	4,4462071	4,0417918	5,5963743	3,8488404	5,0421839	4,289961146	5,5203821	4,0368455	3,945226	4,4036204	5,236334947	4,60198378	3,10055030
204	-99	-99	-99	-99	-99	-99	-99	-99	-99	-99	-99	-99	-99	-99	-99
205	-99	-99	-99	-99	-99	-99	-99	-99	-99	-99	-99	-99	-99	-99	-99
206	-99	-99	-99	-99	-99	-99	-99	-99	-99	-99	-99	-99	-99	-99	-99
207	-99	-99	-99	-99	-99	-99	-99	-99	-99	-99	-99	-99	-99	-99	-99
208	-99	-99	-99	-99	-99	-99	-99	-99	-99	-99	-99	-99	-99	-99	-99
209	3,389171996	3,6781118	5,1289581	4,3314395	5,6325511	4,0521344	5,0647701	4,208994952	5,456363	4,1158539	4,1385279	4,7069999	5,24113803	4,30477300	3,45087423
210	-99	-99	-99	-99	-99	-99	-99	-99	-99	-99	-99	-99	-99	-99	-99
211	-99	-99	-99	-99	-99	-99	-99	-99	-99	-99	-99	-99	-99	-99	-99
212	3,316625878	3,8120794	4,8339921	4,0188908	5,7432876	3,6465602	5,1441605	4,549085599	5,5146735	4,2534657	4,0282306	4,7293644	5,209694804	4,17370243	3,99542357
213	-99	-99	-99	-99	-99	-99	-99	-99	-99	-99	-99	-99	-99	-99	-99
214	3,54513863	3,8168612	5,1414975	4,0539255	5,4998815	4,1063711	4,9971213	4,194629024	5,4564216	4,1094934	4,0597514	4,5534415	5,155245057	3,88425911	3,37048586
215	-99	-99	-99	-99	-99	-99	-99	-99	-99	-99	-99	-99	-99	-99	-99
216	3,090017902	3,9058416	4,6553842	4,1227278	5,6520065	3,2123392	5,1059705	4,363767889	5,4957938	4,200498	4,0800848	4,7434326	5,252961438	4,48585054	3,31726405
217	-99	-99	-99	-99	-99	-99	-99	-99	-99	-99	-99	-99	-99	-99	-99
218	-99	-99	-99	-99	-99	-99	-99	-99	-99	-99	-99	-99	-99	-99	-99
219	-99	-99	-99	-99	-99	-99	-99	-99	-99	-99	-99	-99	-99	-99	-99
220	3,368661157	3,8979074	4,8143422	3,9445009	5,5812056	3,9923288	5,0808614	3,999725644	5,4957444	4,3451259	4,0127362	4,6012441	5,270299549	4,03749719	3,06045143
221	3,002824172	3,6613464	5,2184364	3,9120452	5,7281039	3,2167175	5,2009574	4,410489252	5,3762476	4,1333173	4,185854	4,9258328	5,334541072	3,61005660	3,53080827
222	3,182289015	3,8131673	4,4623362	4,2042558	5,5961312	3,6173297	5,1469654	4,585824201	5,522208	4,3840755	3,9589706	4,5007587	5,147256392	4,48206229	2,91172789
223	3,351487181	3,8862112	5,1669307	3,9160889	5,4883728	3,7270613	5,1173054	4,128524106	5,5241467	4,1936107	3,9995157	4,6026915	5,220580705	4,38534150	2,64892082
224	-99	-99	-99	-99	-99	-99	-99	-99	-99	-99	-99	-99	-99	-99	-99
225	3,569376636	4,0244597	5,1350474	4,2648559	5,5718234	3,9051146	5,1067316	4,024573971	5,5476482	4,089706	4,1327955	4,6830704	5,222647586	4,26616485	3,20205863
226	3,332665353	4,5138936	5,099308	4,6880955	5,6300874	3,7950664	5,2194406	4,137600792	5,4754799	4,1509766	4,0153608	4,8034685	5,293820072	4,36353552	2,79566505
227	-99	-99	-99	-99	-99	-99	-99	-99	-99	-99	-99	-99	-99	-99	-99
228	-99	-99	-99	-99	-99	-99	-99	-99	-99	-99	-99	-99	-99	-99	-99
229	-99	-99	-99	-99	-99	-99	-99	-99	-99	-99	-99	-99	-99	-99	-99
230	-99	-99	-99	-99	-99	-99	-99	-99	-99	-99	-99	-99	-99	-99	-99
231	-99	-99	-99	-99	-99	-99	-99	-99	-99	-99	-99	-99	-99	-99	-99
232	3,155254776	4,5704902	5,1836632	4,1923265	5,7136947	3,2234181	5,0902447	4,594803783	5,4133257	4,0585847	4,1168581	4,7887503	5,306615026	4,12167481	2,77843489
233	3,175344738	4,6295313	5,1393527	4,2739696	5,5054744	3,6152566	5,2495812	4,373728023	5,4678744	4,3945115	3,8850888	4,5482901	5,220220927	4,27110206	3,26703234
234	3,382059385	3,6483995	4,6785745	4,0281419	5,77866	3,6868019	5,1372512	4,757470341	5,2988326	4,2108849	4,0747984	4,6743655	5,23112323	4,21246943	3,30798235
235	3,271782025	4,4172225	5,0499018	4,2103853	5,4789927	3,2878949	5,1053947	4,316435286	5,4023357	3,9472286	4,0830846	4,6746807	5,177676006	4,52540897	3,34910573
236	-99	-99	-99	-99	-99	-99	-99	-99	-99	-99	-99	-99	-99	-99	-99
237	-99	-99	-99	-99	-99	-99	-99	-99	-99	-99	-99	-99	-99	-99	-99
238	-99	-99	-99	-99	-99	-99	-99	-99	-99	-99	-99	-99	-99	-99	-99
239	-99	-99	-99	-99	-99	-99	-99	-99	-99	-99	-99	-99	-99	-99	-99
240	-99	-99	-99	-99	-99	-99	-99	-99	-99	-99	-99	-99	-99	-99	-99
241	-99	-99	-99	-99	-99	-99	-99	-99	-99	-99	-99	-99	-99	-99	-99
242	-99	-99	-99	-99	-99	-99	-99	-99	-99	-99	-99	-99	-99	-99	-99
243	3,166545972	3,6931059	4,6936114	4,0467895	5,5583396	3,0430459	5,0814278	4,243003316	5,478088	4,2240908	4,1282279	4,5992311	5,14888652	4,17471908	3,41725255
244	3,172434419	4,0037685	4,8455908	4,2122979	5,5182529	3,6343952	5,0918411	4,383087004	5,3597019	4,4878888	4,1222201	4,6917705	5,215001696	3,91012184	3,31182006
245	-99	-99	-99	-99	-99	-99	-99	-99	-99	-99	-99	-99	-99	-99	-99
246	3,368915372	4,2966406	5,0289606	4,1845525	5,6569581	3,9998273	5,1342958	4,349582866	5,3990201	4,1900445	4,0437687	4,6560954	5,213447312	4,22290127	3,33046592
247	-99	-99	-99	-99	-99	-99	-99	-99	-99	-99	-99	-99	-99	-99	-99
248	3,348508591	4,2704631	4,9692807	4,1318039	5,5975232	3,3951727	5,1193836	4,209365707	5,4690664	3,9984134	4,0097371	4,7249324	5,263958237	3,90758194	3,25705559
249	3,344585658	3,8418989	4,6984801	3,902519	5,6694207	3,7568465	5,2080754	4,816477232	5,3820695	4,1164027	4,0764079	4,6873968	5,285049576	4,03105344	3,85881766
250	-99	-99	-99	-99	-99	-99	-99	-99	-99	-99	-99	-99	-99	-99	-99
251	3,149726608	4,2999636	5,2090675	4,3837382	5,7659955	3,9310208	5,1718263	4,279042414	5,4228858	4,4306307	4,178419	4,9400297	5,285885851	4,25439525	2,92569055
252	-99	-99	-99	-99	-99	-99	-99	-99	-99	-99	-99	-99	-99	-99	-99
253	-99	-99	-99	-99	-99	-99	-99	-99	-99	-99	-99	-99	-99	-99	-99
254	-99	-99	-99	-99	-99	-99	-99	-99	-99	-99	-99	-99	-99	-99	-99
255	-99	-99	-99	-99	-99	-99	-99	-99	-99	-99	-99	-99	-99	-99	-99
256	3,06936959	4,3708949	5,0586987	4,2733474	5,6151074	3,8268068	5,0867011	4,423176801	5,393186	4,0255123	4,098084	4,6607864	5,251679278	4,35470494	3,30473257
257	-99	-99	-99	-99	-99	-99	-99	-99	-99	-99	-99	-99	-99	-99	-99
258	-99	-99	-99	-99	-99	-99	-99	-99	-99	-99	-99	-99	-99	-99	-99
259	3,268983742	3,8575037	5,2048733	4,1367601	5,5854673	3,4480171	5,21377	4,21140602	5,5317924	4,4567182	3,9019626	4,6009885	5,229464475	4,75156389	2,93613548
260	-99	-99	-99	-99	-99	-99	-99	-99	-99	-99	-99	-99	-99	-99	-99
261	3,194202604	3,9119219	5,0154809	4,1512076	5,5696599	3,7996737	5,1947546	4,190463721	5,5235953	4,3385778	3,9356211	4,4189055	5,211859147	4,58085290	3,16028156
262	3,201504966	4,4329914	5,0242861	4,2924441	5,4651325	3,8368856	5,1006547	4,311920025	5,5014046	4,0716005	3,9780554	4,3139338	5,178627076	4,59095761	3,39690455
263	-99	-99	-99	-99	-99	-99	-99	-99	-99	-99	-99	-99	-99	-99	-99
264	3,390029144	3,8636469	4,5895234	3,9786663	5,6706062	3,6678403	5,1469962	4,298506168	5,4075628	4,3606906	4,1238783	4,7677687	5,287675503	4,82319985	3,26530271
265	3,393389427	3,9480468	4,7222979	4,3092286	5,5569055	3,9768988	5,1417168	4,481634308	5,4620398	4,1889231	4,1961446	4,5995443	5,096611449	4,33575544	3,44595250
	31	32	33	34	35	36	37	38	39	40	41	42	43	44	45

**Location_ Treatment**	MPI_FGH_14_ Co_M1	MPI_FGH_1 4_Co_M2	MPI_FGH_1 4_Co_M3	MPI_FGH_1 4 _Co_M4	MPI_FGH_1 4_Co_M5	MPI_FGH_1 4 _Co_M6	MPI_FGH_1 4_Co_M7	MPI_FGH_14_ Co_M8	MPI_FGH_1 4_Co_M9	MPI_FGH_1 4_Co_M10	MPI_FGH_1 4_Co_M11	MPI_FGH_1 4_Co_M12	MPI_FGH_14_ Co_M13	MPI_FGH_14_ Co _M14	MPI_FGH_14_ Co _M15
**Metabolites F1 individuals**	Salicylic acid-glucopyranoside	A148006-10:	Threonic acic	Ribitol	Malic acid	A250002-10:	Galactinol	Fumaric acid	Quinic acid, 3-caffeoyl-, trans-	A237001-10:	A217004-10:	A174001-10:	A197007-101	Raffinose	Galactaric acid
1	3,681466977	4,0990029	4,9925888	4,0375085	5,5146512	3,8983309	4,7660413	4,125790886	5,5046143	4,67243	3,8923553	4,3378341	5,191944686	-99	3,73744277
2	-99	-99	-99	-99	-99	-99	-99	-99	-99	-99	-99	-99	-99	-99	-99
3	-99	-99	-99	-99	-99	-99	-99	-99	-99	-99	-99	-99	-99	-99	-99
4	-99	-99	-99	-99	-99	-99	-99	-99	-99	-99	-99	-99	-99	-99	-99

5	-99	-99	-99	-99	-99	-99	-99	-99	-99	-99	-99	-99	-99	-99	-99
6	4,090255349	4,0102481	4,8059173	3,9930367	5,5555073	3,6653671	3,8336416	3,897683801	5,3620806	4,2074125	3,8707512	4,3921554	4,995988902	-99	3,16317023
7	-99	-99	-99	-99	-99	-99	-99	-99	-99	-99	-99	-99	-99	-99	-99
8	-99	-99	-99	-99	-99	-99	-99	-99	-99	-99	-99	-99	-99	-99	-99
9	-99	-99	-99	-99	-99	-99	-99	-99	-99	-99	-99	-99	-99	-99	-99
10	-99	-99	-99	-99	-99	-99	-99	-99	-99	-99	-99	-99	-99	-99	-99
11	-99	-99	-99	-99	-99	-99	-99	-99	-99	-99	-99	-99	-99	-99	-99
12	-99	-99	-99	-99	-99	-99	-99	-99	-99	-99	-99	-99	-99	-99	-99
13	-99	-99	-99	-99	-99	-99	-99	-99	-99	-99	-99	-99	-99	-99	-99
14	-99	-99	-99	-99	-99	-99	-99	-99	-99	-99	-99	-99	-99	-99	-99
15	-99	-99	-99	-99	-99	-99	-99	-99	-99	-99	-99	-99	-99	-99	-99
16	2,816131868	4,0638613	4,9991365	4,1262946	5,5389006	-99	4,9601013	3,997488577	5,5452756	4,2005604	3,8169596	4,4217416	5,161826432	-99	2,81885959
17	3,462440606	4,0043084	5,1161073	3,9632498	5,650965	3,9215871	4,0581658	4,259737334	5,5152399	4,1370935	3,9975027	4,2615234	5,178883372	-99	3,06251729
18	3,361684527	3,8879475	4,8495603	4,0634618	5,6147921	3,7637708	4,2457072	4,368050616	5,5330894	3,9594539	3,9800403	4,3031027	5,08811621	-99	3,66210300
19	3,135035681	4,0825729	5,0435996	4,16803	5,4805056	3,7277785	5,1182841	3,829394996	5,5259092	4,030321	4,0560509	4,3420498	5,277764625	-99	3,16048686
20	-99	-99	-99	-99	-99	-99	-99	-99	-99	-99	-99	-99	-99	-99	-99
21	-99	-99	-99	-99	-99	-99	-99	-99	-99	-99	-99	-99	-99	-99	-99
22	-99	-99	-99	-99	-99	-99	-99	-99	-99	-99	-99	-99	-99	-99	-99
23	-99	-99	-99	-99	-99	-99	-99	-99	-99	-99	-99	-99	-99	-99	-99
24	3,150307557	4,2433748	4,926496	4,0711739	5,5177176	3,2901843	5,0508785	4,023333022	5,5072528	3,869186	3,8304243	4,2635375	5,108505549	-99	3,04101266
25	-99	-99	-99	-99	-99	-99	-99	-99	-99	-99	-99	-99	-99	-99	-99
26	-99	-99	-99	-99	-99	-99	-99	-99	-99	-99	-99	-99	-99	-99	-99
27	-99	-99	-99	-99	-99	-99	-99	-99	-99	-99	-99	-99	-99	-99	-99
28	-99	-99	-99	-99	-99	-99	-99	-99	-99	-99	-99	-99	-99	-99	-99
29	-99	-99	-99	-99	-99	-99	-99	-99	-99	-99	-99	-99	-99	-99	-99
30	-99	-99	-99	-99	-99	-99	-99	-99	-99	-99	-99	-99	-99	-99	-99
31	-99	-99	-99	-99	-99	-99	-99	-99	-99	-99	-99	-99	-99	-99	-99
32	-99	-99	-99	-99	-99	-99	-99	-99	-99	-99	-99	-99	-99	-99	-99
33	2,765983858	4,0561385	4,8621788	4,0984505	5,5866441	3,8229619	5,0352608	3,976274347	5,516759	4,339608	4,0592278	4,3240392	5,102078768	-99	3,32365405
34	-99	-99	-99	-99	-99	-99	-99	-99	-99	-99	-99	-99	-99	-99	-99
35	3,150524859	3,914669	4,9068321	4,2381741	5,5634566	-99	4,6016607	3,907844918	5,5230204	4,056887	4,0503418	4,5305751	5,229809971	-99	3,20669516
36	-99	-99	-99	-99	-99	-99	-99	-99	-99	-99	-99	-99	-99	-99	-99
37	-99	-99	-99	-99	-99	-99	-99	-99	-99	-99	-99	-99	-99	-99	-99
38	-99	-99	-99	-99	-99	-99	-99	-99	-99	-99	-99	-99	-99	-99	-99
39	-99	-99	-99	-99	-99	-99	-99	-99	-99	-99	-99	-99	-99	-99	-99
40	3,393616124	4,010952	4,8401144	4,0799323	5,6392406	3,3348499	5,2712641	4,151810143	5,5311027	4,5508888	3,9653542	4,4966959	5,18680226	4,13704024	3,22904129
41	-99	-99	-99	-99	-99	-99	-99	-99	-99	-99	-99	-99	-99	-99	-99
42	-99	-99	-99	-99	-99	-99	-99	-99	-99	-99	-99	-99	-99	-99	-99
43	3,692797294	3,9861141	4,9012554	4,2502041	5,5989147	3,8508292	3,4175526	4,343393716	5,5385515	4,4425732	4,1247082	4,1327867	5,305675992	-99	3,43630197
44	-99	-99	-99	-99	-99	-99	-99	-99	-99	-99	-99	-99	-99	-99	-99
45	-99	-99	-99	-99	-99	-99	-99	-99	-99	-99	-99	-99	-99	-99	-99
46	3,308187498	4,3005345	5,1977449	4,3534552	5,4205175	3,1673235	5,5514992	3,73861265	5,5079553	4,088934	4,1393045	4,5609381	5,417361517	4,00176783	2,82911279
47	-99	-99	-99	-99	-99	-99	-99	-99	-99	-99	-99	-99	-99	-99	-99
48	-99	-99	-99	-99	-99	-99	-99	-99	-99	-99	-99	-99	-99	-99	-99
49	2,89964862	3,8933334	4,8976173	4,044287	5,6794649	3,6870054	4,6513608	4,515176259	5,2639505	3,2900502	4,0156661	4,3772689	5,12404104	-99	3,63907952
50	3,253289491	4,1148391	5,0745359	4,2511145	5,643929	-99	4,95691	4,313376489	5,5261978	4,2280872	3,9933652	4,3540104	5,02913312	-99	-99
51	2,771747359	4,0390342	4,8145748	4,0980208	5,7423984	-99	3,8545163	4,592366656	5,3414928	3,5455509	4,1260576	4,3754538	5,128962654	-99	-99
52	-99	-99	-99	-99	-99	-99	-99	-99	-99	-99	-99	-99	-99	-99	-99
53	-99	-99	-99	-99	-99	-99	-99	-99	-99	-99	-99	-99	-99	-99	-99
54	-99	-99	-99	-99	-99	-99	-99	-99	-99	-99	-99	-99	-99	-99	-99
55	-99	-99	-99	-99	-99	-99	-99	-99	-99	-99	-99	-99	-99	-99	-99
56	-99	-99	-99	-99	-99	-99	-99	-99	-99	-99	-99	-99	-99	-99	-99
57	3,332995051	3,9304005	4,5923801	4,1499209	5,4488706	3,4972423	5,3597256	3,835116284	5,5192308	4,0694195	3,9649532	4,38595	5,150117757	-99	3,49796568
58	3,070624307	4,2048798	5,1568279	3,921992	5,6060579	3,4516908	3,8248334	3,992962634	5,5222579	4,0469136	3,7981427	4,0246311	5,003505127	-99	2,37496585
59	-99	-99	-99	-99	-99	-99	-99	-99	-99	-99	-99	-99	-99	-99	-99
60	-99	-99	-99	-99	-99	-99	-99	-99	-99	-99	-99	-99	-99	-99	-99
61	3,290533621	3,9505629	4,6255825	4,2661104	5,5001464	3,3584366	4,8798724	3,823600351	5,507875	4,2839064	3,9778148	4,5112534	5,197969415	-99	3,35911838
62	-99	-99	-99	-99	-99	-99	-99	-99	-99	-99	-99	-99	-99	-99	-99
63	-99	-99	-99	-99	-99	-99	-99	-99	-99	-99	-99	-99	-99	-99	-99
64	-99	-99	-99	-99	-99	-99	-99	-99	-99	-99	-99	-99	-99	-99	-99
65	2,832377599	4,0422549	4,9702855	4,4123405	5,6259163	3,6358619	5,1896216	4,362470996	5,5383568	3,6846758	4,0074312	4,1786191	5,091677447	4,57639043	2,87274563
66	-99	-99	-99	-99	-99	-99	-99	-99	-99	-99	-99	-99	-99	-99	-99
67	-99	-99	-99	-99	-99	-99	-99	-99	-99	-99	-99	-99	-99	-99	-99
68	-99	-99	-99	-99	-99	-99	-99	-99	-99	-99	-99	-99	-99	-99	-99
69	-99	-99	-99	-99	-99	-99	-99	-99	-99	-99	-99	-99	-99	-99	-99
70	-99	-99	-99	-99	-99	-99	-99	-99	-99	-99	-99	-99	-99	-99	-99
71	-99	-99	-99	-99	-99	-99	-99	-99	-99	-99	-99	-99	-99	-99	-99
72	-99	-99	-99	-99	-99	-99	-99	-99	-99	-99	-99	-99	-99	-99	-99
73	-99	-99	-99	-99	-99	-99	-99	-99	-99	-99	-99	-99	-99	-99	-99
74	-99	-99	-99	-99	-99	-99	-99	-99	-99	-99	-99	-99	-99	-99	-99
75	3,358773775	3,9407245	4,7817471	4,2672246	5,6143424	3,4750059	4,9562512	4,219330676	5,5270572	4,2072141	4,0634251	4,5784309	5,364225565	-99	2,76518851
76	-99	-99	-99	-99	-99	-99	-99	-99	-99	-99	-99	-99	-99	-99	-99
77	-99	-99	-99	-99	-99	-99	-99	-99	-99	-99	-99	-99	-99	-99	-99
78	-99	-99	-99	-99	-99	-99	-99	-99	-99	-99	-99	-99	-99	-99	-99
79	-99	-99	-99	-99	-99	-99	-99	-99	-99	-99	-99	-99	-99	-99	-99
80	-99	-99	-99	-99	-99	-99	-99	-99	-99	-99	-99	-99	-99	-99	-99
81	-99	-99	-99	-99	-99	-99	-99	-99	-99	-99	-99	-99	-99	-99	-99
82	-99	-99	-99	-99	-99	-99	-99	-99	-99	-99	-99	-99	-99	-99	-99
83	-99	-99	-99	-99	-99	-99	-99	-99	-99	-99	-99	-99	-99	-99	-99
84	3,180777788	3,72506	4,8537731	4,0104618	5,6556848	3,3574146	4,9769766	4,122274167	5,533523	3,2324021	3,9843332	4,177392	5,086310531	-99	2,74089244
85	-99	-99	-99	-99	-99	-99	-99	-99	-99	-99	-99	-99	-99	-99	-99
86	-99	-99	-99	-99	-99	-99	-99	-99	-99	-99	-99	-99	-99	-99	-99
87	3,300716118	4,0239856	4,7884772	4,2287599	5,5509489	3,530129	5,124763	4,048586533	5,5202112	4,0046852	4,0828907	4,5747327	5,375222058	-99	3,05665525
88	3,487040681	4,0127241	4,7736574	4,1810957	5,5410102	4,1061584	4,8864732	4,070784859	5,5329847	4,3391258	4,0624976	4,5639423	5,340850716	-99	3,59087712
89	2,701299144	3,8354627	4,7792468	4,2528902	5,6260166	3,7590931	4,9853513	4,209422999	5,5189287	4,4793581	4,0843982	4,5434901	5,244885519	-99	3,15808824
90	-99	-99	-99	-99	-99	-99	-99	-99	-99	-99	-99	-99	-99	-99	-99
91	-99	-99	-99	-99	-99	-99	-99	-99	-99	-99	-99	-99	-99	-99	-99
92	-99	-99	-99	-99	-99	-99	-99	-99	-99	-99	-99	-99	-99	-99	-99
93	-99	-99	-99	-99	-99	-99	-99	-99	-99	-99	-99	-99	-99	-99	-99
94	-99	-99	-99	-99	-99	-99	-99	-99	-99	-99	-99	-99	-99	-99	-99
95	-99	-99	-99	-99	-99	-99	-99	-99	-99	-99	-99	-99	-99	-99	-99
96	-99	-99	-99	-99	-99	-99	-99	-99	-99	-99	-99	-99	-99	-99	-99
97	-99	-99	-99	-99	-99	-99	-99	-99	-99	-99	-99	-99	-99	-99	-99
98	-99	-99	-99	-99	-99	-99	-99	-99	-99	-99	-99	-99	-99	-99	-99
99	3,220209379	4,0856539	4,9516512	4,3385039	5,4467477	2,7936342	5,2030832	3,904099801	5,5218536	4,2305756	4,064313	4,4523049	5,365419496	3,93183228	3,18746915
100	3,206826397	3,7425404	4,5092556	4,0642833	5,6517925	3,5355273	5,1528778	3,889836108	5,4985721	-99	4,0363916	4,1144126	5,349049402	-99	2,84798899
101	-99	-99	-99	-99	-99	-99	-99	-99	-99	-99	-99	-99	-99	-99	-99
102	-99	-99	-99	-99	-99	-99	-99	-99	-99	-99	-99	-99	-99	-99	-99
103	3,249985502	4,158561	5,1781038	4,0408251	5,5280967	2,9260436	4,8800574	4,016443	5,5127249	3,9819984	3,8988542	4,2697269	5,304790313	-99	3,13933593
104	3,275994305	4,3092977	5,0788014	4,1053863	5,4932905	3,6619655	5,4598233	3,861365482	5,5190795	4,0440647	3,9078654	4,4212957	5,195920588	-99	2,92155414
105	-99	-99	-99	-99	-99	-99	-99	-99	-99	-99	-99	-99	-99	-99	-99
106	3,478809272	4,1303888	5,0062917	4,4679915	5,5446497	3,4705771	5,4338653	3,848543018	5,513491	3,9604878	3,9751031	4,4642472	5,18073995	-99	3,57987391
107	2,594089833	4,1072395	5,020225	4,2763873	5,5540389	3,6071677	4,8135249	3,988344804	5,5185584	4,1526989	4,0135405	4,4682241	5,183604955	-99	2,89733723
108	-99	-99	-99	-99	-99	-99	-99	-99	-99	-99	-99	-99	-99	-99	-99
109	-99	-99	-99	-99	-99	-99	-99	-99	-99	-99	-99	-99	-99	-99	-99
110	3,488985744	3,7641965	4,5107018	4,3582769	5,4142929	4,0390248	5,3511124	3,720378038	5,5029513	4,6890498	4,0491466	4,631997	5,434502193	-99	3,16187236
111	-99	-99	-99	-99	-99	-99	-99	-99	-99	-99	-99	-99	-99	-99	-99
112	-99	-99	-99	-99	-99	-99	-99	-99	-99	-99	-99	-99	-99	-99	-99
113	-99	-99	-99	-99	-99	-99	-99	-99	-99	-99	-99	-99	-99	-99	-99
114	3,533701605	3,8583544	4,9839344	4,3504713	5,6025147	3,5356526	5,1812417	4,088700997	5,5125263	3,8922034	4,1435546	4,4876803	5,289066276	4,31608226	2,43667088
115	-99	-99	-99	-99	-99	-99	-99	-99	-99	-99	-99	-99	-99	-99	-99
116	-99	-99	-99	-99	-99	-99	-99	-99	-99	-99	-99	-99	-99	-99	-99

117	-99	-99	-99	-99	-99	-99	-99	-99	-99	-99	-99	-99	-99	-99	-99
118	-99	-99	-99	-99	-99	-99	-99	-99	-99	-99	-99	-99	-99	-99	-99
119	-99	-99	-99	-99	-99	-99	-99	-99	-99	-99	-99	-99	-99	-99	-99
120	3,151080917	4,1762259	5,2244629	4,2193704	5,556675	2,8879544	5,234479	3,990863442	5,5039007	4,1101642	3,9584538	4,3653913	5,288193839	4,66434829	2,23056044
121	2,703072275	4,0209648	4,69187	3,9029619	5,6429129	3,1223768	4,5956307	4,206408904	5,5177717	4,1100044	3,8563298	3,8953853	5,048176359	-99	-99
122	3,394651548	3,9728165	4,9764862	4,403257	5,570778	3,9381825	4,7715145	4,353216736	5,5137859	4,2692827	3,9604034	4,4190026	5,195903191	-99	3,34592272
123	-99	-99	-99	-99	-99	-99	-99	-99	-99	-99	-99	-99	-99	-99	-99
124	-99	-99	-99	-99	-99	-99	-99	-99	-99	-99	-99	-99	-99	-99	-99
125	-99	-99	-99	-99	-99	-99	-99	-99	-99	-99	-99	-99	-99	-99	-99
126	-99	-99	-99	-99	-99	-99	-99	-99	-99	-99	-99	-99	-99	-99	-99
127	3,530120263	4,078267	4,8737171	4,4280261	5,5234553	4,3148189	5,412123	4,134626147	5,5185223	3,9706926	4,0834661	4,5758345	5,220868509	-99	3,73404857
128	-99	-99	-99	-99	-99	-99	-99	-99	-99	-99	-99	-99	-99	-99	-99
129	-99	-99	-99	-99	-99	-99	-99	-99	-99	-99	-99	-99	-99	-99	-99
130	-99	-99	-99	-99	-99	-99	-99	-99	-99	-99	-99	-99	-99	-99	-99
131	-99	-99	-99	-99	-99	-99	-99	-99	-99	-99	-99	-99	-99	-99	-99
132	-99	-99	-99	-99	-99	-99	-99	-99	-99	-99	-99	-99	-99	-99	-99
133	-99	-99	-99	-99	-99	-99	-99	-99	-99	-99	-99	-99	-99	-99	-99
134	-99	-99	-99	-99	-99	-99	-99	-99	-99	-99	-99	-99	-99	-99	-99
135	-99	-99	-99	-99	-99	-99	-99	-99	-99	-99	-99	-99	-99	-99	-99
136	-99	-99	-99	-99	-99	-99	-99	-99	-99	-99	-99	-99	-99	-99	-99
137	-99	-99	-99	-99	-99	-99	-99	-99	-99	-99	-99	-99	-99	-99	-99
138	-99	4,1321405	4,9135783	4,1766451	5,6377846	-99	4,5915792	4,031446535	5,5125525	3,9533843	3,9756765	4,5688668	4,972428847	-99	3,07346994
139	-99	-99	-99	-99	-99	-99	-99	-99	-99	-99	-99	-99	-99	-99	-99
140	3,118840726	4,117773	4,9183359	3,9321644	5,5826487	-99	4,3091105	3,958900903	5,5369822	4,0719017	3,8568493	4,308199	5,099344509	-99	2,64602427
141	3,230389987	4,131877	4,9273505	4,2163976	5,470327	3,9015121	5,443411	3,6539331	5,523005	4,1838943	3,9610952	4,2549185	5,148422464	-99	3,15010779
142	3,475914123	4,008458	4,8396075	4,2073667	5,5566475	3,6476931	5,0590065	3,994758881	5,5247943	4,233718	4,0084302	4,6739074	5,413734748	-99	3,21884752
143	3,53470816	3,8607771	4,8791331	4,2835085	5,4711361	3,7339855	4,8654983	3,830636815	5,5239149	4,3021316	4,0631446	4,3514351	5,279354934	-99	3,13133963
144	-99	-99	-99	-99	-99	-99	-99	-99	-99	-99	-99	-99	-99	-99	-99
145	3,119918633	3,9931751	4,7413173	4,3991619	5,4342122	2,9992392	5,3773254	3,841052313	5,5244343	4,0284971	3,936645	4,4987688	5,364144791	3,77498432	3,16121519
146	-99	-99	-99	-99	-99	-99	-99	-99	-99	-99	-99	-99	-99	-99	-99
147	3,436467877	3,8505236	4,7505279	4,2685823	5,5326319	3,9683784	5,4582627	3,928187598	5,5097945	4,2357034	4,0375912	4,5561583	5,337738552	-99	3,14516041
148	-99	-99	-99	-99	-99	-99	-99	-99	-99	-99	-99	-99	-99	-99	-99
149	-99	-99	-99	-99	-99	-99	-99	-99	-99	-99	-99	-99	-99	-99	-99
150	-99	-99	-99	-99	-99	-99	-99	-99	-99	-99	-99	-99	-99	-99	-99
151	-99	-99	-99	-99	-99	-99	-99	-99	-99	-99	-99	-99	-99	-99	-99
152	3,402908255	3,9109715	4,8059805	4,3690643	5,5948247	3,5025644	5,1942583	4,13879926	5,5165083	4,0047104	4,0747867	4,5528241	5,335774655	-99	3,63819137
153	-99	-99	-99	-99	-99	-99	-99	-99	-99	-99	-99	-99	-99	-99	-99
154	-99	-99	-99	-99	-99	-99	-99	-99	-99	-99	-99	-99	-99	-99	-99
155	-99	-99	-99	-99	-99	-99	-99	-99	-99	-99	-99	-99	-99	-99	-99
156	-99	-99	-99	-99	-99	-99	-99	-99	-99	-99	-99	-99	-99	-99	-99
157	-99	-99	-99	-99	-99	-99	-99	-99	-99	-99	-99	-99	-99	-99	-99
158	-99	-99	-99	-99	-99	-99	-99	-99	-99	-99	-99	-99	-99	-99	-99
159	3,214082311	4,0834708	4,7959002	4,0369535	5,5247831	3,9306855	5,6130542	4,125302675	5,5059353	4,2833291	3,882139	4,2774753	5,207361772	-99	3,38573026
160	3,348464378	4,0307289	4,7158485	4,407321	5,5392993	3,8732957	5,2256975	3,999579692	5,5132466	4,3161309	4,0343231	4,4859379	5,264428377	3,92593705	3,40816416
161	3,118003032	4,1293323	4,9447931	4,0770053	5,5181986	4,1831879	5,6567958	4,122265565	5,3663946	4,1958259	3,9089059	4,534964	5,2782655	-99	3,40623822
162	-99	-99	-99	-99	-99	-99	-99	-99	-99	-99	-99	-99	-99	-99	-99
163	-99	-99	-99	-99	-99	-99	-99	-99	-99	-99	-99	-99	-99	-99	-99
164	3,272741912	4,1859199	5,0544842	4,1328006	5,4856631	3,6686632	4,7698561	3,907489904	5,5254255	4,3598454	3,8476031	4,2236258	5,09195733	-99	3,09534108
165	-99	-99	-99	-99	-99	-99	-99	-99	-99	-99	-99	-99	-99	-99	-99
166	-99	-99	-99	-99	-99	-99	-99	-99	-99	-99	-99	-99	-99	-99	-99
167	3,820220187	3,7286328	4,5232699	4,2828173	5,5587785	3,8960357	5,1162461	3,89569564	5,4845523	4,2414718	3,982411	4,4196318	5,288340398	-99	3,09032912
168	-99	-99	-99	-99	-99	-99	-99	-99	-99	-99	-99	-99	-99	-99	-99
169	-99	-99	-99	-99	-99	-99	-99	-99	-99	-99	-99	-99	-99	-99	-99
170	-99	-99	-99	-99	-99	-99	-99	-99	-99	-99	-99	-99	-99	-99	-99
171	2,978147792	3,9299462	5,0203166	4,0581702	5,6956009	4,0221356	3,5991983	4,266432445	5,455079	4,5083196	4,0985702	4,5804514	5,175486933	-99	2,54505691
172	2,961304115	3,9867433	5,0195978	4,2160966	5,6173608	-99	4,7550841	4,268206046	5,5176895	3,7914683	3,8751369	4,277564	4,940938716	-99	3,29383144
173	-99	-99	-99	-99	-99	-99	-99	-99	-99	-99	-99	-99	-99	-99	-99,00000000
174	3,458301812	4,0452407	4,8693907	4,2185163	5,408105	3,698606	5,296804	3,59910407	5,5316436	4,1559112	3,8048298	4,3667295	5,298294584	4,80012462	3,11324862
175	3,454431779	4,1261827	5,0256284	4,149032	5,6013651	3,571435	5,1704379	4,142316261	5,5202895	4,1869399	3,9686216	4,5784452	5,301574048	-99	3,13283712
176	2,489280575	4,054705	4,8213477	4,1199894	5,6350104	3,4564403	4,155386	4,301786503	5,5395991	3,6024703	3,9789101	4,5473099	5,266727198	-99	2,89770391
177	3,36831984	4,1011211	5,0434862	4,1482079	5,6082466	3,741399	4,6955659	4,173953828	5,5231782	4,2325825	4,0335316	4,5392435	5,182106596	-99	3,26978101
178	-99	-99	-99	-99	-99	-99	-99	-99	-99	-99	-99	-99	-99	-99	-99
179	-99	-99	-99	-99	-99	-99	-99	-99	-99	-99	-99	-99	-99	-99	-99
180	-99	-99	-99	-99	-99	-99	-99	-99	-99	-99	-99	-99	-99	-99	-99
181	-99	-99	-99	-99	-99	-99	-99	-99	-99	-99	-99	-99	-99	-99	-99
182	3,519346232	3,8615369	4,9058534	4,2598353	5,5353704	2,7466998	5,220187	3,971381934	5,5226595	4,0352743	4,0554211	4,7208335	5,427112605	-99	3,55876912
183	3,332609638	3,8170567	4,7791657	4,2408732	5,5976379	3,4977135	4,946229	4,058342348	5,4977849	4,1317304	3,982378	4,1159248	5,08696342	-99	2,86245041
184	-99	-99	-99	-99	-99	-99	-99	-99	-99	-99	-99	-99	-99	-99	-99
185	-99	-99	-99	-99	-99	-99	-99	-99	-99	-99	-99	-99	-99	-99	-99
186	-99	-99	-99	-99	-99	-99	-99	-99	-99	-99	-99	-99	-99	-99	-99
187	-99	-99	-99	-99	-99	-99	-99	-99	-99	-99	-99	-99	-99	-99	-99
188	-99	-99	-99	-99	-99	-99	-99	-99	-99	-99	-99	-99	-99	-99	-99
189	-99	4,2928498	5,0175454	3,808107	5,4742883	-99	4,8986451	4,031470838	5,5289991	4,0925882	3,8315175	4,0434893	5,042573851	-99	2,79995731
190	-99	-99	-99	-99	-99	-99	-99	-99	-99	-99	-99	-99	-99	-99	-99
191	-99	-99	-99	-99	-99	-99	-99	-99	-99	-99	-99	-99	-99	-99	-99
192	-99	-99	-99	-99	-99	-99	-99	-99	-99	-99	-99	-99	-99	-99	-99
193	-99	-99	-99	-99	-99	-99	-99	-99	-99	-99	-99	-99	-99	-99	-99
194	-99	-99	-99	-99	-99	-99	-99	-99	-99	-99	-99	-99	-99	-99	-99
195	-99	-99	-99	-99	-99	-99	-99	-99	-99	-99	-99	-99	-99	-99	-99
196	-99	-99	-99	-99	-99	-99	-99	-99	-99	-99	-99	-99	-99	-99	-99
197	-99	-99	-99	-99	-99	-99	-99	-99	-99	-99	-99	-99	-99	-99	-99
198	3,316043919	4,1741677	4,9594264	4,2439364	5,5667415	3,8290455	4,8244604	3,989001725	5,5226511	4,2614491	3,9994387	4,6180066	5,190681437	3,76984128	2,51639332
199	2,775413922	4,0787873	4,9186349	4,0173467	5,6520156	3,2964425	4,8195289	4,116506668	5,5359535	4,0673612	4,0060651	4,5997744	5,236405367	-99	3,12648445
200	-99	-99	-99	-99	-99	-99	-99	-99	-99	-99	-99	-99	-99	-99	-99
201	-99	-99	-99	-99	-99	-99	-99	-99	-99	-99	-99	-99	-99	-99	-99
202	3,329638344	4,0846374	5,0946416	4,2519526	5,6330539	3,1368607	4,9133476	4,039007475	5,5120737	4,1011174	4,0117737	4,5578408	5,193258959	-99	2,91335916
203	2,944878226	3,9013121	4,7057637	4,0026892	5,6365661	3,8415659	4,5771864	4,163802907	5,518711	3,5100603	3,8582659	4,209393	5,028115854	-99	2,90166547
204	-99	-99	-99	-99	-99	-99	-99	-99	-99	-99	-99	-99	-99	-99	-99
205	-99	-99	-99	-99	-99	-99	-99	-99	-99	-99	-99	-99	-99	-99	-99
206	-99	-99	-99	-99	-99	-99	-99	-99	-99	-99	-99	-99	-99	-99	-99
207	-99	-99	-99	-99	-99	-99	-99	-99	-99	-99	-99	-99	-99	-99	-99
208	-99	-99	-99	-99	-99	-99	-99	-99	-99	-99	-99	-99	-99	-99	-99
209	3,284144312	3,7228827	4,6197509	4,3252907	5,5380629	4,1172524	5,1141689	4,035728363	5,528971	4,2262565	3,9758356	4,5349106	5,196288981	-99	3,23024337
210	-99	-99	-99	-99	-99	-99	-99	-99	-99	-99	-99	-99	-99	-99	-99
211	-99	-99	-99	-99	-99	-99	-99	-99	-99	-99	-99	-99	-99	-99	-99
212	3,319846676	3,9516598	4,7640466	4,0973466	5,6048597	3,4930663	4,8528393	4,364065275	5,5183545	4,1094832	4,0624725	4,4417686	5,173209387	-99	3,50532198
213	-99	-99	-99	-99	-99	-99	-99	-99	-99	-99	-99	-99	-99	-99	-99
214	3,441878789	4,0742119	4,9618003	4,2245413	5,4855526	3,8458044	4,8984139	3,950317248	5,5074382	4,0629902	3,8559048	3,9288927	4,889708037	-99	3,02683919
215	-99	-99	-99	-99	-99	-99	-99	-99	-99	-99	-99	-99	-99	-99	-99
216	3,320513589	4,0733784	4,8929899	4,4010679	5,5699059	3,2336853	5,6992395	4,070333049	5,524023	4,0345882	4,2034546	4,6426028	5,450148136	4,52653221	3,23408782
217	-99	-99	-99	-99	-99	-99	-99	-99	-99	-99	-99	-99	-99	-99	-99
218	-99	-99	-99	-99	-99	-99	-99	-99	-99	-99	-99	-99	-99	-99	-99
219	-99	-99	-99	-99	-99	-99	-99	-99	-99	-99	-99	-99	-99	-99	-99
220	3,400247668	3,8880494	4,9695404	4,1849311	5,5902321	3,7915587	4,6532173	4,336402072	5,5332937	4,0080382	4,0918957	4,3490446	5,25593426	-99	3,24586742
221	3,039490627	3,9550894	4,9032404	4,1532281	5,5865837	2,9000813	4,377703	4,253541245	5,508871	3,5075771	4,0128971	4,7297285	5,274213241	-99	3,45864764
222	2,893873167	3,9634926	4,7586325	4,4102241	5,6178274	3,7747448	4,9045568	4,166830927	5,5179172	4,4677315	3,9322316	4,3385027	4,98071916	-99	3,14505286
223	3,285271038	4,0778931	5,0662573	4,2627874	5,651223	3,3245393	5,0677351	4,249766963	5,5440337	3,7463543	4,0181866	4,5363175	5,162945175	-99	3,17473597
224	-99	-99	-99	-99	-99	-99	-99	-99	-99	-99	-99	-99	-99	-99	-99
225	3,353666844	4,153374	5,2531468	4,4922836	5,6156536	3,7246747	5,3172515	4,225843322	5,5341163	3,8753594	4,1811182	4,7098573	5,262431149	-99	3,21089295
226	3,378339095	4,0516655	4,9789104	4,6443145	5,573638	3,7502286	5,3261978	4,001234988	5,5210469	4,1900595	4,1303379	4,4814657	5,242788601	-99	2,68938817
227	-99	-99	-99	-99	-99	-99	-99	-99	-99	-99	-99	-99	-99	-99	-99
228	-99	-99	-99	-99	-99	-99	-99	-99	-99	-99	-99	-99	-99	-99	-99

229	-99	-99	-99	-99	-99	-99	-99	-99	-99	-99	-99	-99	-99	-99	-99
230	-99	-99	-99	-99	-99	-99	-99	-99	-99	-99	-99	-99	-99	-99	-99
231	-99	-99	-99	-99	-99	-99	-99	-99	-99	-99	-99	-99	-99	-99	-99
232	3,26113568	4,1891116	5,1212064	4,3541208	5,5721922	2,6892306	5,1161594	4,147895727	5,5251001	3,9377626	4,0754732	4,4260352	5,231250187	-99	2,97185740
233	3,381060629	3,9094019	4,9677982	4,4192221	5,5191921	3,5669953	5,6336783	3,801964967	5,5008861	4,2990926	4,0632491	4,6422853	5,329970048	-99	3,20663100
234	3,825870759	4,0694987	4,8879209	4,1035275	5,6509191	3,6887569	4,5974018	4,36518318	5,5128651	4,1404375	3,9685139	4,1594041	5,14812325	-99	3,26878285
235	3,311568827	4,0829086	5,1352155	4,2994975	5,6008413	2,826901	4,3437311	4,301226987	5,5439889	3,4448528	4,0156921	4,4283165	5,139893277	-99	3,14821704
236	-99	-99	-99	-99	-99	-99	-99	-99	-99	-99	-99	-99	-99	-99	-99
237	-99	-99	-99	-99	-99	-99	-99	-99	-99	-99	-99	-99	-99	-99	-99
238	-99	-99	-99	-99	-99	-99	-99	-99	-99	-99	-99	-99	-99	-99	-99
239	-99	-99	-99	-99	-99	-99	-99	-99	-99	-99	-99	-99	-99	-99	-99
240	-99	-99	-99	-99	-99	-99	-99	-99	-99	-99	-99	-99	-99	-99	-99
241	-99	-99	-99	-99	-99	-99	-99	-99	-99	-99	-99	-99	-99	-99	-99
242	-99	-99	-99	-99	-99	-99	-99	-99	-99	-99	-99	-99	-99	-99	-99
243	2,684305651	4,0994635	5,0802686	4,2040589	5,6631748	-99	4,8478228	4,365900175	5,5402134	3,6833428	4,0936882	4,619658	5,239431003	-99	3,35418134
244	3,302302252	3,9011325	5,2657272	4,3267323	5,645287	3,494708	4,9009942	4,325055645	5,5386224	4,0858651	4,1122895	4,2479299	5,036575919	-99	3,30354855
245	-99	-99	-99	-99	-99	-99	-99	-99	-99	-99	-99	-99	-99	-99	-99
246	3,414179474	3,8290236	4,8134185	4,4220836	5,721645	4,2312918	4,9225154	4,260385678	5,5172218	4,369179	4,2385438	4,7800791	5,388592462	-99	3,47026977
247	-99	-99	-99	-99	-99	-99	-99	-99	-99	-99	-99	-99	-99	-99	-99
248	3,088389169	4,2283976	4,9669804	4,1307608	5,5051861	2,7550956	4,5007634	3,938177008	5,5296982	3,833803	3,8594988	4,1644393	5,039699453	-99	2,90891857
249	3,652130168	3,9450569	4,9634557	4,1153281	5,6030335	3,9219944	4,5880212	4,296967422	5,5316291	4,3610158	4,0640842	4,517002	5,367198601	-99	3,55396031
250	-99	-99	-99	-99	-99	-99	-99	-99	-99	-99	-99	-99	-99	-99	-99
251	3,457593786	4,0144338	5,0376634	4,2723573	5,5385347	3,7422932	4,8573607	3,983264658	5,5048509	4,3060918	4,0872353	4,4950994	5,171992626	-99	3,06741049
252	-99	-99	-99	-99	-99	-99	-99	-99	-99	-99	-99	-99	-99	-99	-99
253	-99	-99	-99	-99	-99	-99	-99	-99	-99	-99	-99	-99	-99	-99	-99
254	-99	-99	-99	-99	-99	-99	-99	-99	-99	-99	-99	-99	-99	-99	-99
255	-99	-99	-99	-99	-99	-99	-99	-99	-99	-99	-99	-99	-99	-99	-99
256	3,047460967	4,122011	4,8674835	4,1352858	5,5882299	3,6631139	5,2549742	3,928870992	5,4948919	3,9141735	3,8290592	4,1303522	5,132058589	3,05595393	3,16953955
257	-99	-99	-99	-99	-99	-99	-99	-99	-99	-99	-99	-99	-99	-99	-99
258	-99	-99	-99	-99	-99	-99	-99	-99	-99	-99	-99	-99	-99	-99	-99
259	3,276777356	4,0034819	4,9853069	4,0250795	5,6280009	3,0644533	5,5202783	4,119431633	5,5372779	4,145772	4,0204662	4,456218	5,139955432	4,91739858	2,95700609
260	-99	-99	-99	-99	-99	-99	-99	-99	-99	-99	-99	-99	-99	-99	-99
261	2,972615044	3,8167483	4,8585622	4,2095083	5,5814639	3,197461	5,2056242	3,93895962	5,5035126	3,7396791	4,0234658	4,4182821	5,172218329	-99	3,06712400
262	3,233657174	4,1712373	5,0708782	4,2813238	5,6518643	3,7547897	4,4090298	4,392365829	5,5240341	4,029071	4,0932941	4,3396037	5,344756803	-99	3,35145255
263	-99	-99	-99	-99	-99	-99	-99	-99	-99	-99	-99	-99	-99	-99	-99
264	3,378877858	3,9247569	4,7433788	3,9353498	5,6018319	3,7852271	4,6418201	3,957970694	5,5059537	4,3312178	4,0329482	4,3634376	5,308501393	-99	3,07285219
265	3,489529483	3,9651709	4,8780111	4,0628765	5,6556672	3,8760487	4,22888	4,449052229	5,5329945	4,224107	4,0257331	4,1925822	5,120609613	-99	3,39859615
	46	47	48	49	50	51	52	53	54	55	56	57	58	59	60

**Location_ Treatment**	MPI_FGH_14_ Ds_M1	MPI_FGH_1 4_Ds_M2	MPI_FGH_1 4_Ds_M3	MPI_FGH_1 4_Ds_M4	MPI_FGH_1 4_Ds_M5	MPI_FGH_1 4_Ds_M6	MPI_FGH_1 4_Ds_M7	MPI_FGH_14_ Ds_M8	MPI_FGH_1 4_Ds_M9	MPI_FGH_1 4_Ds_M10	MPI_FGH_1 4_Ds_M11	MPI_FGH_1 4_Ds_M12	MPI_FGH_14_ Ds_M13	MPI_FGH_14_ Ds_M14	MPI_FGH_14_ Ds_M15
**Metabolites F1 individuals**	Salicylic acid-glucopyranoside	A148006-10:	Threonic acic	Ribitol	Malic acid	A250002-10:	Galactinol	Fumaric acid	Quinic acid, 3-caffeoyl-, trans-	A237001-10:	A217004-10:	A174001-10:	A197007-101	Raffinose	Galactaric acid
1	3,51116504	4,0119402	4,8565168	4,2899107	5,5234485	3,7732879	4,9278128	4,195149717	5,5351733	4,6051398	4,0257236	4,4897449	5,324675082	-99	3,78593774
2	-99	-99	-99	-99	-99	-99	-99	-99	-99	-99	-99	-99	-99	-99	-99
3	-99	-99	-99	-99	-99	-99	-99	-99	-99	-99	-99	-99	-99	-99	-99
4	-99	-99	-99	-99	-99	-99	-99	-99	-99	-99	-99	-99	-99	-99	-99
5	-99	-99	-99	-99	-99	-99	-99	-99	-99	-99	-99	-99	-99	-99	-99
6	3,523589509	3,8984781	4,5847575	4,2194599	5,4193692	3,601984	5,5535198	3,747968725	5,5051128	4,2676627	3,9553938	4,5292834	5,281702747	4,59257987	3,31127415
7	-99	-99	-99	-99	-99	-99	-99	-99	-99	-99	-99	-99	-99	-99	-99
8	-99	-99	-99	-99	-99	-99	-99	-99	-99	-99	-99	-99	-99	-99	-99
9	-99	-99	-99	-99	-99	-99	-99	-99	-99	-99	-99	-99	-99	-99	-99
10	-99	-99	-99	-99	-99	-99	-99	-99	-99	-99	-99	-99	-99	-99	-99
11	-99	-99	-99	-99	-99	-99	-99	-99	-99	-99	-99	-99	-99	-99	-99
12	-99	-99	-99	-99	-99	-99	-99	-99	-99	-99	-99	-99	-99	-99	-99
13	-99	-99	-99	-99	-99	-99	-99	-99	-99	-99	-99	-99	-99	-99	-99
14	-99	-99	-99	-99	-99	-99	-99	-99	-99	-99	-99	-99	-99	-99	-99
15	-99	-99	-99	-99	-99	-99	-99	-99	-99	-99	-99	-99	-99	-99	-99
16	3,21310603	4,0273861	4,8483912	4,428754	5,5128248	2,8092407	5,613643	3,995672463	5,4880632	4,3173835	3,9927629	4,7000014	5,440427383	4,54570430	3,20750705
17	3,506656176	4,2179059	5,4032752	3,9965275	5,4858171	3,8874505	4,7777511	3,891492516	5,5193795	4,0976881	3,9445254	4,4554776	5,34433477	-99	3,06453673
18	3,350299289	3,9477419	4,8838132	4,2831213	5,5225065	3,863018	5,2448826	4,303521623	5,5237169	4,1996778	4,0637462	4,5815909	5,329034875	-99	3,65072187
19	3,261985467	4,2712769	5,2267541	4,1765363	5,4322091	3,7775509	5,2006086	3,761402505	5,5131349	4,2772907	4,0184912	4,3818459	5,328302762	-99	3,12380525
20	-99	-99	-99	-99	-99	-99	-99	-99	-99	-99	-99	-99	-99	-99	-99
21	-99	-99	-99	-99	-99	-99	-99	-99	-99	-99	-99	-99	-99	-99	-99
22	-99	-99	-99	-99	-99	-99	-99	-99	-99	-99	-99	-99	-99	-99	-99
23	-99	-99	-99	-99	-99	-99	-99	-99	-99	-99	-99	-99	-99	-99	-99
24	3,275505401	4,1355961	4,946058	4,5161191	5,5243164	3,4683004	5,6014044	4,060871203	5,4572908	3,8975905	4,0993386	4,7071205	5,317654756	4,97156786	3,26661673
25	-99	-99	-99	-99	-99	-99	-99	-99	-99	-99	-99	-99	-99	-99	-99
26	-99	-99	-99	-99	-99	-99	-99	-99	-99	-99	-99	-99	-99	-99	-99
27	-99	-99	-99	-99	-99	-99	-99	-99	-99	-99	-99	-99	-99	-99	-99
28	-99	-99	-99	-99	-99	-99	-99	-99	-99	-99	-99	-99	-99	-99	-99
29	-99	-99	-99	-99	-99	-99	-99	-99	-99	-99	-99	-99	-99	-99	-99
30	-99	-99	-99	-99	-99	-99	-99	-99	-99	-99	-99	-99	-99	-99	-99
31	-99	-99	-99	-99	-99	-99	-99	-99	-99	-99	-99	-99	-99	-99	-99
32	-99	-99	-99	-99	-99	-99	-99	-99	-99	-99	-99	-99	-99	-99	-99
33	3,099115661	4,1702574	4,9045736	3,8983949	5,4882763	3,8412616	5,5475465	3,965919365	5,5186838	4,3319032	3,9751213	4,5706803	5,306917861	4,77943273	3,37552029
34	-99	-99	-99	-99	-99	-99	-99	-99	-99	-99	-99	-99	-99	-99	-99
35	3,277267952	4,0790838	4,9142355	4,4713232	5,4168989	3,1569579	5,534671	3,663236126	5,4757534	4,4295271	4,0984109	4,4830211	5,489546519	5,02286943	3,27541223
36	-99	-99	-99	-99	-99	-99	-99	-99	-99	-99	-99	-99	-99	-99	-99
37	-99	-99	-99	-99	-99	-99	-99	-99	-99	-99	-99	-99	-99	-99	-99
38	-99	-99	-99	-99	-99	-99	-99	-99	-99	-99	-99	-99	-99	-99	-99
39	-99	-99	-99	-99	-99	-99	-99	-99	-99	-99	-99	-99	-99	-99	-99
40	3,23365029	4,2459187	4,8632294	4,2973352	5,4718618	3,6514157	5,5248927	3,999179199	5,486333	4,5565543	3,9519437	4,6047529	5,25723111	5,12168155	3,06751977
41	-99	-99	-99	-99	-99	-99	-99	-99	-99	-99	-99	-99	-99	-99	-99
42	-99	-99	-99	-99	-99	-99	-99	-99	-99	-99	-99	-99	-99	-99	-99
43	3,483904338	4,0891451	5,0394542	4,3305294	5,4112355	3,5113634	5,2336331	3,960618436	5,5014927	4,187427	3,9671741	4,3402317	5,331333636	4,04299431	3,42767888
44	-99	-99	-99	-99	-99	-99	-99	-99	-99	-99	-99	-99	-99	-99	-99
45	-99	-99	-99	-99	-99	-99	-99	-99	-99	-99	-99	-99	-99	-99	-99
46	3,300513752	4,1295941	5,1146006	4,2492492	5,4984619	3,3179214	5,5191509	3,838744606	5,485793	4,2291745	4,0726368	4,6136534	5,400788695	4,84642045	3,00777712
47	-99	-99	-99	-99	-99	-99	-99	-99	-99	-99	-99	-99	-99	-99	-99
48	-99	-99	-99	-99	-99	-99	-99	-99	-99	-99	-99	-99	-99	-99	-99
49	3,41221975	3,9241241	4,8448086	4,3099138	5,3769707	3,6230314	5,6166522	3,757915907	5,4907545	4,1194013	4,0267193	4,4802231	5,3515033	4,40002317	3,68056443
50	3,302825191	4,2000947	4,9658441	4,1889221	5,5565944	-99	4,6194299	4,279503923	5,5280623	4,3035571	3,8569099	4,4995375	4,990129174	-99	3,03021903
51	3,473348546	4,2792344	4,9959516	4,1488862	5,525058	3,5798134	5,1954727	4,183028014	5,5006274	4,2456566	4,1291596	4,5900469	5,412582048	4,46898110	2,88471840
52	-99	-99	-99	-99	-99	-99	-99	-99	-99	-99	-99	-99	-99	-99	-99
53	-99	-99	-99	-99	-99	-99	-99	-99	-99	-99	-99	-99	-99	-99	-99
54	-99	-99	-99	-99	-99	-99	-99	-99	-99	-99	-99	-99	-99	-99	-99
55	-99	-99	-99	-99	-99	-99	-99	-99	-99	-99	-99	-99	-99	-99	-99
56	-99	-99	-99	-99	-99	-99	-99	-99	-99	-99	-99	-99	-99	-99	-99
57	3,677509846	4,1449954	4,8366926	4,0787546	5,3529271	3,6097268	5,5038536	3,781742993	5,4929678	4,4508956	3,9552331	4,4468379	5,302595788	3,87861972	3,82373032
58	3,513961668	4,0509145	5,118089	3,9700406	5,5424475	3,5098829	5,4709302	4,023024973	5,5216542	4,1789177	3,8928676	4,2778063	5,172766263	5,02086957	2,88878344
59	-99	-99	-99	-99	-99	-99	-99	-99	-99	-99	-99	-99	-99	-99	-99
60	-99	-99	-99	-99	-99	-99	-99	-99	-99	-99	-99	-99	-99	-99	-99
61	3,433109607	4,1922882	4,8711209	4,1414335	5,3481227	3,8351657	5,5419852	3,939575193	5,4779268	4,6277815	4,0287924	4,5170957	5,387316186	4,11414037	3,40003116
62	-99	-99	-99	-99	-99	-99	-99	-99	-99	-99	-99	-99	-99	-99	-99
63	-99	-99	-99	-99	-99	-99	-99	-99	-99	-99	-99	-99	-99	-99	-99
64	-99	-99	-99	-99	-99	-99	-99	-99	-99	-99	-99	-99	-99	-99	-99
65	3,293322661	3,8696872	4,8639421	4,2810426	5,4536909	3,5921968	5,5416442	3,81683214	5,5066723	4,3210135	3,9659888	4,3438938	5,319445882	4,81393582	3,05834636
66	-99	-99	-99	-99	-99	-99	-99	-99	-99	-99	-99	-99	-99	-99	-99
67	-99	-99	-99	-99	-99	-99	-99	-99	-99	-99	-99	-99	-99	-99	-99
68	-99	-99	-99	-99	-99	-99	-99	-99	-99	-99	-99	-99	-99	-99	-99

69	-99	-99	-99	-99	-99	-99	-99	-99	-99	-99	-99	-99	-99	-99	-99
70	-99	-99	-99	-99	-99	-99	-99	-99	-99	-99	-99	-99	-99	-99	-99
71	-99	-99	-99	-99	-99	-99	-99	-99	-99	-99	-99	-99	-99	-99	-99
72	-99	-99	-99	-99	-99	-99	-99	-99	-99	-99	-99	-99	-99	-99	-99
73	-99	-99	-99	-99	-99	-99	-99	-99	-99	-99	-99	-99	-99	-99	-99
74	-99	-99	-99	-99	-99	-99	-99	-99	-99	-99	-99	-99	-99	-99	-99
75	3,395949784	4,0338307	4,799828	4,3815193	5,5349701	3,65979	5,5586173	4,129980425	5,4897333	4,4199527	3,9968545	4,6746438	5,434030439	4,45988563	3,15266895
76	-99	-99	-99	-99	-99	-99	-99	-99	-99	-99	-99	-99	-99	-99	-99
77	-99	-99	-99	-99	-99	-99	-99	-99	-99	-99	-99	-99	-99	-99	-99
78	-99	-99	-99	-99	-99	-99	-99	-99	-99	-99	-99	-99	-99	-99	-99
79	-99	-99	-99	-99	-99	-99	-99	-99	-99	-99	-99	-99	-99	-99	-99
80	-99	-99	-99	-99	-99	-99	-99	-99	-99	-99	-99	-99	-99	-99	-99
81	-99	-99	-99	-99	-99	-99	-99	-99	-99	-99	-99	-99	-99	-99	-99
82	-99	-99	-99	-99	-99	-99	-99	-99	-99	-99	-99	-99	-99	-99	-99
83	-99	-99	-99	-99	-99	-99	-99	-99	-99	-99	-99	-99	-99	-99	-99
84	3,104011225	4,0836456	5,0706441	4,3779312	5,5392851	3,809534	5,5141217	4,046017282	5,4923337	4,1333179	4,1472508	4,7471997	5,439666027	4,22675426	3,23582128
85	-99	-99	-99	-99	-99	-99	-99	-99	-99	-99	-99	-99	-99	-99	-99
86	-99	-99	-99	-99	-99	-99	-99	-99	-99	-99	-99	-99	-99	-99	-99
87	3,22022164	4,2310402	5,1176211	4,2486862	5,3796709	3,4120601	5,4794131	3,751276474	5,4846502	4,1876158	4,0089173	4,5082596	5,384061418	4,81350864	3,09052433
88	3,364018348	4,0672226	4,8618941	3,9860945	5,5296711	3,851498	5,0017225	4,22044655	5,5248455	4,3000334	3,8948642	4,3100188	5,146689707	-99	3,39054493
89	3,109466166	4,1419045	5,1223722	4,2055967	5,5014274	3,7094984	5,4234606	3,971041178	5,5227958	4,371644	4,0008877	4,5268326	5,261931479	3,79766696	3,09464367
90	-99	-99	-99	-99	-99	-99	-99	-99	-99	-99	-99	-99	-99	-99	-99
91	-99	-99	-99	-99	-99	-99	-99	-99	-99	-99	-99	-99	-99	-99	-99
92	-99	-99	-99	-99	-99	-99	-99	-99	-99	-99	-99	-99	-99	-99	-99
93	-99	-99	-99	-99	-99	-99	-99	-99	-99	-99	-99	-99	-99	-99	-99
94	-99	-99	-99	-99	-99	-99	-99	-99	-99	-99	-99	-99	-99	-99	-99
95	-99	-99	-99	-99	-99	-99	-99	-99	-99	-99	-99	-99	-99	-99	-99
96	-99	-99	-99	-99	-99	-99	-99	-99	-99	-99	-99	-99	-99	-99	-99
97	-99	-99	-99	-99	-99	-99	-99	-99	-99	-99	-99	-99	-99	-99	-99
98	-99	-99	-99	-99	-99	-99	-99	-99	-99	-99	-99	-99	-99	-99	-99
99	3,186969802	4,0127806	5,0378679	4,2501671	5,4770334	2,9230318	5,2704413	3,843532284	5,4943221	4,3307436	4,0433169	4,4782367	5,421839664	3,74822378	3,25635078
100	3,462977289	4,1494795	5,1124558	4,1446822	5,4042925	3,6748065	5,5795847	3,587535585	5,4782282	4,1865866	4,0157251	4,247168	5,303403614	4,86655088	2,78467559
101	-99	-99	-99	-99	-99	-99	-99	-99	-99	-99	-99	-99	-99	-99	-99
102	-99	-99	-99	-99	-99	-99	-99	-99	-99	-99	-99	-99	-99	-99	-99
103	3,240980367	4,3206979	5,297958	4,0648338	5,4763779	2,9606106	5,2429456	4,251178208	5,5088105	4,0274789	3,853016	4,2401229	5,219542934	4,31204244	3,20570117
104	3,309400266	4,2210683	5,0442089	4,312159	5,4074973	3,8595947	5,5828543	3,849818573	5,4740094	4,3106339	4,0827752	4,6340761	5,467954694	4,88933899	2,89112552
105	-99	-99	-99	-99	-99	-99	-99	-99	-99	-99	-99	-99	-99	-99	-99
106	3,382125055	4,2061543	5,0412456	4,5444238	5,4299914	3,6659253	5,5276996	3,988171767	5,4636636	4,2517964	3,9759057	4,5935753	5,388067645	4,12842659	3,69052290
107	3,056620433	3,9258735	5,0265182	4,2665482	5,4868664	3,8898065	5,4563521	4,27396298	5,4483928	4,2207391	3,9832706	4,7116037	5,174862417	4,61762605	3,05257463
108	-99	-99	-99	-99	-99	-99	-99	-99	-99	-99	-99	-99	-99	-99	-99
109	-99	-99	-99	-99	-99	-99	-99	-99	-99	-99	-99	-99	-99	-99	-99
110	3,386082736	4,1567712	5,0206427	4,2052441	5,5459401	3,9438581	5,0146137	4,070403147	5,5053519	4,4558054	3,9489414	4,7166021	5,320724173	3,67750326	3,15356861
111	-99	-99	-99	-99	-99	-99	-99	-99	-99	-99	-99	-99	-99	-99	-99
112	-99	-99	-99	-99	-99	-99	-99	-99	-99	-99	-99	-99	-99	-99	-99
113	-99	-99	-99	-99	-99	-99	-99	-99	-99	-99	-99	-99	-99	-99	-99
114	3,36624276	3,9022287	4,888185	3,9713174	5,5234052	3,7035595	4,5583189	4,157120459	5,5384008	4,2283235	3,987737	4,2431245	5,098646973	-99	2,35173911
115	-99	-99	-99	-99	-99	-99	-99	-99	-99	-99	-99	-99	-99	-99	-99
116	-99	-99	-99	-99	-99	-99	-99	-99	-99	-99	-99	-99	-99	-99	-99
117	-99	-99	-99	-99	-99	-99	-99	-99	-99	-99	-99	-99	-99	-99	-99
118	-99	-99	-99	-99	-99	-99	-99	-99	-99	-99	-99	-99	-99	-99	-99
119	-99	-99	-99	-99	-99	-99	-99	-99	-99	-99	-99	-99	-99	-99	-99
120	3,07468469	4,1279206	5,0887208	4,3843572	5,5281065	2,9556499	5,3575139	3,912883393	5,4964998	4,1126827	3,9371451	4,4200985	5,307866988	4,37080420	2,24439685
121	3,397620003	4,2227075	5,0369368	3,9781195	5,4966901	3,582305	5,4259771	3,9484659	5,5096079	4,3121744	3,926471	4,4427753	5,233556869	4,38933354	2,86197148
122	3,388578269	4,0118853	4,9116347	4,313064	5,5126284	3,6786216	5,2556059	4,142849212	5,527473	4,1734165	3,8914474	4,4264288	5,179872627	4,38348186	3,46342746
123	-99	-99	-99	-99	-99	-99	-99	-99	-99	-99	-99	-99	-99	-99	-99
124	-99	-99	-99	-99	-99	-99	-99	-99	-99	-99	-99	-99	-99	-99	-99
125	-99	-99	-99	-99	-99	-99	-99	-99	-99	-99	-99	-99	-99	-99	-99
126	-99	-99	-99	-99	-99	-99	-99	-99	-99	-99	-99	-99	-99	-99	-99
127	3,476538648	4,0966717	4,8410875	4,4889152	5,4224656	4,27338	5,5794453	3,809188346	5,5120842	4,2305323	4,0122063	4,6193506	5,398574585	4,88957469	3,70842410
128	-99	-99	-99	-99	-99	-99	-99	-99	-99	-99	-99	-99	-99	-99	-99
129	-99	-99	-99	-99	-99	-99	-99	-99	-99	-99	-99	-99	-99	-99	-99
130	-99	-99	-99	-99	-99	-99	-99	-99	-99	-99	-99	-99	-99	-99	-99
131	-99	-99	-99	-99	-99	-99	-99	-99	-99	-99	-99	-99	-99	-99	-99
132	-99	-99	-99	-99	-99	-99	-99	-99	-99	-99	-99	-99	-99	-99	-99
133	-99	-99	-99	-99	-99	-99	-99	-99	-99	-99	-99	-99	-99	-99	-99
134	-99	-99	-99	-99	-99	-99	-99	-99	-99	-99	-99	-99	-99	-99	-99
135	-99	-99	-99	-99	-99	-99	-99	-99	-99	-99	-99	-99	-99	-99	-99
136	-99	-99	-99	-99	-99	-99	-99	-99	-99	-99	-99	-99	-99	-99	-99
137	-99	-99	-99	-99	-99	-99	-99	-99	-99	-99	-99	-99	-99	-99	-99
138	3,006804882	4,1345163	4,9395293	4,1307654	5,4038313	3,4519918	5,676644	3,89310837	5,5105945	4,2774861	4,0019116	4,5830859	5,244075668	4,86372515	3,33194617
139	-99	-99	-99	-99	-99	-99	-99	-99	-99	-99	-99	-99	-99	-99	-99
140	3,394721735	4,3044615	5,0451975	4,3170877	5,4012522	3,5584017	5,3987534	3,681639714	5,508489	4,5437976	4,0459783	4,4093499	5,427336307	-99	3,11430083
141	3,273912022	4,1456413	5,0101674	4,0886943	5,4593251	3,9792657	5,5281488	3,829532069	5,5240606	4,3136371	3,9618191	4,5173963	5,323843786	4,63991091	3,17757824
142	3,434288597	4,0809964	4,620527	4,0250496	5,4395877	3,9547672	5,3605002	3,630407849	5,5123689	4,7546356	4,034036	4,713349	5,493949335	-99	3,35939906
143	3,528398854	4,0042298	4,9617927	4,2832106	5,4827293	3,9817566	5,53607	3,987967297	5,4791954	4,3085815	4,0846718	4,5588445	5,400905951	4,72128250	3,22130948
144	-99	-99	-99	-99	-99	-99	-99	-99	-99	-99	-99	-99	-99	-99	-99
145	3,195493017	4,210412	5,0868412	4,2820094	5,4771931	3,035611	5,0249947	4,025381724	5,4805443	4,3687086	3,9252234	4,6693138	5,32032783	3,81859263	3,26162955
146	-99	-99	-99	-99	-99	-99	-99	-99	-99	-99	-99	-99	-99	-99	-99
147	-99	3,9967625	5,1740099	4,3203483	6,0211922	4,0581642	5,3318929	4,237362133	5,3574106	4,3724235	4,1467805	4,7993981	5,36082932	-99	3,19324791
148	-99	-99	-99	-99	-99	-99	-99	-99	-99	-99	-99	-99	-99	-99	-99
149	-99	-99	-99	-99	-99	-99	-99	-99	-99	-99	-99	-99	-99	-99	-99
150	-99	-99	-99	-99	-99	-99	-99	-99	-99	-99	-99	-99	-99	-99	-99
151	-99	-99	-99	-99	-99	-99	-99	-99	-99	-99	-99	-99	-99	-99	-99
152	3,379214584	4,2147412	5,0952953	4,5266622	5,4129695	3,5937146	5,6589171	3,920912401	5,4911542	4,150623	4,0535668	4,566137	5,464121018	5,10645374	3,41518174
153	-99	-99	-99	-99	-99	-99	-99	-99	-99	-99	-99	-99	-99	-99	-99
154	-99	-99	-99	-99	-99	-99	-99	-99	-99	-99	-99	-99	-99	-99	-99
155	-99	-99	-99	-99	-99	-99	-99	-99	-99	-99	-99	-99	-99	-99	-99
156	-99	-99	-99	-99	-99	-99	-99	-99	-99	-99	-99	-99	-99	-99	-99
157	-99	-99	-99	-99	-99	-99	-99	-99	-99	-99	-99	-99	-99	-99	-99
158	-99	-99	-99	-99	-99	-99	-99	-99	-99	-99	-99	-99	-99	-99	-99
159	3,206392977	4,0502968	4,8822648	4,1826319	5,4959076	4,0057824	5,5676417	4,006306347	5,4792619	4,2103123	3,9121458	4,4142255	5,236605983	4,87403601	3,40387037
160	3,266487404	4,2613723	5,0393986	4,119147	5,4728243	3,9346484	5,2735834	3,903357304	5,5003774	4,3635416	3,9158358	4,4717995	5,223433042	4,35927081	3,25638164
161	3,284466985	4,0971163	4,9291233	4,0022857	5,5143284	4,0657114	5,5095865	4,058264665	5,4475643	4,4786257	3,8609288	4,170918	5,269278667	-99	3,26131378
162	-99	-99	-99	-99	-99	-99	-99	-99	-99	-99	-99	-99	-99	-99	-99
163	-99	-99	-99	-99	-99	-99	-99	-99	-99	-99	-99	-99	-99	-99	-99
164	3,013477228	4,4118602	5,3225027	4,2888507	5,5857361	3,5868272	5,0143272	4,118023626	5,5288002	4,3580525	3,9147914	4,540107	5,265706044	-99	3,24159754
165	-99	-99	-99	-99	-99	-99	-99	-99	-99	-99	-99	-99	-99	-99	-99
166	-99	-99	-99	-99	-99	-99	-99	-99	-99	-99	-99	-99	-99	-99	-99
167	3,859472925	4,0145077	4,934255	4,1773988	5,4539879	3,804482	5,1329316	3,923151322	5,4965297	4,4415678	3,8926866	4,4912502	5,335389841	-99	3,18115063
168	-99	-99	-99	-99	-99	-99	-99	-99	-99	-99	-99	-99	-99	-99	-99
169	-99	-99	-99	-99	-99	-99	-99	-99	-99	-99	-99	-99	-99	-99	-99
170	-99	-99	-99	-99	-99	-99	-99	-99	-99	-99	-99	-99	-99	-99	-99
171	3,288363452	4,1598166	5,3089887	4,3504437	5,5126439	3,6834314	5,6284885	4,062011691	5,5163139	4,52064	4,1010292	4,5683169	5,345155289	4,16115037	3,17455682
172	3,028492969	4,0691873	4,9411215	4,1526974	5,4529293	2,4848204	5,4635738	4,061195773	5,529723	3,9040511	3,7505941	4,3016076	5,137819915	4,39712245	3,10484003
173	-99	-99	-99	-99	-99	-99	-99	-99	-99	-99	-99	-99	-99	-99,00000000	-99,00000000
174	3,429386323	3,7625675	4,5143824	3,9952308	5,4183141	3,8816392	5,4783697	3,534705465	5,4936744	4,2154575	3,8676573	4,4904288	5,402862454	5,29356488	3,09031447
175	3,188443731	3,8703132	4,8402871	4,4097835	5,5052216	3,4372762	5,5825384	3,98892614	5,51048	4,1927479	3,9778472	4,6408609	5,358888122	4,08824468	3,12603696
176	3,226098807	4,1933532	4,9261853	4,3975002	5,5783452	3,6722707	5,4095175	4,353271692	5,5120435	4,1866061	4,1492801	4,7562474	5,339634251	4,27307873	3,18826649
177	3,533369237	4,0234272	4,8986554	4,1182535	5,553748	3,6345707	5,5339892	4,029122728	5,4847503	4,4360735	4,0284795	4,5840503	5,292688899	-99	3,26591323
178	-99	-99	-99	-99	-99	-99	-99	-99	-99	-99	-99	-99	-99	-99	-99
179	-99	-99	-99	-99	-99	-99	-99	-99	-99	-99	-99	-99	-99	-99	-99
180	-99	-99	-99	-99	-99	-99	-99	-99	-99	-99	-99	-99	-99	-99	-99

181	-99	-99	-99	-99	-99	-99	-99	-99	-99	-99	-99	-99	-99	-99	-99
182	3,352906484	4,0687009	4,9736527	4,1652972	5,4803948	3,126378	5,3684827	3,785371107	5,4988069	4,2336672	4,0029814	4,6921934	5,422278335	4,48664887	3,51744317
183	3,403844952	4,0508214	5,0696318	4,3305115	5,4639828	3,3207406	5,5581203	3,893712301	5,5009472	3,8887817	4,0538868	4,4951902	5,337417531	4,42804381	3,17089806
184	-99	-99	-99	-99	-99	-99	-99	-99	-99	-99	-99	-99	-99	-99	-99
185	-99	-99	-99	-99	-99	-99	-99	-99	-99	-99	-99	-99	-99	-99	-99
186	-99	-99	-99	-99	-99	-99	-99	-99	-99	-99	-99	-99	-99	-99	-99
187	-99	-99	-99	-99	-99	-99	-99	-99	-99	-99	-99	-99	-99	-99	-99
188	-99	-99	-99	-99	-99	-99	-99	-99	-99	-99	-99	-99	-99	-99	-99
189	3,143187313	4,2542828	5,1814127	4,0361433	5,4801645	3,1686576	5,6433862	4,127221931	5,4961	4,1377782	4,0463856	4,3873509	5,264153143	3,72882405	3,25964231
190	-99	-99	-99	-99	-99	-99	-99	-99	-99	-99	-99	-99	-99	-99	-99
191	-99	-99	-99	-99	-99	-99	-99	-99	-99	-99	-99	-99	-99	-99	-99
192	-99	-99	-99	-99	-99	-99	-99	-99	-99	-99	-99	-99	-99	-99	-99
193	-99	-99	-99	-99	-99	-99	-99	-99	-99	-99	-99	-99	-99	-99	-99
194	-99	-99	-99	-99	-99	-99	-99	-99	-99	-99	-99	-99	-99	-99	-99
195	-99	-99	-99	-99	-99	-99	-99	-99	-99	-99	-99	-99	-99	-99	-99
196	-99	-99	-99	-99	-99	-99	-99	-99	-99	-99	-99	-99	-99	-99	-99
197	-99	-99	-99	-99	-99	-99	-99	-99	-99	-99	-99	-99	-99	-99	-99
198	3,436637974	4,0761791	4,8552944	4,3065065	5,5087302	4,0548034	5,4666547	3,916006926	5,4978097	4,4614283	4,0757382	4,7563116	5,343425706	4,97353709	2,99991835
199	3,383678128	4,1056491	4,9457682	4,0309161	5,5690409	3,385705	5,0150434	4,134064387	5,5299285	4,1926603	3,9018833	4,4778059	5,194152059	-99	3,22679549
200	-99	-99	-99	-99	-99	-99	-99	-99	-99	-99	-99	-99	-99	-99	-99
201	-99	-99	-99	-99	-99	-99	-99	-99	-99	-99	-99	-99	-99	-99	-99
202	3,193556197	4,4690523	5,3045465	3,9482561	5,3888253	2,8521704	5,0351637	3,897286733	5,4895687	4,4180521	3,8926331	4,4273717	5,279998454	4,02709524	3,00138967
203	3,564594573	3,889337	4,7092853	4,2362766	5,4309218	3,9818999	5,4671535	3,805665769	5,4651615	4,1071534	3,9793979	4,397522	5,347324016	5,50678887	3,13516012
204	-99	-99	-99	-99	-99	-99	-99	-99	-99	-99	-99	-99	-99	-99	-99
205	-99	-99	-99	-99	-99	-99	-99	-99	-99	-99	-99	-99	-99	-99	-99
206	-99	-99	-99	-99	-99	-99	-99	-99	-99	-99	-99	-99	-99	-99	-99
207	-99	-99	-99	-99	-99	-99	-99	-99	-99	-99	-99	-99	-99	-99	-99
208	-99	-99	-99	-99	-99	-99	-99	-99	-99	-99	-99	-99	-99	-99	-99
209	3,35028472	3,9249966	4,9225891	4,3328251	5,40998	4,1317648	5,6414387	3,891436614	5,4891934	4,4725242	3,9276108	4,5538949	5,341582282	4,48453300	3,36247040
210	-99	-99	-99	-99	-99	-99	-99	-99	-99	-99	-99	-99	-99	-99	-99
211	-99	-99	-99	-99	-99	-99	-99	-99	-99	-99	-99	-99	-99	-99	-99
212	3,524192327	4,1991172	4,8468685	3,9421239	5,5701042	3,598423	4,9412846	4,217938131	5,5068822	4,5842662	4,080138	4,4774174	5,155365086	-99	3,59831459
213	-99	-99	-99	-99	-99	-99	-99	-99	-99	-99	-99	-99	-99	-99	-99
214	3,504528552	3,833733	4,557192	4,6380715	5,4803163	4,0398319	5,4717005	4,191077167	5,4724153	3,9958433	4,1106262	4,6691158	5,497549576	4,67042225	3,28069282
215	-99	-99	-99	-99	-99	-99	-99	-99	-99	-99	-99	-99	-99	-99	-99
216	3,276849373	4,0283321	4,7162868	4,359837	5,5133357	3,47028	5,7566644	4,012034245	5,5150864	4,3465011	4,0751078	4,6830125	5,35484002	4,96231054	3,27378772
217	-99	-99	-99	-99	-99	-99	-99	-99	-99	-99	-99	-99	-99	-99	-99
218	-99	-99	-99	-99	-99	-99	-99	-99	-99	-99	-99	-99	-99	-99	-99
219	-99	-99	-99	-99	-99	-99	-99	-99	-99	-99	-99	-99	-99	-99	-99
220	3,373653484	4,0444143	5,0117587	4,1178901	5,3559368	3,7842194	5,5182827	3,583175657	5,5042068	4,3589306	3,9919476	4,4260101	5,341057521	4,12492392	3,38345956
221	3,243178703	4,2774845	5,1871528	3,9801128	5,4425297	3,0912573	5,1533569	3,903318476	5,504884	4,2565905	3,8910921	4,4981733	5,370076934	-99	3,36640974
222	3,382565257	3,9197014	4,6315917	4,519035	5,4582368	3,7950808	5,6068278	3,904124253	5,4915038	4,4200861	3,9413252	4,5342395	5,207865667	4,91087133	3,39268331
223	3,21055797	4,5164439	5,378127	3,9472417	5,4658917	3,5819732	5,3436826	3,893933861	5,5292592	4,2884772	3,9421975	4,3477945	5,250116242	3,90911602	2,87223607
224	-99	-99	-99	-99	-99	-99	-99	-99	-99	-99	-99	-99	-99	-99	-99
225	3,318775947	3,6995992	4,7515433	4,6972984	5,5685654	3,7460326	5,6180137	4,223941956	5,4933543	3,9851613	4,2096375	4,784793	5,450009403	4,36033426	3,32803582
226	3,525509255	4,2807573	5,1350776	4,5148815	5,4757886	3,7602055	5,3907038	3,981693292	5,4977002	4,5668289	4,0276967	4,5556425	5,257730472	3,63589577	3,03627525
227	-99	-99	-99	-99	-99	-99	-99	-99	-99	-99	-99	-99	-99	-99	-99
228	-99	-99	-99	-99	-99	-99	-99	-99	-99	-99	-99	-99	-99	-99	-99
229	-99	-99	-99	-99	-99	-99	-99	-99	-99	-99	-99	-99	-99	-99	-99
230	-99	-99	-99	-99	-99	-99	-99	-99	-99	-99	-99	-99	-99	-99	-99
231	-99	-99	-99	-99	-99	-99	-99	-99	-99	-99	-99	-99	-99	-99	-99
232	3,32970012	4,114894	5,2901653	4,0928927	5,3654637	3,0993422	5,5826088	3,796366241	5,5123391	4,2040037	3,9557946	4,3184298	5,211535608	4,99215819	3,07450651
233	3,292568546	3,9841165	4,8974818	4,4655046	5,3330398	3,4052904	5,4829346	3,791473536	5,4746872	4,241625	4,0244292	4,4712157	5,405531434	4,65239397	3,29581571
234	3,823522201	4,148339	5,1219917	3,9865805	5,441183	3,7125402	5,3829361	3,956151085	5,5195901	4,1452286	3,8941121	4,3722841	5,255728819	4,70362768	3,28673755
235	3,396676548	4,0376811	4,9906553	4,4098404	5,36656	3,1356148	5,5315119	3,811425559	5,4731915	4,1395941	4,0471859	4,4641365	5,392582139	5,02554744	3,15979165
236	-99	-99	-99	-99	-99	-99	-99	-99	-99	-99	-99	-99	-99	-99	-99
237	-99	-99	-99	-99	-99	-99	-99	-99	-99	-99	-99	-99	-99	-99	-99
238	-99	-99	-99	-99	-99	-99	-99	-99	-99	-99	-99	-99	-99	-99	-99
239	-99	-99	-99	-99	-99	-99	-99	-99	-99	-99	-99	-99	-99	-99	-99
240	-99	-99	-99	-99	-99	-99	-99	-99	-99	-99	-99	-99	-99	-99	-99
241	-99	-99	-99	-99	-99	-99	-99	-99	-99	-99	-99	-99	-99	-99	-99
242	-99	-99	-99	-99	-99	-99	-99	-99	-99	-99	-99	-99	-99	-99	-99
243	3,400597602	4,0434823	5,0361748	4,2793891	5,5251434	2,5922746	5,5942598	4,061830933	5,5059914	4,3320293	4,1509194	4,679553	5,389415201	4,39456264	3,41239417
244	3,26042895	3,7586332	4,7233551	4,3381903	5,4449941	3,5652233	5,5064803	3,865409277	5,4821441	4,4333197	4,1447035	4,4279438	5,334224857	4,33897264	3,42586764
245	-99	-99	-99	-99	-99	-99	-99	-99	-99	-99	-99	-99	-99	-99	-99
246	3,383039566	3,9442252	4,8099978	4,6163345	5,5259826	4,2012319	5,6513779	4,063201791	5,5042962	4,1441717	4,1347592	4,6913329	5,455889616	4,48176281	3,27363425
247	-99	-99	-99	-99	-99	-99	-99	-99	-99	-99	-99	-99	-99	-99	-99
248	3,343673172	4,1227715	4,7824309	4,2848331	5,3987213	3,1421175	5,5712284	3,893505436	5,4609049	4,1305325	3,9627285	4,5405707	5,371978499	4,52522671	3,17659589
249	3,494375317	3,9901903	4,8684796	4,1168153	5,5863219	3,8519071	4,7620808	4,419008827	5,5206314	4,4833703	3,9799167	4,5706895	5,37388875	-99	3,61167062
250	-99	-99	-99	-99	-99	-99	-99	-99	-99	-99	-99	-99	-99	-99	-99
251	3,327898664	4,3958444	5,0251756	4,3342123	5,2998874	3,8140681	5,4255061	3,469926436	5,4577718	3,2562346	4,0257617	3,4944292	4,677697444	-99	-99
252	-99	-99	-99	-99	-99	-99	-99	-99	-99	-99	-99	-99	-99	-99	-99
253	-99	-99	-99	-99	-99	-99	-99	-99	-99	-99	-99	-99	-99	-99	-99
254	-99	-99	-99	-99	-99	-99	-99	-99	-99	-99	-99	-99	-99	-99	-99
255	-99	-99	-99	-99	-99	-99	-99	-99	-99	-99	-99	-99	-99	-99	-99
256	3,24381103	4,2057104	5,1281866	4,4028304	5,4215334	4,0147586	5,4792693	3,937734296	5,4785102	4,2924575	4,0287962	4,5668396	5,447743654	5,04680929	3,32727530
257	-99	-99	-99	-99	-99	-99	-99	-99	-99	-99	-99	-99	-99	-99	-99
258	-99	-99	-99	-99	-99	-99	-99	-99	-99	-99	-99	-99	-99	-99	-99
259	3,350368378	4,0697205	5,0482373	4,0014733	5,5680419	3,1701935	5,4509669	4,142248644	5,5390861	4,3181724	3,9713138	4,4409451	5,218130464	4,80592125	3,03479719
260	-99	-99	-99	-99	-99	-99	-99	-99	-99	-99	-99	-99	-99	-99	-99
261	3,323601006	4,0530074	4,9369458	4,2598961	5,5599317	3,8783737	5,3809719	4,109297095	5,5192714	4,3097631	4,0503698	4,7189474	5,398005694	4,43654927	3,35056921
262	3,293402322	4,1909533	5,0751026	4,413878	5,6006452	4,0576189	5,3875652	4,285548871	5,503703	4,0907819	4,1550456	4,7418412	5,412856547	-99	3,48687417
263	-99	-99	-99	-99	-99	-99	-99	-99	-99	-99	-99	-99	-99	-99	-99
264	3,283802567	4,0997322	5,1045234	4,1493961	5,5501734	3,8370377	5,7055378	4,02974253	5,5258481	4,2460077	4,1283589	4,6212971	5,381697228	4,99542972	3,18007991
265	3,537979663	4,0961023	4,9417938	4,085704	5,4712862	3,8170442	5,2231465	4,175224516	5,5301597	4,1519895	4,0389878	4,2501872	5,225963217	3,78638453	3,34756828

Table S8 Selected metabolites previously identified as drought responsive [13] and respective mQTL overlapping with DRYM QTL.

Metabolite[1]	LG1[2]		LG2		LG3		LG4		LG5		LG6		LG7		LG8		LG9		LG10		LG11		LG12		Number of
	1A	1R	2A	2R	3A	3R	4A	4R	5A	5R	6A	6R	7A	7R	8A	8R	9A	9R	10A	10R	11A	11R	12A	12R	overlaps
Ribitol	2Ds, 2Co	1Ds, 1Co																							6
A217004-101		1Ds												1Ds, 1Co		1Ds									4
Fumaric acid	1Co			1Co												1Co						1Ds			4
Salicylic acid-glucopyranoside				1Ds, 1Co	1Ds																		1Ds		4
Malic acid																1Co						1Co		1Ds	3
A197007-101		1Co						1Ds								1Co									3
A237001-101				1Ds																				1Ds	2
Quinic acid, 3-caffeoyl-, trans-																						1Co	1Co		2
Galactaric acid		1Ds																							1
Galactinol														1Co											1
Threonic acid																							1Co		1
Raffinose																									0
A148006-101																									0
A174001-101																									0
A250002-101																									0

[1]Metabolites are sorted according to the descending number of mQTL overlapping with DRYM QTL.

[2]grey, no DRYM QTL on the linkage group (LG); Co, overlapping mQTL under control conditions; Ds, overlapping mQTL under drought stress

Table S9: AFLP primer pairs used for the development of the genetic maps.

E-name	E-primer	M-name	M-primer
E32	E-AAC	M47	M-CAA
E32	E-AAC	M48	M-CAC
E32	E-AAC	M49	M-CAG
E32	E-AAC	M50	M-CAT
E32	E-AAC	M60	M-CTC
E32	E-AAC	M61	M-CTG
E32	E-AAC	M62	M-CTT
E33	E-AAG	M47	M-CAA
E33	E-AAG	M48	M-CAC
E33	E-AAG	M49	M-CAG
E33	E-AAG	M50	M-CAT
E33	E-AAG	M60	M-CTC
E33	E-AAG	M61	M-CTG
E33	E-AAG	M62	M-CTT
E35	E-ACA	M47	M-CAA
E35	E-ACA	M48	M-CAC
E35	E-ACA	M49	M-CAG
E35	E-ACA	M50	M-CAT
E35	E-ACA	M60	M-CTC
E35	E-ACA	M61	M-CTG
E35	E-ACA	M62	M-CTT
E36	E-ACC	M47	M-CAA
E36	E-ACC	M50	M-CAT
E36	E-ACC	M59	M-CTA
E36	E-ACC	M62	M-CTT
E37	E-ACG	M47	M-CAA
E37	E-ACG	M50	M-CAT
E38	E-ACT	M48	M-CAC
E38	E-ACT	M49	M-CAG
E38	E-ACT	M50	M-CAT
E38	E-ACT	M61	M-CTG
E38	E-ACT	M62	M-CTT
E39	E-AGA	M49	M-CAG
E39	E-AGA	M50	M-CAT
E39	E-AGA	M60	M-CTC
E40	E-AGC	M47	M-CAA
E40	E-AGC	M59	M-CTA
E40	E-AGC	M61	M-CTG
E40	E-AGC	M62	M-CTT
E41	E-AGG	M47	M-CAA
E41	E-AGG	M48	M-CAC
E41	E-AGG	M49	M-CAG
E41	E-AGG	M61	M-CTG
E41	E-AGG	M62	M-CTT
E42	E-AGT	M49	M-CAG
E45	E-ATG	M59	M-CTA
E45	E-ATG	M60	M-CTC
